# Improving video surveillance systems in banks using deep learning techniques

**DOI:** 10.1038/s41598-023-35190-9

**Published:** 2023-05-16

**Authors:** Mohammad Zahrawi, Khaled Shaalan

**Affiliations:** Faculty of Engineering & IT, British University in Dubai, Dubai, UAE

**Keywords:** Electrical and electronic engineering, Software, Computational science, Information technology

## Abstract

In the contemporary world, security and safety are significant concerns for any country that wants to succeed in tourism, attracting investors, and economics. Manually, guards monitoring 24/7 for robberies or crimes becomes an exhaustive task, and real-time response is essential and helpful for preventing armed robberies at banks, casinos, houses, and ATMs. This paper presents a study based on real-time object detection systems for weapons auto-detection in video surveillance systems. We propose an early weapon detection framework using state-of-the-art, real-time object detection systems such as YOLO and SSD (Single Shot Multi-Box Detector). In addition, we considered closely reducing the number of false alarms in order to employ the model in real-life applications. The model is suitable for indoor surveillance cameras in banks, supermarkets, malls, gas stations, and so forth. The model can be employed as a precautionary system to prevent robberies by implying the model in outdoor surveillance cameras.

## Introduction

Automated surveillance has grown over the past few years due to technological advancements and the development of intelligent video processing techniques. However, the widespread use of handguns has led to an increase in crime rates throughout the world. According to the Small Arms Survey 2017, approximately one billion firearms are in global circulation, and 857 million are in civilian hands^1^. A safe environment is one of the nine pillars of prosperity^2^. Therefore, it is crucial to keep all institutions safe for more prosperity.

Advancements in camera, sensor, and robotic technology have led to the creation of better security tools and, as a result, a rise in their use to safeguard private residences, commercial buildings, and public areas. The use of sensors with ultra-high-resolution cameras in many AI projects allows them to be activated by seeing a specific item rather than just motion. For example, security systems can identify the faces of people who live or work in a home or office space. If a security camera detects the face of an intruder, it could set off an alert system.

Aside from image capture, specific video surveillance systems have many other features. For example, some systems can read and analyze data, including license plates, and compare it to a database. They can also map the movements of individuals and cars and inform the police or a security patrol. In addition, an increasing number of robots now come with incorporated AI software that is set up to scan or "patrol" routes, check their surroundings, detect hazards of unauthorized entrance from people or vehicles, and send alerts.

Deep learning plays a crucial role in enhancing security control systems^3^. Deep learning utilizes layers of non-linear processing elements for feature extraction and conversion^4^. For example, a pixel in an image can be thought of as a vector of density values; pixels form a feature, where a feature is a cluster of custom shapes such as corners, edges, and roundness^5^. The simple design of the deep learning model is the convolutional neural network (CNN), which includes convolution filters, pooling, activation function, dropout, fully connected, and classification layers^6^. In addition, there are various methods for object detection using deep learning, including Faster R-CNN, You Only Look Once (YOLO), and Single Shot Detection (SSD).

Nowadays, most robbers use handguns to commit crimes^7^. Several studies have shown that handgun weapons are the most popular criminal tools used for crimes^8^, such as robbery, illegal hunting, and terrorism. The proposed solution to stop such illegal activities is to install video surveillance systems incorporated with artificial intelligence techniques^9,10^. So, the security guards can take prompt action in the early stages^7^.

## Related work

In the realm of computer vision, many object detection techniques were put forth to enhance surveillance systems. Many fields, including anomaly detection, self-driving cars, person detection, and traffic monitoring, have employed object detection models^11^. In a brief discussion, R. Chellapa et al. examined the deployment of object detection models in video surveillance cameras^12^. In 2007, L. Ward et al. presented for the first time the notion of identifying firearms using real-time object detection techniques^13^.

Furthermore, it should also be highlighted that there are other promising methods for identifying people with dangerous weapons. According to research by Yong et al., microwave swept-frequency radar can be used to detect metal objects, such as weapons and knives^14^. Also, authors used a YOLOv3 model combined with a human pose to detect handguns^15^. An approach that combines color-based segmentation and an interest point detector has been utilized to automatically identify weapons. The purpose is to detect objects and analyze their features in order to compare them to descriptions of guns^16^.

Moreover, Olmos et al. has suggested techniques to reduce the number of false positives and false negatives, such as employing a symmetric dual camera system to enhance the selection of potential features, thereby boosting the model's accuracy^17^. The study^18^ presents different transfer learning models, namely AlexNet, VGG16, and VGG19, used to train.gun and knife detection models.

A study on the detection of firearms and knives presented models that inform security guards when handguns or knives are identified in video surveillance systems^17^. Another study demonstrates a hybrid weapon detection model designed to identify guns and knives; it uses fuzzy logic and other parameters to enhance the result and decrease false alarms^18^.

## Methodology

**Figure 1:**
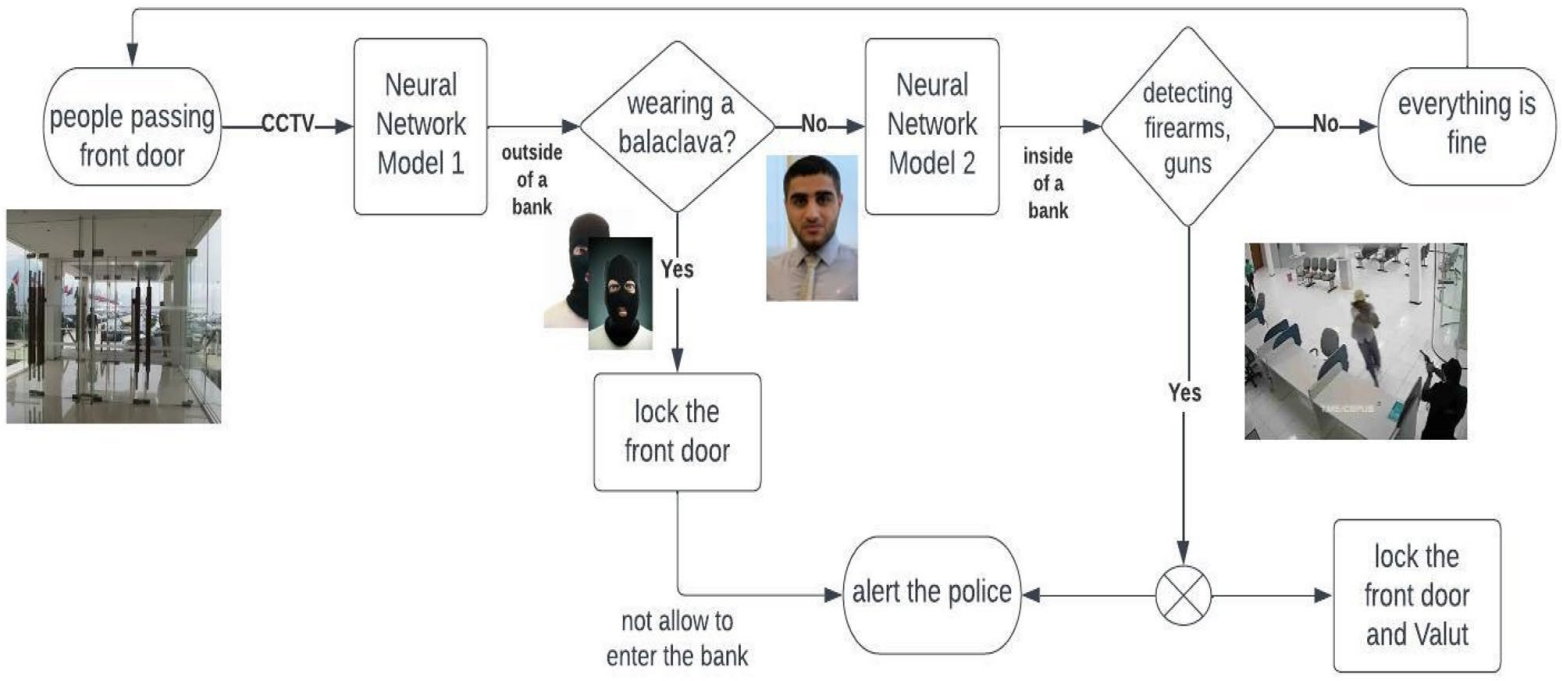
Conceptual framework for the proposed project.

It is essential to introduce a conceptual framework. This entails understanding the problem, the trade-off between time response and model performance, and pointing out deficiencies in the proposed model including data quality. Figure 1 presents conceptual framework approach for the proposed project, showing the location of each neural network models.

**Figure 2:**
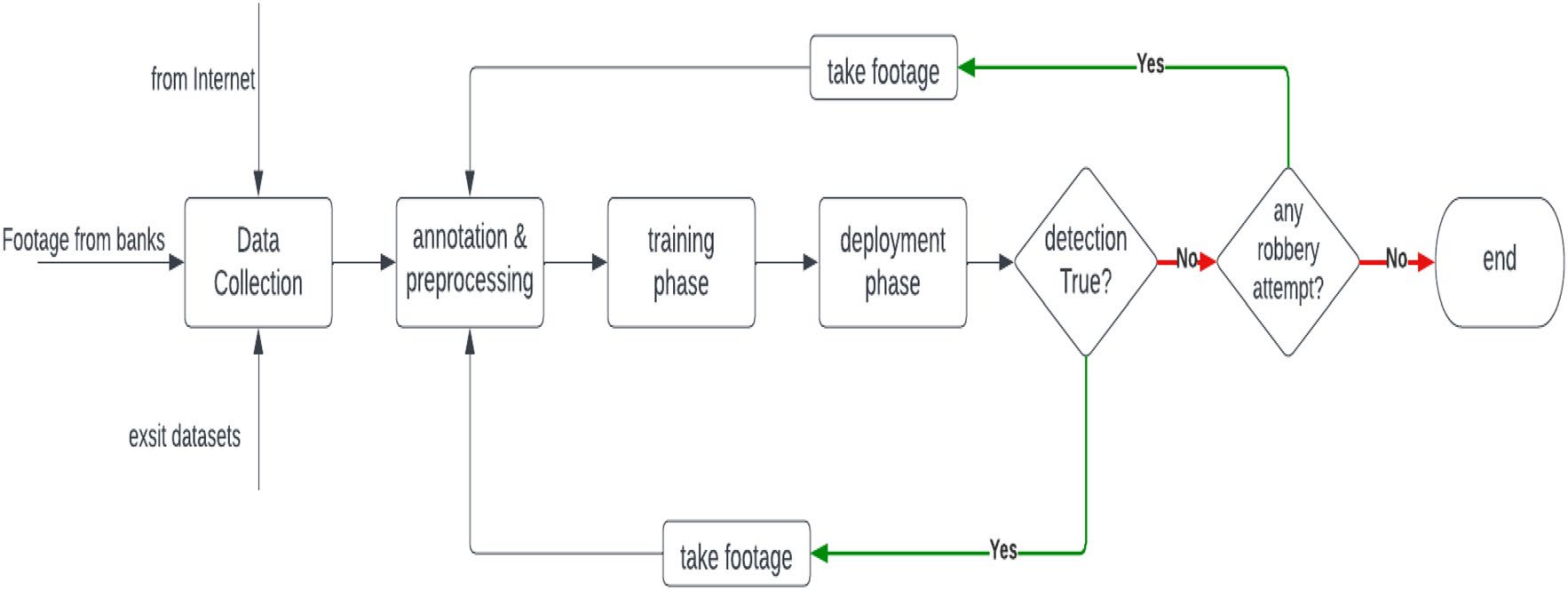
Flowchart of training and developing weapon detection model.

In this research, we are looking to train two models using neural networks, the first model to allow people with faces uncovered to pass the front gate, and the second model to detect any dangerous items that indicate criminals or robberies inside the bank. The process of detecting balaclava and weapons can be broken down into two main parts, locating the bounding box of the target object (e.g. weapon) and classifying it. Our approach for weapon detection is applying transfer learning using STATE-OF-THE-ART object detection models such as YOLO and SSD. Figure 2 shows a flowchart of training and developing proposed neural network models.

However, we build five weapon detection models by using transfer learning and fine-tuning from pre-trained models. The models are SSD Inception V2, SSD MobileNet V2, SSD ResNet50 V1, YOLOv4, and YOLOv5. All models were trained on a Colab notebook using TensorFlow and GPU accelerated. Starting with SSD Inception V2 models, it uses VGG-16 as a feature extractor, the batch size is 32, and the input layer receives images of resolution 300 × 300. One of the solutions to decrease time response that trains a model on grayscale data, therefore, it yields a lightweight model that is appropriate for CCTV cameras.

## Results

In surveillance applications, it is expected that the input images are of relatively high quality^19^. Processing time and high precision are essential factors for evaluating real-time weapon detectors. Accurate, relevant, and high-quality images are crucial in building and developing any computer vision model. Research has been conducted on armed robbery and states that the most frequent weapon used was firearms^20^.

### Datasets

Our study only focuses on four classes: pistol, knife, rifle, and robber mask. We include revolvers and handguns in pistol class. Also, we include shotguns in rifle class. However, many objects are most likely to be confused with a pistol, such as wallets, cell phones, selfie sticks, money, and so forth. So, it would be a great idea to label them as a non-weapon class, hence reducing the number of false positives and false negatives, therefore increasing the overall accuracy. We have made two datasets, which are explained below.

**Figure 3:**
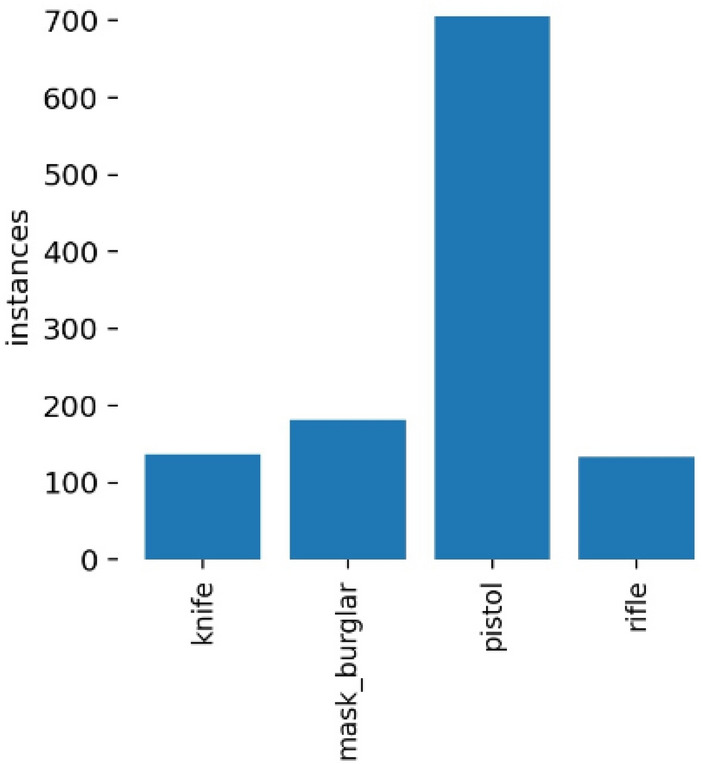
Class distribution in dataset 1.

**Figure 4:**
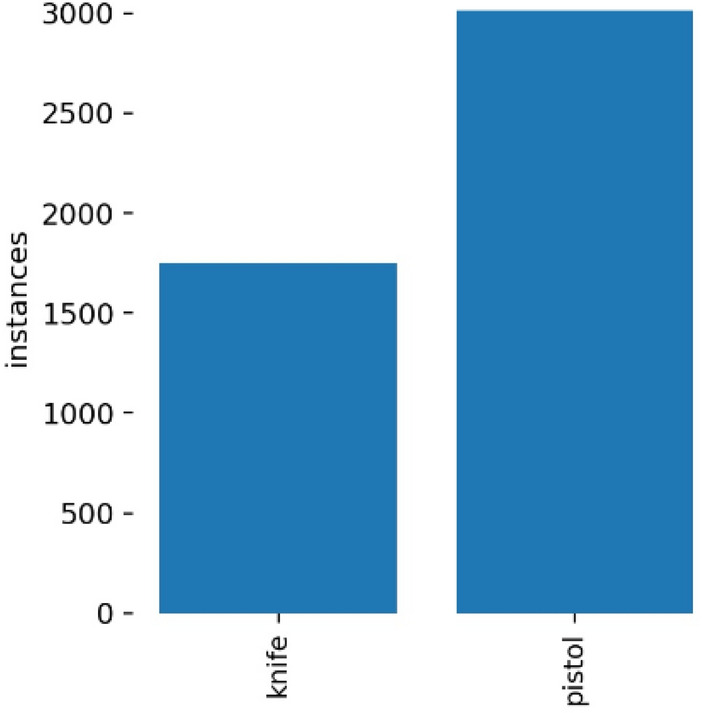
Class distribution in dataset 2.

Dataset 1 consists of four classes: pistol, knife, rifle, and robber mask. We have 1160 images in total. The majority of images belong to the pistol class because nearly 95% of weapons used in robberies are either pistols or revolvers. Datasets were separated into train and test with split size shown in Table 1. Dataset 2 is used to build a weapon detection binary classifier, so two classes were made, knife and pistol. There are 5000 images, with 4250 images in the train set and 750 in the test set. Figure 3 shows the distribution of the four classes in dataset 1, which is a severely imbalanced dataset due to the high number of pistol images. Meanwhile, a distribution of two classes in dataset 2 is shown in Fig. 4.

**Table 1:** Data distribution.

No	Category	Total data	Training data	Test data	Split size
1	Dataset 1	1160	920	240	79%
2	Dataset 2	5000	4250	750	85%

### Training results

In this research, we trained weapon detection models by using transfer learning and fine-tuning from pre-trained models. The models are SSD Inception V2, SSD MobileNet V2, SSD ResNet50 V1, YOLOv4, and YOLOv5. All models were trained on a Colab notebook using TensorFlow and GPU accelerated. Starting with SSD Inception V2 models, it uses VGG-16 as a feature extractor, the batch size is 32, and the input layer receives images of resolution 300 × 300. The num_step parameter is 10,000 and defines how many training steps it will run. The model uses RMSprop as an optimizer designed for deep neural networks.

**Figure 5:**
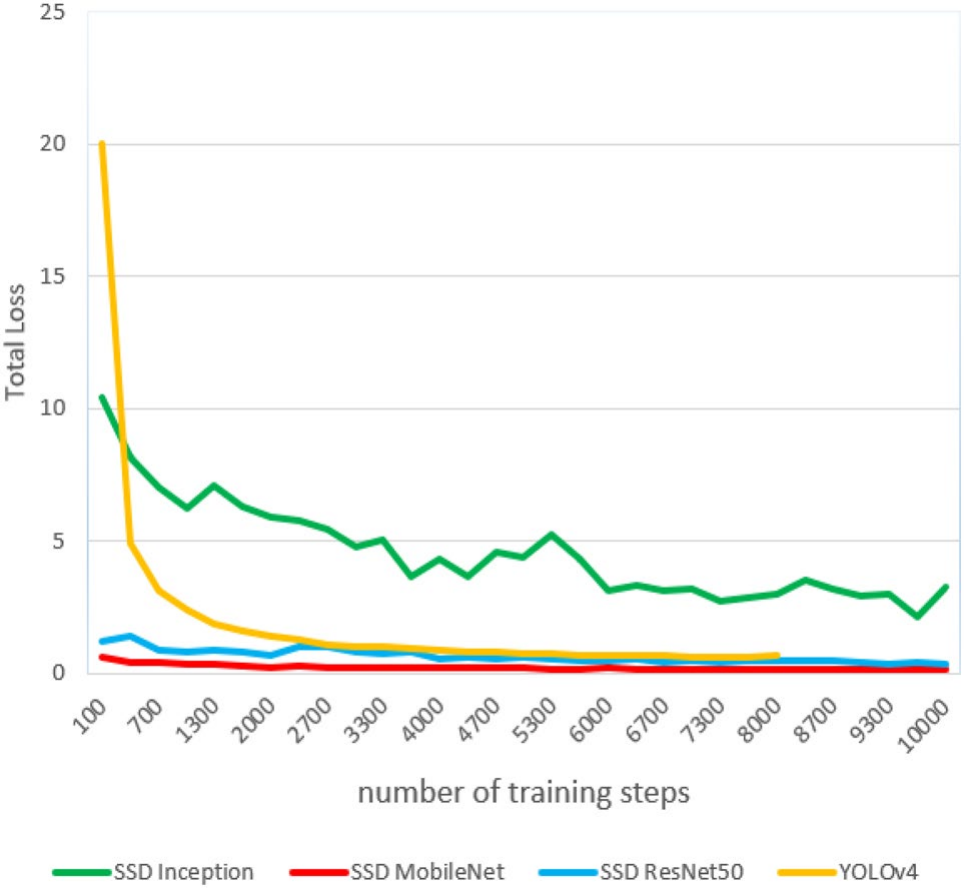
Training results: total loss of the weapon detectors on dataset 1.

**Figure 6:**
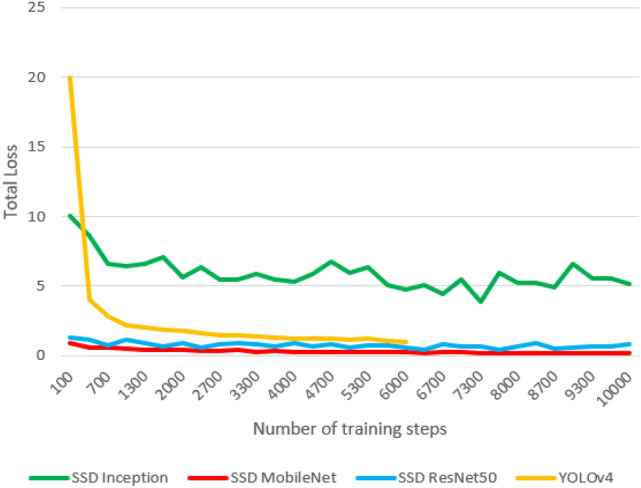
Training results: total loss of the weapon detectors on dataset 2.

The training was performed with different training steps for each model; the SSD Inception, SSD MobilNet, and SSD ResNet models were trained for 10,000 training steps, with batch sizes ranging from 8 to 32, depending on the dimensions of the input images. Meanwhile, YOLOv4 has trained for 6000–8000 training steps only to prevent the overfitting problem. YOLOv5 was trained on dataset 1 for only 270 epochs and dataset 2 for 832 epochs since no improvement was observed in the last 100 epochs. Training results are shown below in Figs. 5 and 6.

The behavior of total loss drops in models for dataset 1 and dataset 2 is quite similar. As the loss starts to decrease through training, the model's accuracy increases until it stagnates. After 10,000 training steps, the value of the total loss of the SSD MobileNet model on dataset 1 is around 0.16, and the learning rate has vanished to zero. However, the model with the highest total loss is SSD Inception in datasets 1 and 2.

**Figure 7:**
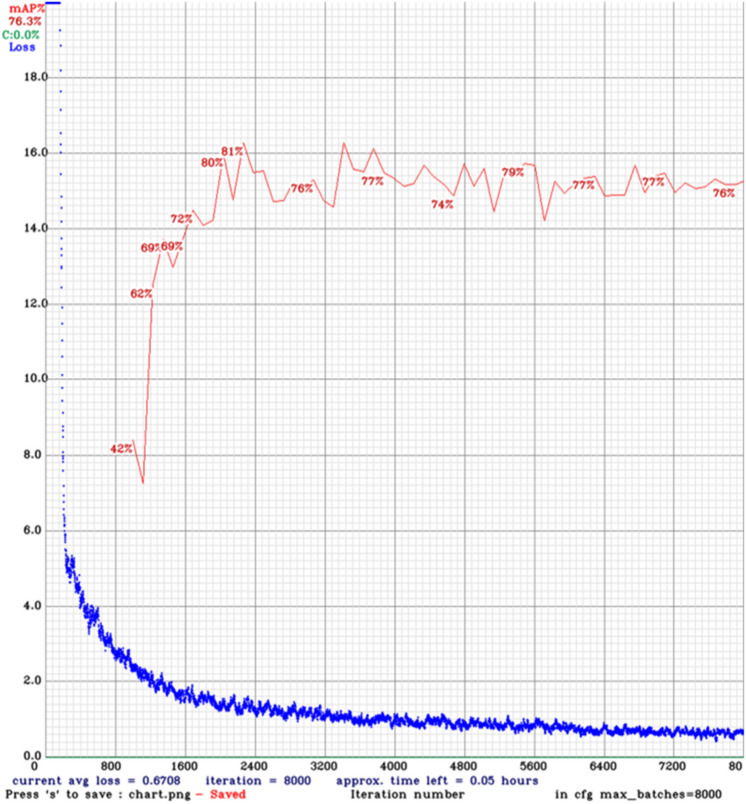
Chart of loss and mAP from YOLOv4 model training on dataset 1.

**Figure 8:**
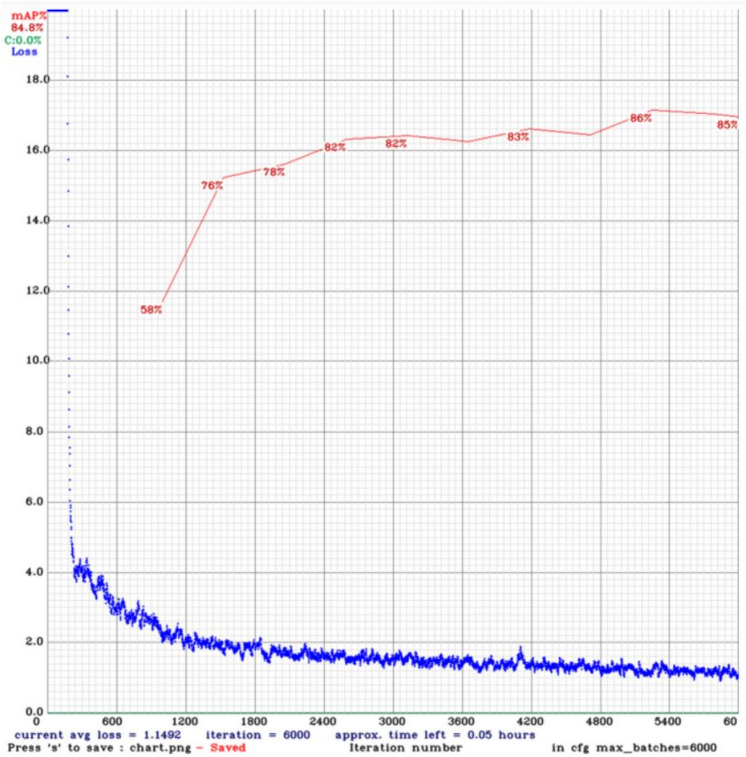
Chart of loss and mAP from YOLOv4 model training on dataset 2.

The total loss is the sum of classification loss, localization loss, and regularization loss in SSD models. However, the loss function for YOLO models composes of classification loss, localization loss, and confidence loss (objectness loss). Figures 7 and 8 show the loss function and mAP chart for the YOLOv4 model throughout the training phase on datasets 1 and 2, respectively.

The blue curve represents the training loss, which decreases as the number of iterations increases. The red line is the mean average precision when the intersection-over-union threshold is 0.5. The most critical hyperparameter while configuring a neural network is the learning rate, which determines how much to change the model based on the estimated error every time the model weights are updated.

**Figure 9:**
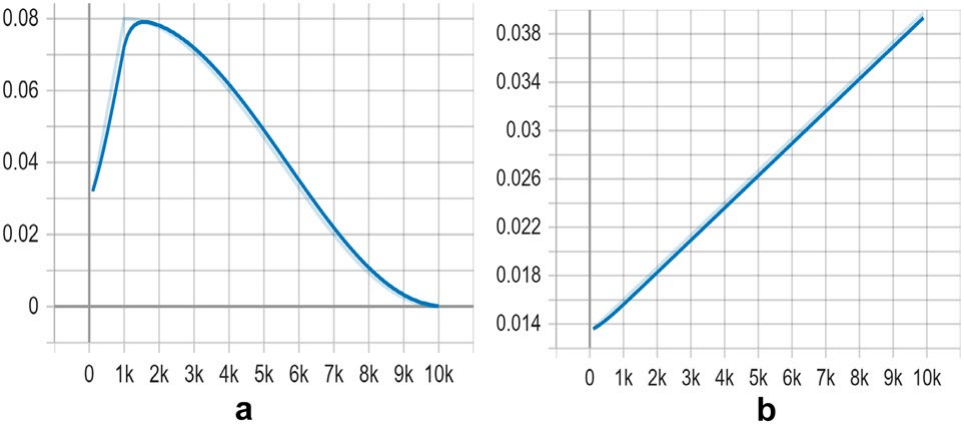
(**a**) Behavior of learning rate for SSD MobileNet model. (**b**) Behavior of learning rate for SSD ResNet50 model.

Figure 9 shows the learning rate behavior for the SSD MobileNet model on dataset 1 and the SSD ResNet50 model on dataset 2.

The images used in building weapon detectors should be cleaned, preprocessed, and appropriately annotated to achieve high precision. Unfortunately, the process of collecting images and labeling them is delicate and tough.

### Evaluation results

Test data was created to assess the accuracy of the newly weapon detection models, where images represent various weapons in different scenarios. This section presents the evaluation results for all experiments in tables and graphical representations.

All object detection models were evaluated on mAP@0.5 (Pascal VOC), mAP@0.5:0.95 (COCO), and mAP@0.75 (strict metric). Tables 2 and 3 show evaluation results for dataset 1 and dataset 2, respectively. Our primary metric used to compare the models is AP@IoU = 0.5:0.95, which is the average precision for IoU thresholds from 0.5 to 0.95 with a step size of 0.05.

**Table 2:** Evaluation results on dataset 1.

Metric/model	area	Max detection	SSD Inception V2	SSD MobileNet	SSD ResNet 50	YOLOv4	YOLOv5
AP@IoU = 0.5:0.95	All	100	0.215	0.469	0.364	–	–
**AP@IoU = 0.5**	**All**	**100**	**0.375**	**0.717**	**0.577**	**0.795**	**0.724**
AP@IoU = 0.75	All	100	0.206	0.529	0.392	–	–
AP@IoU = 0.5:0.95	Small	100	0.001	0.247	0.201	–	–
AP@IoU = 0.5:0.95	Medium	100	0.087	0.311	0.243	–	–
AP@IoU = 0.5:0.95	Large	100	0.254	0.511	0.397	–	–
AR@IoU = 0.5:0.95	All	1	0.261	0.488	0.399	–	–
AR@IoU = 0.5:0.95	All	10	0.329	0.574	0.523	–	–
AR@IoU = 0.5:0.95	All	100	0.361	0.581	0.548	–	–
AR@IoU = 0.5:0.95	Small	100	0.033	0.256	0.344	–	–
AR@IoU = 0.5:0.95	Medium	100	0.255	0.367	0.414	–	–
AR@IoU = 0.5:0.95	Large	100	0.400	0.636	0.584	–	–

Significant values are in bold.

**Table 3:** Evaluation results on dataset 2.

Metric/model	area	Max Detection	SSD Inception V2	SSD MobileNet	SSD ResNet 50	YOLOv4	YOLOv5
AP@IoU = 0.5:0.95	All	100	0.177	0.411	0.247	–	–
**AP@IoU = 0.5**	**All**	**100**	**0.279**	**0.674**	**0.406**	**0.771**	**0.82**
AP@IoU = 0.75	All	100	0.193	0.384	0.251	–	–
AP@IoU = 0.5:0.95	Small	100	0	0.015	0.001	–	–
AP@IoU = 0.5:0.95	Medium	100	0.013	0.148	0.060	–	–
AP@IoU = 0.5:0.95	Large	100	0.213	0.479	0.292	–	–
AR@IoU = 0.5:0.95	All	1	0.195	0.436	0.285	–	–
AR@IoU = 0.5:0.95	All	10	0.237	0.499	0.389	–	–
AR@IoU = 0.5:0.95	All	100	0.268	0.534	0.428	–	–
AR@IoU = 0.5:0.95	Small	100	0.000	0.114	0.050	–	–
AR@IoU = 0.5:0.95	Medium	100	0.055	0.351	0.233	–	–
AR@IoU = 0.5:0.95	Large	100	0.32	0.584	0.479	–	–

Significant values are in bold.

A few more evaluation metrics have been used in this paper, like AP for evaluating detection for different weapon sizes inside the image and determining whether the model is suitable for only large weapons, small weapons, or both. SSD models are evaluated on three different sizes, small, medium, and large, where small objects have area an (h*w) less than 32^2^ pixel scale, medium objects have an area more than 32^2^ and less than 96^2^ pixels, and large objects have an area more than 96^2^ pixels. However, the average precision for IoU threshold of 0.5 (AP@IoU = 0.5) is used to evaluate weapon detection models based on YOLO algorithms. The average recall metric is also used in the evaluation, given a fixed number of detections per image.

**Figure 10:**
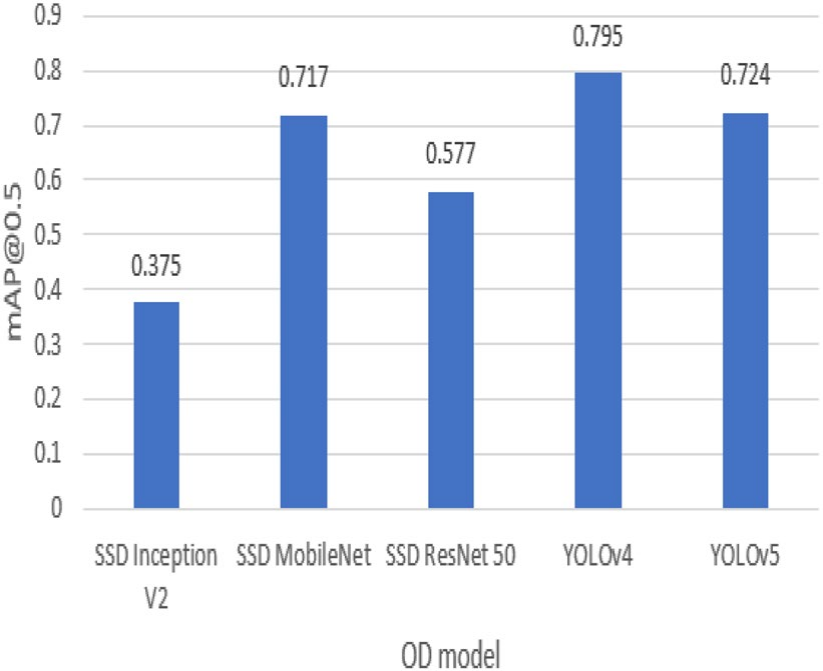
Weapon detection models comparison on dataset 1 using mAP@0.5 metric.

**Figure 11:**
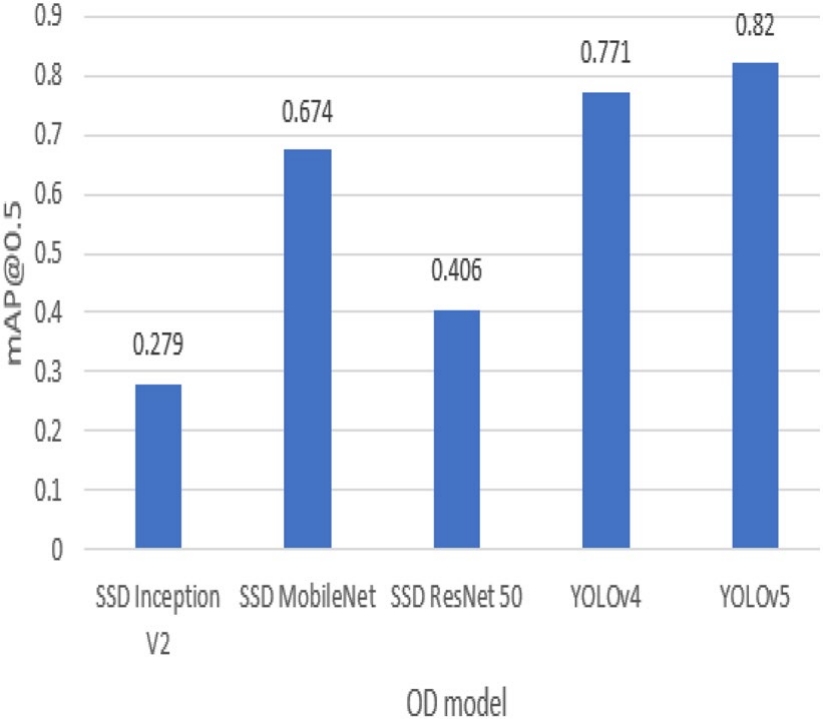
Weapon detection models comparison on dataset 2 using mAP@0.5 metric.

According to Tables 2 and 3, the average precision (AP) of the SSD MobileNet model surpasses the other two SSD models in detecting small weapons for datasets 1 and 2. For the average recall (AR) metric, the SSD ResNet50 model performs better than the SSD MobileNet and SSD Inception models in detecting small weapons on dataset 1. In contrast, the SSD MobileNet model performs better on dataset 2. However, for detecting medium and large objects, SSD MobileNet achieves better performance than other SSD models in both datasets 1 and 2. The primary metric used in comparing weapon detection models is AP@IoU = 0.5; by comparing the models, it is obvious that YOLOv4 surpasses other models with AP 0.795 on dataset 1, while the least efficient model is SSD Inception with AP around 0.375. In contrast with dataset 2, YOLOv5s is the best model for weapon detection with an average precision of 0.82, and the lowest-performing model is SSD Inception with an AP of 0.279. Figures 10 and 11 show detection model comparisons on datasets 1 and 2, respectively.

As mentioned previously, there are different model sizes for YOLOv5. In this paper, we trained both datasets on YOLOv5s, where “s” stands for a small-size model. Meanwhile, the x-large YOLOv5 model (YOLOv5x) achieves higher performance than YOLOv5s, with a mAP around 0.762 on dataset 1.

### Confusion matrix

**Figure 12:**
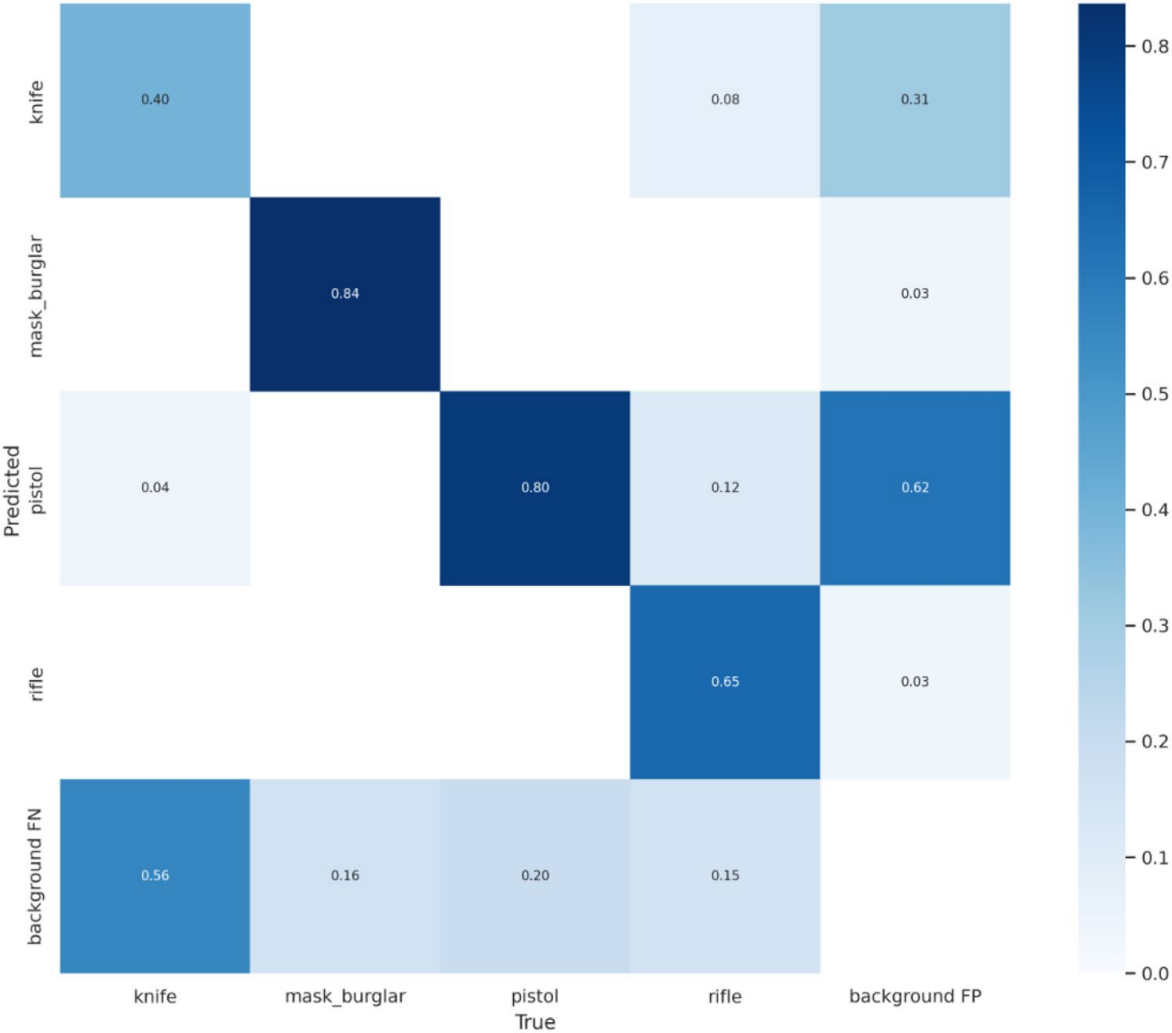
Confusion matrix of weapon detection for YOLOv5 on dataset 1.

**Figure 13:**
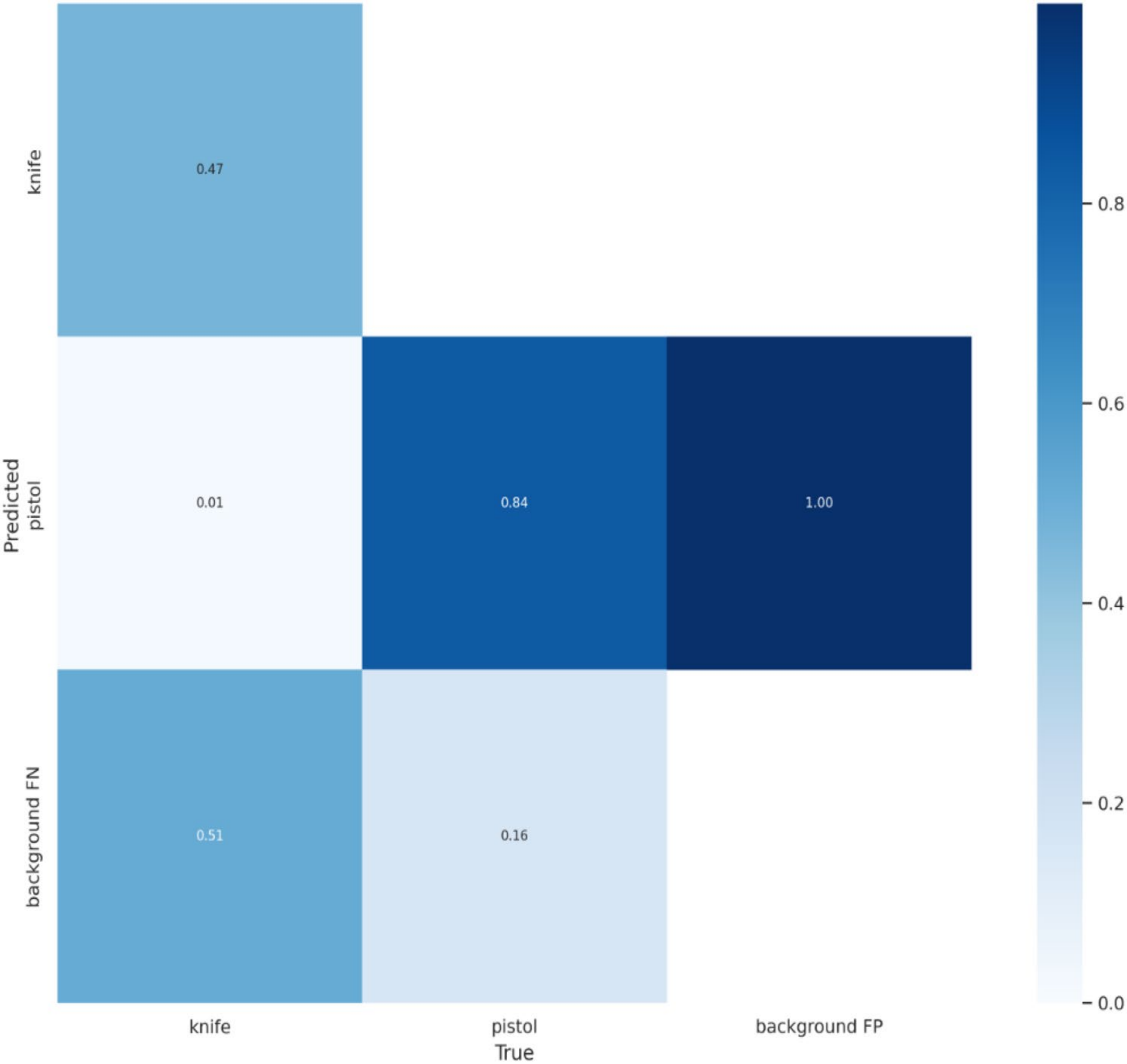
Confusion matrix of weapon detection for YOLOv5 on dataset 2.

Dataset 1 contains 240 test images with four classes: knife, mask burglar, pistol, and rifle. Moreover, dataset 2 contains 750 test images with two classes, pistol and knife. The confusion matrix in Fig. 12 shows that the mask burglar class had the highest rate of successful weapon detection (0.84), while the knife class had the lowest rate (0.4). Similarly, the most successful weapon detection rates as shown in confusion matrix in Fig. 13 were obtained for the pistol class at 0.84, and the least successful rates were for the knife class at 0.47.

It can be seen that around half of the knife weapons in both datasets are confused with the background. Also, around a fifth of the pistol images in both datasets was misclassified as background. In addition, 0.12 of rifle images were misclassified as pistol class in dataset 1. In comparison, 0.08 of rifle images were misclassified as knife images, and 0.04 of knife images were misclassified as pistol images.

**Figure 14:**
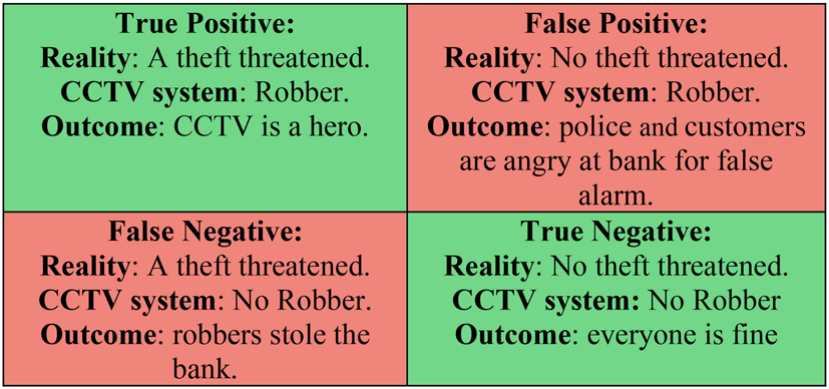
Understanding Confusion Matrix.

In the confusion matrix, background FP indicates the model may think detection is a weapon when it is not, and background FN indicates the model misses detecting real weapons. By looking at previous confusion matrices, background FP refers to the model incorrectly predicting the weapons, and background FN refers to the model incorrectly predicting the negative class. Therefore, we can summarize our weapon detection model in the bank using a 2 × 2 confusion matrix in Fig. 14 that depicts all four possible outcomes.

### Recall × precision

**Figure 15:**
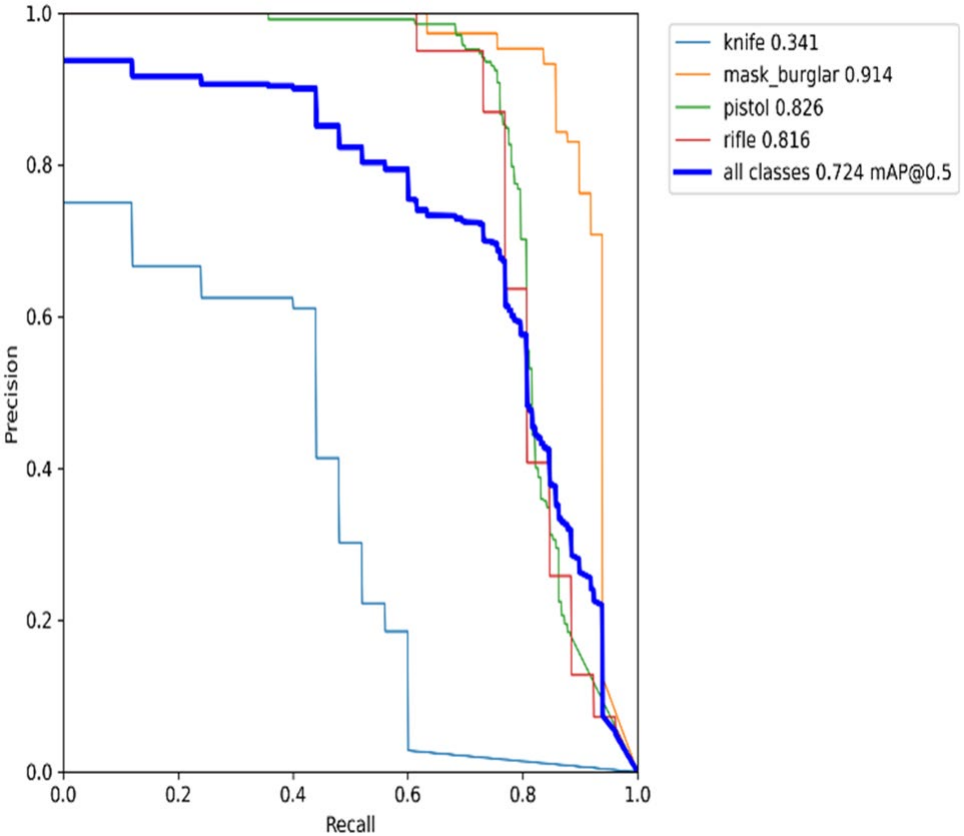
Precision recall graph for YOLOv5s on dataset 1.

**Figure 16:**
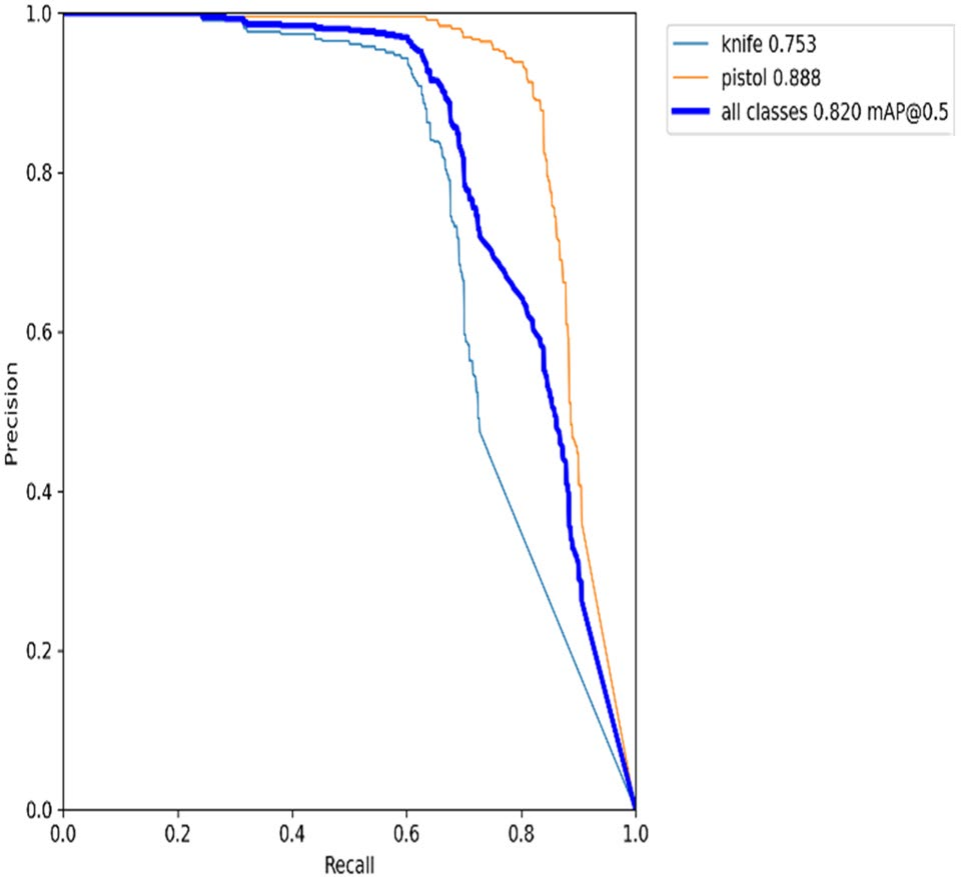
Precision recall graph for YOLOv5s on dataset 2.

The precision-recall curve depicts the trade-off between precision and recall for different classes. A broad area under the curve indicates high recall and precision. A high recall is linked to a low false negative rate, but high accuracy is linked to a low false positive rate. The precision-recall graph for the YOLOv5s model on datasets 1 and 2 is shown in Figs. 15 and 16, respectively.

According to Fig. 15, it can be seen that the model is returning accurate results for detecting burglar mask items, with a mAP of 0.914. The model also performs quite well-detecting pistols and rifles, with a mAP of around 0.82. Meanwhile, the model poorly detects knife items with mAP 0.341 in real-time. Moving on to Fig. 16, it is evident that the performance model of detecting pistol items is higher than detecting knife class due to the quality of knife images in the dataset.

### F1 × confidence

**Figure 17:**
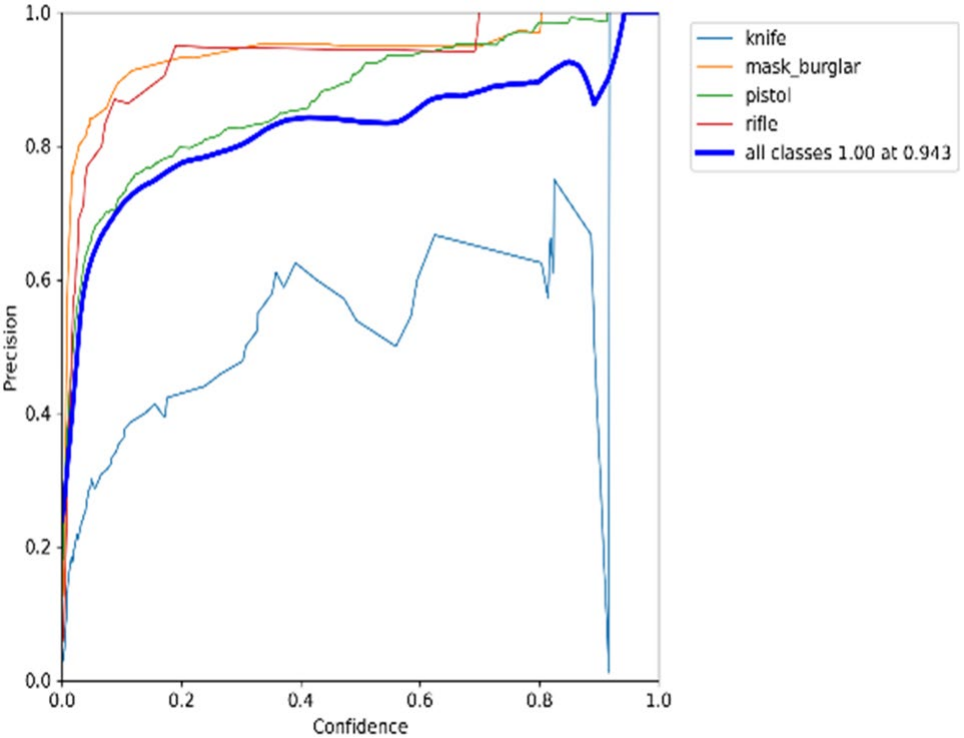
Trade-off between precision and confidence for YOLOv5 model on dataset 1.

**Figure 18:**
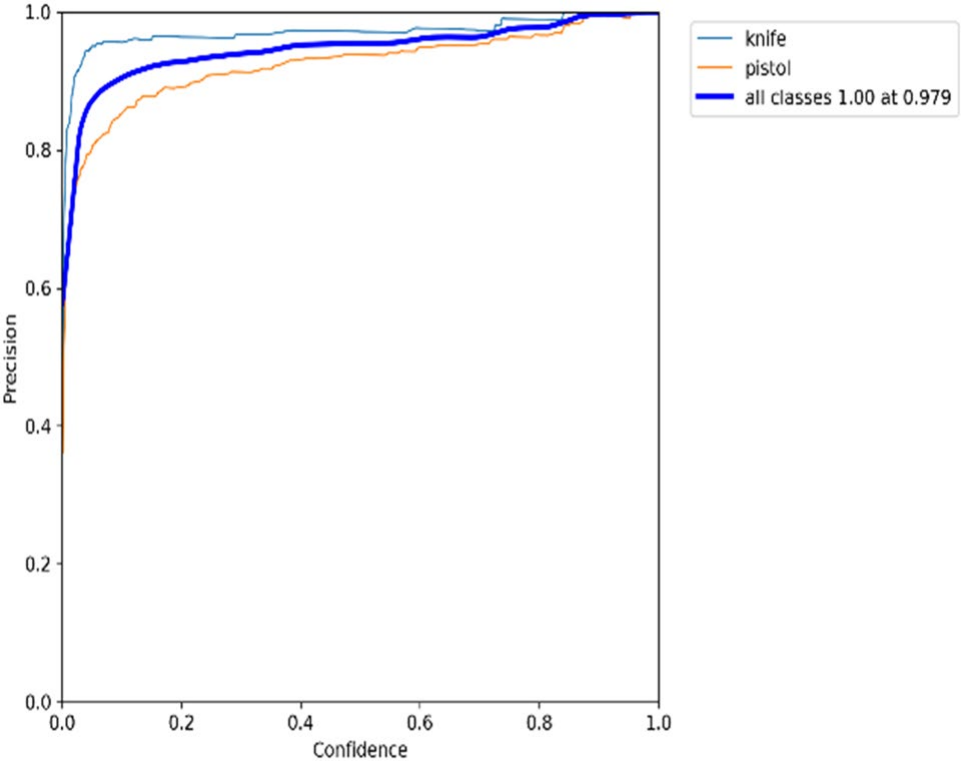
Trade-off between precision and confidence for YOLOv5 model on dataset 2.

The confidence score shows how probable it is that the bounding box contains the target object and how confident the weapon detection model is about it. The confidence score will be zero when no weapon items are detected in the bounding box. As a reminder, the IoU value can be obtained by dividing the shared area between the predicted bounding box and the ground-truth bounding box by the area of their union. The match is perfect when the ground truth and predicted bounding boxes have the same area and location. The precision-confidence curves for the YOLOv5 model on datasets 1 and 2, respectively, are shown in Figs. 17 and 18.

**Figure 19:**
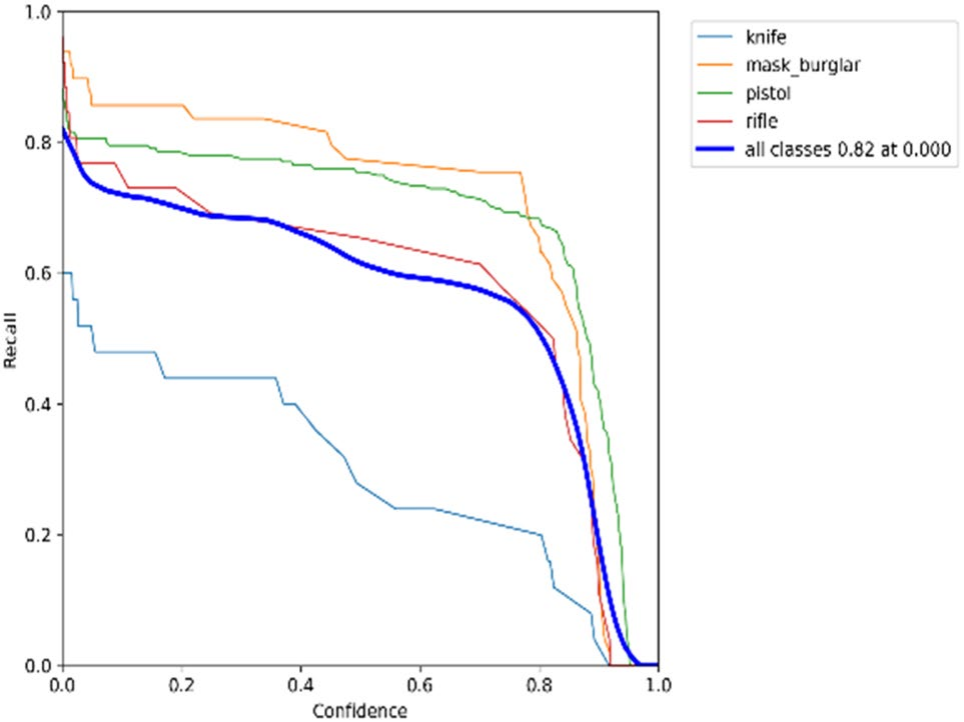
Trade-off between recall and confidence for YOLOv5 model on dataset 1.

**Figure 20:**
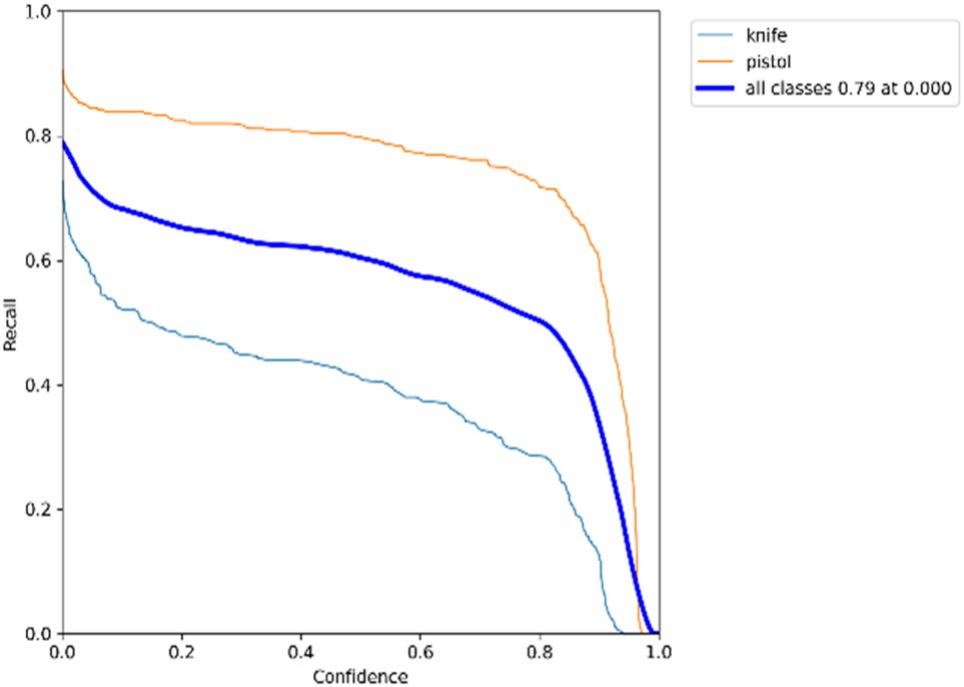
Trade-off between recall and confidence for YOLOv5 model on dataset 2.

Recall-confidence curves for the YOLOv5 model on datasets 1 and 2 are displayed in Figs. 19 and 20, respectively.

**Figure 21:**
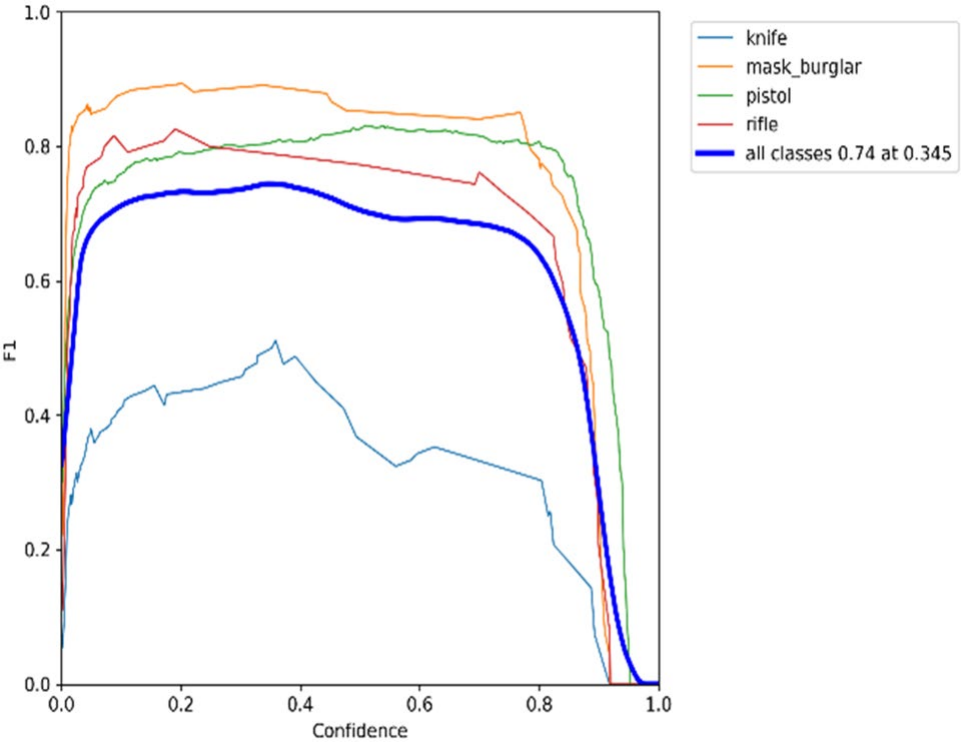
F1-Confidence curve presents best F1 score of 0.74 with a confidence threshold of 0.345 on dataset 1.

**Figure 22:**
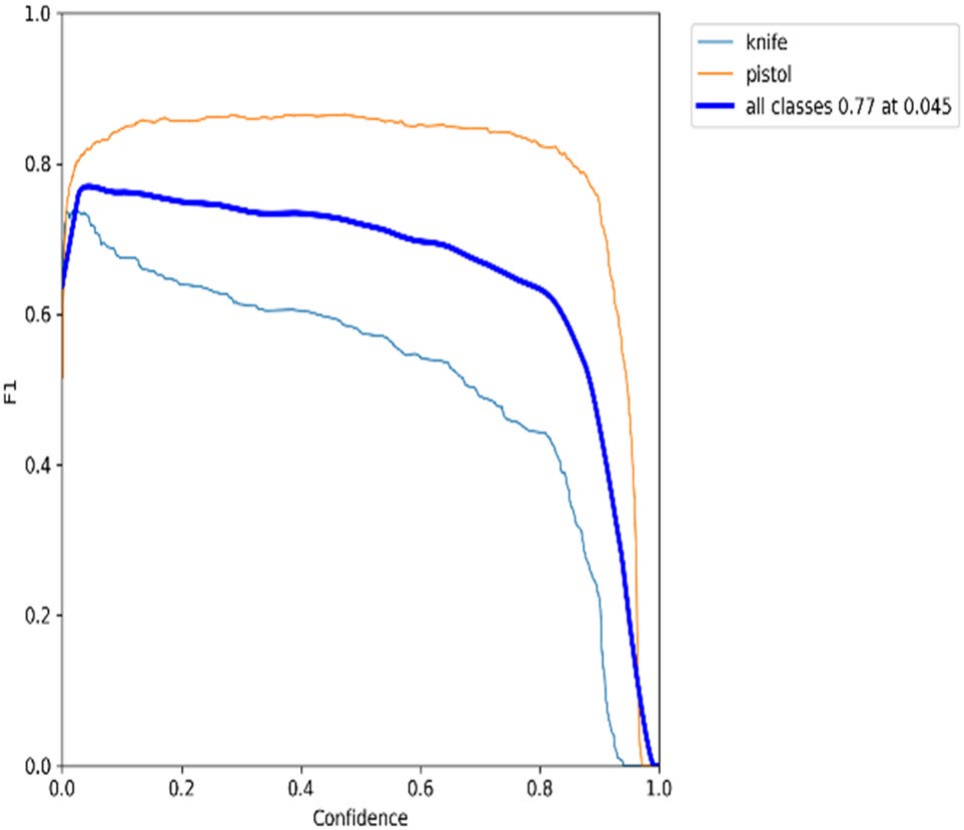
F1-Confidence curve presents best F1 score of 0.77 with a confidence threshold of 0.045 on dataset 2.

A bounding box, class, and confidence score represent the outcome of an object detector. Figures 17 and 18 show that precision increases when the confidence score increases while recall decreases. The detection is considered valid (positive) when its confidence is higher than a confidence threshold. Otherwise, it is a negative detection. The number of TPs and FPs decreases when the confidence threshold increases. Conversely, the recall measure decreased as the confidence threshold increased due to an increase in false negatives. For example, according to the F1-confidence curve shown in Fig. 21, the confidence value that optimizes the precision and recall is 0.345 for the YOLOv5 model on dataset 1, and the best F1 score for all classes is 0.74. Similarly, to Fig. 22, the best confidence value that optimizes precision and recall is 0.045 for the same model on dataset 2, and the best F1 score is 0.77.

## Discussion

Weapon detection models were trained using several configurations based on model architecture. Many factors have an impact on model performance and results, such as different image resolutions, batch sizes, training steps, rescaling, and data augmentation options. The ideal parameters for each model should be investigated using a hyperparameter optimization pipeline in order to balance out or improve the results of weapon detectors.

The evaluation part is next; weapon detectors are evaluated based on the model's primary purpose. For example, if we want to use the proposed model as a weapon recognizer without considering box overlap, In that case, we can merely minimize the IoU threshold to close to zero while keeping the number of false negatives low and false positives high. In other words, the recall score will be high and the precision low. However, in the case of building a weapon detector instead of a weapon recognizer, we consider the location of a predicted box. Therefore, it would be recommended to evaluate the model using the COCO evaluation metric (AP@IoU = 0.5:0.95). This metric describes how the model works in the area of overlapping that we are most interested in.

**Figure 23:**
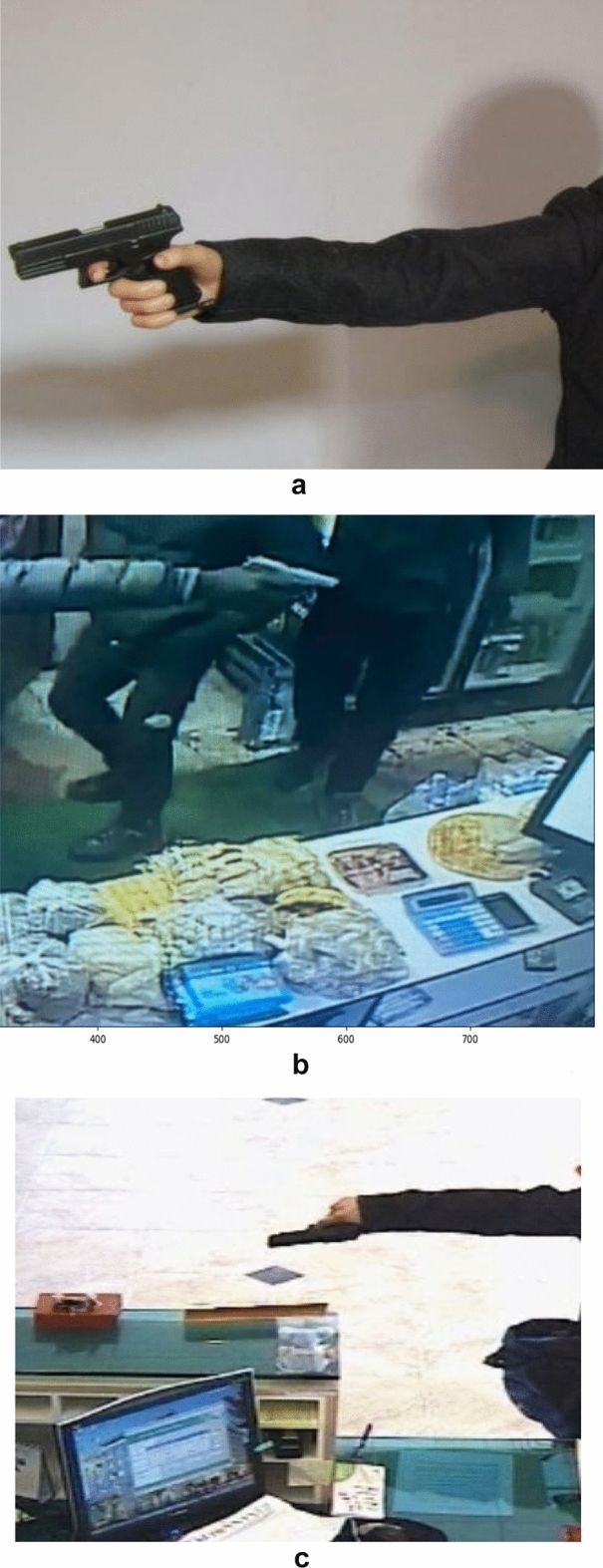
(**a**) Object detection model could not find the pistol because of low resolution of image. (**b**) Object detection model could not find the pistol because of small size of pistol. (**c**). object detection model could not find the pistol because of low resolution of image.

**Figure 24:**
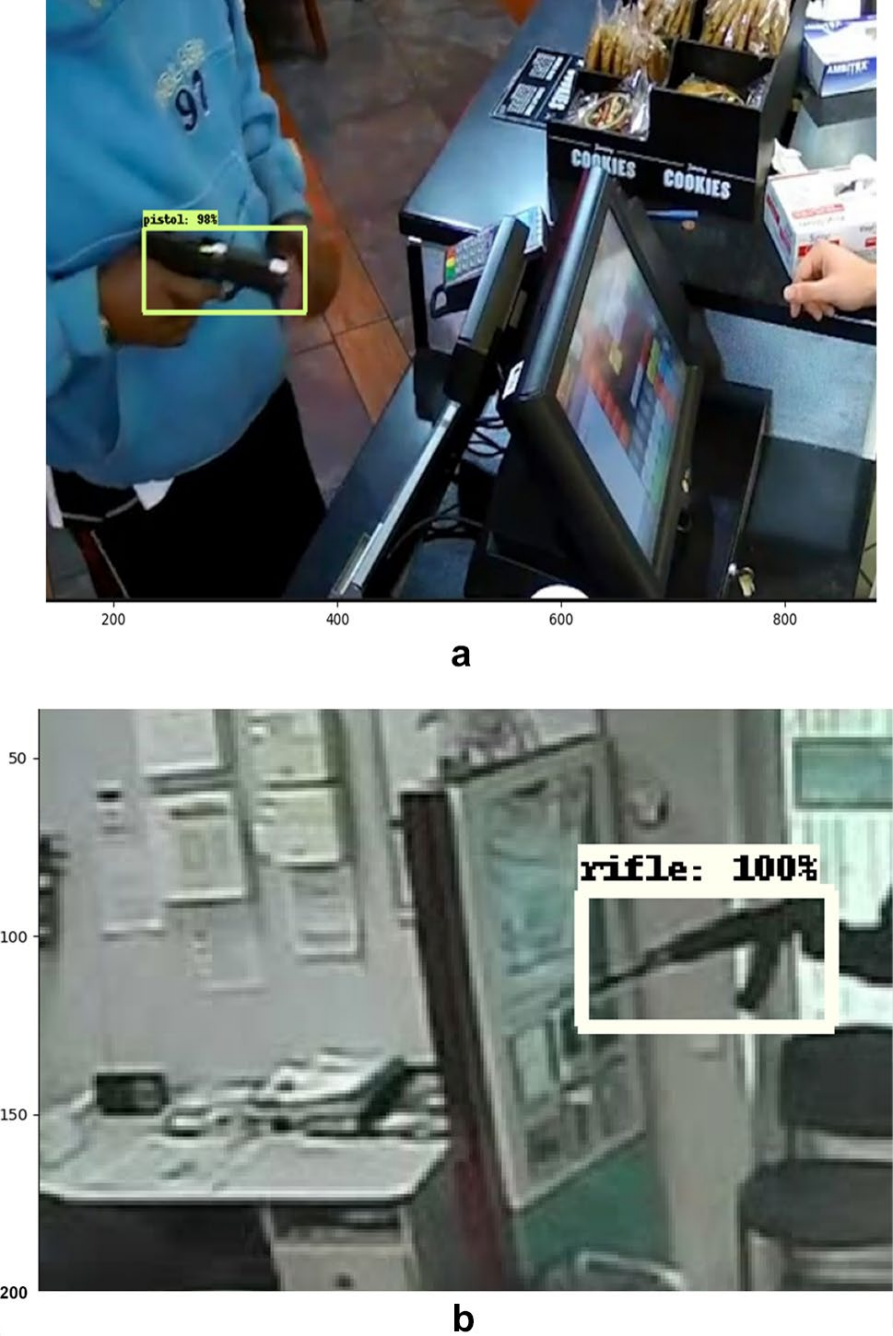
(**a**) Detection Results of weapon detection model on sample images were taken from CCTV camera. (**b**) Detection Results of weapon detection model on sample images were taken from CCTV camera.

Different models and classes all need a certain confidence threshold to boost their performance and thus maximize it. Figures 17, 18, 19 and 20 offer a tradeoff between confidence, precision, and recall to find the best confidence threshold for each class and model. It is hard to select a model based on a single metric. Thus, many metrics were used in the evaluation process. The proposed models fail to detect weapon items in images, as shown in Fig. 23. Figure 24 shows weapon detection results of footages from monitoring cameras in real life scenarios.

Generally, low-quality images are one of the core problems of deep learning models. For example, almost all the CCTV footage in our dataset is grainy, blurry, monochromatic, and of poor quality. The main reason CCTV footage is of low quality is that the amount of data required to record at higher quality would be enormous; hence, more data storage is needed. In addition, outdated cameras, a weak internet connection, and poor lens conditions are factors responsible for low-quality images.

Besides poor-quality images, many challenges appear regarding the quality of the dataset. One of these challenges is background clutter, where the target object may blend into the environment, making it difficult to identify. Also, occlusion issue when a small portion of the target object is visible. Furthermore, viewpoint variation challenge where a target object can be rotated in any direction with respect to the observer or camera. Lastly, scale variation occurs when an object appears in images of different sizes.

## Conclusion

To wrap up the results and come to a conclusion, the most suitable weapon detector for the task would be YOLOv4 for dataset 1 and YOLOv5s for dataset 2. This is because YOLO models perform better than SSD models in terms of detection speed and average precision. In addition, YOLO models are better options when the weapon size is small. This work studied several weapon detection models (SSD and YOLO) applied to the video surveillance system. The two main objectives of the study were to compare the performance of five different models (SSD Inception, SSD MobileNet, SSD ResNet50, YOLOv4, and YOLOv5) and to examine the improvement of the detection quality by training on two datasets with different classes and number of images (Supplementary files 1, 2).

The evaluation of the results in the five experiments was carried out on 240 test images in dataset 1 and 750 test images in dataset 2. According to the results in Chapter 4, we find that those weapon detectors using YOLOv4 outperform YOLOv5 and SSD models on dataset 1 with a mean average precision of 0.795. Meanwhile, YOLOv5 is the best option for dataset 2, with a mean average precision of around 0.82. Finally, unlike pistols in both datasets, the model's performance is poor in detecting knife items.

## Supplementary Information

Supplementary Information 1.

Supplementary Information 2.

## Data Availability

All datasets can be downloaded from the following GitHub repositor. https://github.com/Mohammad-H-Zahrawi/Projects/tree/main/Weapon%20Detection.
