# Supplementary figures and images for: Improving video surveillance systems in banks using deep learning techniques (part 2 of 4)
Source: Sci Rep. 2023 May 16;13:7911. doi: 10.1038/s41598-023-35190-9 (PMC10188611; doi:10.1038/s41598-023-35190-9)

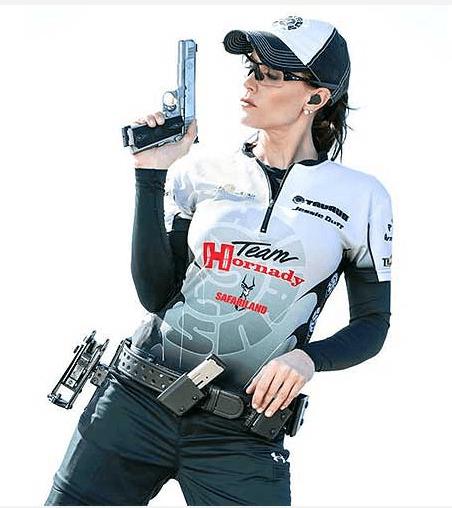

Supplement: Supplementary file 1 — Supplementary Information 1. [file 41598_2023_35190_MOESM1_ESM.zip › test/images/armas--2995-_jpg.rf.873cfa9329bca5170383d295cac8be61.jpg]

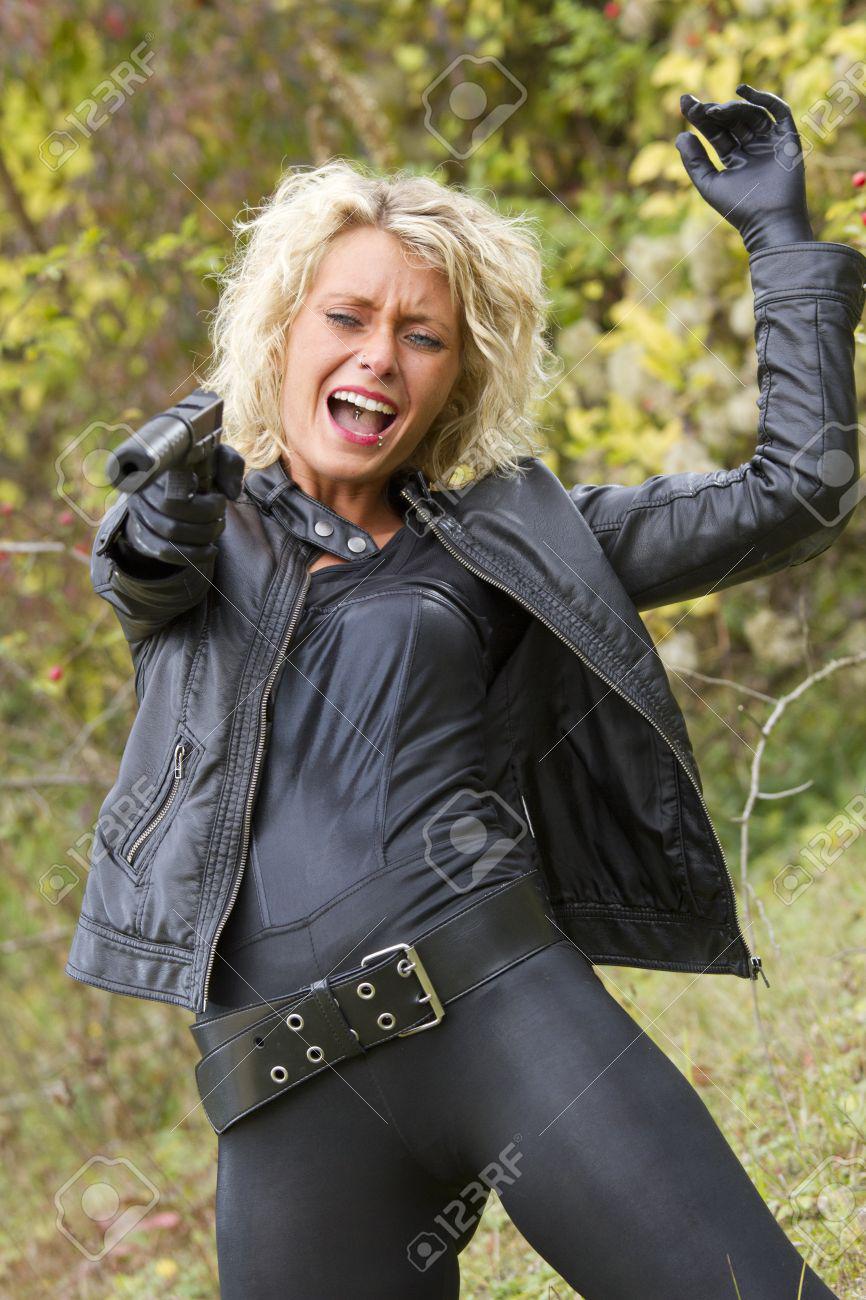

Supplement: Supplementary file 1 — Supplementary Information 1. [file 41598_2023_35190_MOESM1_ESM.zip › test/images/armas--30-_jpg.rf.47942e9b8009ac9ac07f7eab4234ab9b.jpg]

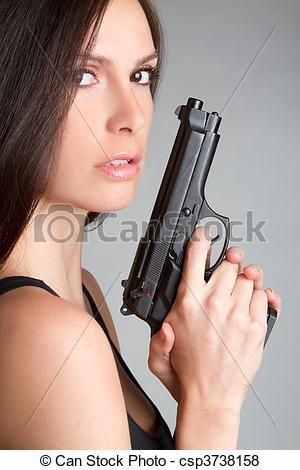

Supplement: Supplementary file 1 — Supplementary Information 1. [file 41598_2023_35190_MOESM1_ESM.zip › test/images/armas--330-_jpg.rf.db540608250920c116fb456246e230b4.jpg]

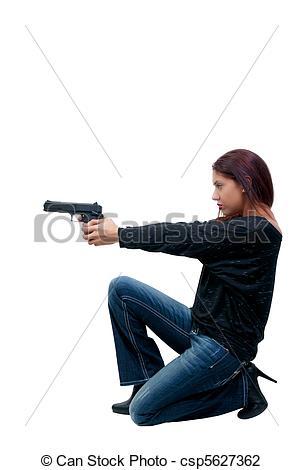

Supplement: Supplementary file 1 — Supplementary Information 1. [file 41598_2023_35190_MOESM1_ESM.zip › test/images/armas--332-_jpg.rf.41de5e5073e06f5655f5abe4229bd6b7.jpg]

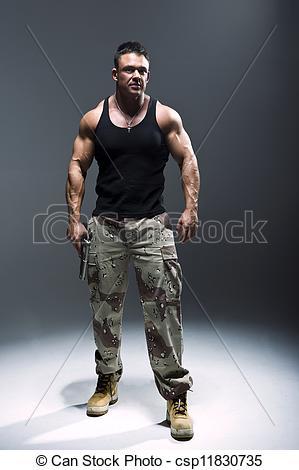

Supplement: Supplementary file 1 — Supplementary Information 1. [file 41598_2023_35190_MOESM1_ESM.zip › test/images/armas--338-_jpg.rf.ba9236bb4f886719ea88cf6a2e05d2b4.jpg]

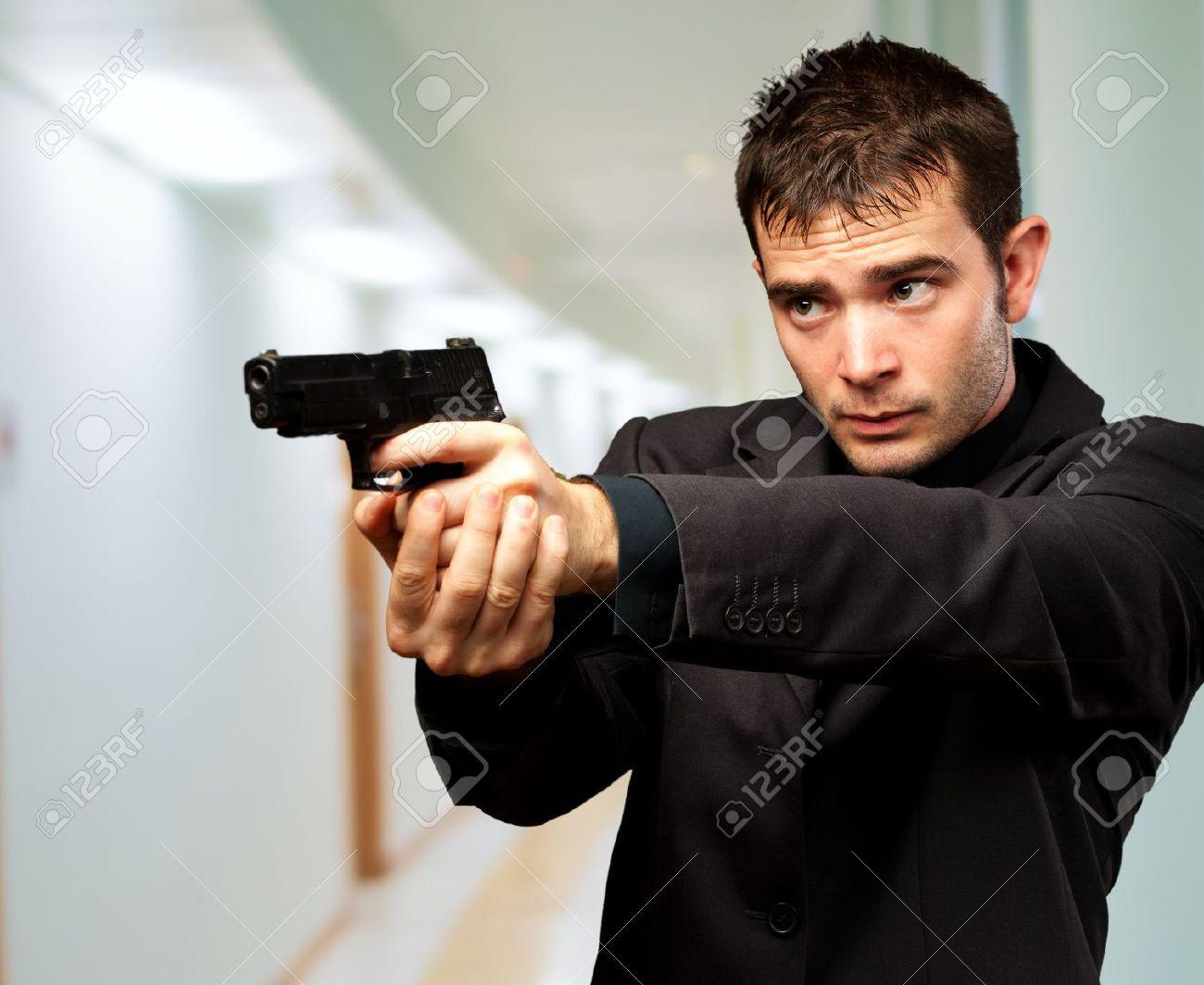

Supplement: Supplementary file 1 — Supplementary Information 1. [file 41598_2023_35190_MOESM1_ESM.zip › test/images/armas--34-_jpg.rf.7bd8e47dbf37b91b31fdeb8061269948.jpg]

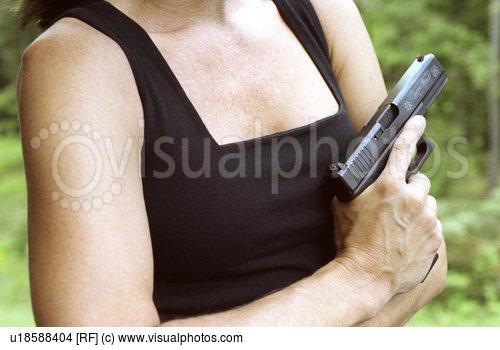

Supplement: Supplementary file 1 — Supplementary Information 1. [file 41598_2023_35190_MOESM1_ESM.zip › test/images/armas--347-_jpg.rf.1fa0aae8b0af75fbc1c42dfe6a21d20c.jpg]

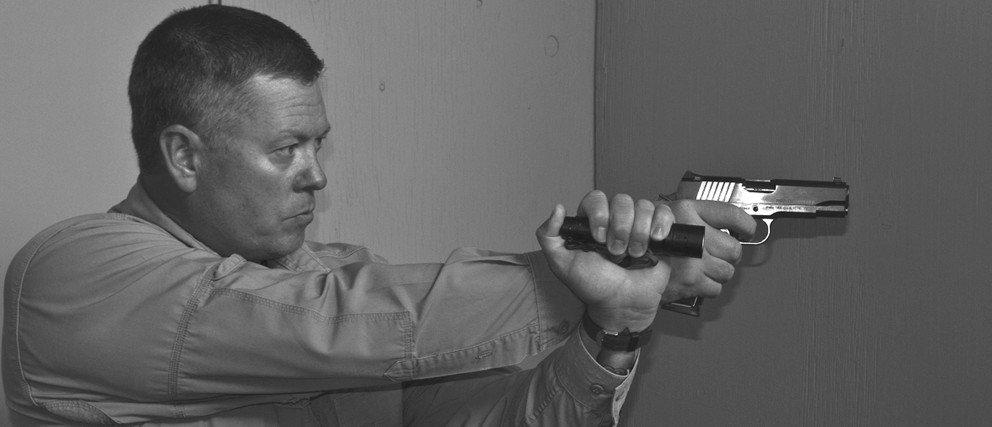

Supplement: Supplementary file 1 — Supplementary Information 1. [file 41598_2023_35190_MOESM1_ESM.zip › test/images/armas--349-_jpg.rf.485067c1ad267f2d65fcb9ea9ca4e371.jpg]

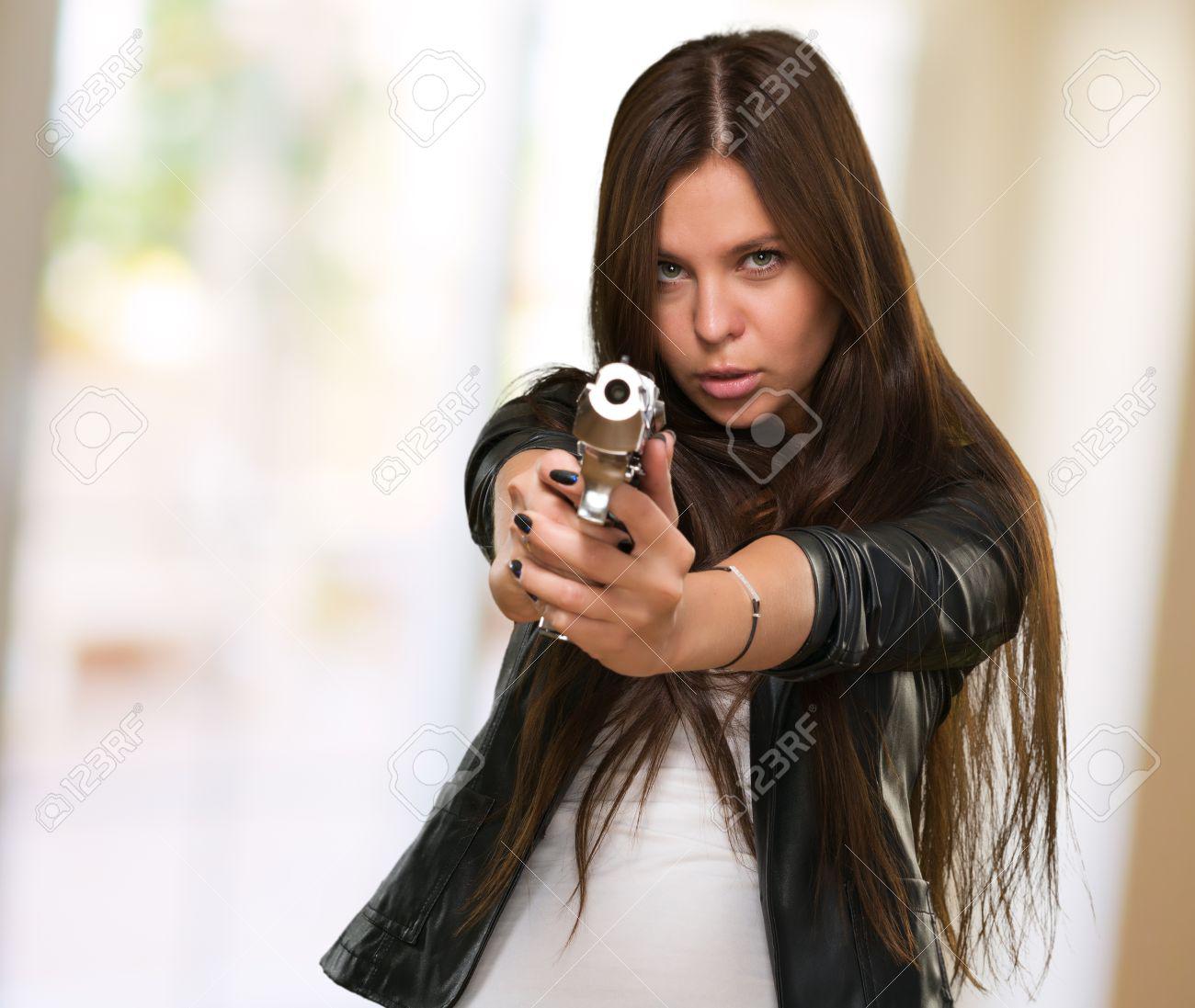

Supplement: Supplementary file 1 — Supplementary Information 1. [file 41598_2023_35190_MOESM1_ESM.zip › test/images/armas--35-_jpg.rf.eccd66de60be400d09f269c58cc1b897.jpg]

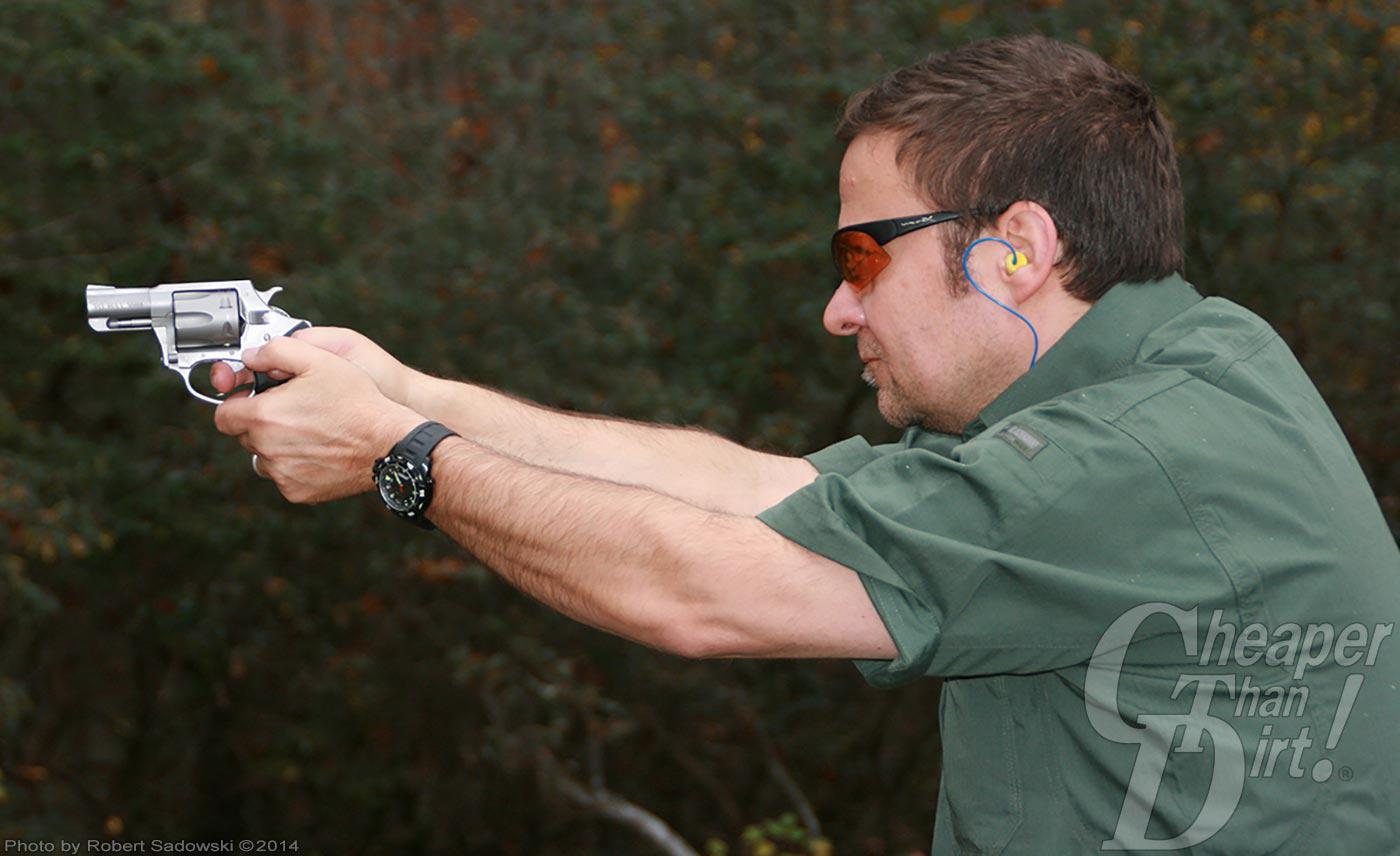

Supplement: Supplementary file 1 — Supplementary Information 1. [file 41598_2023_35190_MOESM1_ESM.zip › test/images/armas--351-_jpg.rf.aecdd591ac814a499cd108aed46b88e1.jpg]

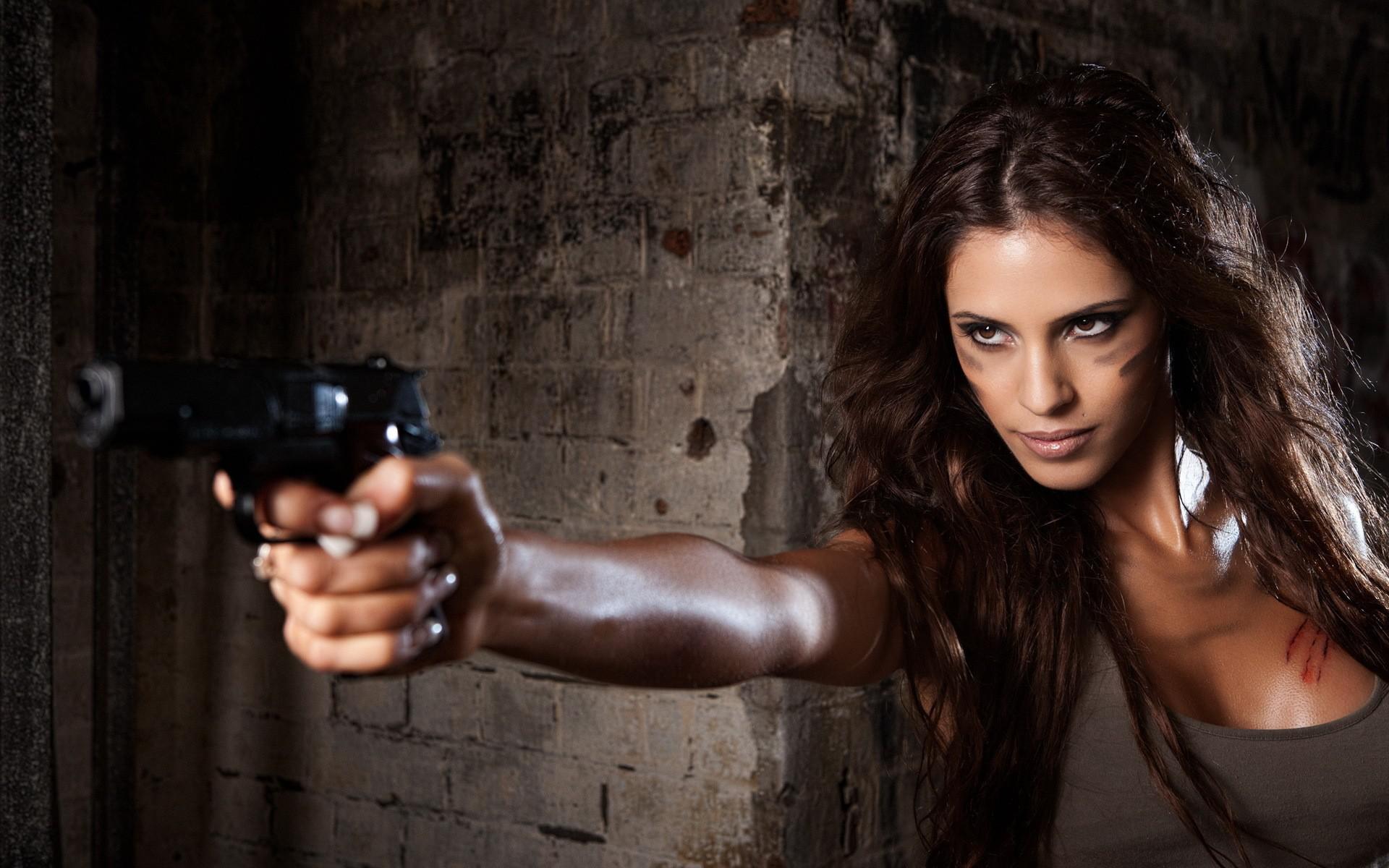

Supplement: Supplementary file 1 — Supplementary Information 1. [file 41598_2023_35190_MOESM1_ESM.zip › test/images/armas--352-_jpg.rf.7d915967cec145a39aa326aa8661d79c.jpg]

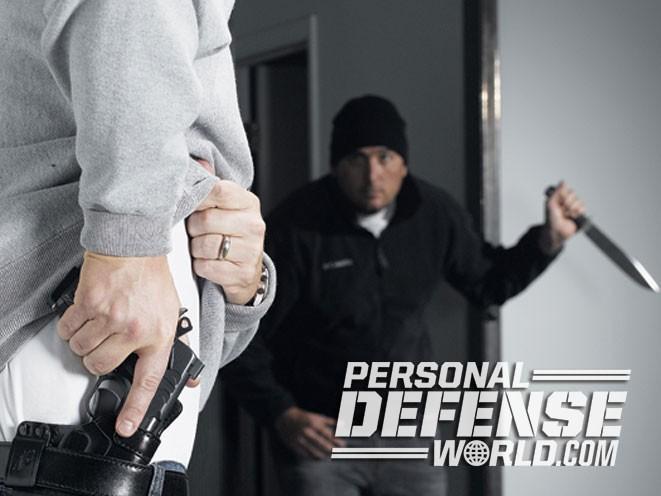

Supplement: Supplementary file 1 — Supplementary Information 1. [file 41598_2023_35190_MOESM1_ESM.zip › test/images/armas--360-_jpg.rf.5116f37c1cae7f28814ad7592ee1827f.jpg]

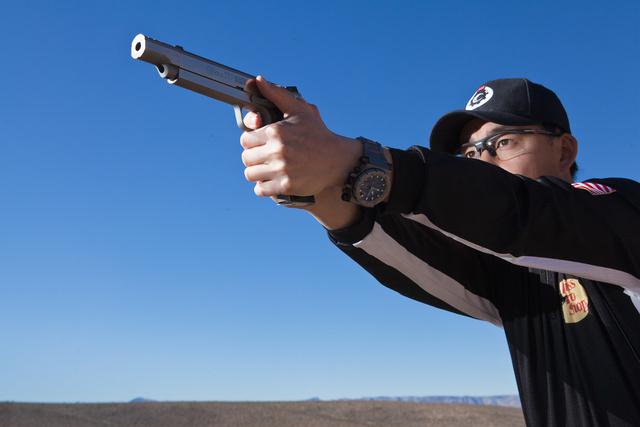

Supplement: Supplementary file 1 — Supplementary Information 1. [file 41598_2023_35190_MOESM1_ESM.zip › test/images/armas--365-_jpg.rf.67c0511196f37e0f754c20df422897f6.jpg]

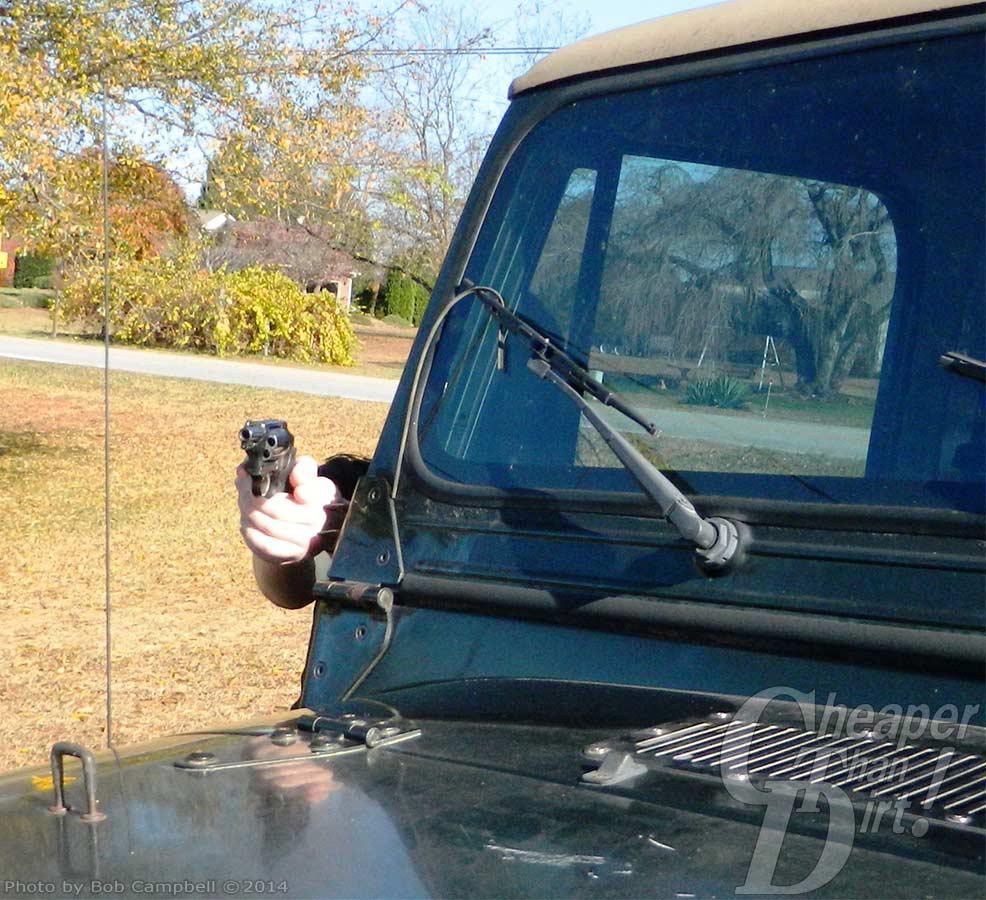

Supplement: Supplementary file 1 — Supplementary Information 1. [file 41598_2023_35190_MOESM1_ESM.zip › test/images/armas--366-_jpg.rf.80ccddb03c45fe43f8cf39f8d0db58f6.jpg]

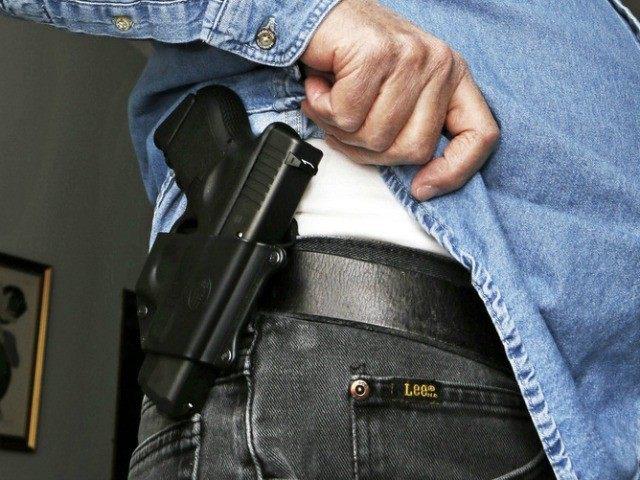

Supplement: Supplementary file 1 — Supplementary Information 1. [file 41598_2023_35190_MOESM1_ESM.zip › test/images/armas--367-_jpg.rf.5df66b5f46cb2115db1c4d3f5827b260.jpg]

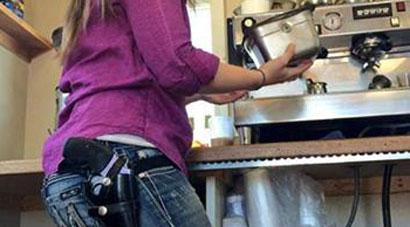

Supplement: Supplementary file 1 — Supplementary Information 1. [file 41598_2023_35190_MOESM1_ESM.zip › test/images/armas--372-_jpg.rf.c8e64b0fd8f4e689959fef5c9e3637a1.jpg]

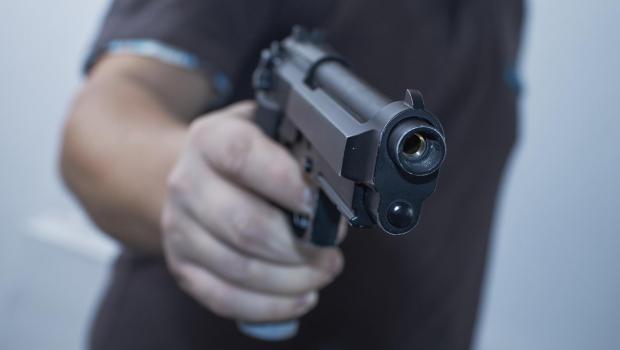

Supplement: Supplementary file 1 — Supplementary Information 1. [file 41598_2023_35190_MOESM1_ESM.zip › test/images/armas--373-_jpg.rf.974845eed0d9d1c9a07fe2cba63d98db.jpg]

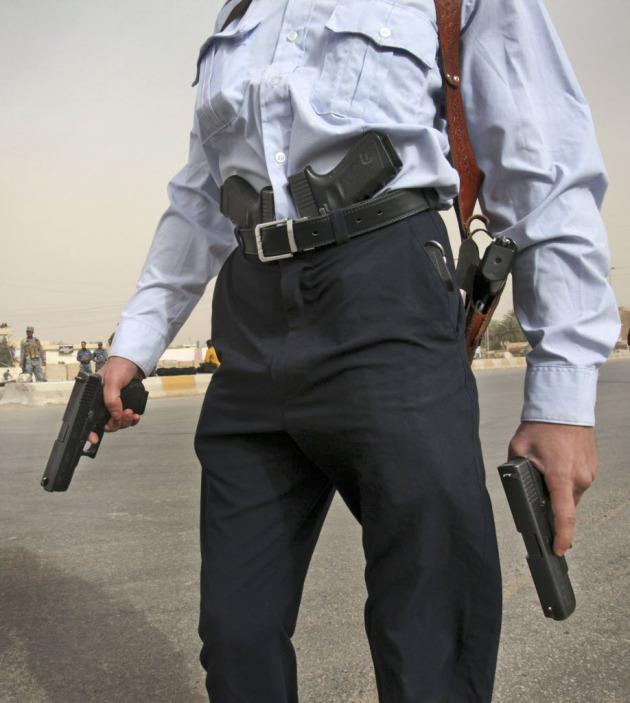

Supplement: Supplementary file 1 — Supplementary Information 1. [file 41598_2023_35190_MOESM1_ESM.zip › test/images/armas--376-_jpg.rf.8e89cf4e347ae64d6cc10f807a727afd.jpg]

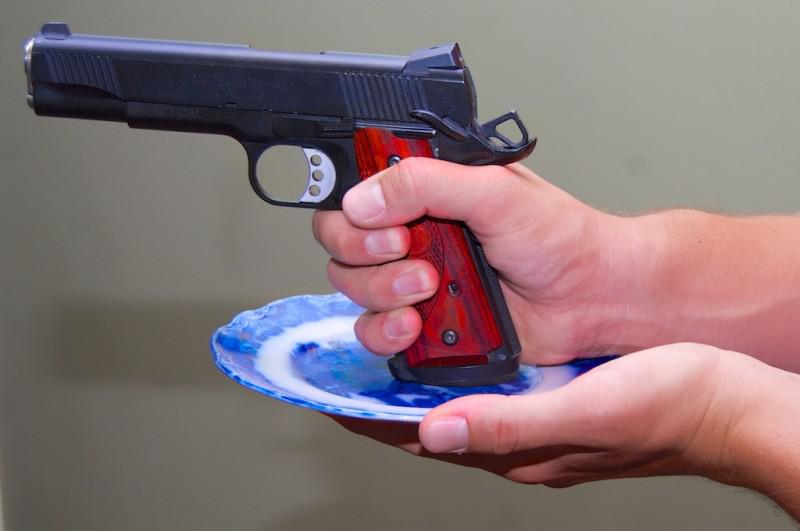

Supplement: Supplementary file 1 — Supplementary Information 1. [file 41598_2023_35190_MOESM1_ESM.zip › test/images/armas--378-_jpg.rf.52296feea77c5e02ec29ca56c89638ac.jpg]

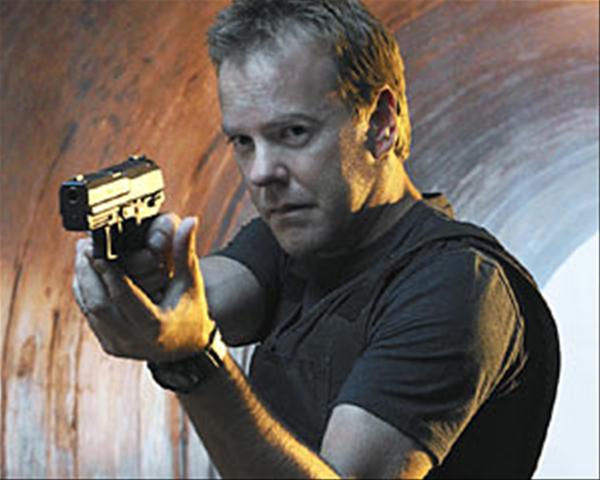

Supplement: Supplementary file 1 — Supplementary Information 1. [file 41598_2023_35190_MOESM1_ESM.zip › test/images/armas--379-_jpg.rf.6e0bd03a1a63997b031710111834f0b3.jpg]

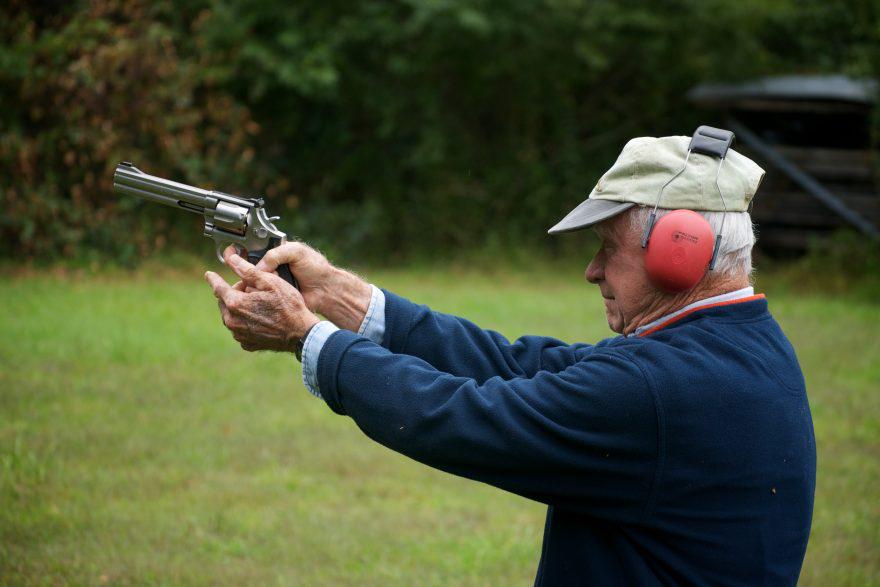

Supplement: Supplementary file 1 — Supplementary Information 1. [file 41598_2023_35190_MOESM1_ESM.zip › test/images/armas--391-_jpg.rf.193f5645b21b9cc56c2a9fb02329316d.jpg]

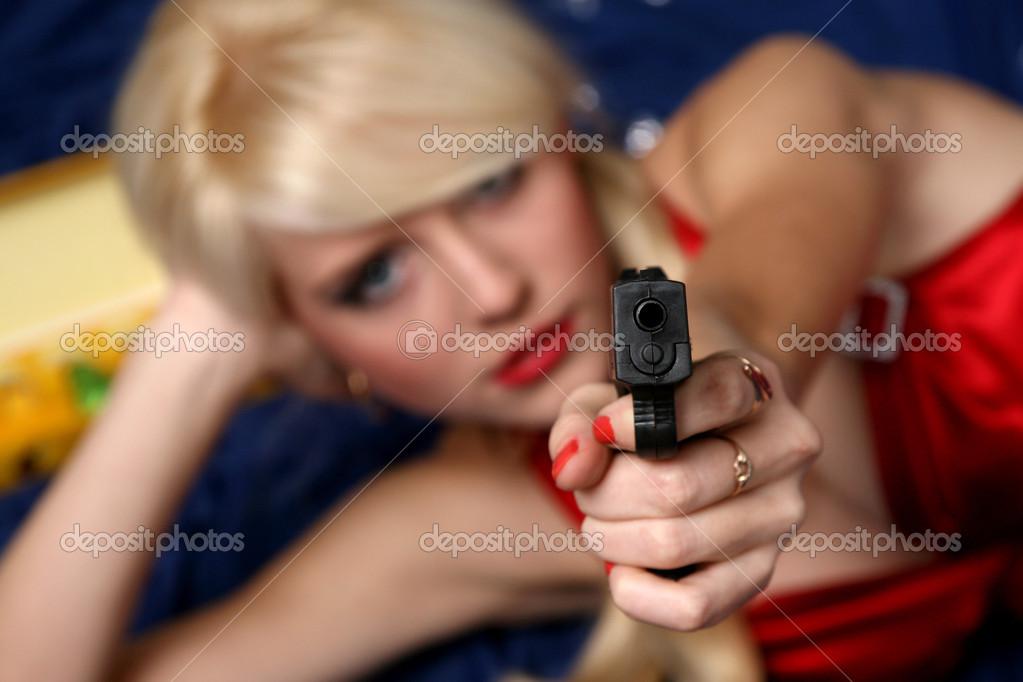

Supplement: Supplementary file 1 — Supplementary Information 1. [file 41598_2023_35190_MOESM1_ESM.zip › test/images/armas--394-_jpg.rf.08e9db0f69f3c470a9b40b96a99961f7.jpg]

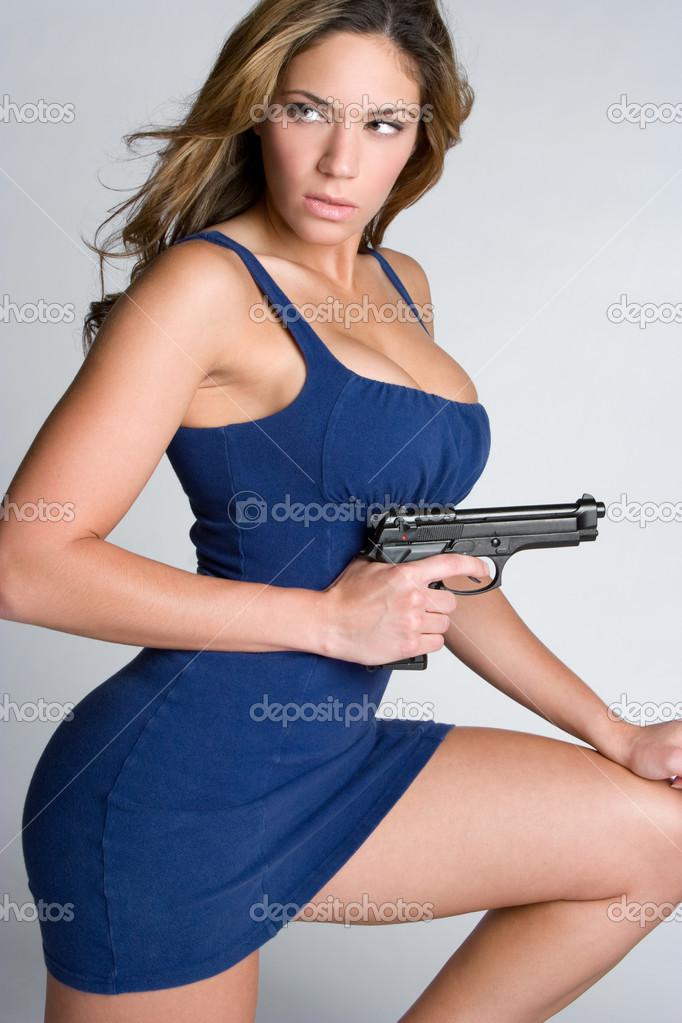

Supplement: Supplementary file 1 — Supplementary Information 1. [file 41598_2023_35190_MOESM1_ESM.zip › test/images/armas--397-_jpg.rf.c7afe3f3927d27a42dfce7dcd306849d.jpg]

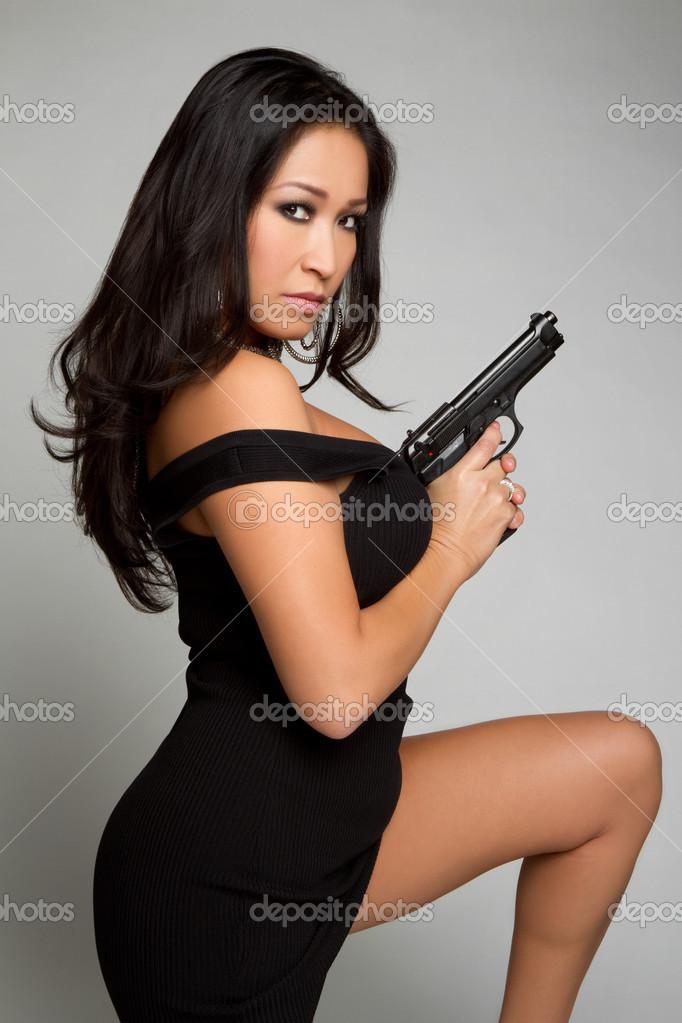

Supplement: Supplementary file 1 — Supplementary Information 1. [file 41598_2023_35190_MOESM1_ESM.zip › test/images/armas--398-_jpg.rf.876e6a1cd1e516f3e5d6bd2c5e9ece14.jpg]

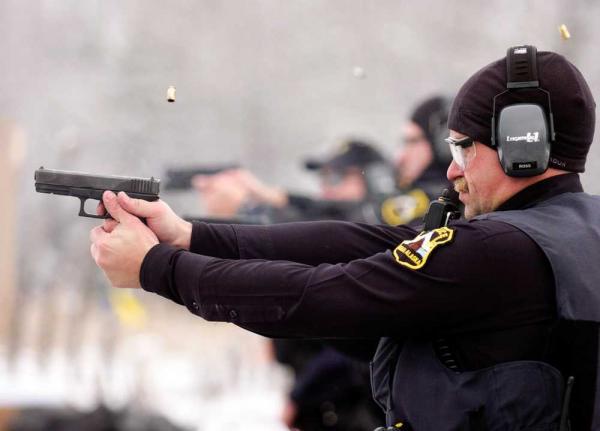

Supplement: Supplementary file 1 — Supplementary Information 1. [file 41598_2023_35190_MOESM1_ESM.zip › test/images/armas--4-_jpg.rf.e9a04f20967290b232d09d0207aac5a7.jpg]

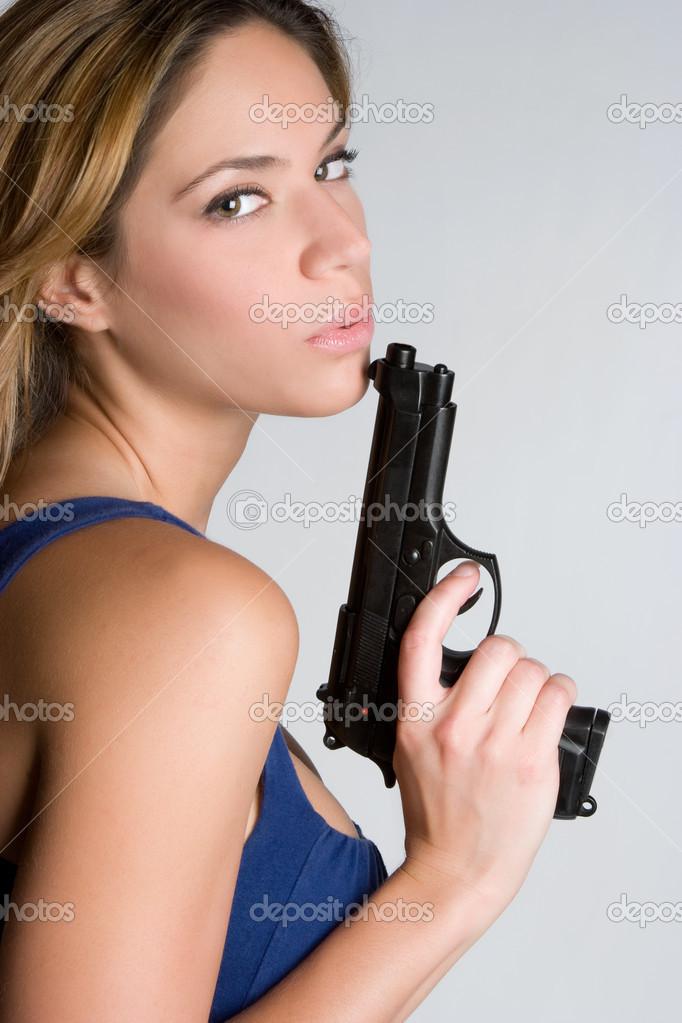

Supplement: Supplementary file 1 — Supplementary Information 1. [file 41598_2023_35190_MOESM1_ESM.zip › test/images/armas--400-_jpg.rf.623ba139518b1c0b82a67982b99f4810.jpg]

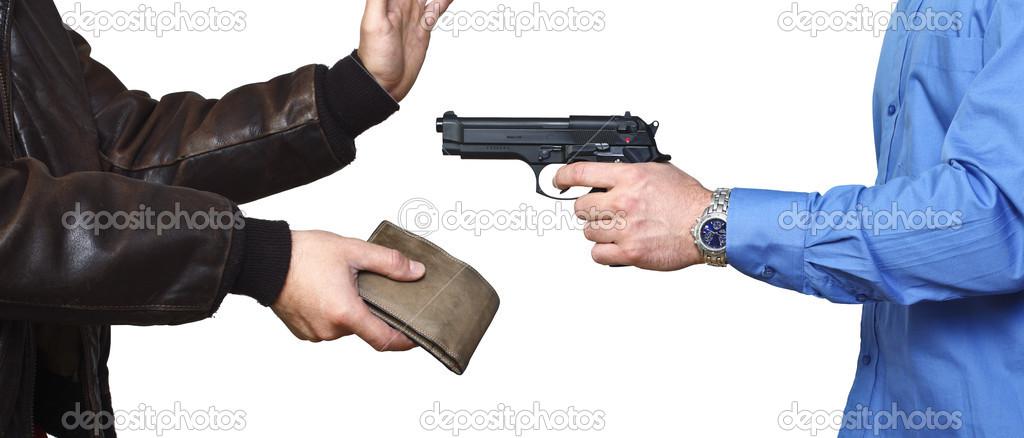

Supplement: Supplementary file 1 — Supplementary Information 1. [file 41598_2023_35190_MOESM1_ESM.zip › test/images/armas--401-_jpg.rf.dcb0e6e31801567a7d3db8cdb0f63932.jpg]

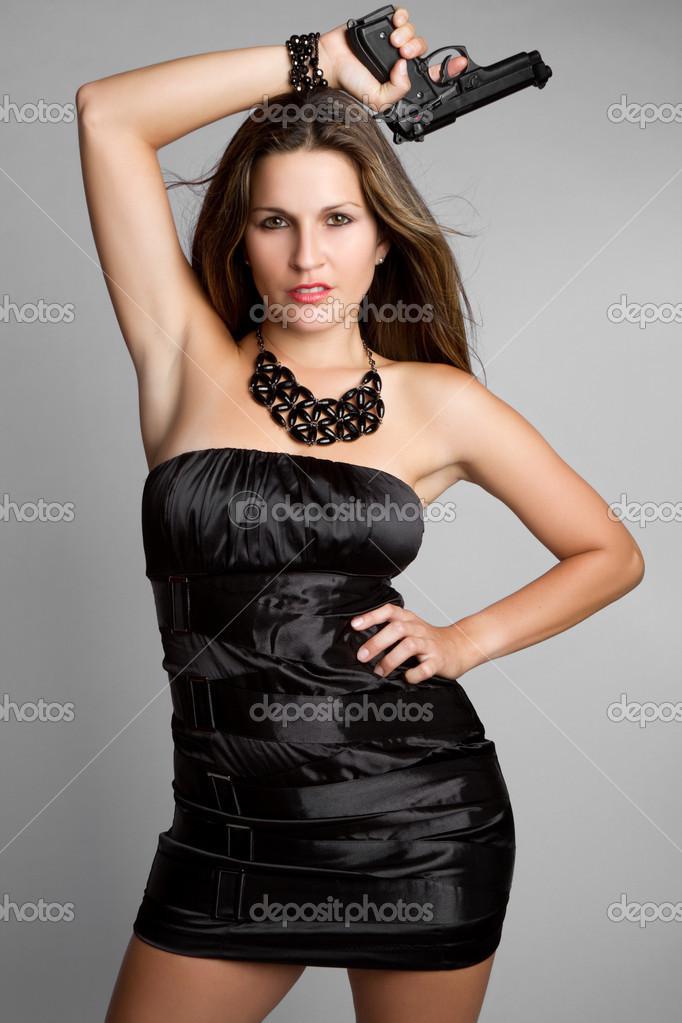

Supplement: Supplementary file 1 — Supplementary Information 1. [file 41598_2023_35190_MOESM1_ESM.zip › test/images/armas--402-_jpg.rf.9add89edc3e4d3ea3dd17f036589a3db.jpg]

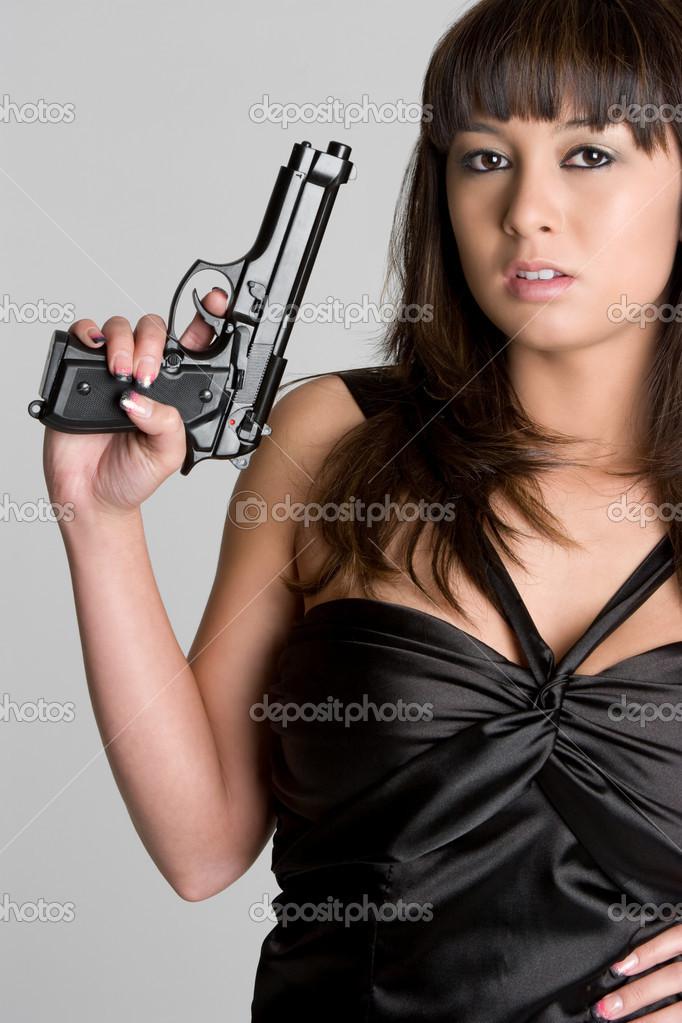

Supplement: Supplementary file 1 — Supplementary Information 1. [file 41598_2023_35190_MOESM1_ESM.zip › test/images/armas--403-_jpg.rf.bccd2304e5a9412c2d477303eb68dc36.jpg]

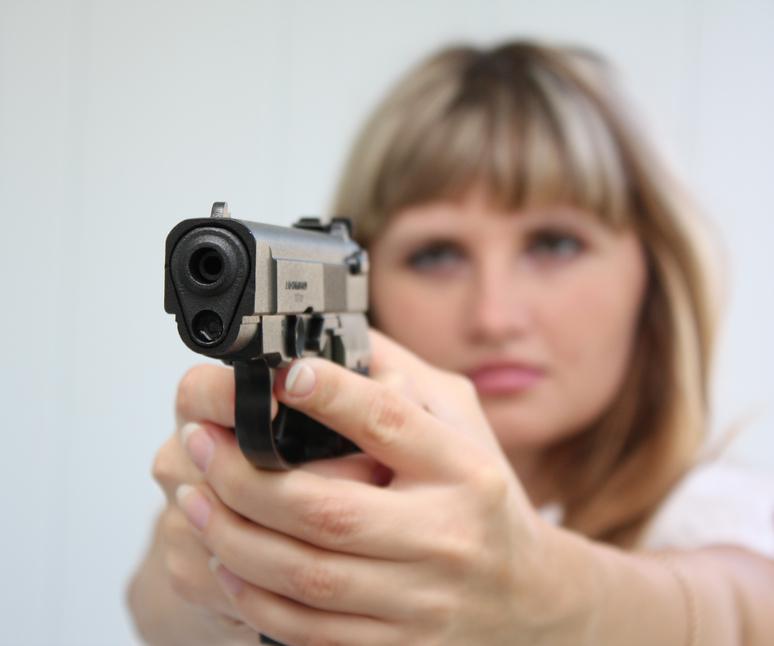

Supplement: Supplementary file 1 — Supplementary Information 1. [file 41598_2023_35190_MOESM1_ESM.zip › test/images/armas--406-_jpg.rf.2d5cc9f79ecf30e7f49ed972c9c9ec7e.jpg]

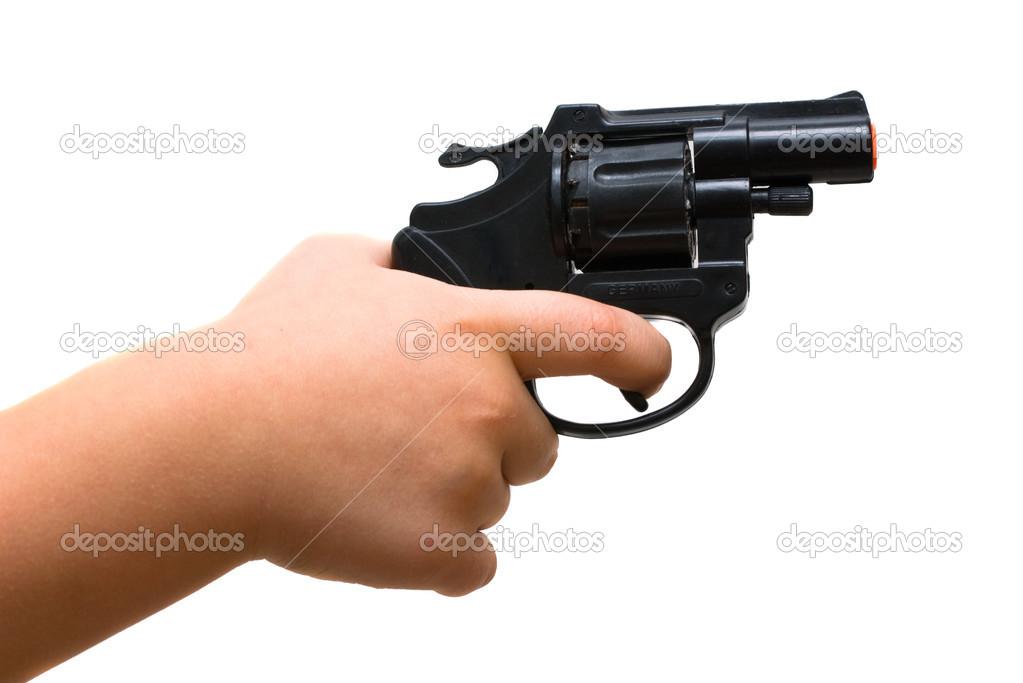

Supplement: Supplementary file 1 — Supplementary Information 1. [file 41598_2023_35190_MOESM1_ESM.zip › test/images/armas--408-_jpg.rf.97c841db0cdb4647466bed3a8c8b9f8f.jpg]

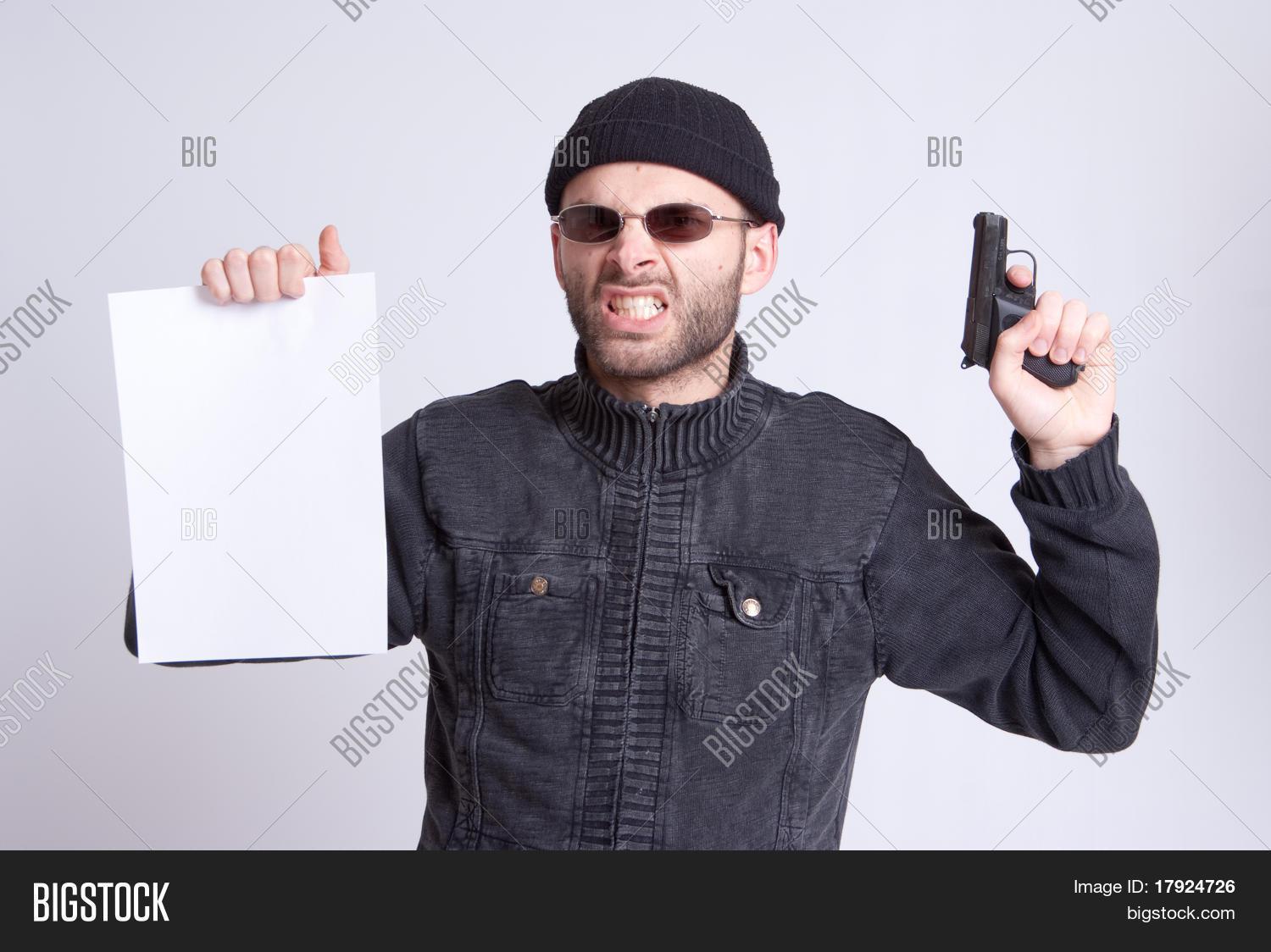

Supplement: Supplementary file 1 — Supplementary Information 1. [file 41598_2023_35190_MOESM1_ESM.zip › test/images/armas--41-_jpg.rf.b8771100b59ab4f3ddb08f0f193dde8a.jpg]

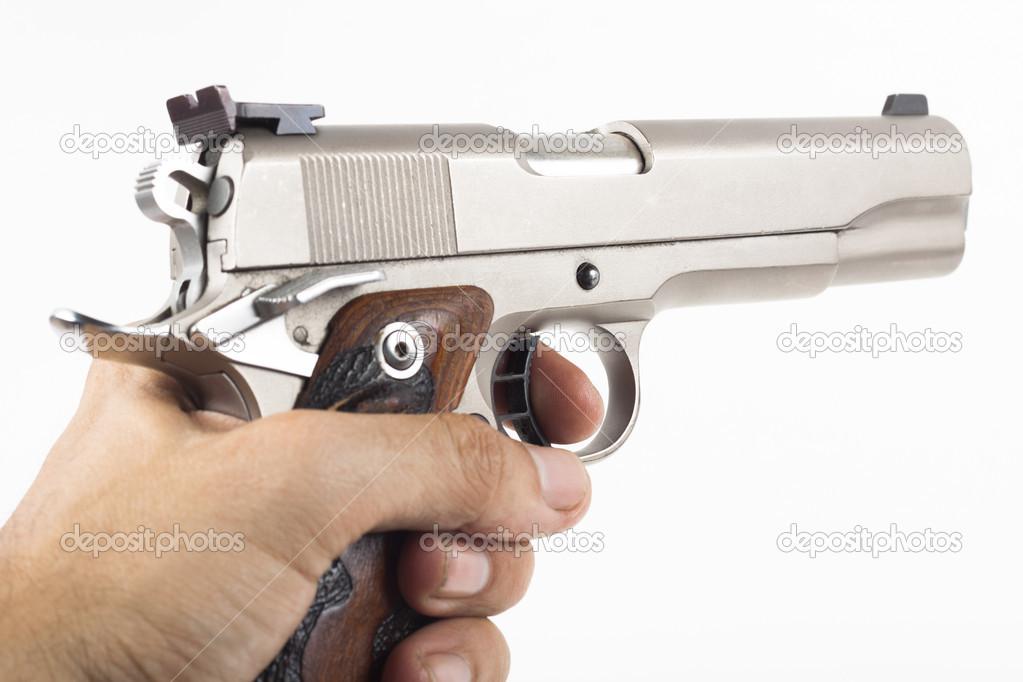

Supplement: Supplementary file 1 — Supplementary Information 1. [file 41598_2023_35190_MOESM1_ESM.zip › test/images/armas--413-_jpg.rf.47dbbf4c2a11758989708249baa53c2d.jpg]

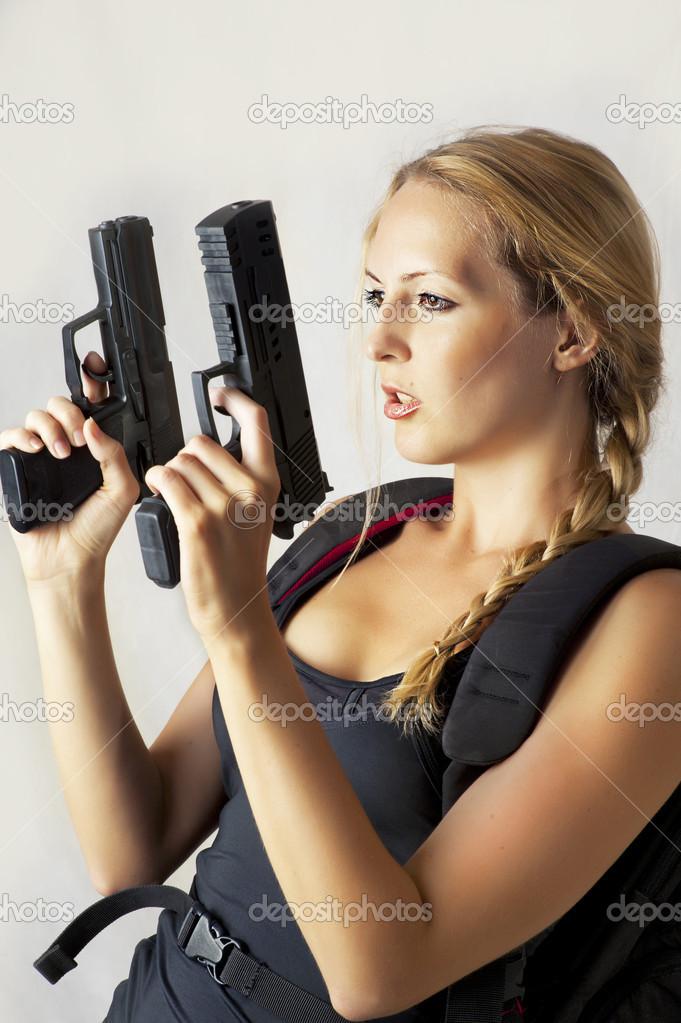

Supplement: Supplementary file 1 — Supplementary Information 1. [file 41598_2023_35190_MOESM1_ESM.zip › test/images/armas--414-_jpg.rf.bafe3606d55054d6552b3d27c9b49b56.jpg]

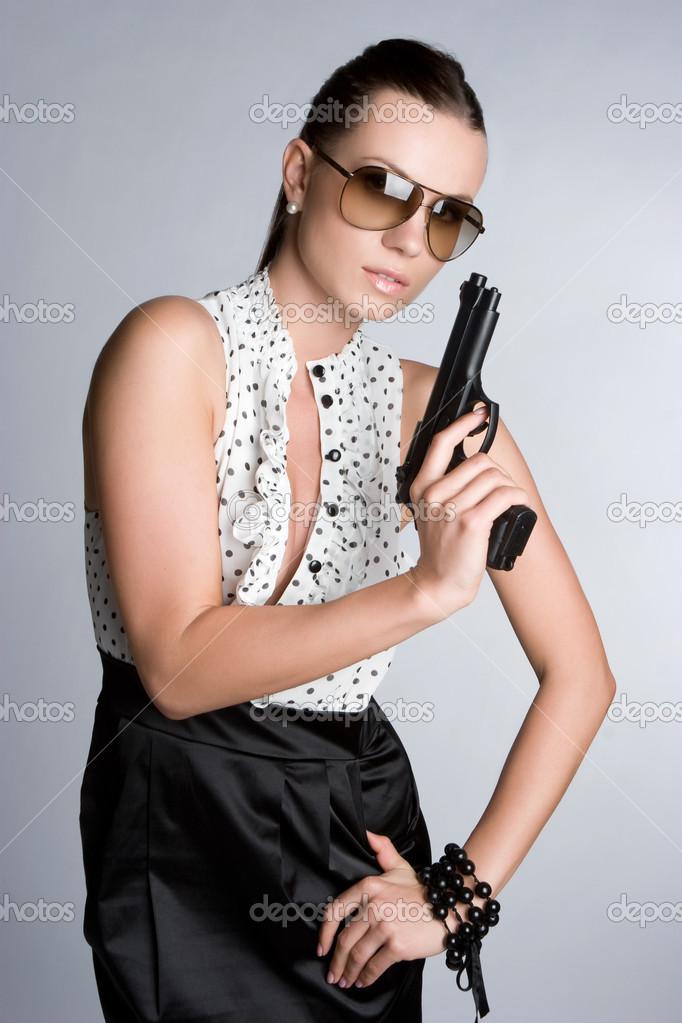

Supplement: Supplementary file 1 — Supplementary Information 1. [file 41598_2023_35190_MOESM1_ESM.zip › test/images/armas--415-_jpg.rf.695bcc1b2207e2f63c094f62c992b41f.jpg]

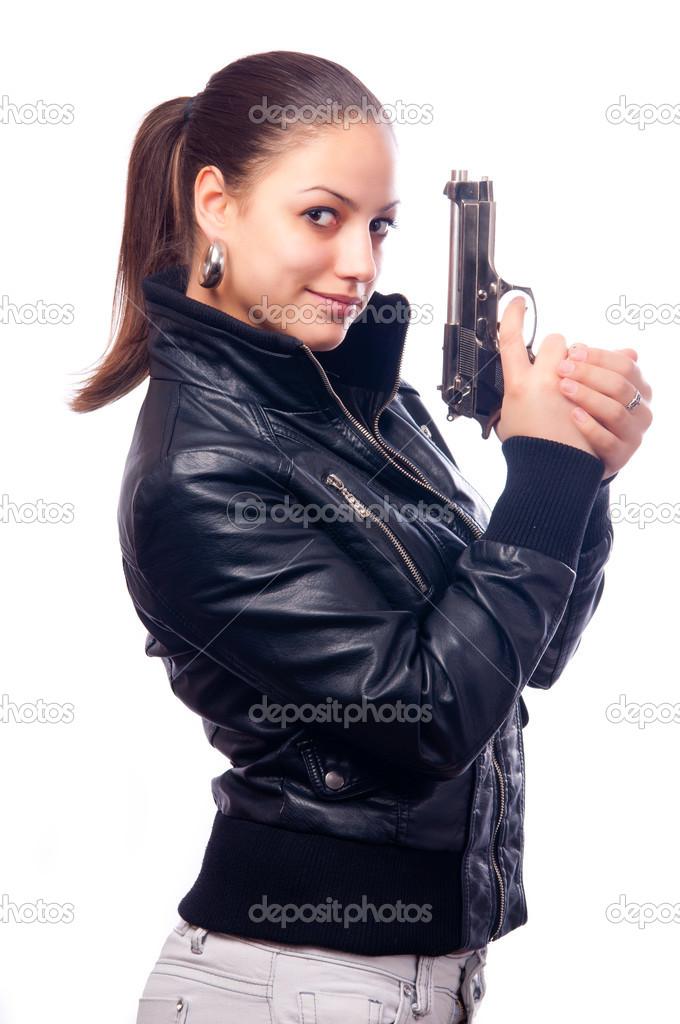

Supplement: Supplementary file 1 — Supplementary Information 1. [file 41598_2023_35190_MOESM1_ESM.zip › test/images/armas--418-_jpg.rf.79d4a8aba39f4638032959eef962e1e4.jpg]

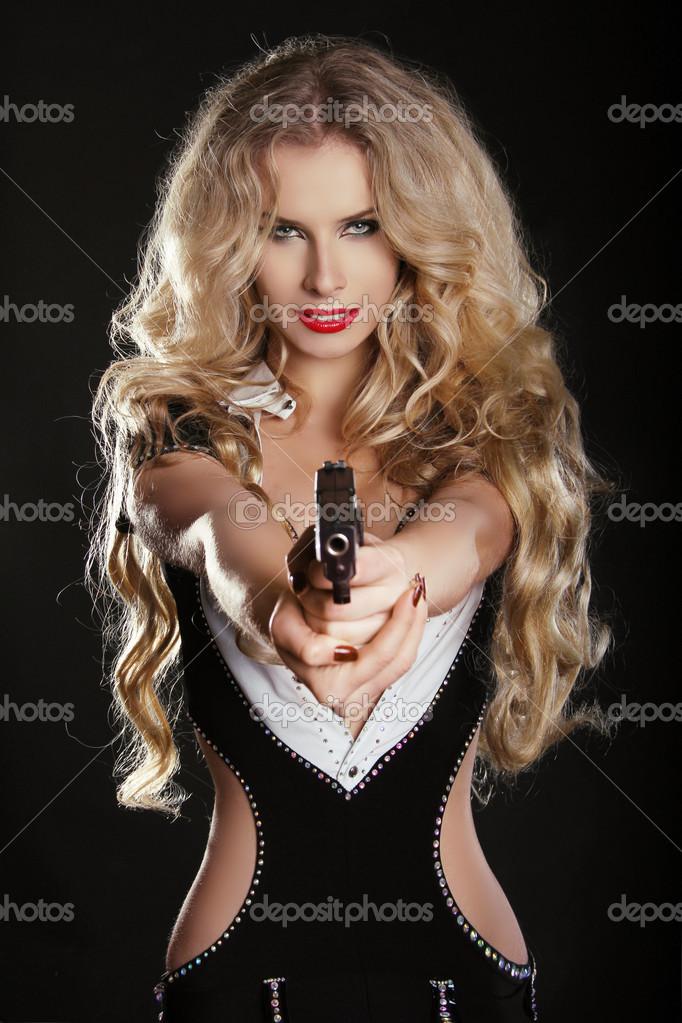

Supplement: Supplementary file 1 — Supplementary Information 1. [file 41598_2023_35190_MOESM1_ESM.zip › test/images/armas--421-_jpg.rf.38eeca2f4ee37285d0f65322d8159ab7.jpg]

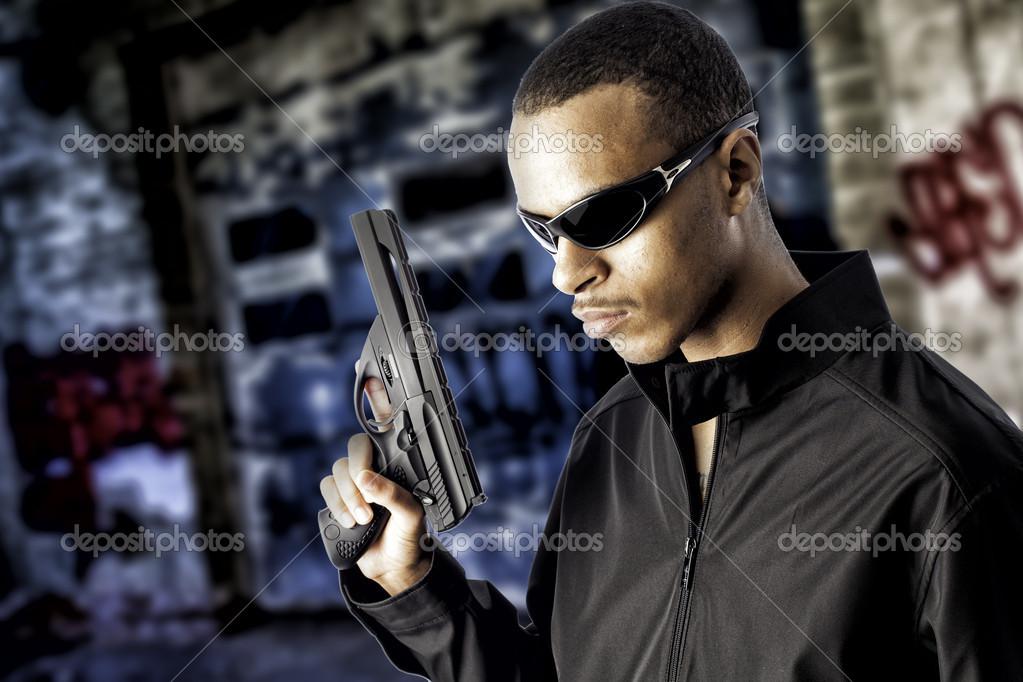

Supplement: Supplementary file 1 — Supplementary Information 1. [file 41598_2023_35190_MOESM1_ESM.zip › test/images/armas--422-_jpg.rf.78905a320a7785009f1479f6983e961e.jpg]

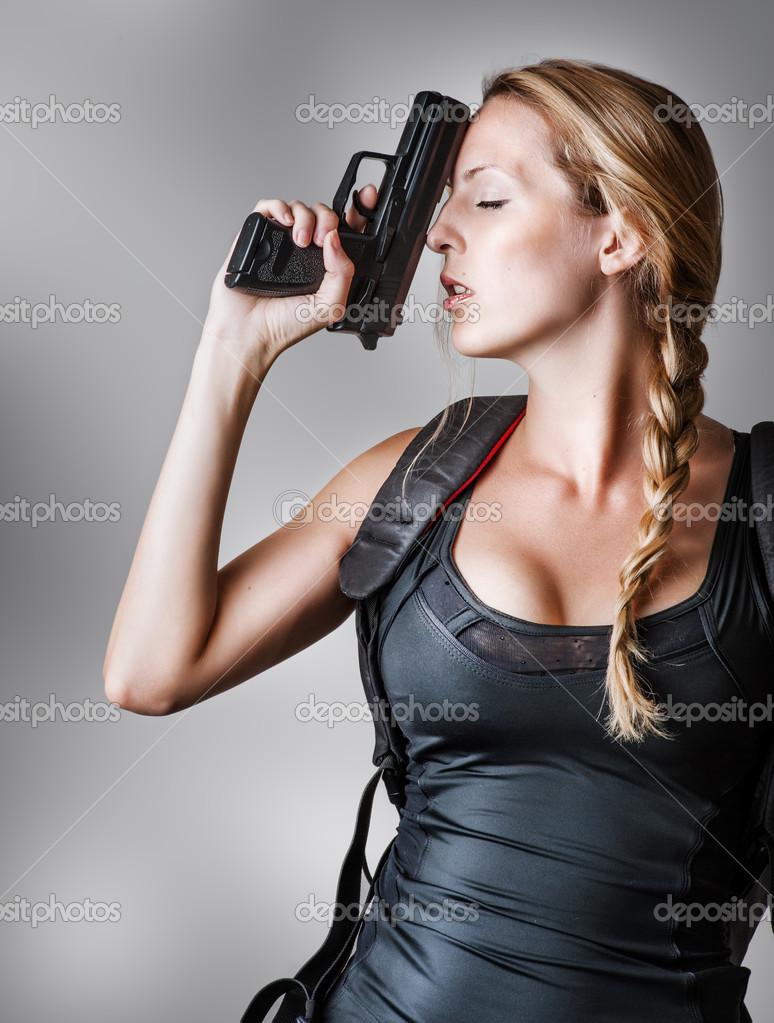

Supplement: Supplementary file 1 — Supplementary Information 1. [file 41598_2023_35190_MOESM1_ESM.zip › test/images/armas--426-_jpg.rf.0957706d84ad1ba733d38bdc621b6e19.jpg]

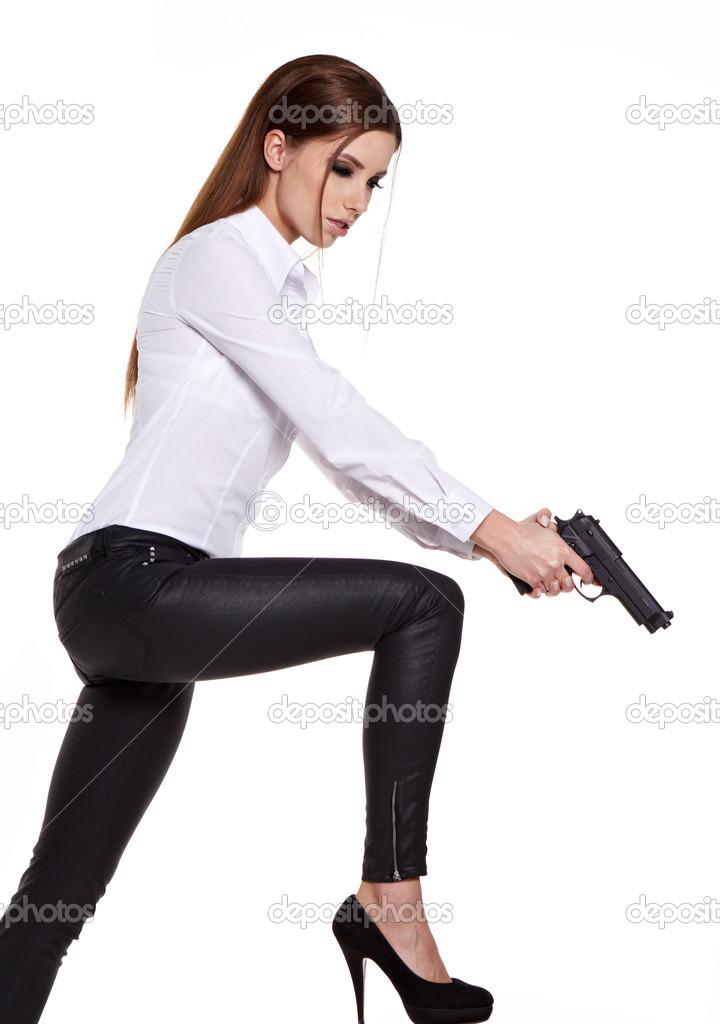

Supplement: Supplementary file 1 — Supplementary Information 1. [file 41598_2023_35190_MOESM1_ESM.zip › test/images/armas--436-_jpg.rf.a95d9446b47be45ad0d0f6c4edb247a0.jpg]

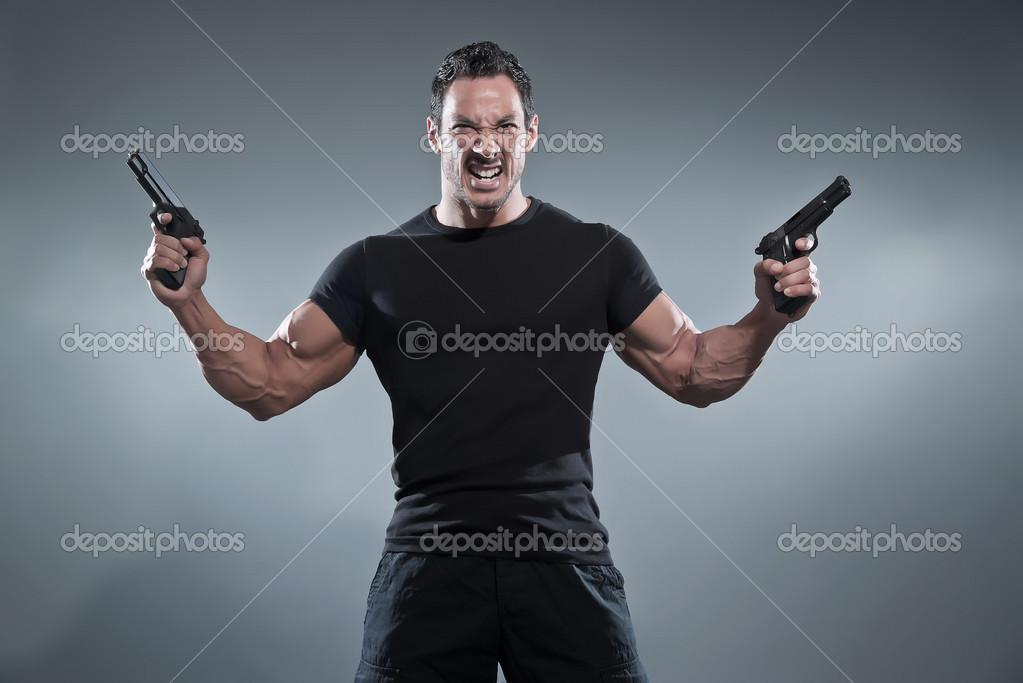

Supplement: Supplementary file 1 — Supplementary Information 1. [file 41598_2023_35190_MOESM1_ESM.zip › test/images/armas--454-_jpg.rf.ce6bc67c0377b474cba77b6db57404d6.jpg]

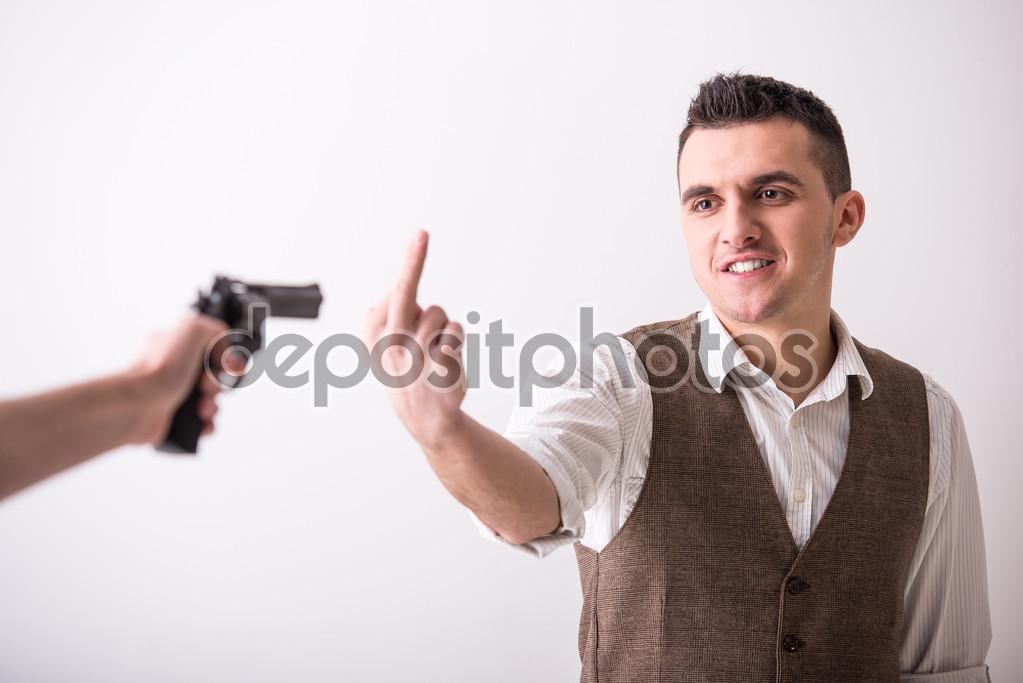

Supplement: Supplementary file 1 — Supplementary Information 1. [file 41598_2023_35190_MOESM1_ESM.zip › test/images/armas--466-_jpg.rf.3106951f00655ebe09aa58247105e617.jpg]

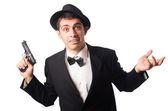

Supplement: Supplementary file 1 — Supplementary Information 1. [file 41598_2023_35190_MOESM1_ESM.zip › test/images/armas--479-_jpg.rf.f295981f0654534cd77ef04d8f9b5cdb.jpg]

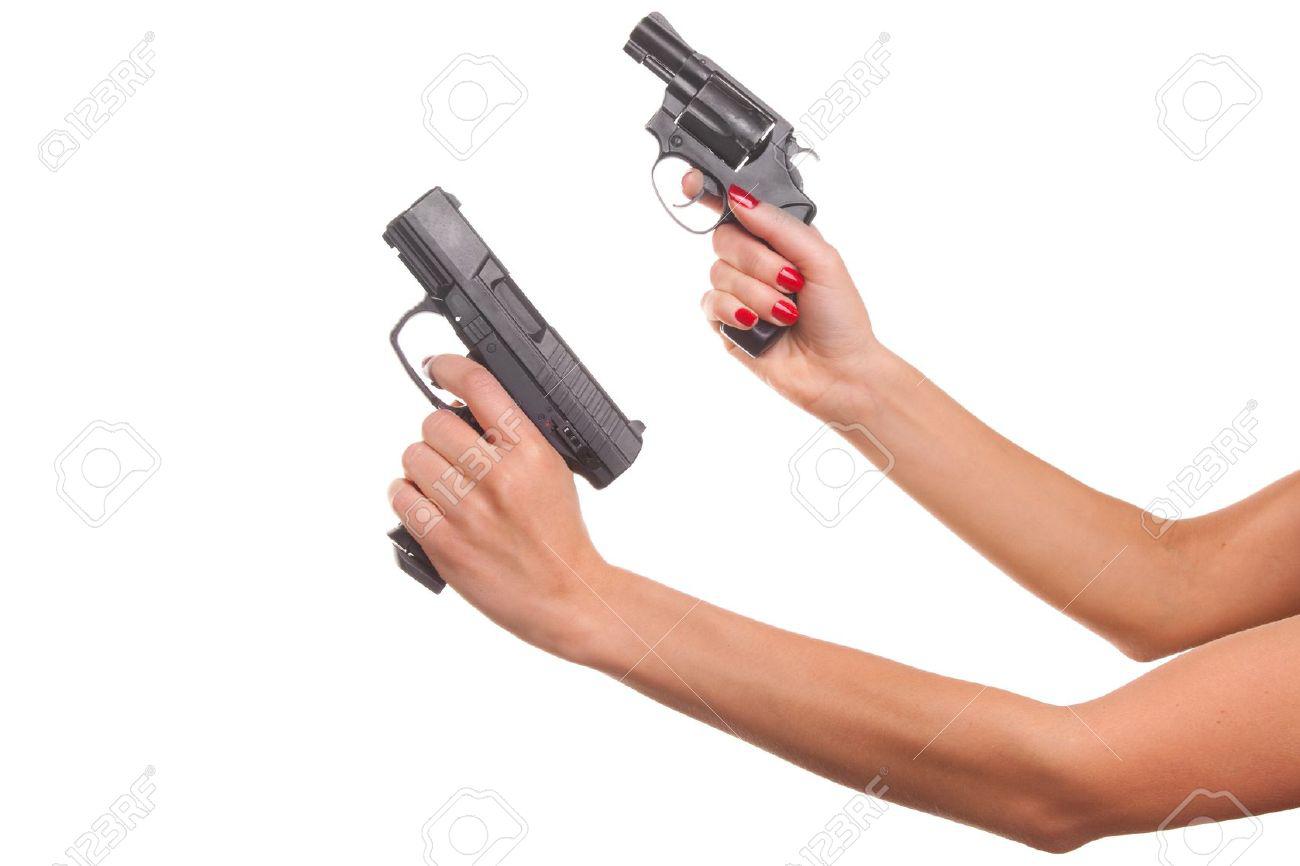

Supplement: Supplementary file 1 — Supplementary Information 1. [file 41598_2023_35190_MOESM1_ESM.zip › test/images/armas--5-_jpg.rf.f7e72ffeea73e064ce9b451507448aa3.jpg]

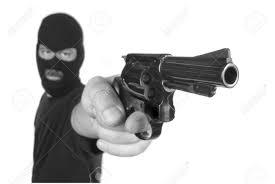

Supplement: Supplementary file 1 — Supplementary Information 1. [file 41598_2023_35190_MOESM1_ESM.zip › test/images/armas--506-_jpg.rf.7658d85fd01f9f31ead7847ca80e8add.jpg]

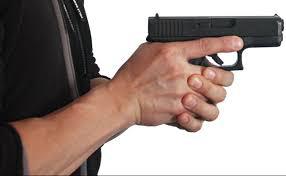

Supplement: Supplementary file 1 — Supplementary Information 1. [file 41598_2023_35190_MOESM1_ESM.zip › test/images/armas--508-_jpg.rf.75cfc082412171ae2c7e251157d02c5c.jpg]

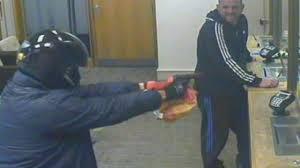

Supplement: Supplementary file 1 — Supplementary Information 1. [file 41598_2023_35190_MOESM1_ESM.zip › test/images/armas--510-_jpg.rf.d8efcb7da66103a1c6421b8d0459a620.jpg]

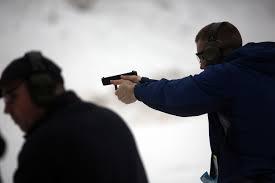

Supplement: Supplementary file 1 — Supplementary Information 1. [file 41598_2023_35190_MOESM1_ESM.zip › test/images/armas--549-_jpg.rf.d4f4e2407e2fceeccf808a5d1c0312a3.jpg]

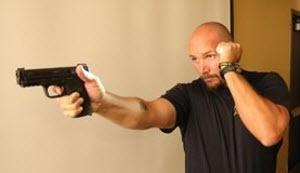

Supplement: Supplementary file 1 — Supplementary Information 1. [file 41598_2023_35190_MOESM1_ESM.zip › test/images/armas--582-_jpg.rf.a7c8737aef07b21ec5aed2749d362239.jpg]

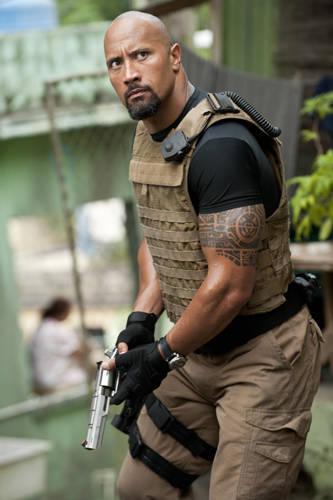

Supplement: Supplementary file 1 — Supplementary Information 1. [file 41598_2023_35190_MOESM1_ESM.zip › test/images/armas--589-_jpg.rf.fc572a40bfa1a794aa7e9b443a0678e1.jpg]

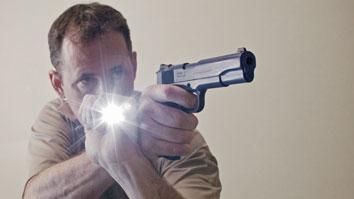

Supplement: Supplementary file 1 — Supplementary Information 1. [file 41598_2023_35190_MOESM1_ESM.zip › test/images/armas--600-_jpg.rf.8ab375d93798c77148c31843a9b9e3e2.jpg]

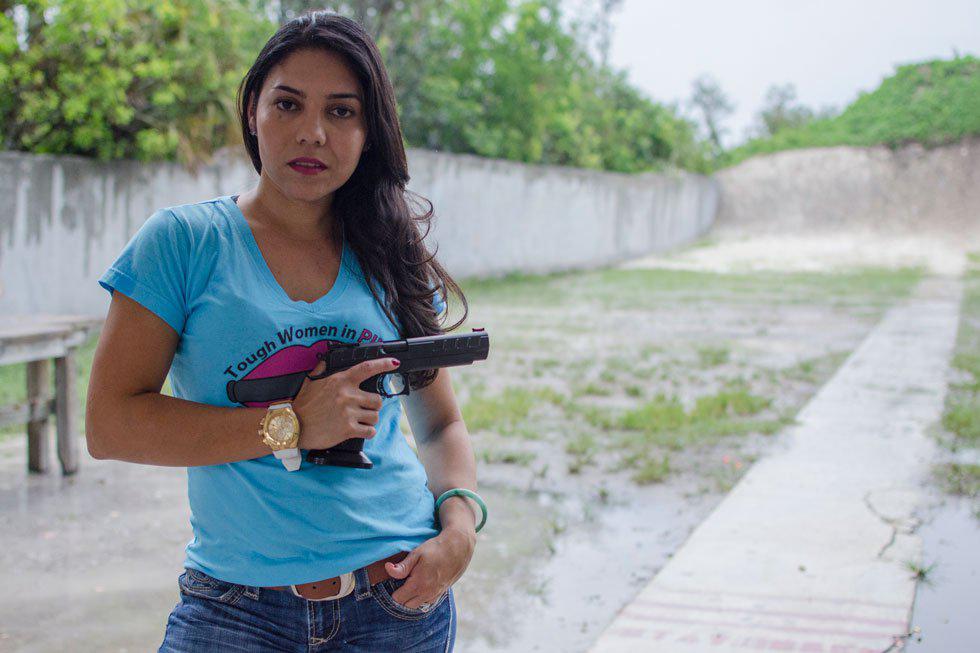

Supplement: Supplementary file 1 — Supplementary Information 1. [file 41598_2023_35190_MOESM1_ESM.zip › test/images/armas--609-_jpg.rf.dc1f3947b33ef0bf270a61053d3fc077.jpg]

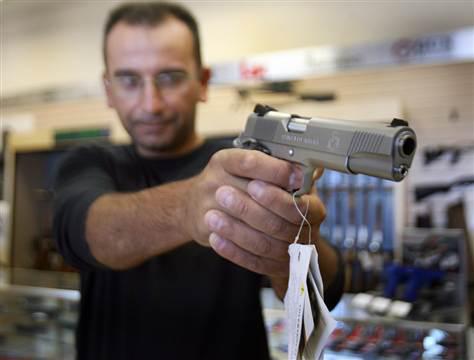

Supplement: Supplementary file 1 — Supplementary Information 1. [file 41598_2023_35190_MOESM1_ESM.zip › test/images/armas--630-_jpg.rf.40428109ff447f945a77ee8c5b27fe64.jpg]

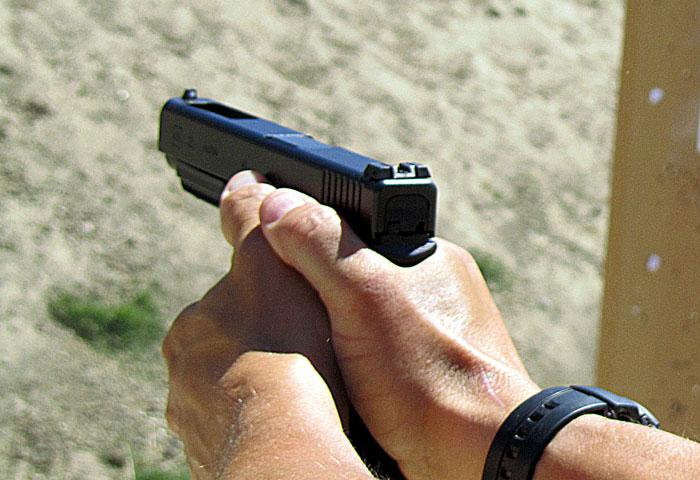

Supplement: Supplementary file 1 — Supplementary Information 1. [file 41598_2023_35190_MOESM1_ESM.zip › test/images/armas--656-_jpg.rf.e31dc32b4e20670cdbd454e33f09a49c.jpg]

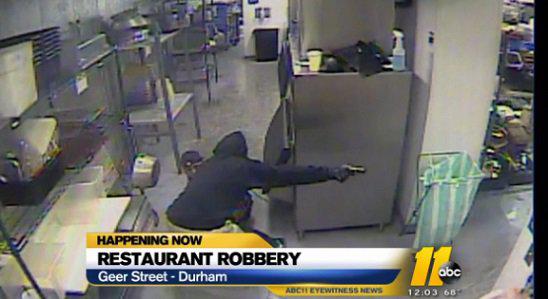

Supplement: Supplementary file 1 — Supplementary Information 1. [file 41598_2023_35190_MOESM1_ESM.zip › test/images/armas--658-_jpg.rf.7c688294d171f44a0f3e81445555b741.jpg]

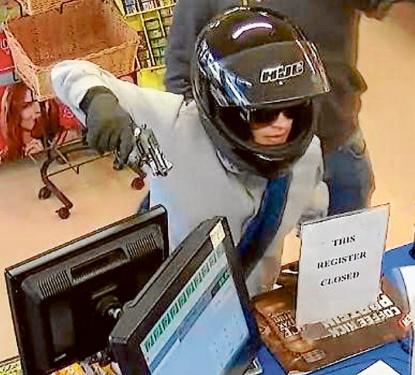

Supplement: Supplementary file 1 — Supplementary Information 1. [file 41598_2023_35190_MOESM1_ESM.zip › test/images/armas--663-_jpg.rf.4c4c491e337ae04901983ee7aa029dda.jpg]

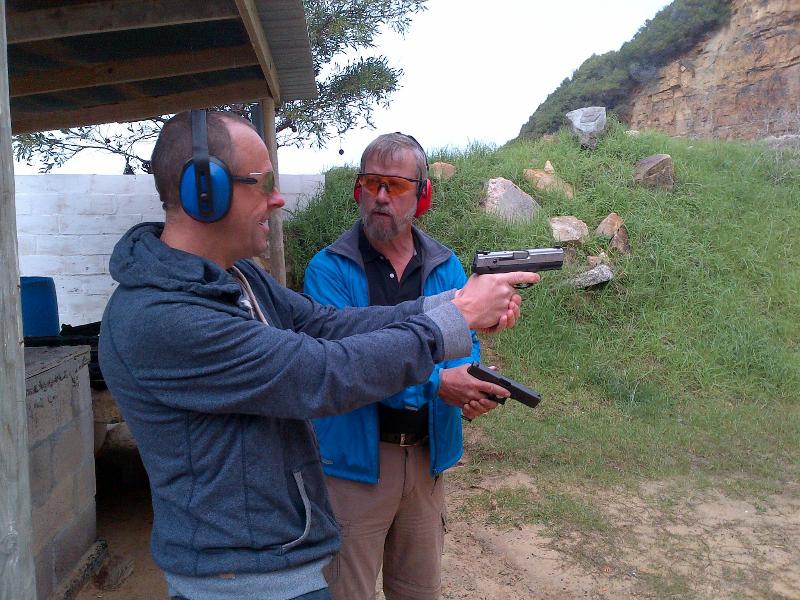

Supplement: Supplementary file 1 — Supplementary Information 1. [file 41598_2023_35190_MOESM1_ESM.zip › test/images/armas--689-_jpg.rf.0e9d45a459765980897eca77279cfcc2.jpg]

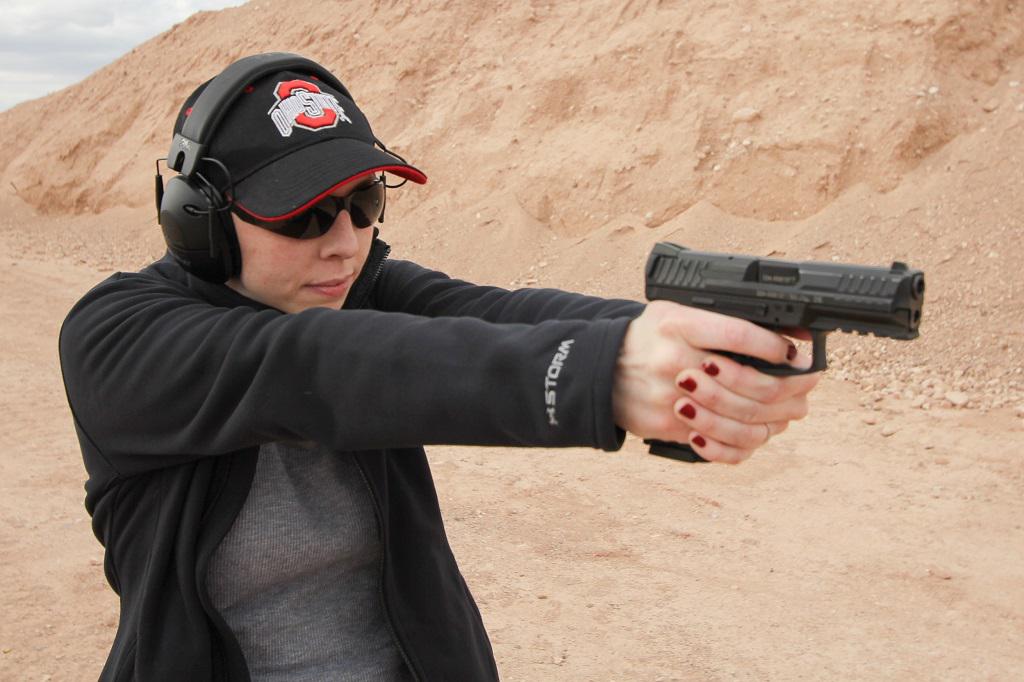

Supplement: Supplementary file 1 — Supplementary Information 1. [file 41598_2023_35190_MOESM1_ESM.zip › test/images/armas--696-_jpg.rf.bcafadab9d713696d06daf13e387cdef.jpg]

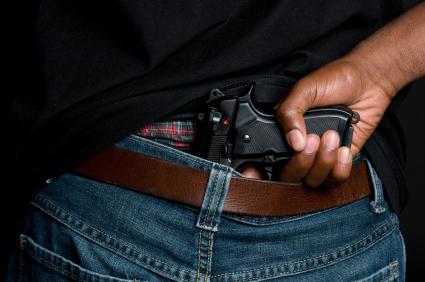

Supplement: Supplementary file 1 — Supplementary Information 1. [file 41598_2023_35190_MOESM1_ESM.zip › test/images/armas--697-_jpg.rf.7d82dc1f6277f25bf055b0741113ce33.jpg]

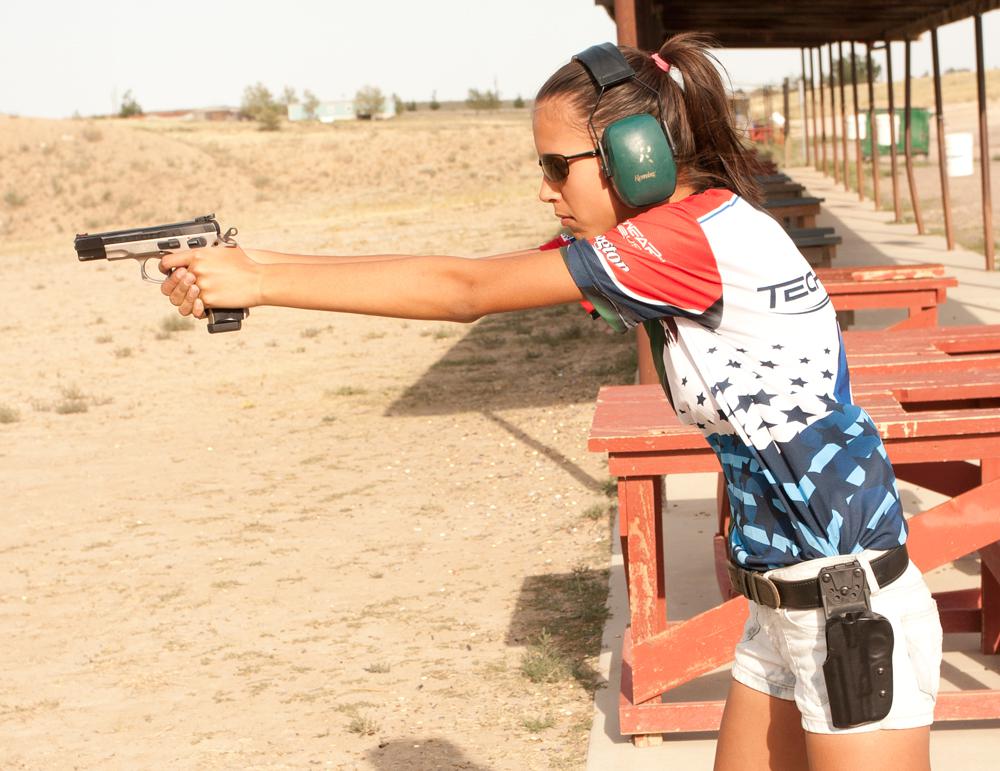

Supplement: Supplementary file 1 — Supplementary Information 1. [file 41598_2023_35190_MOESM1_ESM.zip › test/images/armas--699-_jpg.rf.b99d85d4da31ad4a1142213f6bd77db8.jpg]

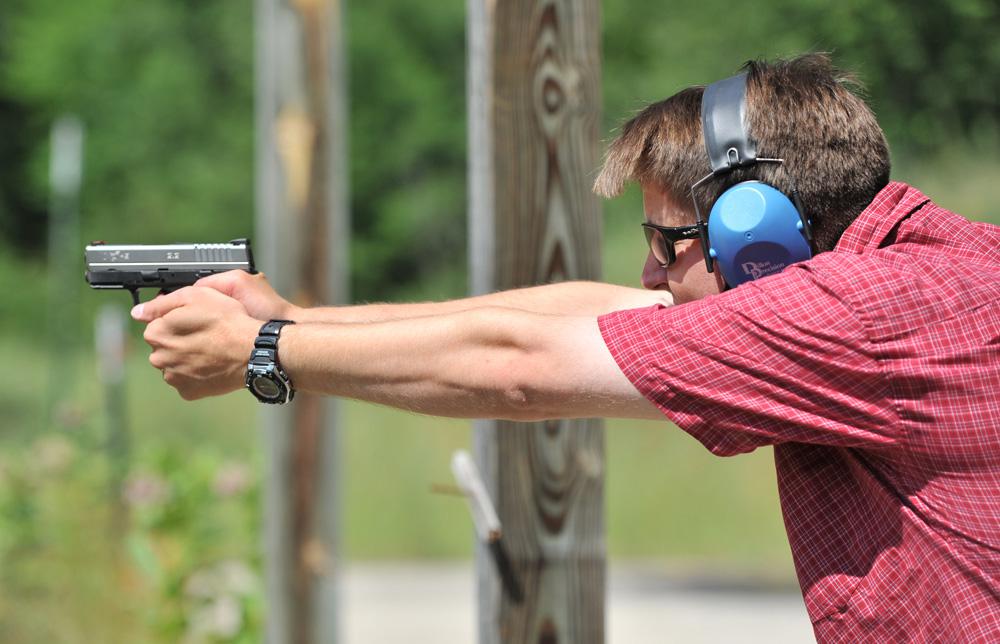

Supplement: Supplementary file 1 — Supplementary Information 1. [file 41598_2023_35190_MOESM1_ESM.zip › test/images/armas--707-_jpg.rf.d32e680bfaaf90c4ea619f2179d95f1e.jpg]

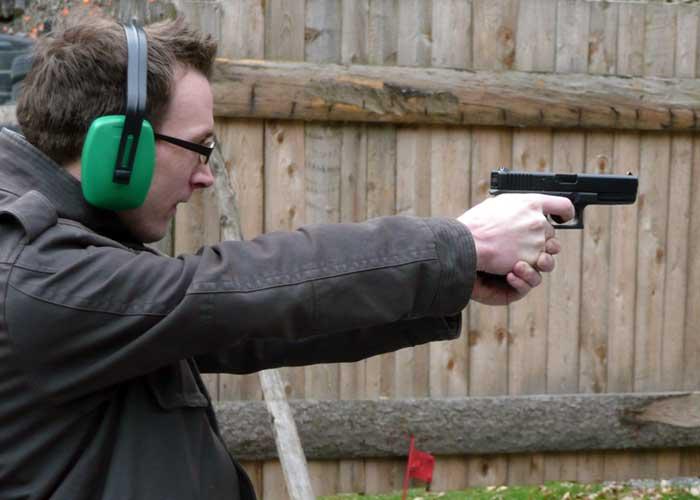

Supplement: Supplementary file 1 — Supplementary Information 1. [file 41598_2023_35190_MOESM1_ESM.zip › test/images/armas--708-_jpg.rf.275db9bd0894064b16e8634e583b1b5c.jpg]

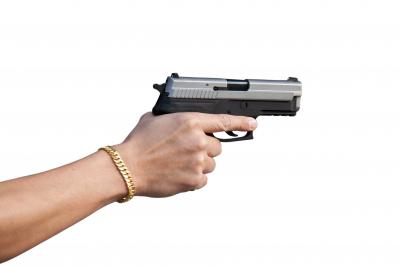

Supplement: Supplementary file 1 — Supplementary Information 1. [file 41598_2023_35190_MOESM1_ESM.zip › test/images/armas--715-_jpg.rf.616a1600eab0590cb7013d981d83435a.jpg]

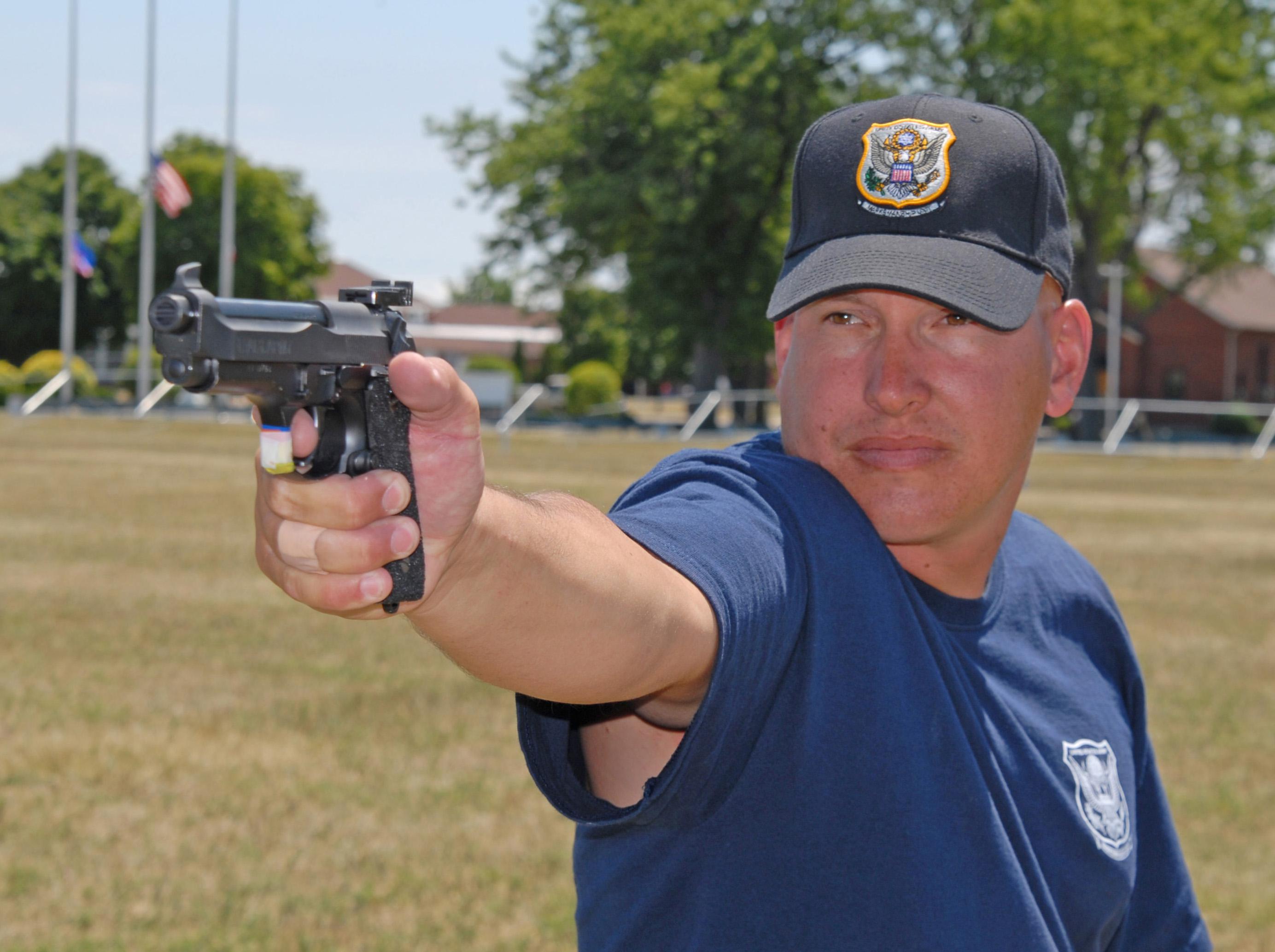

Supplement: Supplementary file 1 — Supplementary Information 1. [file 41598_2023_35190_MOESM1_ESM.zip › test/images/armas--723-_jpg.rf.409ab27296e1782f97845a4eb1dc6a60.jpg]

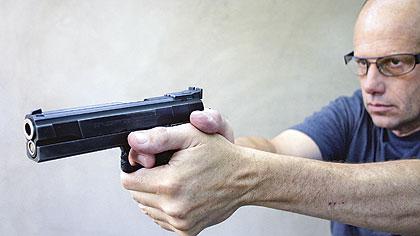

Supplement: Supplementary file 1 — Supplementary Information 1. [file 41598_2023_35190_MOESM1_ESM.zip › test/images/armas--724-_jpg.rf.c2ebf30d1d0d23a136c713a100da300e.jpg]

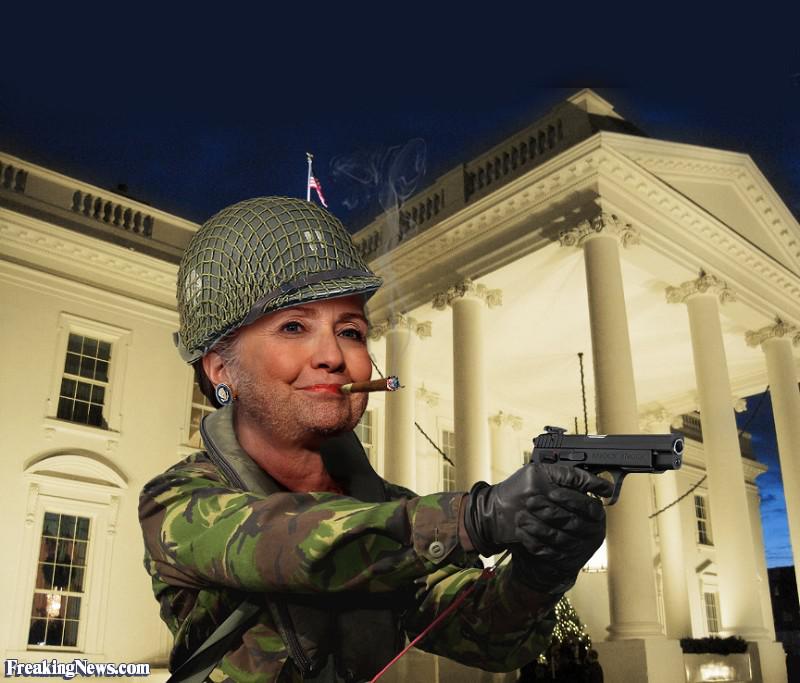

Supplement: Supplementary file 1 — Supplementary Information 1. [file 41598_2023_35190_MOESM1_ESM.zip › test/images/armas--730-_jpg.rf.f4caabdf28bd2b89cb9322a10b00a4e7.jpg]

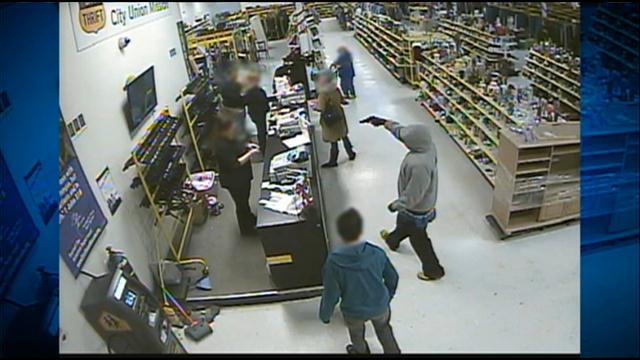

Supplement: Supplementary file 1 — Supplementary Information 1. [file 41598_2023_35190_MOESM1_ESM.zip › test/images/armas--74-_jpg.rf.89fae8a9a71476d103ef8260fb652fdf.jpg]

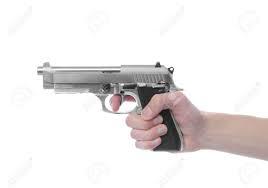

Supplement: Supplementary file 1 — Supplementary Information 1. [file 41598_2023_35190_MOESM1_ESM.zip › test/images/armas--847-_jpg.rf.a65070a971a2c0e8f766613eb63459f2.jpg]

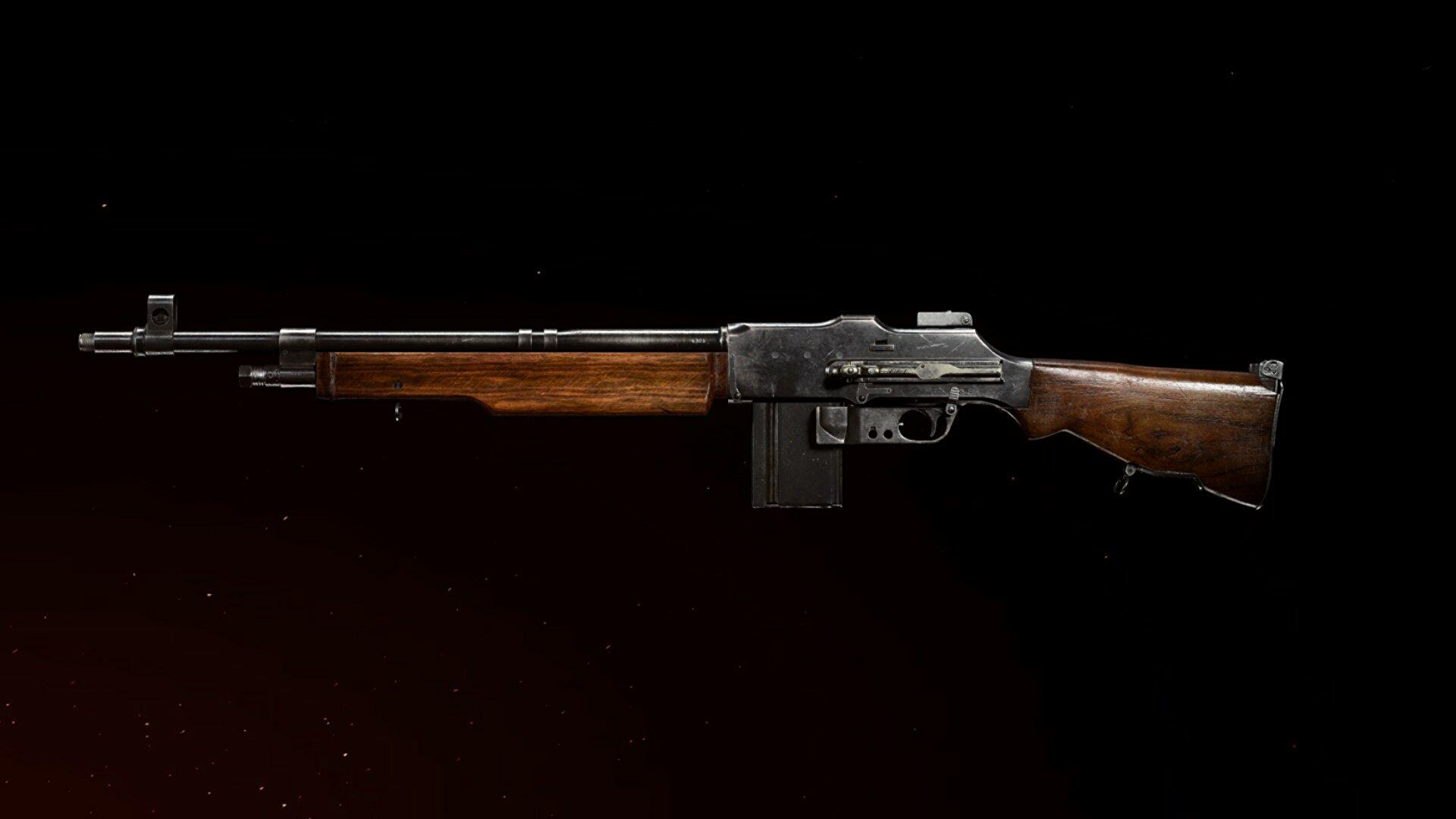

Supplement: Supplementary file 1 — Supplementary Information 1. [file 41598_2023_35190_MOESM1_ESM.zip › test/images/cod-vanguard-bar_jpg.rf.84d775acddad0b8c2d37888fd645dc74.jpg]

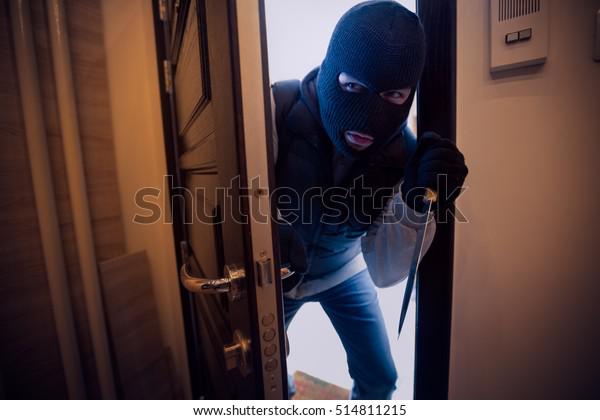

Supplement: Supplementary file 1 — Supplementary Information 1. [file 41598_2023_35190_MOESM1_ESM.zip › test/images/dangerous-burglar-sneaking-into-house-600w-514811215_jpg.rf.7b60345285d6440f285efa59cd484fac.jpg]

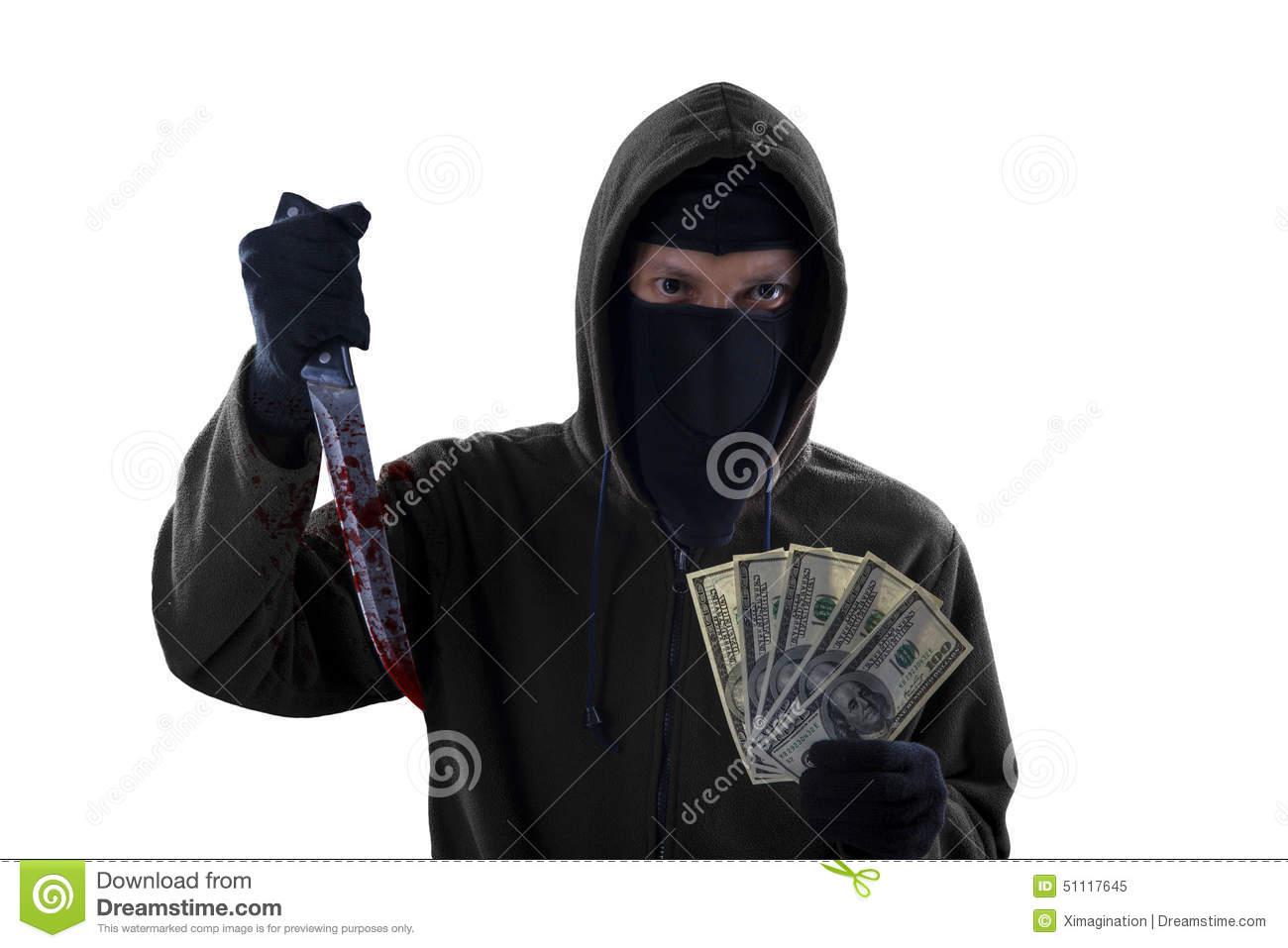

Supplement: Supplementary file 1 — Supplementary Information 1. [file 41598_2023_35190_MOESM1_ESM.zip › test/images/e-dollar-male-wearing-mask-hoodie-holding-money-cash-isolated-white-51117645_jpg.rf.4e7faa56e615cebe4cbba5e6c4a8d307.jpg]

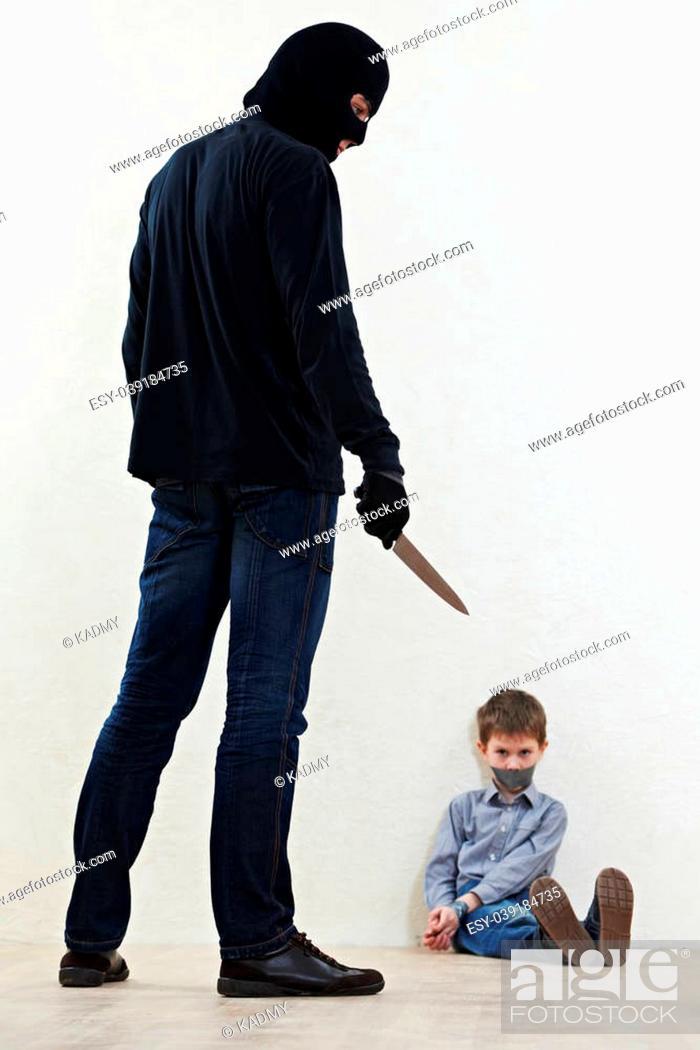

Supplement: Supplementary file 1 — Supplementary Information 1. [file 41598_2023_35190_MOESM1_ESM.zip › test/images/esy-039184735_jpg.rf.1ddcbc00ff8ca0ca14a95baa4975ad5b.jpg]

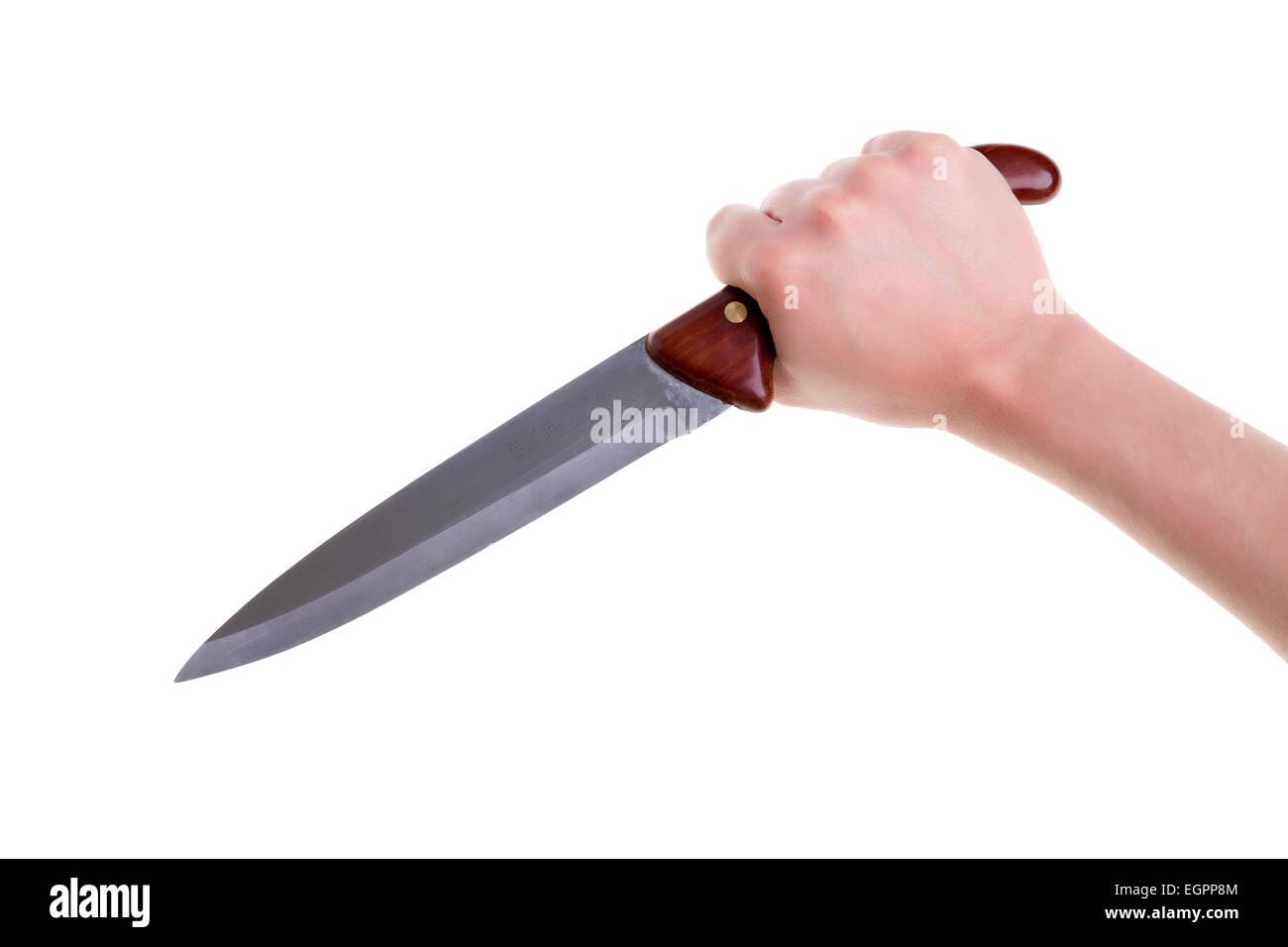

Supplement: Supplementary file 1 — Supplementary Information 1. [file 41598_2023_35190_MOESM1_ESM.zip › test/images/female-hand-holding-sharp-knife-on-white-background-EGPP8M_jpg.rf.cf9f9b09b53eefb719865e04ba27e22c.jpg]

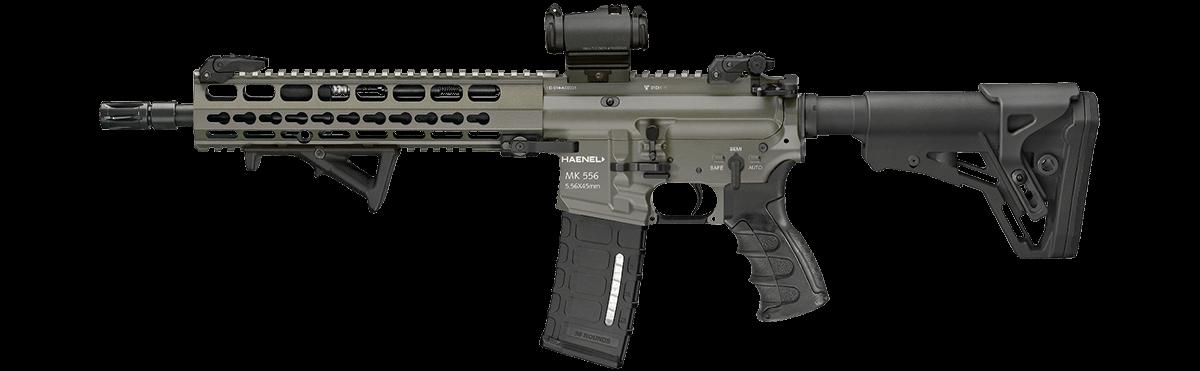

Supplement: Supplementary file 1 — Supplementary Information 1. [file 41598_2023_35190_MOESM1_ESM.zip › test/images/haenel-mk556-vollautomat-gruen-links_png.rf.e0780081ee3cb9e5bac655a4ca6f3887.jpg]

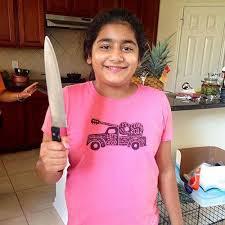

Supplement: Supplementary file 1 — Supplementary Information 1. [file 41598_2023_35190_MOESM1_ESM.zip › test/images/images16_jpg.rf.027c1f228d04fc43b848a224295df628.jpg]

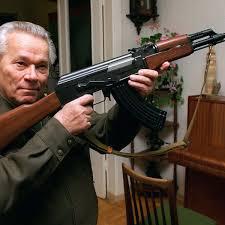

Supplement: Supplementary file 1 — Supplementary Information 1. [file 41598_2023_35190_MOESM1_ESM.zip › test/images/images4_jpg.rf.c97f4aea539c2485b3727fa476095e30.jpg]

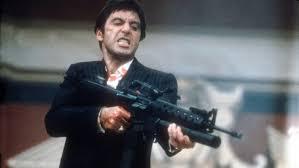

Supplement: Supplementary file 1 — Supplementary Information 1. [file 41598_2023_35190_MOESM1_ESM.zip › test/images/images_jpg.rf.1468e22dbeaccca64b58e95069e27c03.jpg]

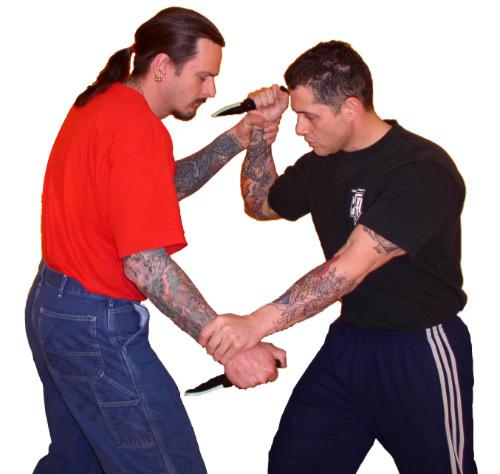

Supplement: Supplementary file 1 — Supplementary Information 1. [file 41598_2023_35190_MOESM1_ESM.zip › test/images/knife-fighting-cqc_jpg.rf.4c618b19a74427836d1b99e3cf9ba780.jpg]

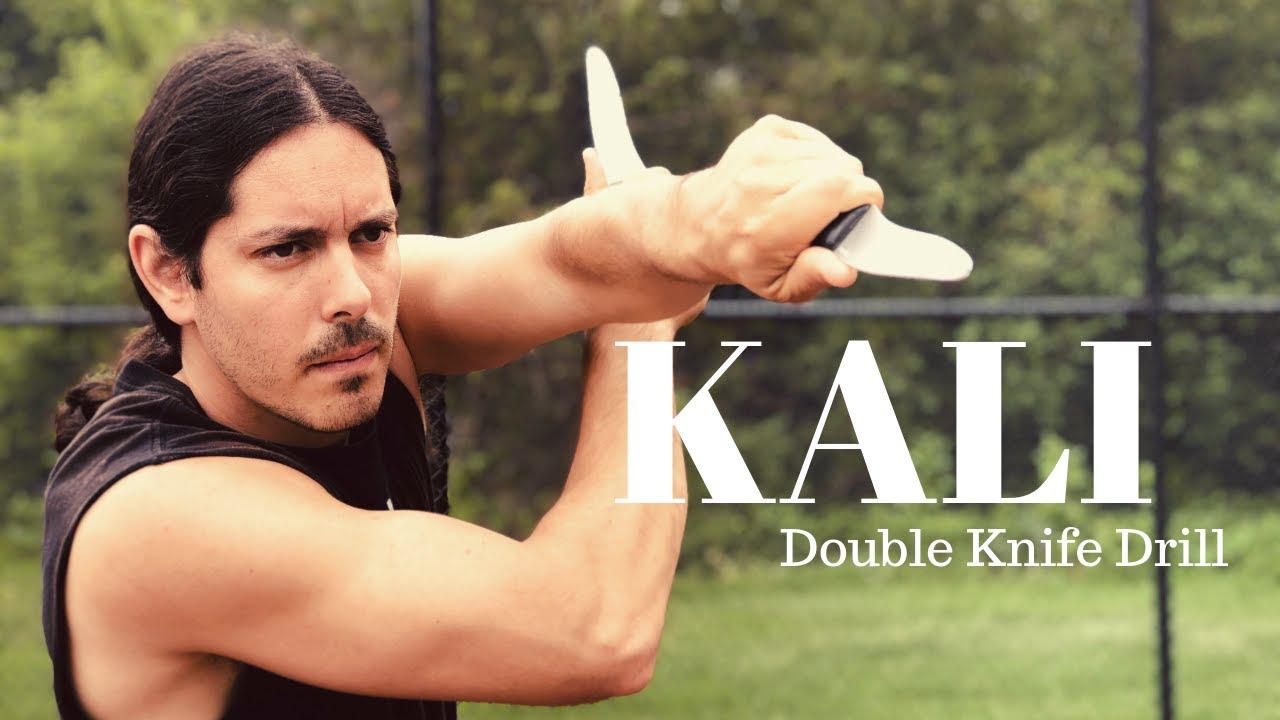

Supplement: Supplementary file 1 — Supplementary Information 1. [file 41598_2023_35190_MOESM1_ESM.zip › test/images/maxresdefault_jpg.rf.e61909d436fde2ca46cceadc0bb94b27.jpg]

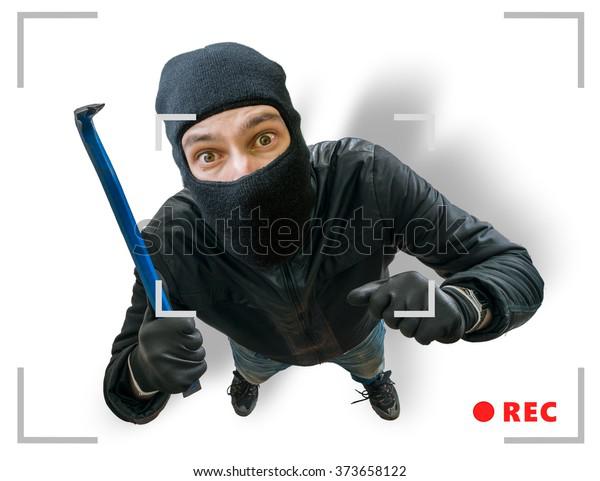

Supplement: Supplementary file 1 — Supplementary Information 1. [file 41598_2023_35190_MOESM1_ESM.zip › test/images/my_dataset_weapon-442_jpg.rf.0bc6d854c53575658c65eb5031df47fb.jpg]

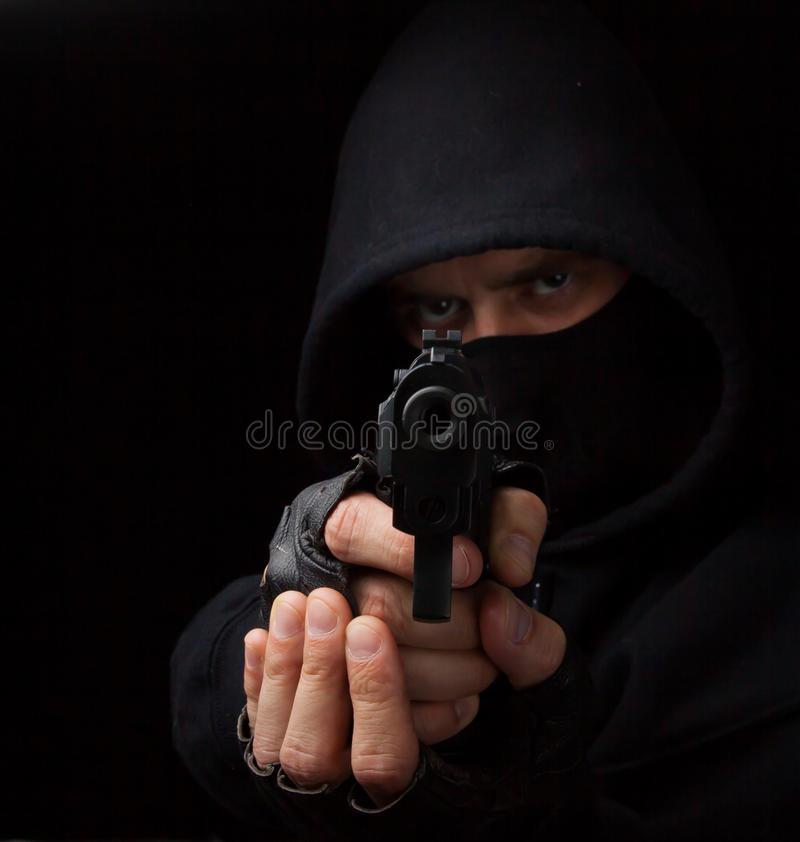

Supplement: Supplementary file 1 — Supplementary Information 1. [file 41598_2023_35190_MOESM1_ESM.zip › test/images/my_dataset_weapon-443_jpg.rf.e9564d3f0eb4eb6c869aea6b9e4da3e7.jpg]

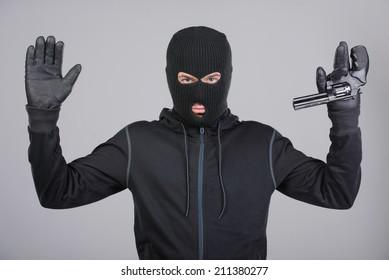

Supplement: Supplementary file 1 — Supplementary Information 1. [file 41598_2023_35190_MOESM1_ESM.zip › test/images/my_dataset_weapon-444_jpg.rf.63dcad72a8822e14334e85cf7fbe0cb2.jpg]

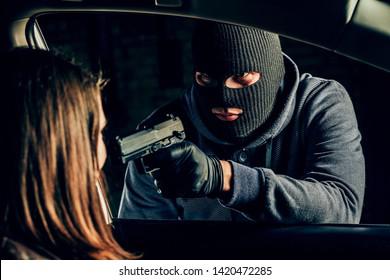

Supplement: Supplementary file 1 — Supplementary Information 1. [file 41598_2023_35190_MOESM1_ESM.zip › test/images/my_dataset_weapon-446_jpg.rf.9322b31ba166d0ffadb43a26b2b29446.jpg]

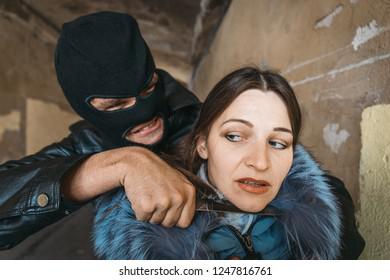

Supplement: Supplementary file 1 — Supplementary Information 1. [file 41598_2023_35190_MOESM1_ESM.zip › test/images/my_dataset_weapon-448_jpg.rf.dfe38727b9ed5d22bf5297fd86bea54d.jpg]

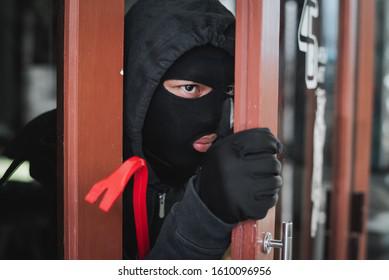

Supplement: Supplementary file 1 — Supplementary Information 1. [file 41598_2023_35190_MOESM1_ESM.zip › test/images/my_dataset_weapon-449_jpg.rf.a72f270b7b8131cabf8e47687be426bf.jpg]

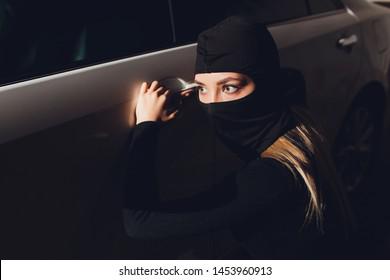

Supplement: Supplementary file 1 — Supplementary Information 1. [file 41598_2023_35190_MOESM1_ESM.zip › test/images/my_dataset_weapon-450_jpg.rf.77d06ca94b64b959ef5787fd787ea79d.jpg]

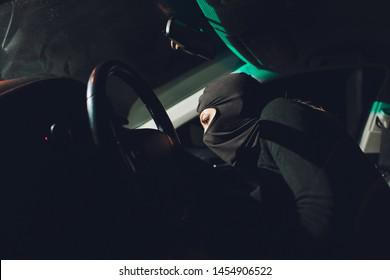

Supplement: Supplementary file 1 — Supplementary Information 1. [file 41598_2023_35190_MOESM1_ESM.zip › test/images/my_dataset_weapon-451_jpg.rf.729a43e19b99cc2b2c11c8b38aebda28.jpg]

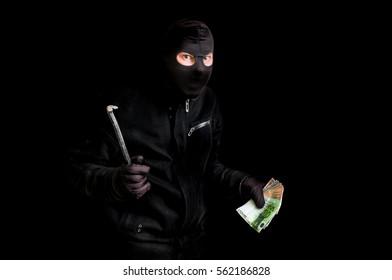

Supplement: Supplementary file 1 — Supplementary Information 1. [file 41598_2023_35190_MOESM1_ESM.zip › test/images/my_dataset_weapon-452_jpg.rf.8c411bd7217ecd1daf4b8cfac3cecf5a.jpg]

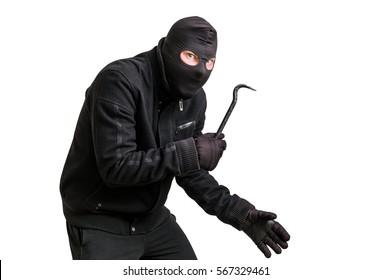

Supplement: Supplementary file 1 — Supplementary Information 1. [file 41598_2023_35190_MOESM1_ESM.zip › test/images/my_dataset_weapon-453_jpg.rf.74e7cd759c97e81e36ff3d4d11b0c794.jpg]

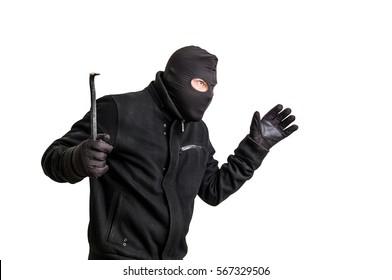

Supplement: Supplementary file 1 — Supplementary Information 1. [file 41598_2023_35190_MOESM1_ESM.zip › test/images/my_dataset_weapon-454_jpg.rf.94d96907417090ff2290eb6f0e079c9e.jpg]

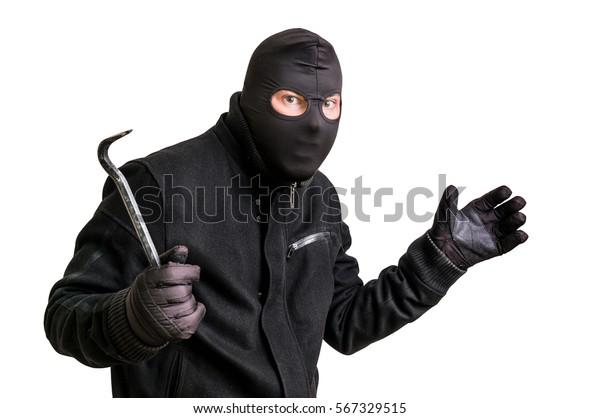

Supplement: Supplementary file 1 — Supplementary Information 1. [file 41598_2023_35190_MOESM1_ESM.zip › test/images/my_dataset_weapon-455_jpg.rf.eb554e7ca8818e0e0c5a7b93d623a455.jpg]

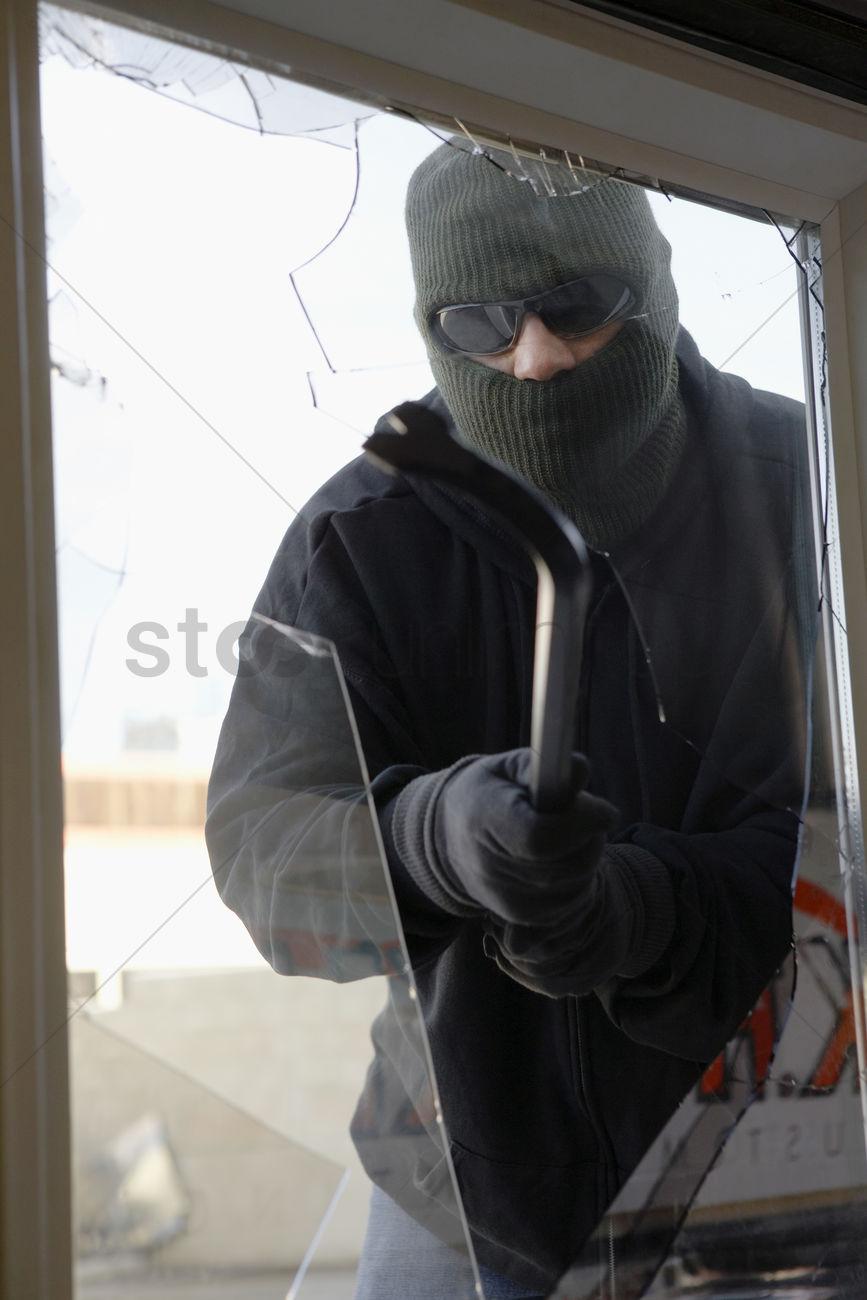

Supplement: Supplementary file 1 — Supplementary Information 1. [file 41598_2023_35190_MOESM1_ESM.zip › test/images/my_dataset_weapon-456_jpg.rf.51f12f55ae30d551bc0db6c2052853d7.jpg]

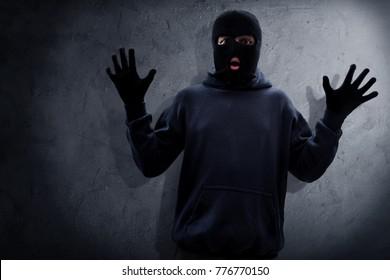

Supplement: Supplementary file 1 — Supplementary Information 1. [file 41598_2023_35190_MOESM1_ESM.zip › test/images/my_dataset_weapon-457_jpg.rf.8aea80f3cb9aa6290d9fece62f4f777b.jpg]

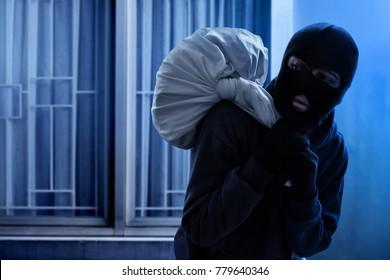

Supplement: Supplementary file 1 — Supplementary Information 1. [file 41598_2023_35190_MOESM1_ESM.zip › test/images/my_dataset_weapon-458_jpg.rf.849ce8934cef3c71f296a6e14df4cdb0.jpg]

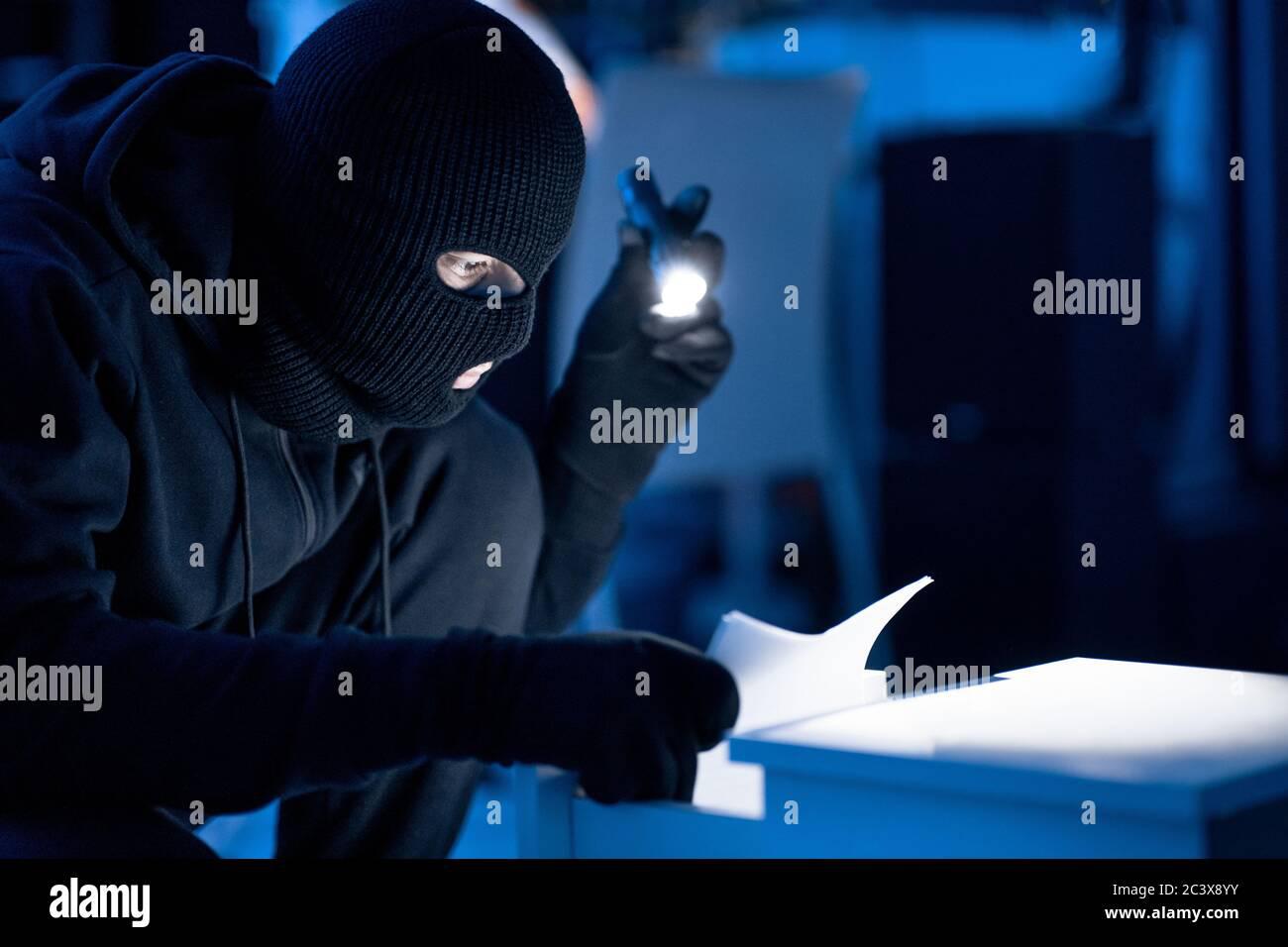

Supplement: Supplementary file 1 — Supplementary Information 1. [file 41598_2023_35190_MOESM1_ESM.zip › test/images/my_dataset_weapon-460_jpg.rf.a1df4008f011f3b66360d2b7a2a04e12.jpg]

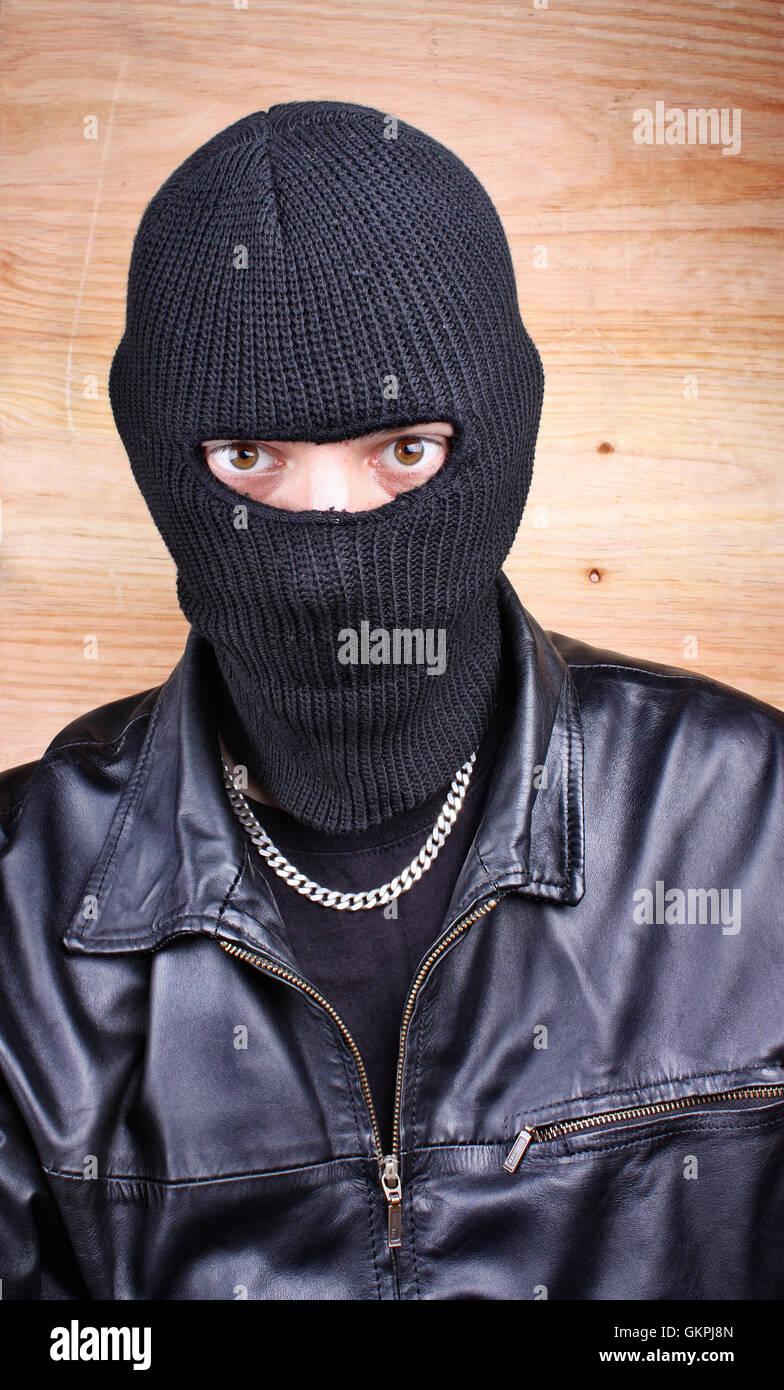

Supplement: Supplementary file 1 — Supplementary Information 1. [file 41598_2023_35190_MOESM1_ESM.zip › test/images/my_dataset_weapon-464_jpg.rf.837902f6d972f3ba12f3c03ff52f6cdb.jpg]

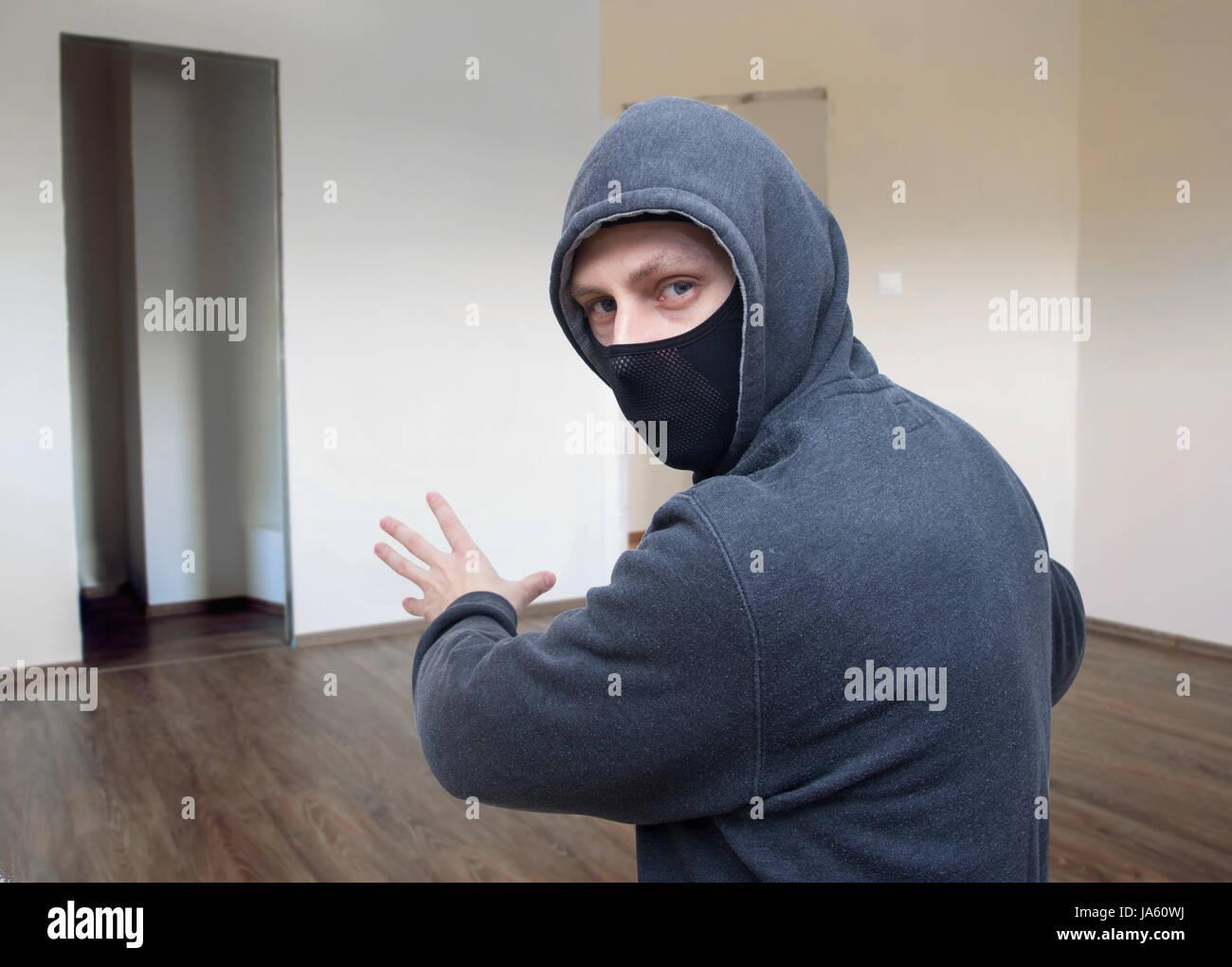

Supplement: Supplementary file 1 — Supplementary Information 1. [file 41598_2023_35190_MOESM1_ESM.zip › test/images/my_dataset_weapon-465_jpg.rf.e7caa0b56dfd9e0da0a9ee5d9425ac4a.jpg]

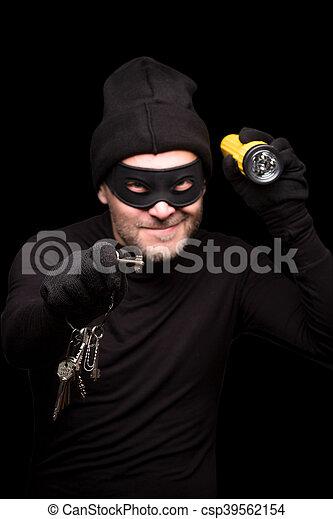

Supplement: Supplementary file 1 — Supplementary Information 1. [file 41598_2023_35190_MOESM1_ESM.zip › test/images/my_dataset_weapon-466_jpg.rf.ec0c4acccfae4ed053b1590f19dcea6c.jpg]

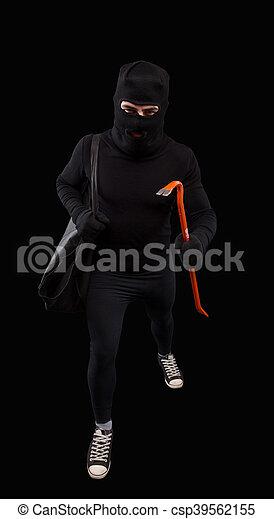

Supplement: Supplementary file 1 — Supplementary Information 1. [file 41598_2023_35190_MOESM1_ESM.zip › test/images/my_dataset_weapon-467_jpg.rf.0263b6a668246e7f1e66d8d3746b0173.jpg]

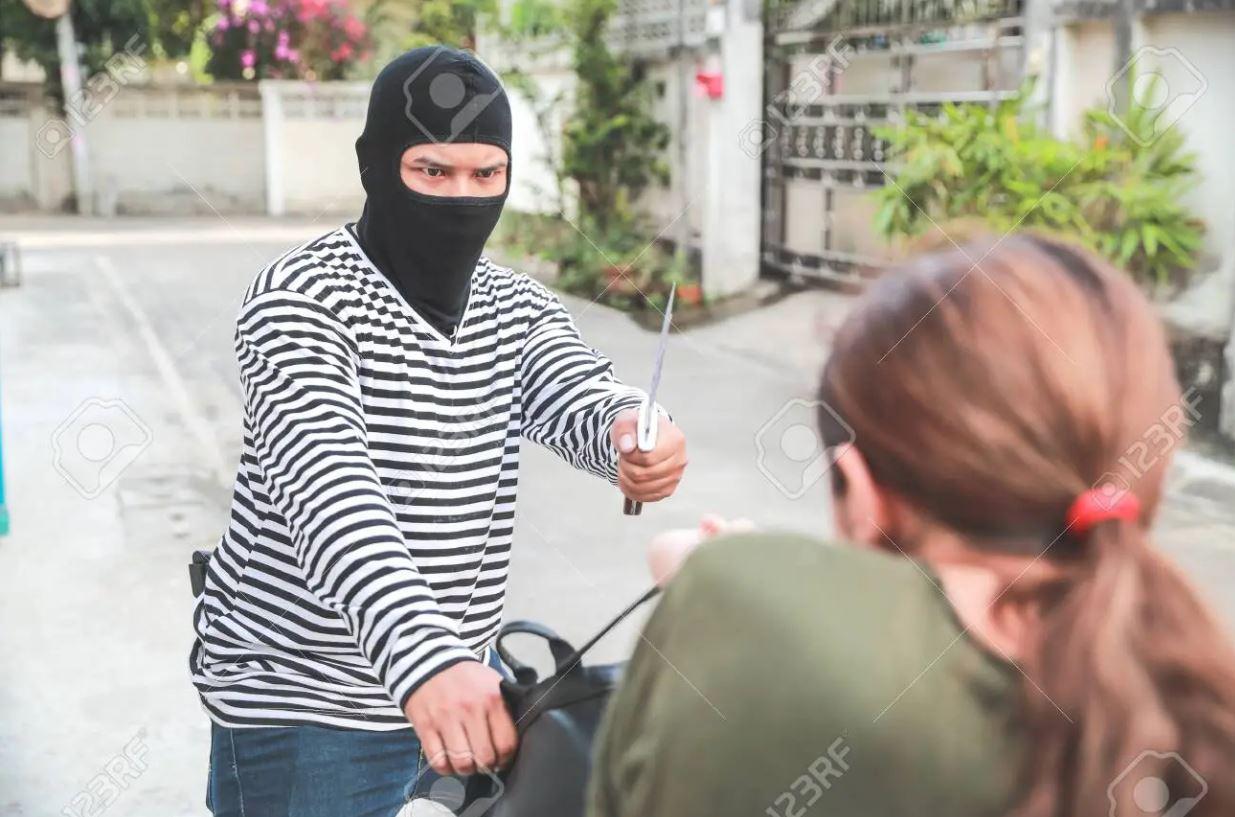

Supplement: Supplementary file 2 — Supplementary Information 2. [file 41598_2023_35190_MOESM2_ESM.zip › test/images/Caapture_JPG.rf.b27a3d51c7f446cb63428828eb2122f0.jpg]
