# Supplementary figures and images for: Improving video surveillance systems in banks using deep learning techniques (part 1 of 4)
Source: Sci Rep. 2023 May 16;13:7911. doi: 10.1038/s41598-023-35190-9 (PMC10188611; doi:10.1038/s41598-023-35190-9)

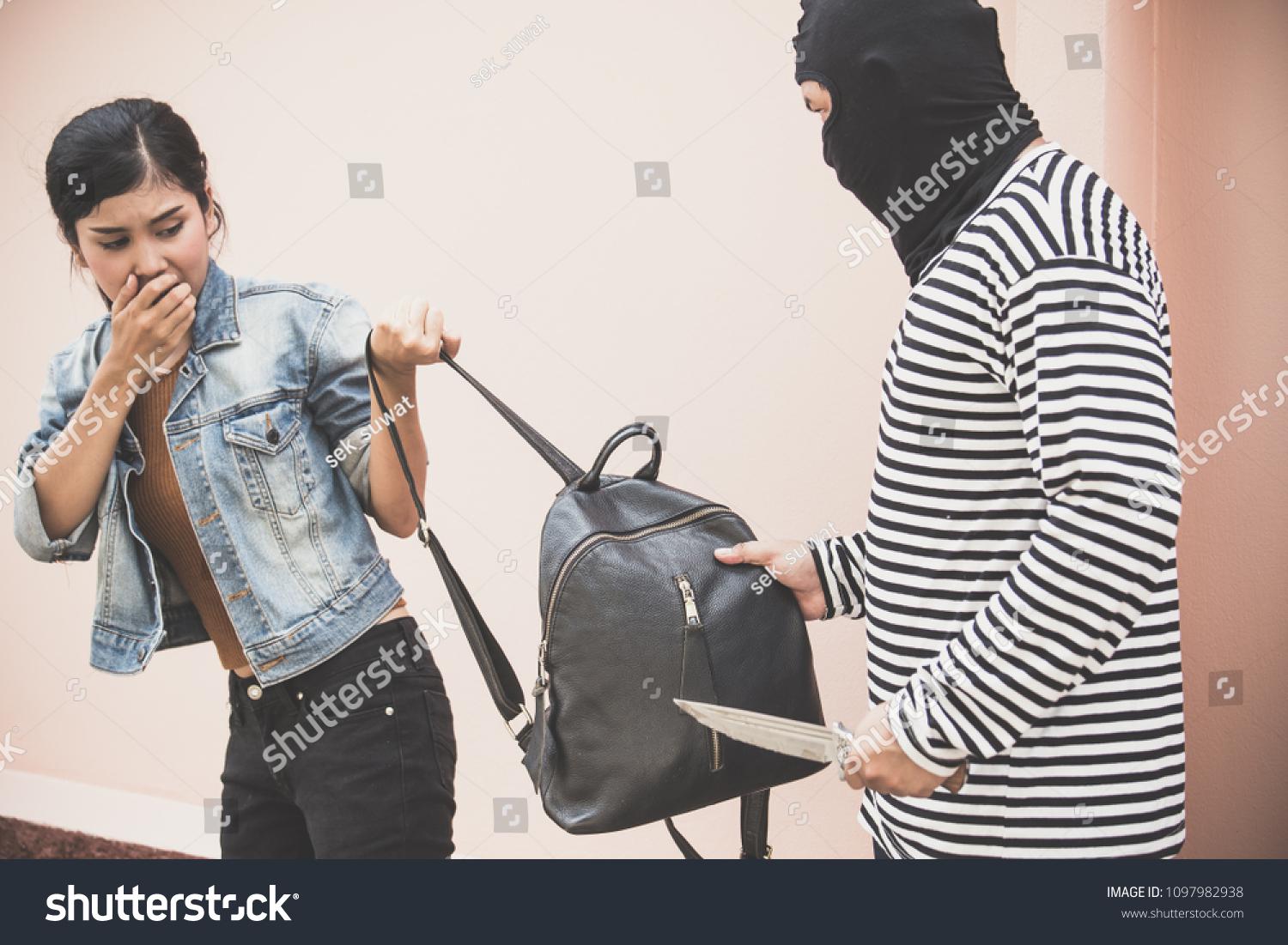

Supplement: Supplementary file 1 — Supplementary Information 1. [file 41598_2023_35190_MOESM1_ESM.zip › test/images/-robber-with-knife-attacking-shoulder-bag-robber-or-thief-holding-1097982938_jpg.rf.5e0ed7086a407d83cc1edab95c0a2347.jpg]

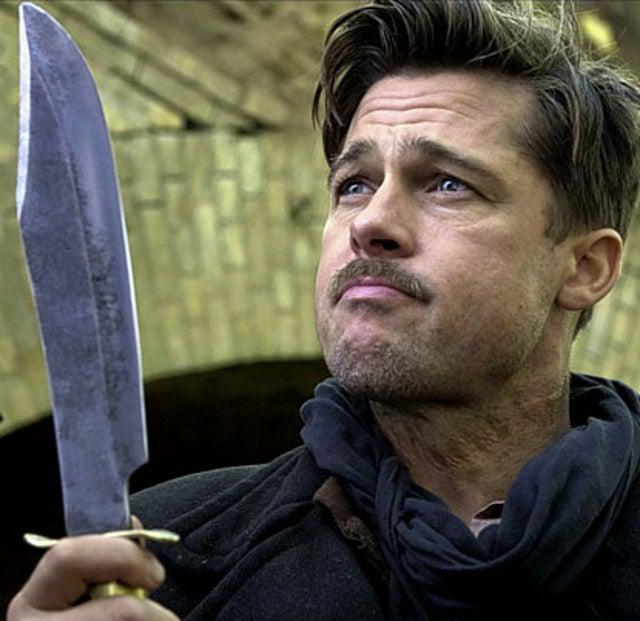

Supplement: Supplementary file 1 — Supplementary Information 1. [file 41598_2023_35190_MOESM1_ESM.zip › test/images/12-brad-INGLOURIOUS-B-415_jpg.rf.43d9080d949b422c94f51a946cfd67fe.jpg]

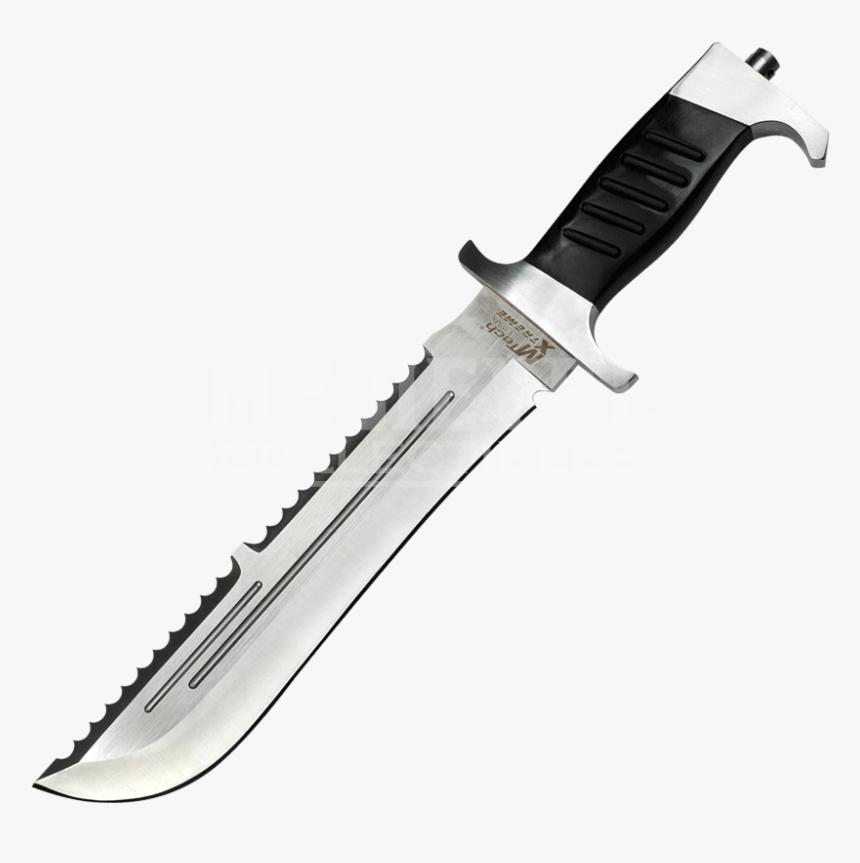

Supplement: Supplementary file 1 — Supplementary Information 1. [file 41598_2023_35190_MOESM1_ESM.zip › test/images/169-1697990_road-warrior-combat-knife-combat-knife-transparent-hd_png.rf.43eedcb33d51e6ae31d09a959e8217ea.jpg]

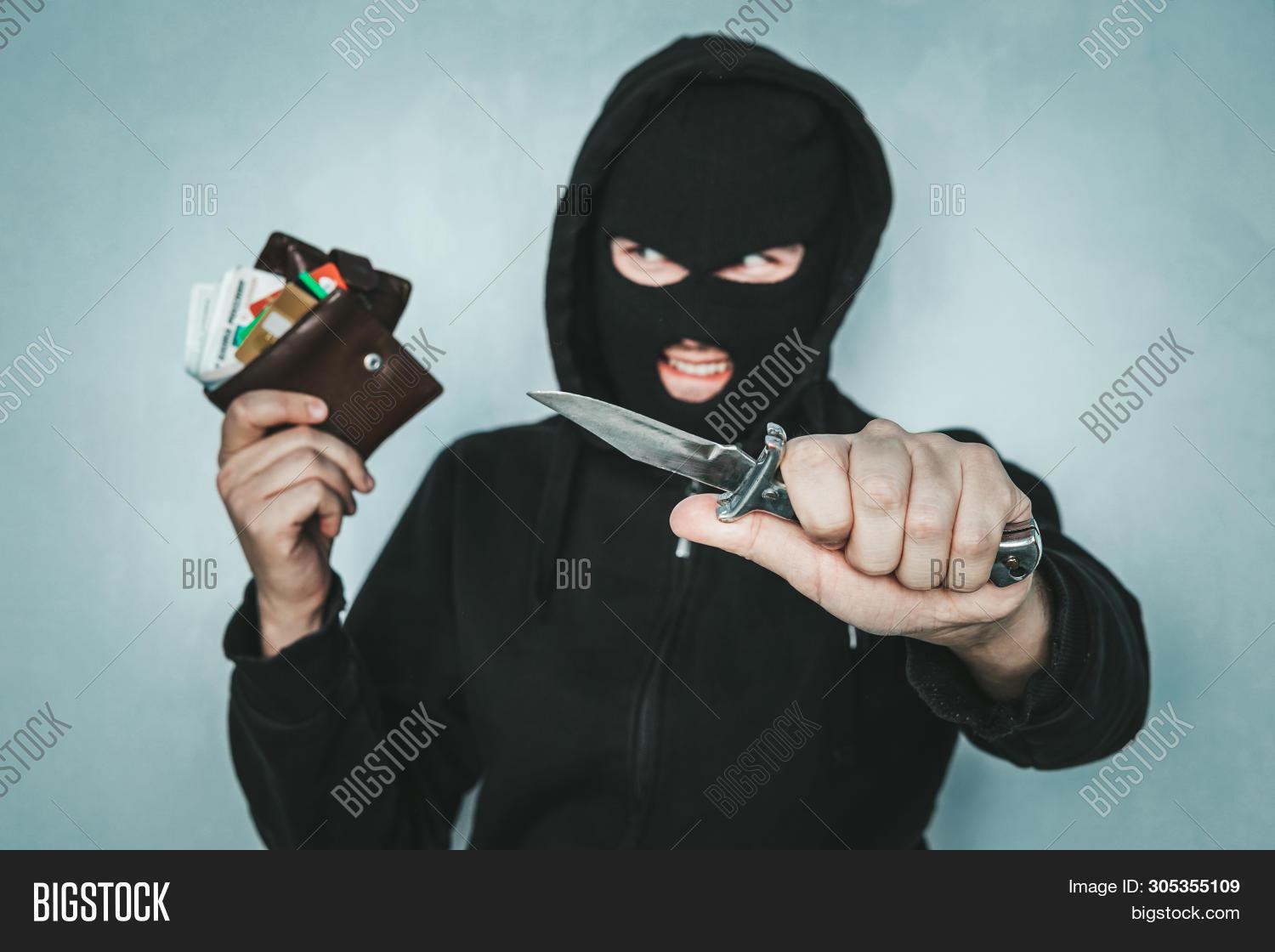

Supplement: Supplementary file 1 — Supplementary Information 1. [file 41598_2023_35190_MOESM1_ESM.zip › test/images/305355109_jpg.rf.12ade91ea1064e8211bbde255c3a95e8.jpg]

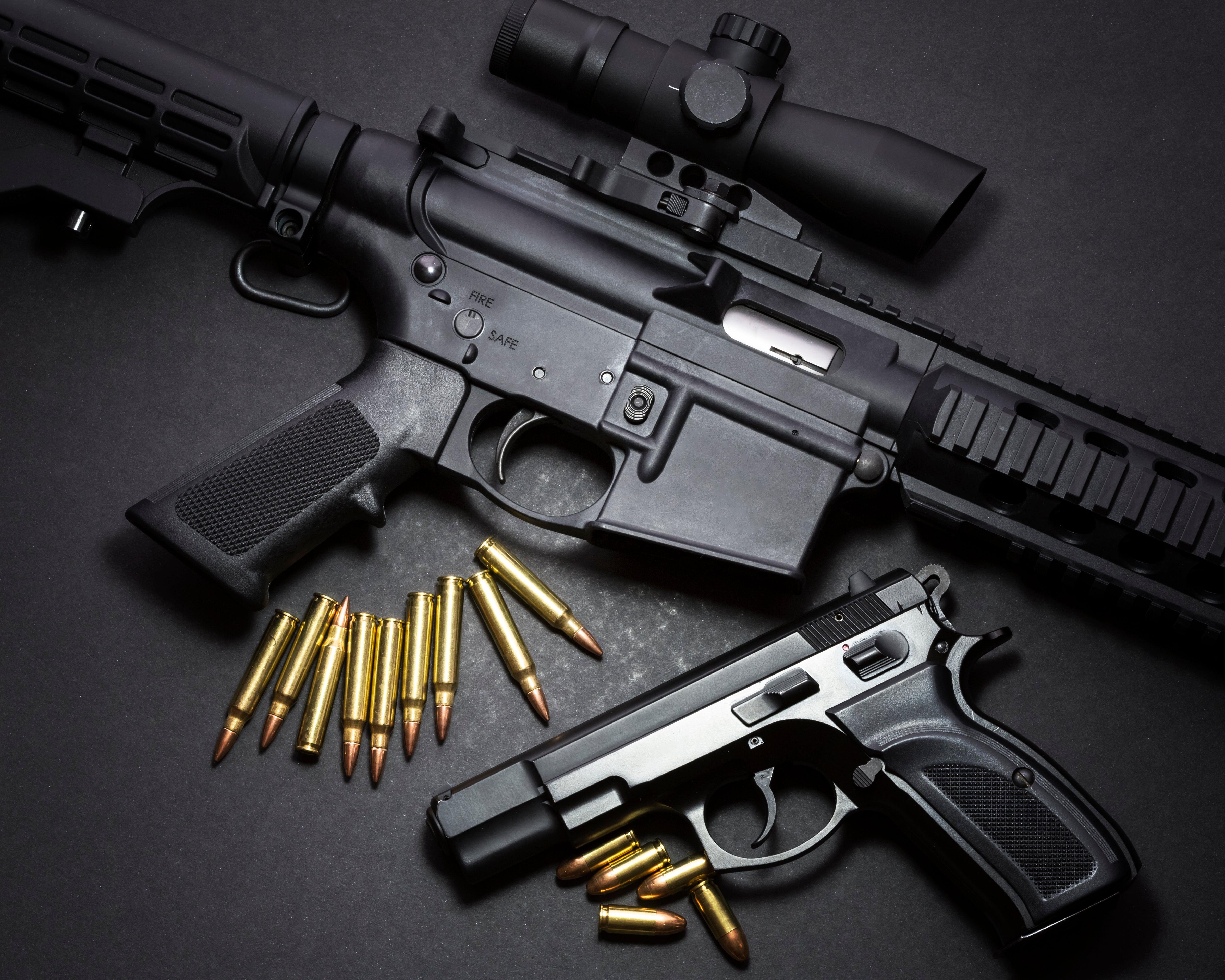

Supplement: Supplementary file 1 — Supplementary Information 1. [file 41598_2023_35190_MOESM1_ESM.zip › test/images/32472ED8-8A08-49F5-9A2AB70DA387E5B7_source_jpg.rf.5cc59d66870ae056f5b4615b31bdfaef.jpg]

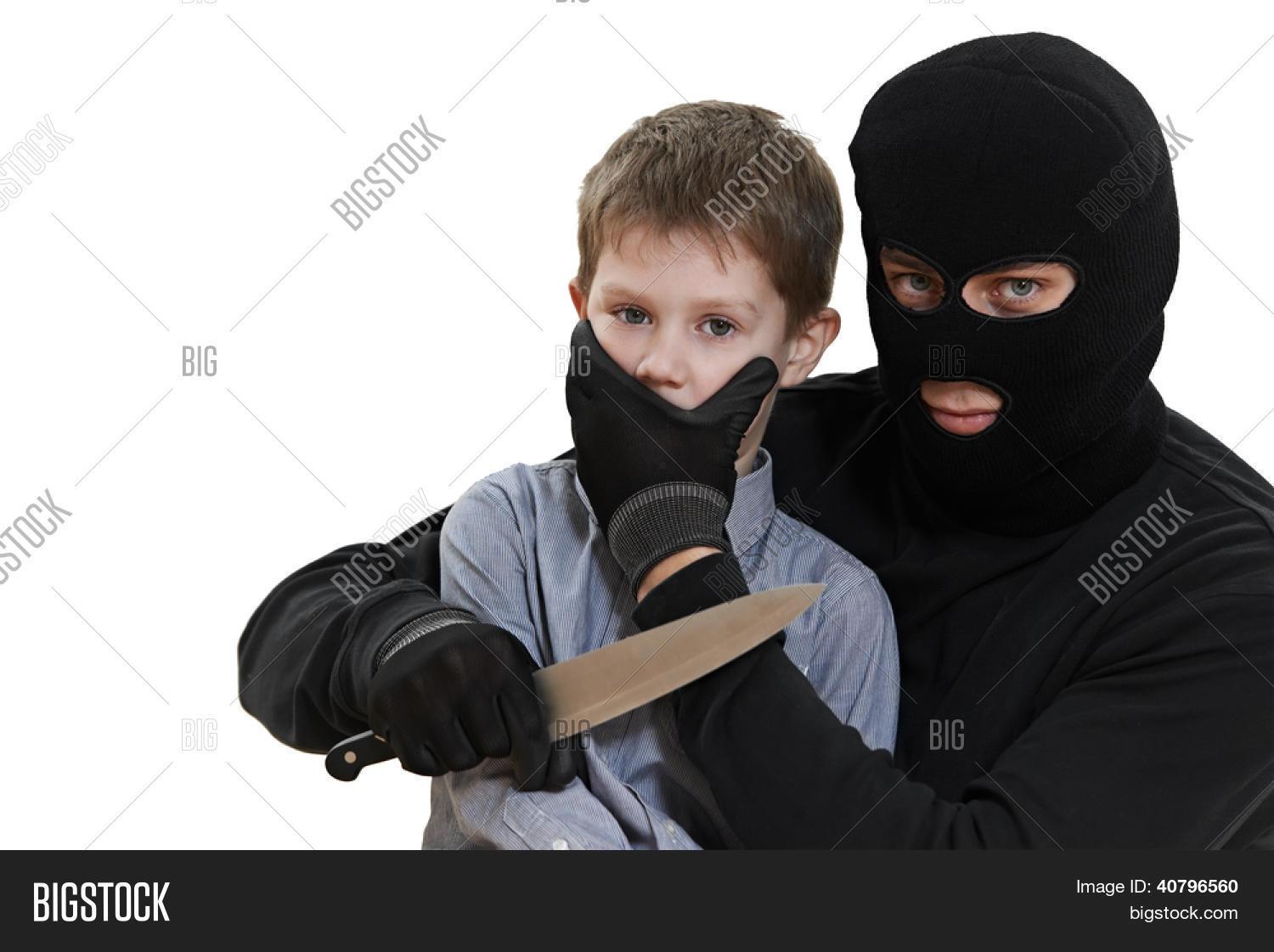

Supplement: Supplementary file 1 — Supplementary Information 1. [file 41598_2023_35190_MOESM1_ESM.zip › test/images/40796560_jpg.rf.22853a143e7a0436dee2f83e92fe6364.jpg]

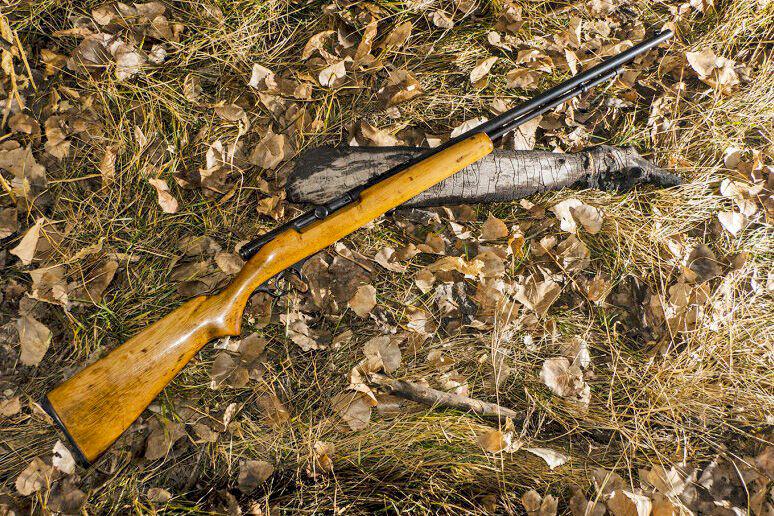

Supplement: Supplementary file 1 — Supplementary Information 1. [file 41598_2023_35190_MOESM1_ESM.zip › test/images/6235773f2742b-image_jpg.rf.648c3af3d5c5f2f64ff8849e7e8168a9.jpg]

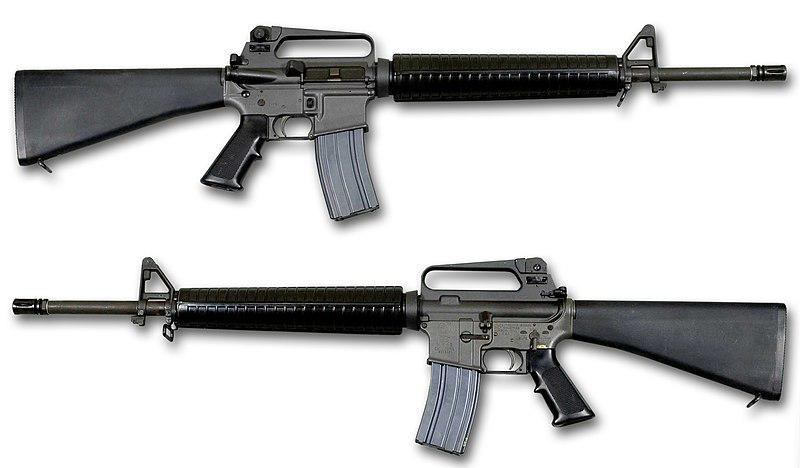

Supplement: Supplementary file 1 — Supplementary Information 1. [file 41598_2023_35190_MOESM1_ESM.zip › test/images/800px-M16A2_noBG_jpg.rf.2741c1fd11a128cb84f2ff485b180c9c.jpg]

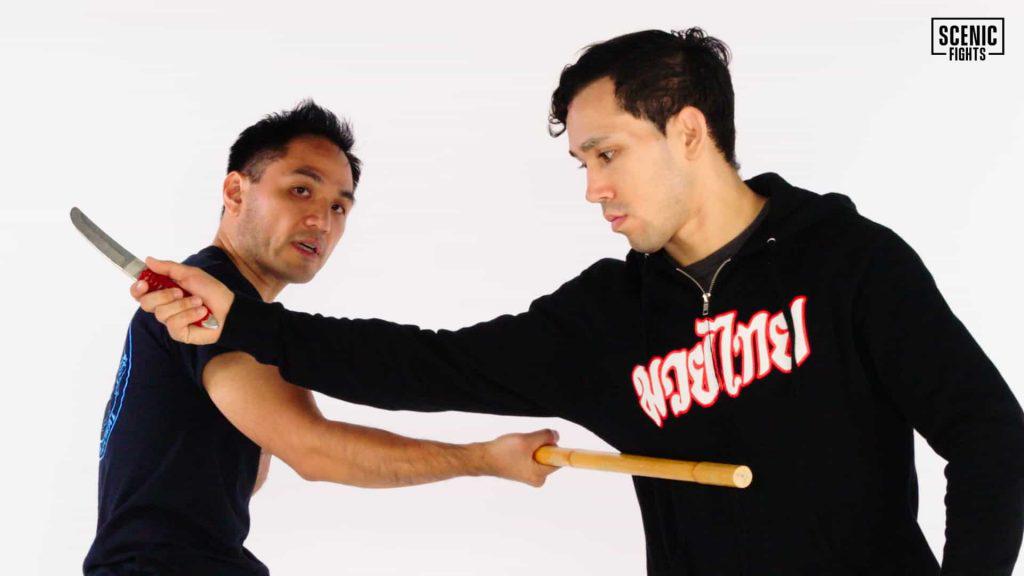

Supplement: Supplementary file 1 — Supplementary Information 1. [file 41598_2023_35190_MOESM1_ESM.zip › test/images/9-3-1024x576_jpg.rf.f80a5d200be4c4efa748024dae817838.jpg]

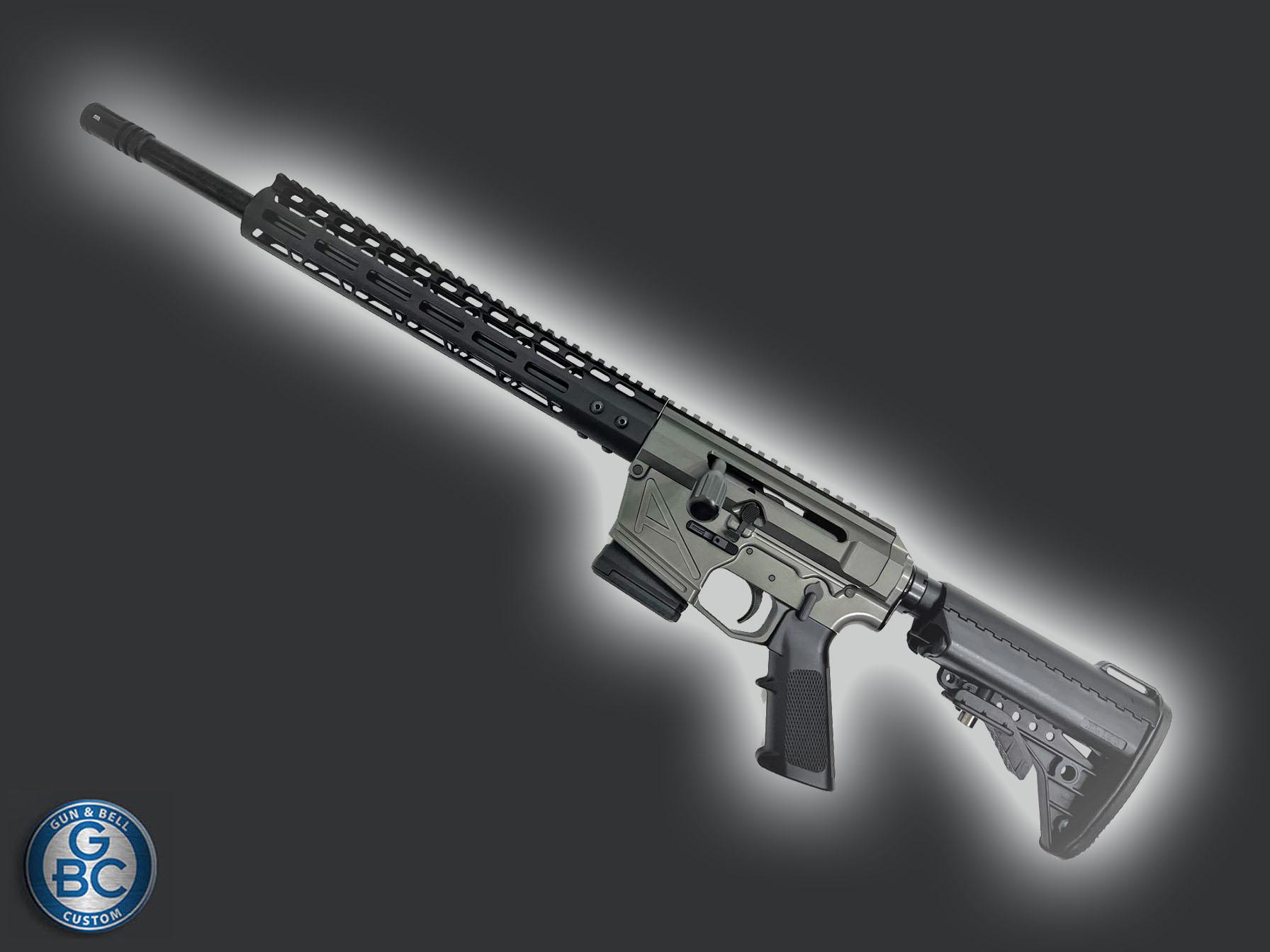

Supplement: Supplementary file 1 — Supplementary Information 1. [file 41598_2023_35190_MOESM1_ESM.zip › test/images/ABYSS_NH_LH_-JPG_jpg.rf.f56a8015c1a80c7e9417252242355361.jpg]

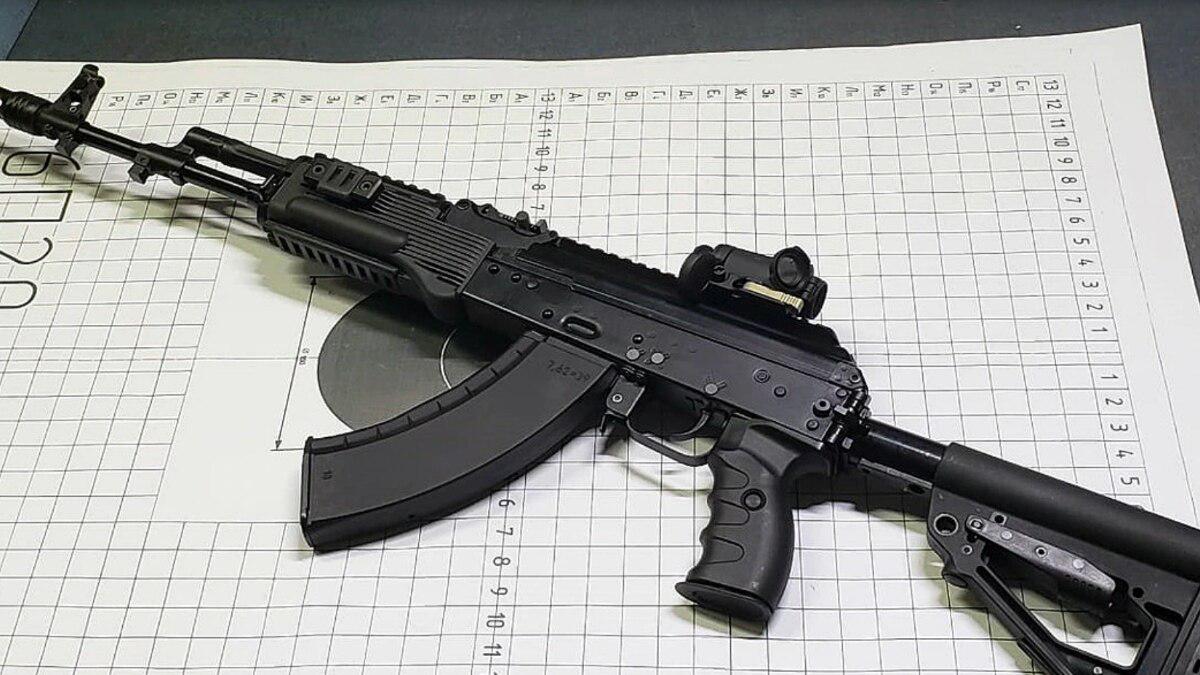

Supplement: Supplementary file 1 — Supplementary Information 1. [file 41598_2023_35190_MOESM1_ESM.zip › test/images/AK203_assault_rifle_2_1200x768_png.rf.535a9ccd9cb49d11d783fc2a5162dd77.jpg]

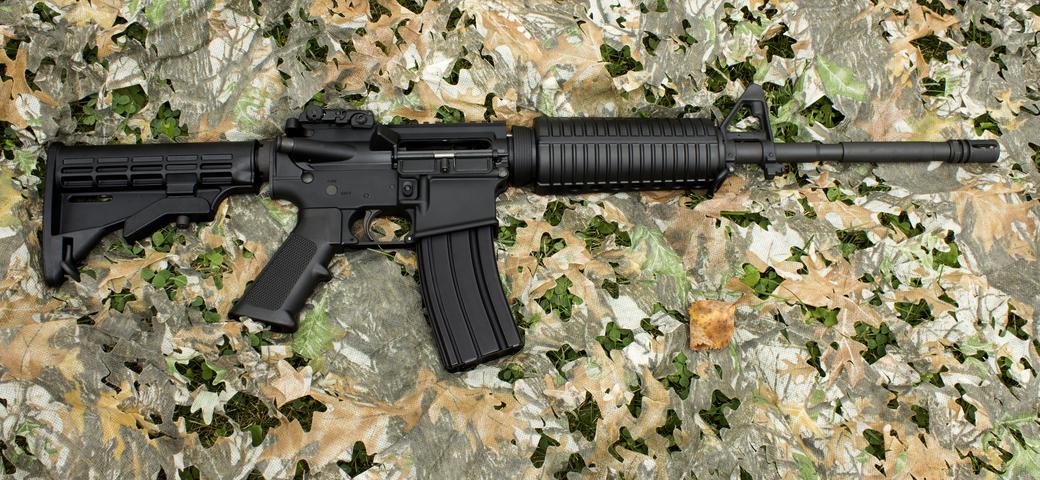

Supplement: Supplementary file 1 — Supplementary Information 1. [file 41598_2023_35190_MOESM1_ESM.zip › test/images/AR-15_jpg.rf.a99b581fc9b1366d5a19c458d819248d.jpg]

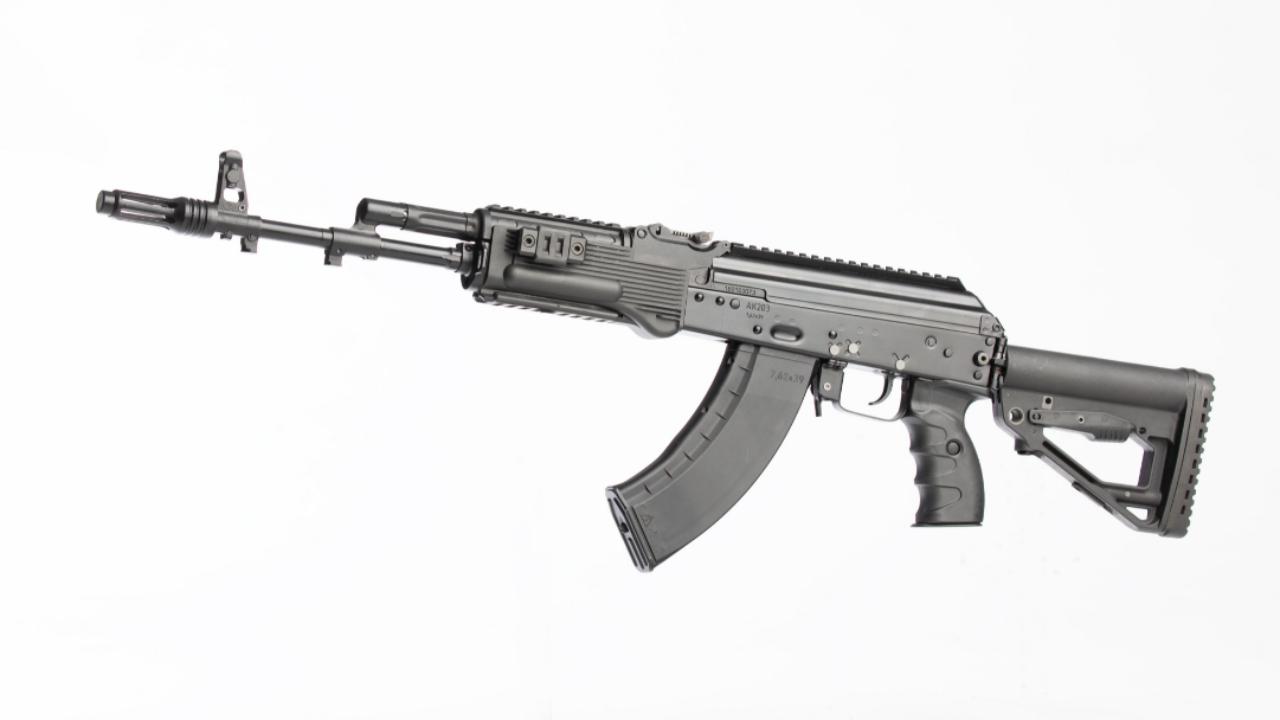

Supplement: Supplementary file 1 — Supplementary Information 1. [file 41598_2023_35190_MOESM1_ESM.zip › test/images/Capture-2-1280x720_png.rf.078e135179c6f9d806f51101d5dde3d4.jpg]

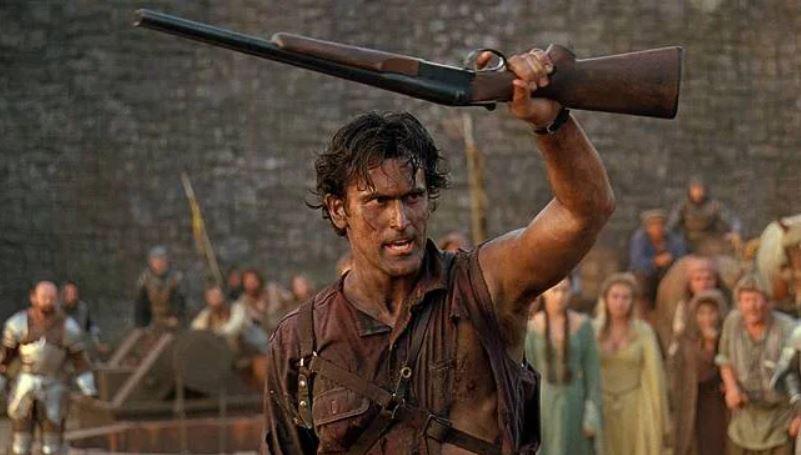

Supplement: Supplementary file 1 — Supplementary Information 1. [file 41598_2023_35190_MOESM1_ESM.zip › test/images/Capture_JPG.rf.2febd2ce4744cce2f5226c7961b9d3c9.jpg]

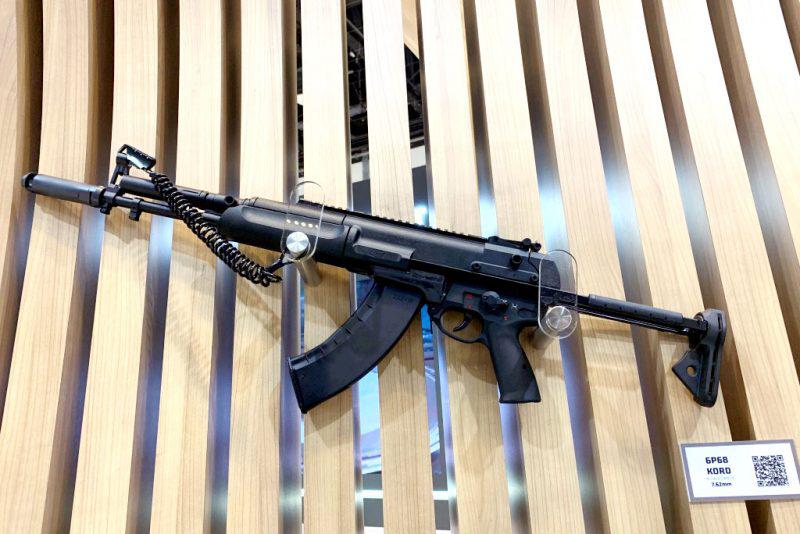

Supplement: Supplementary file 1 — Supplementary Information 1. [file 41598_2023_35190_MOESM1_ESM.zip › test/images/Kalashnikov-Kord-800x534_jpg.rf.15859ede6c2614442bd42dcfb7521e23.jpg]

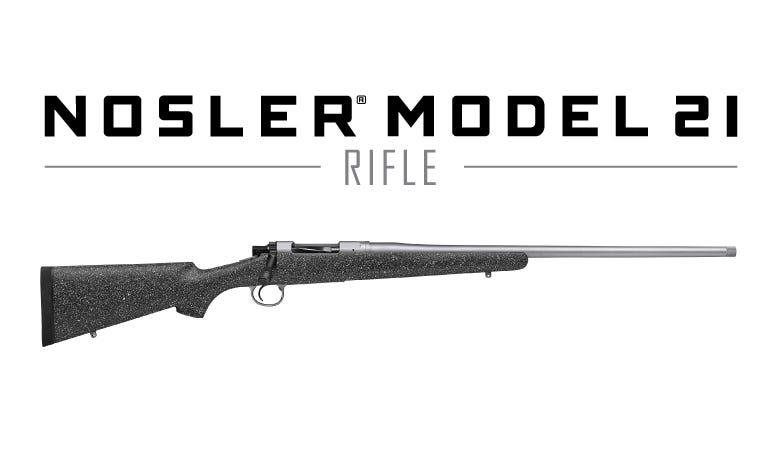

Supplement: Supplementary file 1 — Supplementary Information 1. [file 41598_2023_35190_MOESM1_ESM.zip › test/images/M21-Banner-no-bases-767x472_jpg.rf.5a45dd854df2f7803a91108c67b7d641.jpg]

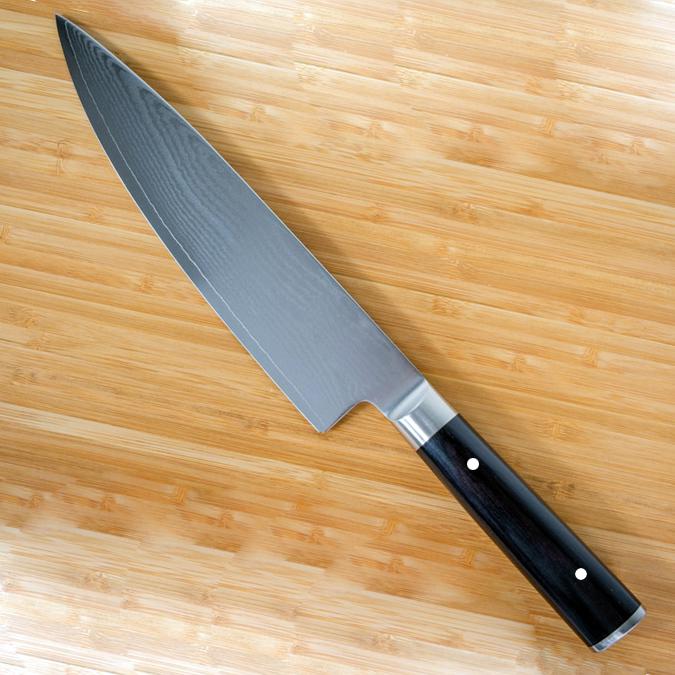

Supplement: Supplementary file 1 — Supplementary Information 1. [file 41598_2023_35190_MOESM1_ESM.zip › test/images/U52377925f1944801a675946ca86f5244b_jpg.rf.23f9722ddb26583f7857048acdd0f213.jpg]

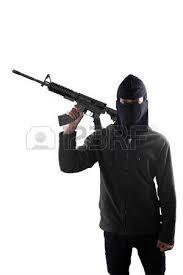

Supplement: Supplementary file 1 — Supplementary Information 1. [file 41598_2023_35190_MOESM1_ESM.zip › test/images/armas--1004-_jpg.rf.0a96e08673b0a0584bf300934cc9d7fe.jpg]

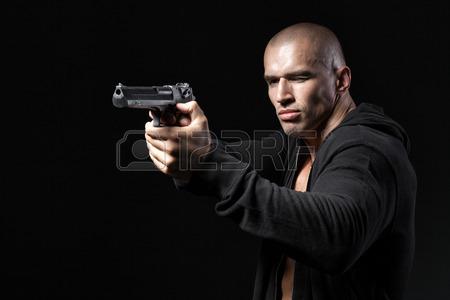

Supplement: Supplementary file 1 — Supplementary Information 1. [file 41598_2023_35190_MOESM1_ESM.zip › test/images/armas--103-_jpg.rf.02029ec27466cddb78d70c8c733a9c92.jpg]

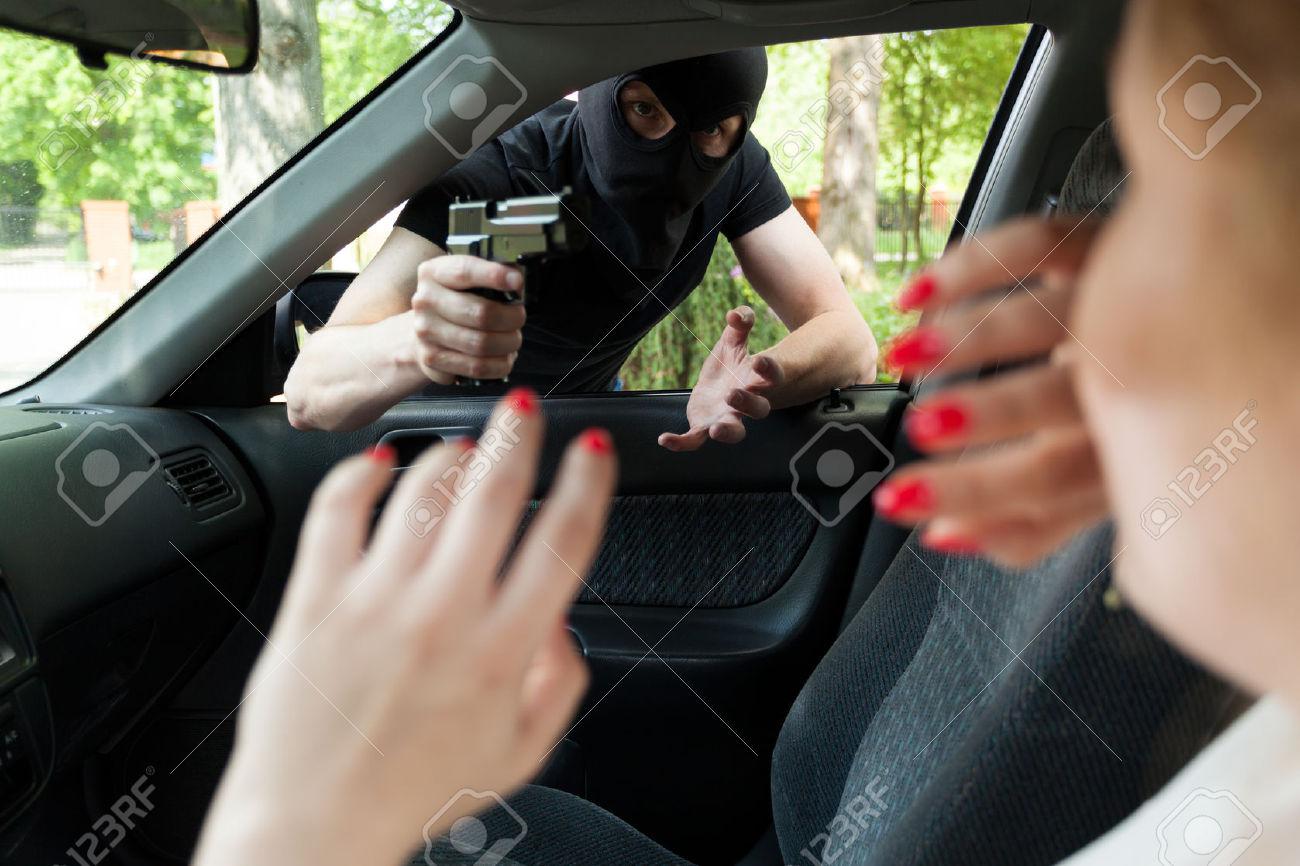

Supplement: Supplementary file 1 — Supplementary Information 1. [file 41598_2023_35190_MOESM1_ESM.zip › test/images/armas--106-_jpg.rf.bebb303a689fbac8f8bd32e05f2aa97b.jpg]

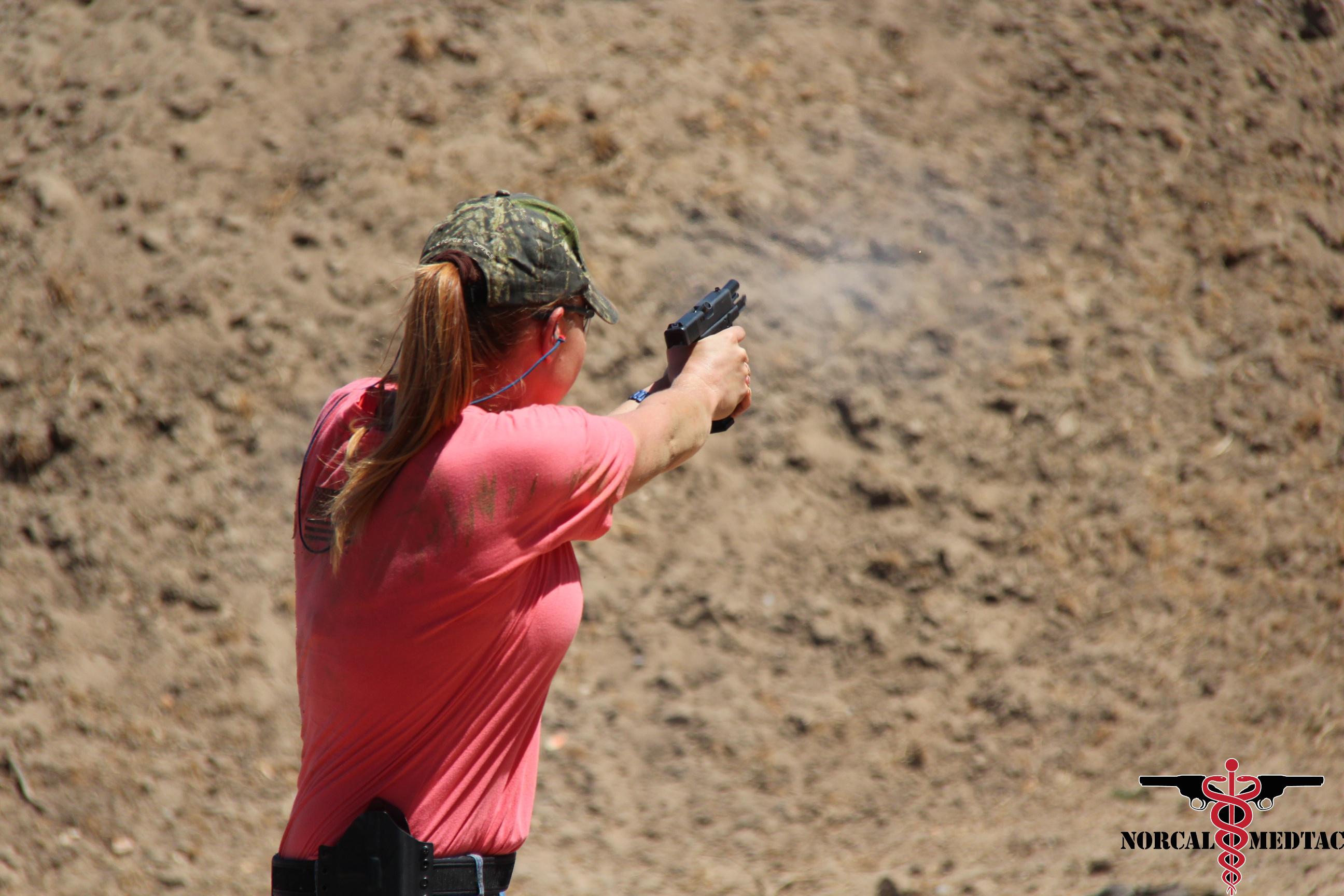

Supplement: Supplementary file 1 — Supplementary Information 1. [file 41598_2023_35190_MOESM1_ESM.zip › test/images/armas--1061-_jpg.rf.8fff66aac21cf596a192f929409a8e5b.jpg]

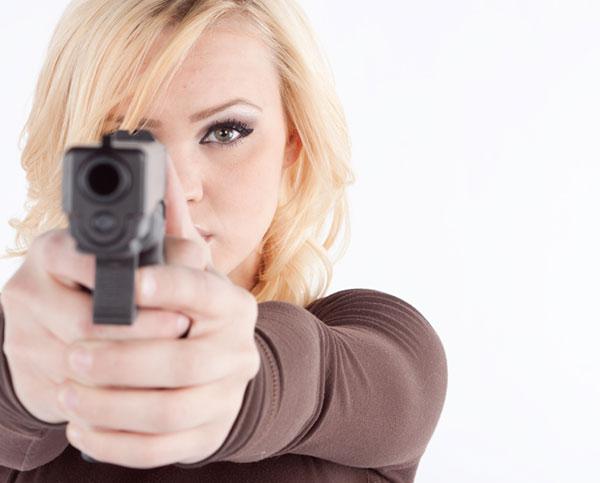

Supplement: Supplementary file 1 — Supplementary Information 1. [file 41598_2023_35190_MOESM1_ESM.zip › test/images/armas--1065-_jpg.rf.cdad25c2f7fabeaae5813805b9f2c0c5.jpg]

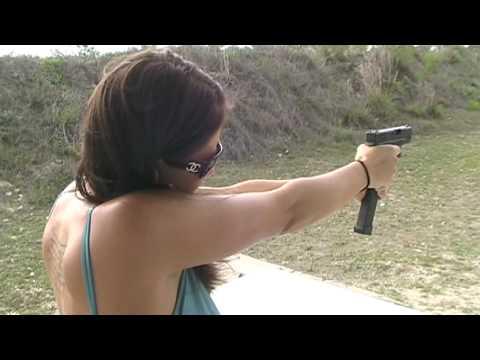

Supplement: Supplementary file 1 — Supplementary Information 1. [file 41598_2023_35190_MOESM1_ESM.zip › test/images/armas--1076-_jpg.rf.e66a9e0f2881fe12a2de661aeab29b3e.jpg]

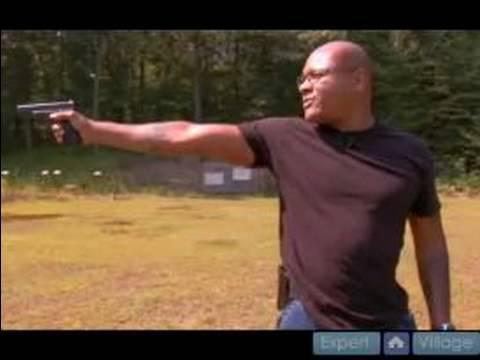

Supplement: Supplementary file 1 — Supplementary Information 1. [file 41598_2023_35190_MOESM1_ESM.zip › test/images/armas--1078-_jpg.rf.daf30fbefd624be0749ee20019a6b83f.jpg]

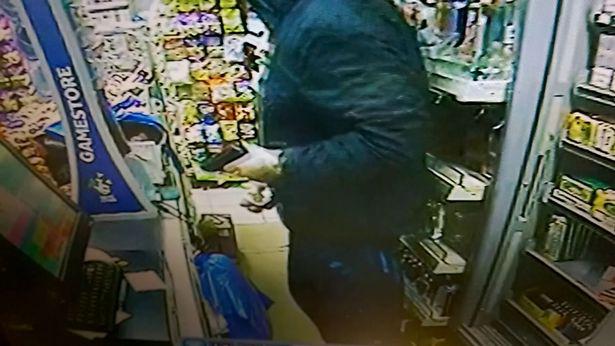

Supplement: Supplementary file 1 — Supplementary Information 1. [file 41598_2023_35190_MOESM1_ESM.zip › test/images/armas--1079-_jpg.rf.c183ea1848caf34836f995900677aa84.jpg]

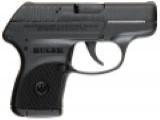

Supplement: Supplementary file 1 — Supplementary Information 1. [file 41598_2023_35190_MOESM1_ESM.zip › test/images/armas--2503-_jpg.rf.c630b3aca1cbcf1e88f3a35b29c89a31.jpg]

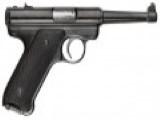

Supplement: Supplementary file 1 — Supplementary Information 1. [file 41598_2023_35190_MOESM1_ESM.zip › test/images/armas--2504-_jpg.rf.2a8bf7c06f2b3dba61797619cc95502b.jpg]

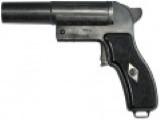

Supplement: Supplementary file 1 — Supplementary Information 1. [file 41598_2023_35190_MOESM1_ESM.zip › test/images/armas--2532-_jpg.rf.e51ef2e01e17da71c9d280a8e64d0e60.jpg]

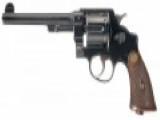

Supplement: Supplementary file 1 — Supplementary Information 1. [file 41598_2023_35190_MOESM1_ESM.zip › test/images/armas--2534-_jpg.rf.7b38d456c1a9421830a6117b4020bc32.jpg]

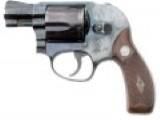

Supplement: Supplementary file 1 — Supplementary Information 1. [file 41598_2023_35190_MOESM1_ESM.zip › test/images/armas--2535-_jpg.rf.ce16bd4d91bd38e6cc8e6acc81daec7a.jpg]

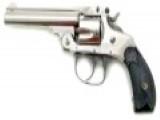

Supplement: Supplementary file 1 — Supplementary Information 1. [file 41598_2023_35190_MOESM1_ESM.zip › test/images/armas--2536-_jpg.rf.9cf9f21ebe07653851a91e63ed8df618.jpg]

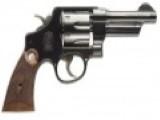

Supplement: Supplementary file 1 — Supplementary Information 1. [file 41598_2023_35190_MOESM1_ESM.zip › test/images/armas--2537-_jpg.rf.e4412251f2070b950be061f24c250174.jpg]

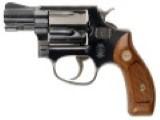

Supplement: Supplementary file 1 — Supplementary Information 1. [file 41598_2023_35190_MOESM1_ESM.zip › test/images/armas--2538-_jpg.rf.fade40fdd5b10cdbd4e0604de0a1618a.jpg]

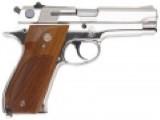

Supplement: Supplementary file 1 — Supplementary Information 1. [file 41598_2023_35190_MOESM1_ESM.zip › test/images/armas--2539-_jpg.rf.0559b359f5e56346b90b3e73c0b7544e.jpg]

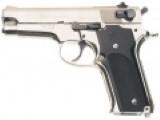

Supplement: Supplementary file 1 — Supplementary Information 1. [file 41598_2023_35190_MOESM1_ESM.zip › test/images/armas--2540-_jpg.rf.4a71dde216c906425c74eb7698c022f5.jpg]

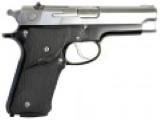

Supplement: Supplementary file 1 — Supplementary Information 1. [file 41598_2023_35190_MOESM1_ESM.zip › test/images/armas--2541-_jpg.rf.39ce055e4b6641b2eb4b11de86f49653.jpg]

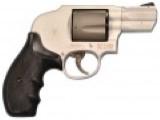

Supplement: Supplementary file 1 — Supplementary Information 1. [file 41598_2023_35190_MOESM1_ESM.zip › test/images/armas--2542-_jpg.rf.2cb347eb02d7ba30844729f62ea49812.jpg]

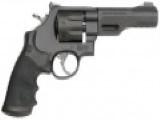

Supplement: Supplementary file 1 — Supplementary Information 1. [file 41598_2023_35190_MOESM1_ESM.zip › test/images/armas--2543-_jpg.rf.df291560aa55f29593bdfaadb9f1262f.jpg]

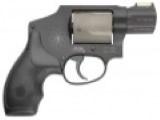

Supplement: Supplementary file 1 — Supplementary Information 1. [file 41598_2023_35190_MOESM1_ESM.zip › test/images/armas--2544-_jpg.rf.c93b036676748ab7d9f93663670a4770.jpg]

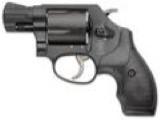

Supplement: Supplementary file 1 — Supplementary Information 1. [file 41598_2023_35190_MOESM1_ESM.zip › test/images/armas--2545-_jpg.rf.4a2cc0acad52b40310802efd50ad1802.jpg]

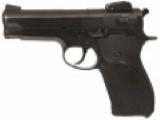

Supplement: Supplementary file 1 — Supplementary Information 1. [file 41598_2023_35190_MOESM1_ESM.zip › test/images/armas--2546-_jpg.rf.a9503d0ebc74cd01b68f89297d1d1ec7.jpg]

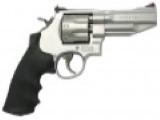

Supplement: Supplementary file 1 — Supplementary Information 1. [file 41598_2023_35190_MOESM1_ESM.zip › test/images/armas--2547-_jpg.rf.bab0f2a4cfa08f3f9ef3a6194b6d67ed.jpg]

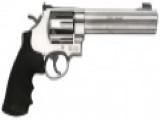

Supplement: Supplementary file 1 — Supplementary Information 1. [file 41598_2023_35190_MOESM1_ESM.zip › test/images/armas--2548-_jpg.rf.cf7fa5a3a5a859a7de540ffcf0fc05c0.jpg]

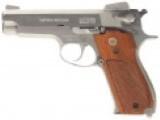

Supplement: Supplementary file 1 — Supplementary Information 1. [file 41598_2023_35190_MOESM1_ESM.zip › test/images/armas--2550-_jpg.rf.62034900efe2b36467d740453cb5f433.jpg]

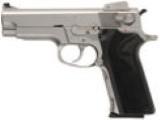

Supplement: Supplementary file 1 — Supplementary Information 1. [file 41598_2023_35190_MOESM1_ESM.zip › test/images/armas--2559-_jpg.rf.e542b33a3cbd1802bdecb04171e2df4a.jpg]

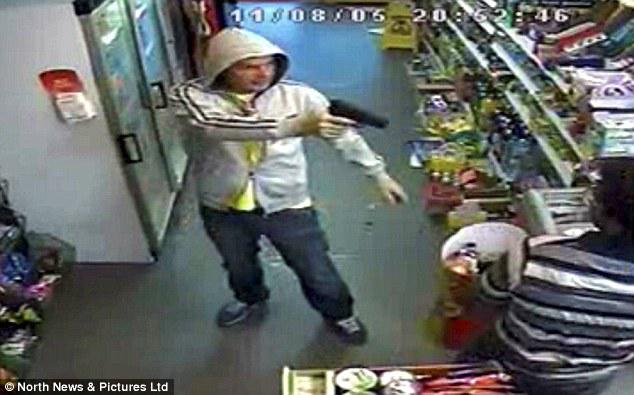

Supplement: Supplementary file 1 — Supplementary Information 1. [file 41598_2023_35190_MOESM1_ESM.zip › test/images/armas--256-_jpg.rf.84e79620c2f70e08eb7ab73cec6db5dd.jpg]

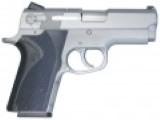

Supplement: Supplementary file 1 — Supplementary Information 1. [file 41598_2023_35190_MOESM1_ESM.zip › test/images/armas--2560-_jpg.rf.735239c32df0e8871600b6537dde1b9d.jpg]

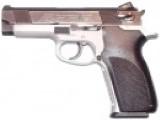

Supplement: Supplementary file 1 — Supplementary Information 1. [file 41598_2023_35190_MOESM1_ESM.zip › test/images/armas--2561-_jpg.rf.3f17b1da4aba8a49437bed99ab12262d.jpg]

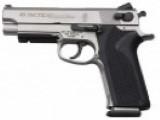

Supplement: Supplementary file 1 — Supplementary Information 1. [file 41598_2023_35190_MOESM1_ESM.zip › test/images/armas--2562-_jpg.rf.df78d57883fd2501c5a47c26826d87ad.jpg]

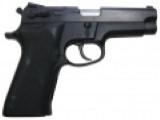

Supplement: Supplementary file 1 — Supplementary Information 1. [file 41598_2023_35190_MOESM1_ESM.zip › test/images/armas--2563-_jpg.rf.a56eede3591f99ed8568c09dad36b0c3.jpg]

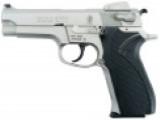

Supplement: Supplementary file 1 — Supplementary Information 1. [file 41598_2023_35190_MOESM1_ESM.zip › test/images/armas--2564-_jpg.rf.6c5637872bdcb5a14b9ffb277c4196ed.jpg]

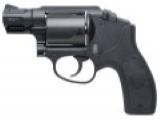

Supplement: Supplementary file 1 — Supplementary Information 1. [file 41598_2023_35190_MOESM1_ESM.zip › test/images/armas--2566-_jpg.rf.ff4c3326a96f063eb2aa46d861c3ced0.jpg]

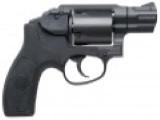

Supplement: Supplementary file 1 — Supplementary Information 1. [file 41598_2023_35190_MOESM1_ESM.zip › test/images/armas--2567-_jpg.rf.b6d20352bd93b5123d84b9c9086423d6.jpg]

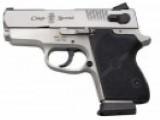

Supplement: Supplementary file 1 — Supplementary Information 1. [file 41598_2023_35190_MOESM1_ESM.zip › test/images/armas--2568-_jpg.rf.00bb789d312a246a0a07e96451050f68.jpg]

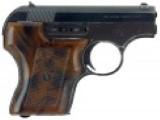

Supplement: Supplementary file 1 — Supplementary Information 1. [file 41598_2023_35190_MOESM1_ESM.zip › test/images/armas--2569-_jpg.rf.60196bf79ae8882d399601c3171f1ffb.jpg]

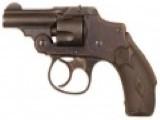

Supplement: Supplementary file 1 — Supplementary Information 1. [file 41598_2023_35190_MOESM1_ESM.zip › test/images/armas--2570-_jpg.rf.a0dcdae7f12eb3a51d3c7eff2a64d859.jpg]

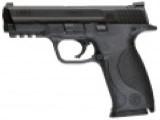

Supplement: Supplementary file 1 — Supplementary Information 1. [file 41598_2023_35190_MOESM1_ESM.zip › test/images/armas--2571-_jpg.rf.499bc44890b9a46a0e80a886657f5f26.jpg]

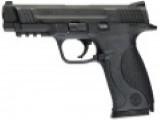

Supplement: Supplementary file 1 — Supplementary Information 1. [file 41598_2023_35190_MOESM1_ESM.zip › test/images/armas--2572-_jpg.rf.855882bf5e9d0ac8a8e1a04d2aeb87e4.jpg]

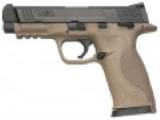

Supplement: Supplementary file 1 — Supplementary Information 1. [file 41598_2023_35190_MOESM1_ESM.zip › test/images/armas--2573-_jpg.rf.edc55b05b731a919c33cb35f1b612a31.jpg]

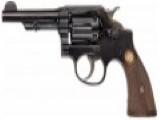

Supplement: Supplementary file 1 — Supplementary Information 1. [file 41598_2023_35190_MOESM1_ESM.zip › test/images/armas--2574-_jpg.rf.2f26004325f9e9977c12b19d383b666a.jpg]

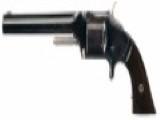

Supplement: Supplementary file 1 — Supplementary Information 1. [file 41598_2023_35190_MOESM1_ESM.zip › test/images/armas--2575-_jpg.rf.59e6ef7ee5ff14fc9392f288768cefbb.jpg]

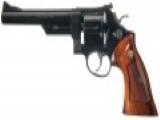

Supplement: Supplementary file 1 — Supplementary Information 1. [file 41598_2023_35190_MOESM1_ESM.zip › test/images/armas--2576-_jpg.rf.138f9774570597c7fec99e0a88ae4be1.jpg]

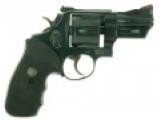

Supplement: Supplementary file 1 — Supplementary Information 1. [file 41598_2023_35190_MOESM1_ESM.zip › test/images/armas--2577-_jpg.rf.e50f76e90fcc7d505eb4f0c347668737.jpg]

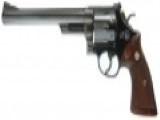

Supplement: Supplementary file 1 — Supplementary Information 1. [file 41598_2023_35190_MOESM1_ESM.zip › test/images/armas--2578-_jpg.rf.9f492de27826822c9fd483ccea5c822e.jpg]

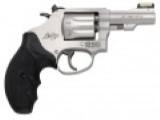

Supplement: Supplementary file 1 — Supplementary Information 1. [file 41598_2023_35190_MOESM1_ESM.zip › test/images/armas--2579-_jpg.rf.2d67e3ce93a7c779f015bb71801d1d9c.jpg]

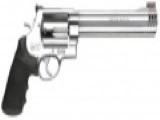

Supplement: Supplementary file 1 — Supplementary Information 1. [file 41598_2023_35190_MOESM1_ESM.zip › test/images/armas--2580-_jpg.rf.fd9668cd139ed6f935a751bbef7f92c9.jpg]

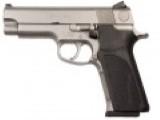

Supplement: Supplementary file 1 — Supplementary Information 1. [file 41598_2023_35190_MOESM1_ESM.zip › test/images/armas--2582-_jpg.rf.66eacabcb67aaff320738ac220f8f783.jpg]

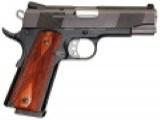

Supplement: Supplementary file 1 — Supplementary Information 1. [file 41598_2023_35190_MOESM1_ESM.zip › test/images/armas--2583-_jpg.rf.2f1892727efbc8a626f59f9f3c4d88d5.jpg]

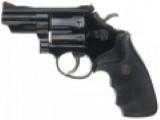

Supplement: Supplementary file 1 — Supplementary Information 1. [file 41598_2023_35190_MOESM1_ESM.zip › test/images/armas--2584-_jpg.rf.9de07be892630c0798d1fa8a7b905d03.jpg]

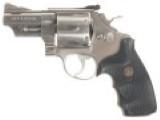

Supplement: Supplementary file 1 — Supplementary Information 1. [file 41598_2023_35190_MOESM1_ESM.zip › test/images/armas--2585-_jpg.rf.33d283e4a07a76d7de1d28024326e2a9.jpg]

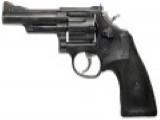

Supplement: Supplementary file 1 — Supplementary Information 1. [file 41598_2023_35190_MOESM1_ESM.zip › test/images/armas--2586-_jpg.rf.f45e5d36ba2f70eee5e2c5e2434161e4.jpg]

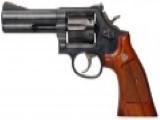

Supplement: Supplementary file 1 — Supplementary Information 1. [file 41598_2023_35190_MOESM1_ESM.zip › test/images/armas--2587-_jpg.rf.5e64b20d8ac6479150628e2ea5ef49a9.jpg]

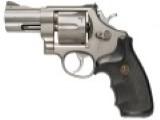

Supplement: Supplementary file 1 — Supplementary Information 1. [file 41598_2023_35190_MOESM1_ESM.zip › test/images/armas--2588-_jpg.rf.14b223af353372a30ef8fc01b4db7f83.jpg]

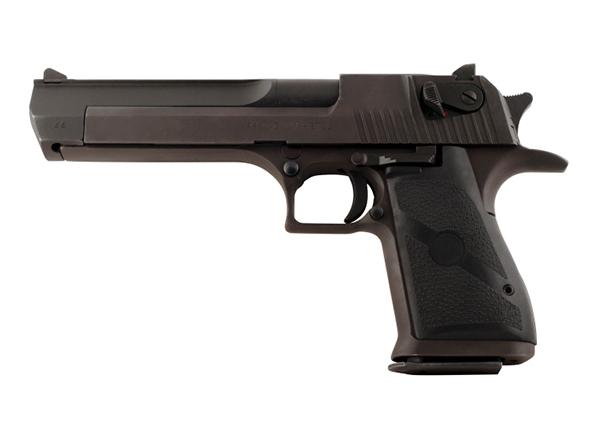

Supplement: Supplementary file 1 — Supplementary Information 1. [file 41598_2023_35190_MOESM1_ESM.zip › test/images/armas--2847-_jpg.rf.76f26c2ec5fbff98b75c61b000372833.jpg]

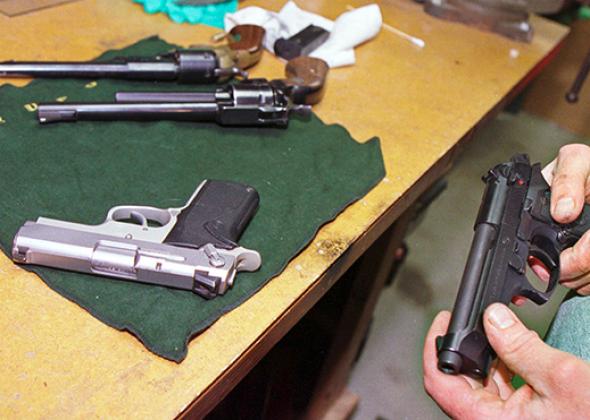

Supplement: Supplementary file 1 — Supplementary Information 1. [file 41598_2023_35190_MOESM1_ESM.zip › test/images/armas--2848-_jpg.rf.88e2dca4cb3286258849110f8424d882.jpg]

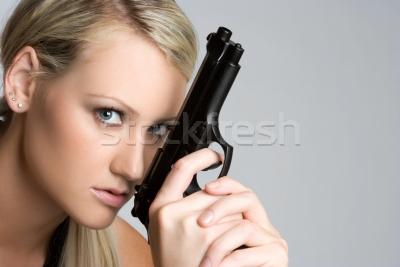

Supplement: Supplementary file 1 — Supplementary Information 1. [file 41598_2023_35190_MOESM1_ESM.zip › test/images/armas--2853-_jpg.rf.0c949be9cfb151aa4dc6ec61921cbad9.jpg]

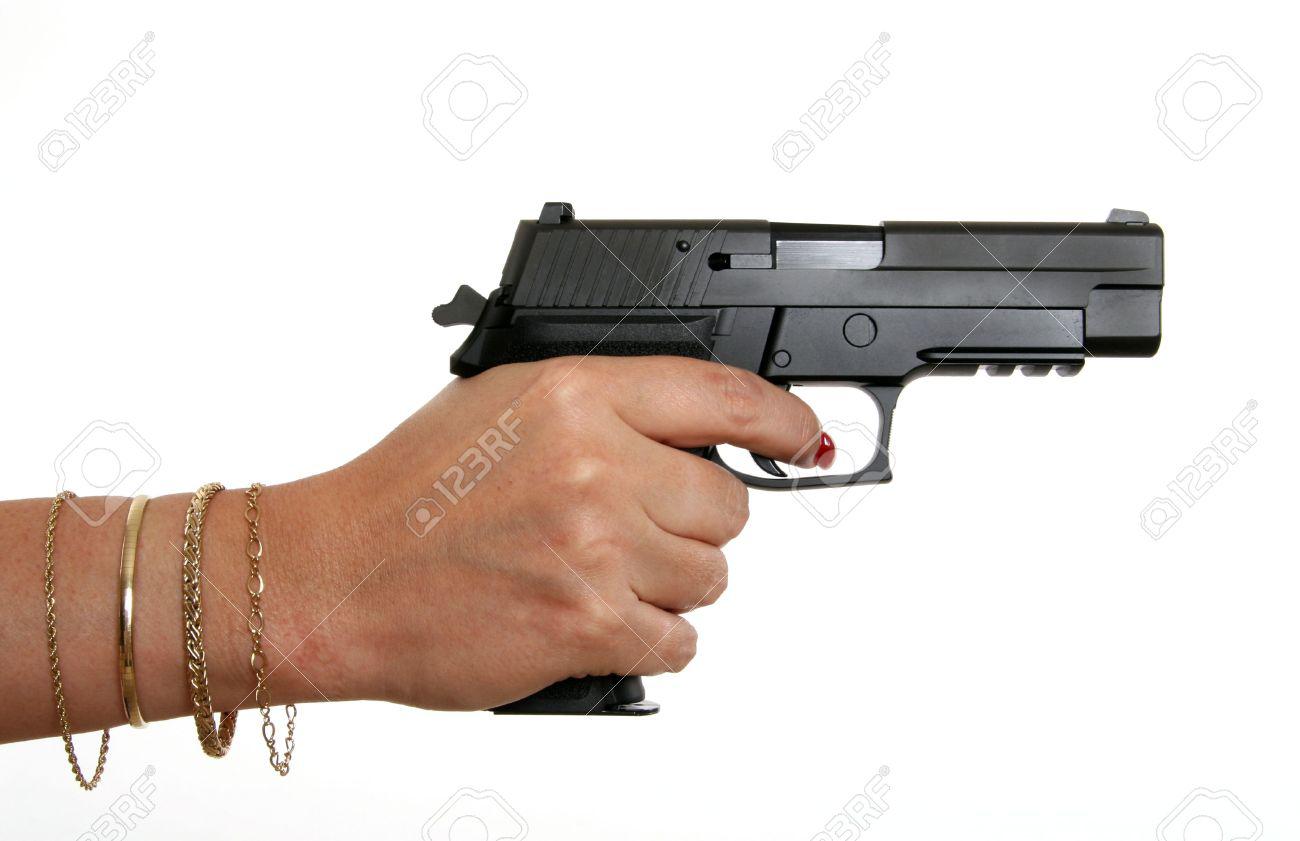

Supplement: Supplementary file 1 — Supplementary Information 1. [file 41598_2023_35190_MOESM1_ESM.zip › test/images/armas--2857-_jpg.rf.457870da9e04eb4a4578917bfbf9034c.jpg]

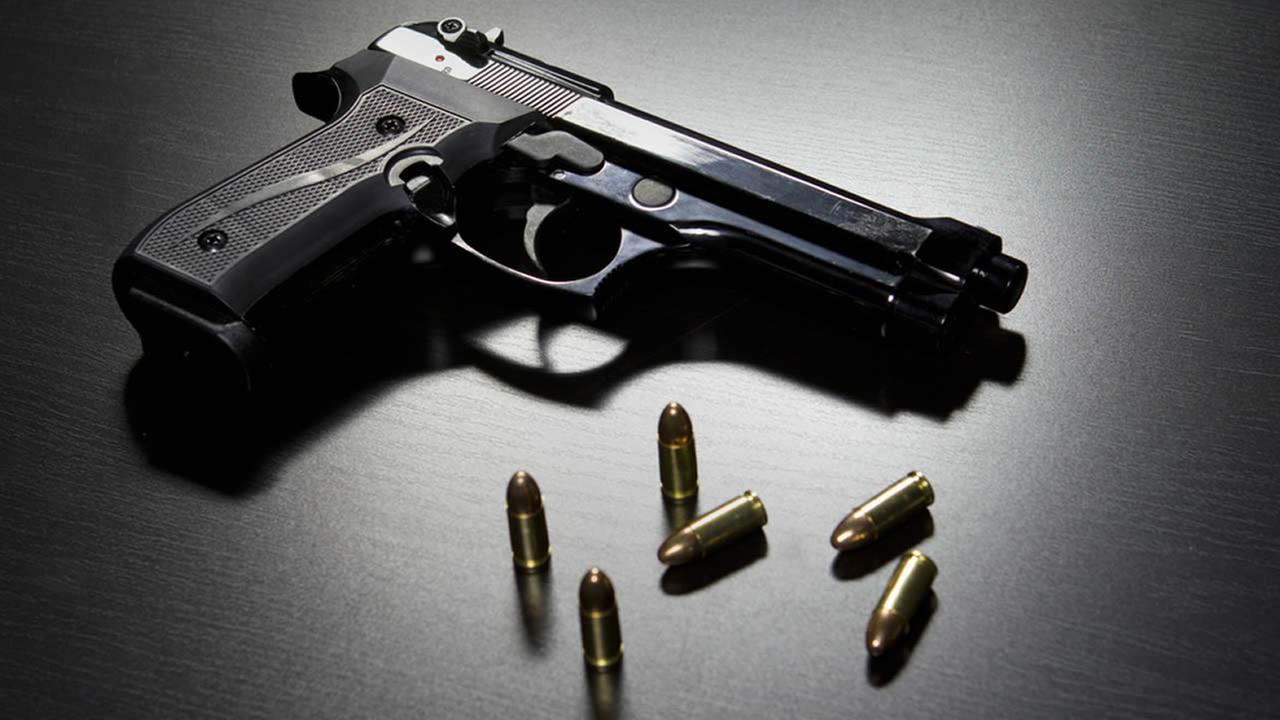

Supplement: Supplementary file 1 — Supplementary Information 1. [file 41598_2023_35190_MOESM1_ESM.zip › test/images/armas--2859-_jpg.rf.4e4c90b1f5eaed40af4846f67796cc02.jpg]

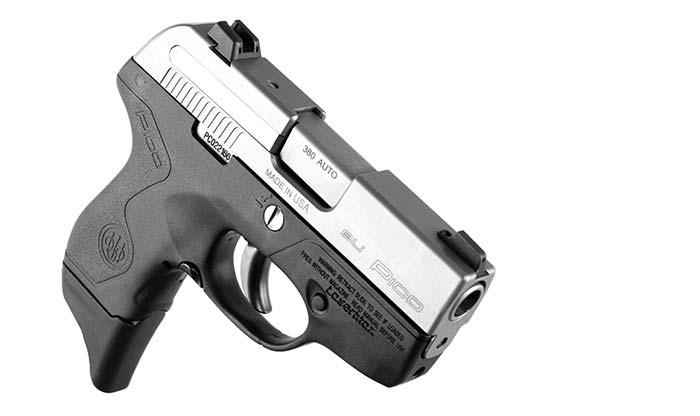

Supplement: Supplementary file 1 — Supplementary Information 1. [file 41598_2023_35190_MOESM1_ESM.zip › test/images/armas--286-_jpg.rf.e6eeaed6211ec3ae776956a07ab6fde9.jpg]

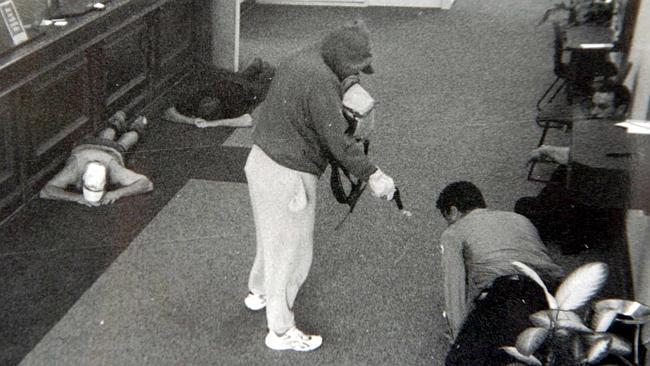

Supplement: Supplementary file 1 — Supplementary Information 1. [file 41598_2023_35190_MOESM1_ESM.zip › test/images/armas--2862-_jpg.rf.4d1bf50cb6d1a99987f8318581407607.jpg]

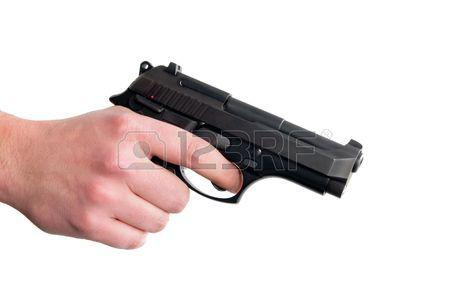

Supplement: Supplementary file 1 — Supplementary Information 1. [file 41598_2023_35190_MOESM1_ESM.zip › test/images/armas--2863-_jpg.rf.3f50c1cc6e7d72713db50977a7337704.jpg]

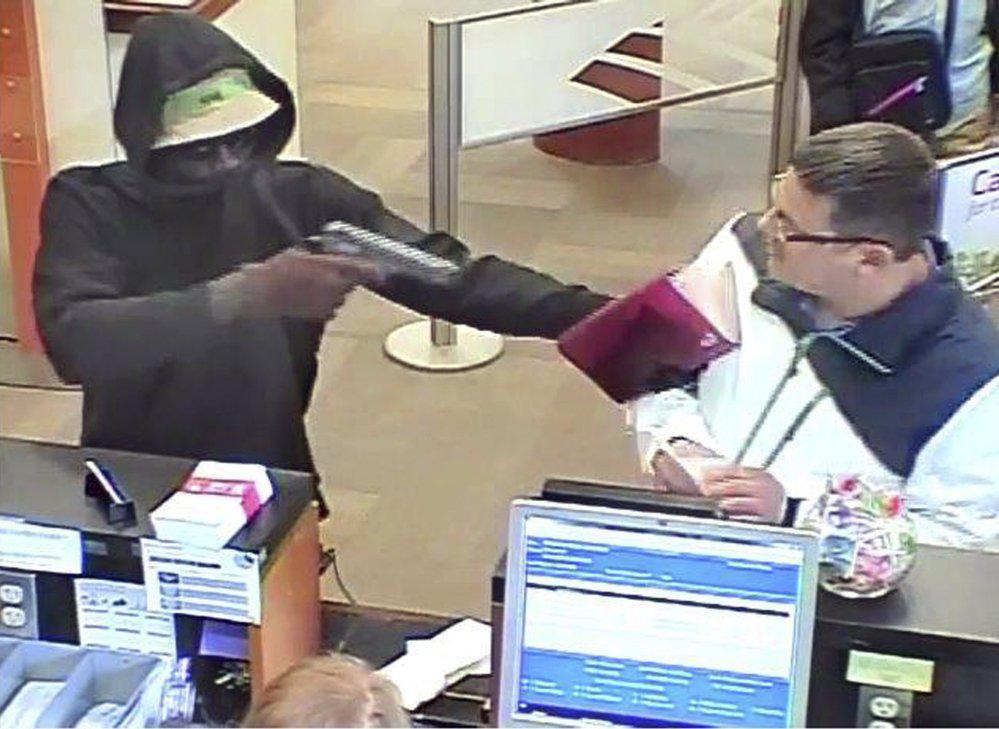

Supplement: Supplementary file 1 — Supplementary Information 1. [file 41598_2023_35190_MOESM1_ESM.zip › test/images/armas--2864-_jpg.rf.5d8a8fa10dd078fce5fc9483420329de.jpg]

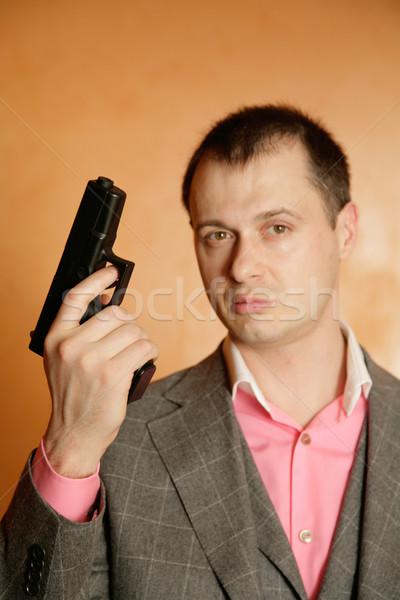

Supplement: Supplementary file 1 — Supplementary Information 1. [file 41598_2023_35190_MOESM1_ESM.zip › test/images/armas--2866-_jpg.rf.29132a465f58956738c7c2b0c7255f62.jpg]

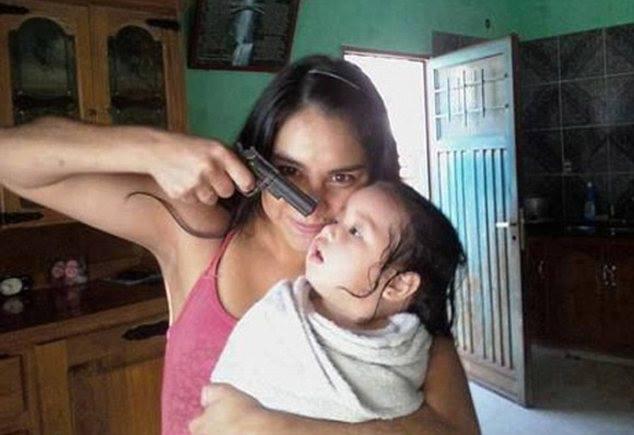

Supplement: Supplementary file 1 — Supplementary Information 1. [file 41598_2023_35190_MOESM1_ESM.zip › test/images/armas--2869-_jpg.rf.e9105d050a89bdd517f78d55d703a31a.jpg]

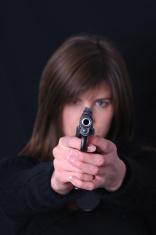

Supplement: Supplementary file 1 — Supplementary Information 1. [file 41598_2023_35190_MOESM1_ESM.zip › test/images/armas--2871-_jpg.rf.94c33f3898a7c702028ff0eee27b8b31.jpg]

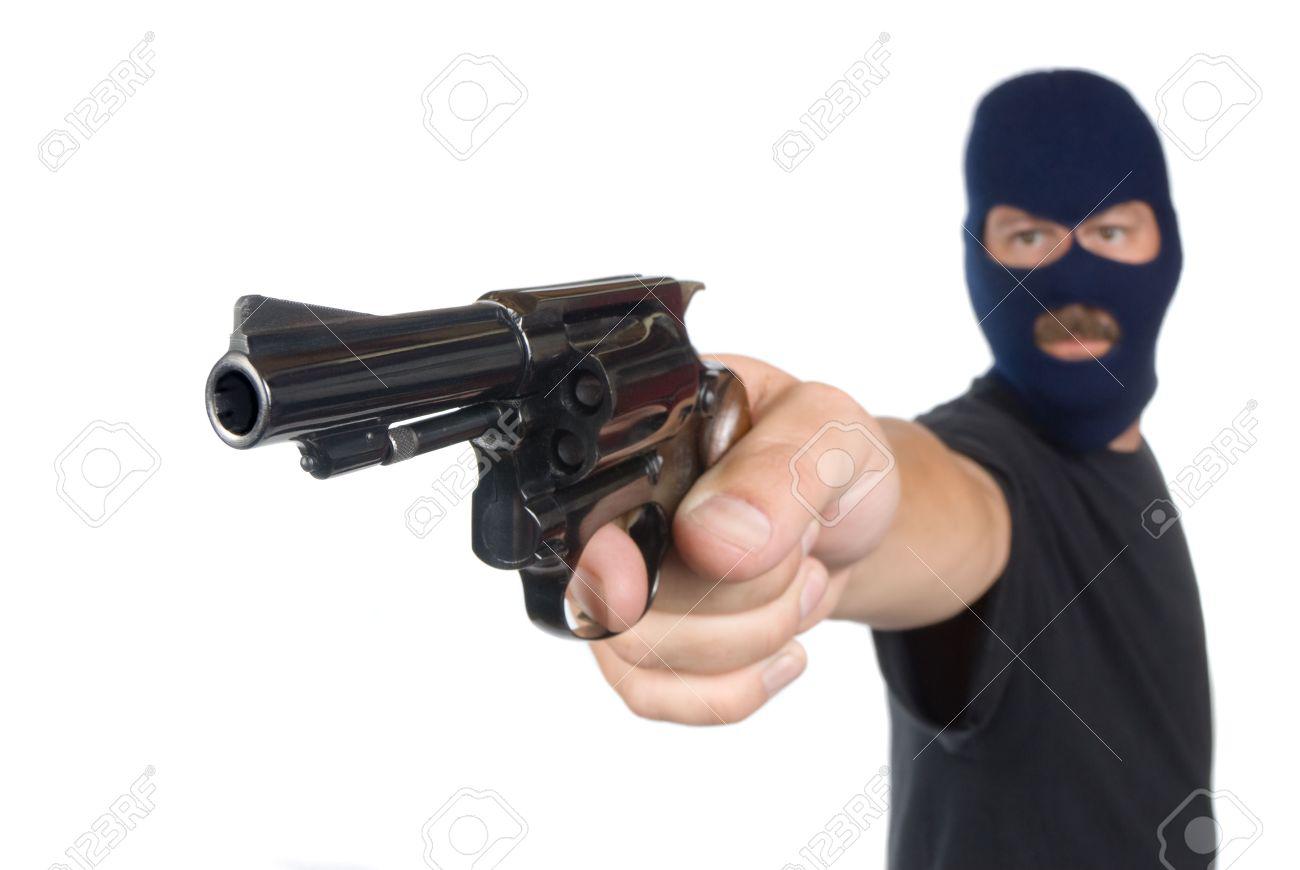

Supplement: Supplementary file 1 — Supplementary Information 1. [file 41598_2023_35190_MOESM1_ESM.zip › test/images/armas--2878-_jpg.rf.462a334ca914333c385ff36872bed3d2.jpg]

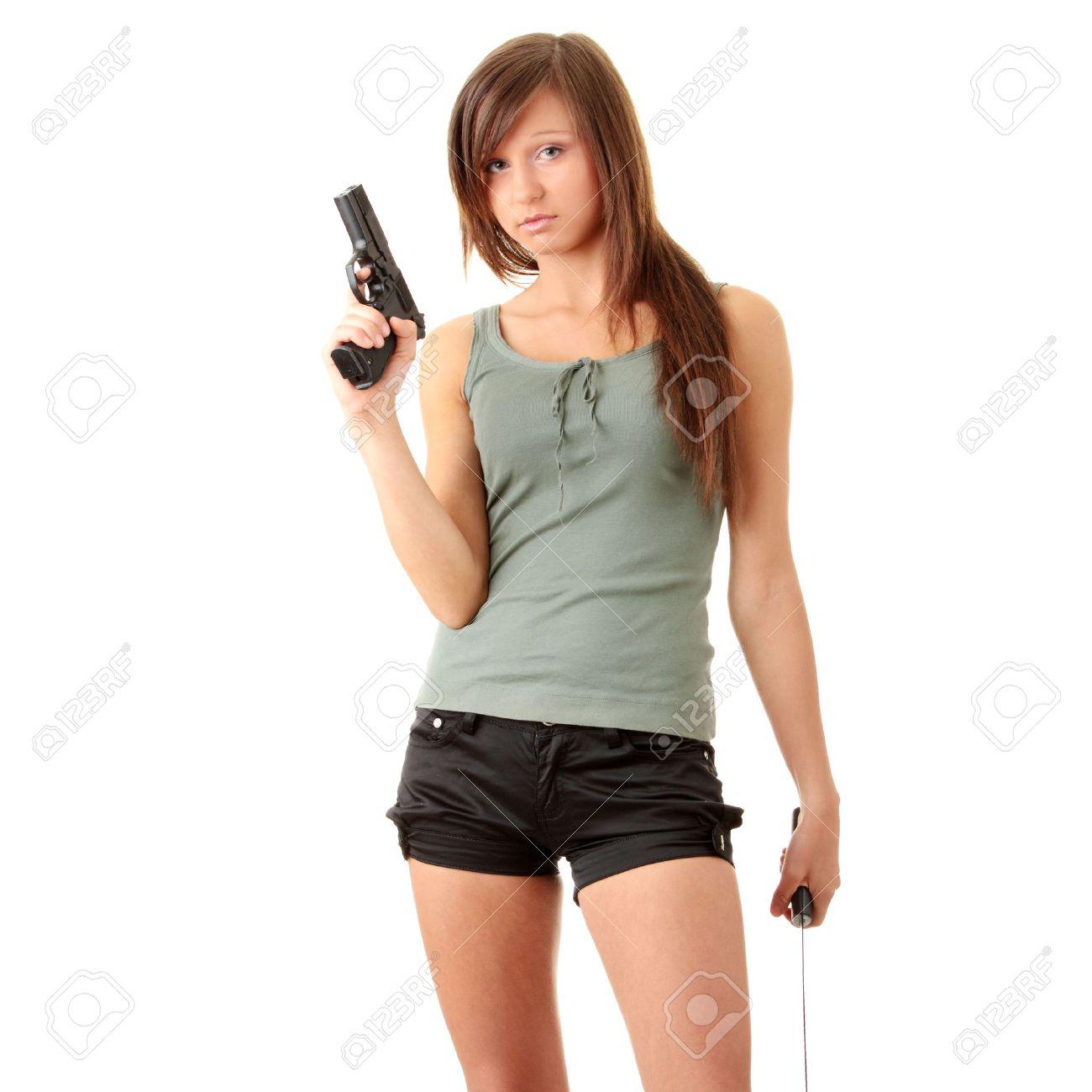

Supplement: Supplementary file 1 — Supplementary Information 1. [file 41598_2023_35190_MOESM1_ESM.zip › test/images/armas--2882-_jpg.rf.a3703e08140ec2979376301ae634602b.jpg]

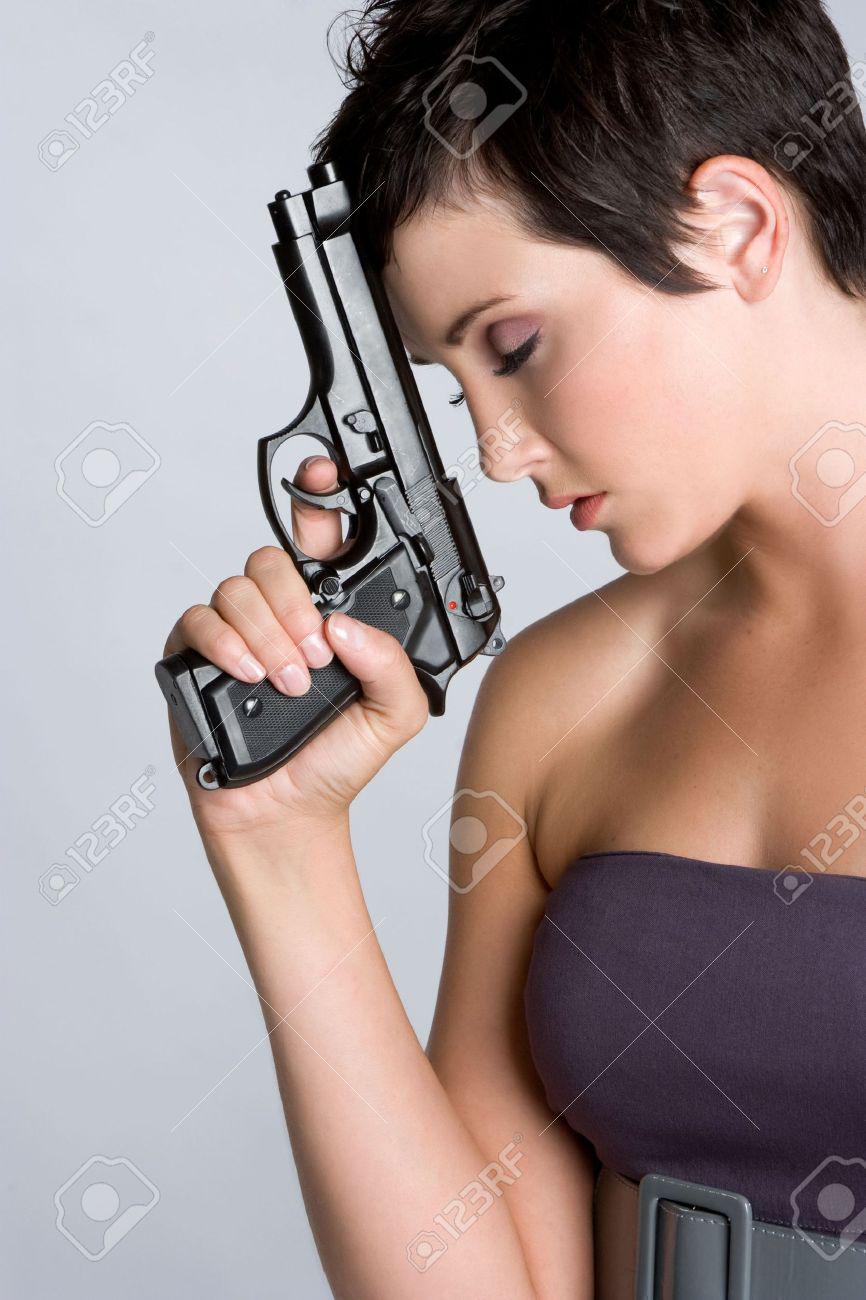

Supplement: Supplementary file 1 — Supplementary Information 1. [file 41598_2023_35190_MOESM1_ESM.zip › test/images/armas--2884-_jpg.rf.f7f3a1dc99e5d9d376eb0ebc8bb95b2c.jpg]

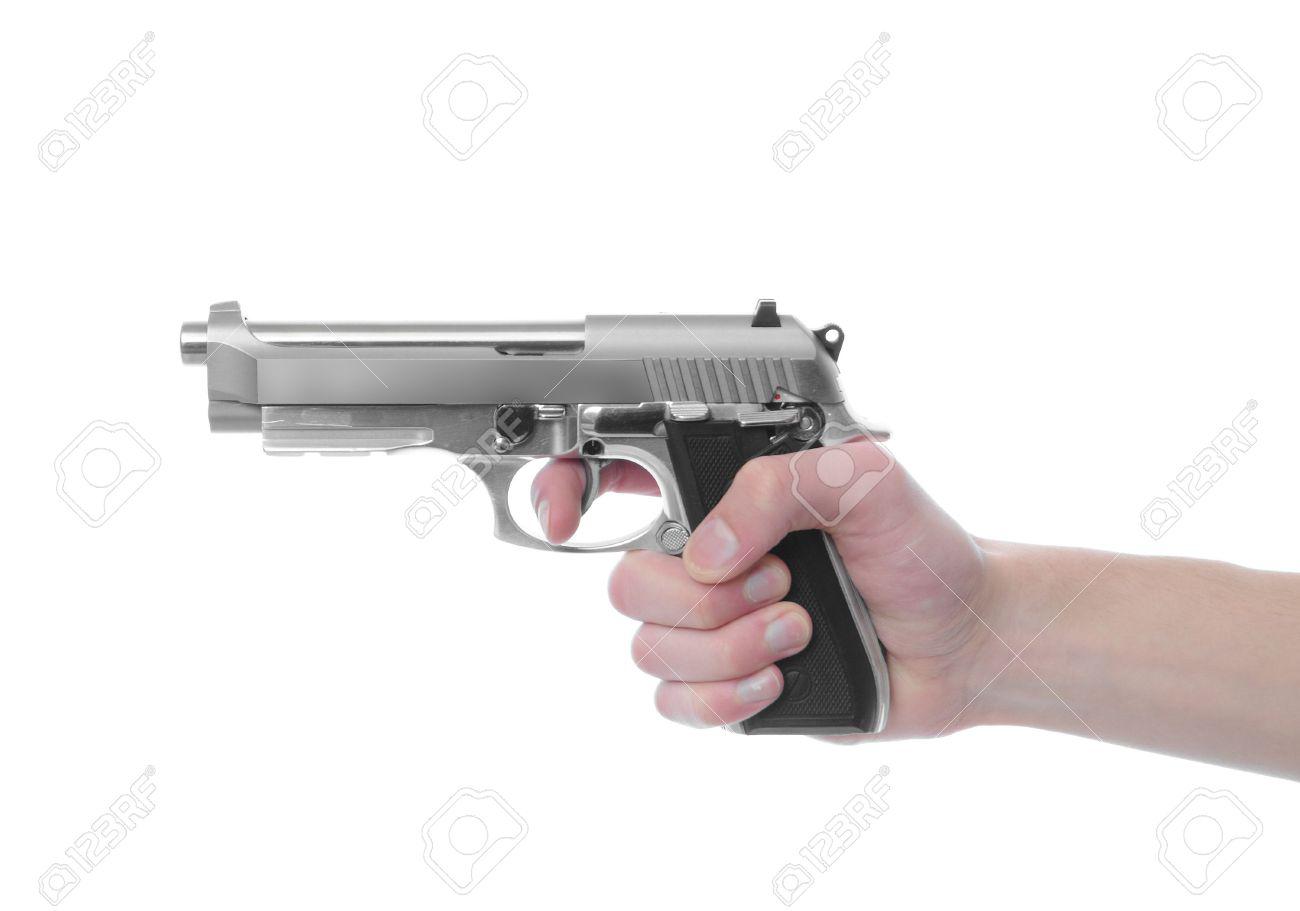

Supplement: Supplementary file 1 — Supplementary Information 1. [file 41598_2023_35190_MOESM1_ESM.zip › test/images/armas--2889-_jpg.rf.9972a94a7377c8153d37a12e04c575c0.jpg]

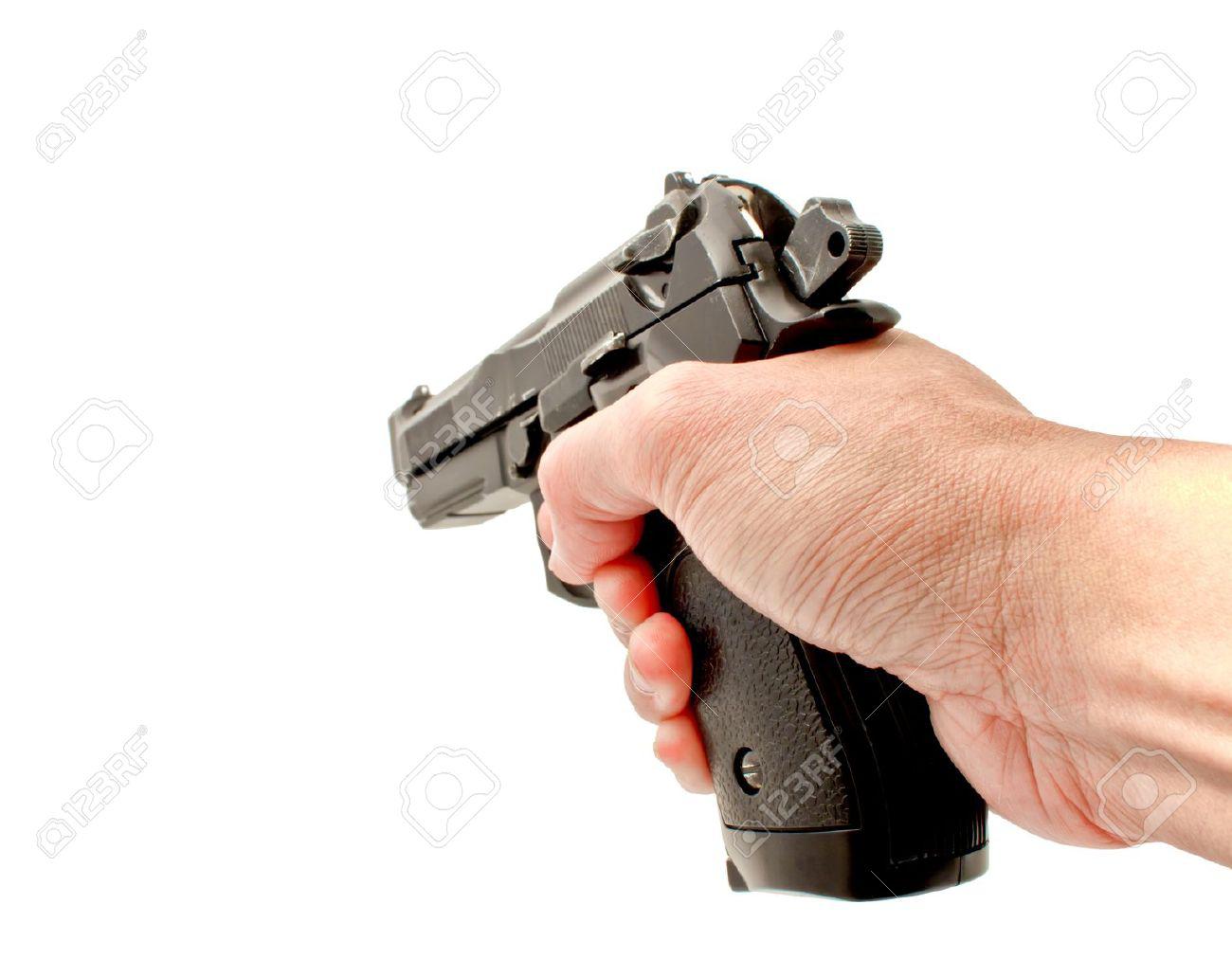

Supplement: Supplementary file 1 — Supplementary Information 1. [file 41598_2023_35190_MOESM1_ESM.zip › test/images/armas--2933-_jpg.rf.456dc4535d6b12e4dfa0cd05555fbb3e.jpg]

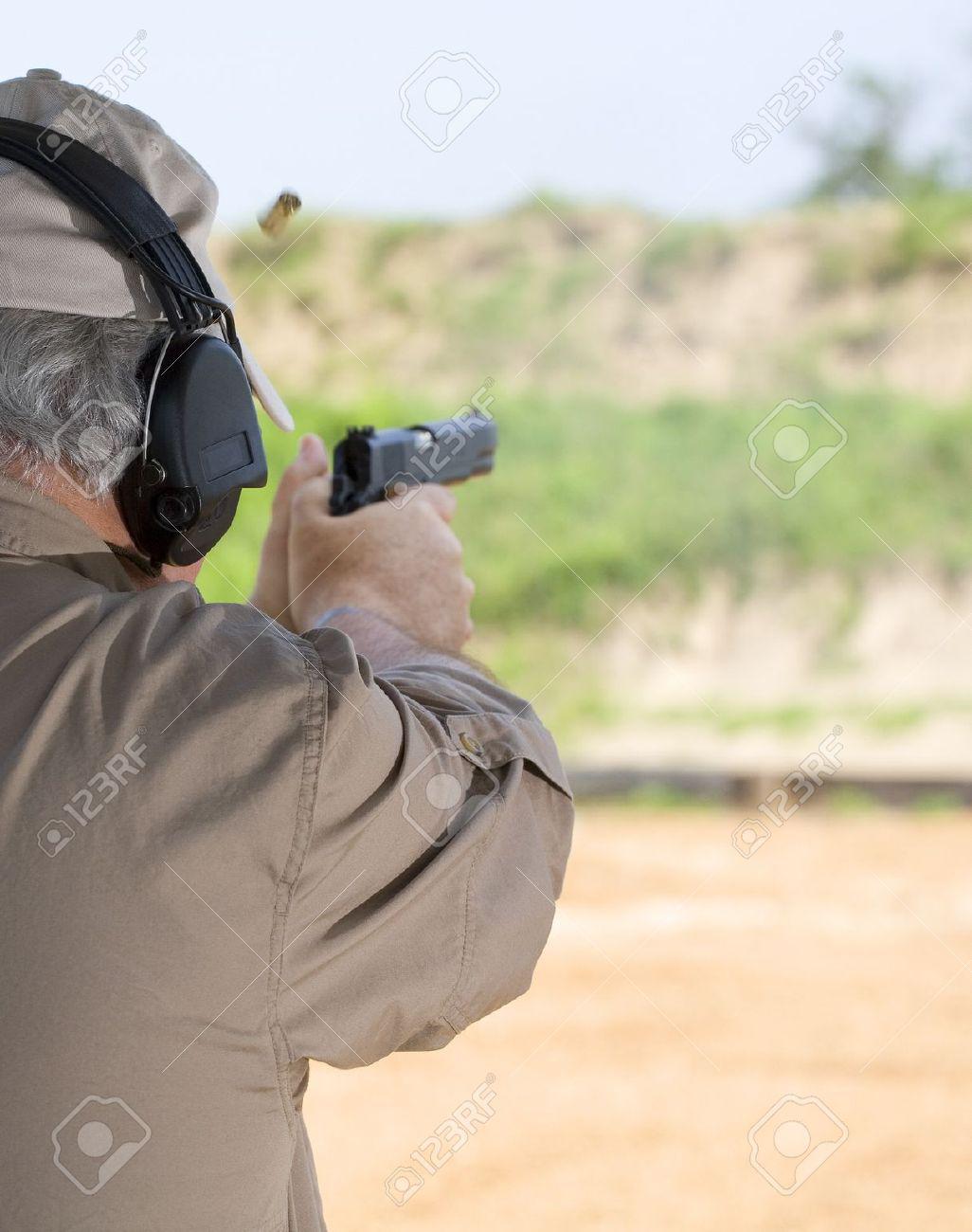

Supplement: Supplementary file 1 — Supplementary Information 1. [file 41598_2023_35190_MOESM1_ESM.zip › test/images/armas--2939-_jpg.rf.39307272d4c876dd1260333921d6584d.jpg]

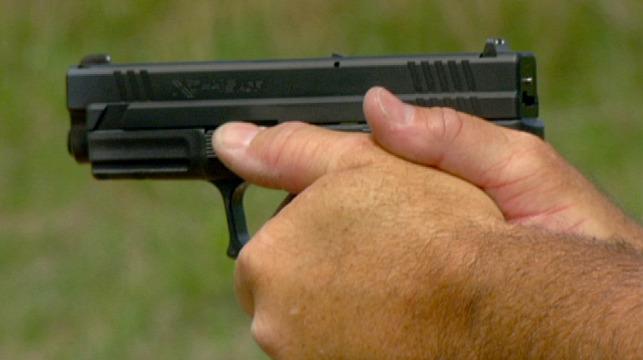

Supplement: Supplementary file 1 — Supplementary Information 1. [file 41598_2023_35190_MOESM1_ESM.zip › test/images/armas--2941-_jpg.rf.c0cb832b0339160e8c85fd4271ed1624.jpg]

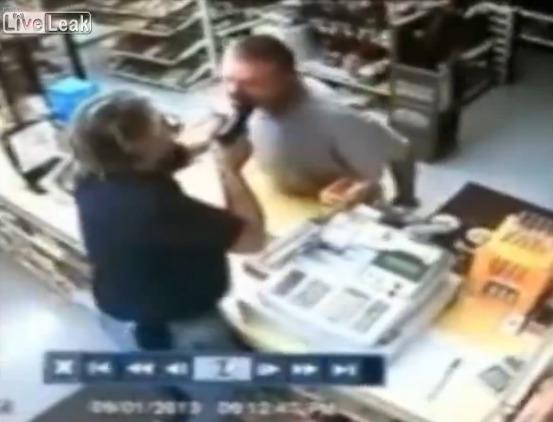

Supplement: Supplementary file 1 — Supplementary Information 1. [file 41598_2023_35190_MOESM1_ESM.zip › test/images/armas--2942-_jpg.rf.875ea538d60de49422b3ce1972f01d13.jpg]

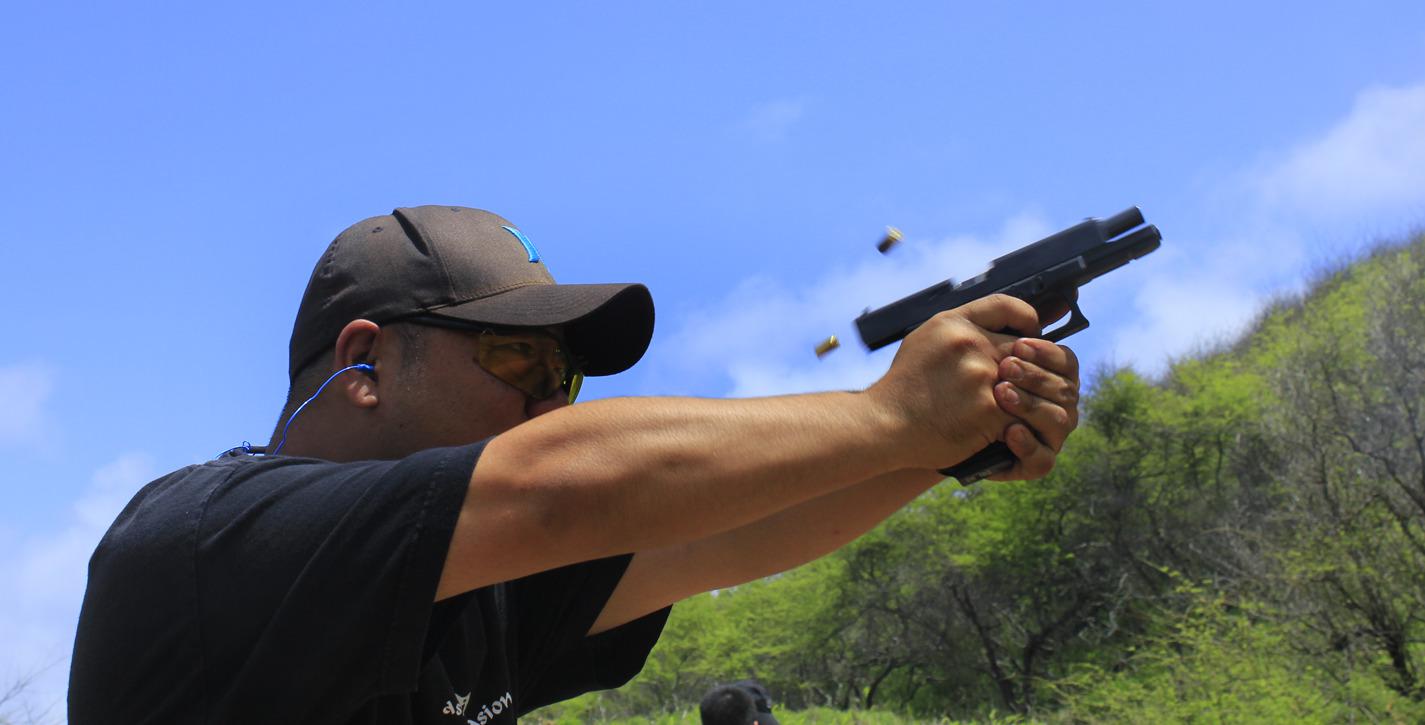

Supplement: Supplementary file 1 — Supplementary Information 1. [file 41598_2023_35190_MOESM1_ESM.zip › test/images/armas--2962-_jpg.rf.9c25aaea34ae763fa8ad40c1d390a0e2.jpg]

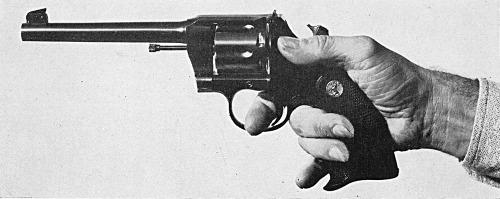

Supplement: Supplementary file 1 — Supplementary Information 1. [file 41598_2023_35190_MOESM1_ESM.zip › test/images/armas--2965-_jpg.rf.b5a13f1f6af8d029488f241e1ca7bdee.jpg]

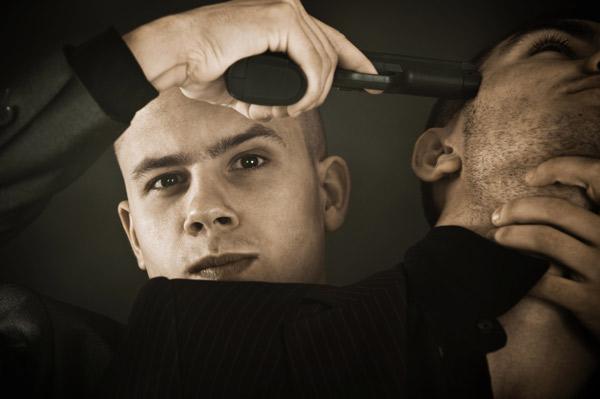

Supplement: Supplementary file 1 — Supplementary Information 1. [file 41598_2023_35190_MOESM1_ESM.zip › test/images/armas--297-_jpg.rf.12f8acb66f4b60bfbd69f799b34dd6c4.jpg]

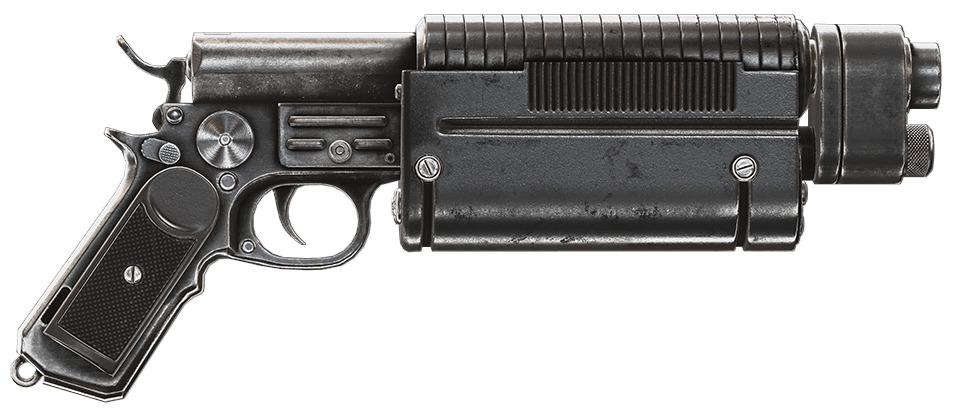

Supplement: Supplementary file 1 — Supplementary Information 1. [file 41598_2023_35190_MOESM1_ESM.zip › test/images/armas--2971-_jpg.rf.209ad71421bf2fbdd5b07607bf35cde7.jpg]

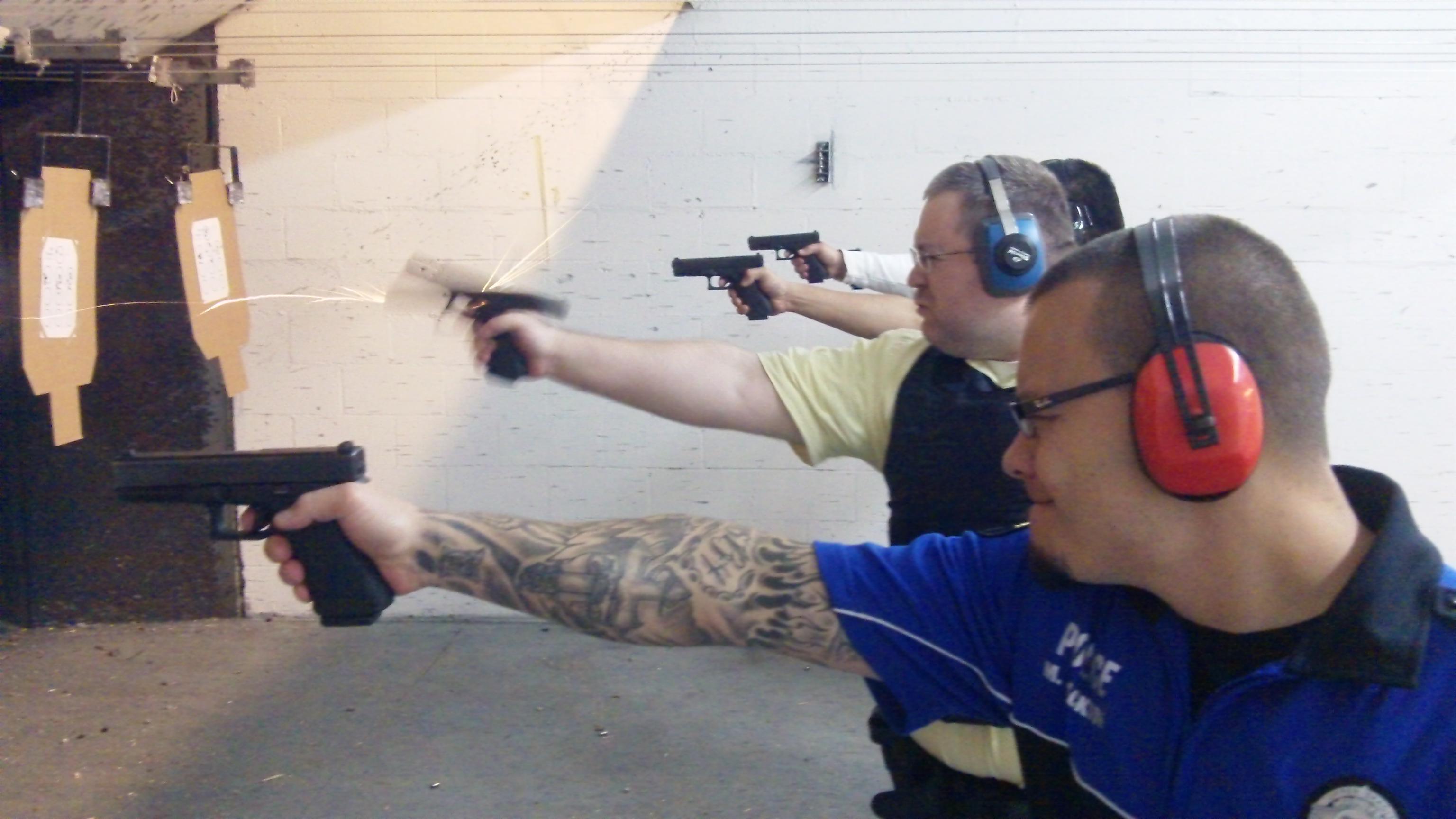

Supplement: Supplementary file 1 — Supplementary Information 1. [file 41598_2023_35190_MOESM1_ESM.zip › test/images/armas--298-_jpg.rf.7ce0b82f461f49e32f33b7211d6865a0.jpg]

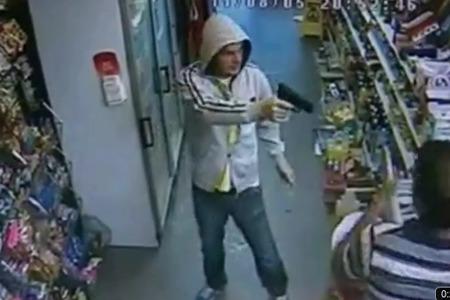

Supplement: Supplementary file 1 — Supplementary Information 1. [file 41598_2023_35190_MOESM1_ESM.zip › test/images/armas--2992-_jpg.rf.e802d654374649af86860b31b6fa5e0a.jpg]

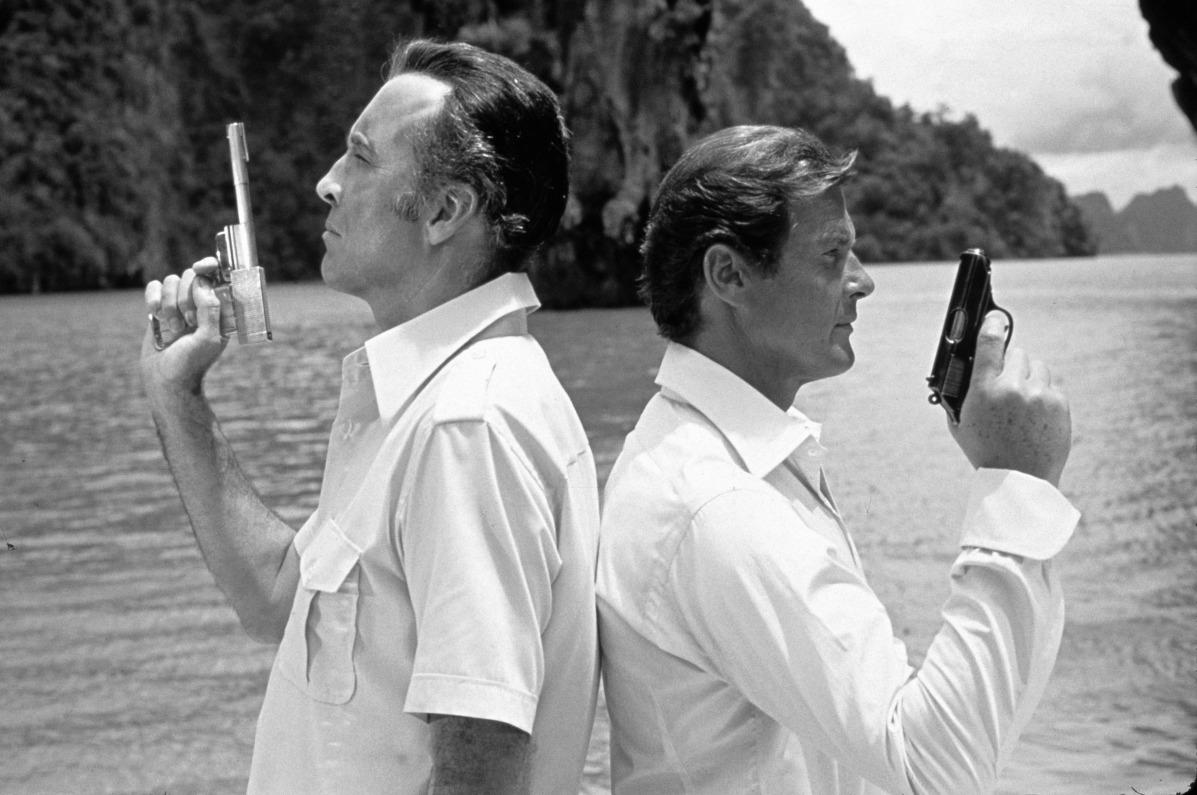

Supplement: Supplementary file 1 — Supplementary Information 1. [file 41598_2023_35190_MOESM1_ESM.zip › test/images/armas--2993-_jpg.rf.332016b66c7a9396cfa8fb6117c57032.jpg]
