# Supplementary figures and images for: Improving video surveillance systems in banks using deep learning techniques (part 4 of 4)
Source: Sci Rep. 2023 May 16;13:7911. doi: 10.1038/s41598-023-35190-9 (PMC10188611; doi:10.1038/s41598-023-35190-9)

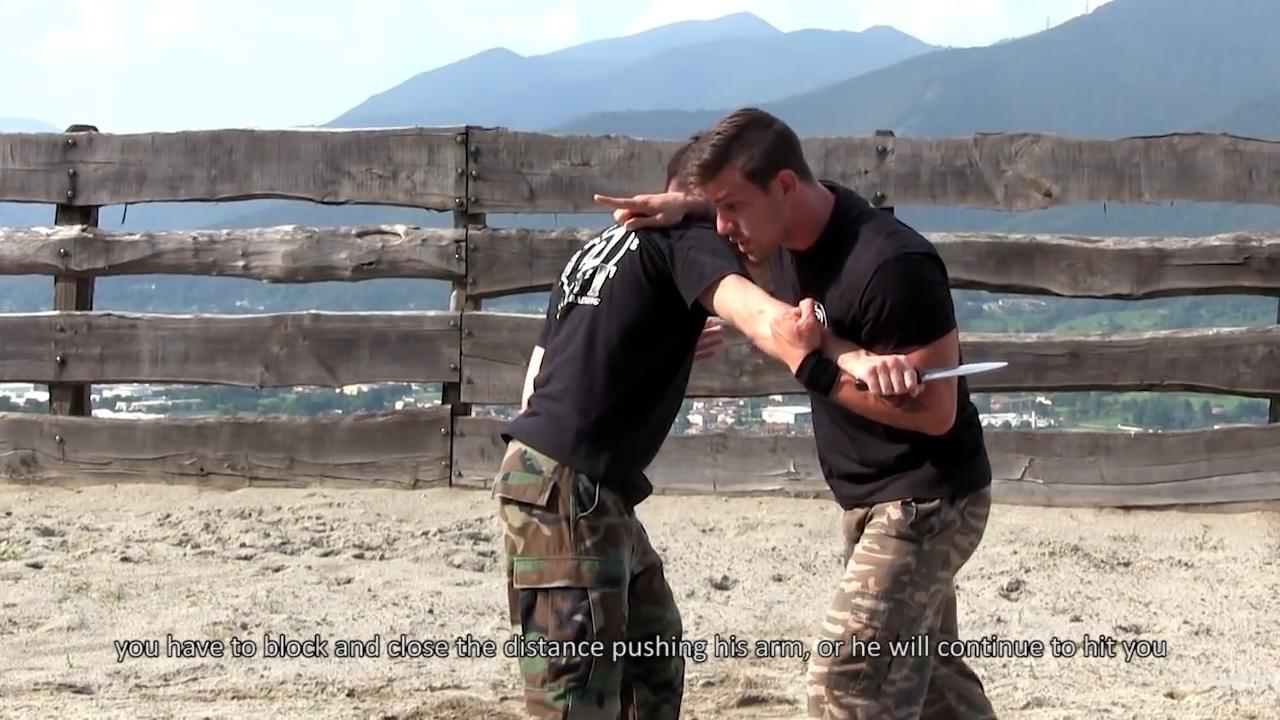

Supplement: Supplementary file 2 — Supplementary Information 2. [file 41598_2023_35190_MOESM2_ESM.zip › test/images/KravMagaTraining20357_jpg.rf.2d07d6eb790fdc24f09aa47540f71cf5.jpg]

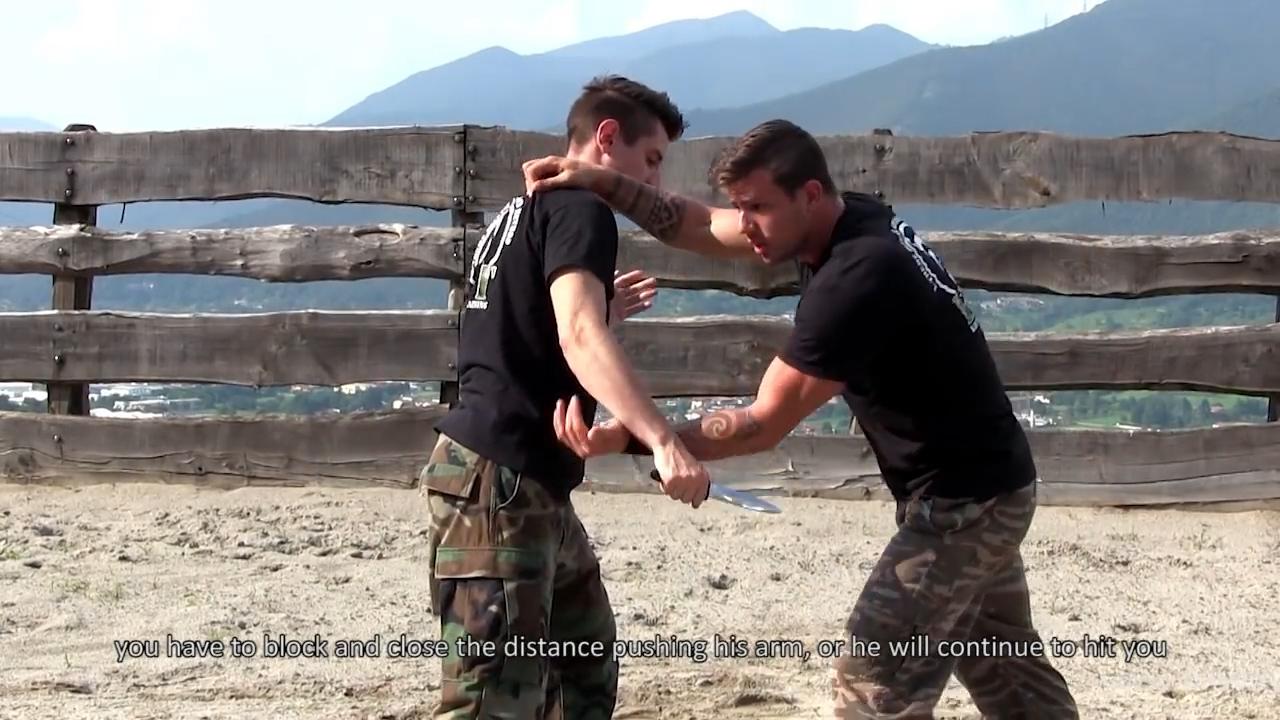

Supplement: Supplementary file 2 — Supplementary Information 2. [file 41598_2023_35190_MOESM2_ESM.zip › test/images/KravMagaTraining20389_jpg.rf.dc26bc497142fa02d21b6263aabf78ba.jpg]

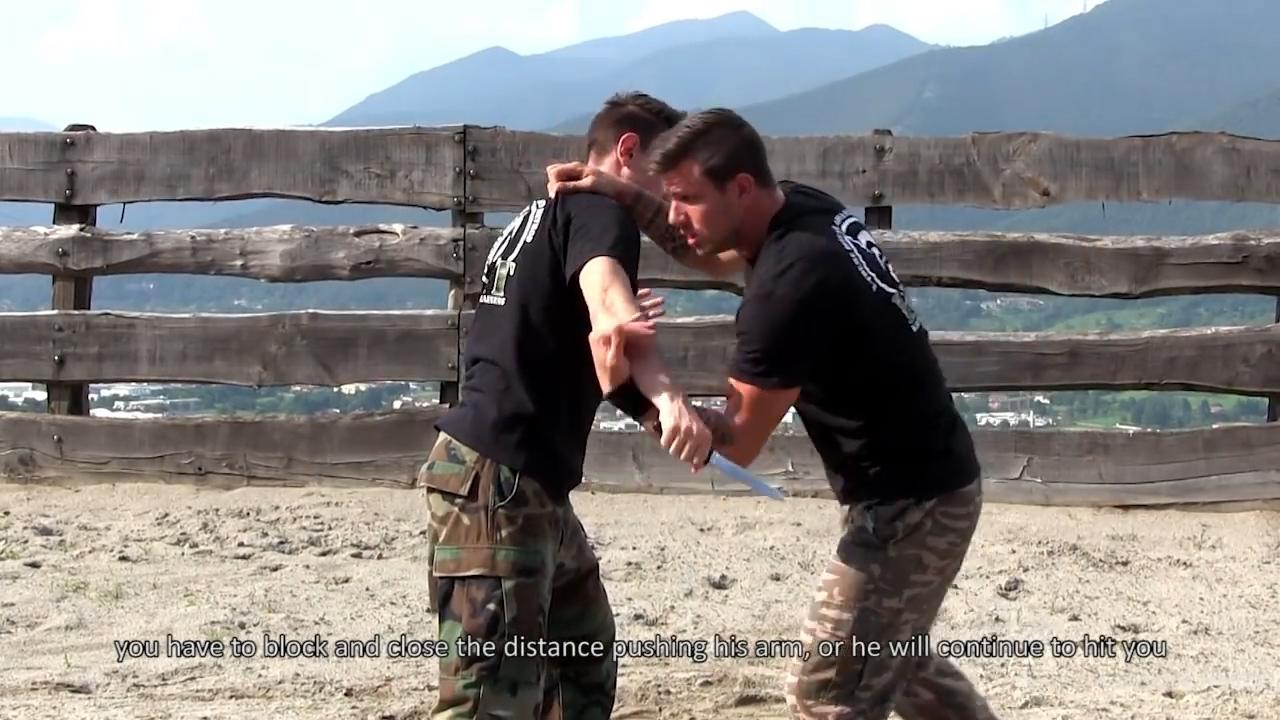

Supplement: Supplementary file 2 — Supplementary Information 2. [file 41598_2023_35190_MOESM2_ESM.zip › test/images/KravMagaTraining20390_jpg.rf.e079301bdba807bb25913d45e3fa73cf.jpg]

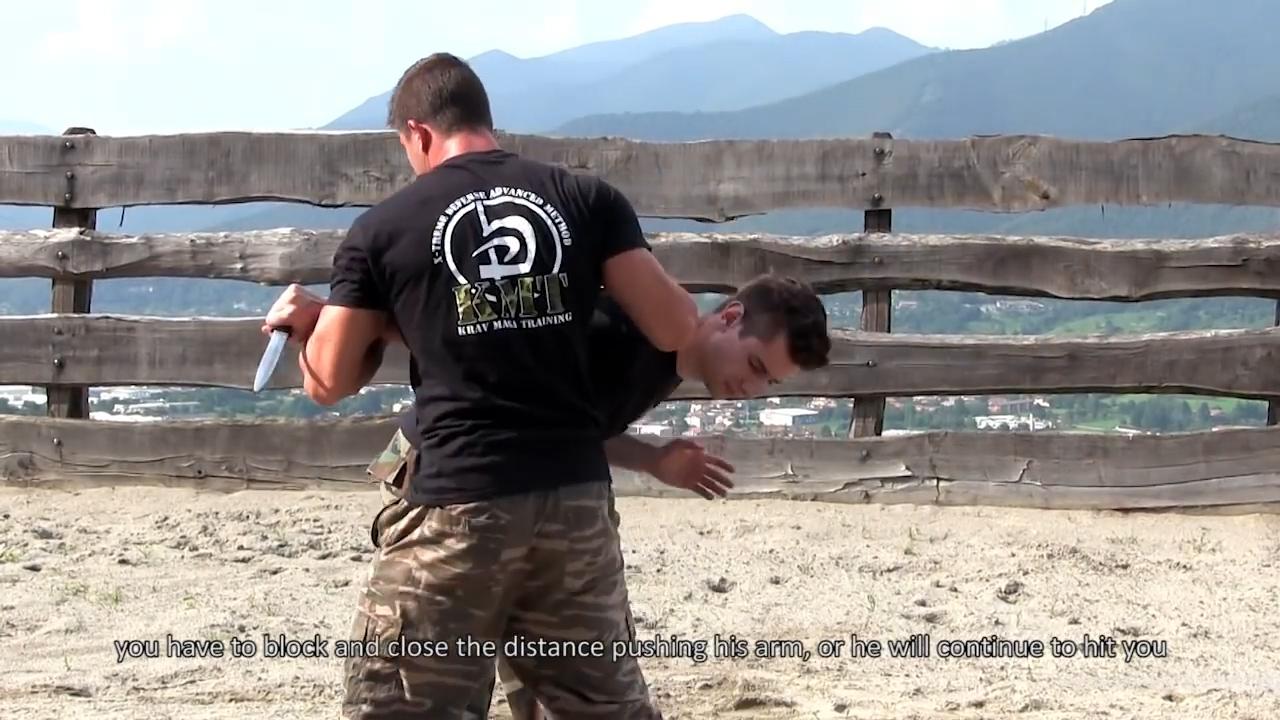

Supplement: Supplementary file 2 — Supplementary Information 2. [file 41598_2023_35190_MOESM2_ESM.zip › test/images/KravMagaTraining20399_jpg.rf.b8a4c15398fec4ec3a219a16d63a5b23.jpg]

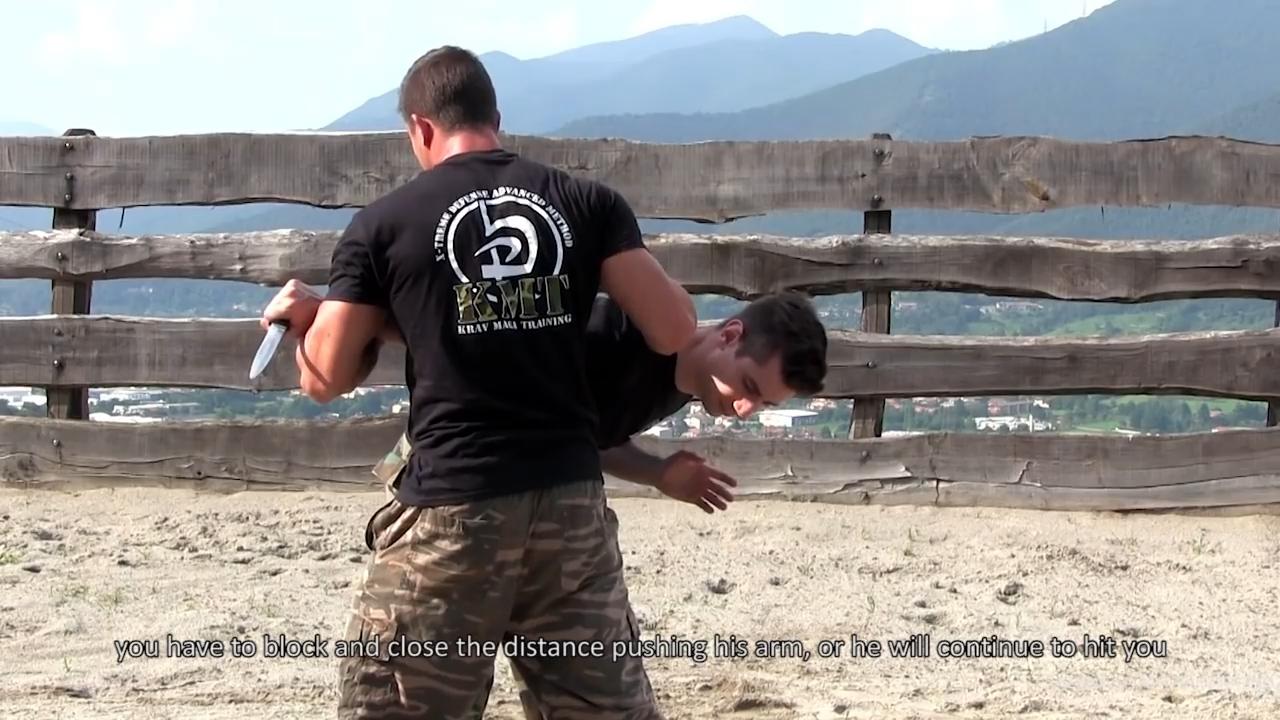

Supplement: Supplementary file 2 — Supplementary Information 2. [file 41598_2023_35190_MOESM2_ESM.zip › test/images/KravMagaTraining20402_jpg.rf.5b359af9bf6b91da599aa9a94b8fbb18.jpg]

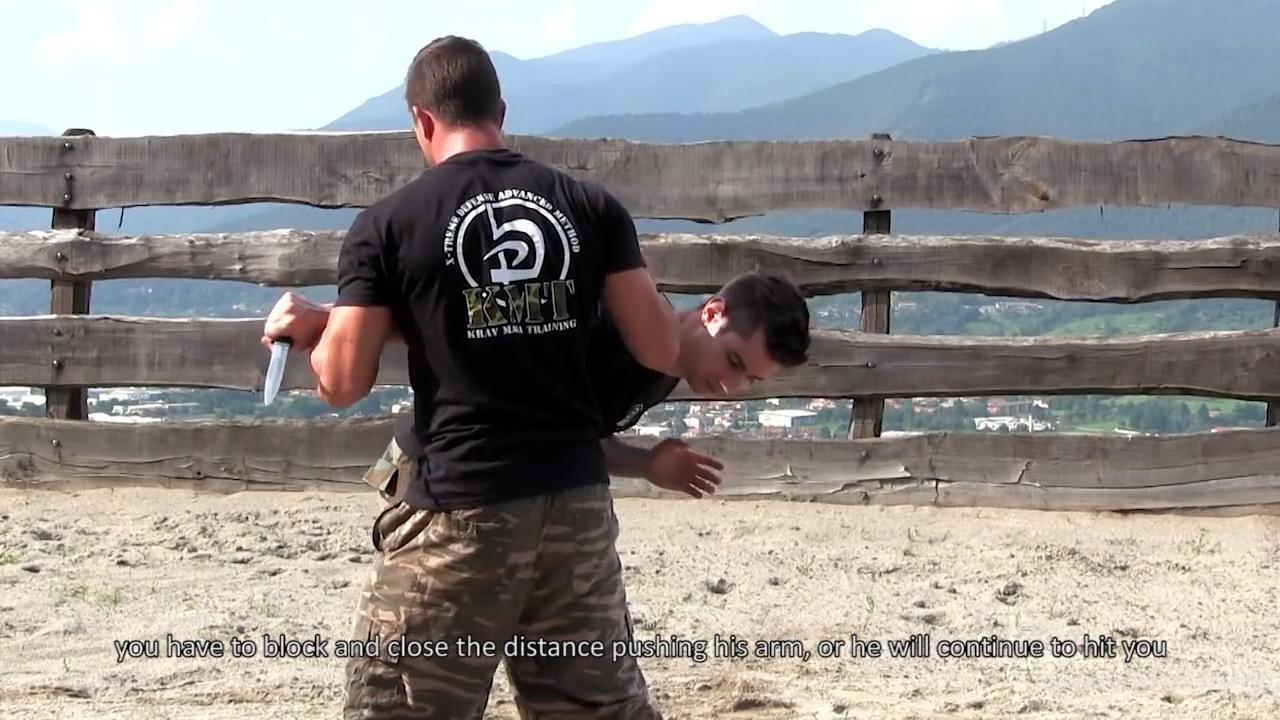

Supplement: Supplementary file 2 — Supplementary Information 2. [file 41598_2023_35190_MOESM2_ESM.zip › test/images/KravMagaTraining20404_jpg.rf.6359f3b8c362cb8073971e2a49d59fe3.jpg]

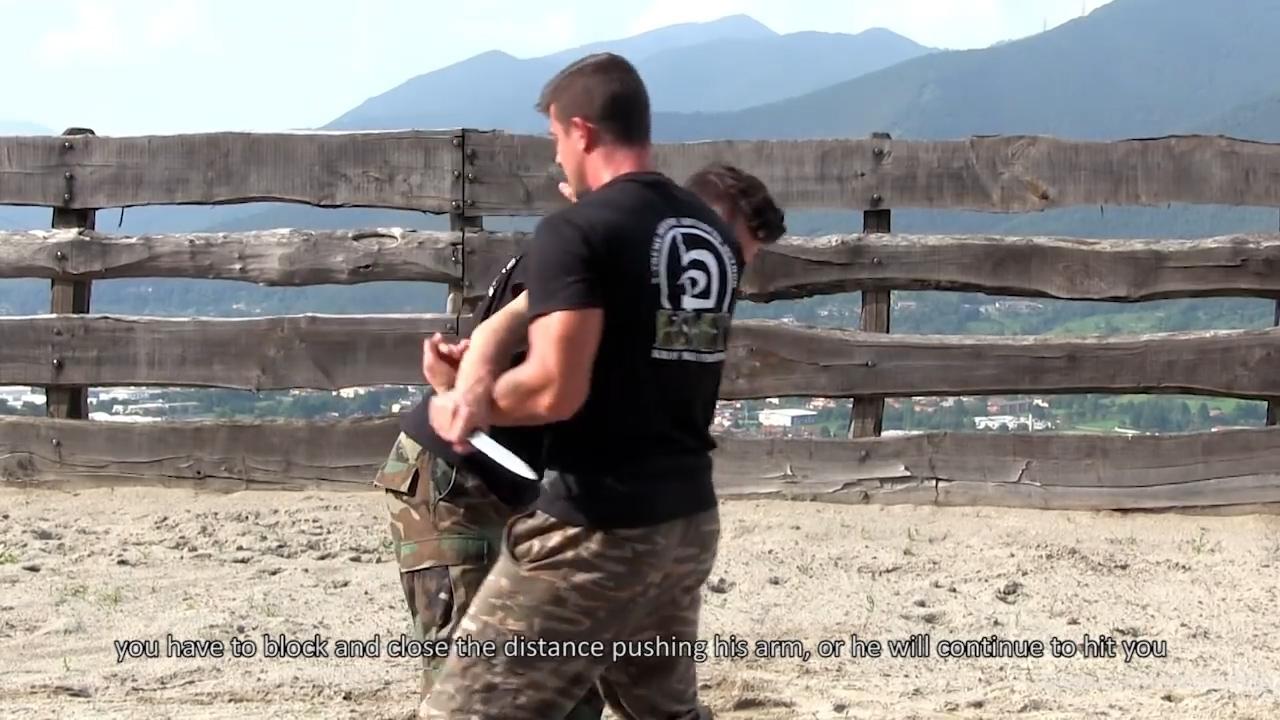

Supplement: Supplementary file 2 — Supplementary Information 2. [file 41598_2023_35190_MOESM2_ESM.zip › test/images/KravMagaTraining20406_jpg.rf.792e45fb54f93dceed8e917e6286688c.jpg]

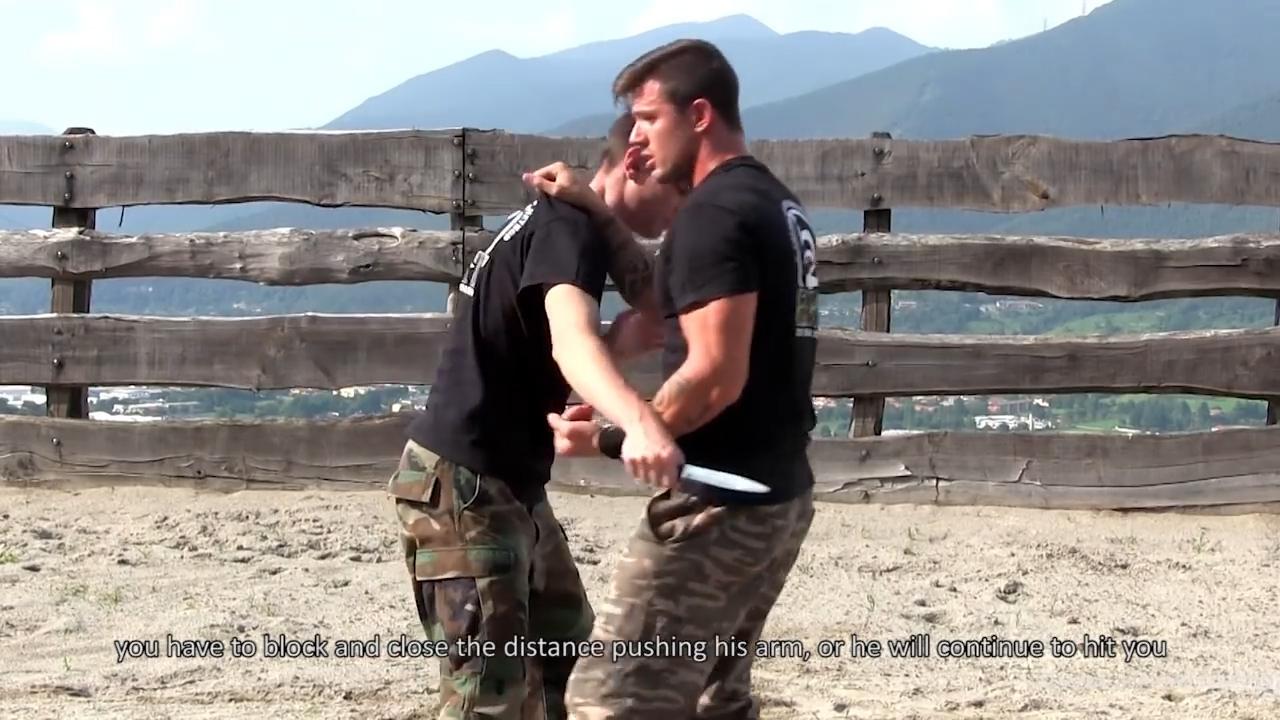

Supplement: Supplementary file 2 — Supplementary Information 2. [file 41598_2023_35190_MOESM2_ESM.zip › test/images/KravMagaTraining20407_jpg.rf.af4613839aa7749303c2838177d00e69.jpg]

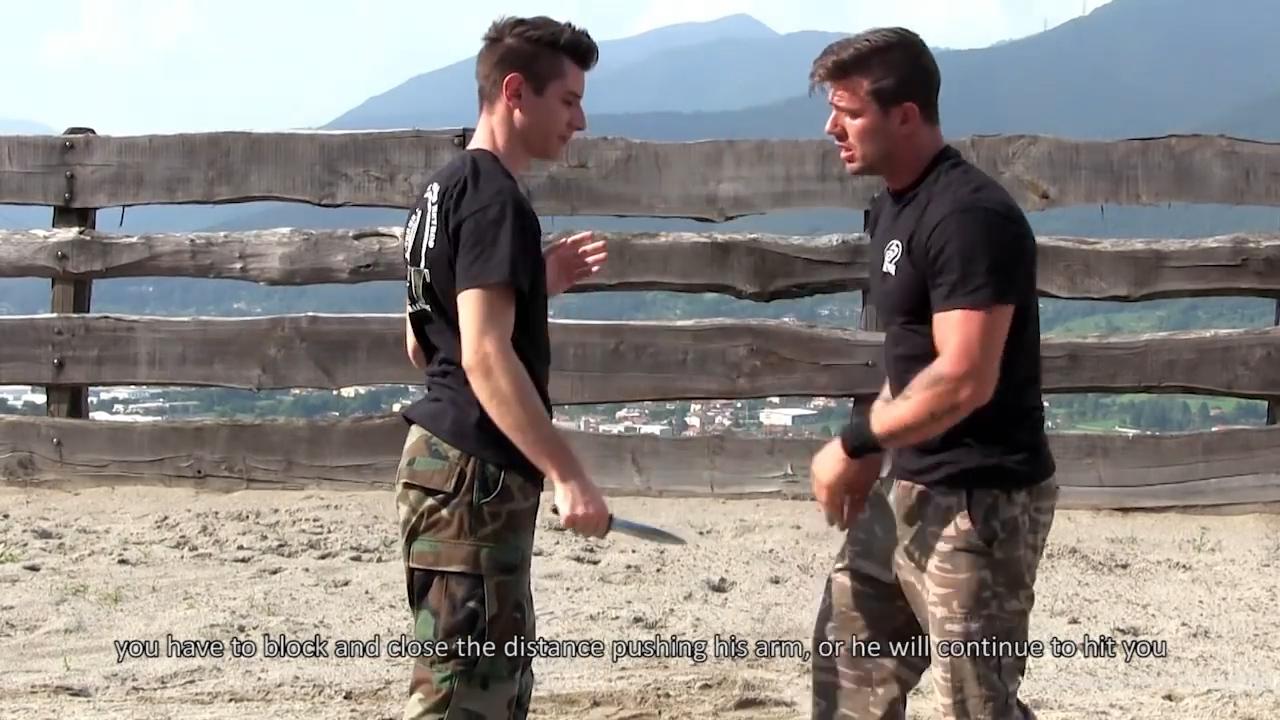

Supplement: Supplementary file 2 — Supplementary Information 2. [file 41598_2023_35190_MOESM2_ESM.zip › test/images/KravMagaTraining20410_jpg.rf.2e73919a46ff8bec2508856a6e4b2c23.jpg]

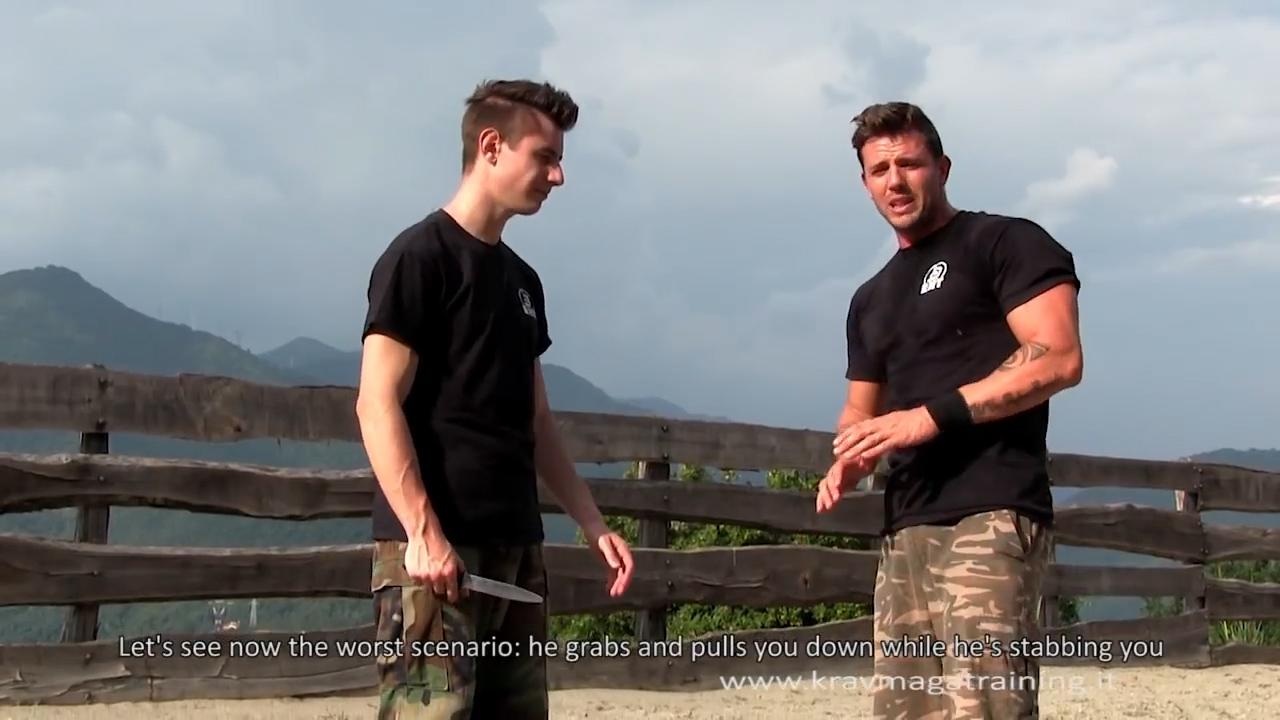

Supplement: Supplementary file 2 — Supplementary Information 2. [file 41598_2023_35190_MOESM2_ESM.zip › test/images/KravMagaTraining20582_jpg.rf.3c28fdf067583493b071264777f5d67a.jpg]

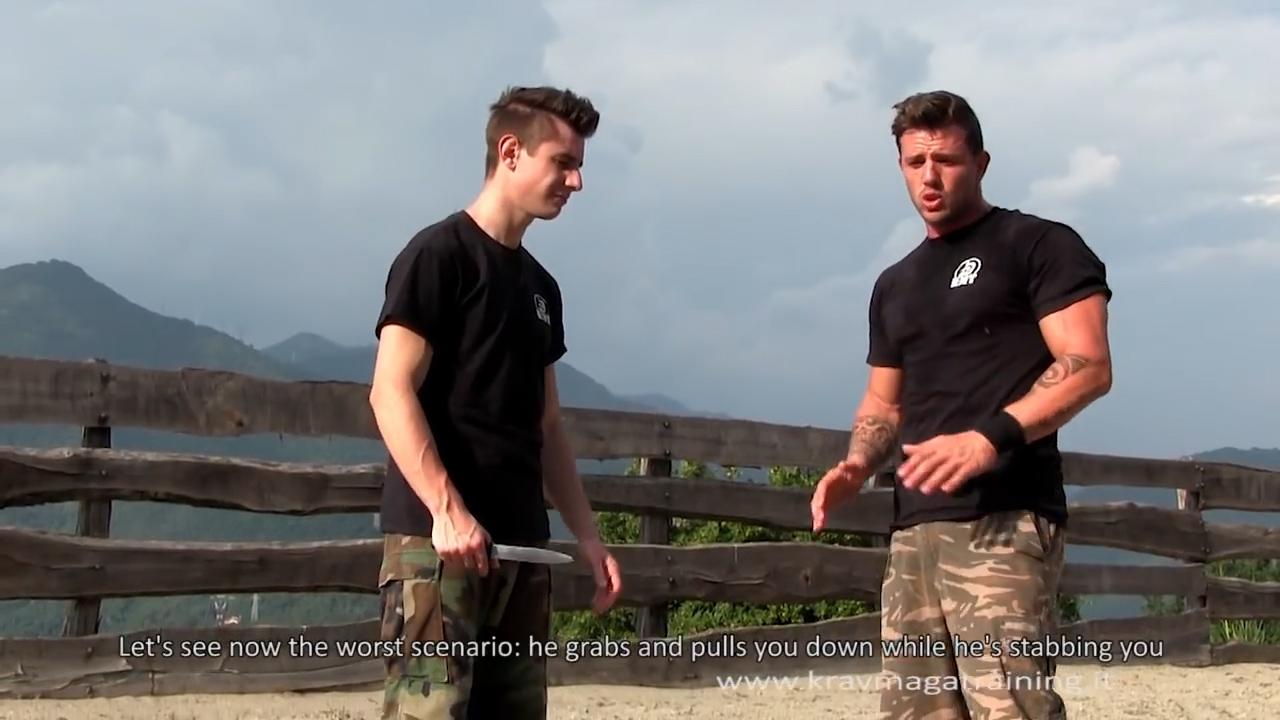

Supplement: Supplementary file 2 — Supplementary Information 2. [file 41598_2023_35190_MOESM2_ESM.zip › test/images/KravMagaTraining20583_jpg.rf.07dc2128a3609beaa580dda29ad7798f.jpg]

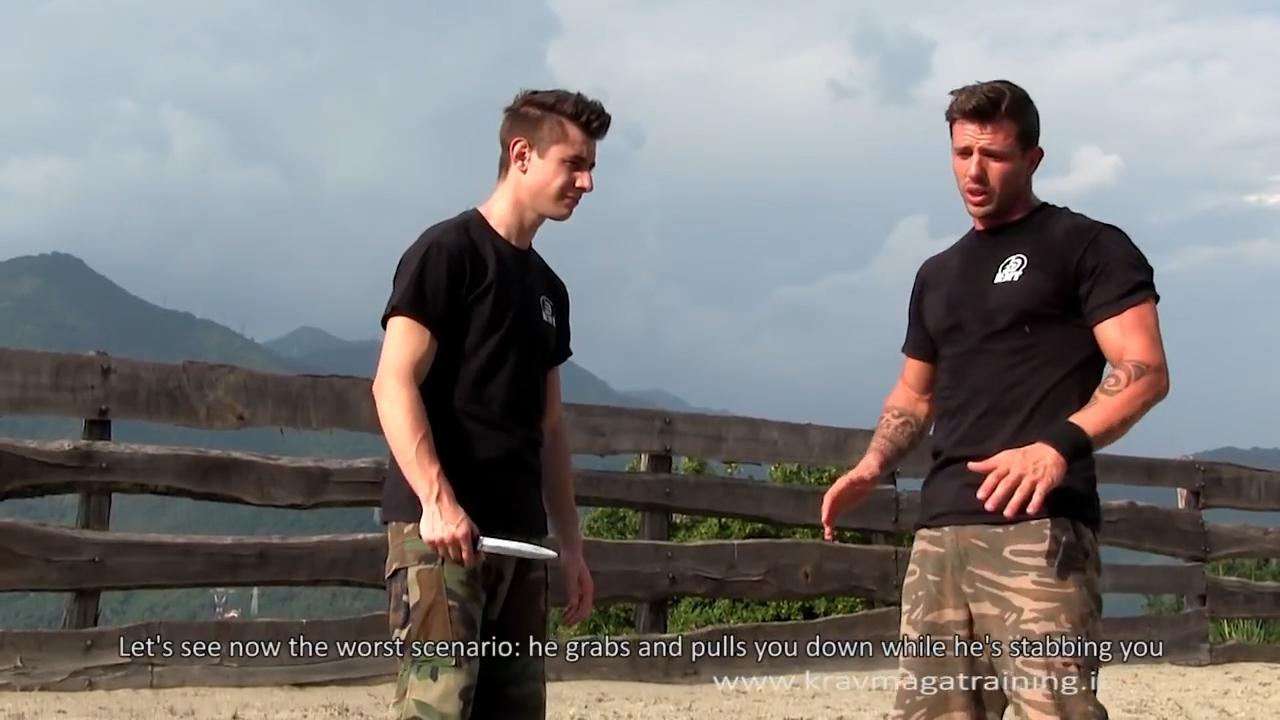

Supplement: Supplementary file 2 — Supplementary Information 2. [file 41598_2023_35190_MOESM2_ESM.zip › test/images/KravMagaTraining20584_jpg.rf.edc64fc2d2b9a052a413703595c2bf0a.jpg]

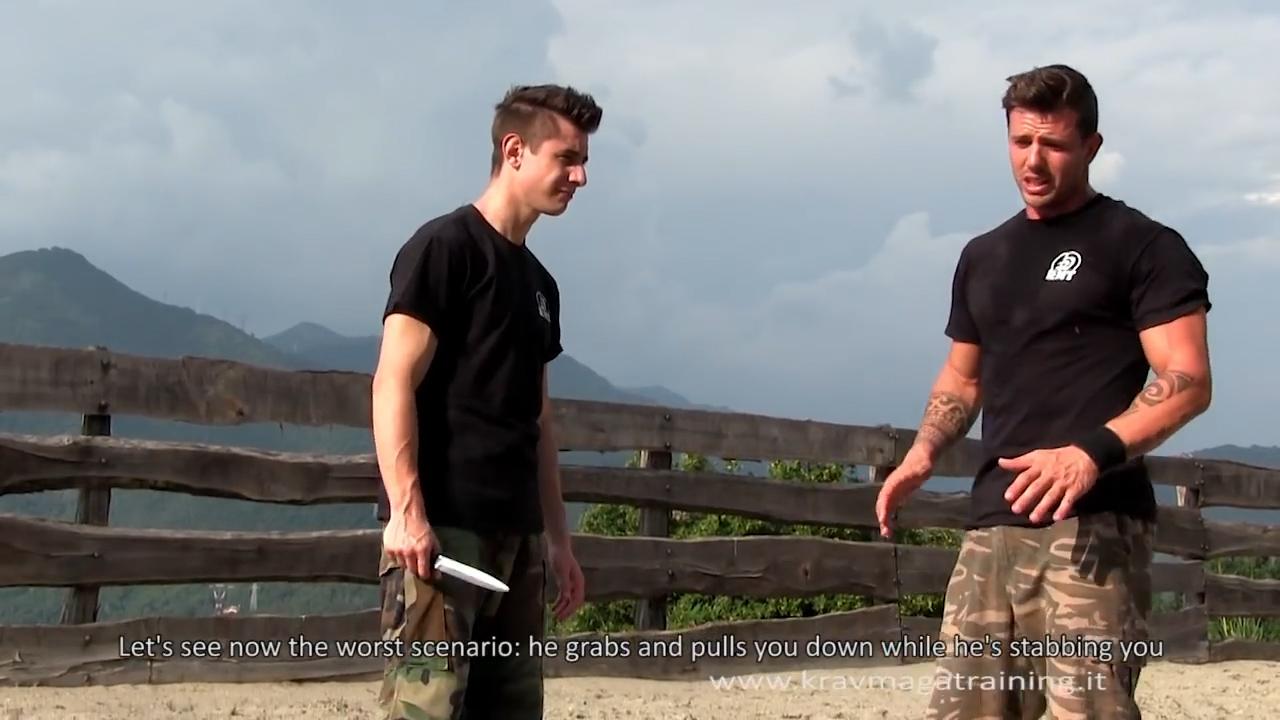

Supplement: Supplementary file 2 — Supplementary Information 2. [file 41598_2023_35190_MOESM2_ESM.zip › test/images/KravMagaTraining20585_jpg.rf.44a0561f7019e61deb447a4e97c92699.jpg]

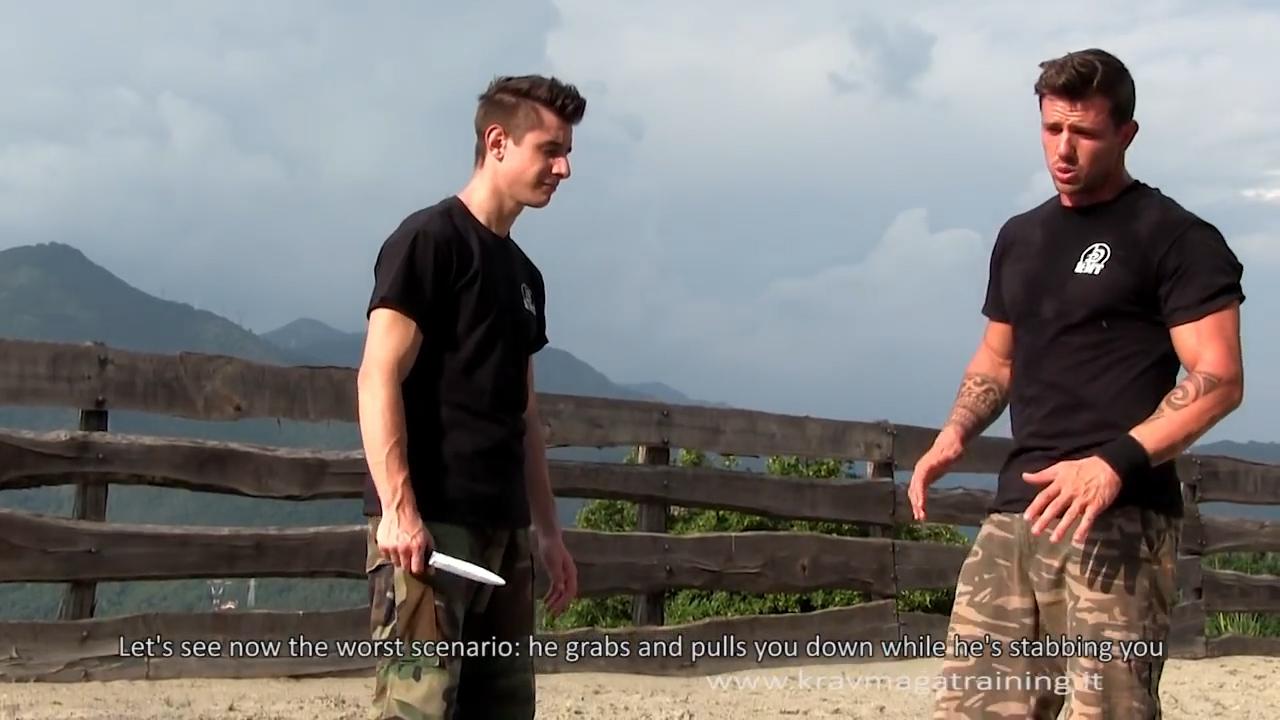

Supplement: Supplementary file 2 — Supplementary Information 2. [file 41598_2023_35190_MOESM2_ESM.zip › test/images/KravMagaTraining20586_jpg.rf.05b47a02fa6459bbea111cce485438e6.jpg]

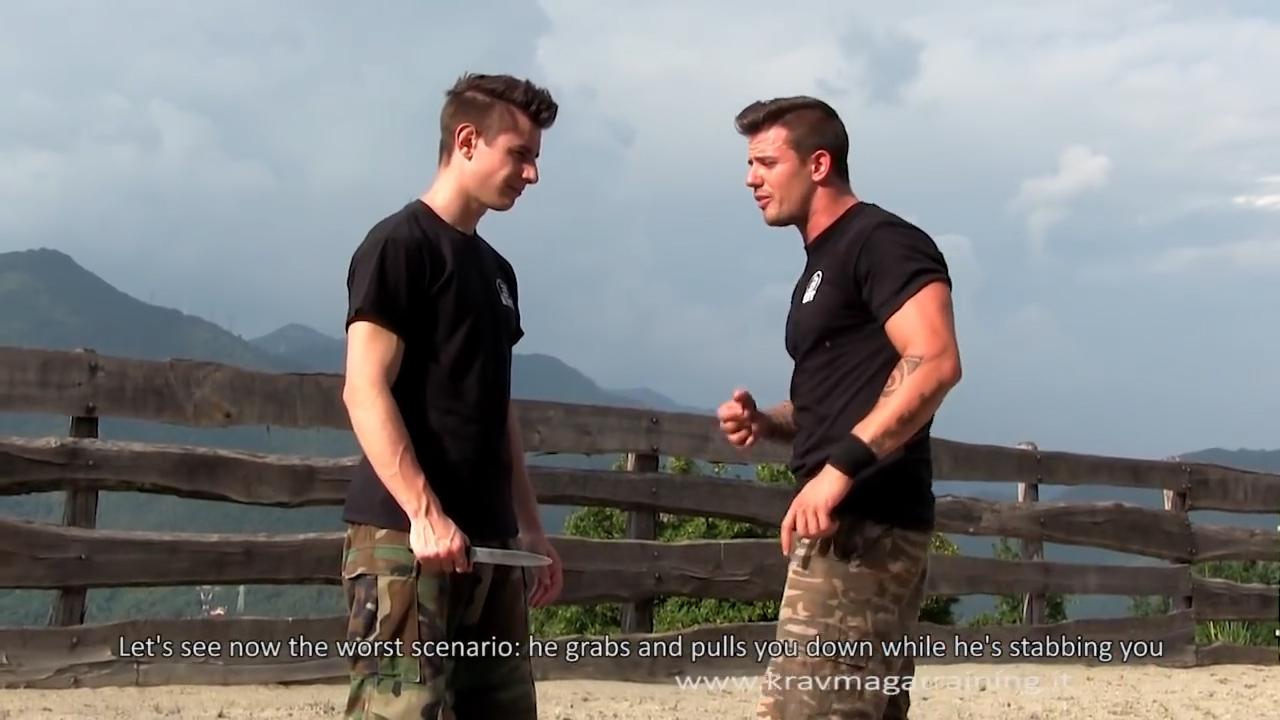

Supplement: Supplementary file 2 — Supplementary Information 2. [file 41598_2023_35190_MOESM2_ESM.zip › test/images/KravMagaTraining20595_jpg.rf.f90b0fbb7d1093e88fa5e6fec78ca972.jpg]

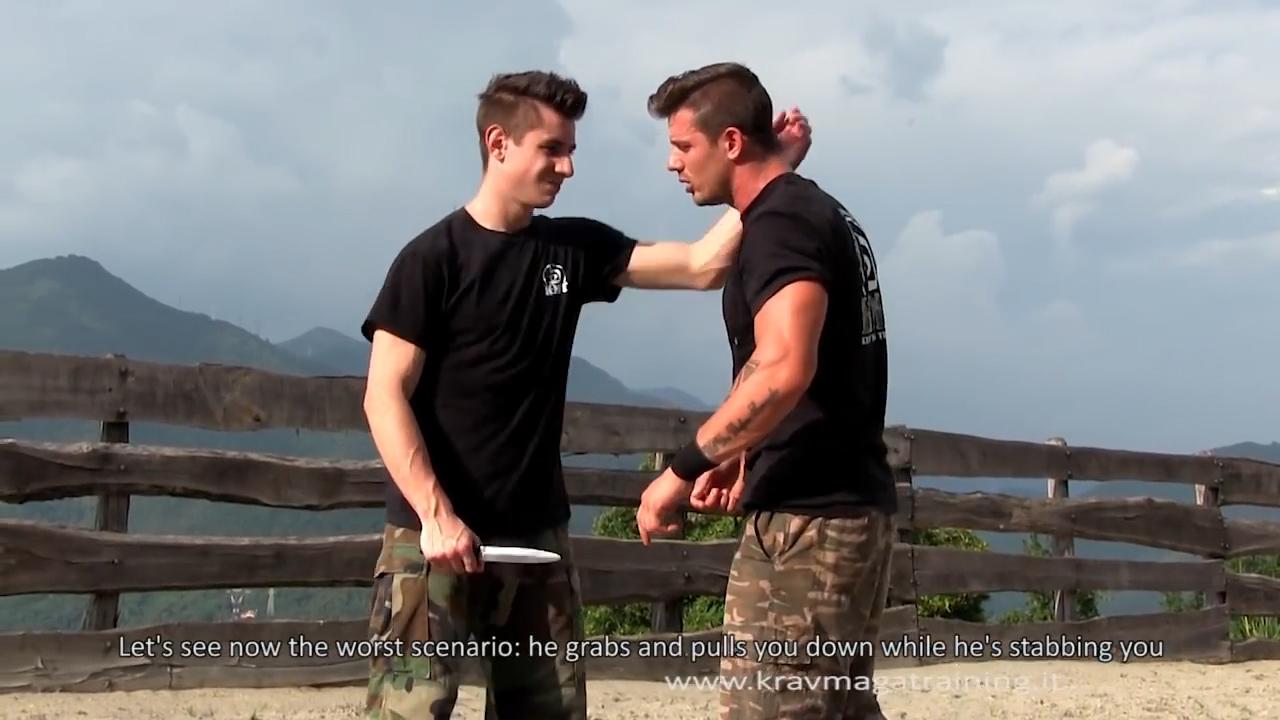

Supplement: Supplementary file 2 — Supplementary Information 2. [file 41598_2023_35190_MOESM2_ESM.zip › test/images/KravMagaTraining20598_jpg.rf.5b0f9b700e4c436bb79ba4538bf59170.jpg]

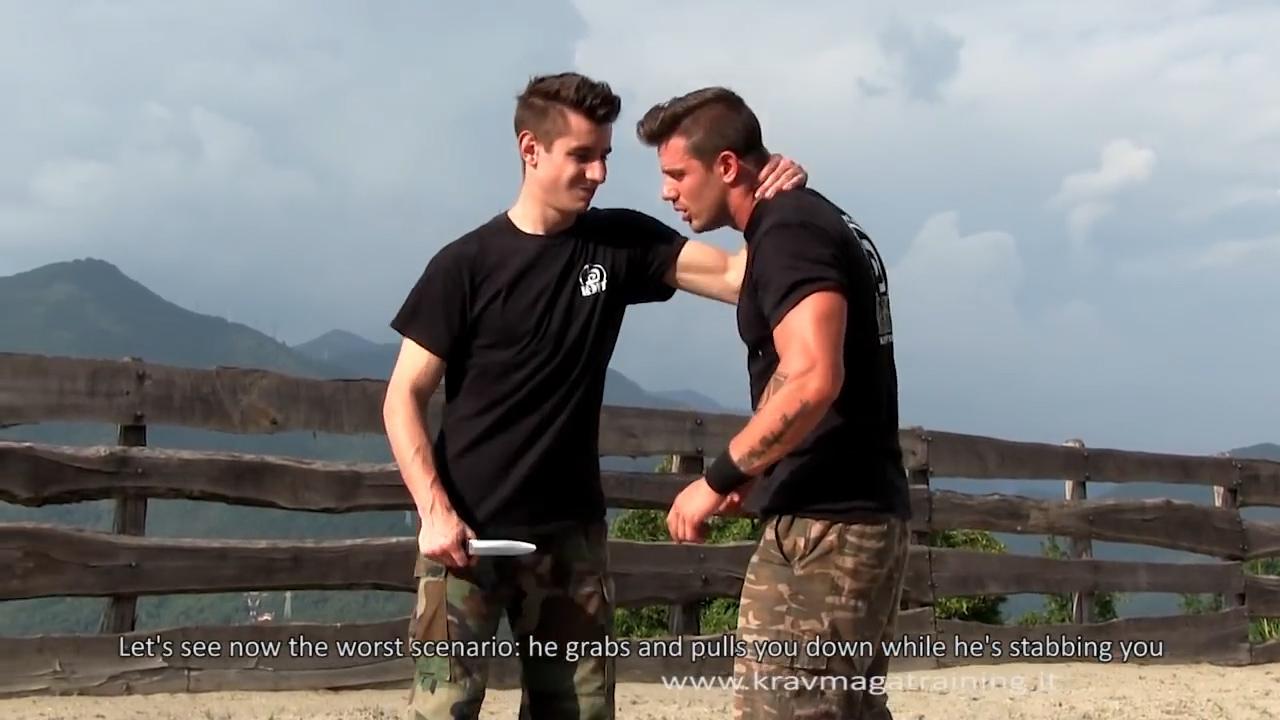

Supplement: Supplementary file 2 — Supplementary Information 2. [file 41598_2023_35190_MOESM2_ESM.zip › test/images/KravMagaTraining20599_jpg.rf.e68fc924b432343ee0ceae8a67b29f31.jpg]

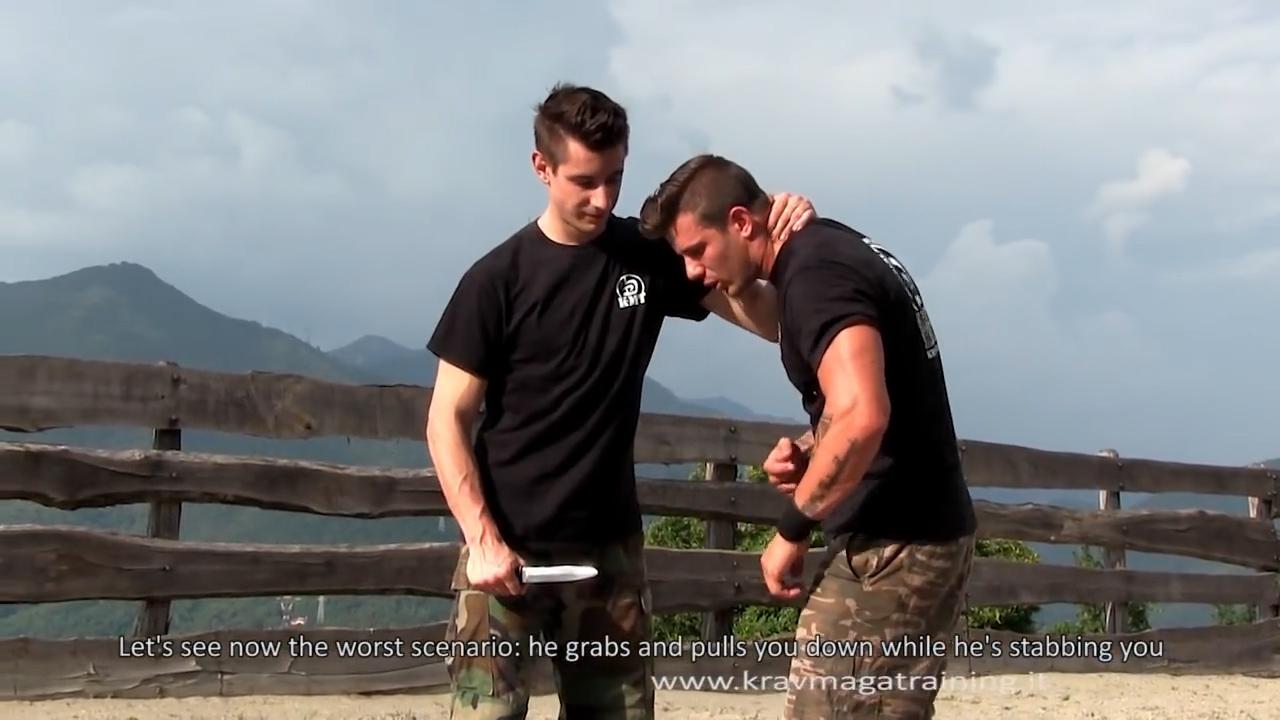

Supplement: Supplementary file 2 — Supplementary Information 2. [file 41598_2023_35190_MOESM2_ESM.zip › test/images/KravMagaTraining20602_jpg.rf.5a50ef5e8c9034028713b8977ec5632a.jpg]

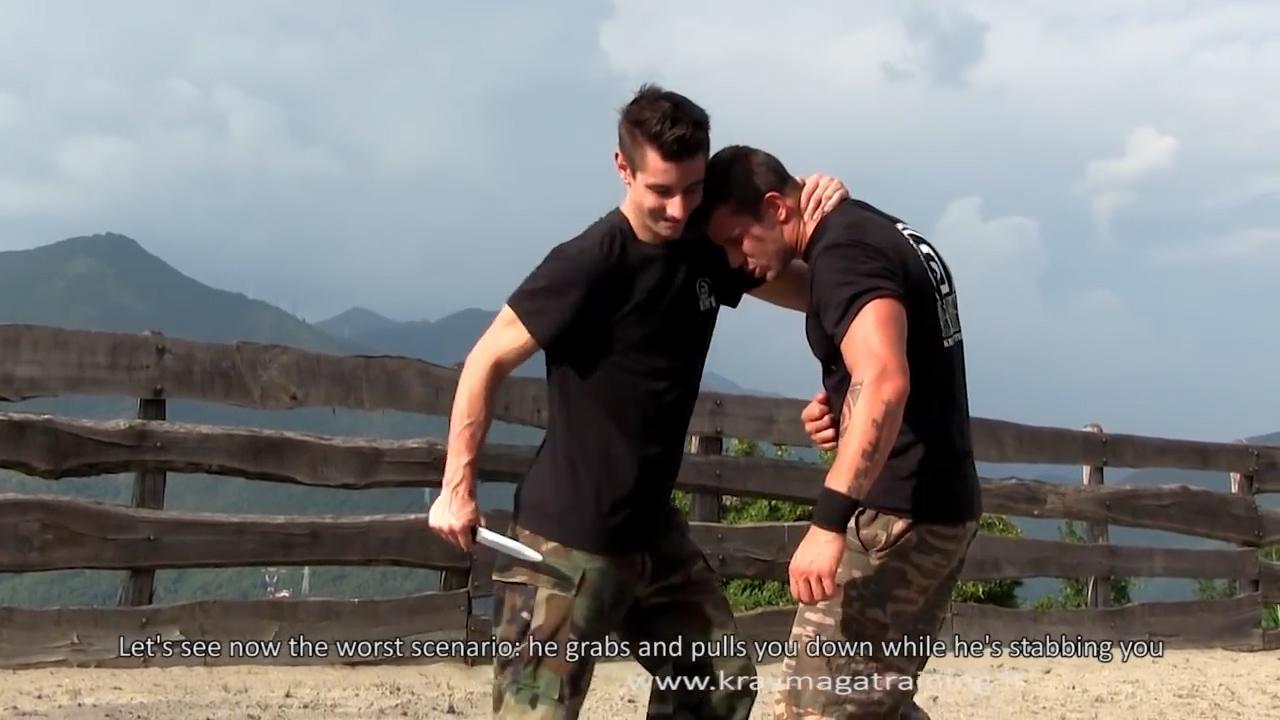

Supplement: Supplementary file 2 — Supplementary Information 2. [file 41598_2023_35190_MOESM2_ESM.zip › test/images/KravMagaTraining20609_jpg.rf.8688d0603b0d302f4ed276b104fef2c5.jpg]

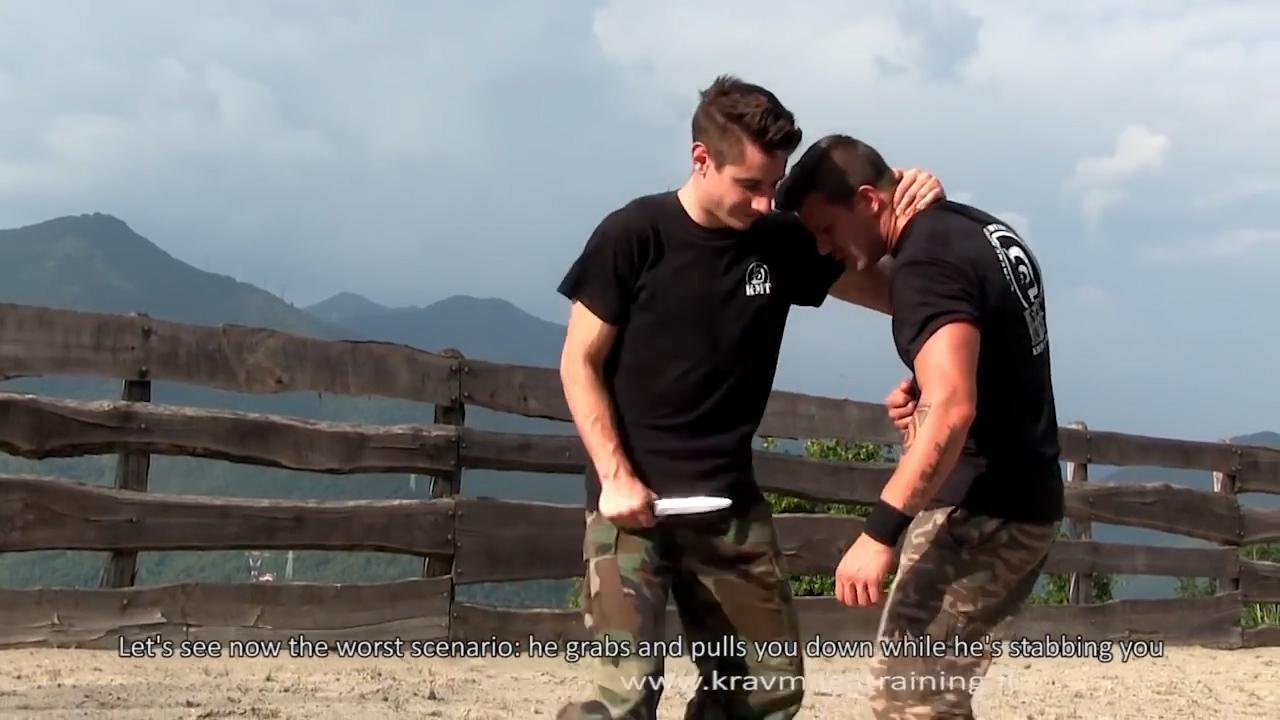

Supplement: Supplementary file 2 — Supplementary Information 2. [file 41598_2023_35190_MOESM2_ESM.zip › test/images/KravMagaTraining20611_jpg.rf.5f3f9b0979e9c309031f7f001d7f7890.jpg]

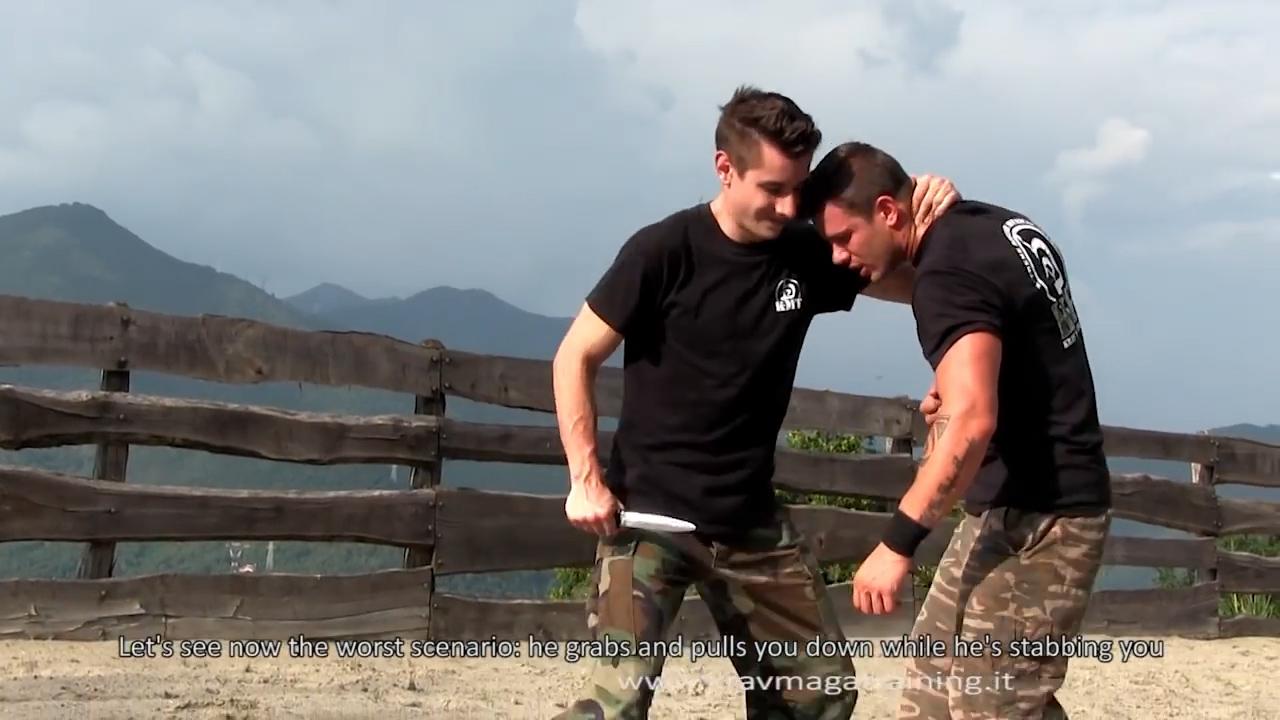

Supplement: Supplementary file 2 — Supplementary Information 2. [file 41598_2023_35190_MOESM2_ESM.zip › test/images/KravMagaTraining20612_jpg.rf.21283627468fff0d9eb772b244e8f7e6.jpg]

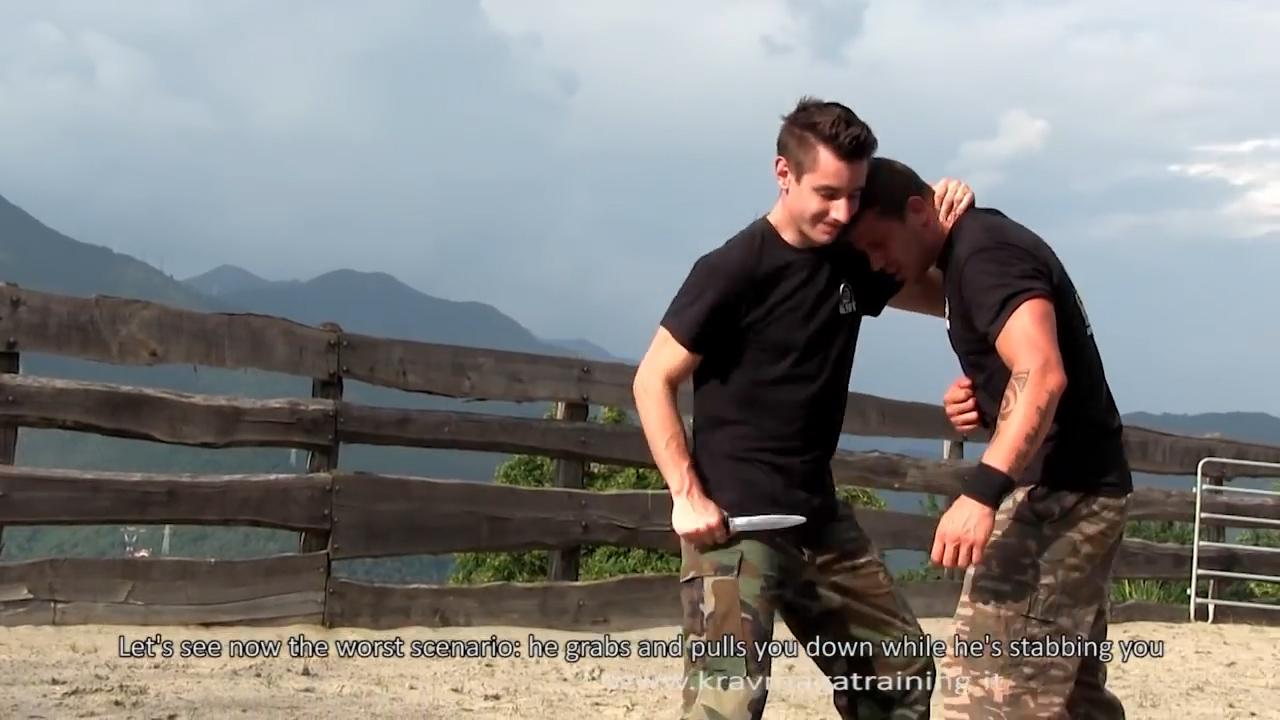

Supplement: Supplementary file 2 — Supplementary Information 2. [file 41598_2023_35190_MOESM2_ESM.zip › test/images/KravMagaTraining20615_jpg.rf.0a9defdd4b46293c1ecb04883ccb7f0d.jpg]

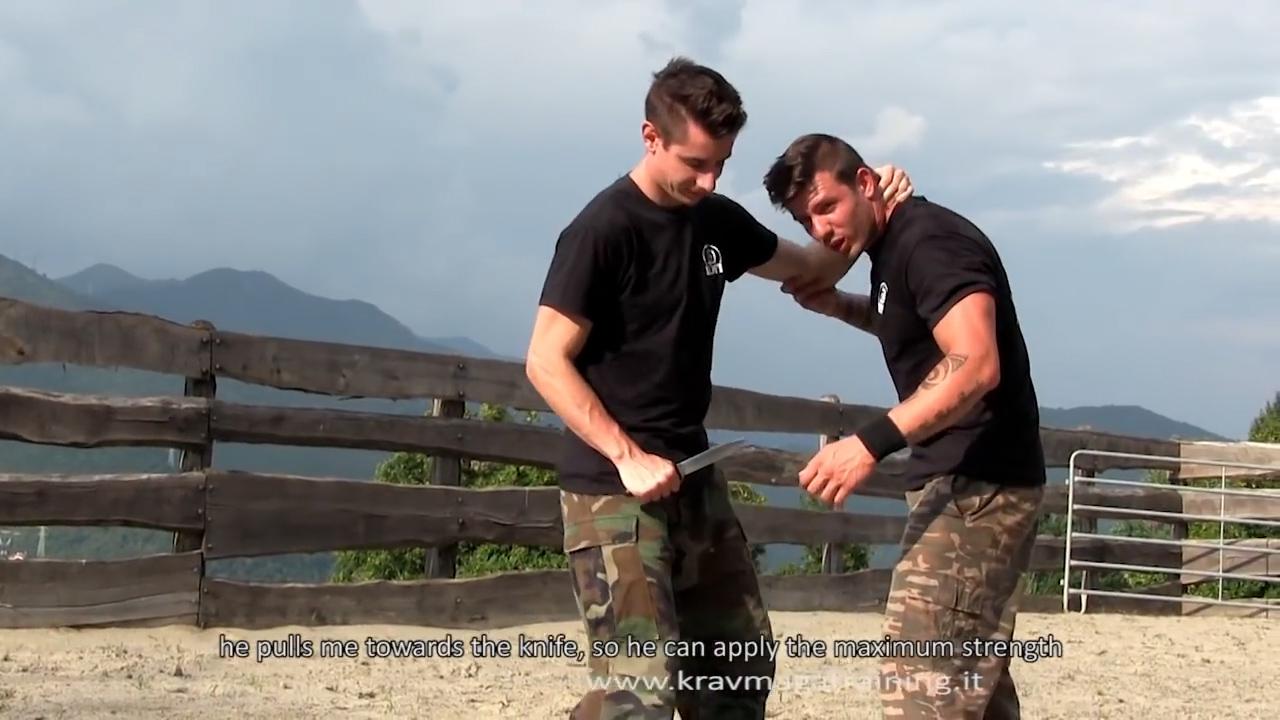

Supplement: Supplementary file 2 — Supplementary Information 2. [file 41598_2023_35190_MOESM2_ESM.zip › test/images/KravMagaTraining20621_jpg.rf.f6274820571e4e8b5c196ef942cbded0.jpg]

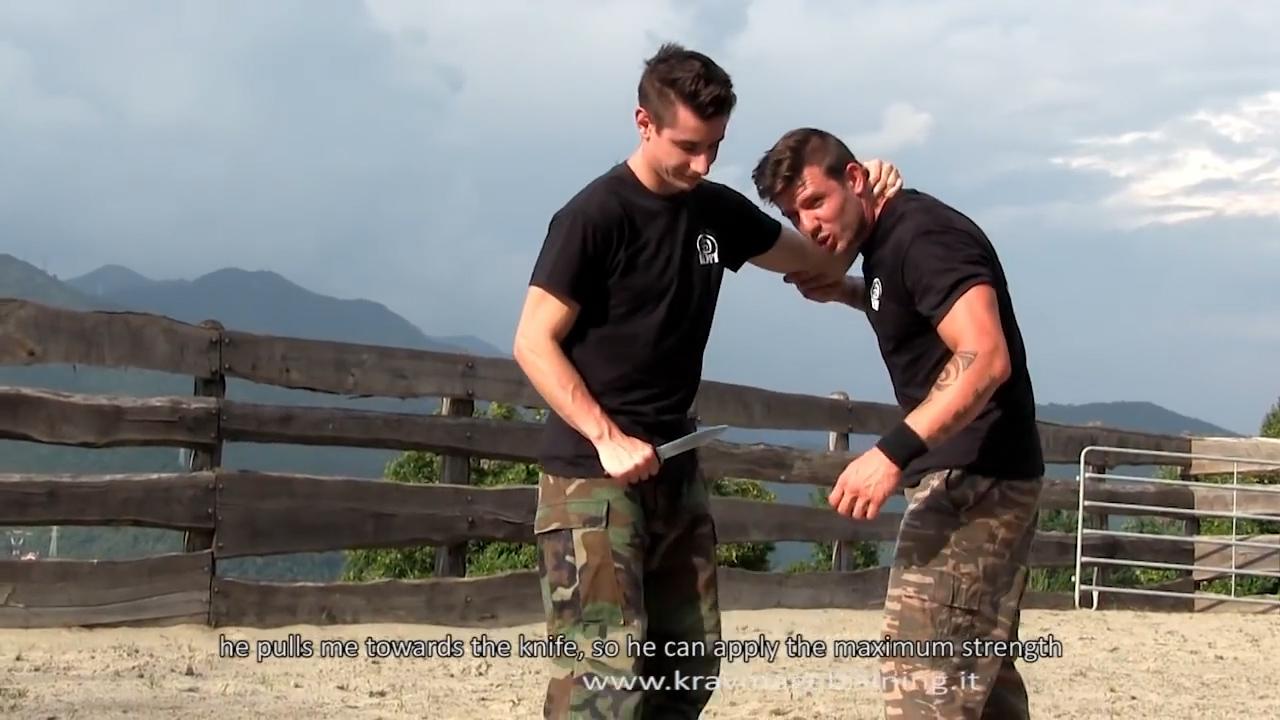

Supplement: Supplementary file 2 — Supplementary Information 2. [file 41598_2023_35190_MOESM2_ESM.zip › test/images/KravMagaTraining20622_jpg.rf.3524b6288f06c4e4ab2c082c3af01605.jpg]

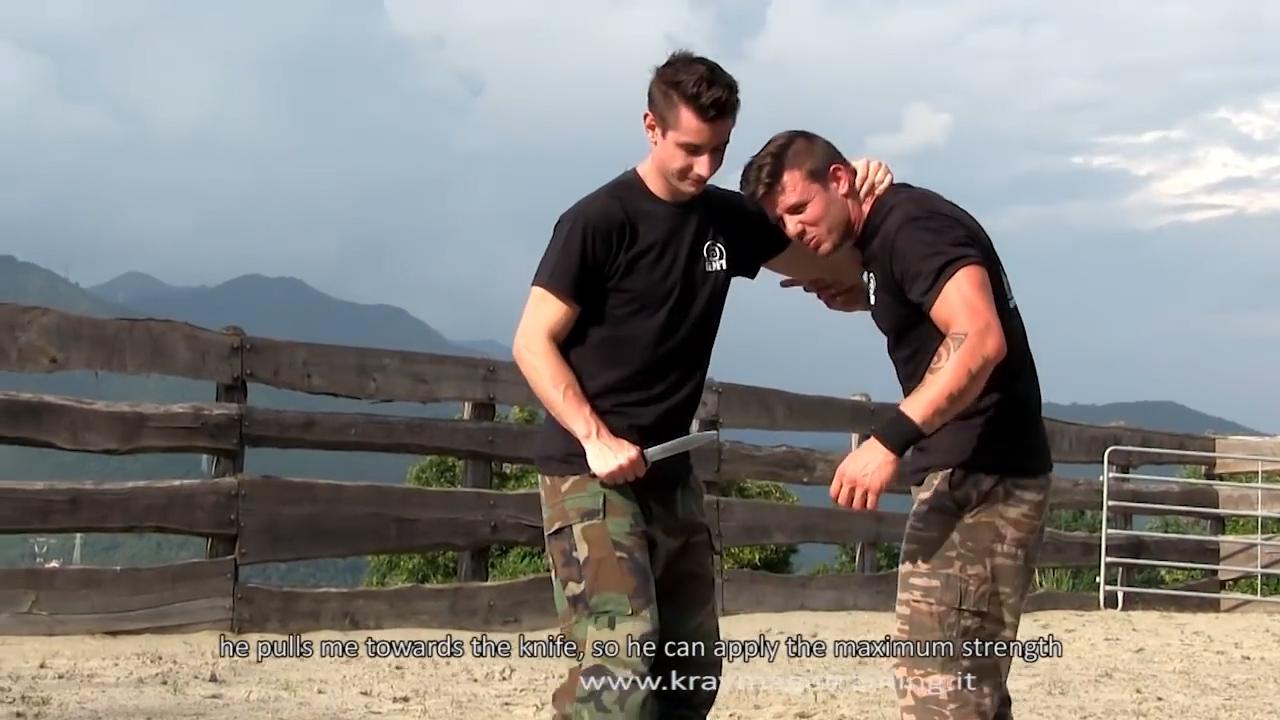

Supplement: Supplementary file 2 — Supplementary Information 2. [file 41598_2023_35190_MOESM2_ESM.zip › test/images/KravMagaTraining20623_jpg.rf.9318c703bf7b7c73ecd188f5422a4903.jpg]

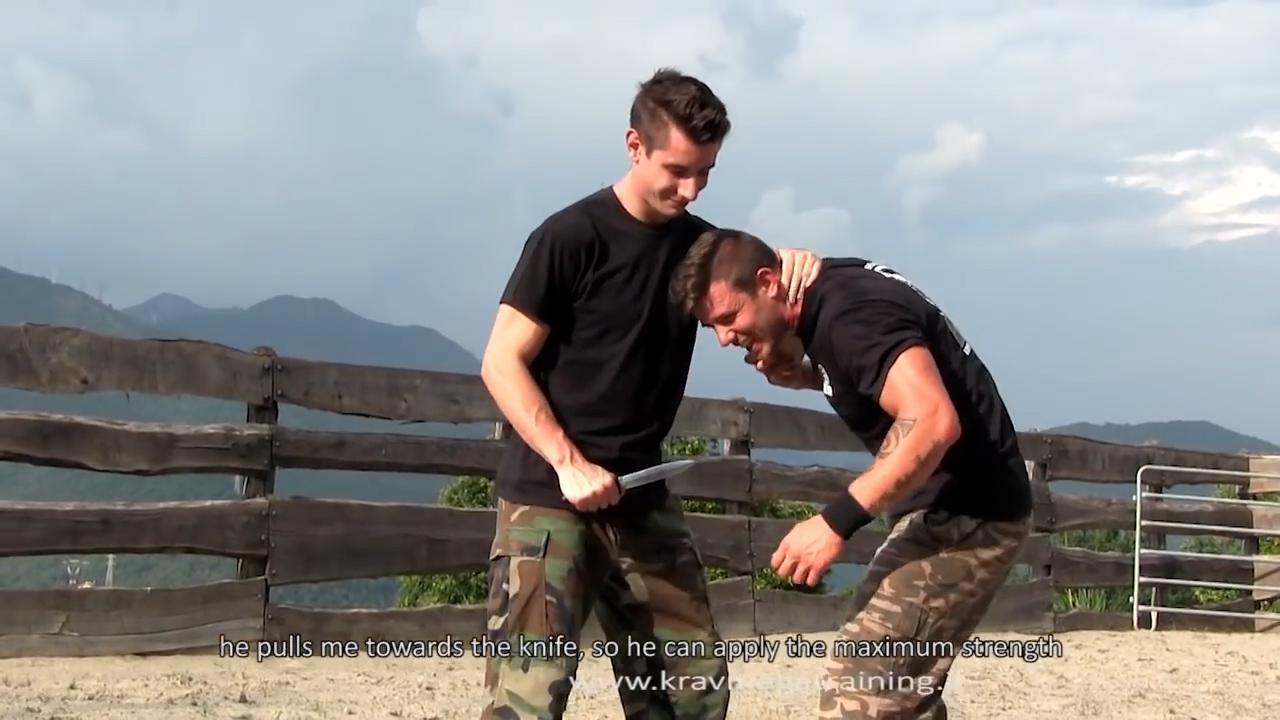

Supplement: Supplementary file 2 — Supplementary Information 2. [file 41598_2023_35190_MOESM2_ESM.zip › test/images/KravMagaTraining20626_jpg.rf.adeed0879472cbbe384e518fbacc4614.jpg]

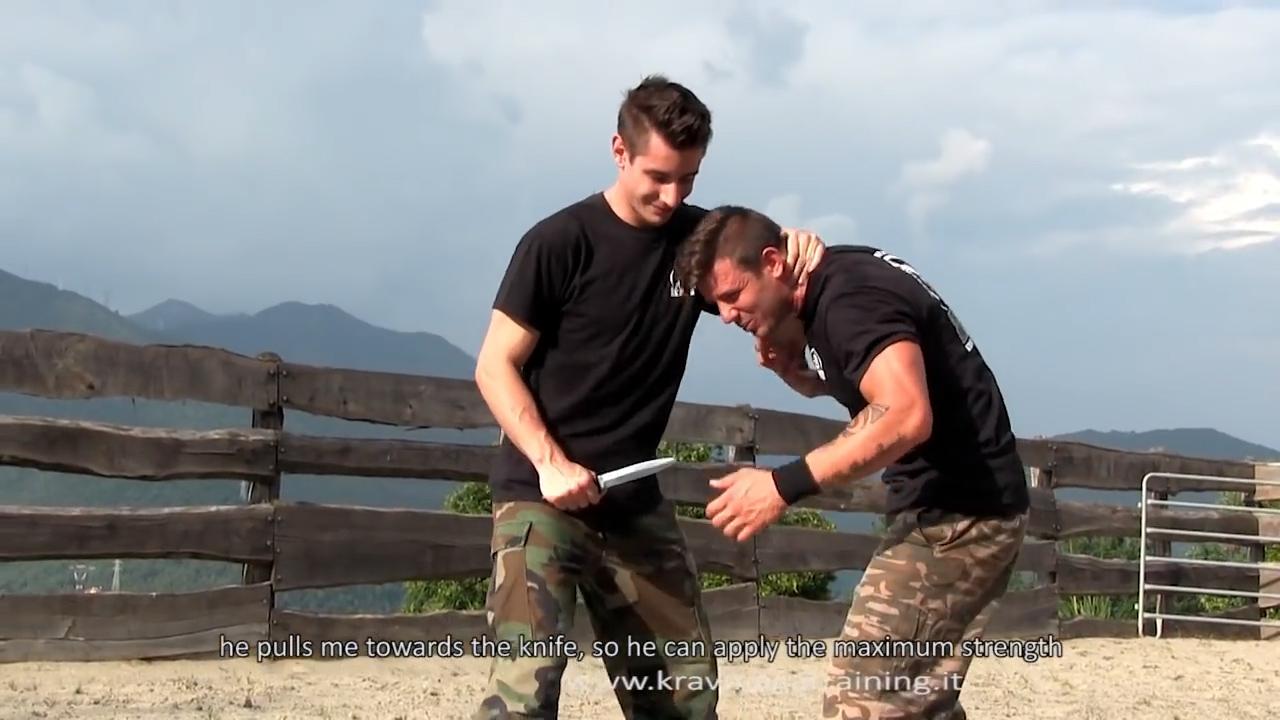

Supplement: Supplementary file 2 — Supplementary Information 2. [file 41598_2023_35190_MOESM2_ESM.zip › test/images/KravMagaTraining20627_jpg.rf.e58f8952f4b15231ad1960dfa72f6d0d.jpg]

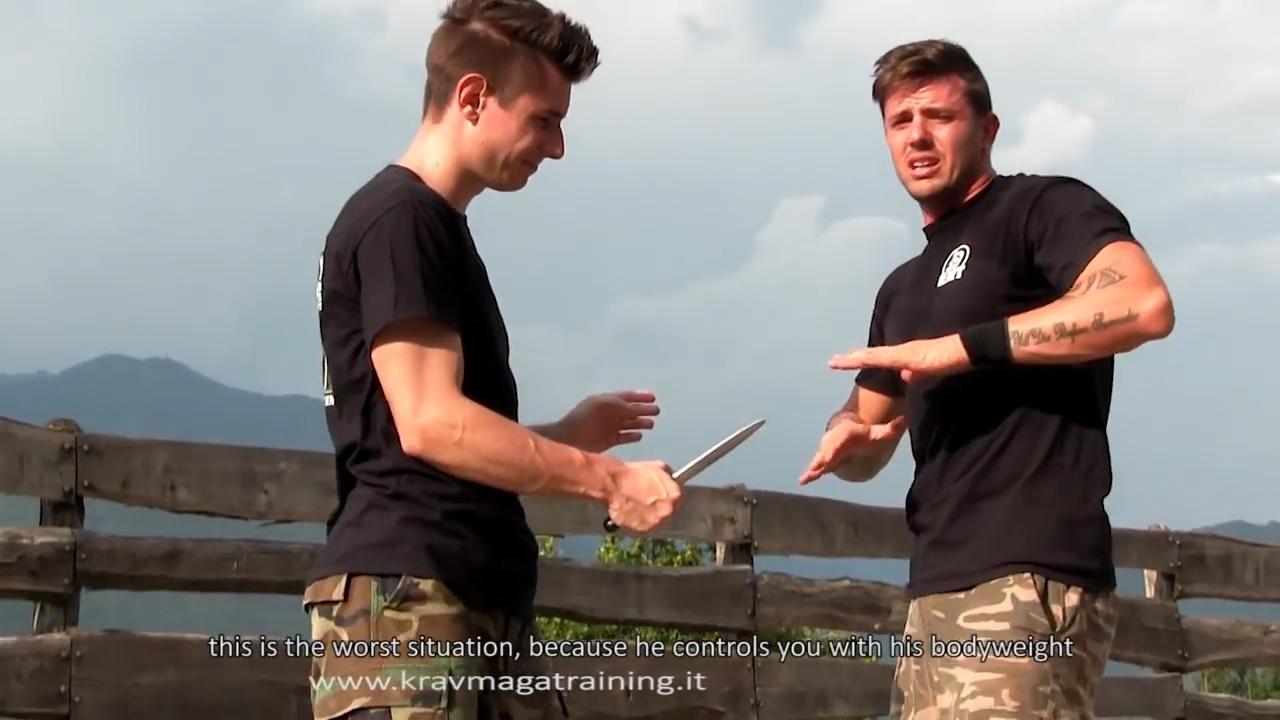

Supplement: Supplementary file 2 — Supplementary Information 2. [file 41598_2023_35190_MOESM2_ESM.zip › test/images/KravMagaTraining20702_jpg.rf.4ac716e0673b059d87a9baaa1ab64985.jpg]

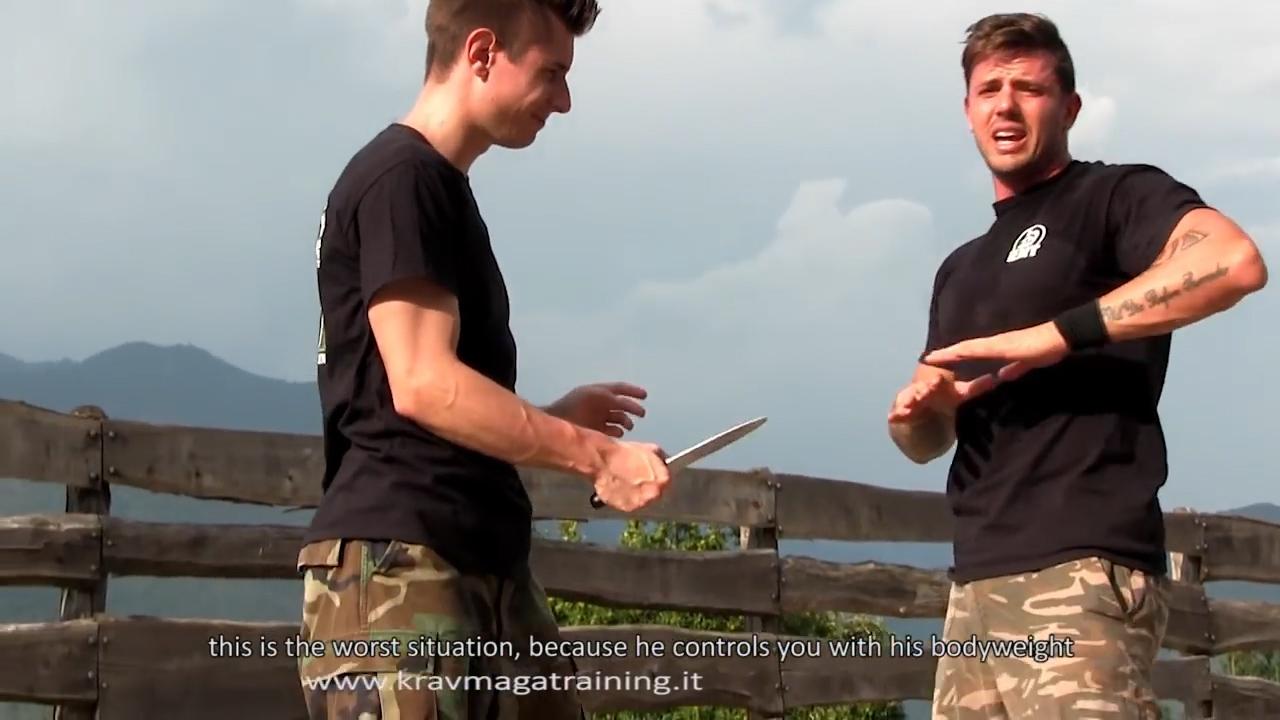

Supplement: Supplementary file 2 — Supplementary Information 2. [file 41598_2023_35190_MOESM2_ESM.zip › test/images/KravMagaTraining20703_jpg.rf.eafe826f6f69f9c22f78dcd27c44c1fb.jpg]

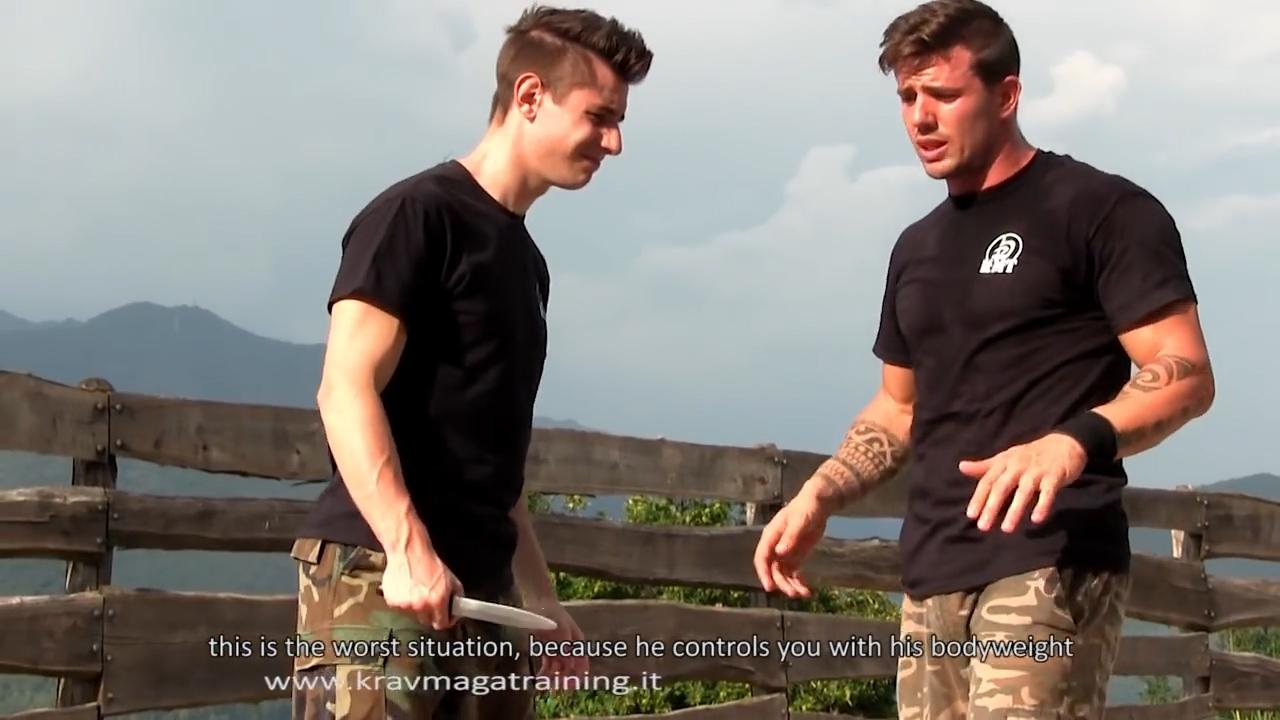

Supplement: Supplementary file 2 — Supplementary Information 2. [file 41598_2023_35190_MOESM2_ESM.zip › test/images/KravMagaTraining20715_jpg.rf.1faa5baee667e018480bce2d823e31a5.jpg]

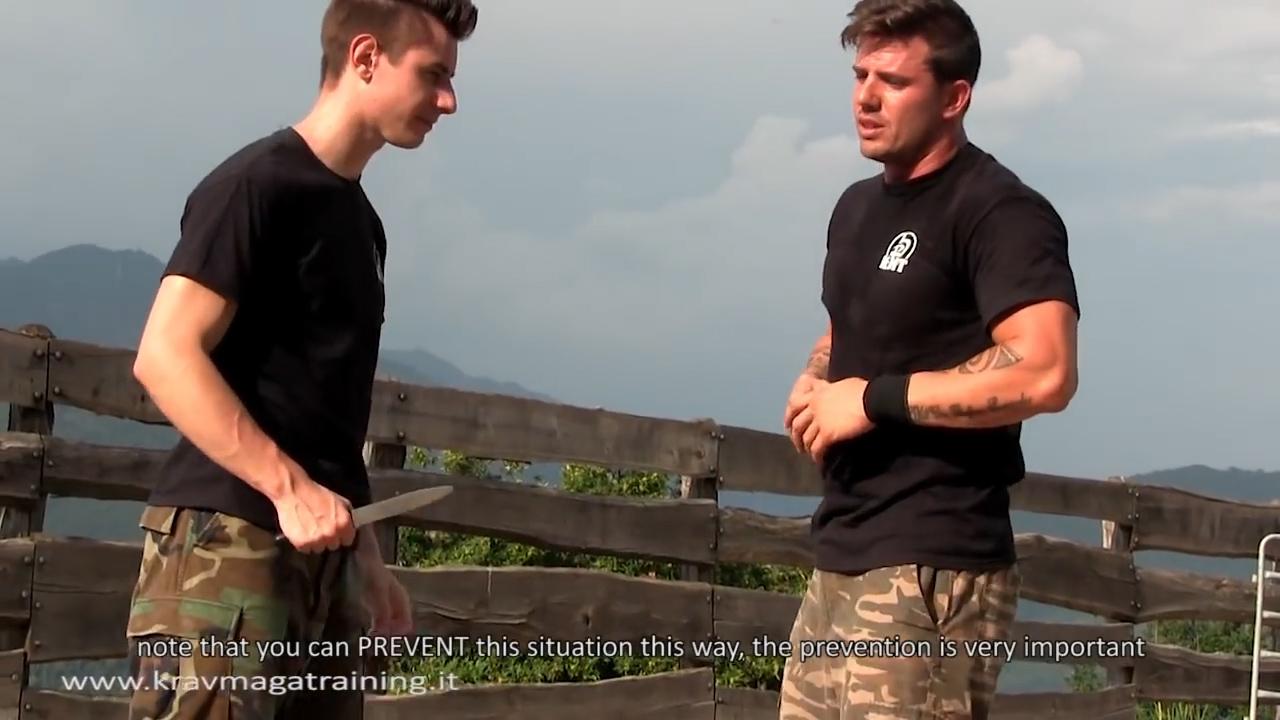

Supplement: Supplementary file 2 — Supplementary Information 2. [file 41598_2023_35190_MOESM2_ESM.zip › test/images/KravMagaTraining20774_jpg.rf.c21281b4cdd25a8684d0dbc98c64e910.jpg]

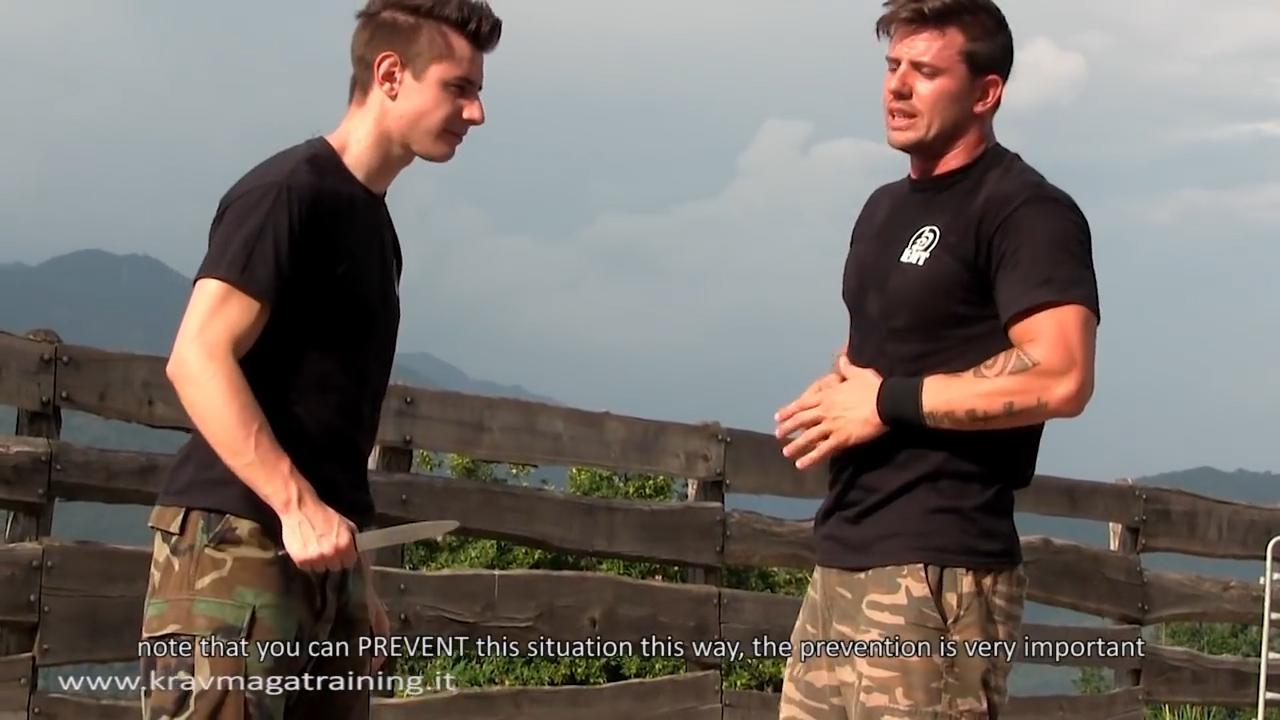

Supplement: Supplementary file 2 — Supplementary Information 2. [file 41598_2023_35190_MOESM2_ESM.zip › test/images/KravMagaTraining20775_jpg.rf.c001681b4085cb23b4494036b6cfb1b5.jpg]

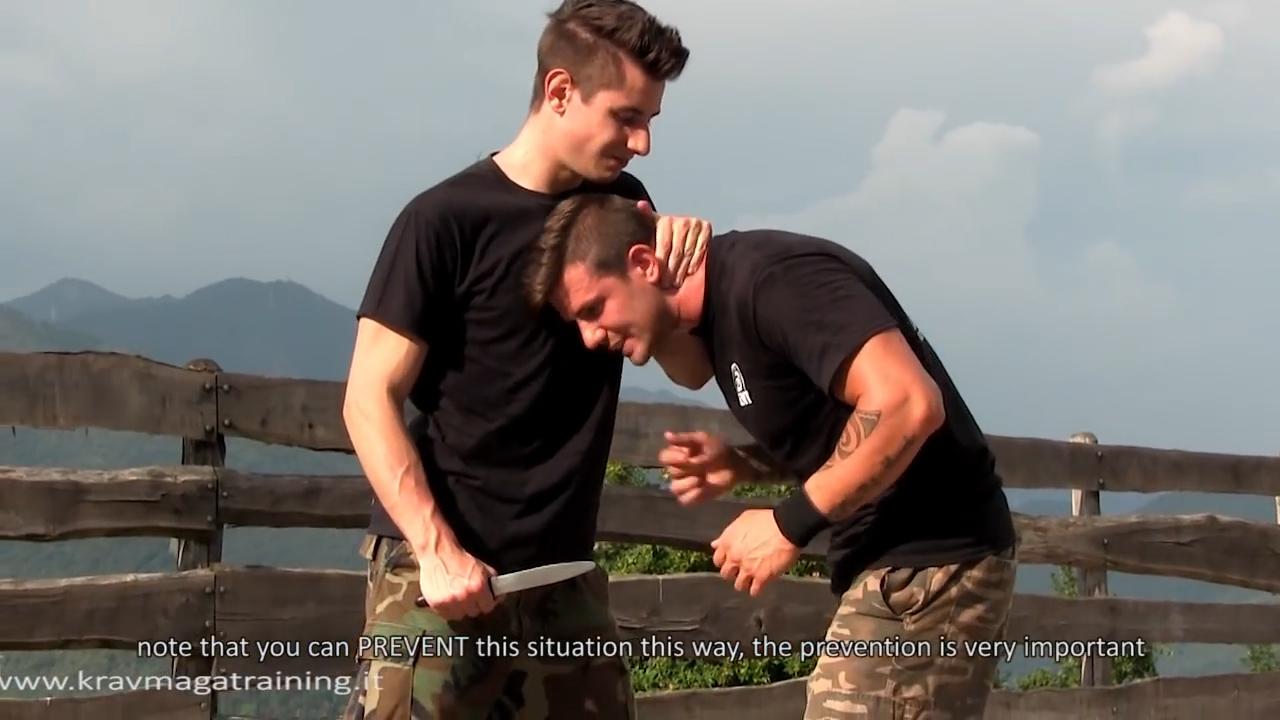

Supplement: Supplementary file 2 — Supplementary Information 2. [file 41598_2023_35190_MOESM2_ESM.zip › test/images/KravMagaTraining20796_jpg.rf.b41055325018e71fe6b2208a8764241c.jpg]

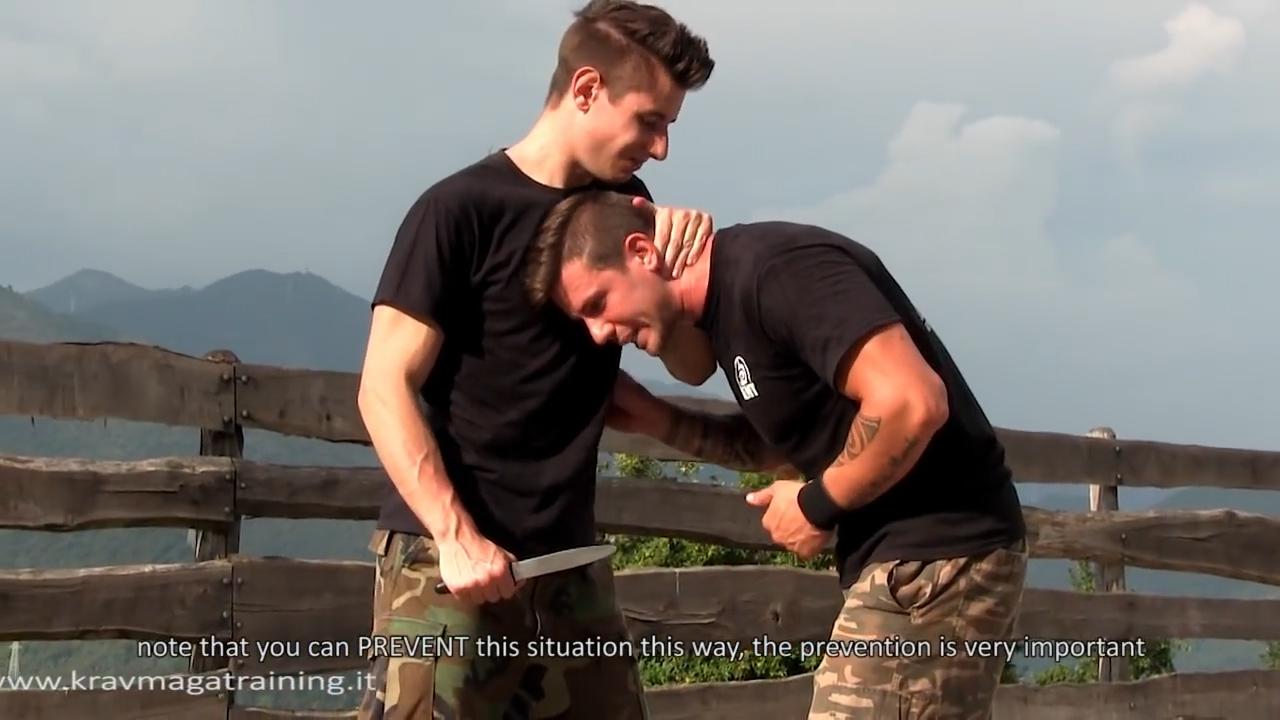

Supplement: Supplementary file 2 — Supplementary Information 2. [file 41598_2023_35190_MOESM2_ESM.zip › test/images/KravMagaTraining20798_jpg.rf.3078f7f8845d0ca9384b93c926690e3a.jpg]

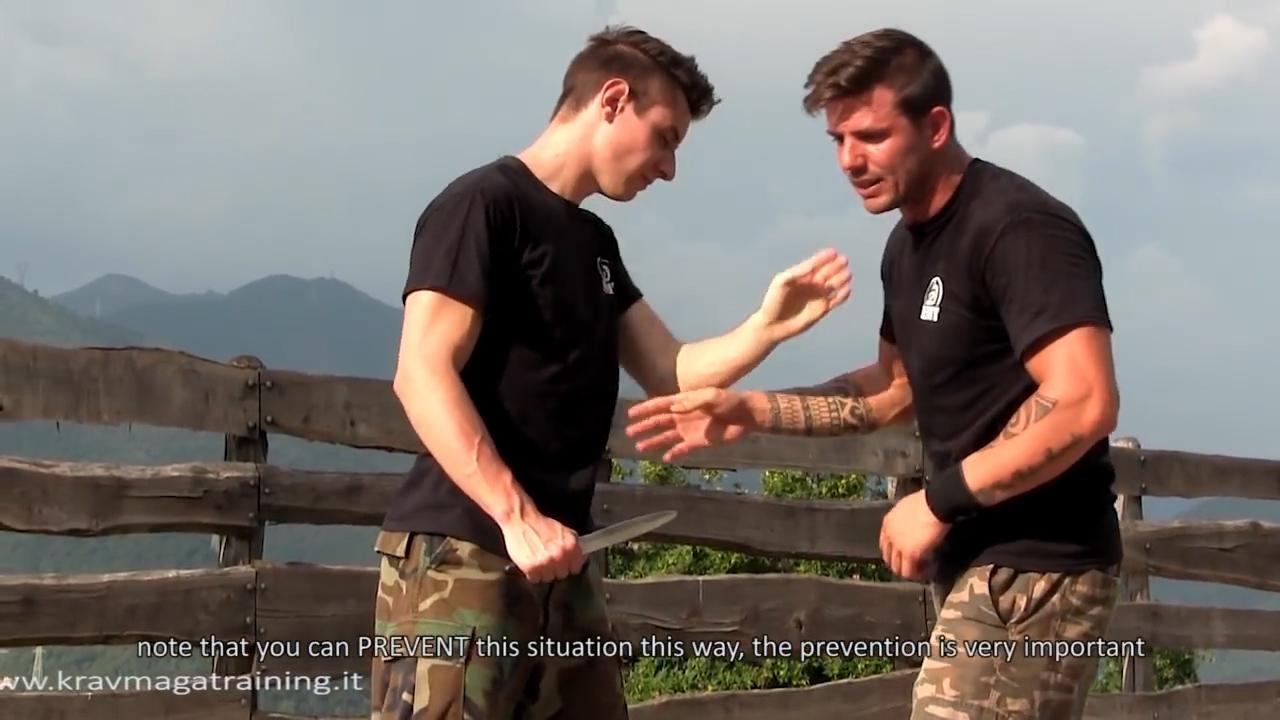

Supplement: Supplementary file 2 — Supplementary Information 2. [file 41598_2023_35190_MOESM2_ESM.zip › test/images/KravMagaTraining20802_jpg.rf.123cc5e4ae2f15adb477ef1b6c378a2c.jpg]

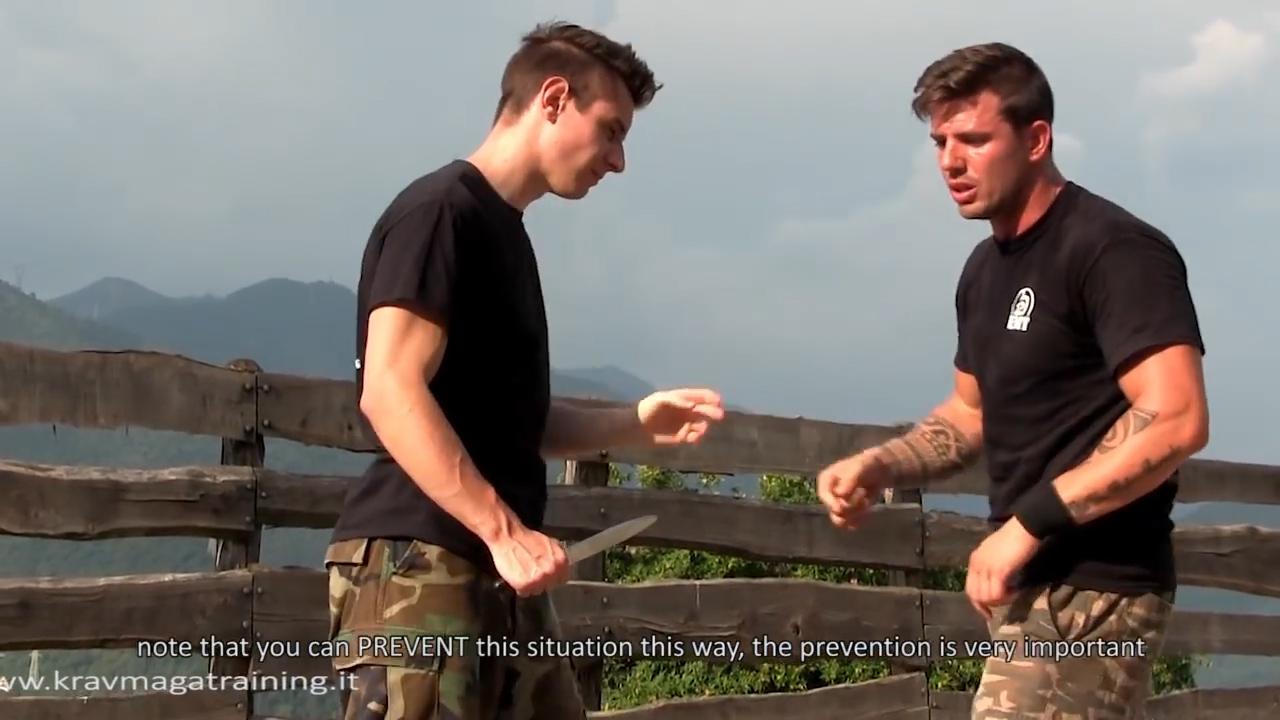

Supplement: Supplementary file 2 — Supplementary Information 2. [file 41598_2023_35190_MOESM2_ESM.zip › test/images/KravMagaTraining20803_jpg.rf.8275344347c4c78174cdb582eb49ea16.jpg]

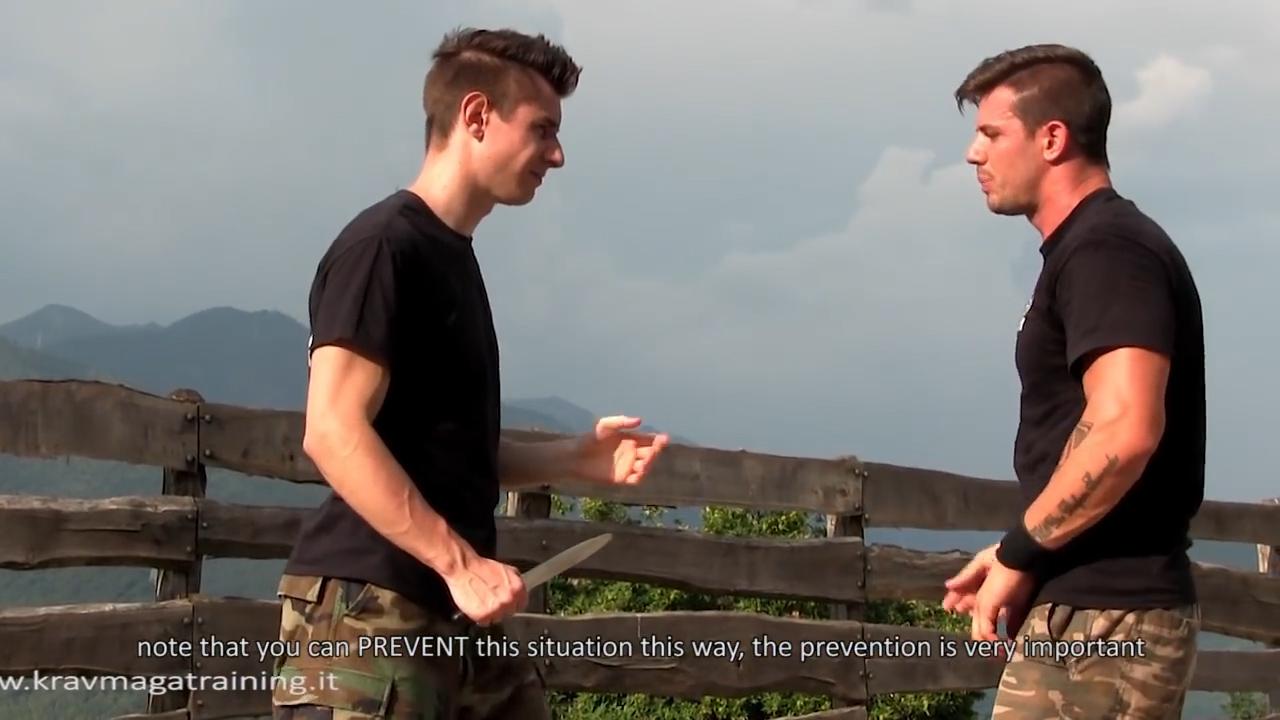

Supplement: Supplementary file 2 — Supplementary Information 2. [file 41598_2023_35190_MOESM2_ESM.zip › test/images/KravMagaTraining20809_jpg.rf.07b38c95b776efa9f152851f194bf5db.jpg]

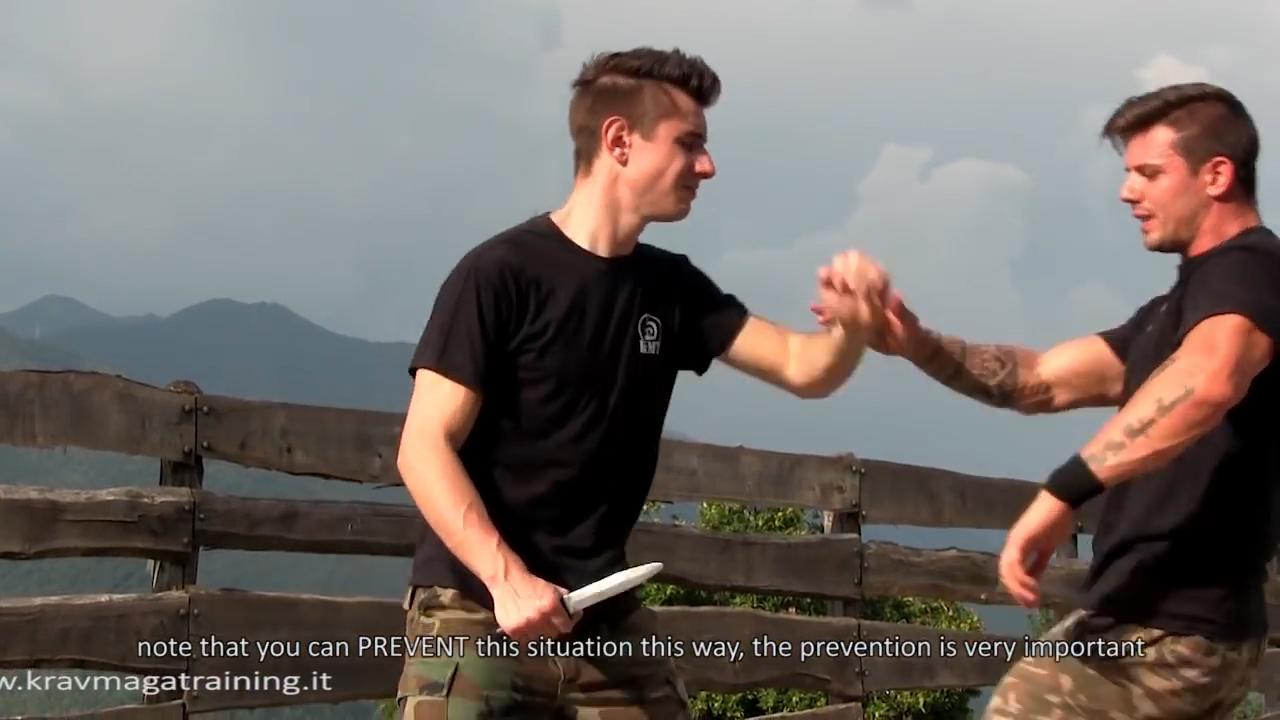

Supplement: Supplementary file 2 — Supplementary Information 2. [file 41598_2023_35190_MOESM2_ESM.zip › test/images/KravMagaTraining20811_jpg.rf.0a49e5b3f912e4ecc0073bcc959cfe50.jpg]

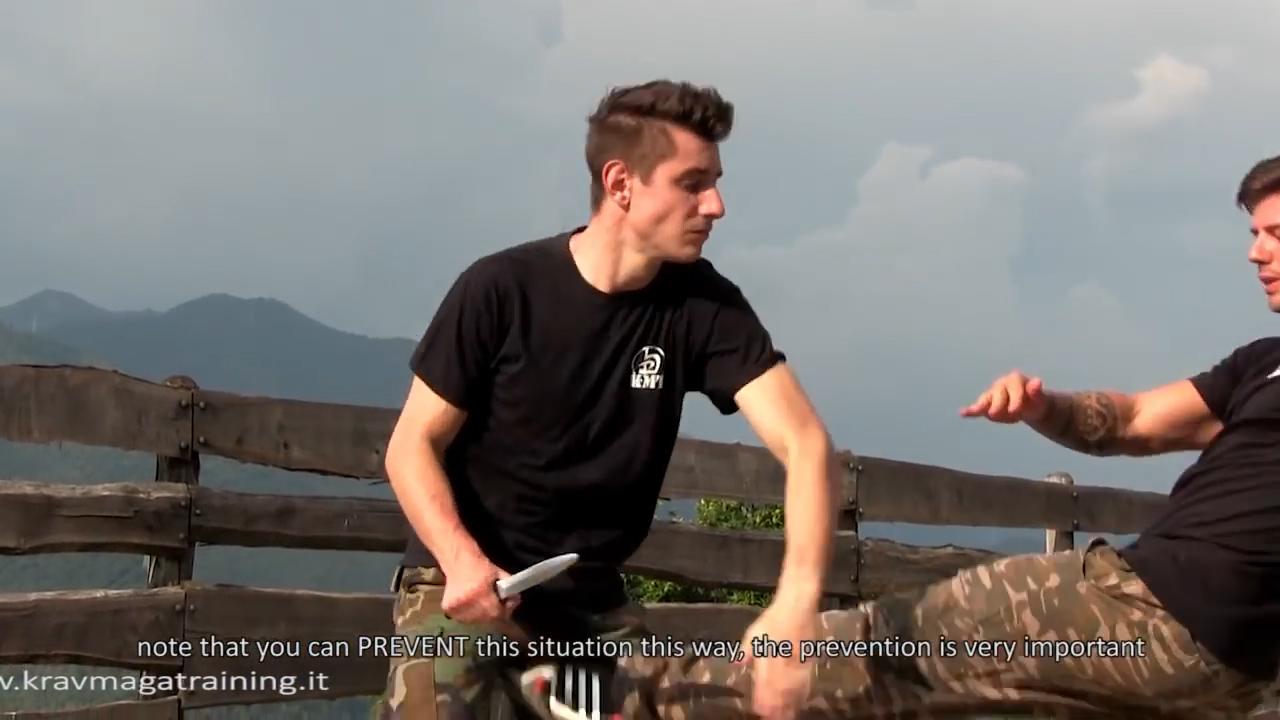

Supplement: Supplementary file 2 — Supplementary Information 2. [file 41598_2023_35190_MOESM2_ESM.zip › test/images/KravMagaTraining20812_jpg.rf.f60c3a38b924307211067359b2c703b3.jpg]

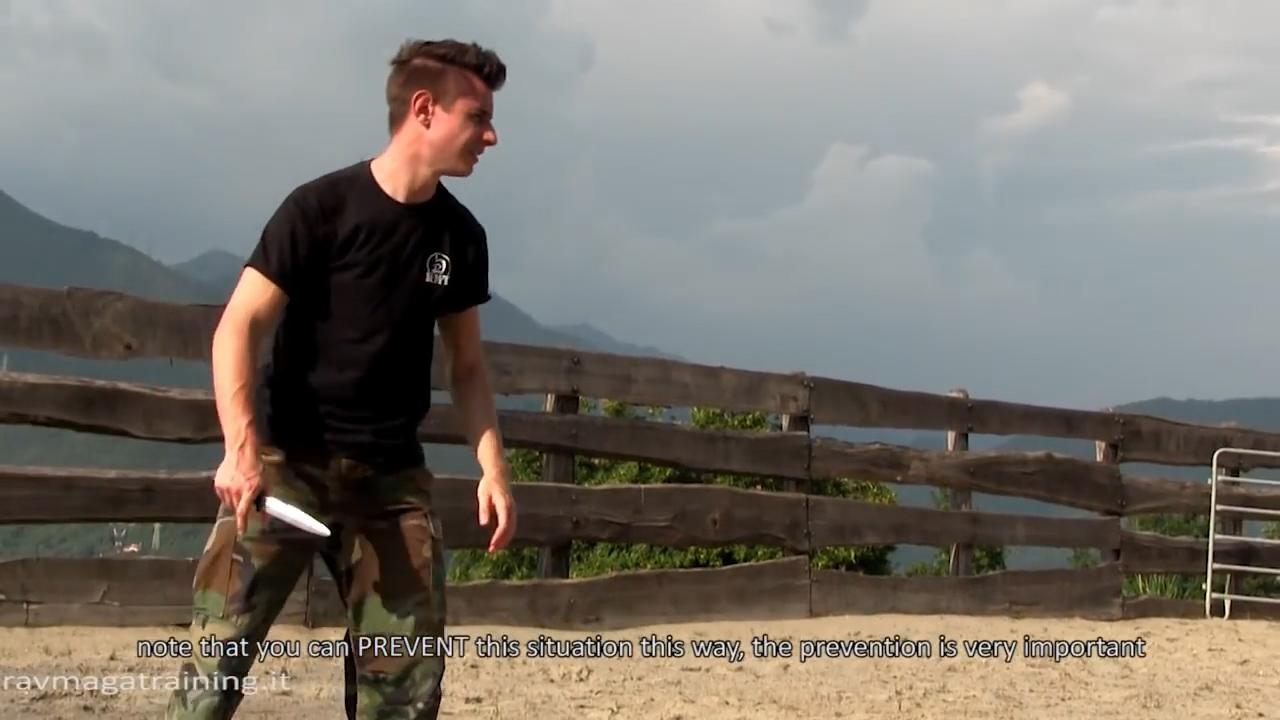

Supplement: Supplementary file 2 — Supplementary Information 2. [file 41598_2023_35190_MOESM2_ESM.zip › test/images/KravMagaTraining20823_jpg.rf.33ca0da7793ba8939ba4c3da605a67d1.jpg]

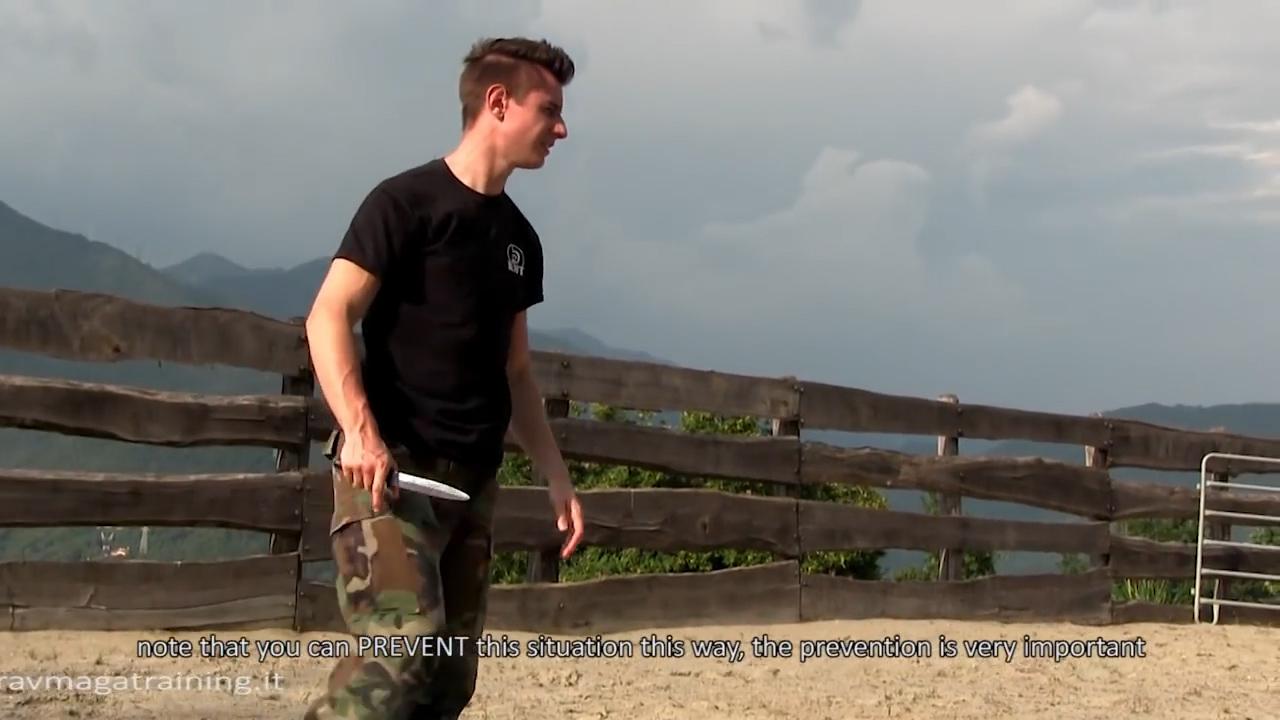

Supplement: Supplementary file 2 — Supplementary Information 2. [file 41598_2023_35190_MOESM2_ESM.zip › test/images/KravMagaTraining20825_jpg.rf.bf3d4fb14e46a98a5cebd5f0363a8b4e.jpg]

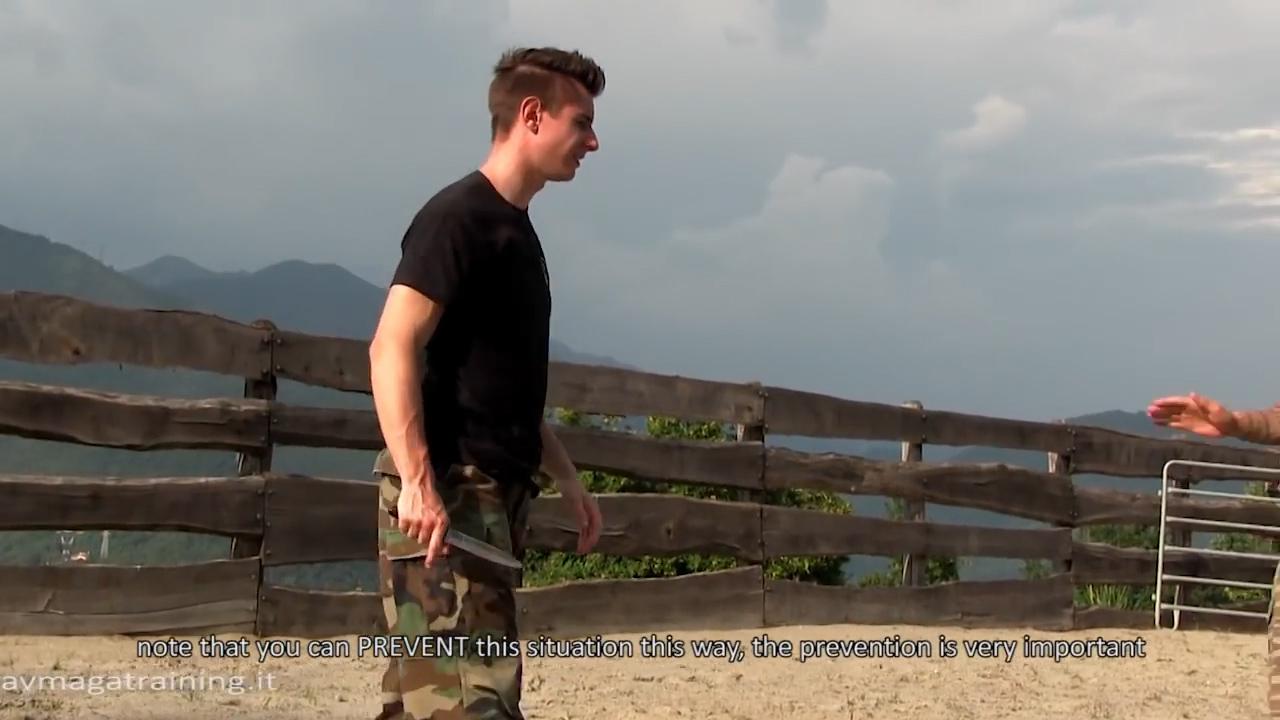

Supplement: Supplementary file 2 — Supplementary Information 2. [file 41598_2023_35190_MOESM2_ESM.zip › test/images/KravMagaTraining20827_jpg.rf.2b24e797166ef6357462bef4a3c817df.jpg]

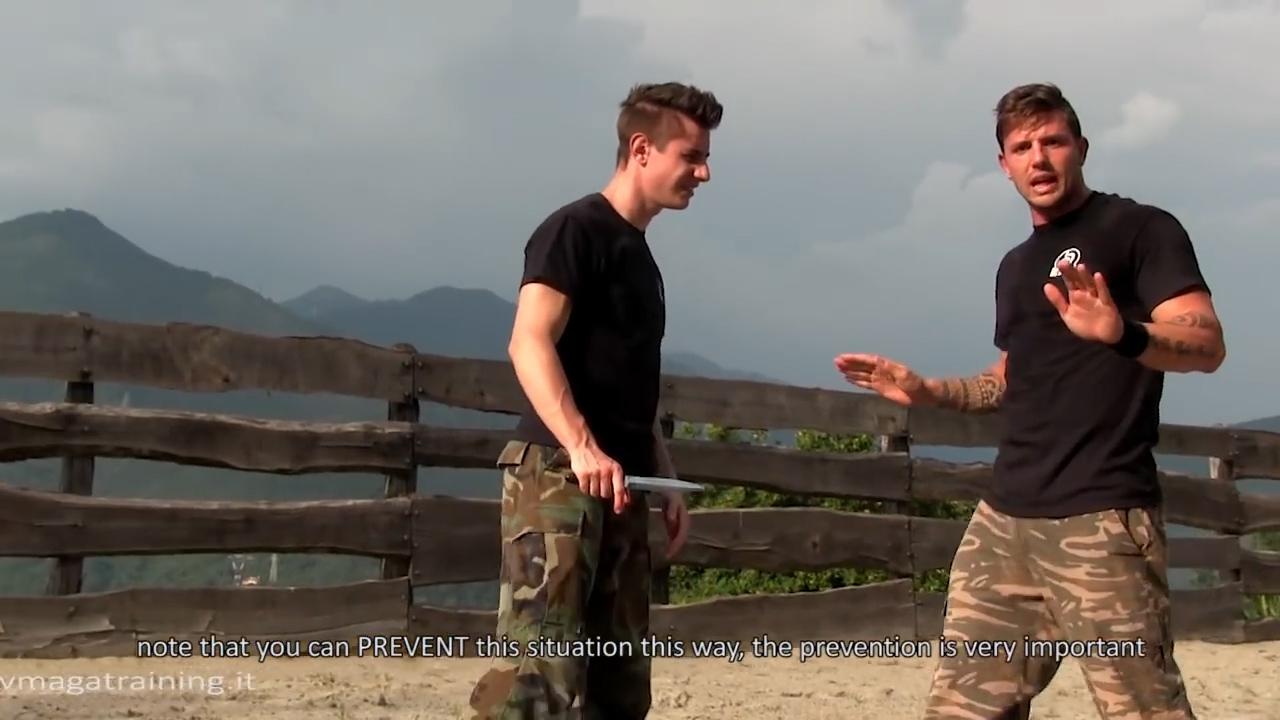

Supplement: Supplementary file 2 — Supplementary Information 2. [file 41598_2023_35190_MOESM2_ESM.zip › test/images/KravMagaTraining20833_jpg.rf.3bb8bfde3f2cb1072b78c82d48f0c558.jpg]

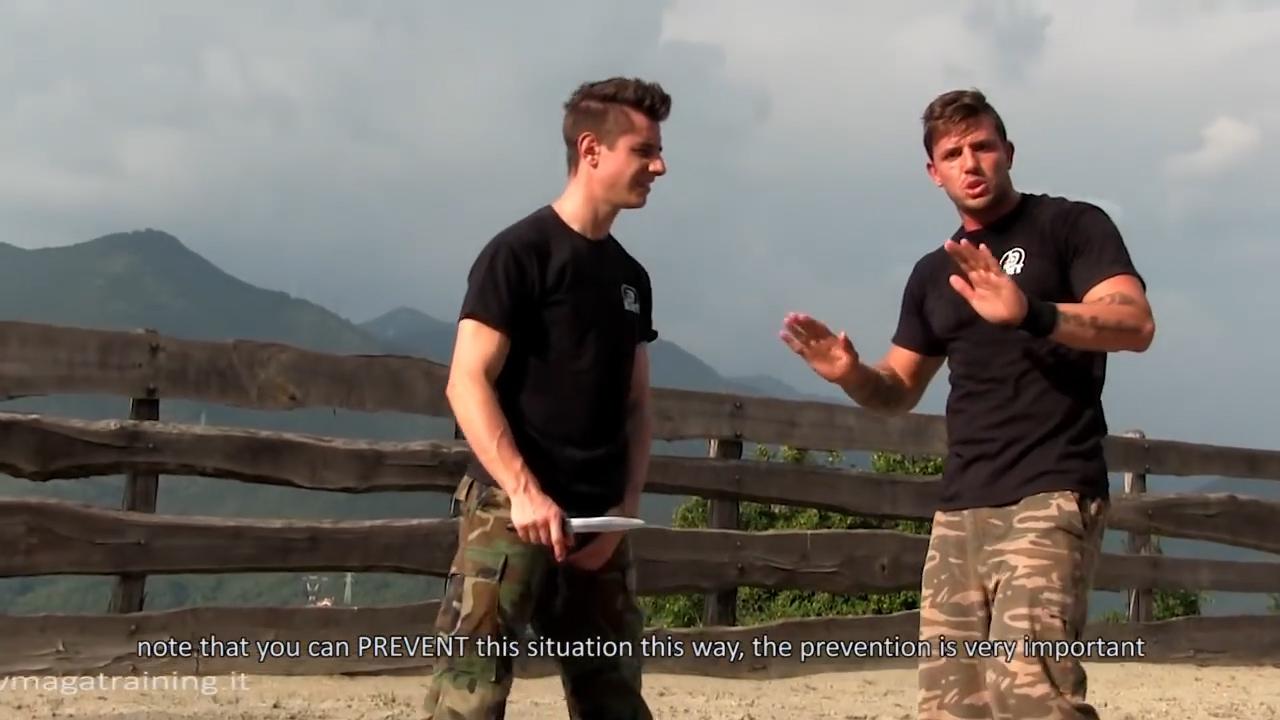

Supplement: Supplementary file 2 — Supplementary Information 2. [file 41598_2023_35190_MOESM2_ESM.zip › test/images/KravMagaTraining20835_jpg.rf.83d99523dd164e5e6ddf55fe14d07e20.jpg]

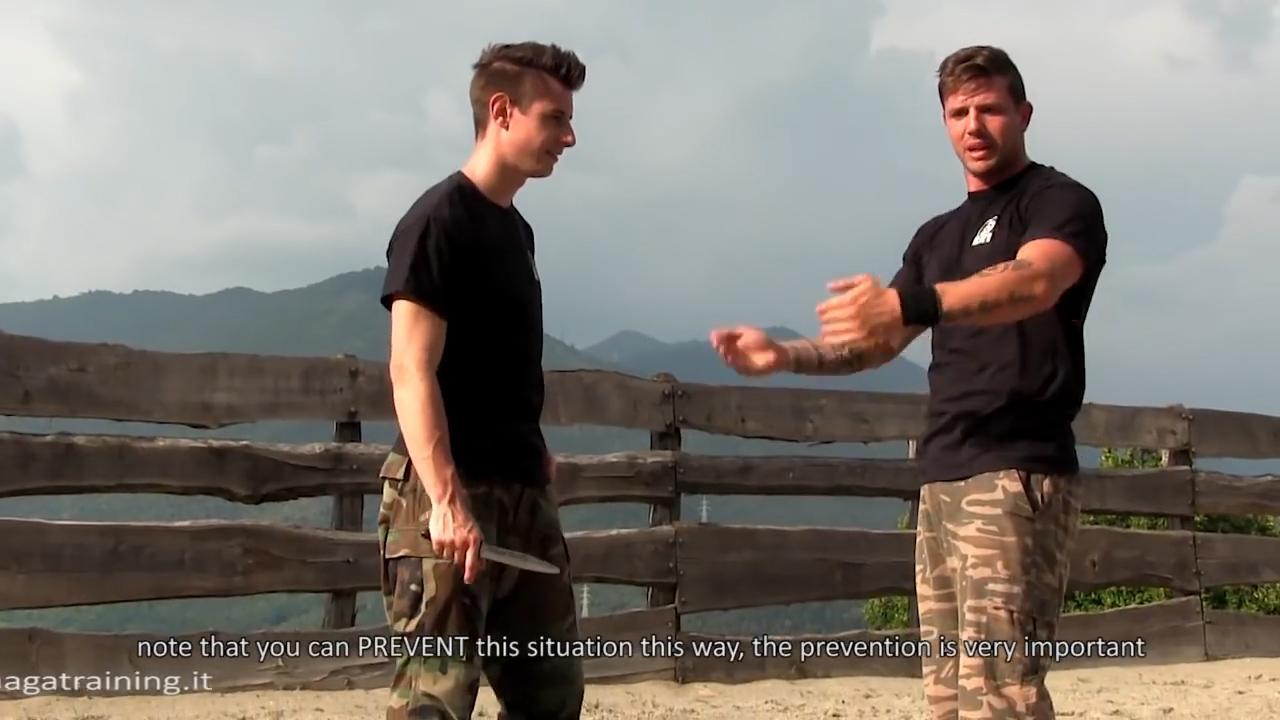

Supplement: Supplementary file 2 — Supplementary Information 2. [file 41598_2023_35190_MOESM2_ESM.zip › test/images/KravMagaTraining20846_jpg.rf.155cb627cd9450cd9823eed9c5593a16.jpg]

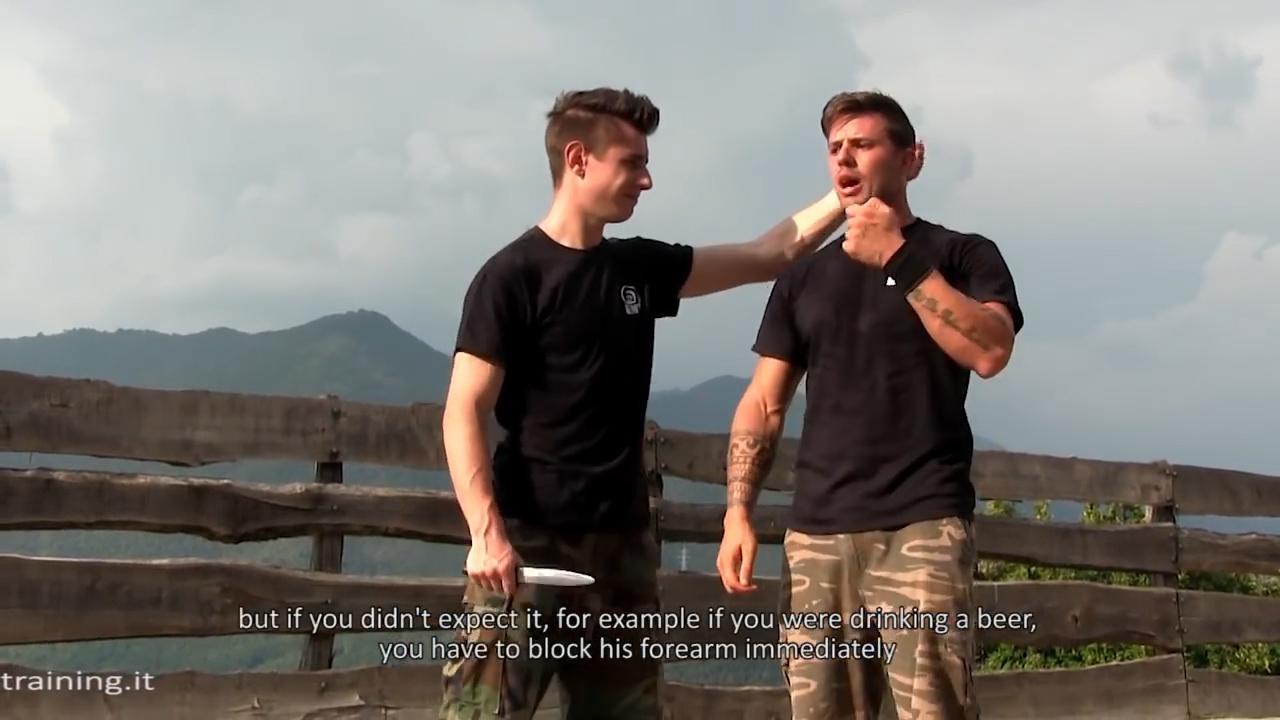

Supplement: Supplementary file 2 — Supplementary Information 2. [file 41598_2023_35190_MOESM2_ESM.zip › test/images/KravMagaTraining20863_jpg.rf.9e3a80f77afbc89b4933060d80827aa3.jpg]

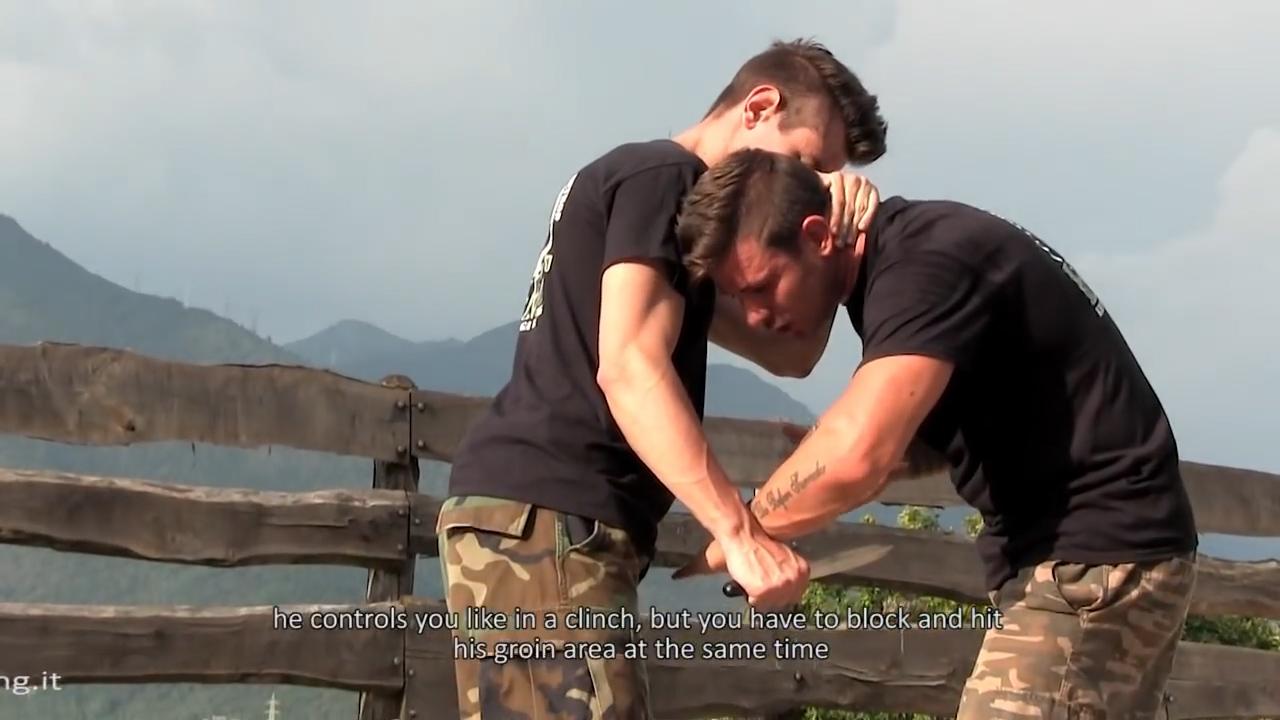

Supplement: Supplementary file 2 — Supplementary Information 2. [file 41598_2023_35190_MOESM2_ESM.zip › test/images/KravMagaTraining20890_jpg.rf.5cbf5925c799f9bec44f747ecde42adc.jpg]

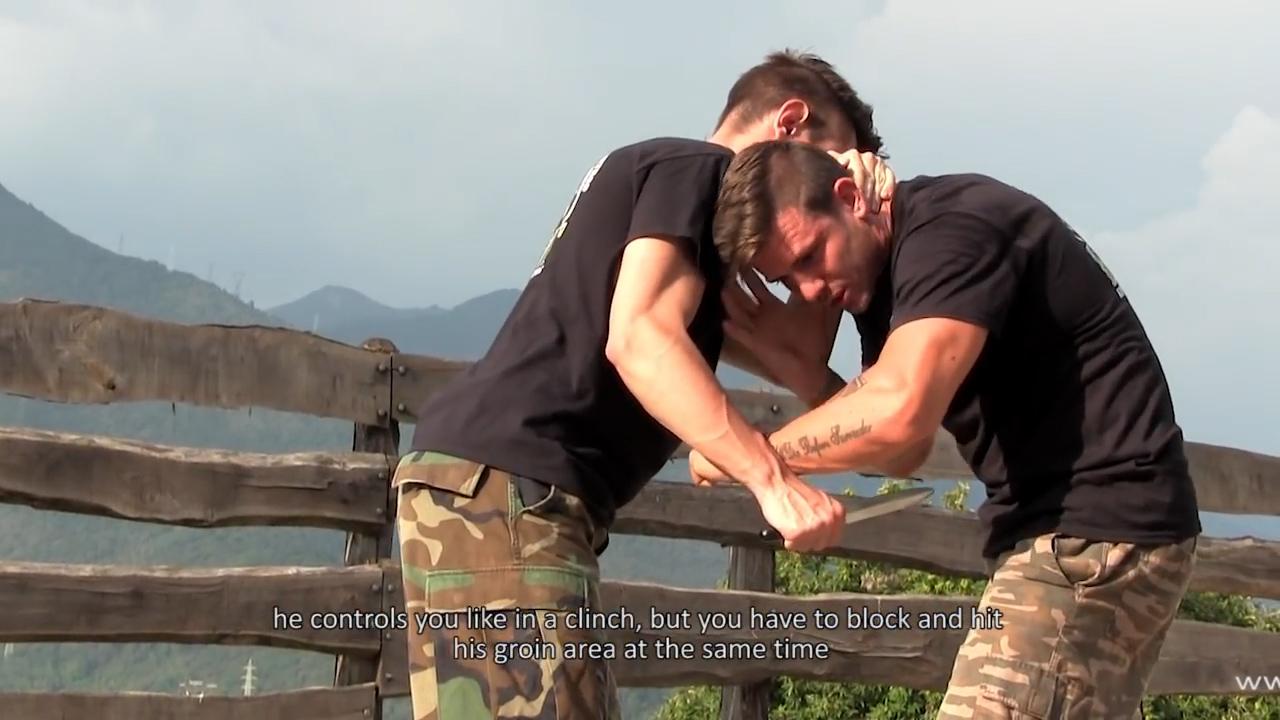

Supplement: Supplementary file 2 — Supplementary Information 2. [file 41598_2023_35190_MOESM2_ESM.zip › test/images/KravMagaTraining20921_jpg.rf.4109e68207b5b80afb53e0632e37827e.jpg]

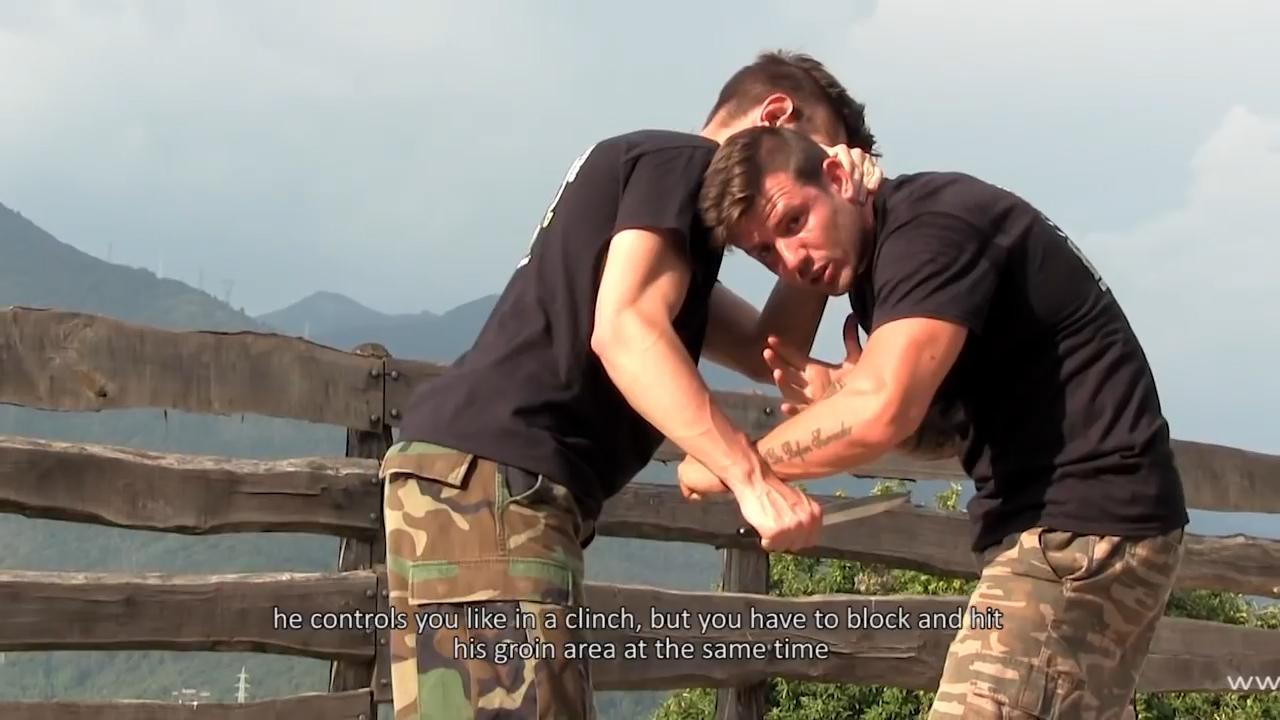

Supplement: Supplementary file 2 — Supplementary Information 2. [file 41598_2023_35190_MOESM2_ESM.zip › test/images/KravMagaTraining20924_jpg.rf.e05f19cc042145b0a1c92987ca675bfb.jpg]

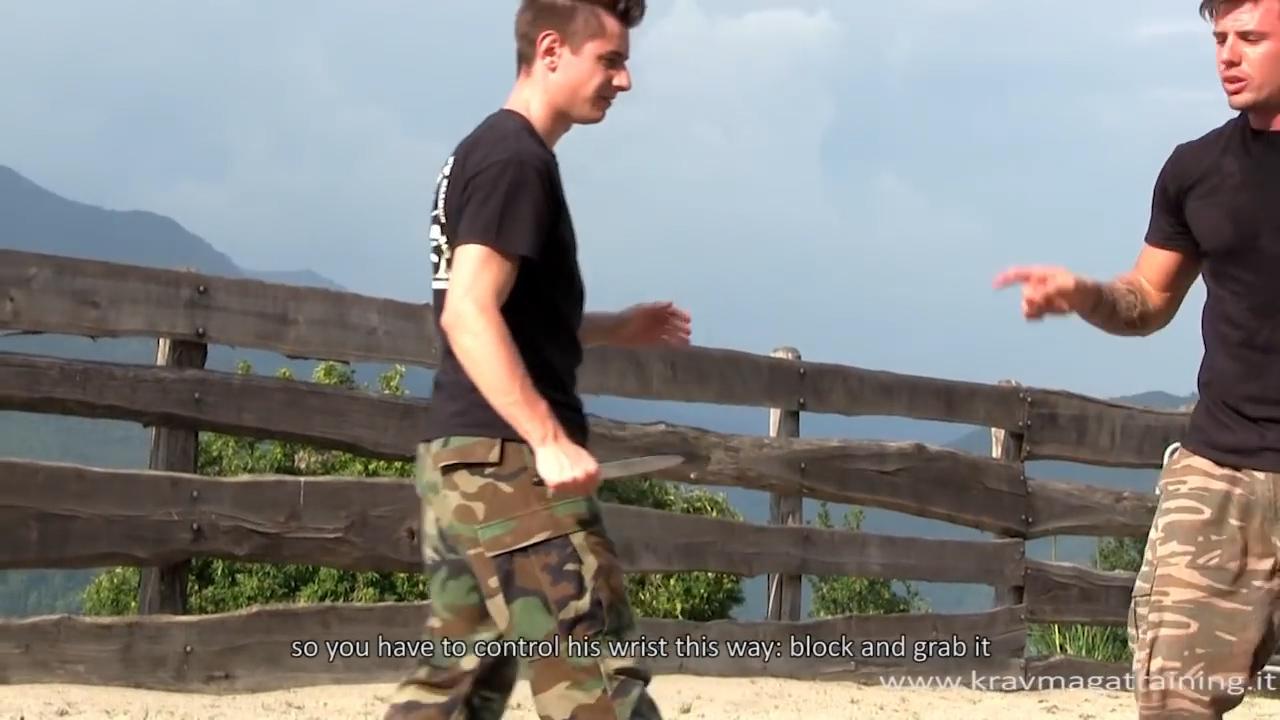

Supplement: Supplementary file 2 — Supplementary Information 2. [file 41598_2023_35190_MOESM2_ESM.zip › test/images/KravMagaTraining20967_jpg.rf.8548e3cf31348013188d874102341a24.jpg]

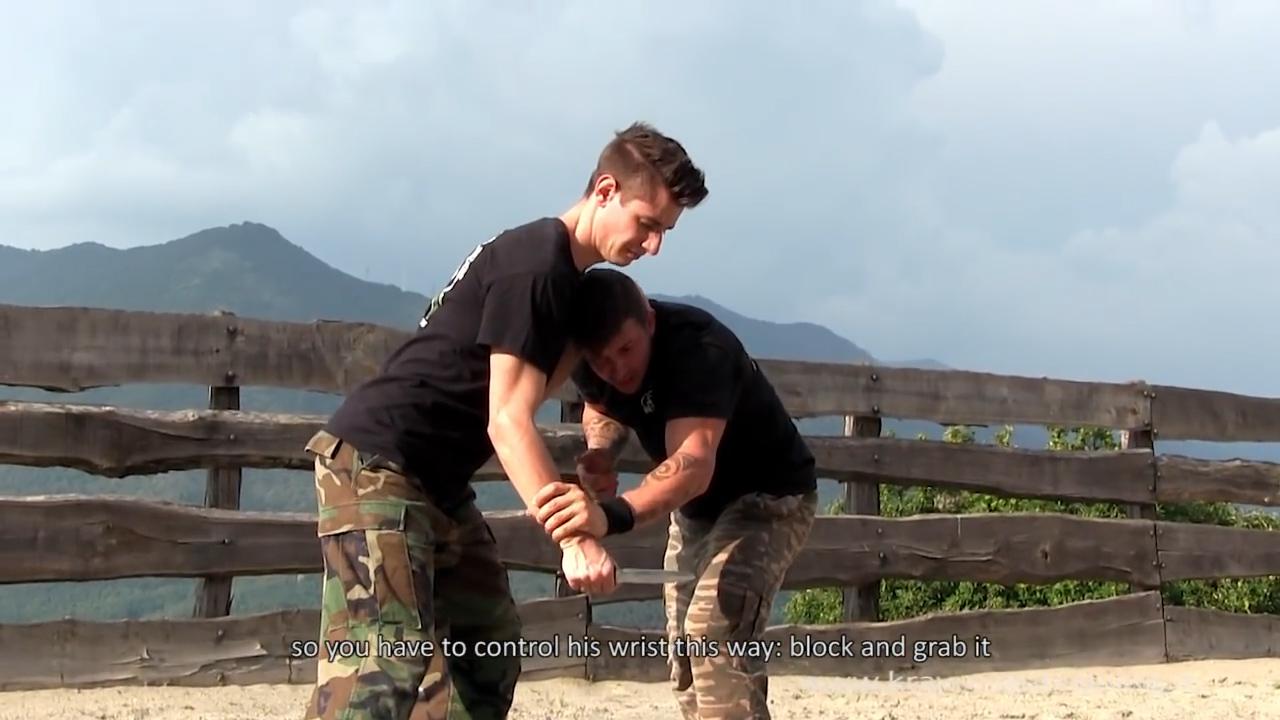

Supplement: Supplementary file 2 — Supplementary Information 2. [file 41598_2023_35190_MOESM2_ESM.zip › test/images/KravMagaTraining20997_jpg.rf.9b558b34c88d5f94df82be9309b0dfcf.jpg]

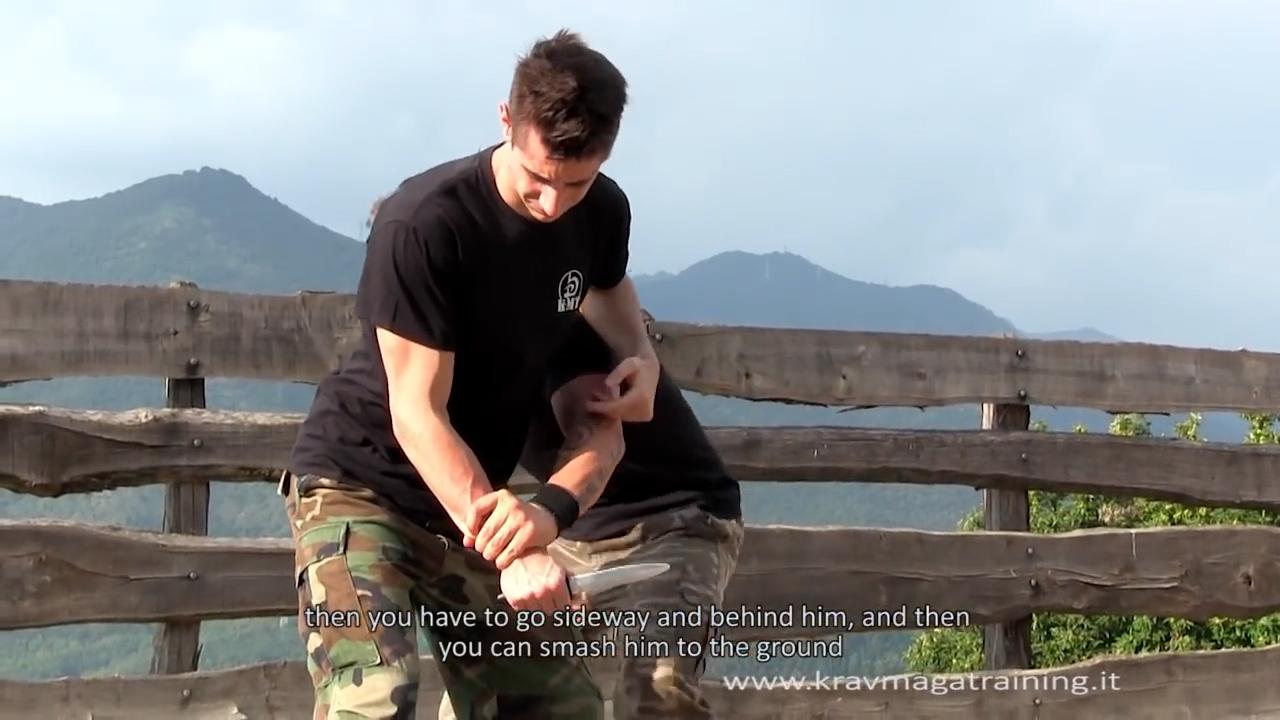

Supplement: Supplementary file 2 — Supplementary Information 2. [file 41598_2023_35190_MOESM2_ESM.zip › test/images/KravMagaTraining21024_jpg.rf.5bfa6f8f74a16cac9644c8014efd4457.jpg]

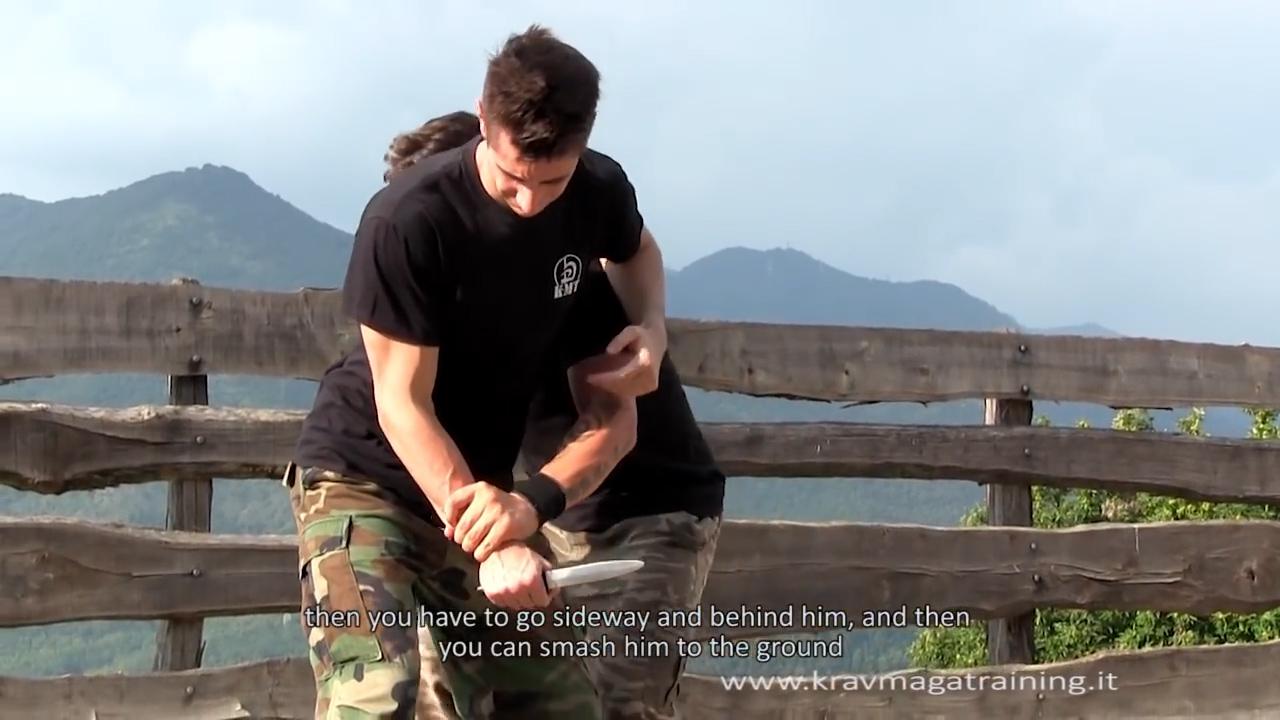

Supplement: Supplementary file 2 — Supplementary Information 2. [file 41598_2023_35190_MOESM2_ESM.zip › test/images/KravMagaTraining21025_jpg.rf.ade9c994e73df39aa50fe0be33b8d76b.jpg]

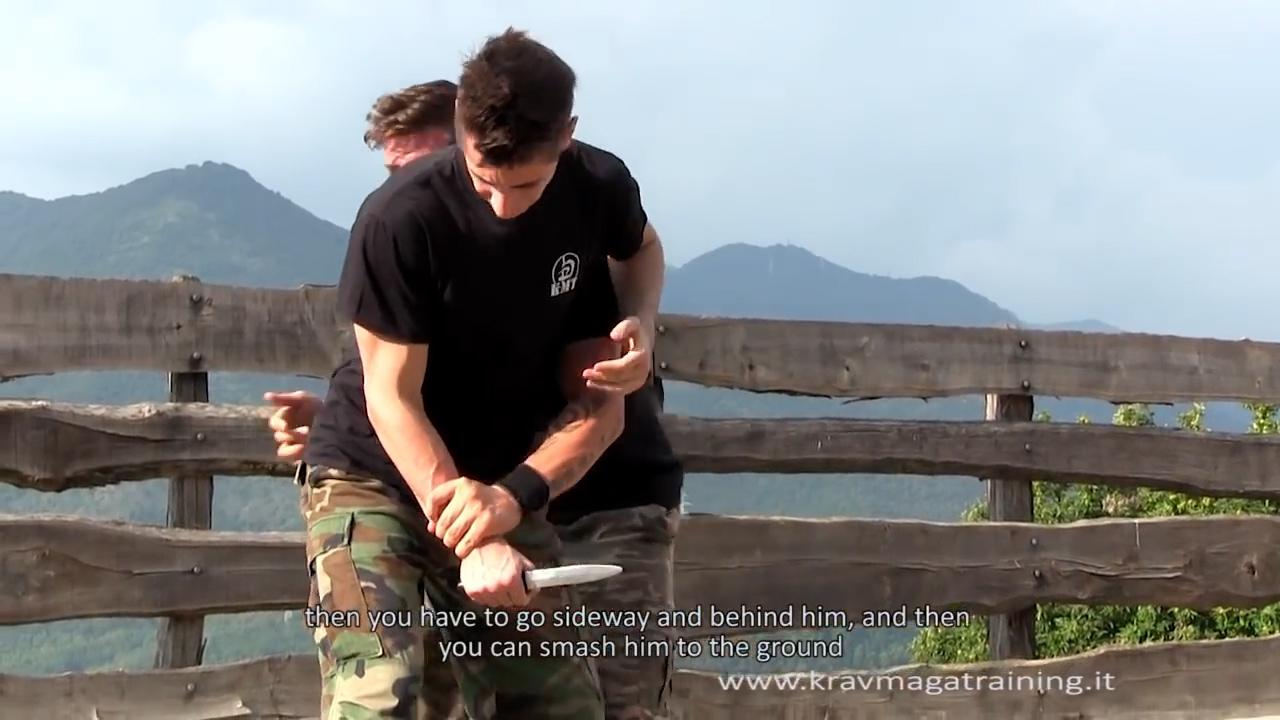

Supplement: Supplementary file 2 — Supplementary Information 2. [file 41598_2023_35190_MOESM2_ESM.zip › test/images/KravMagaTraining21026_jpg.rf.366021d378de2df7678a0de1c553b20a.jpg]

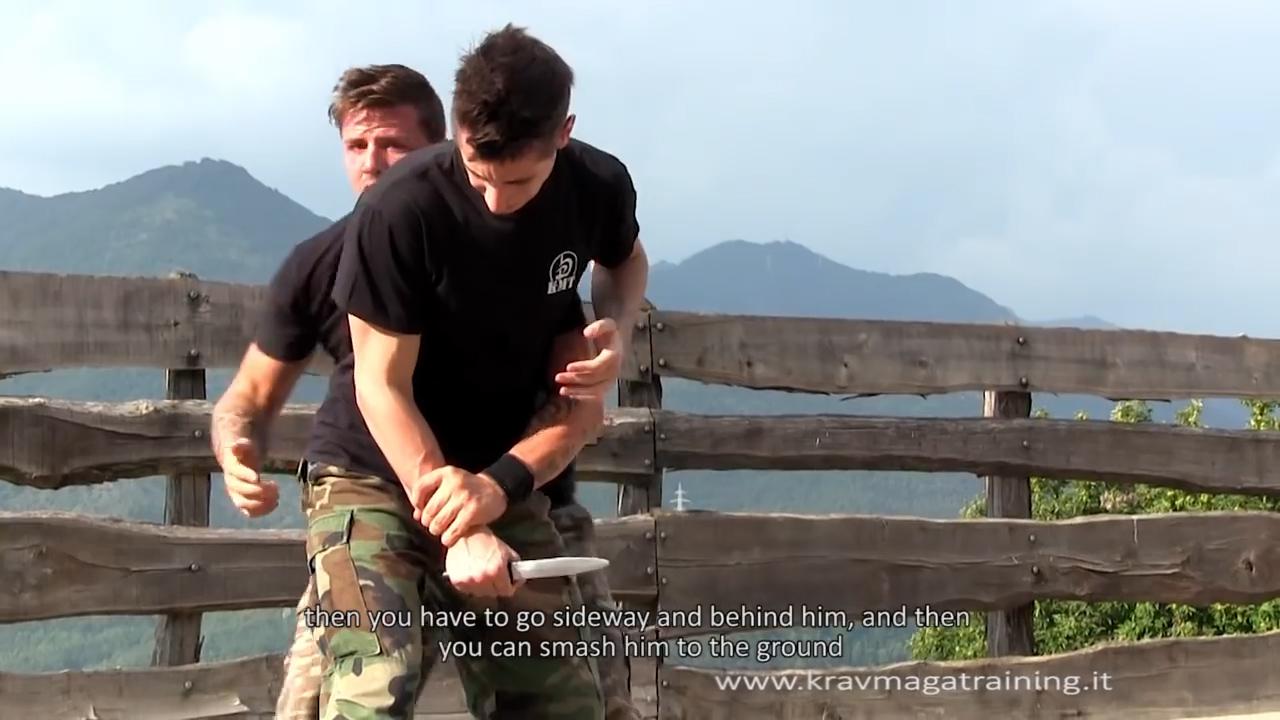

Supplement: Supplementary file 2 — Supplementary Information 2. [file 41598_2023_35190_MOESM2_ESM.zip › test/images/KravMagaTraining21027_jpg.rf.c24613e89a6570650103c0967cb49f6d.jpg]

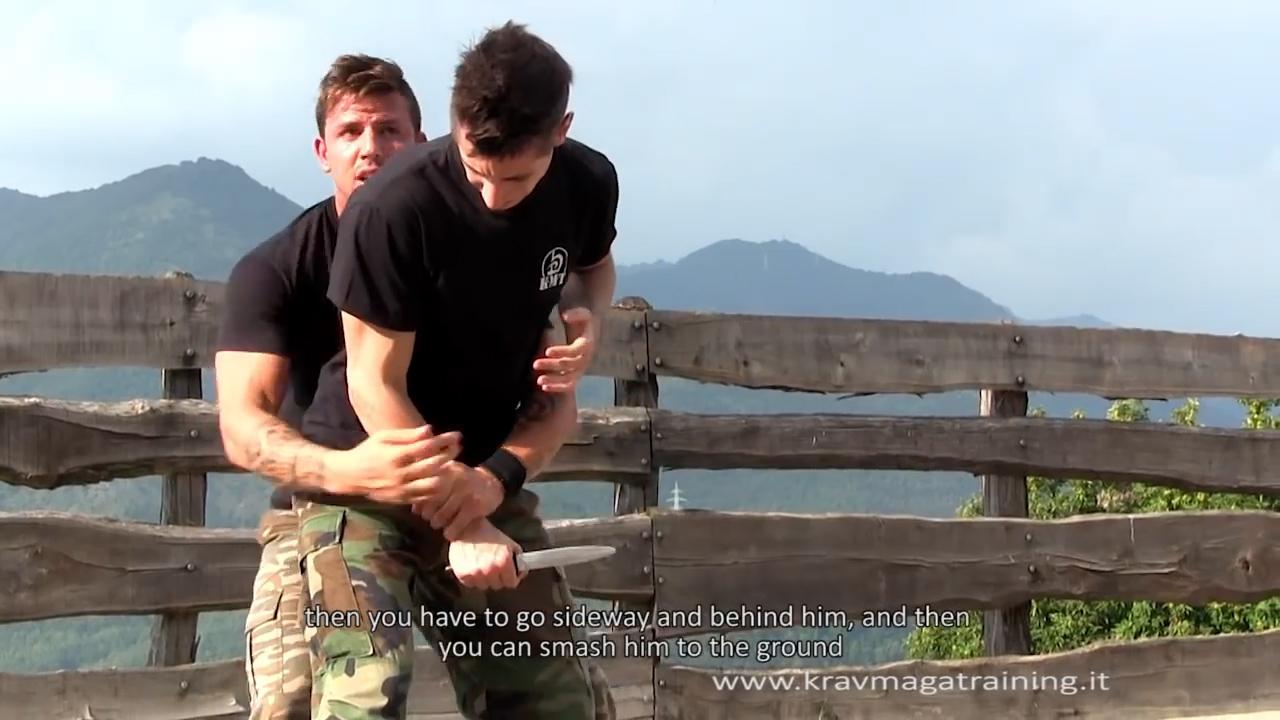

Supplement: Supplementary file 2 — Supplementary Information 2. [file 41598_2023_35190_MOESM2_ESM.zip › test/images/KravMagaTraining21028_jpg.rf.5011047263aeae4b3b0d4528f3d824c5.jpg]

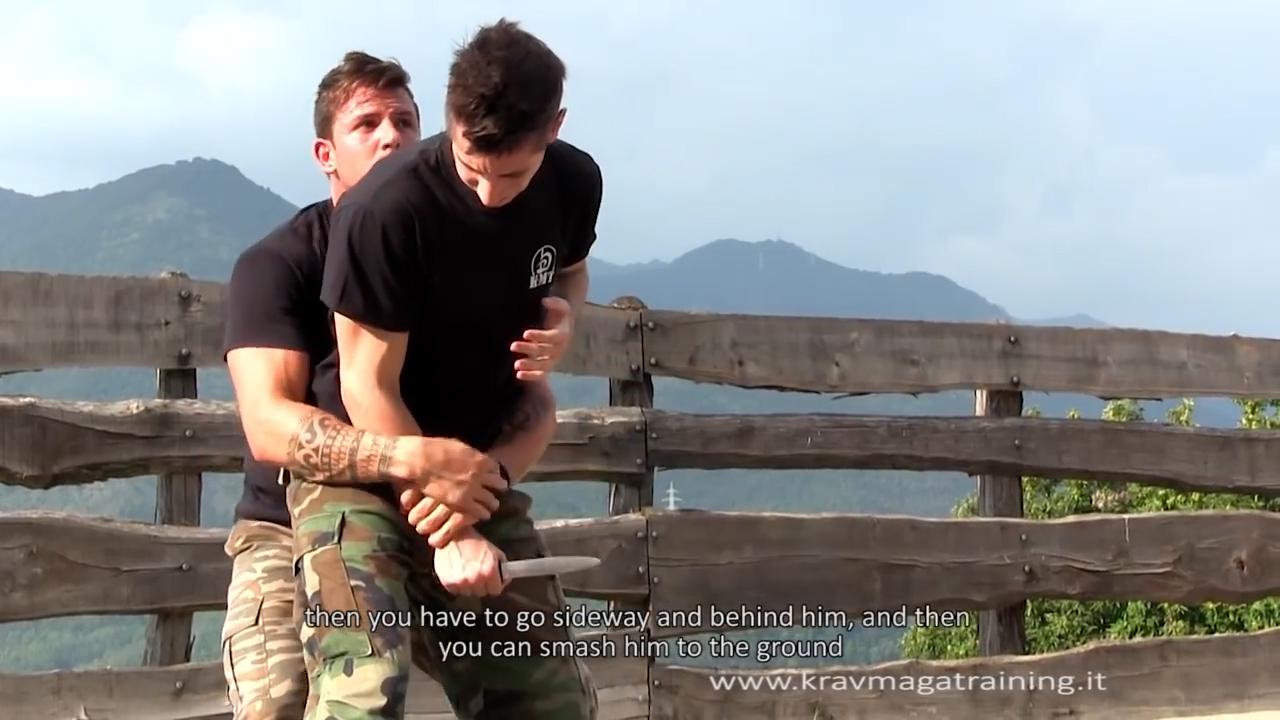

Supplement: Supplementary file 2 — Supplementary Information 2. [file 41598_2023_35190_MOESM2_ESM.zip › test/images/KravMagaTraining21029_jpg.rf.97bbe3bf81b5de9771a5b7a4b49ddb91.jpg]

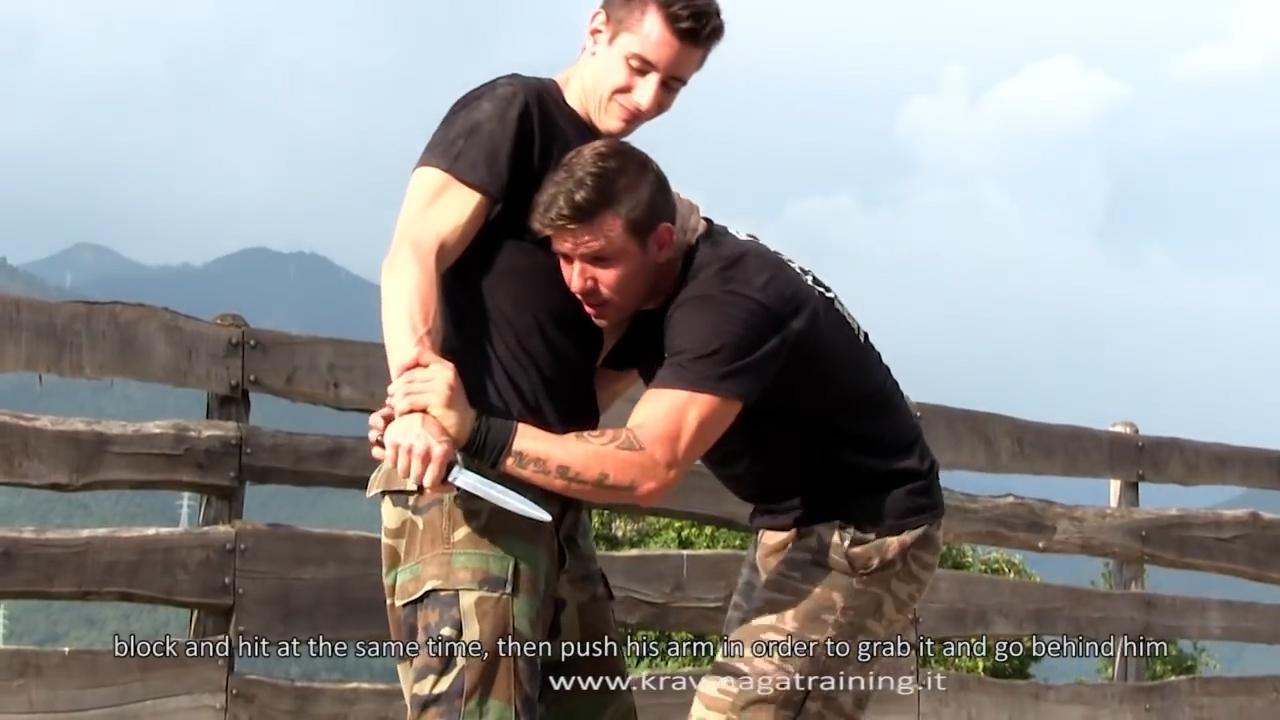

Supplement: Supplementary file 2 — Supplementary Information 2. [file 41598_2023_35190_MOESM2_ESM.zip › test/images/KravMagaTraining21066_jpg.rf.b0d9e94d879c292c445339379c5927e2.jpg]

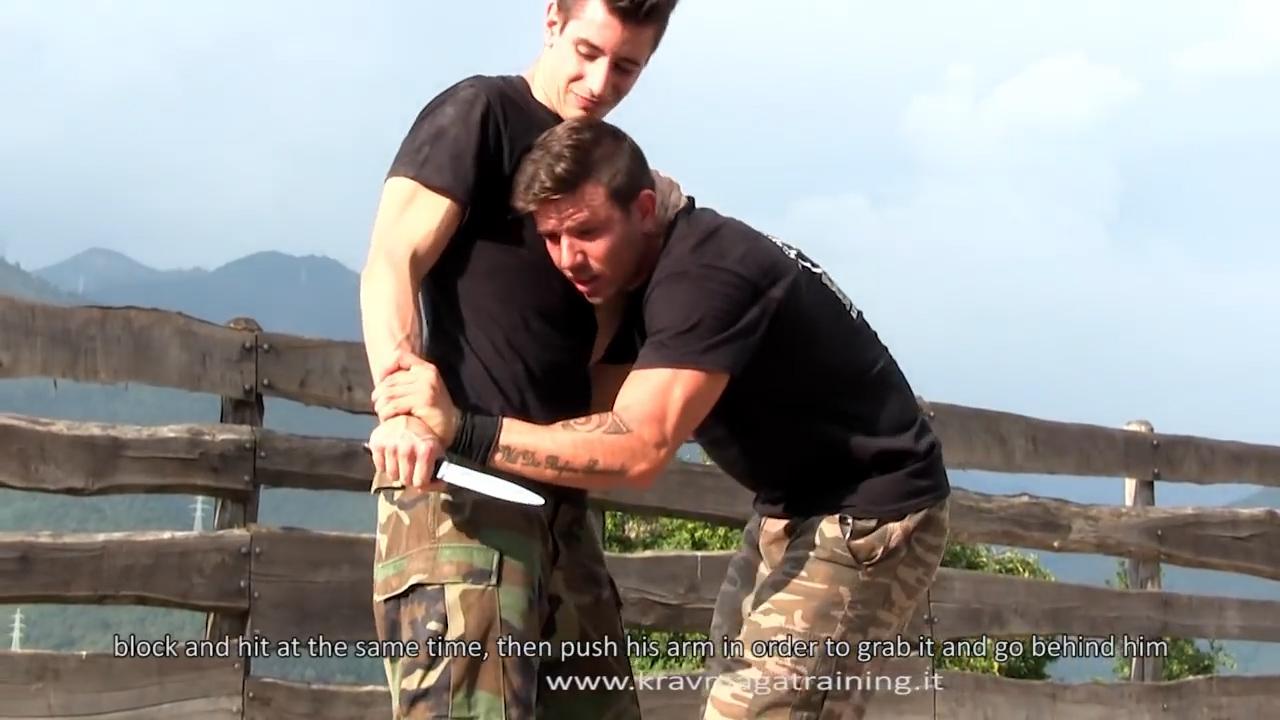

Supplement: Supplementary file 2 — Supplementary Information 2. [file 41598_2023_35190_MOESM2_ESM.zip › test/images/KravMagaTraining21067_jpg.rf.04c81d7f227ec136cb7aafc47d1ce8ac.jpg]

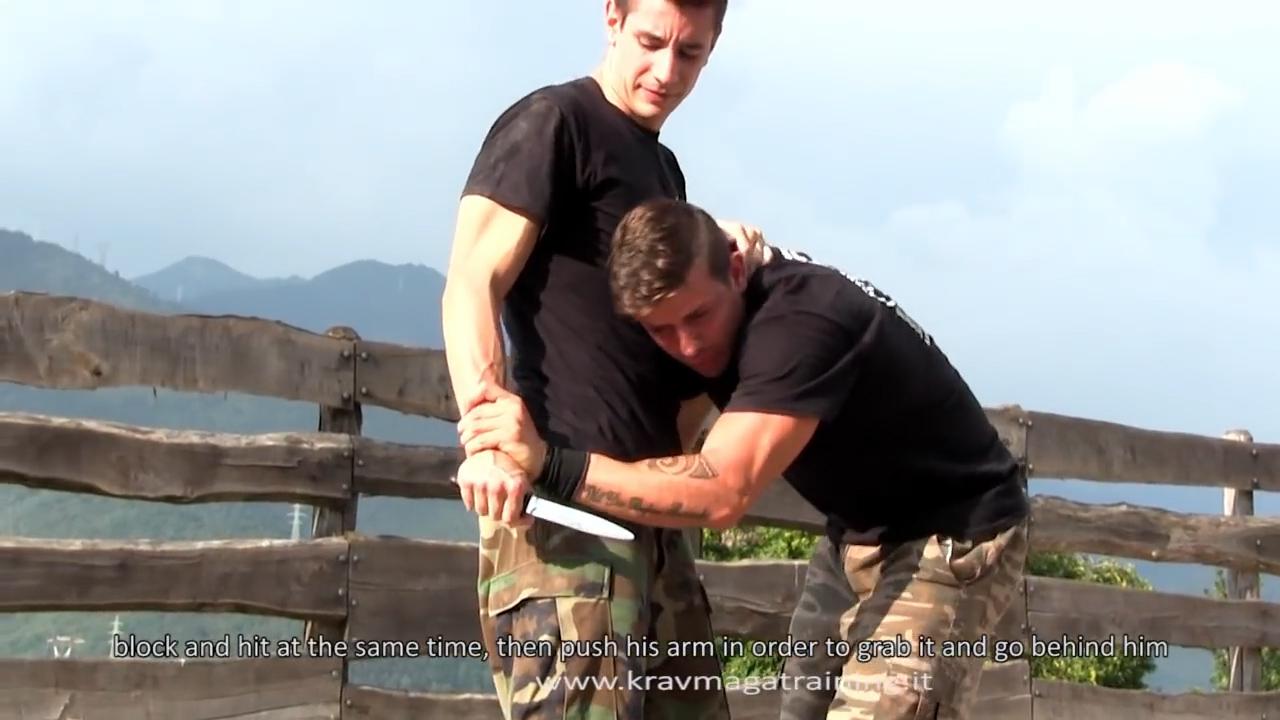

Supplement: Supplementary file 2 — Supplementary Information 2. [file 41598_2023_35190_MOESM2_ESM.zip › test/images/KravMagaTraining21071_jpg.rf.5c1358d1e64333026b2678b8e5b31b0e.jpg]

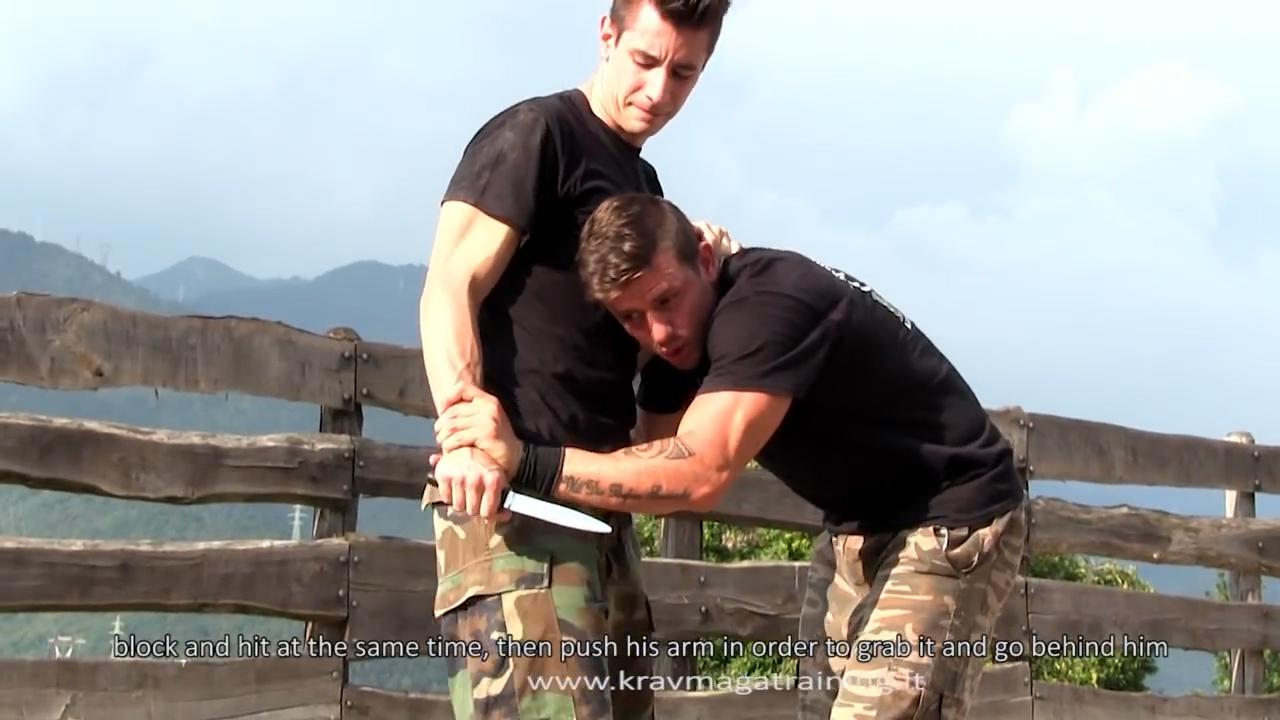

Supplement: Supplementary file 2 — Supplementary Information 2. [file 41598_2023_35190_MOESM2_ESM.zip › test/images/KravMagaTraining21074_jpg.rf.dd423e428f0dae9ac1d2e1f647121cb1.jpg]

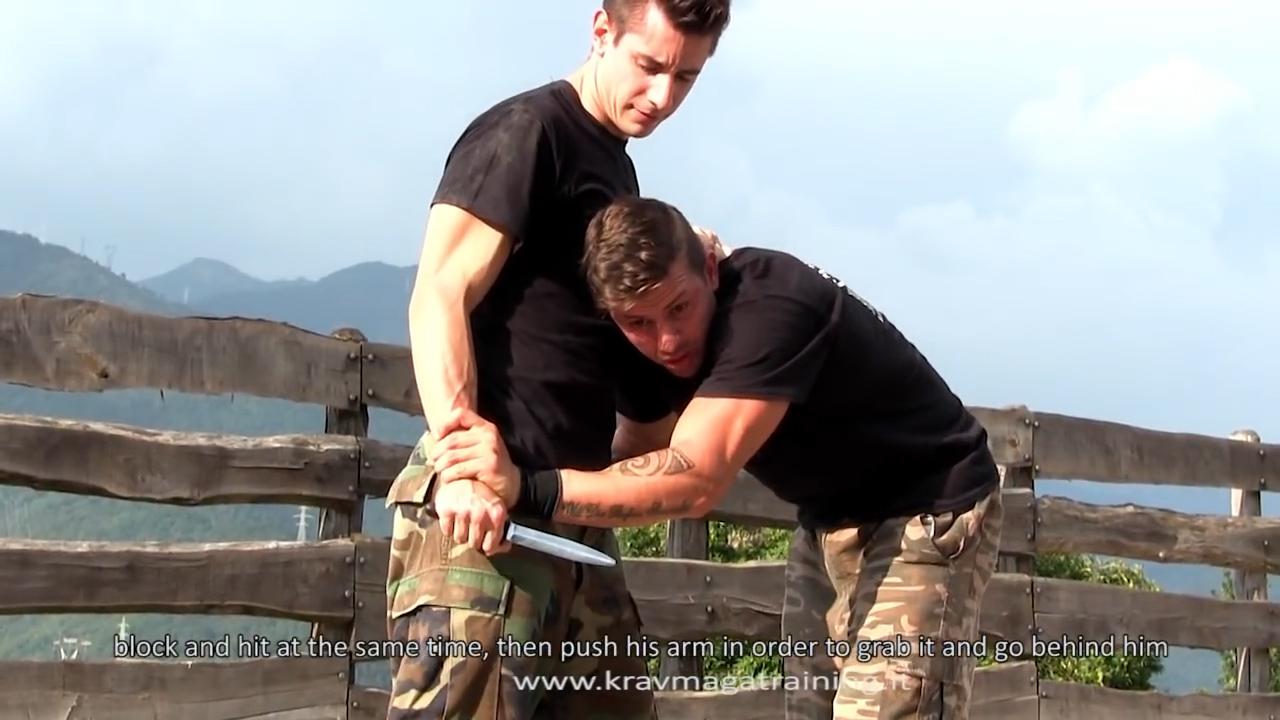

Supplement: Supplementary file 2 — Supplementary Information 2. [file 41598_2023_35190_MOESM2_ESM.zip › test/images/KravMagaTraining21079_jpg.rf.960900e42d6dae406e423378cf932c48.jpg]

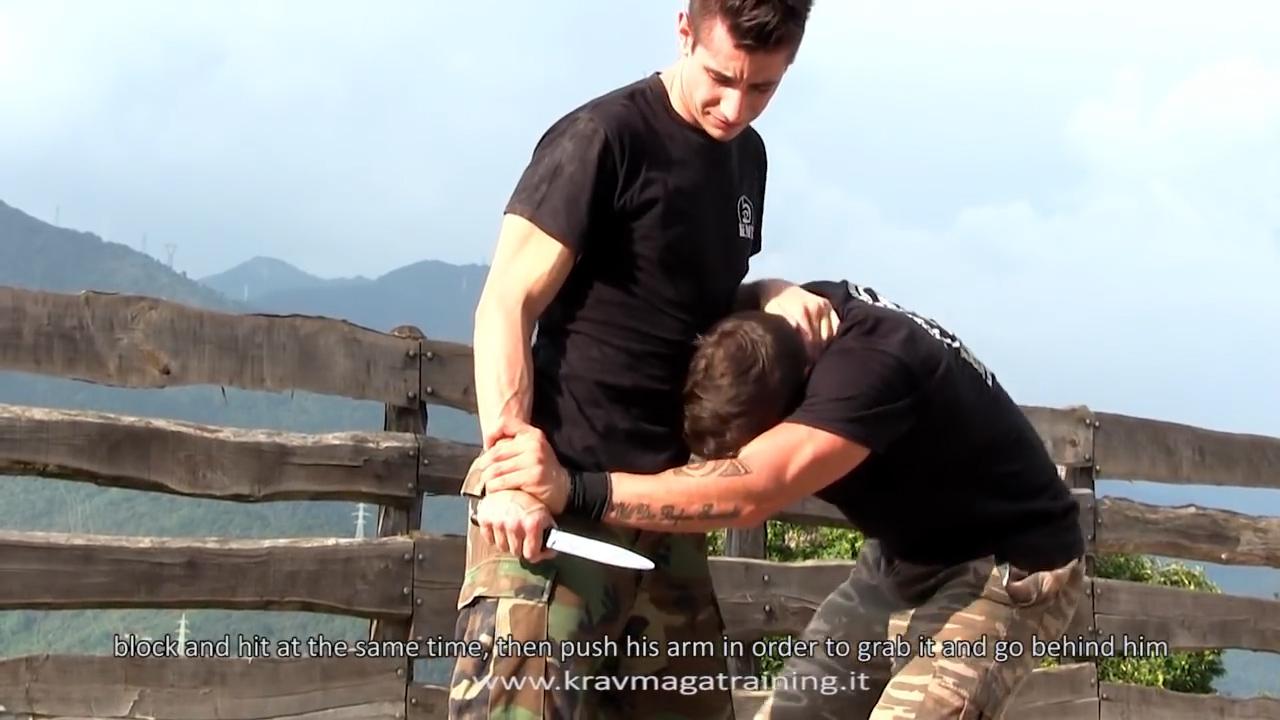

Supplement: Supplementary file 2 — Supplementary Information 2. [file 41598_2023_35190_MOESM2_ESM.zip › test/images/KravMagaTraining21094_jpg.rf.5fd36f2dfb11c952faa3f21701ce57b4.jpg]

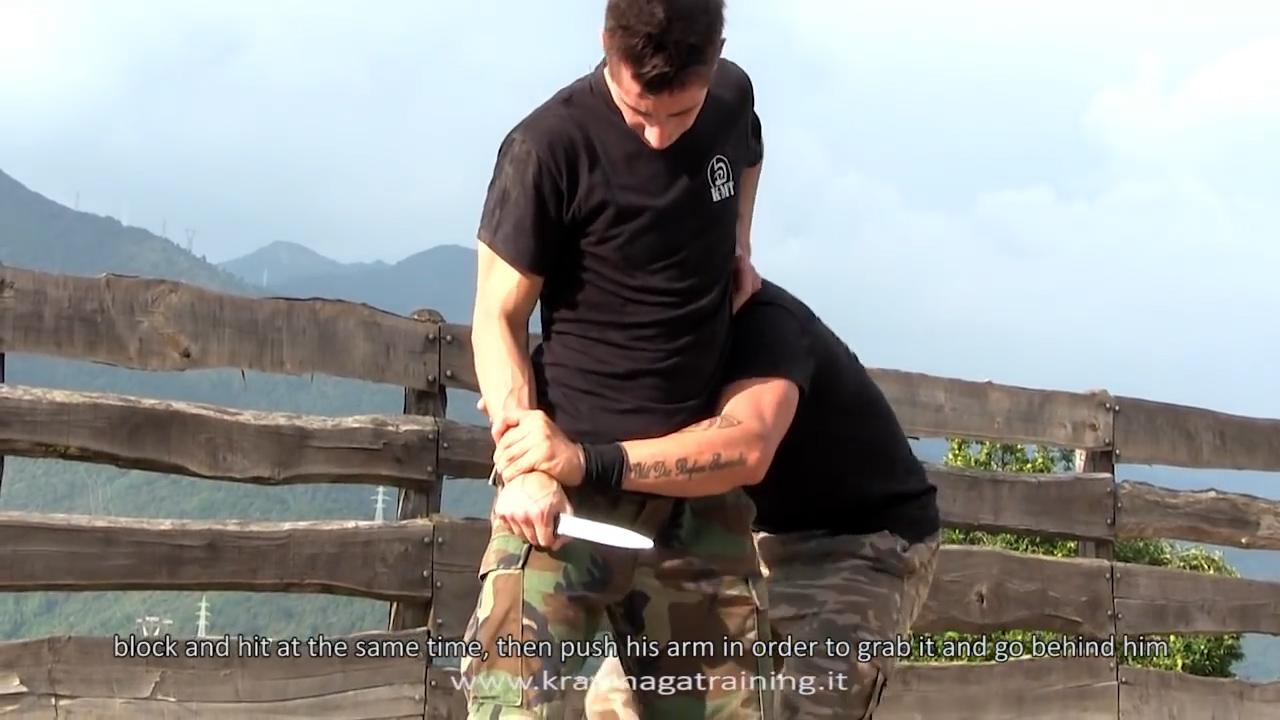

Supplement: Supplementary file 2 — Supplementary Information 2. [file 41598_2023_35190_MOESM2_ESM.zip › test/images/KravMagaTraining21102_jpg.rf.477c112edfbe48dc6f74d2fa239338cb.jpg]

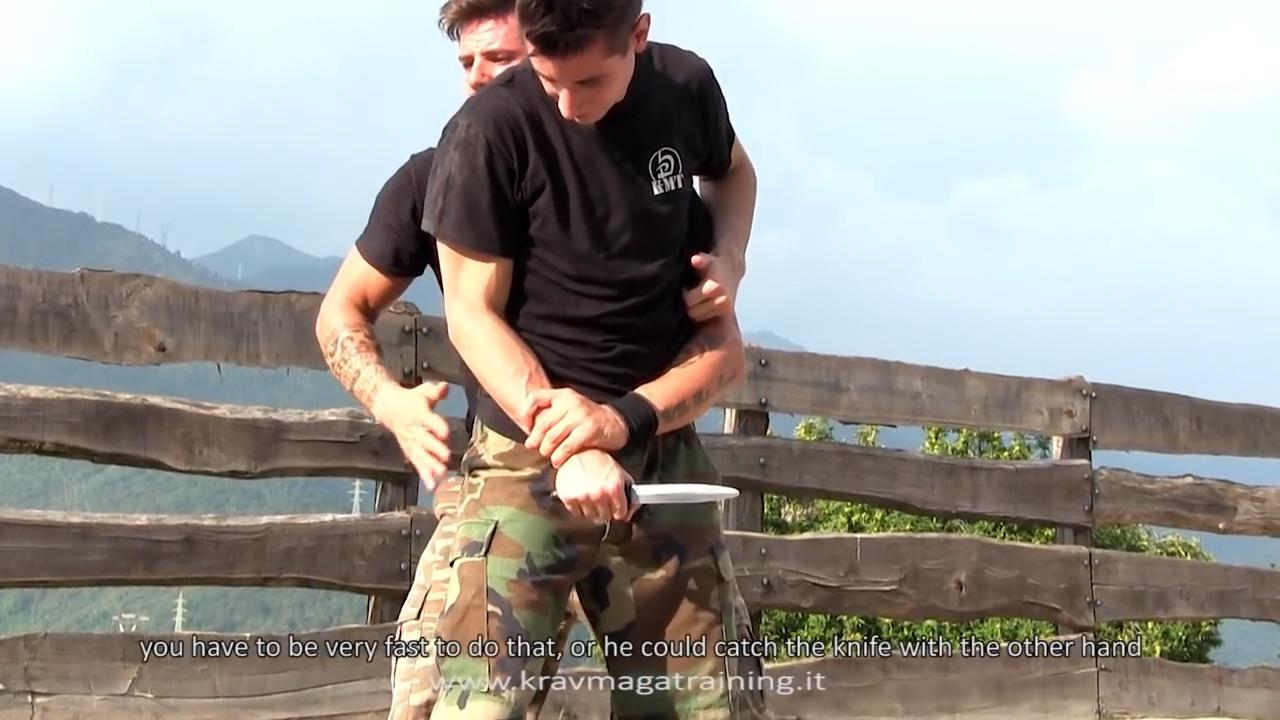

Supplement: Supplementary file 2 — Supplementary Information 2. [file 41598_2023_35190_MOESM2_ESM.zip › test/images/KravMagaTraining21110_jpg.rf.530cfed675c987a7ddb211af5c8596f7.jpg]

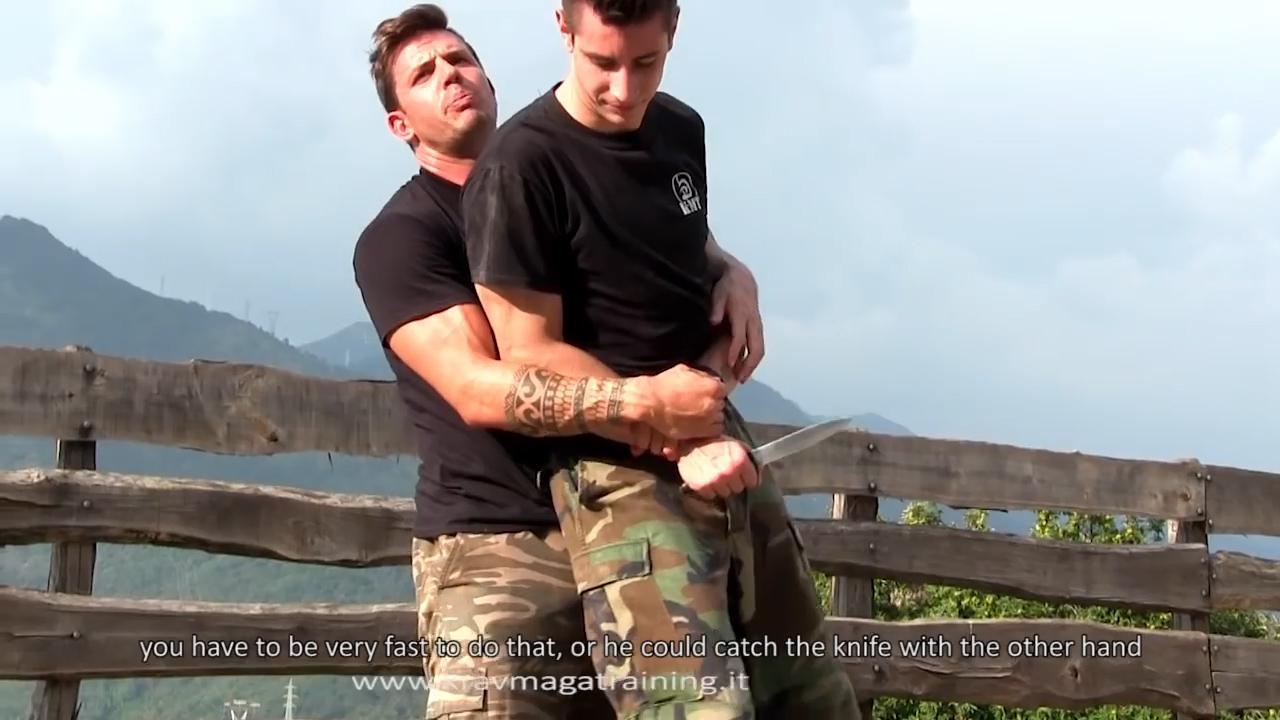

Supplement: Supplementary file 2 — Supplementary Information 2. [file 41598_2023_35190_MOESM2_ESM.zip › test/images/KravMagaTraining21138_jpg.rf.3fd5cbee3db6b8b6df06a906d80b5b7a.jpg]

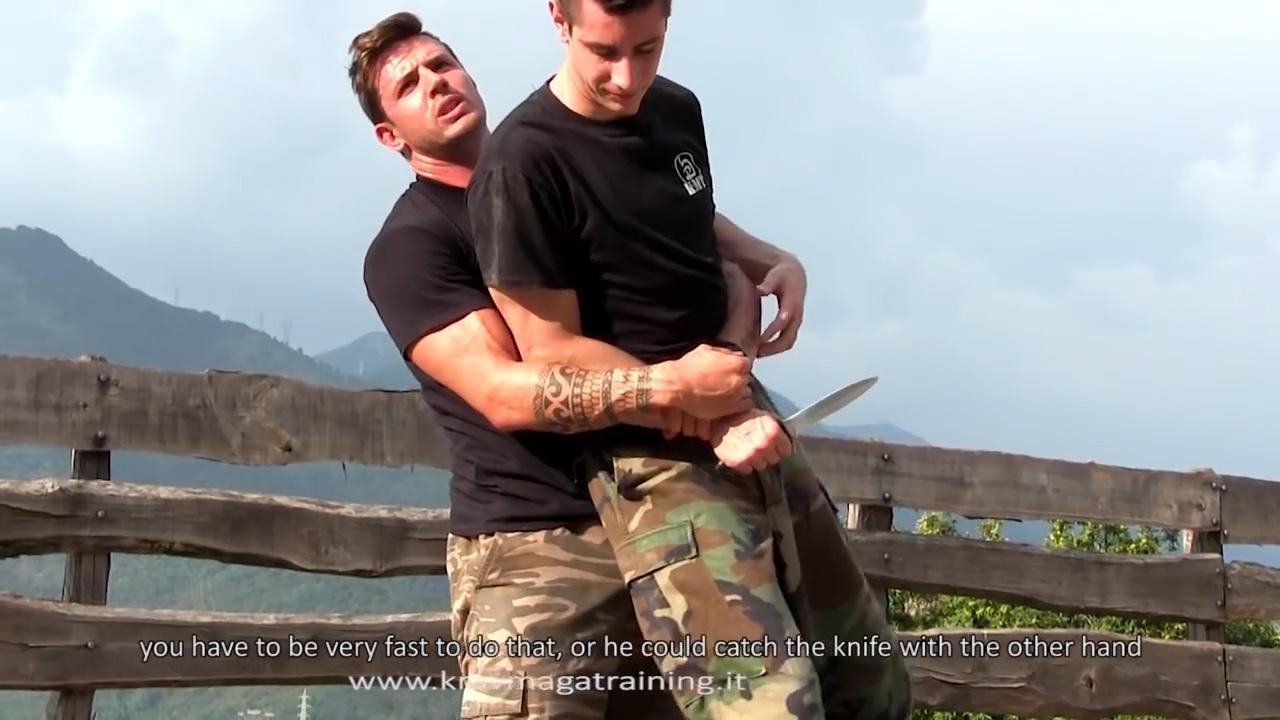

Supplement: Supplementary file 2 — Supplementary Information 2. [file 41598_2023_35190_MOESM2_ESM.zip › test/images/KravMagaTraining21139_jpg.rf.5e26c74bf6dc7cc39d04b0cbed9569e9.jpg]

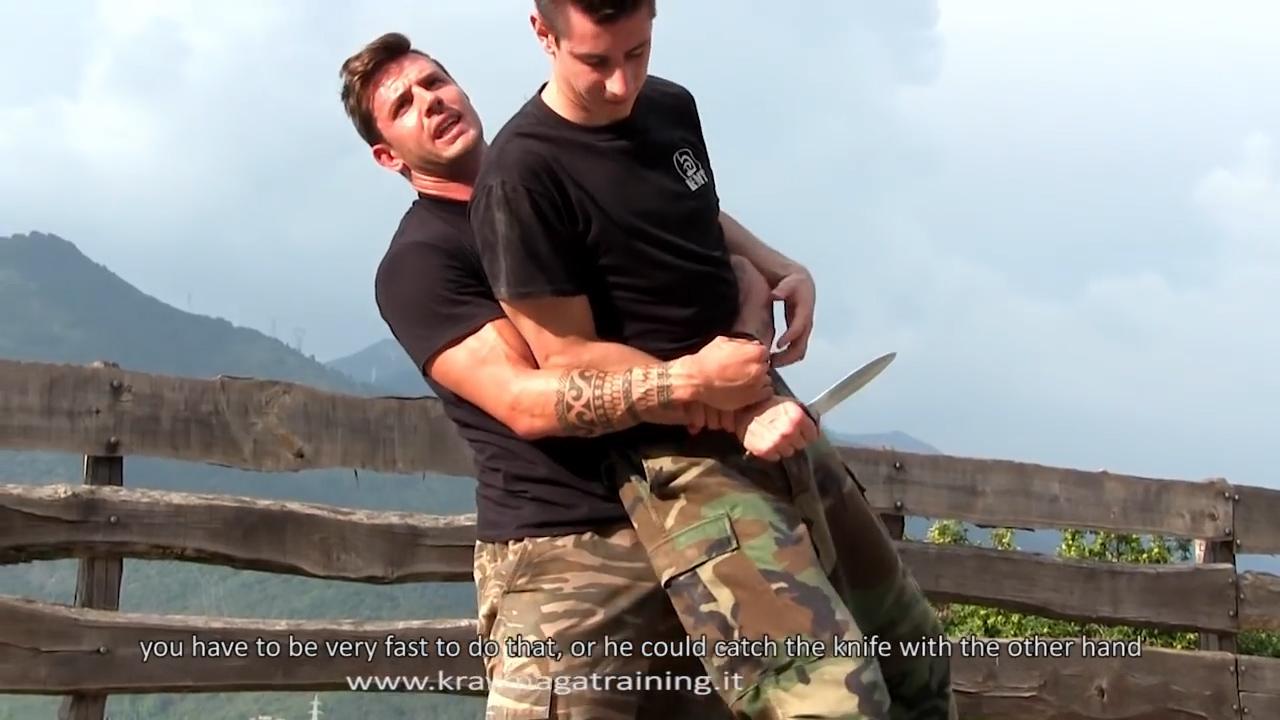

Supplement: Supplementary file 2 — Supplementary Information 2. [file 41598_2023_35190_MOESM2_ESM.zip › test/images/KravMagaTraining21140_jpg.rf.071f3ae8453c6c87f74a0a3ef30adec9.jpg]

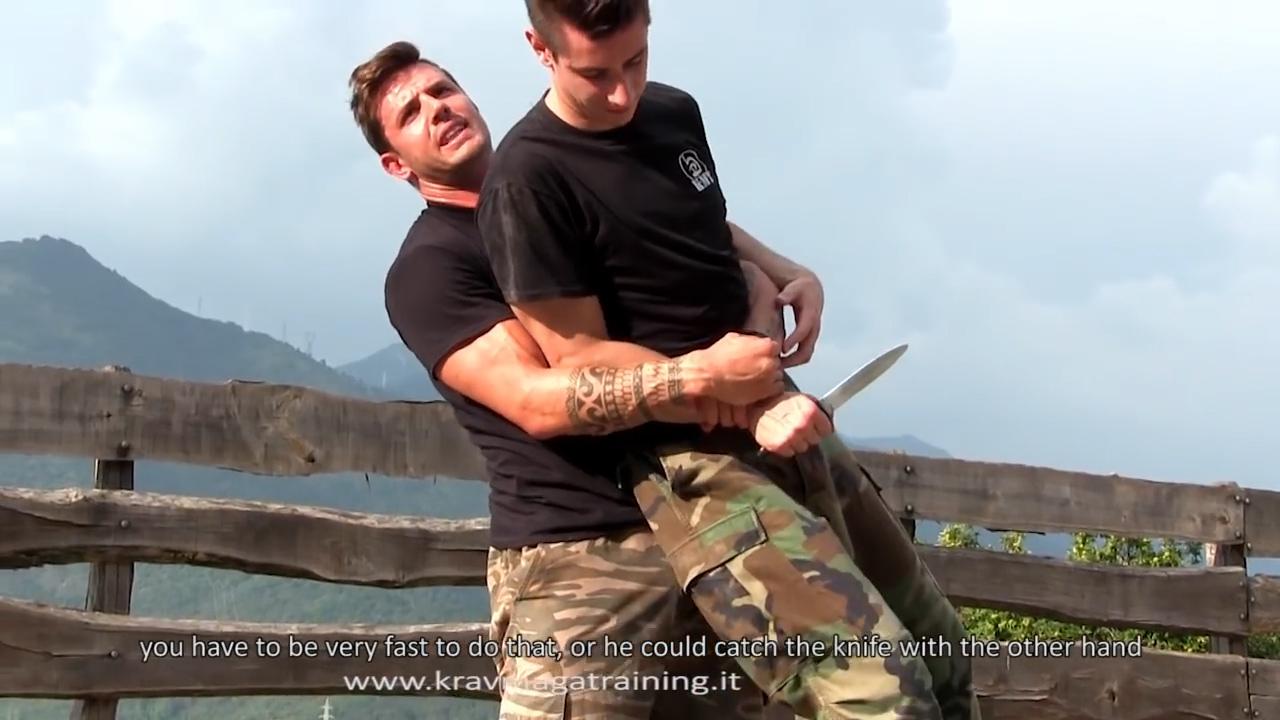

Supplement: Supplementary file 2 — Supplementary Information 2. [file 41598_2023_35190_MOESM2_ESM.zip › test/images/KravMagaTraining21141_jpg.rf.4225ef25f7e50c84a7bdb6517bd73698.jpg]

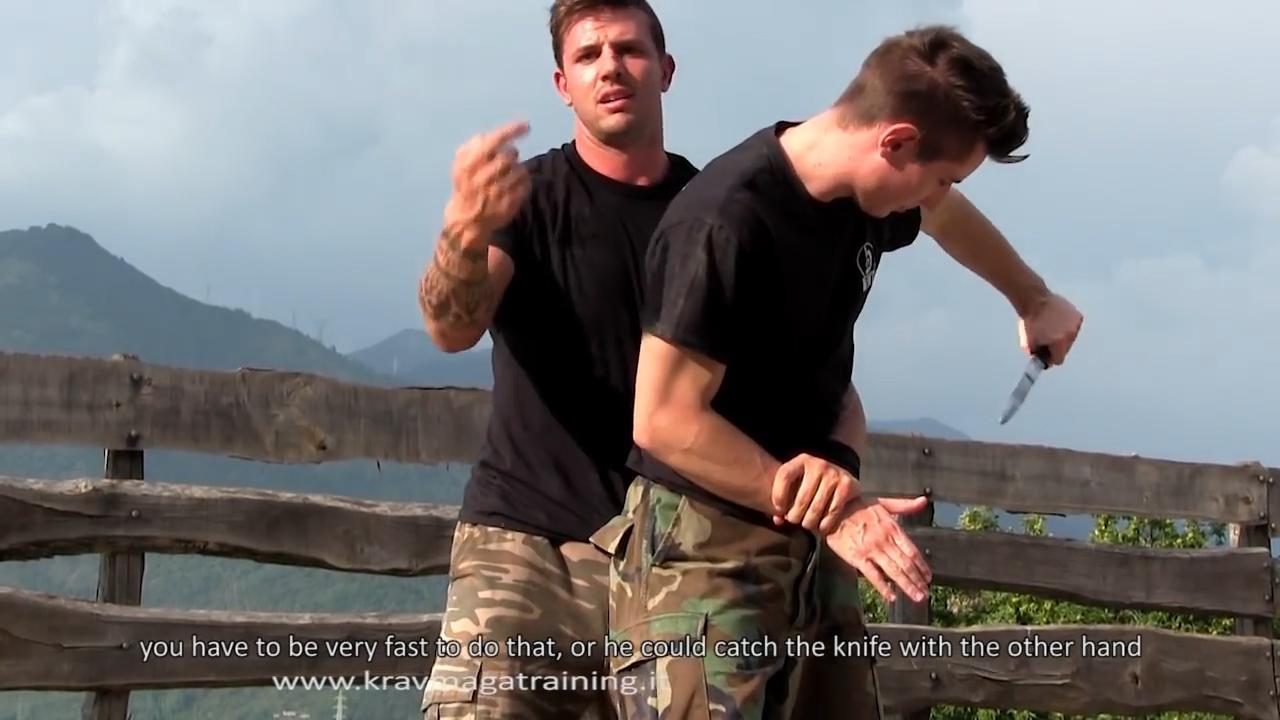

Supplement: Supplementary file 2 — Supplementary Information 2. [file 41598_2023_35190_MOESM2_ESM.zip › test/images/KravMagaTraining21167_jpg.rf.e73c35e6ea2ab64e01707e8b078647ed.jpg]

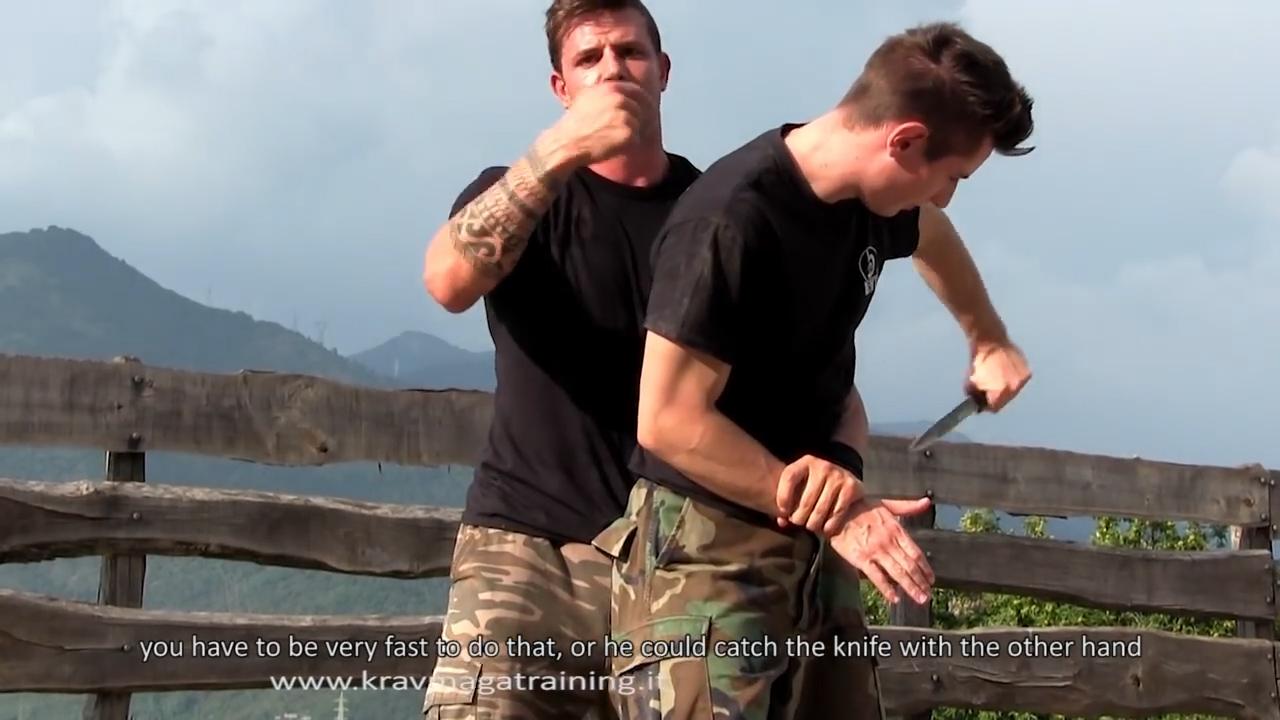

Supplement: Supplementary file 2 — Supplementary Information 2. [file 41598_2023_35190_MOESM2_ESM.zip › test/images/KravMagaTraining21168_jpg.rf.30ddf8c5bf4fbb36e5d08ca352462502.jpg]

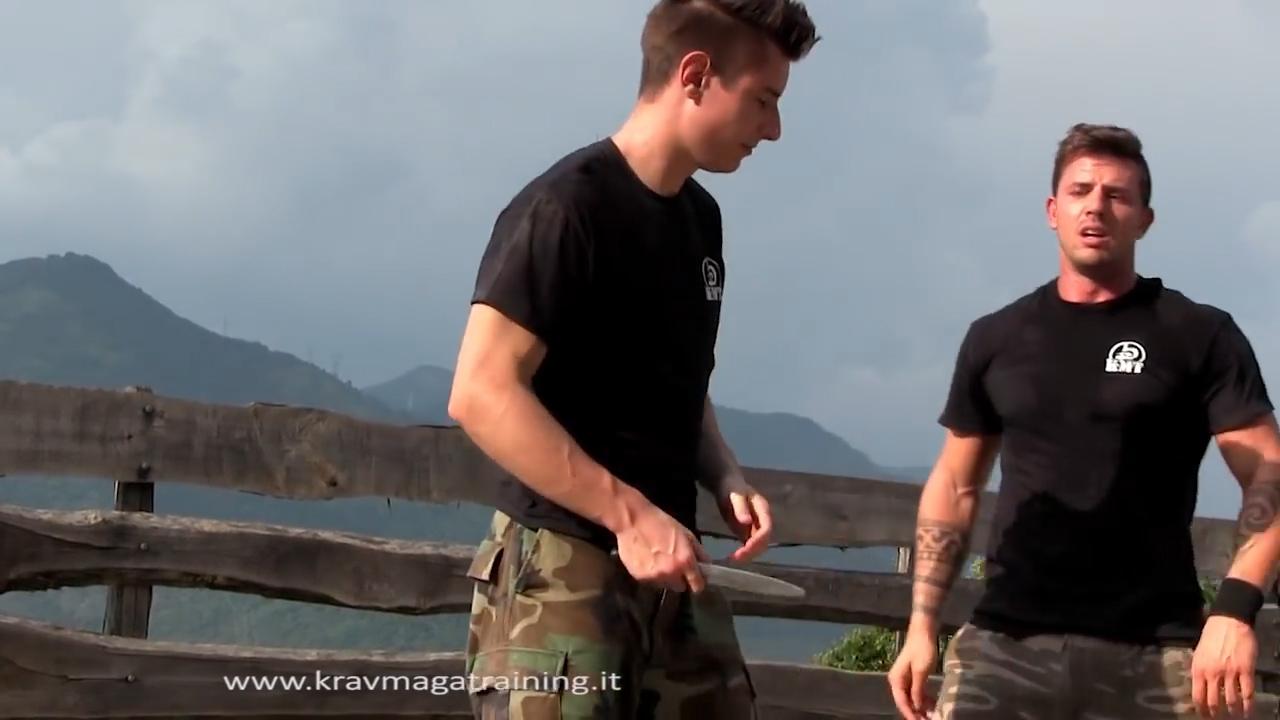

Supplement: Supplementary file 2 — Supplementary Information 2. [file 41598_2023_35190_MOESM2_ESM.zip › test/images/KravMagaTraining21185_jpg.rf.231850d9e0a15478950c0a9cff4784cd.jpg]

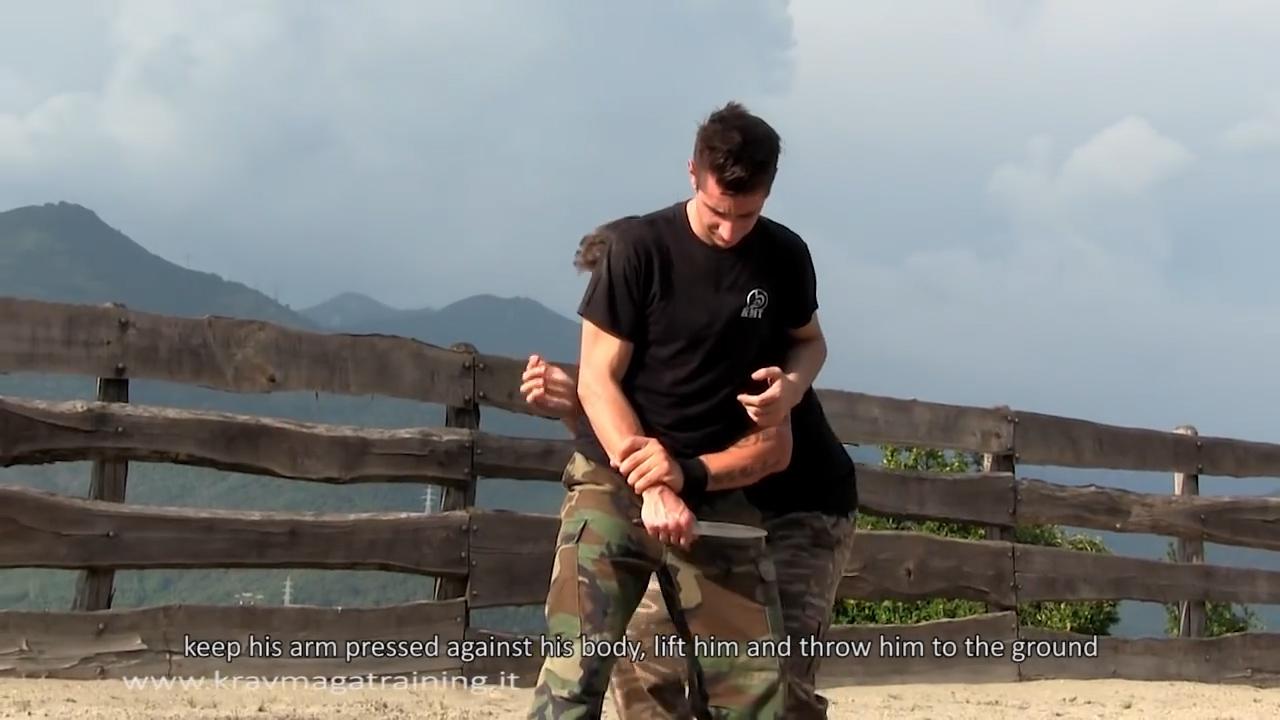

Supplement: Supplementary file 2 — Supplementary Information 2. [file 41598_2023_35190_MOESM2_ESM.zip › test/images/KravMagaTraining21222_jpg.rf.6f262e8222f27470b4f2c7ae954d93dd.jpg]

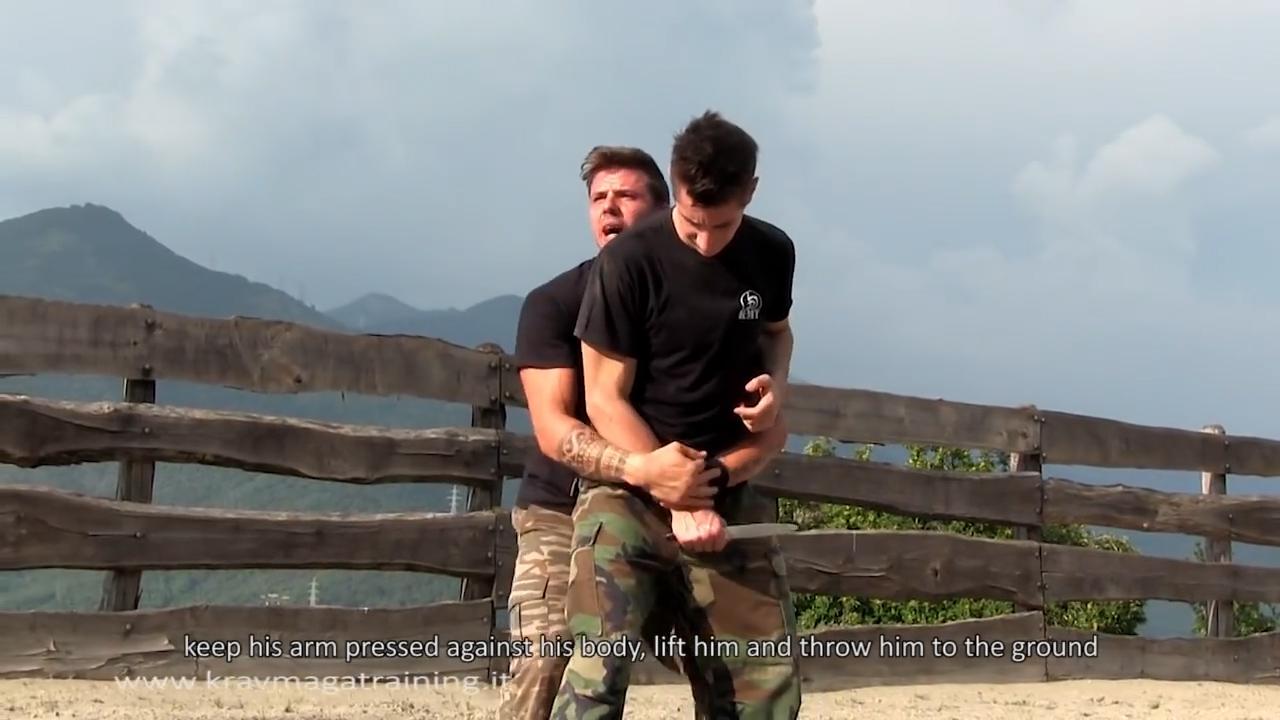

Supplement: Supplementary file 2 — Supplementary Information 2. [file 41598_2023_35190_MOESM2_ESM.zip › test/images/KravMagaTraining21225_jpg.rf.b1fd616607840a764f07879896c44b2c.jpg]

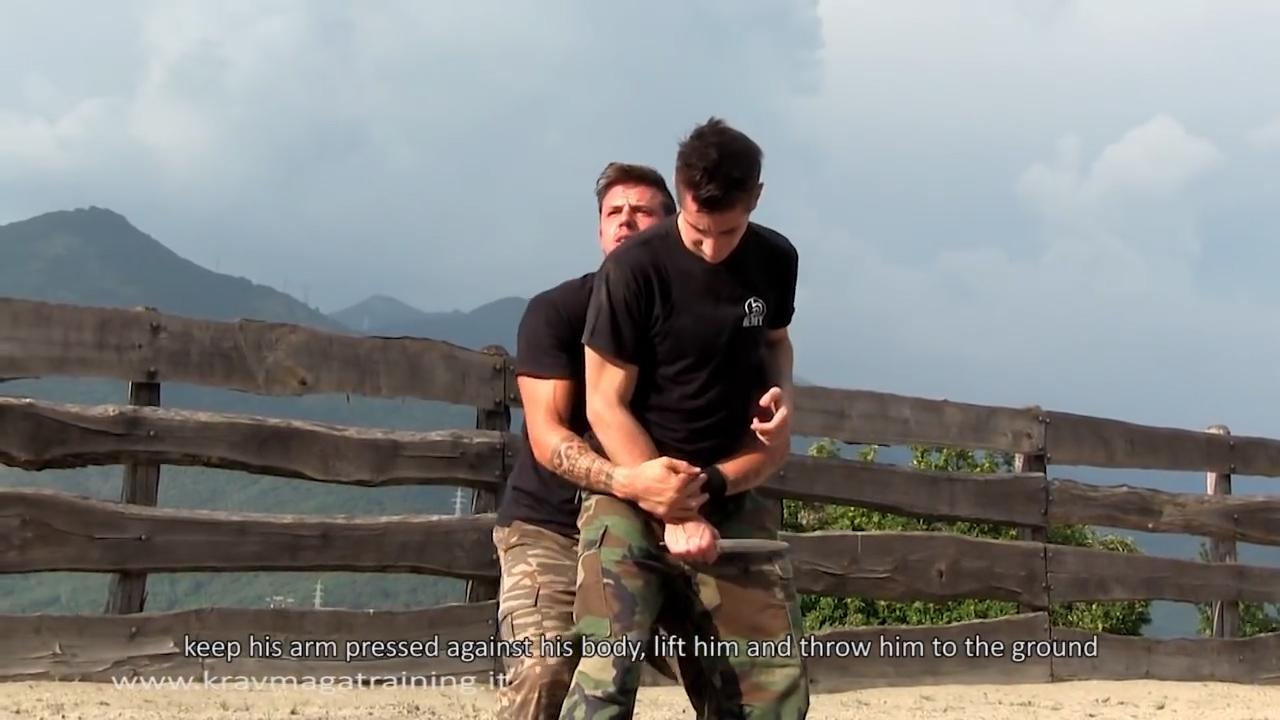

Supplement: Supplementary file 2 — Supplementary Information 2. [file 41598_2023_35190_MOESM2_ESM.zip › test/images/KravMagaTraining21226_jpg.rf.47820129d5e392a258eff93caff15b3c.jpg]

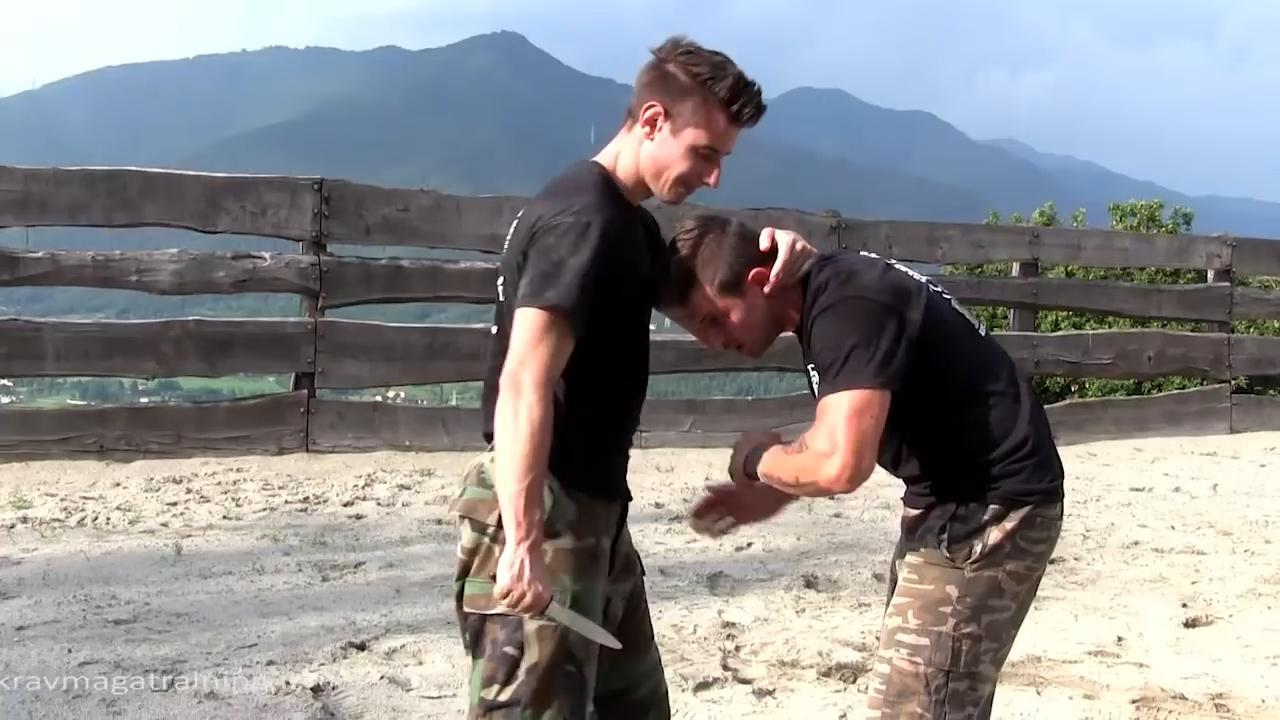

Supplement: Supplementary file 2 — Supplementary Information 2. [file 41598_2023_35190_MOESM2_ESM.zip › test/images/KravMagaTraining21302_jpg.rf.cf349d83ef38fe0e543ea7a78a57d614.jpg]

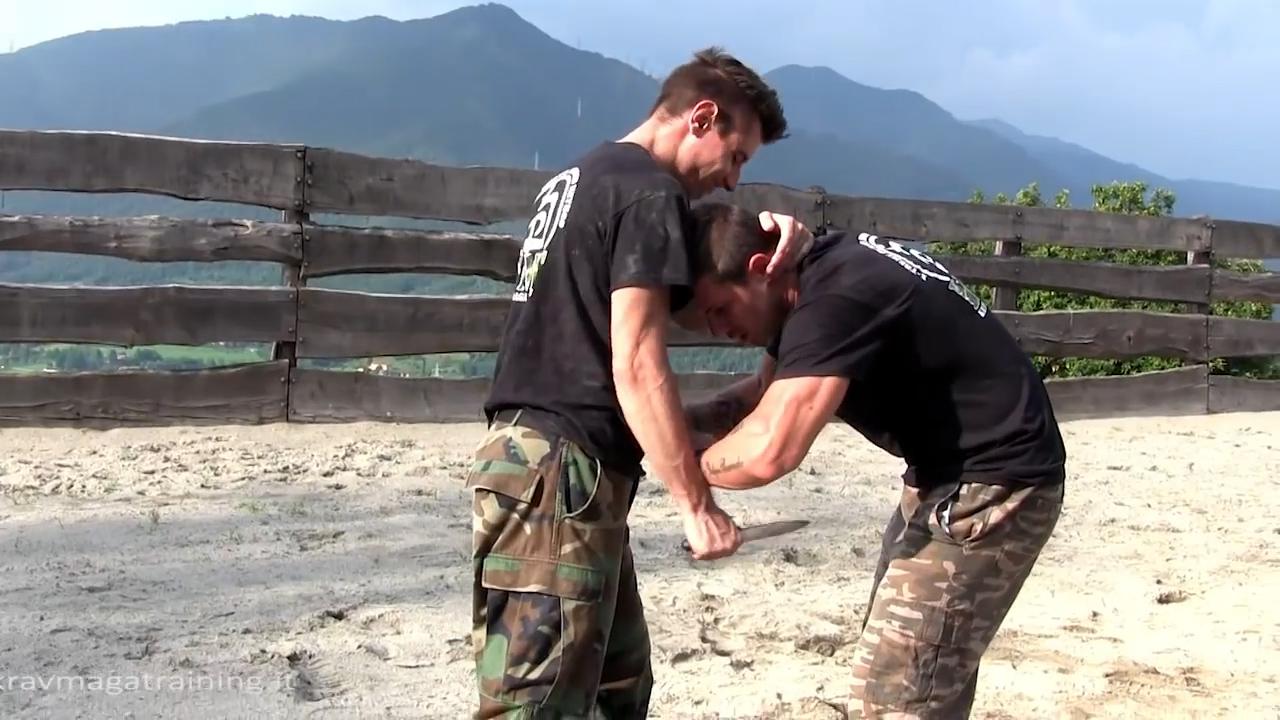

Supplement: Supplementary file 2 — Supplementary Information 2. [file 41598_2023_35190_MOESM2_ESM.zip › test/images/KravMagaTraining21304_jpg.rf.8eee3828f688dd07ee857be561318fea.jpg]

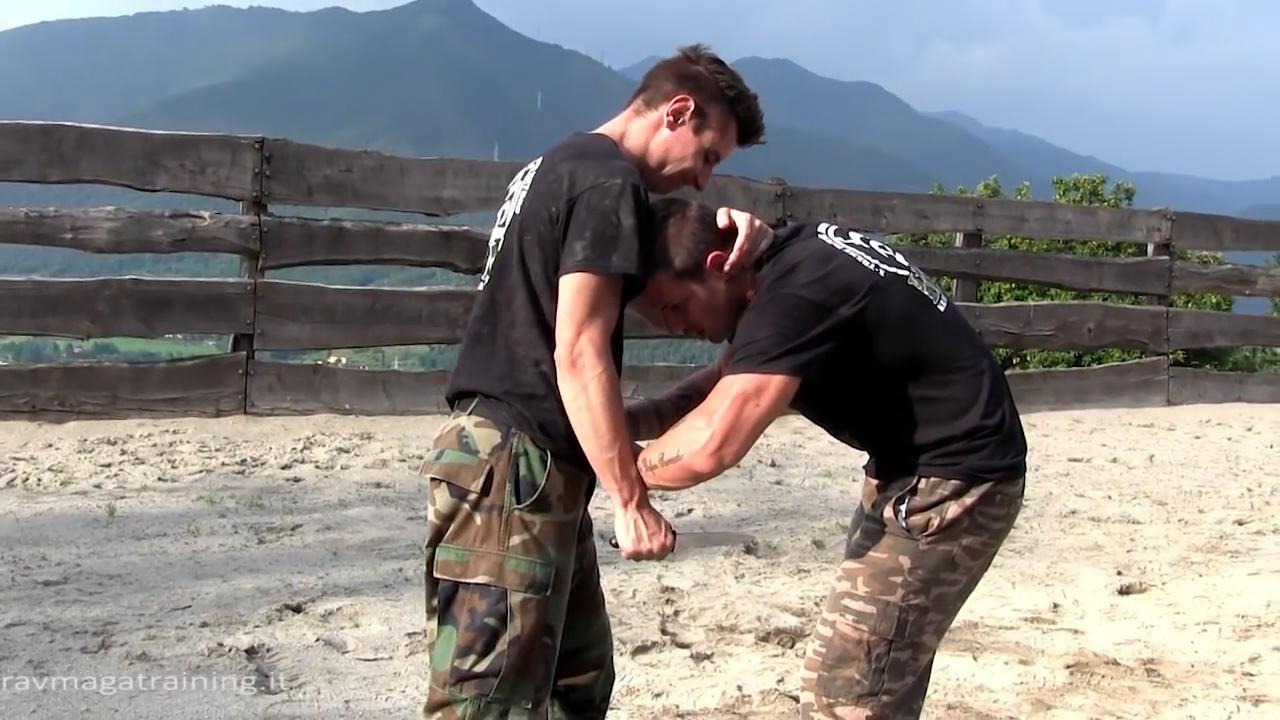

Supplement: Supplementary file 2 — Supplementary Information 2. [file 41598_2023_35190_MOESM2_ESM.zip › test/images/KravMagaTraining21306_jpg.rf.6e03f1297e46398d00a800e27d87fe5c.jpg]

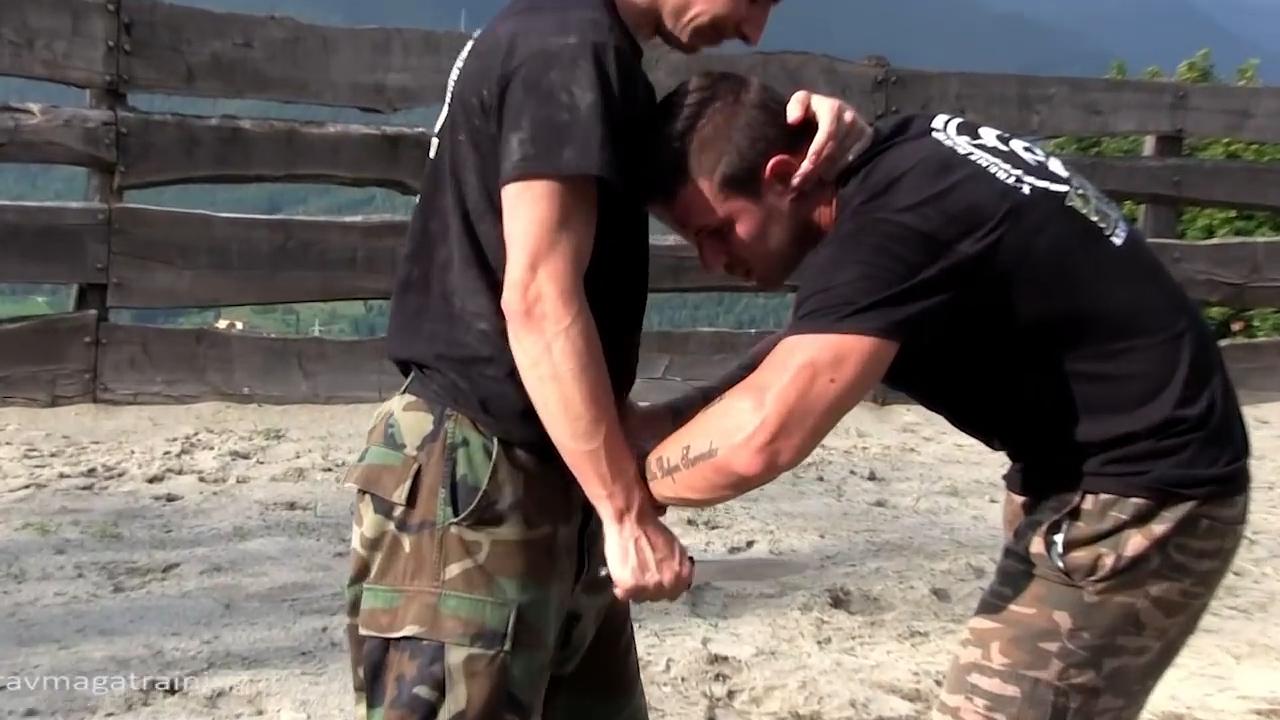

Supplement: Supplementary file 2 — Supplementary Information 2. [file 41598_2023_35190_MOESM2_ESM.zip › test/images/KravMagaTraining21309_jpg.rf.48685d66c5cb0c0cee02b44fb1f678b1.jpg]

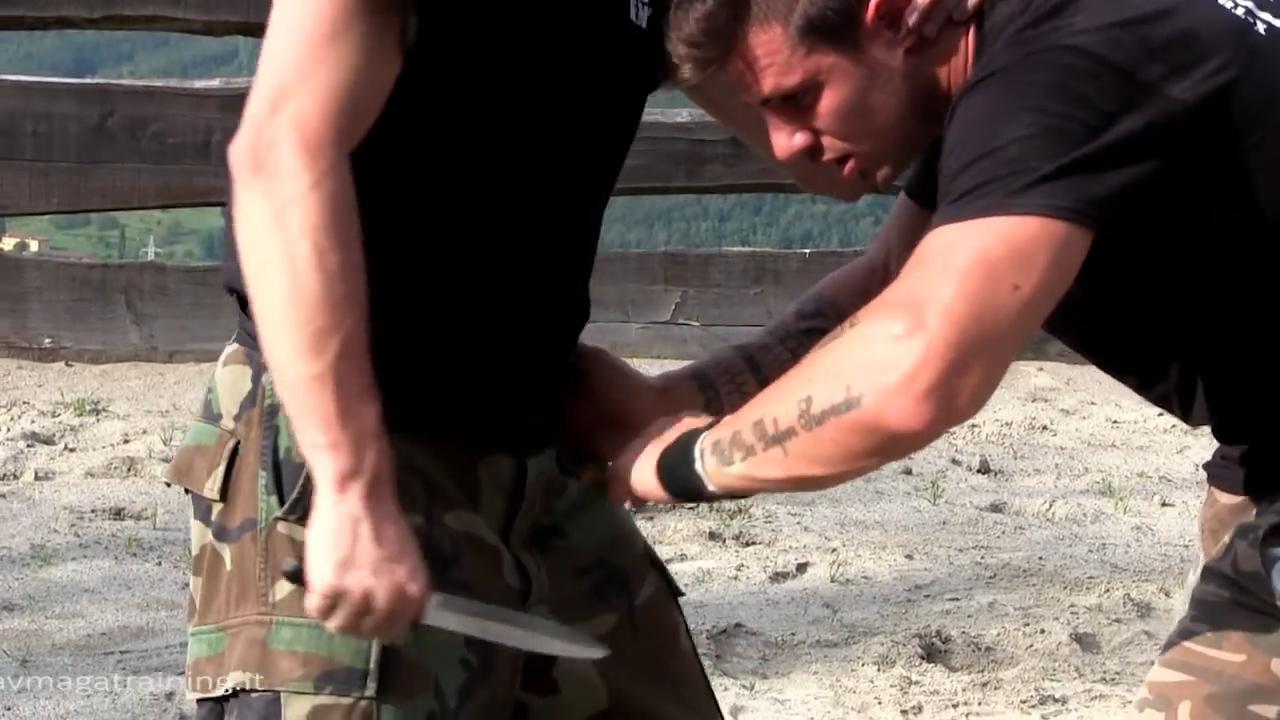

Supplement: Supplementary file 2 — Supplementary Information 2. [file 41598_2023_35190_MOESM2_ESM.zip › test/images/KravMagaTraining21315_jpg.rf.a7644094c6a29a6424dd1e0b3de2a20d.jpg]

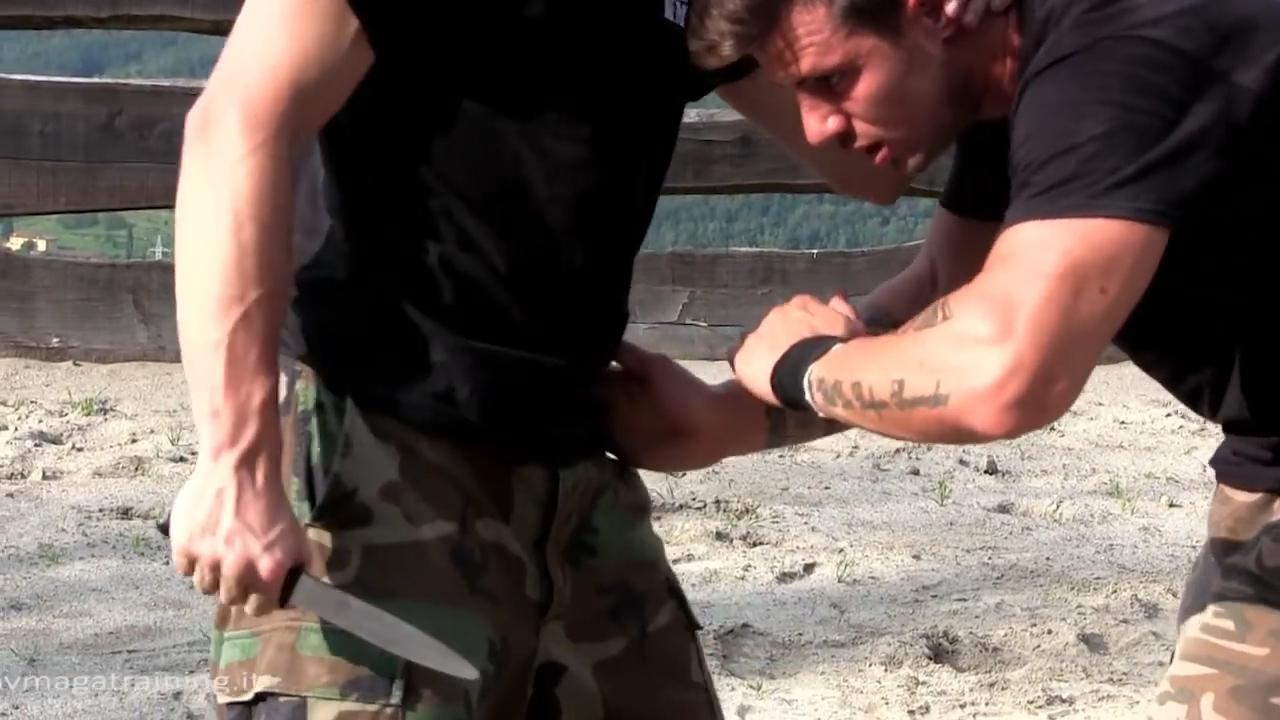

Supplement: Supplementary file 2 — Supplementary Information 2. [file 41598_2023_35190_MOESM2_ESM.zip › test/images/KravMagaTraining21316_jpg.rf.e944cbc46a100ae1c1ff01c66898a67b.jpg]

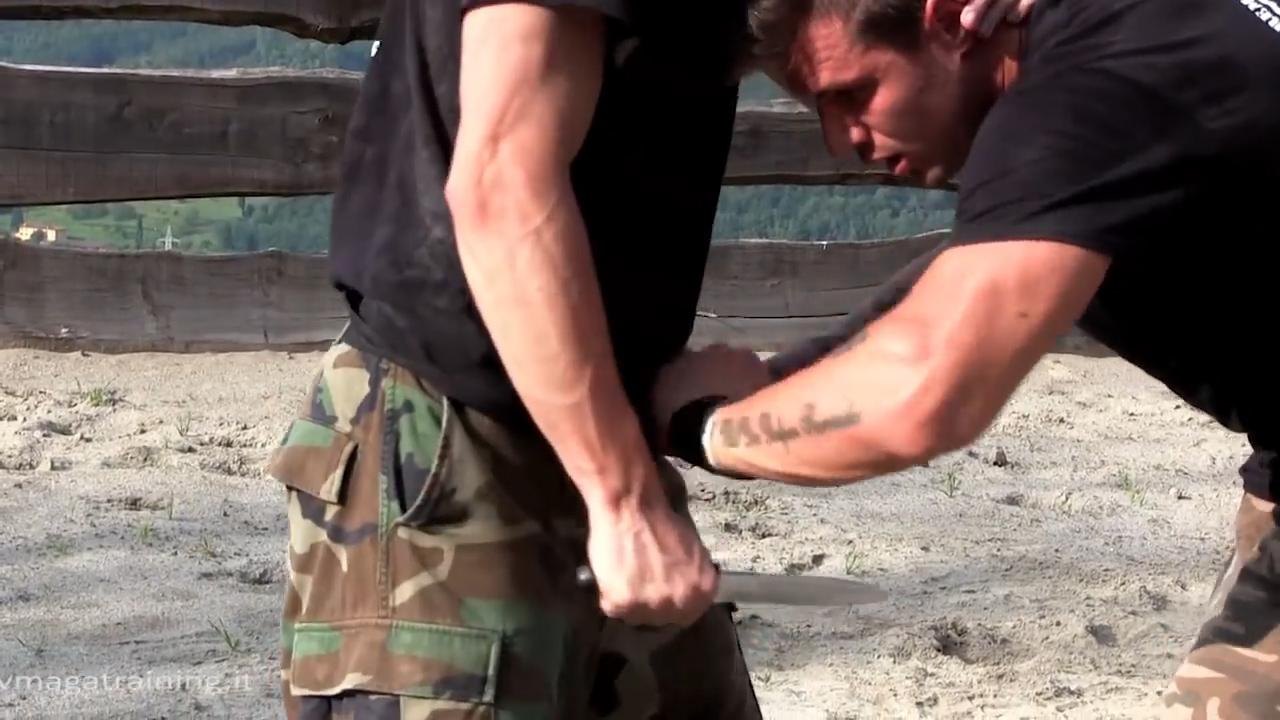

Supplement: Supplementary file 2 — Supplementary Information 2. [file 41598_2023_35190_MOESM2_ESM.zip › test/images/KravMagaTraining21319_jpg.rf.a5b2e3cf664c0f5e24f1ff5383f7a2eb.jpg]

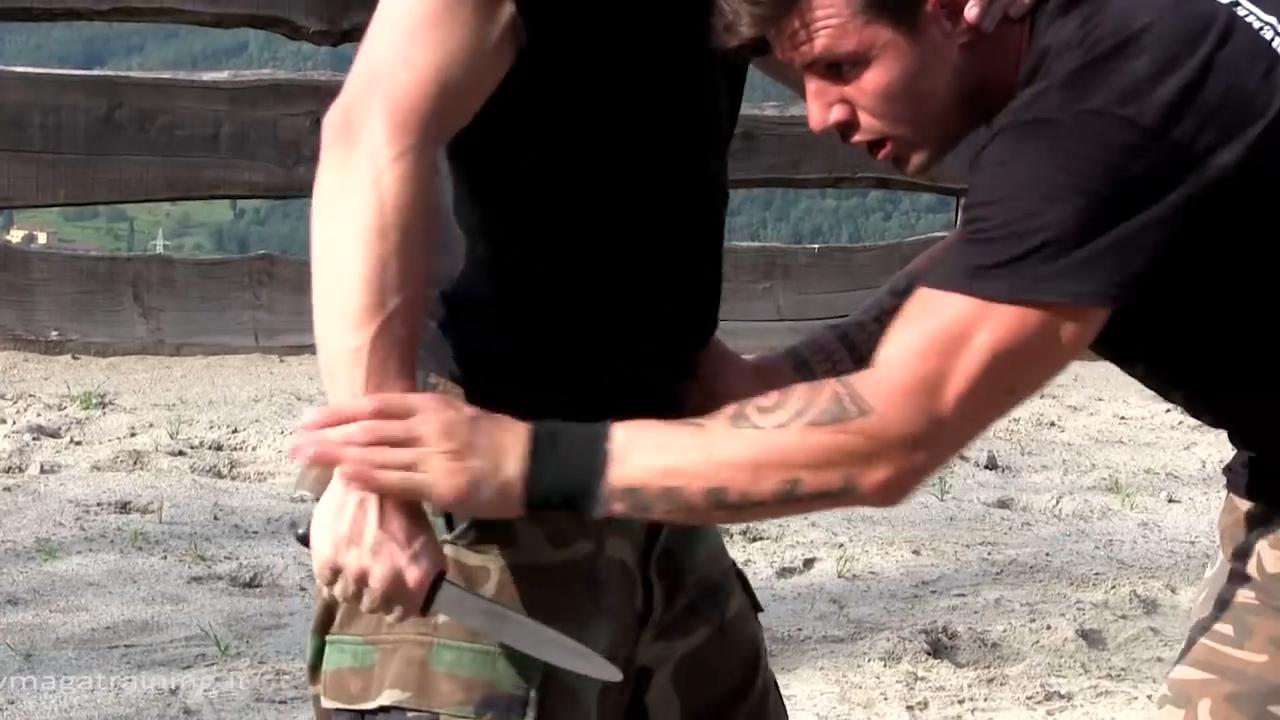

Supplement: Supplementary file 2 — Supplementary Information 2. [file 41598_2023_35190_MOESM2_ESM.zip › test/images/KravMagaTraining21321_jpg.rf.52b69d72aa79c7a112d13eba0f9c87c0.jpg]

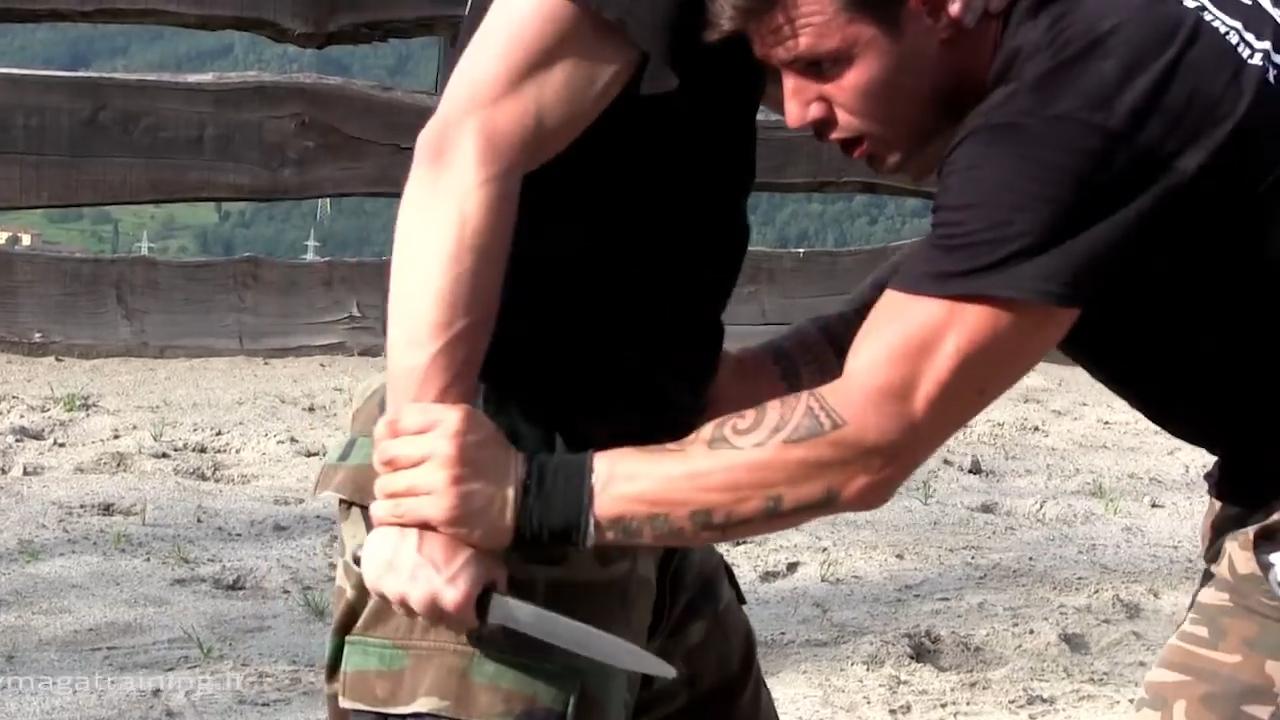

Supplement: Supplementary file 2 — Supplementary Information 2. [file 41598_2023_35190_MOESM2_ESM.zip › test/images/KravMagaTraining21322_jpg.rf.32bef26ef87e8ab865303185e91337b4.jpg]

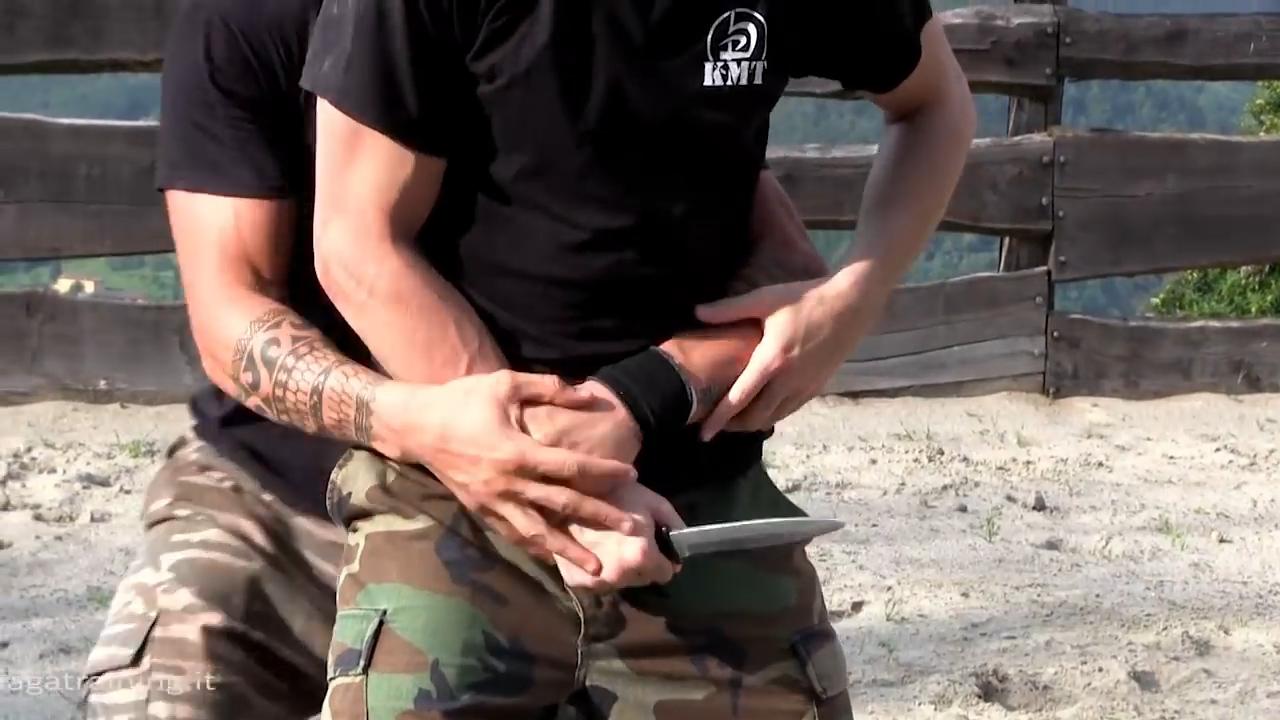

Supplement: Supplementary file 2 — Supplementary Information 2. [file 41598_2023_35190_MOESM2_ESM.zip › test/images/KravMagaTraining21333_jpg.rf.d3d10614a31ceca837f1d288e6b8bbbf.jpg]

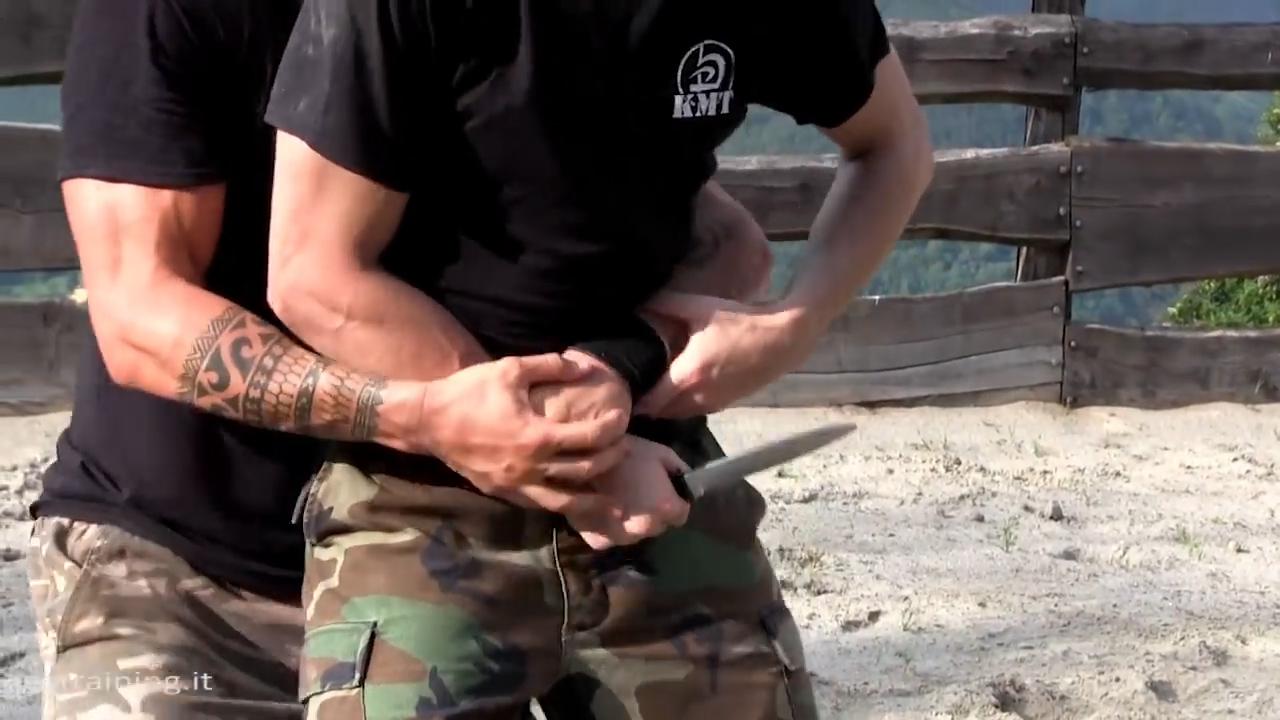

Supplement: Supplementary file 2 — Supplementary Information 2. [file 41598_2023_35190_MOESM2_ESM.zip › test/images/KravMagaTraining21334_jpg.rf.44bb60dc4747c500d6b0f803dcc97faa.jpg]

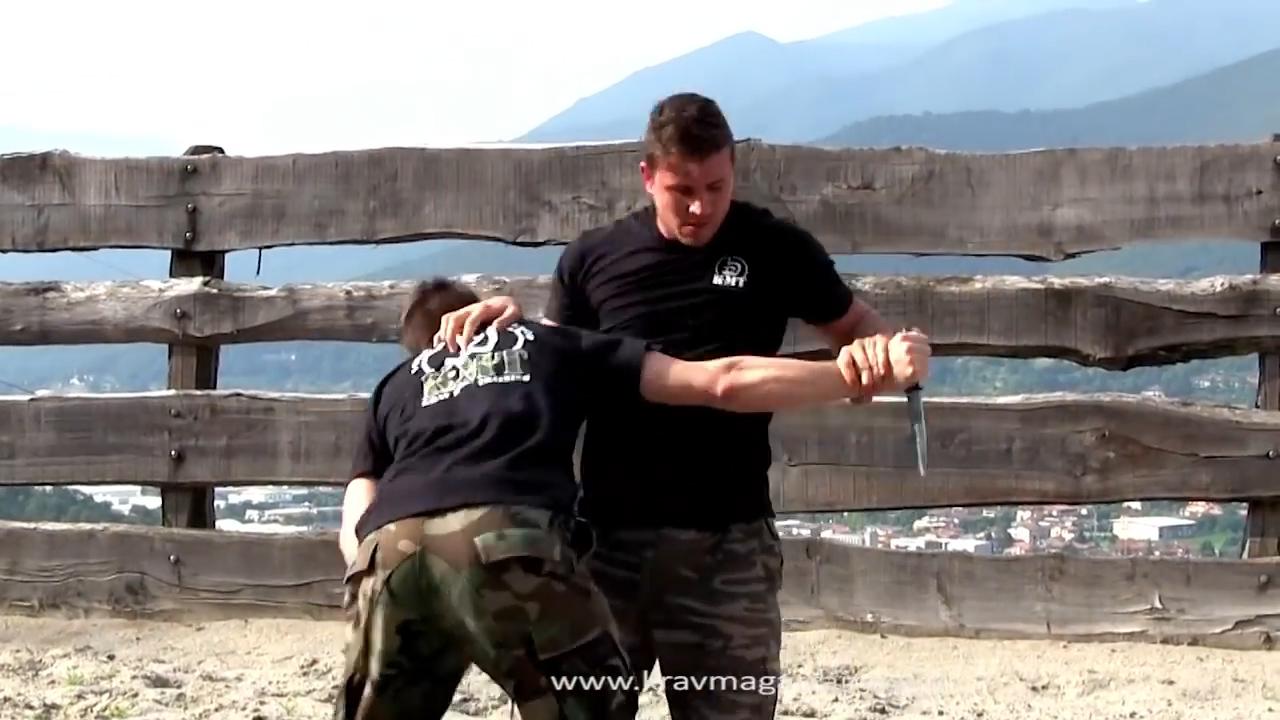

Supplement: Supplementary file 2 — Supplementary Information 2. [file 41598_2023_35190_MOESM2_ESM.zip › test/images/KravMagaTraining215_jpg.rf.18e4898dafa959743c8a4201ae5474ae.jpg]

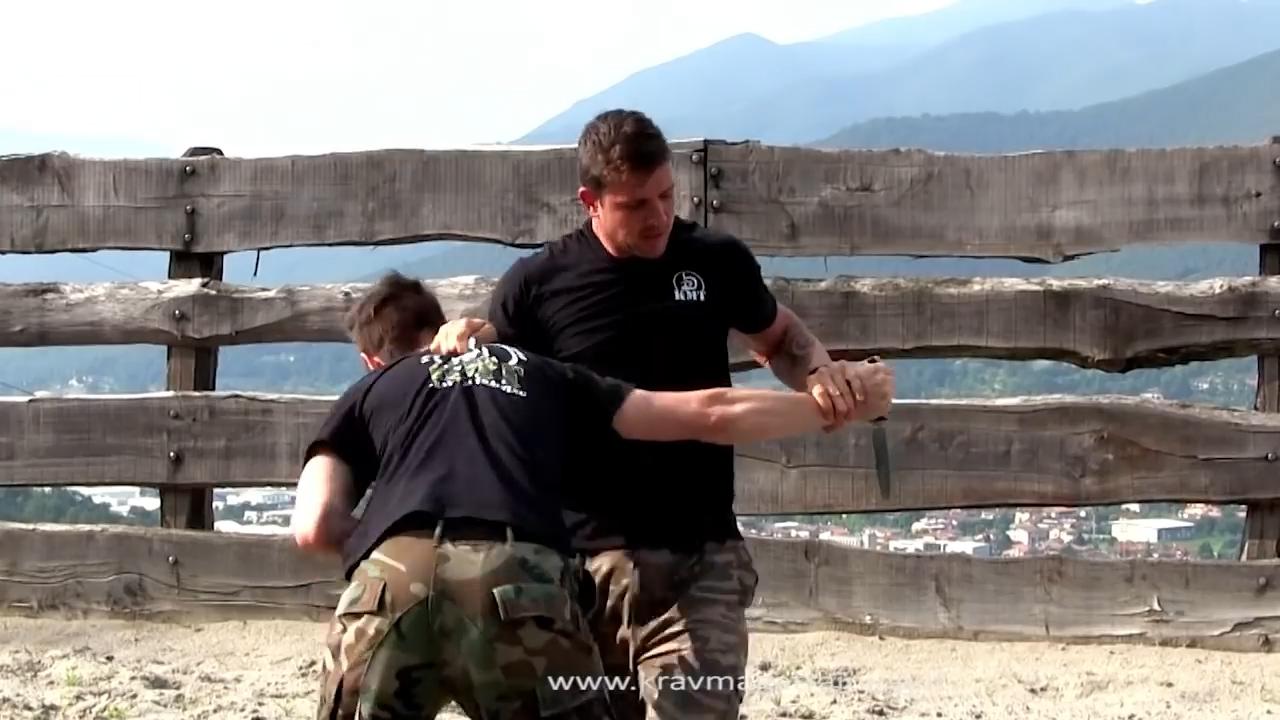

Supplement: Supplementary file 2 — Supplementary Information 2. [file 41598_2023_35190_MOESM2_ESM.zip › test/images/KravMagaTraining216_jpg.rf.c81f8f218699ae31047a5648d49f0ef1.jpg]

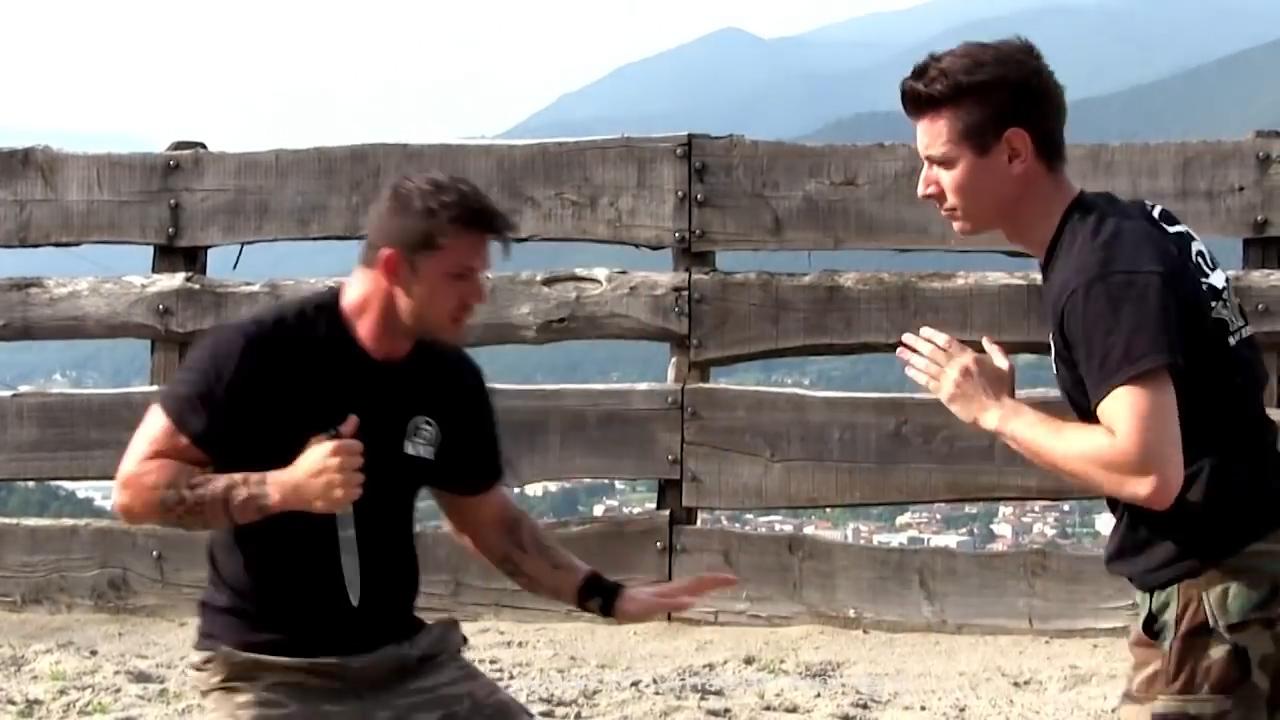

Supplement: Supplementary file 2 — Supplementary Information 2. [file 41598_2023_35190_MOESM2_ESM.zip › test/images/KravMagaTraining226_jpg.rf.ed4fc2f06e6d7d452d7bb30fed5027d6.jpg]

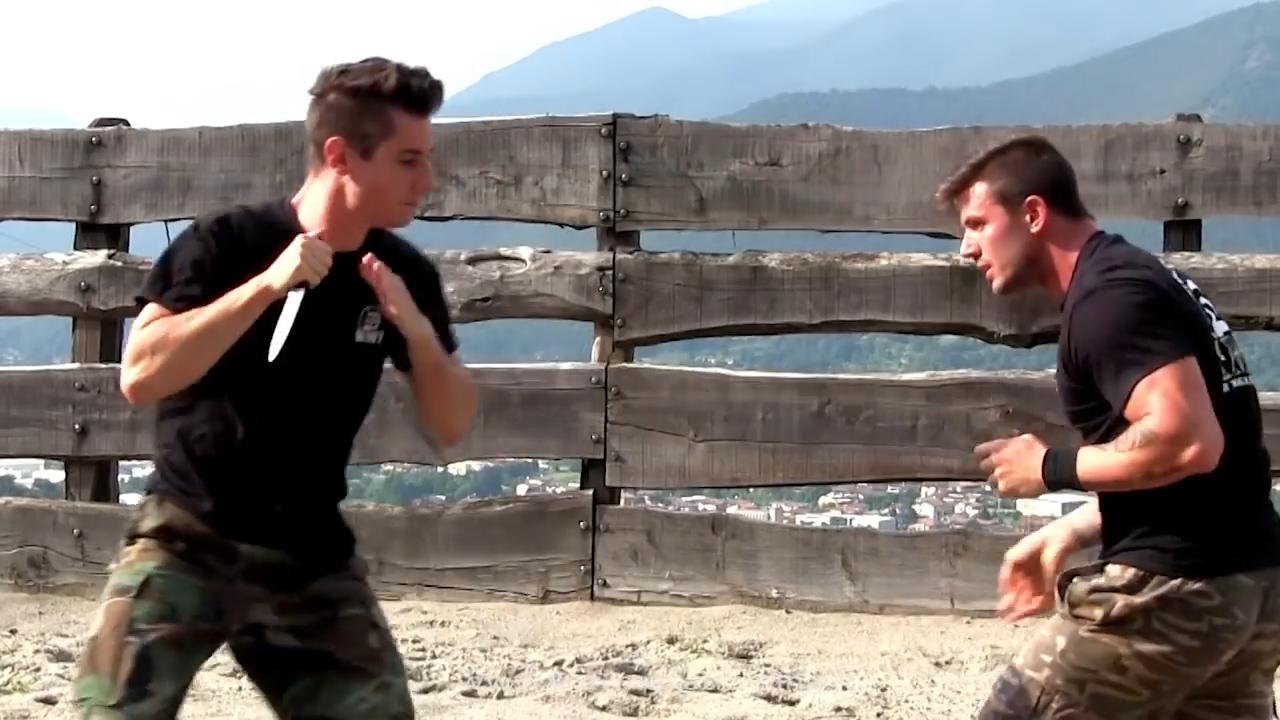

Supplement: Supplementary file 2 — Supplementary Information 2. [file 41598_2023_35190_MOESM2_ESM.zip › test/images/KravMagaTraining244_jpg.rf.db1af6112a6990fe675b791e7cac3e76.jpg]

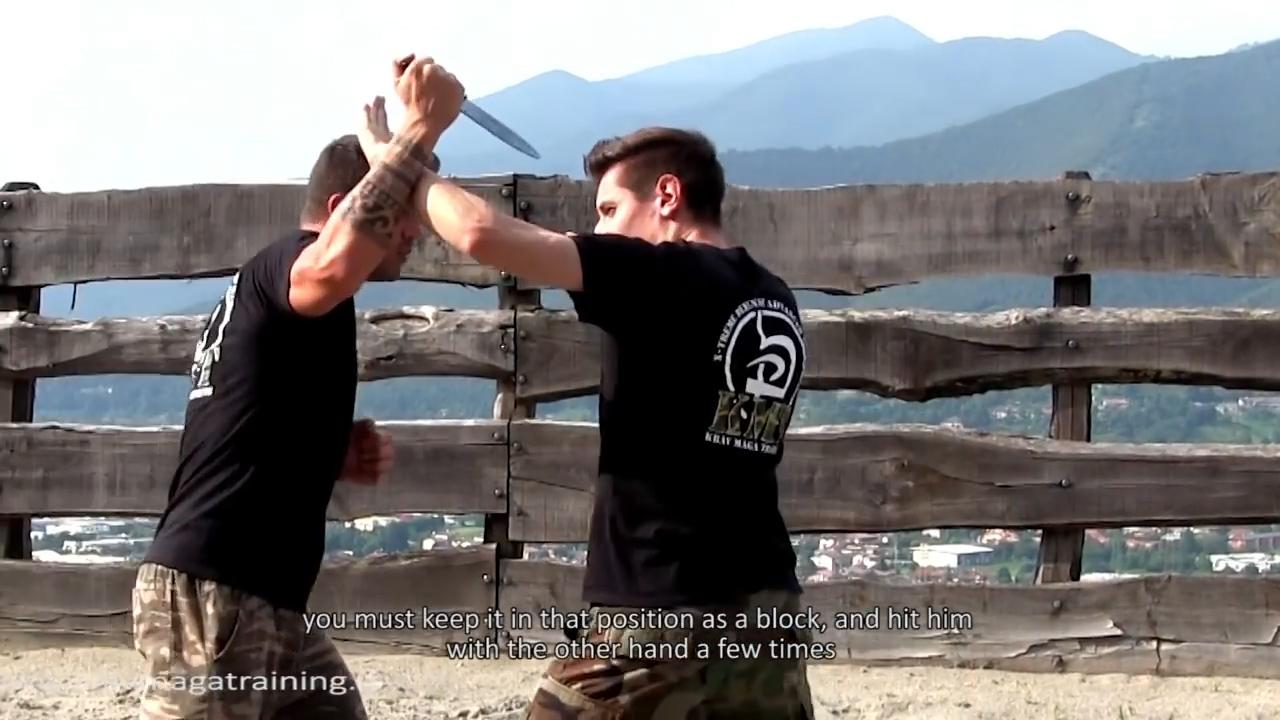

Supplement: Supplementary file 2 — Supplementary Information 2. [file 41598_2023_35190_MOESM2_ESM.zip › test/images/KravMagaTraining253_jpg.rf.ebf0c364189636ff022e60d1d6fc7014.jpg]

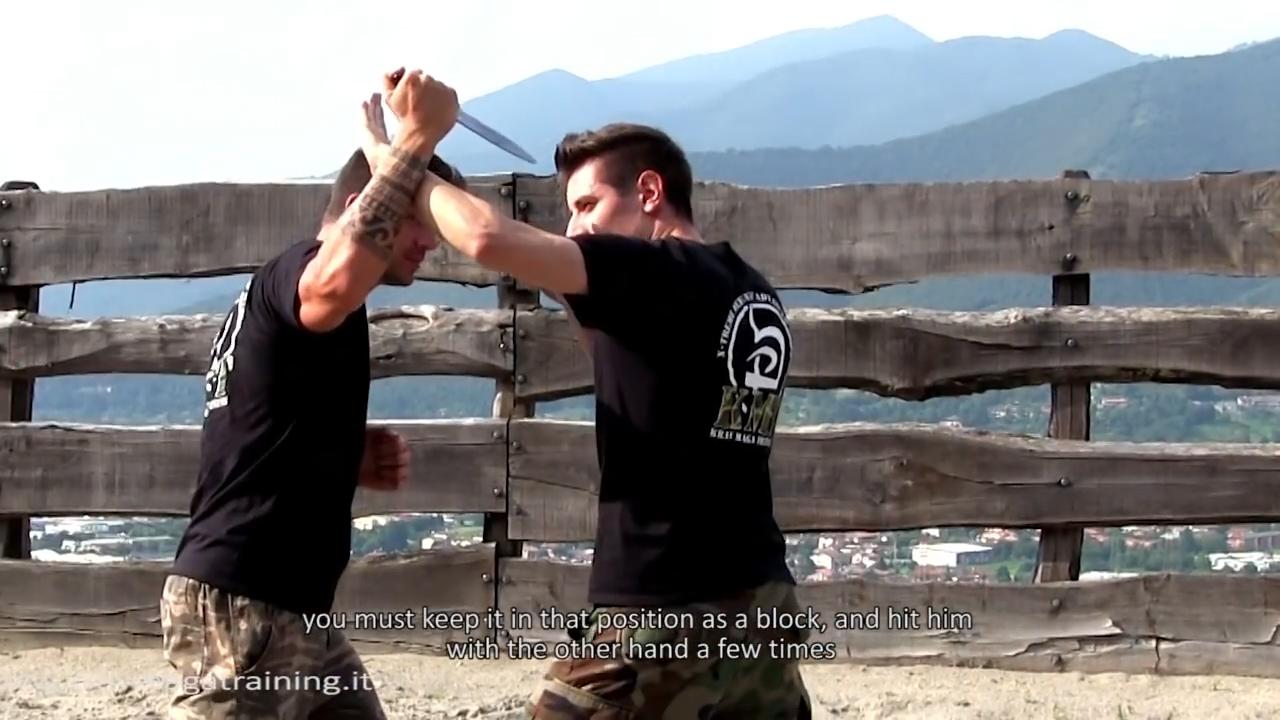

Supplement: Supplementary file 2 — Supplementary Information 2. [file 41598_2023_35190_MOESM2_ESM.zip › test/images/KravMagaTraining255_jpg.rf.567144f79ed517e7440fc41dc095d803.jpg]

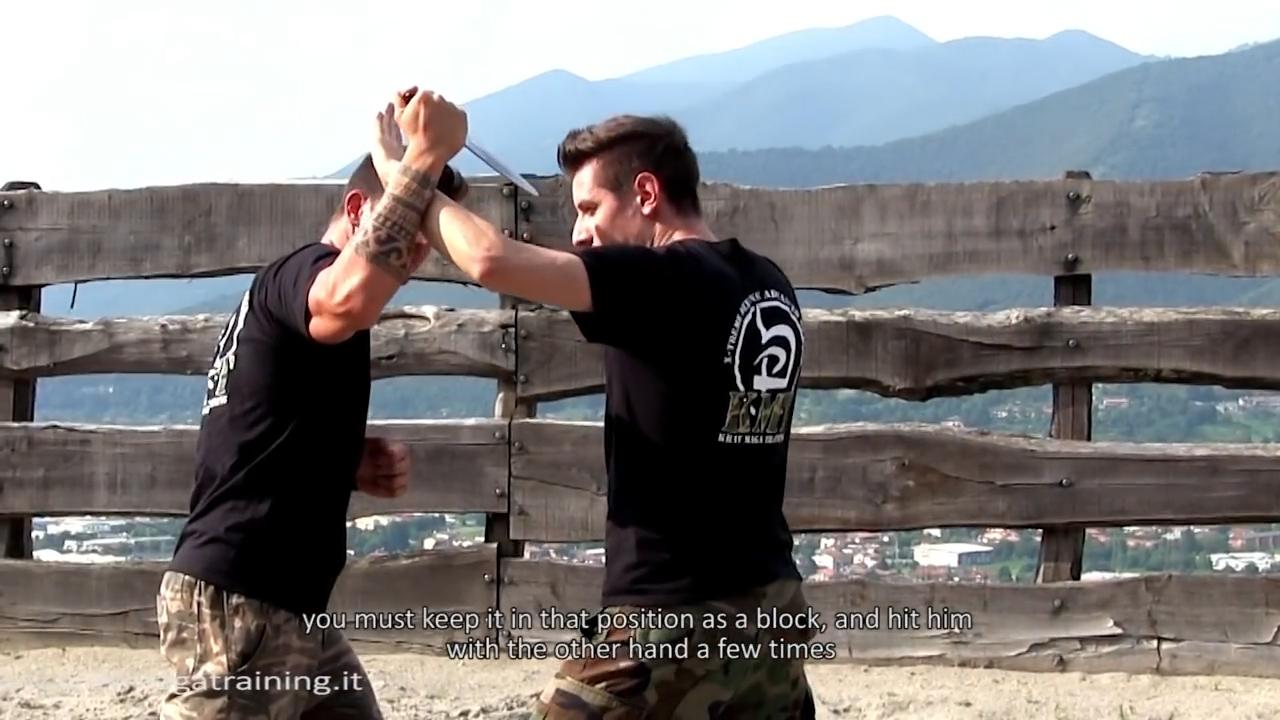

Supplement: Supplementary file 2 — Supplementary Information 2. [file 41598_2023_35190_MOESM2_ESM.zip › test/images/KravMagaTraining258_jpg.rf.a20b0aaee57feffb5fdbf3a078dce8a6.jpg]

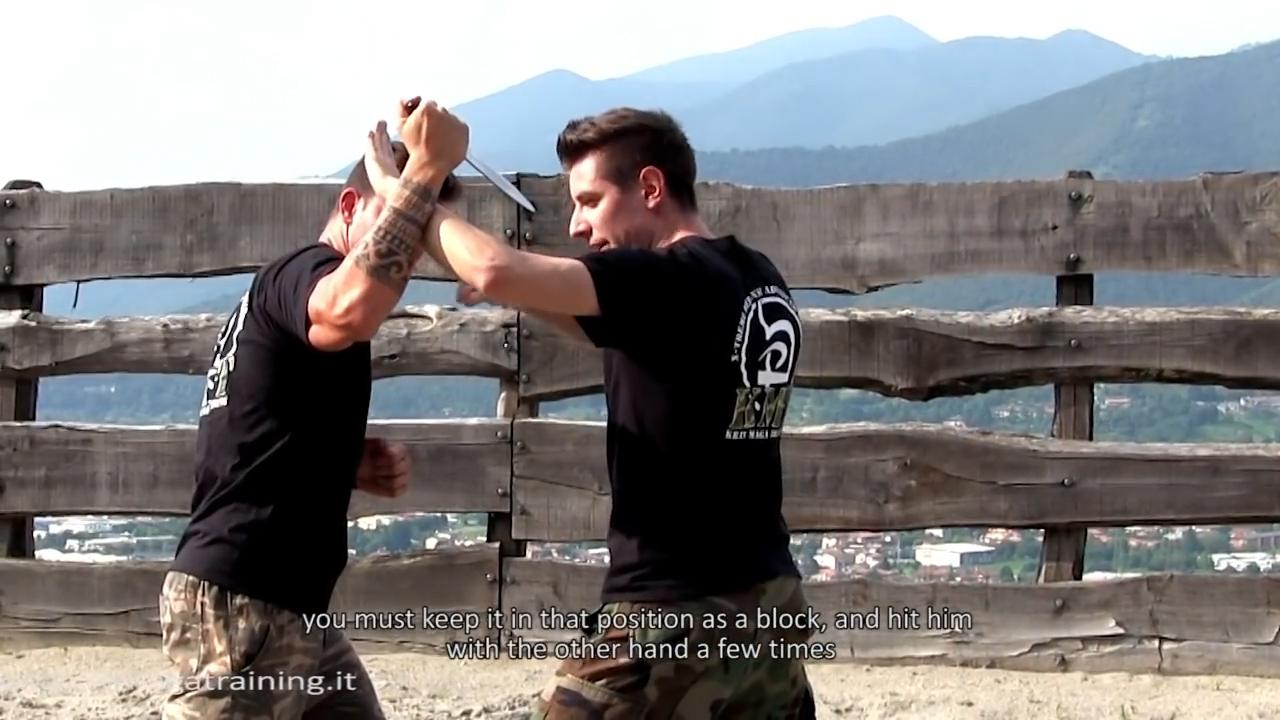

Supplement: Supplementary file 2 — Supplementary Information 2. [file 41598_2023_35190_MOESM2_ESM.zip › test/images/KravMagaTraining260_jpg.rf.4a17a31d01b4e4570ff8aad91e6c11cd.jpg]

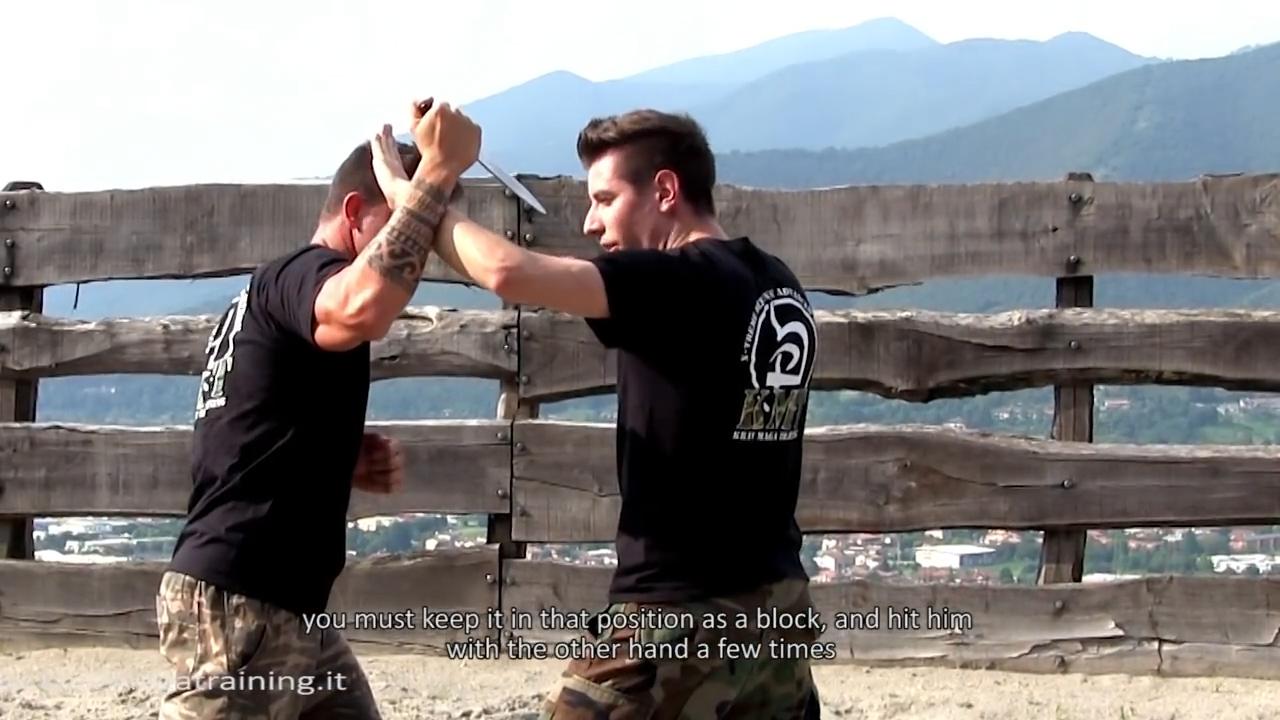

Supplement: Supplementary file 2 — Supplementary Information 2. [file 41598_2023_35190_MOESM2_ESM.zip › test/images/KravMagaTraining263_jpg.rf.82391d3291ede40c99b148b833ba0476.jpg]

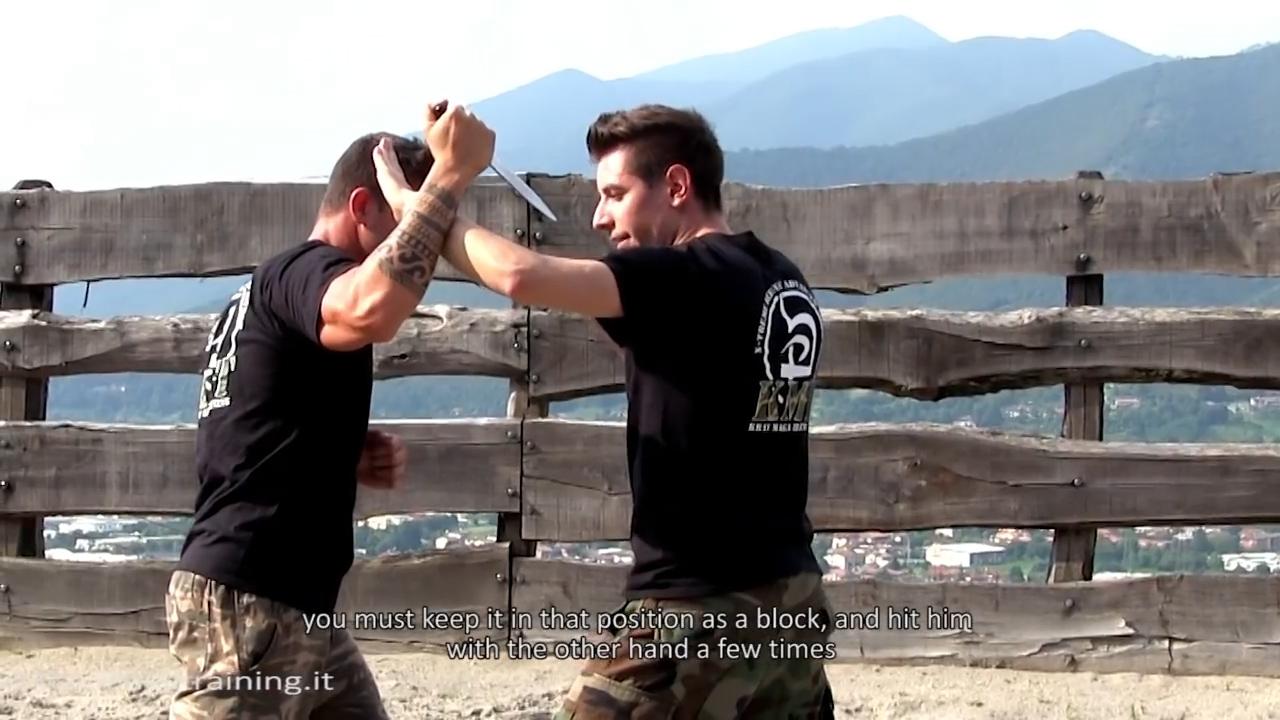

Supplement: Supplementary file 2 — Supplementary Information 2. [file 41598_2023_35190_MOESM2_ESM.zip › test/images/KravMagaTraining267_jpg.rf.6390872a54cfece6c1ab769f9bfd0b0b.jpg]
