# Supplementary figures and images for: Improving video surveillance systems in banks using deep learning techniques (part 3 of 4)
Source: Sci Rep. 2023 May 16;13:7911. doi: 10.1038/s41598-023-35190-9 (PMC10188611; doi:10.1038/s41598-023-35190-9)

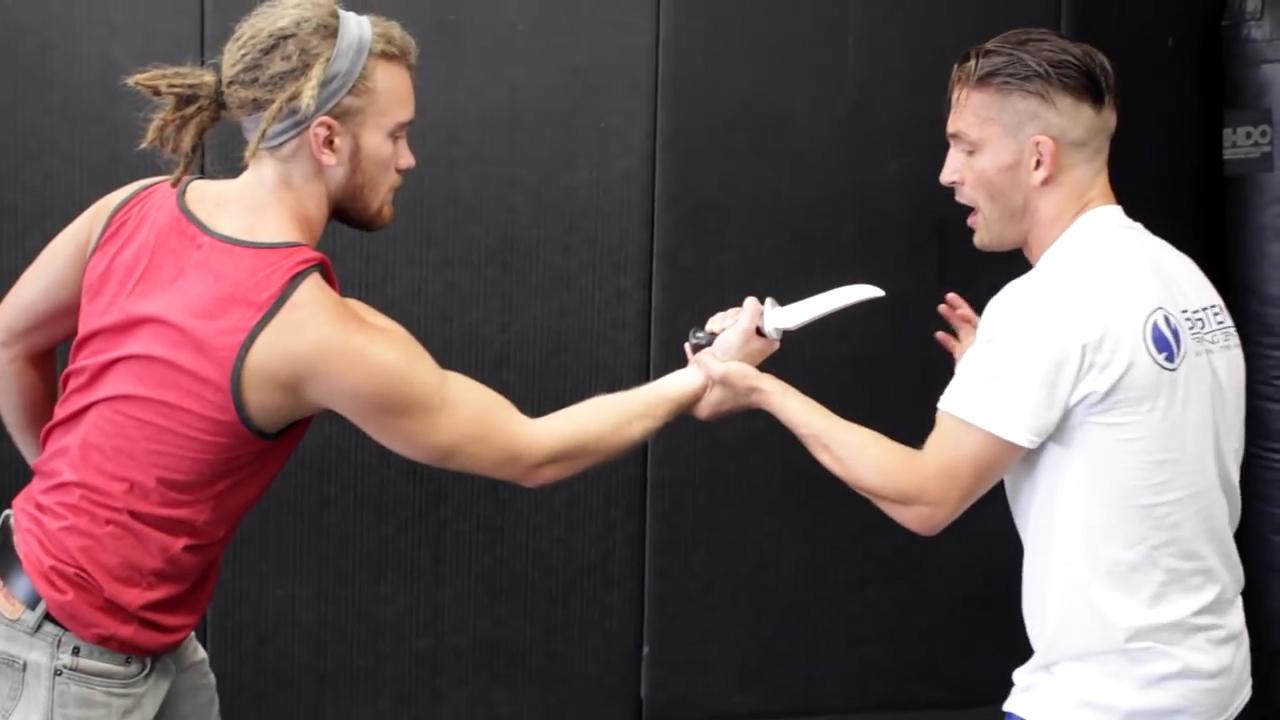

Supplement: Supplementary file 2 — Supplementary Information 2. [file 41598_2023_35190_MOESM2_ESM.zip › test/images/KravMagaKnifeDefenseTechniques267_jpg.rf.9d2688d265f91bfc943bbf896e4d4716.jpg]

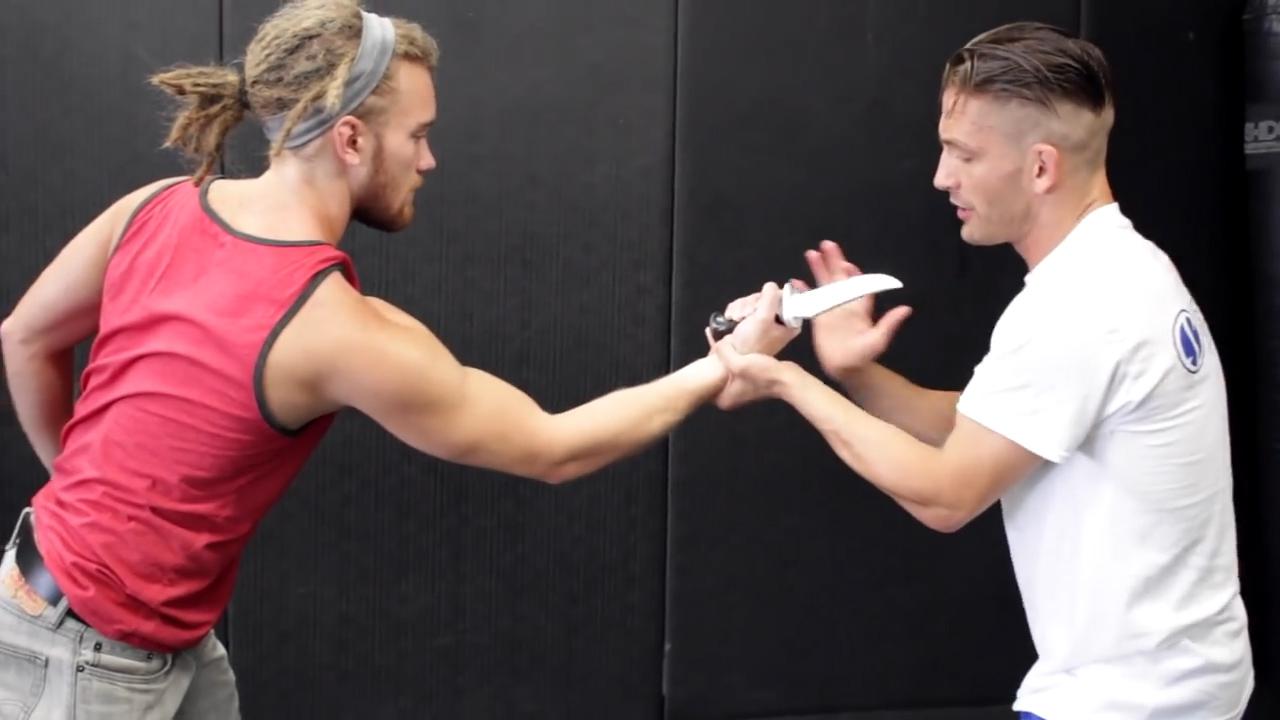

Supplement: Supplementary file 2 — Supplementary Information 2. [file 41598_2023_35190_MOESM2_ESM.zip › test/images/KravMagaKnifeDefenseTechniques269_jpg.rf.3fd3706333b59b83eaa808232ad85638.jpg]

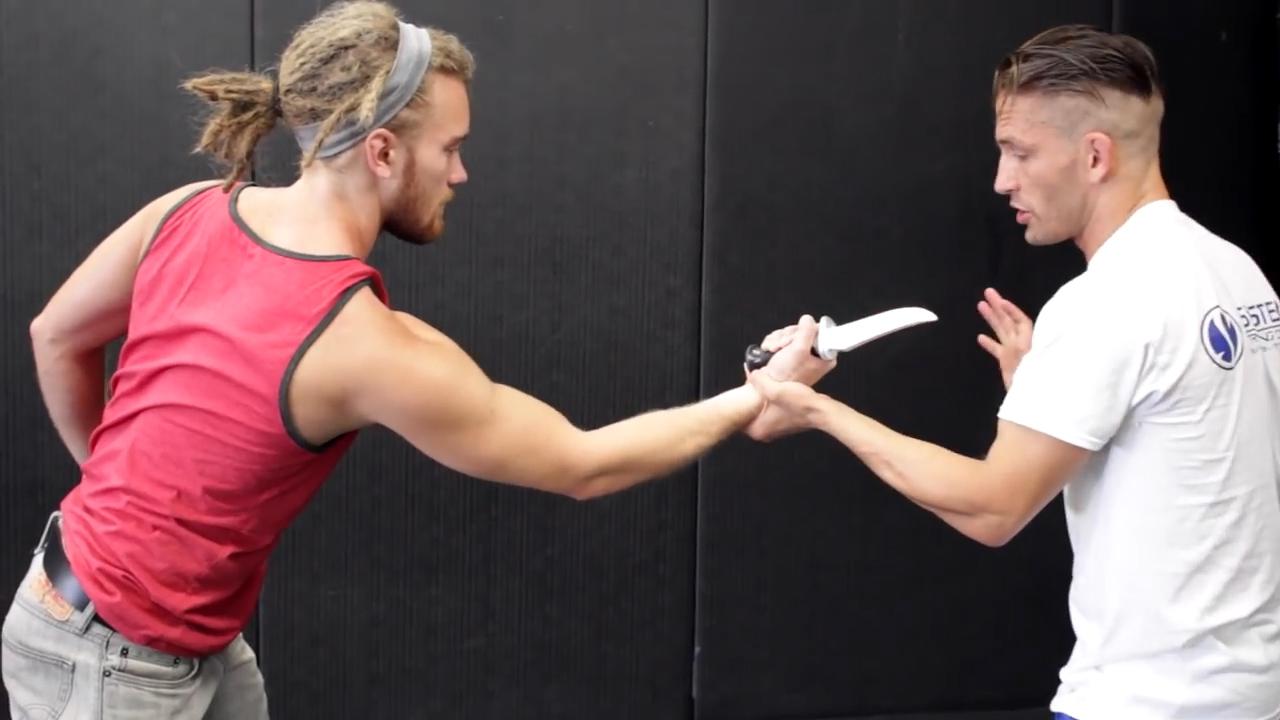

Supplement: Supplementary file 2 — Supplementary Information 2. [file 41598_2023_35190_MOESM2_ESM.zip › test/images/KravMagaKnifeDefenseTechniques278_jpg.rf.6fc8d4a30350b2265162d8c27bbe6bf0.jpg]

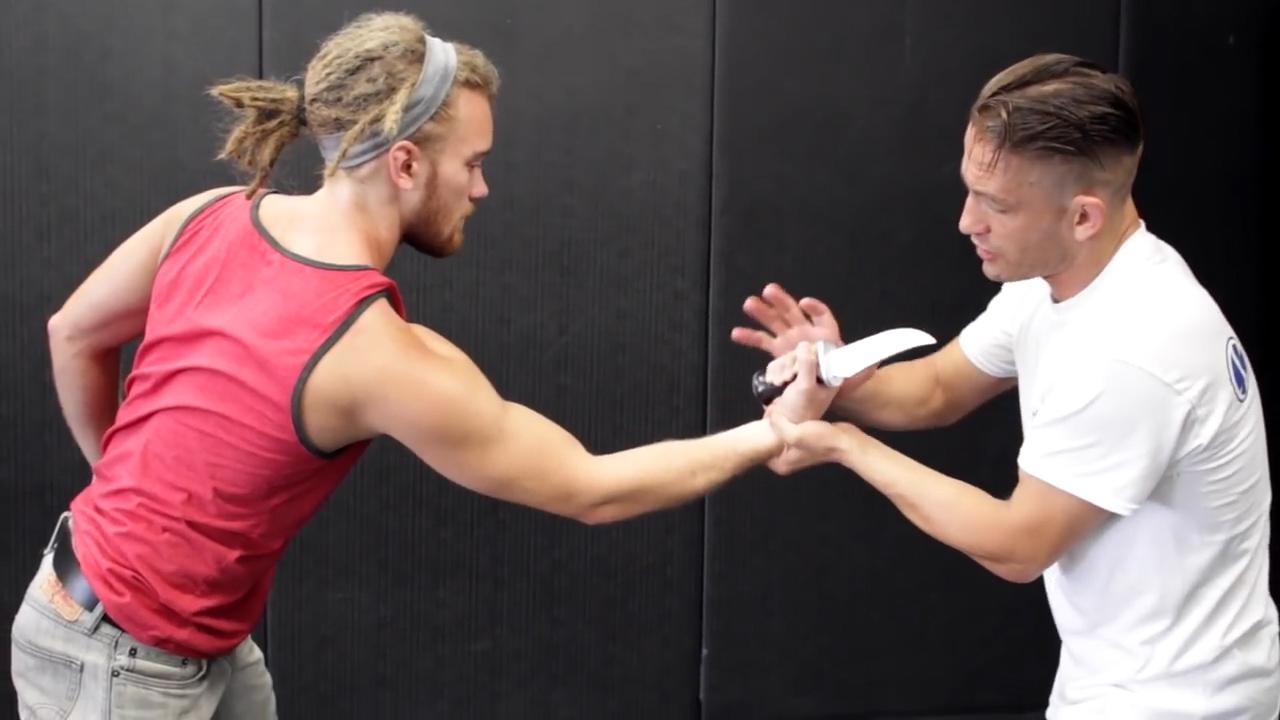

Supplement: Supplementary file 2 — Supplementary Information 2. [file 41598_2023_35190_MOESM2_ESM.zip › test/images/KravMagaKnifeDefenseTechniques284_jpg.rf.0714854ca6839356a102f043cda1a2c7.jpg]

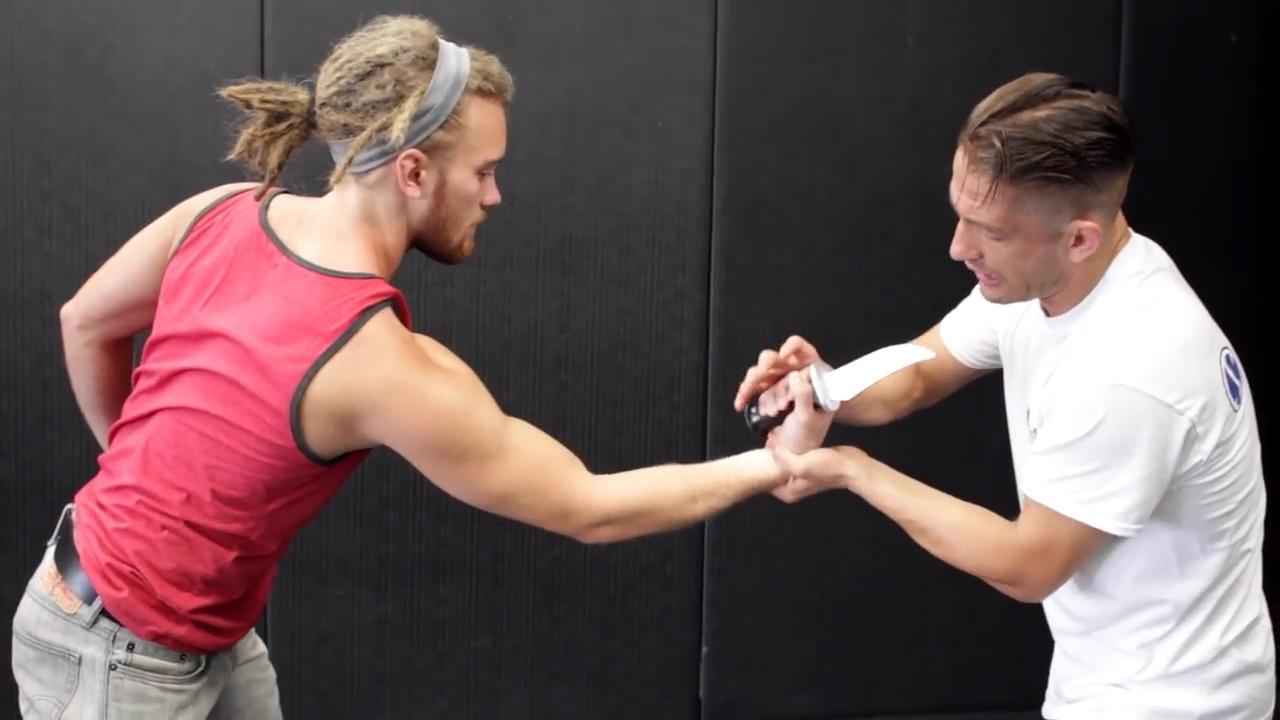

Supplement: Supplementary file 2 — Supplementary Information 2. [file 41598_2023_35190_MOESM2_ESM.zip › test/images/KravMagaKnifeDefenseTechniques286_jpg.rf.f21d4e08ab48eebe7a41601d63f046b0.jpg]

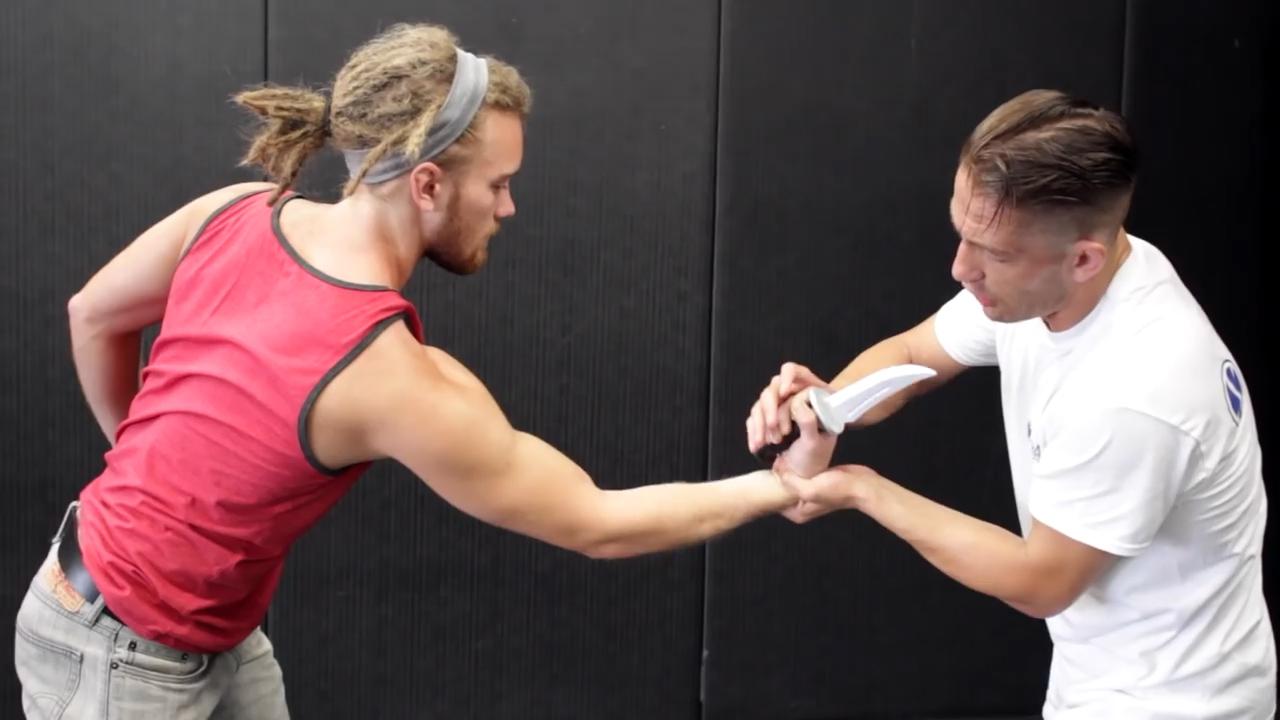

Supplement: Supplementary file 2 — Supplementary Information 2. [file 41598_2023_35190_MOESM2_ESM.zip › test/images/KravMagaKnifeDefenseTechniques288_jpg.rf.88ed6dbb494b35f509cc28a3867e3414.jpg]

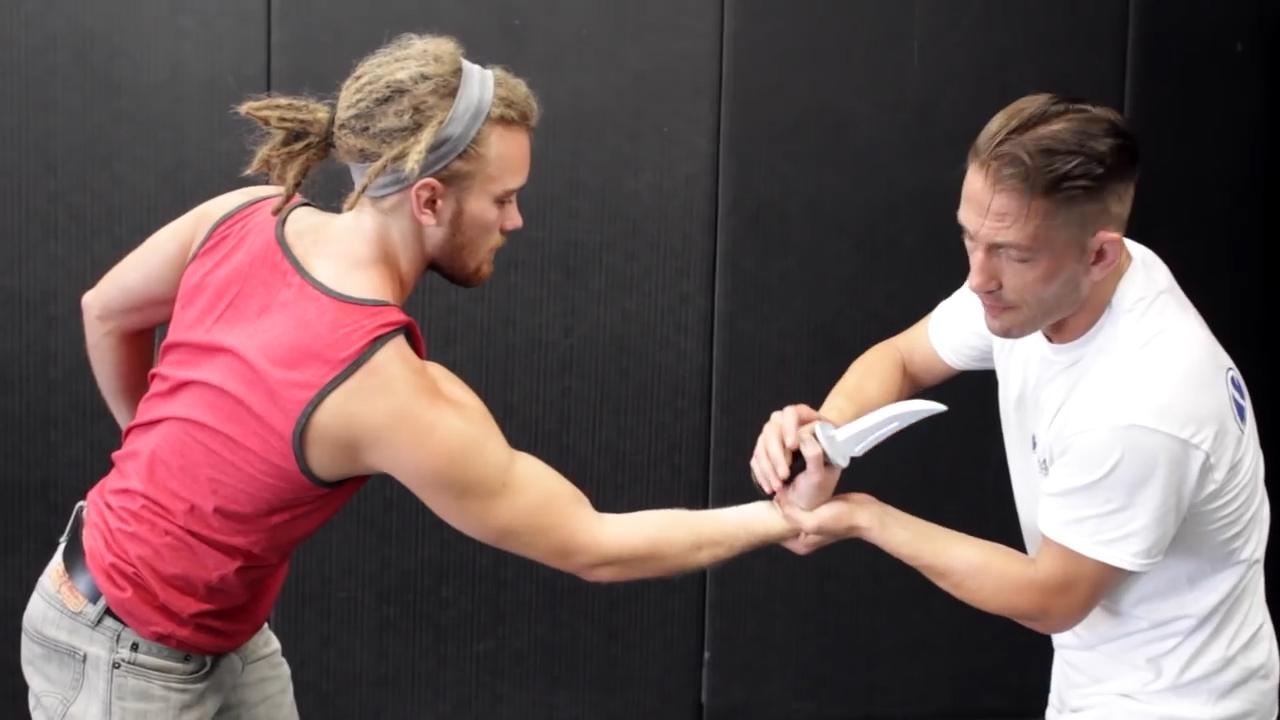

Supplement: Supplementary file 2 — Supplementary Information 2. [file 41598_2023_35190_MOESM2_ESM.zip › test/images/KravMagaKnifeDefenseTechniques290_jpg.rf.c7c6c456716c1591d88533b8a1ab3521.jpg]

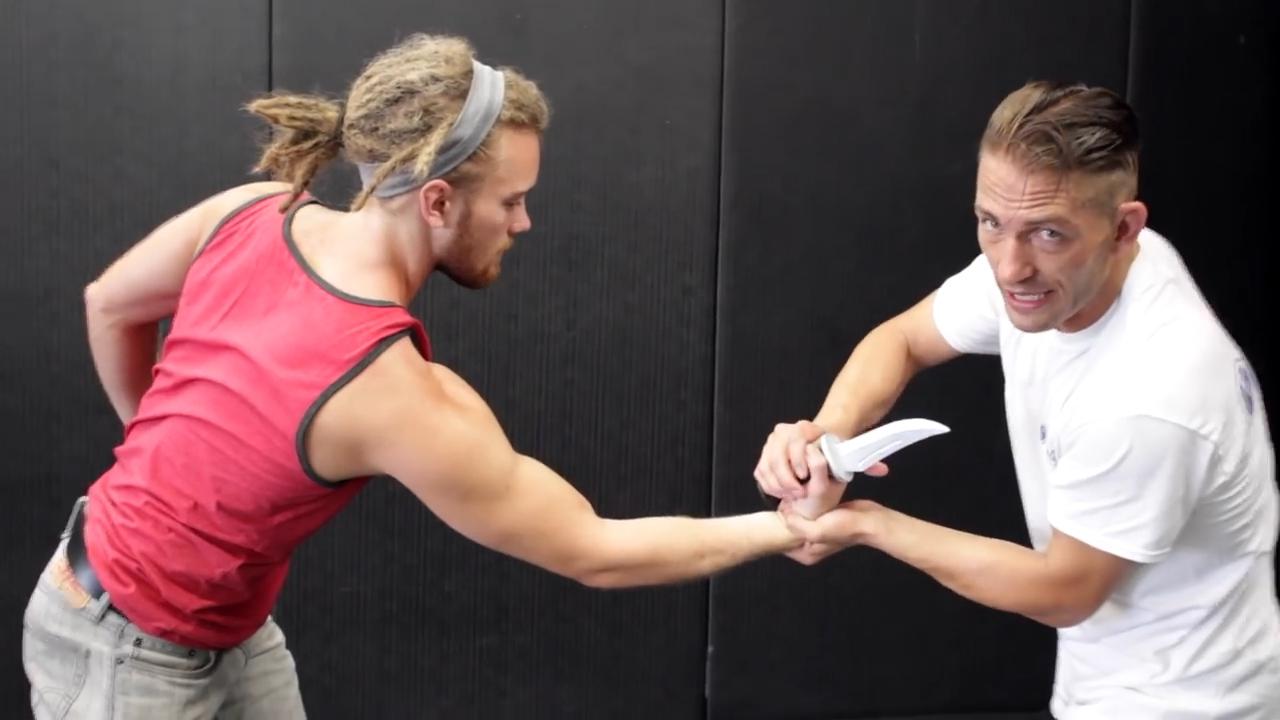

Supplement: Supplementary file 2 — Supplementary Information 2. [file 41598_2023_35190_MOESM2_ESM.zip › test/images/KravMagaKnifeDefenseTechniques292_jpg.rf.2911b5d7570590233c4ba08990ef3669.jpg]

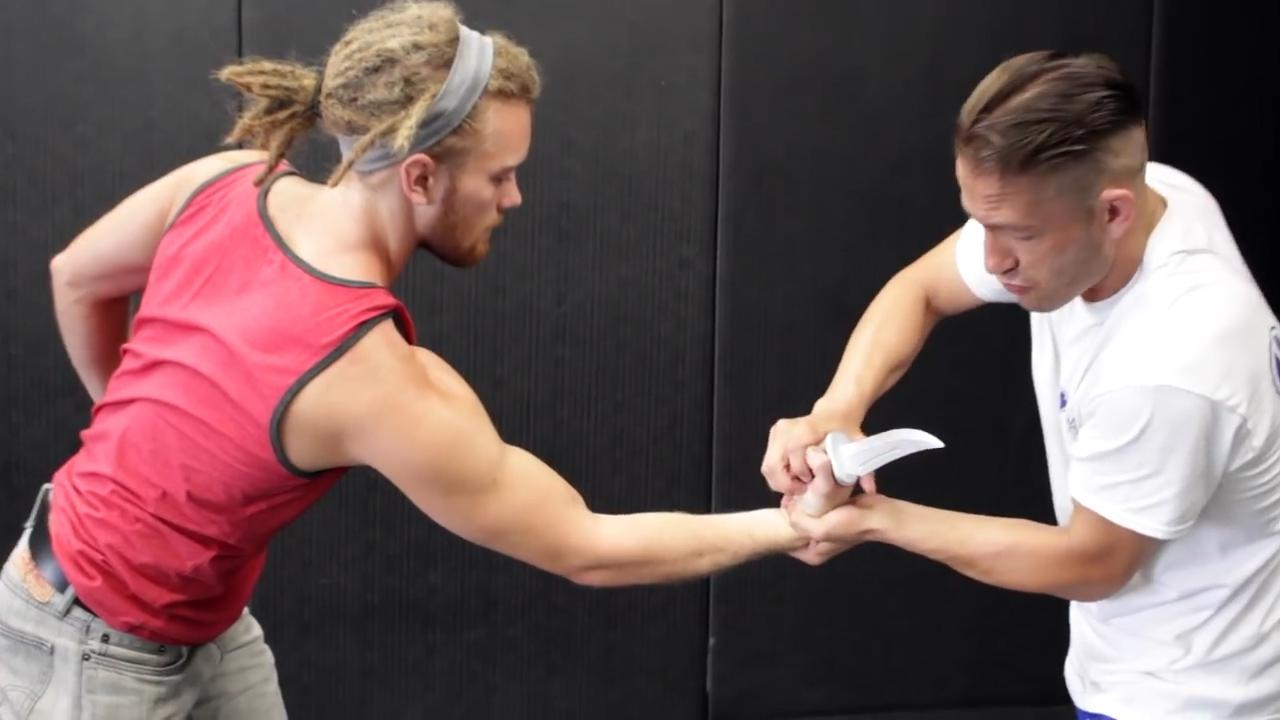

Supplement: Supplementary file 2 — Supplementary Information 2. [file 41598_2023_35190_MOESM2_ESM.zip › test/images/KravMagaKnifeDefenseTechniques294_jpg.rf.d1198e41bb9a3d0acb8f1a28d239eb83.jpg]

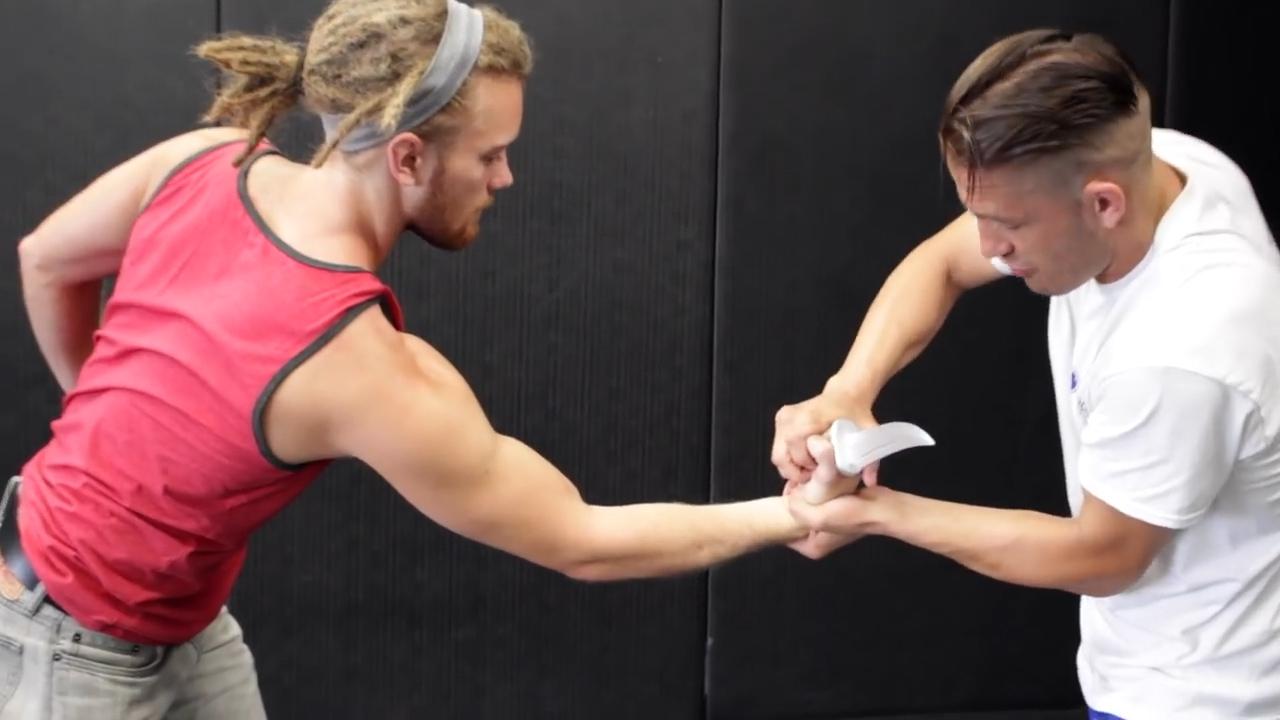

Supplement: Supplementary file 2 — Supplementary Information 2. [file 41598_2023_35190_MOESM2_ESM.zip › test/images/KravMagaKnifeDefenseTechniques295_jpg.rf.5e7e2cd71d7fc9b4926f38d324c94096.jpg]

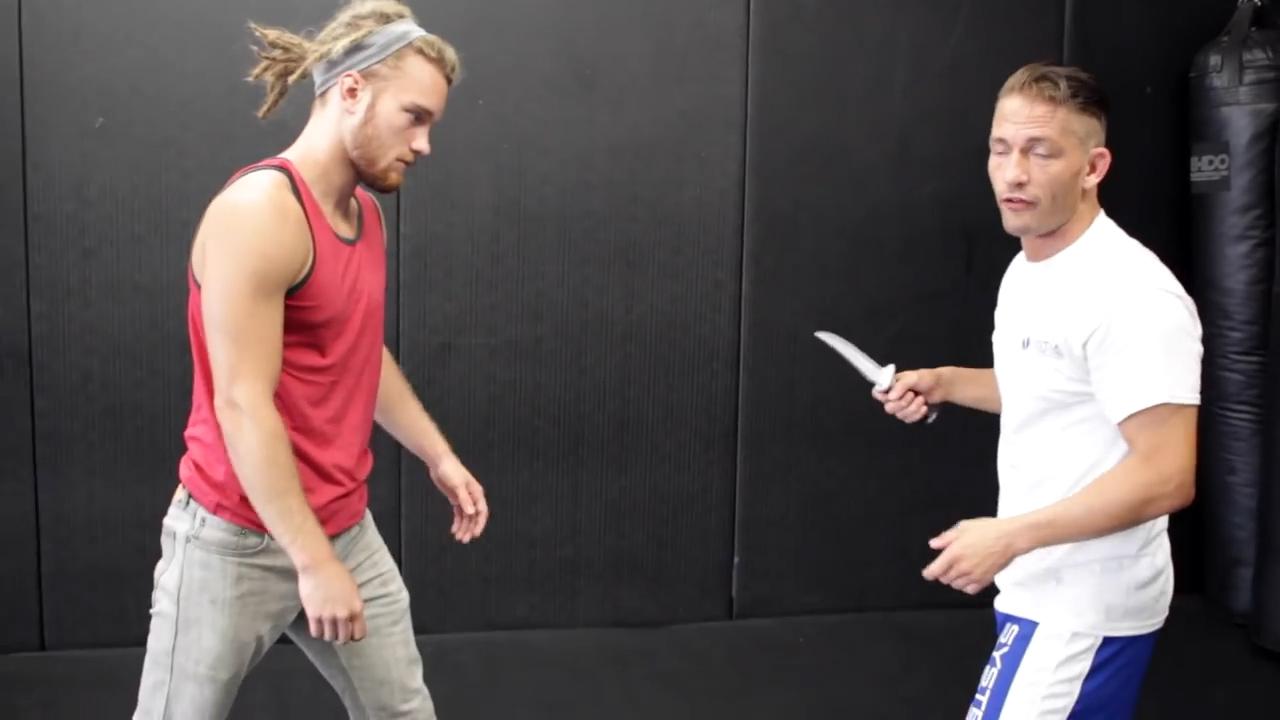

Supplement: Supplementary file 2 — Supplementary Information 2. [file 41598_2023_35190_MOESM2_ESM.zip › test/images/KravMagaKnifeDefenseTechniques316_jpg.rf.67a5b0b7de3d712e3ff0986559701fd5.jpg]

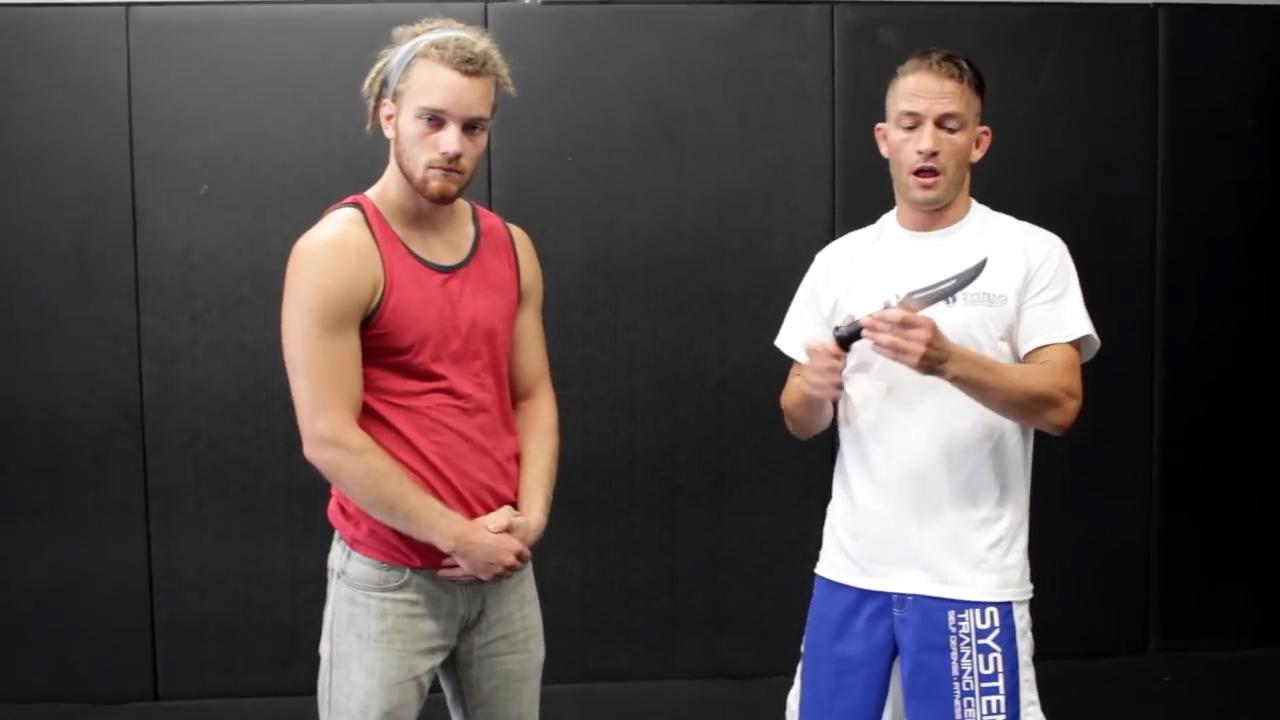

Supplement: Supplementary file 2 — Supplementary Information 2. [file 41598_2023_35190_MOESM2_ESM.zip › test/images/KravMagaKnifeDefenseTechniques363_jpg.rf.5157586cdf8a96b39b4c88adcd3d369e.jpg]

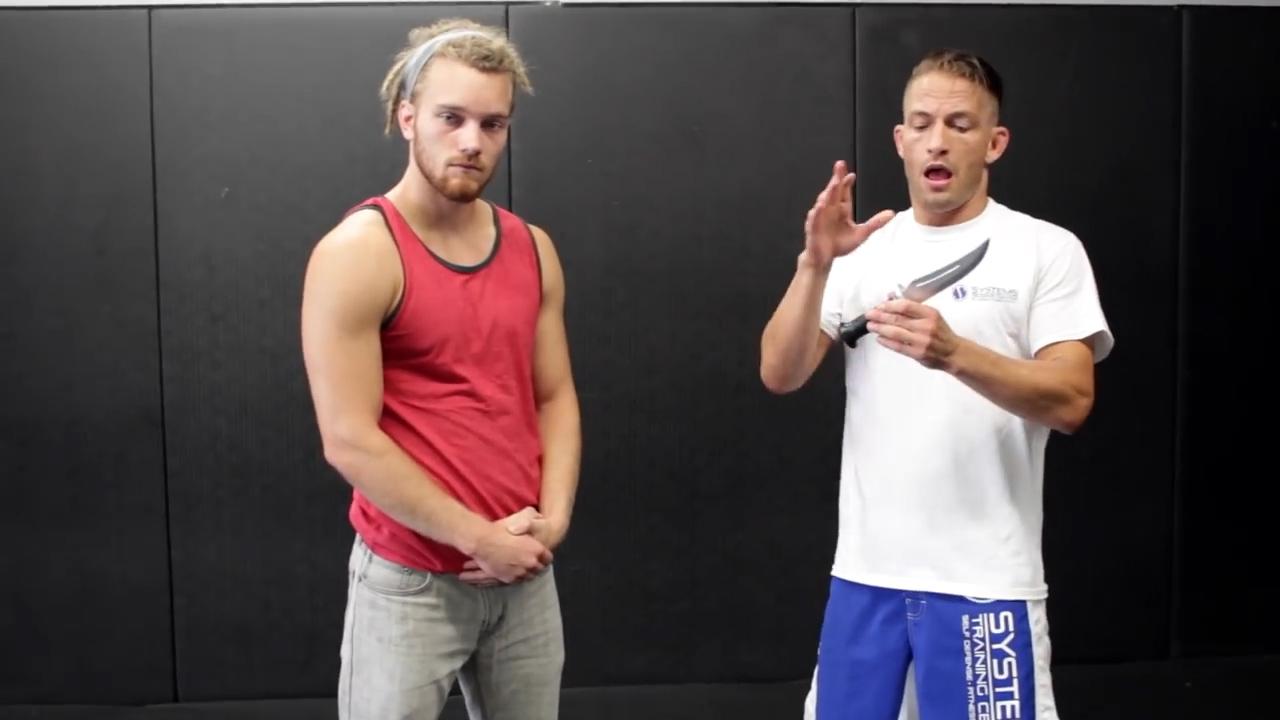

Supplement: Supplementary file 2 — Supplementary Information 2. [file 41598_2023_35190_MOESM2_ESM.zip › test/images/KravMagaKnifeDefenseTechniques365_jpg.rf.db00a49642d5fb216dc714454df37b26.jpg]

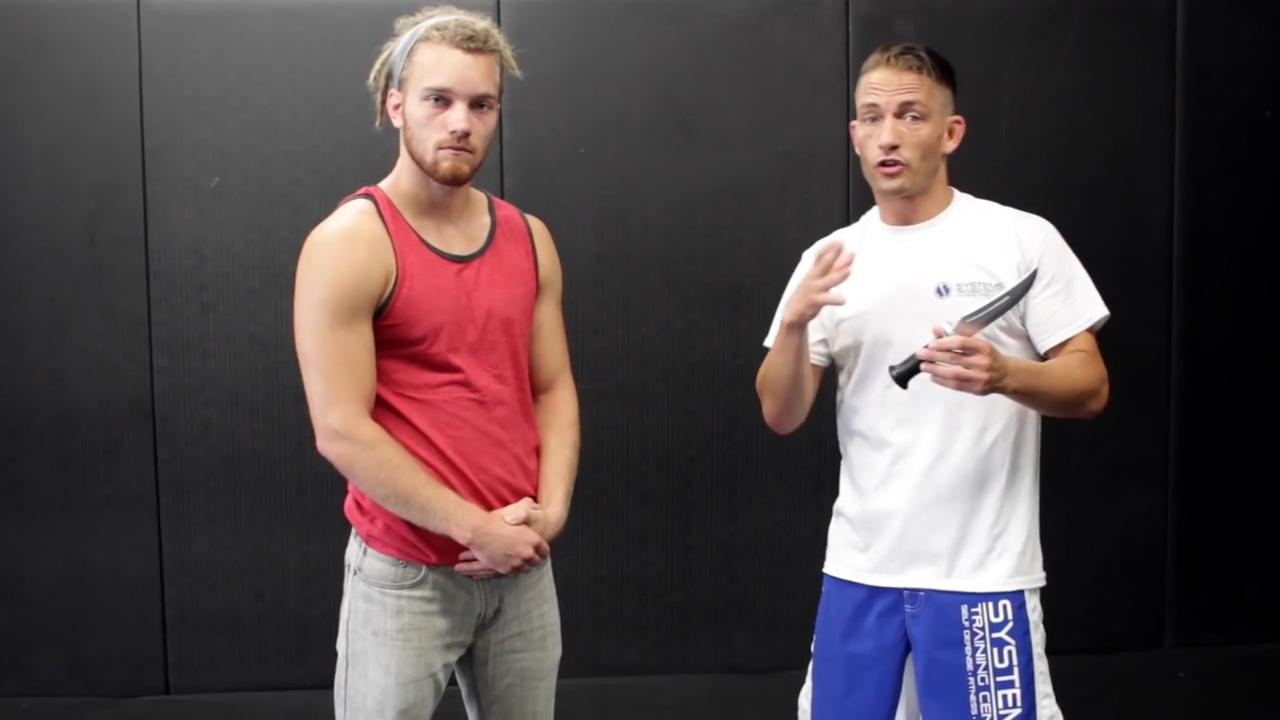

Supplement: Supplementary file 2 — Supplementary Information 2. [file 41598_2023_35190_MOESM2_ESM.zip › test/images/KravMagaKnifeDefenseTechniques370_jpg.rf.b7289adb95e0ad6f2ad46e20a92246c9.jpg]

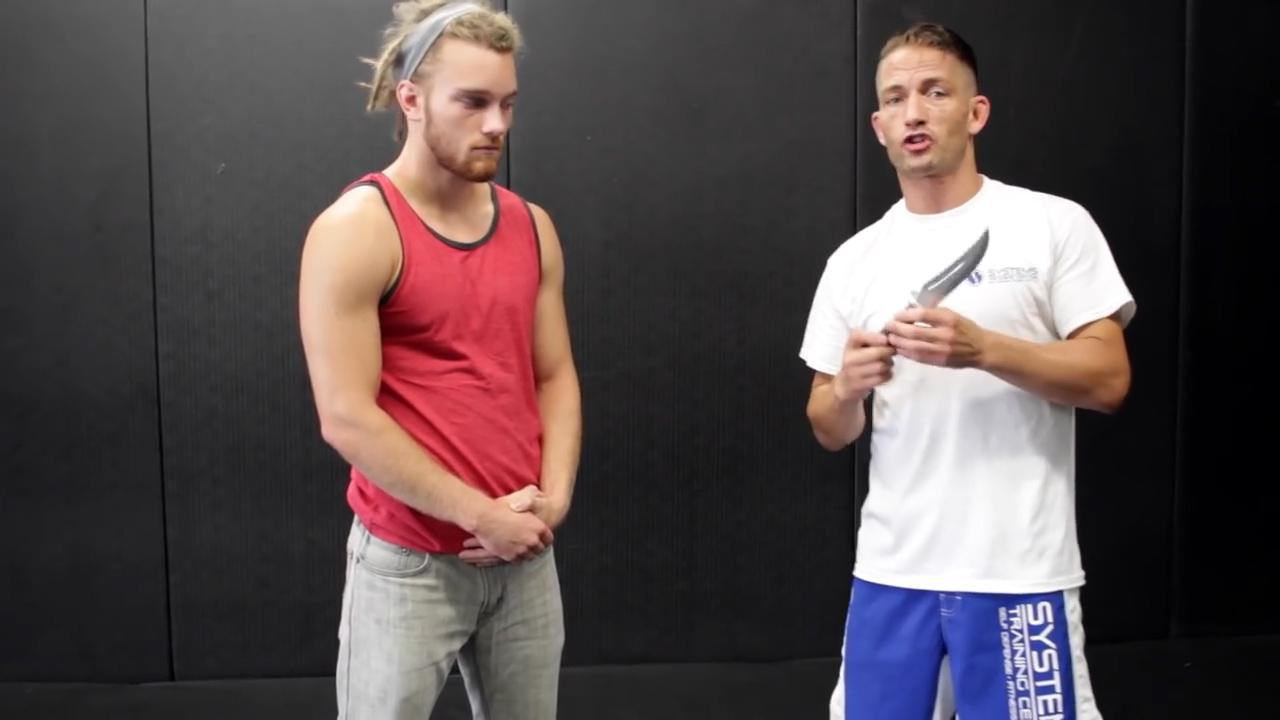

Supplement: Supplementary file 2 — Supplementary Information 2. [file 41598_2023_35190_MOESM2_ESM.zip › test/images/KravMagaKnifeDefenseTechniques377_jpg.rf.20d21165b36408c6365f50fbdd8f85ba.jpg]

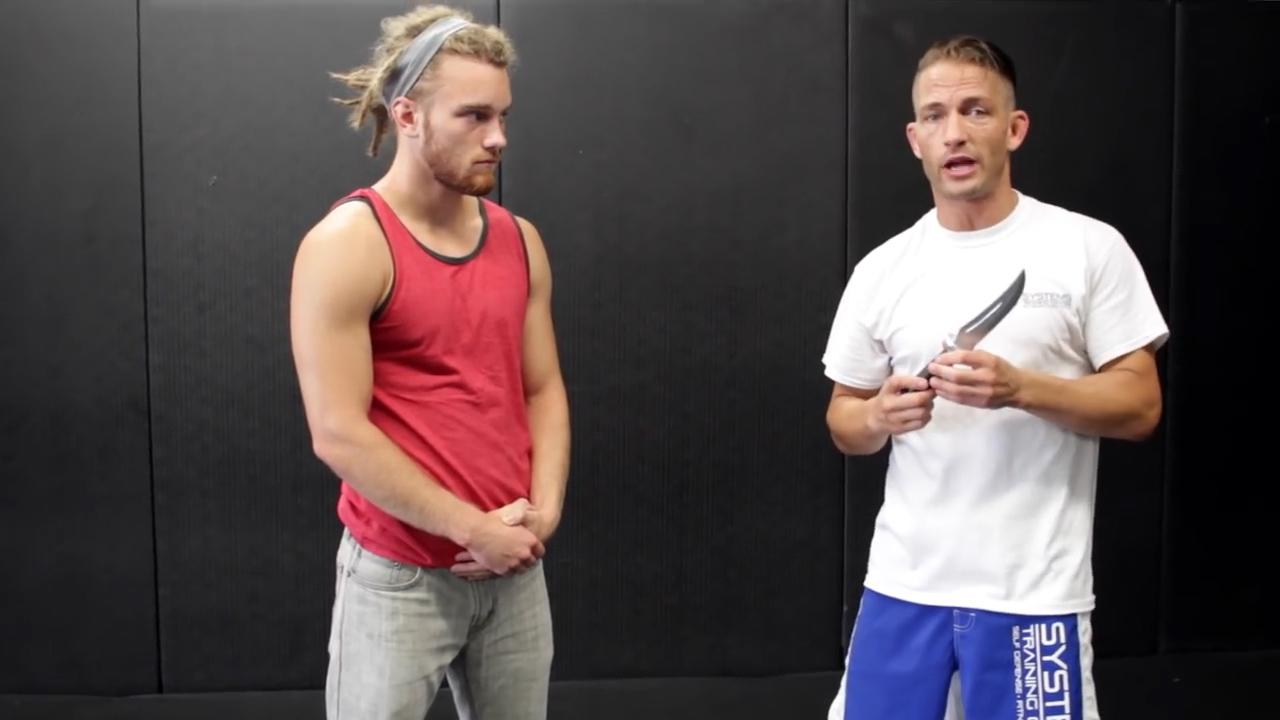

Supplement: Supplementary file 2 — Supplementary Information 2. [file 41598_2023_35190_MOESM2_ESM.zip › test/images/KravMagaKnifeDefenseTechniques380_jpg.rf.327e2bc6a70b947e910721f05928c5a8.jpg]

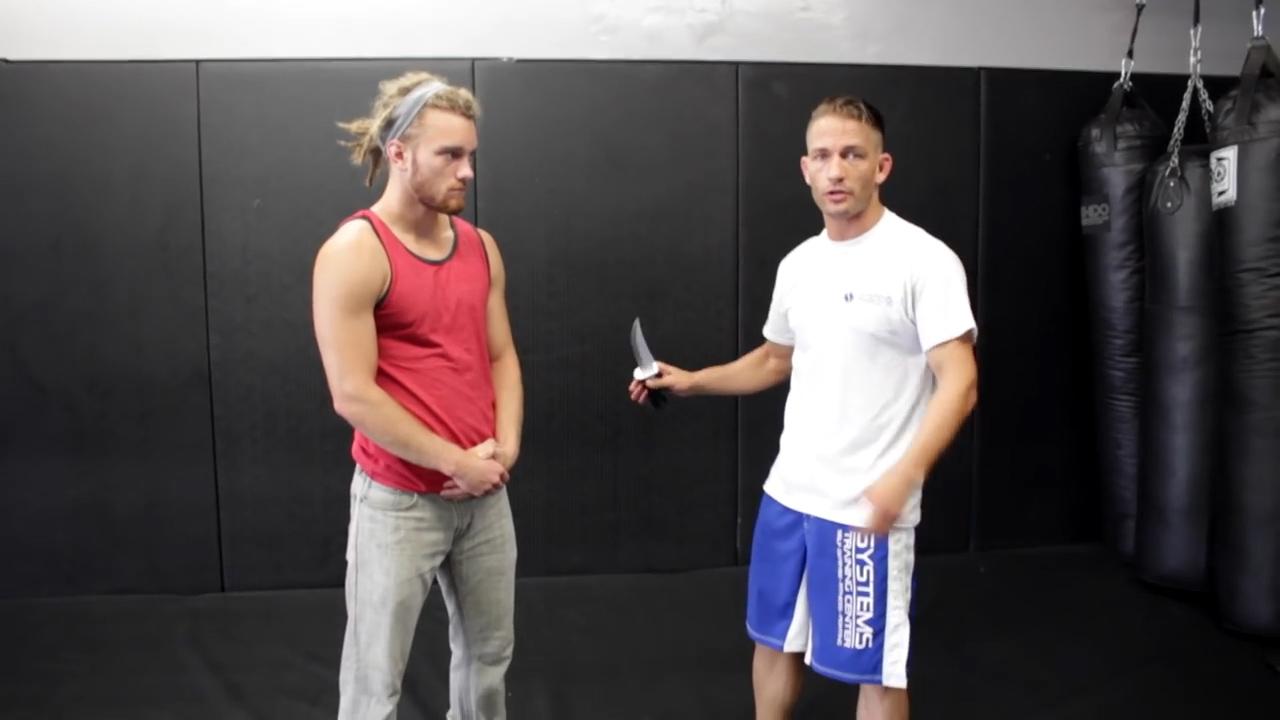

Supplement: Supplementary file 2 — Supplementary Information 2. [file 41598_2023_35190_MOESM2_ESM.zip › test/images/KravMagaKnifeDefenseTechniques398_jpg.rf.0098bf8bebf4d84713d15d975bfebd32.jpg]

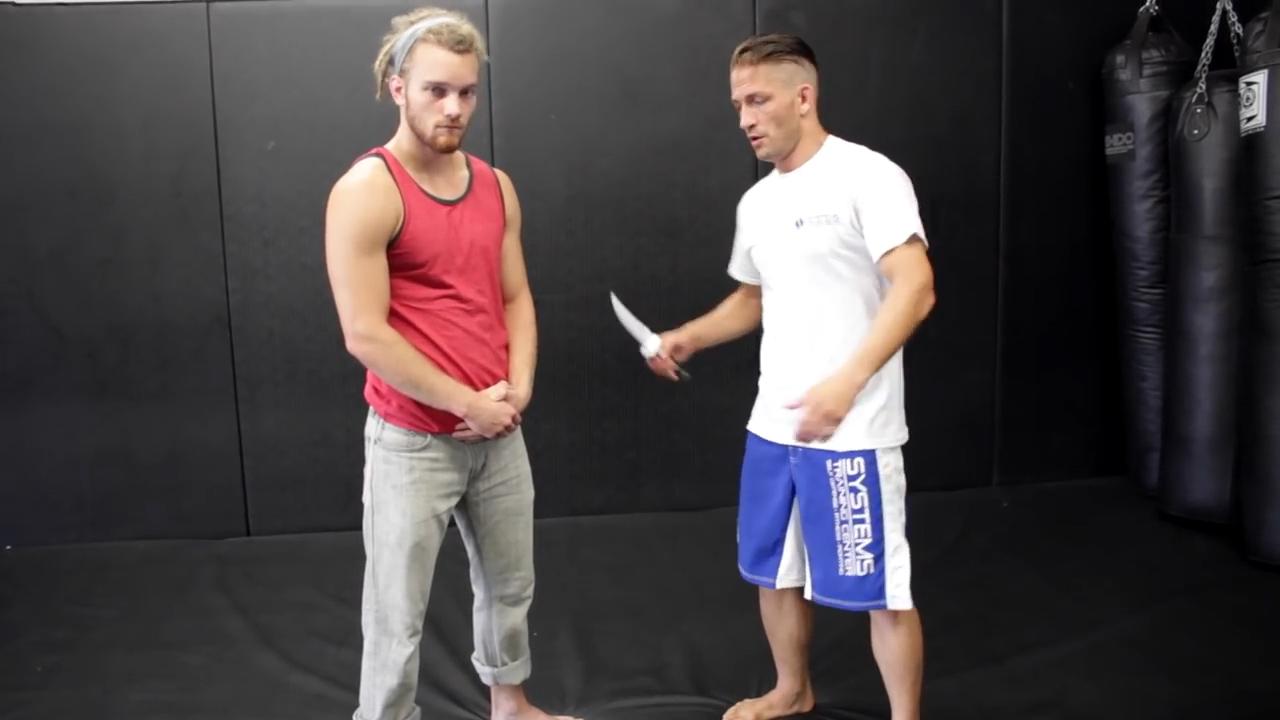

Supplement: Supplementary file 2 — Supplementary Information 2. [file 41598_2023_35190_MOESM2_ESM.zip › test/images/KravMagaKnifeDefenseTechniques416_jpg.rf.7db4fa160882f4a4a952efbce436ad23.jpg]

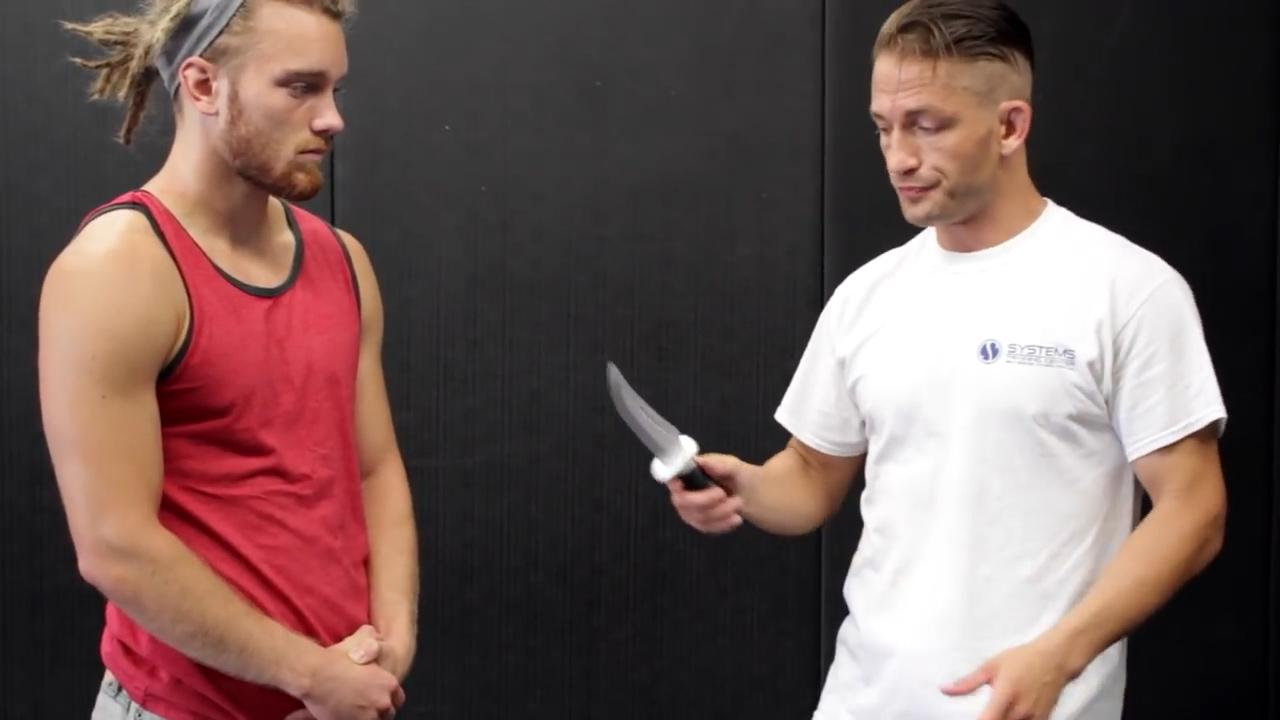

Supplement: Supplementary file 2 — Supplementary Information 2. [file 41598_2023_35190_MOESM2_ESM.zip › test/images/KravMagaKnifeDefenseTechniques442_jpg.rf.8d9a0dfb58ba43eff1281b09a221c964.jpg]

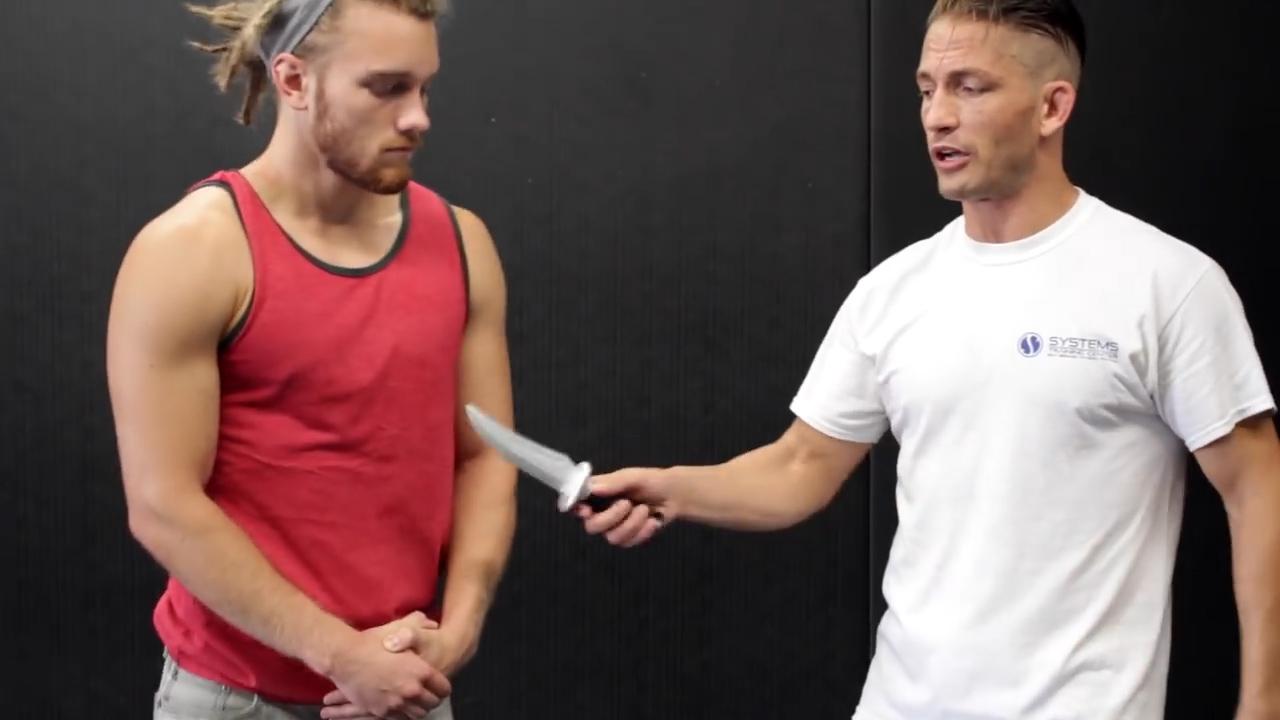

Supplement: Supplementary file 2 — Supplementary Information 2. [file 41598_2023_35190_MOESM2_ESM.zip › test/images/KravMagaKnifeDefenseTechniques453_jpg.rf.fc61dff43b9c3c7cb0a62c7d25a34bfd.jpg]

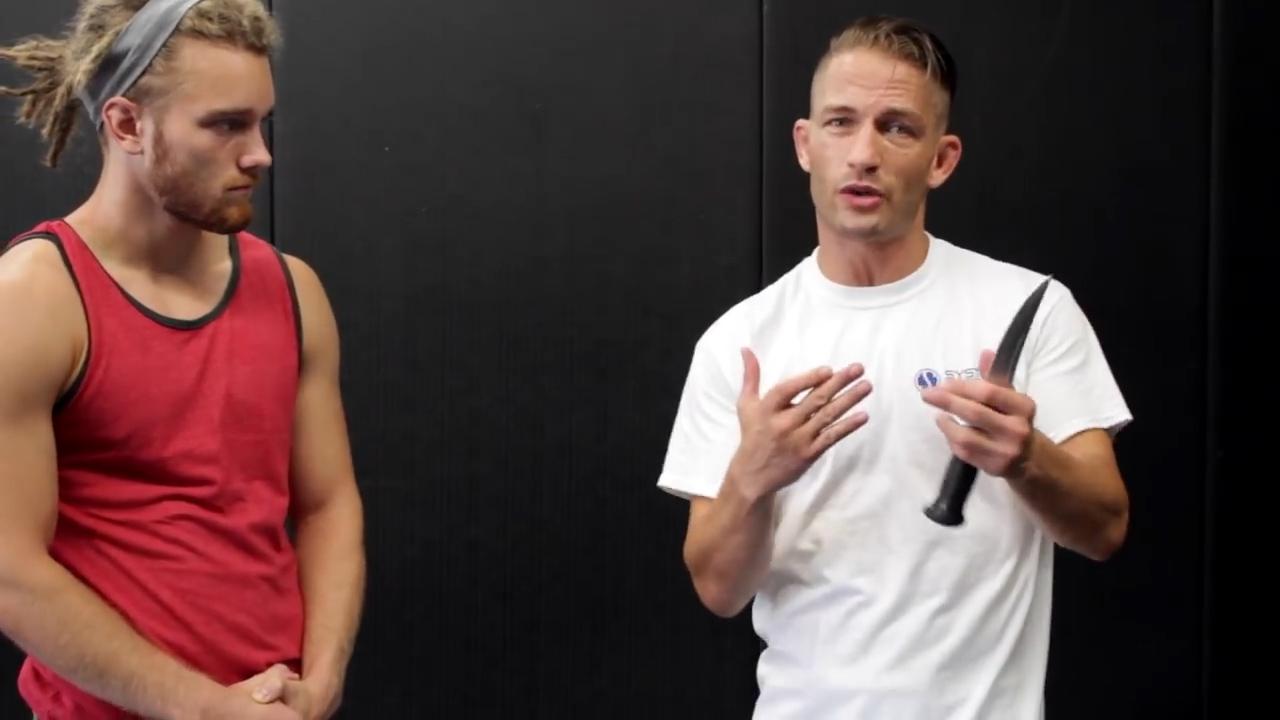

Supplement: Supplementary file 2 — Supplementary Information 2. [file 41598_2023_35190_MOESM2_ESM.zip › test/images/KravMagaKnifeDefenseTechniques474_jpg.rf.bdf1e47d7c95ac8d355dc46a5302839a.jpg]

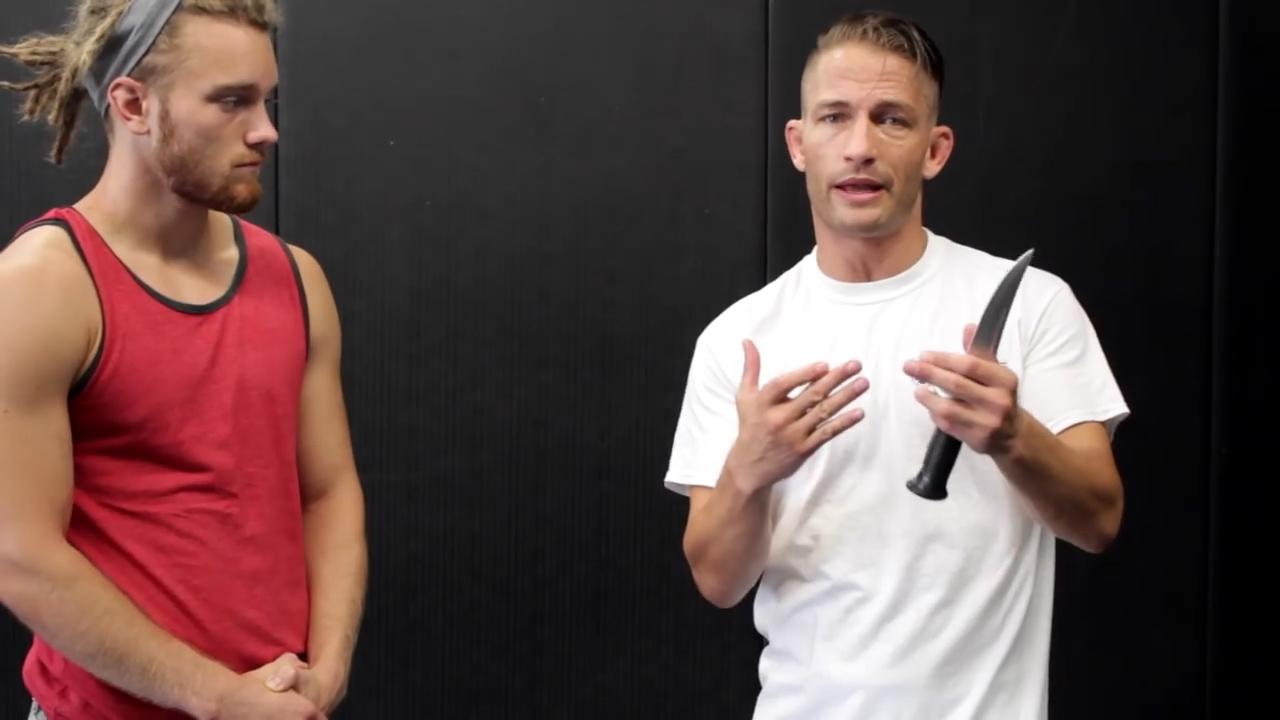

Supplement: Supplementary file 2 — Supplementary Information 2. [file 41598_2023_35190_MOESM2_ESM.zip › test/images/KravMagaKnifeDefenseTechniques476_jpg.rf.8f5df313422771d84224ac5fda6052b3.jpg]

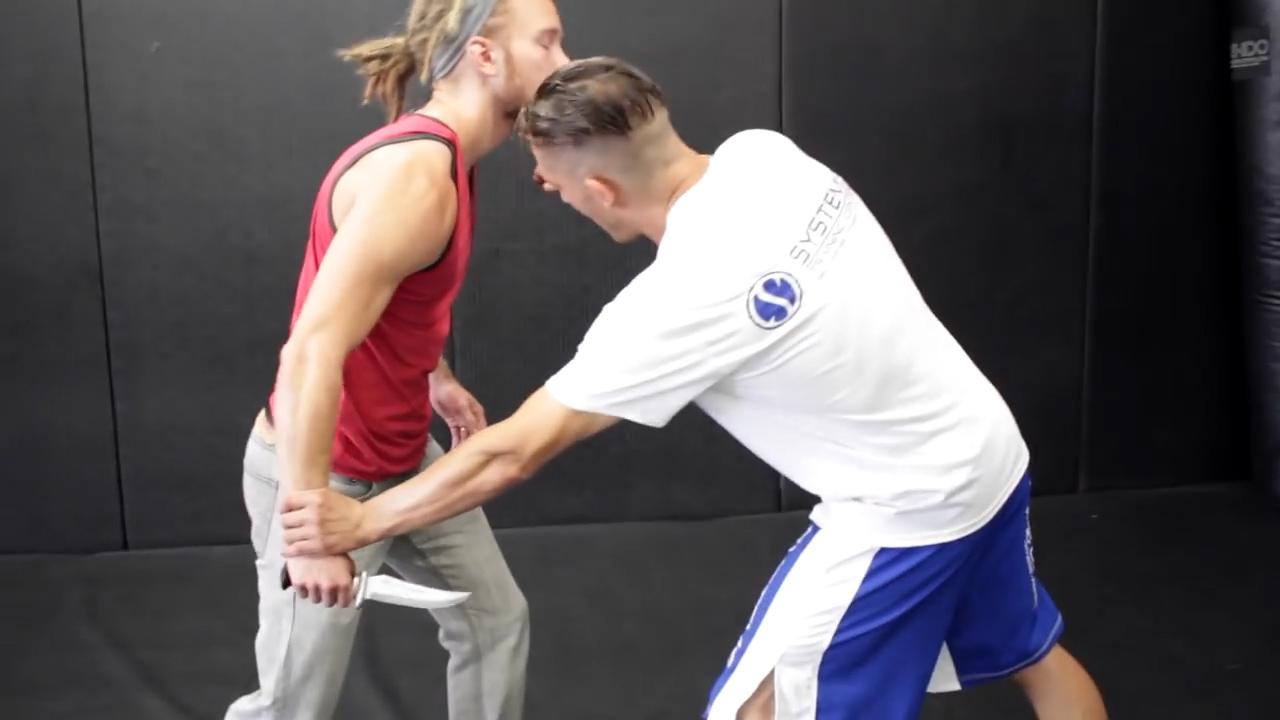

Supplement: Supplementary file 2 — Supplementary Information 2. [file 41598_2023_35190_MOESM2_ESM.zip › test/images/KravMagaKnifeDefenseTechniques585_jpg.rf.b9a375851db0879af027c3b26a1be187.jpg]

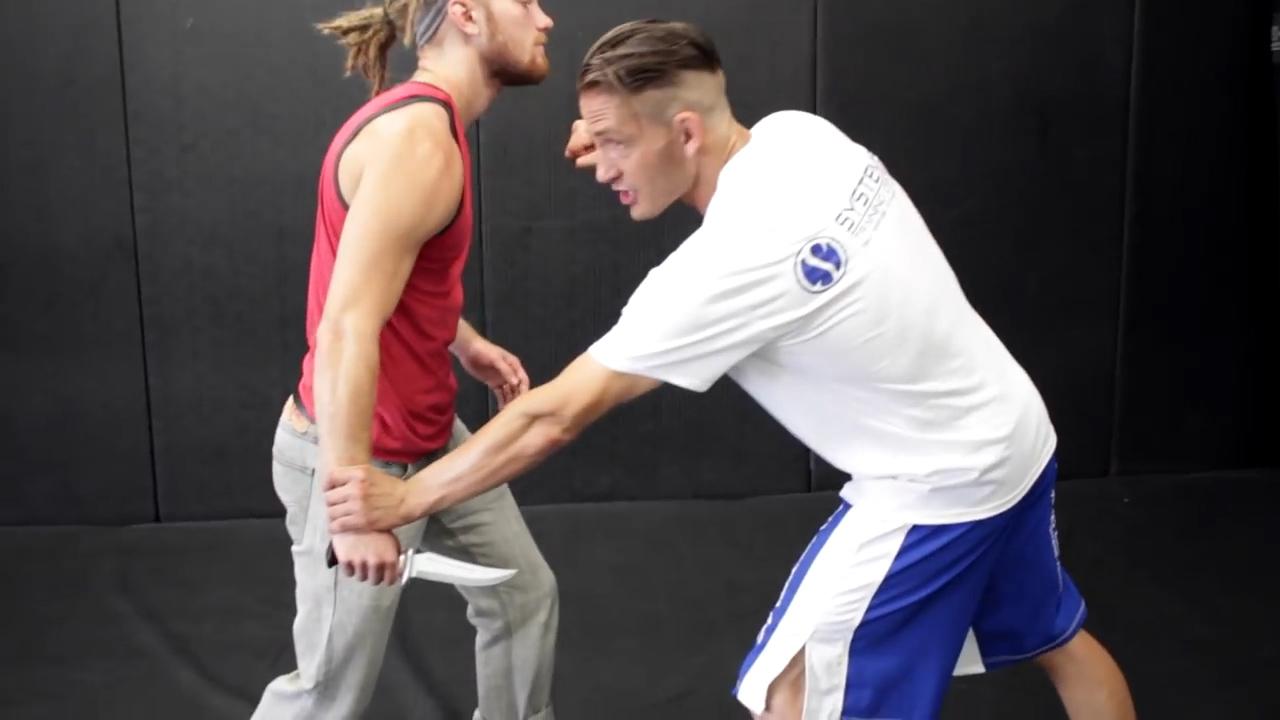

Supplement: Supplementary file 2 — Supplementary Information 2. [file 41598_2023_35190_MOESM2_ESM.zip › test/images/KravMagaKnifeDefenseTechniques587_jpg.rf.0286360ab5b037a58a62129dc9f44759.jpg]

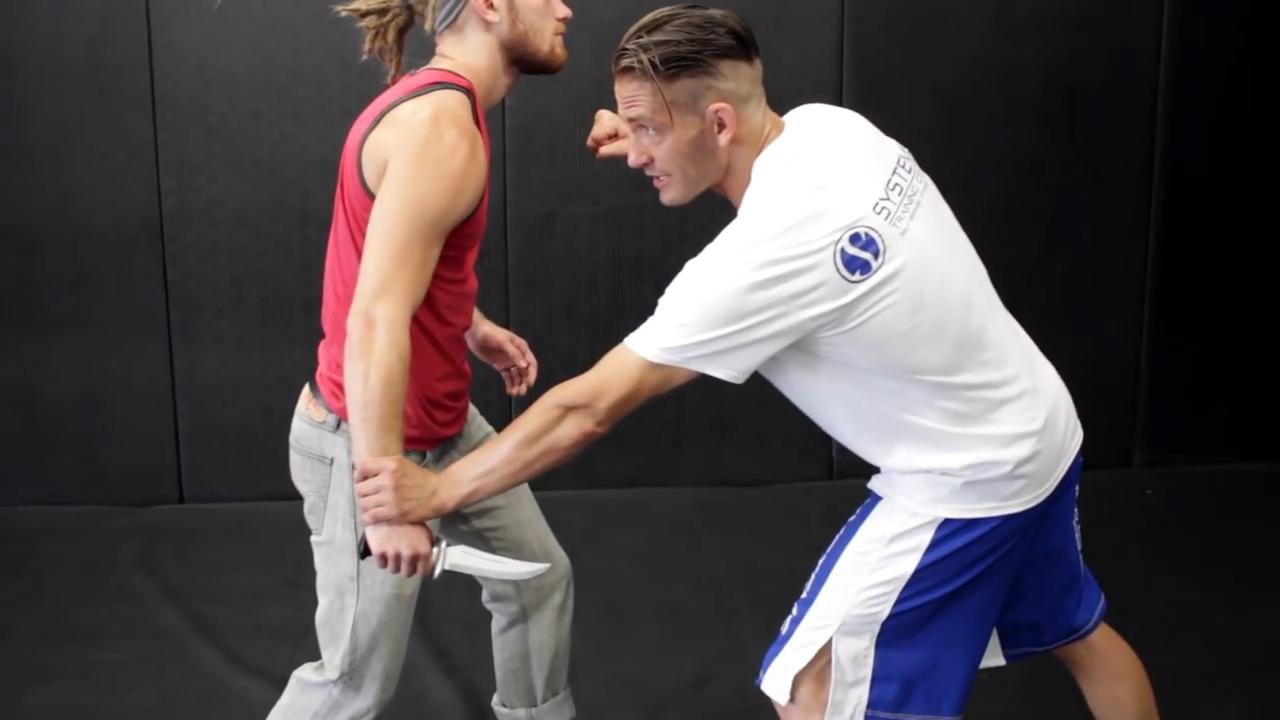

Supplement: Supplementary file 2 — Supplementary Information 2. [file 41598_2023_35190_MOESM2_ESM.zip › test/images/KravMagaKnifeDefenseTechniques590_jpg.rf.c7a7a1a39388e4b9a2b7241f3c922bcb.jpg]

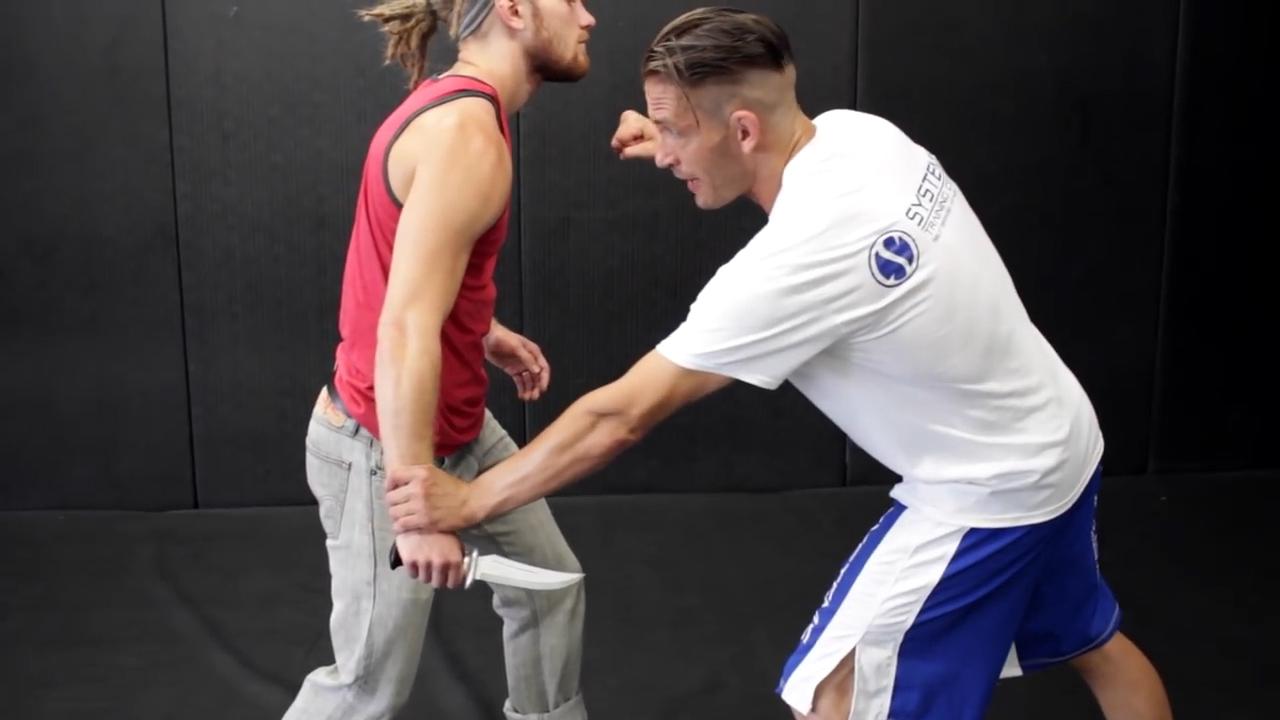

Supplement: Supplementary file 2 — Supplementary Information 2. [file 41598_2023_35190_MOESM2_ESM.zip › test/images/KravMagaKnifeDefenseTechniques594_jpg.rf.fe9c04c448ec29eb31587b29f5766db9.jpg]

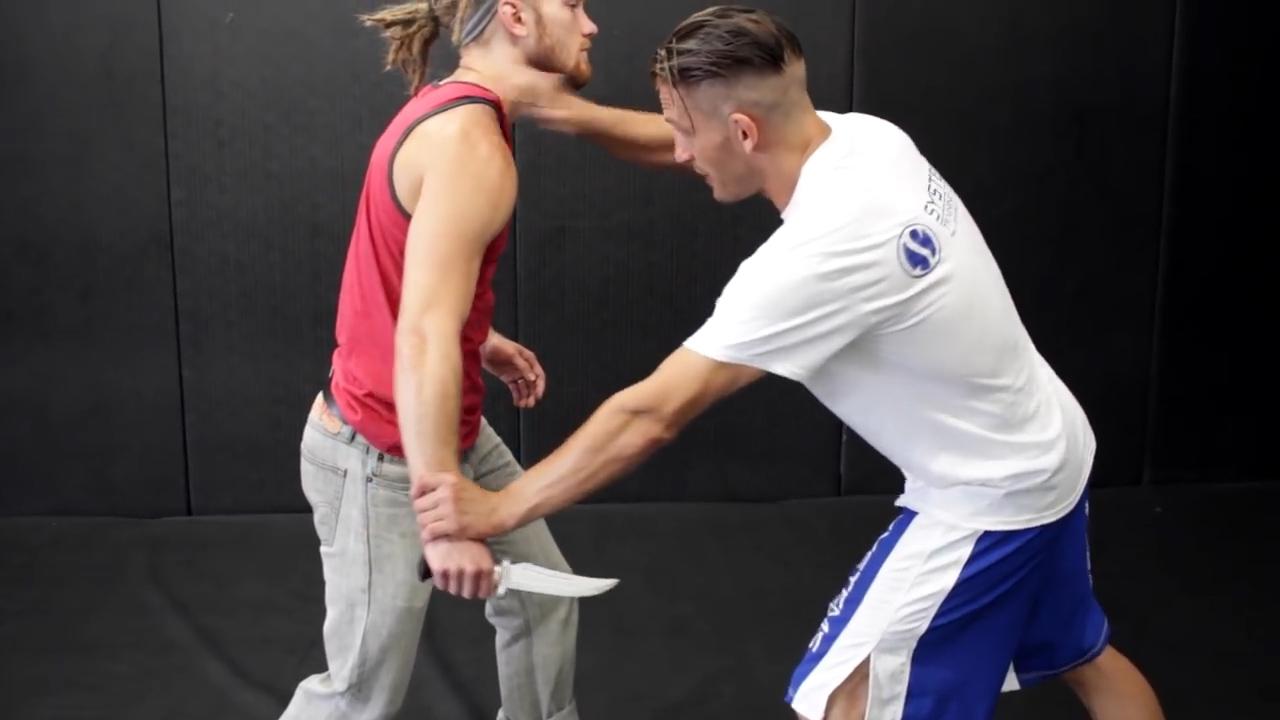

Supplement: Supplementary file 2 — Supplementary Information 2. [file 41598_2023_35190_MOESM2_ESM.zip › test/images/KravMagaKnifeDefenseTechniques597_jpg.rf.e99faabceb7d38436d4d532d896bddfc.jpg]

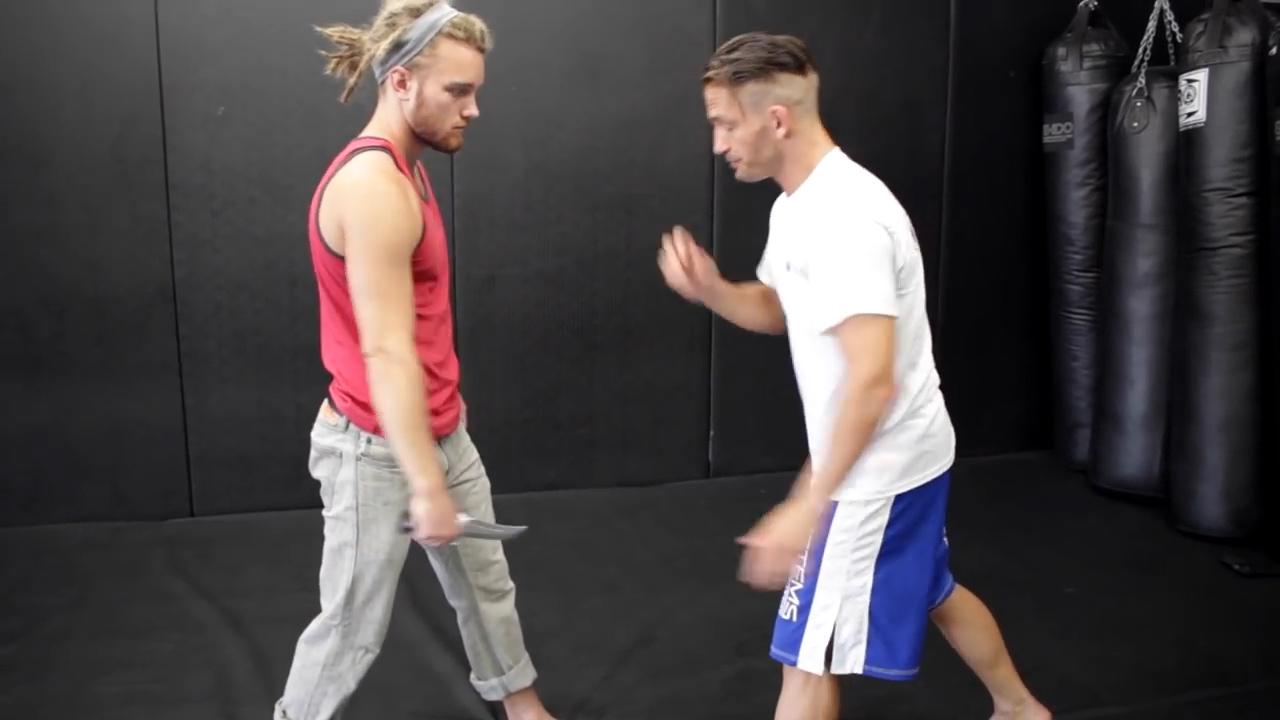

Supplement: Supplementary file 2 — Supplementary Information 2. [file 41598_2023_35190_MOESM2_ESM.zip › test/images/KravMagaKnifeDefenseTechniques624_jpg.rf.c57b03211e0f0dd55dabaaacc6717328.jpg]

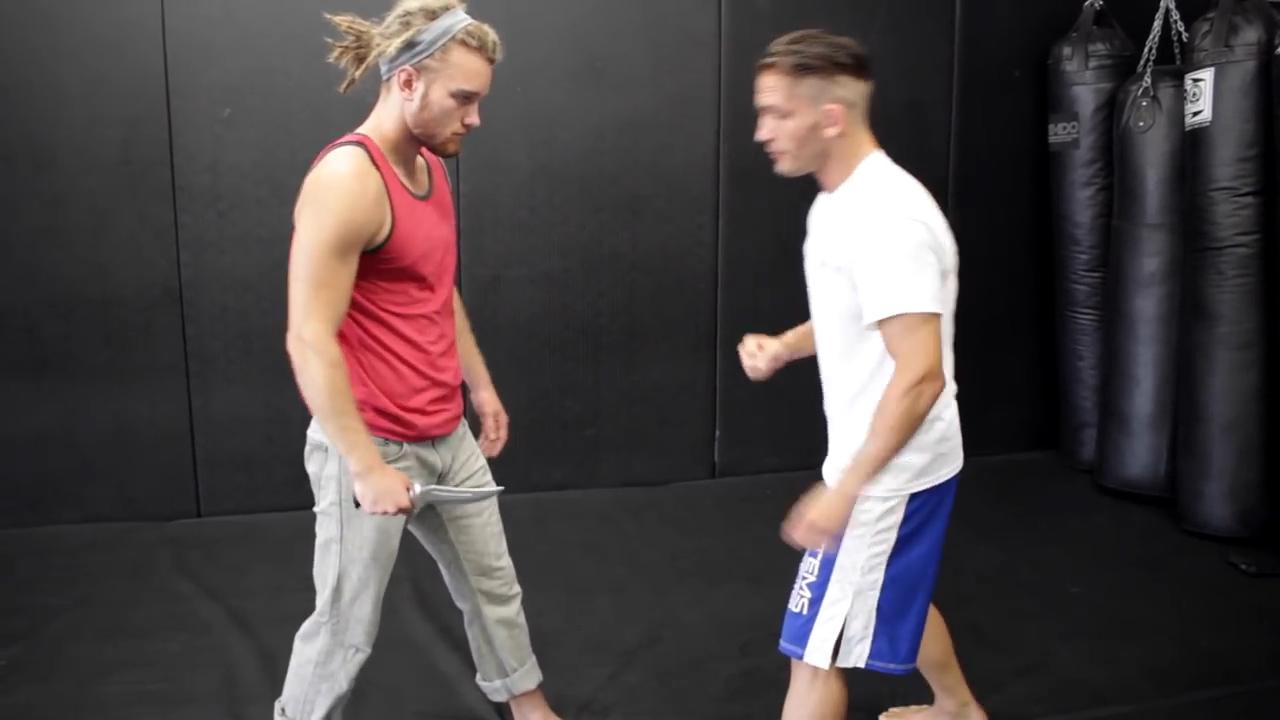

Supplement: Supplementary file 2 — Supplementary Information 2. [file 41598_2023_35190_MOESM2_ESM.zip › test/images/KravMagaKnifeDefenseTechniques626_jpg.rf.a86210beedfccde4d3a847c37df09ab7.jpg]

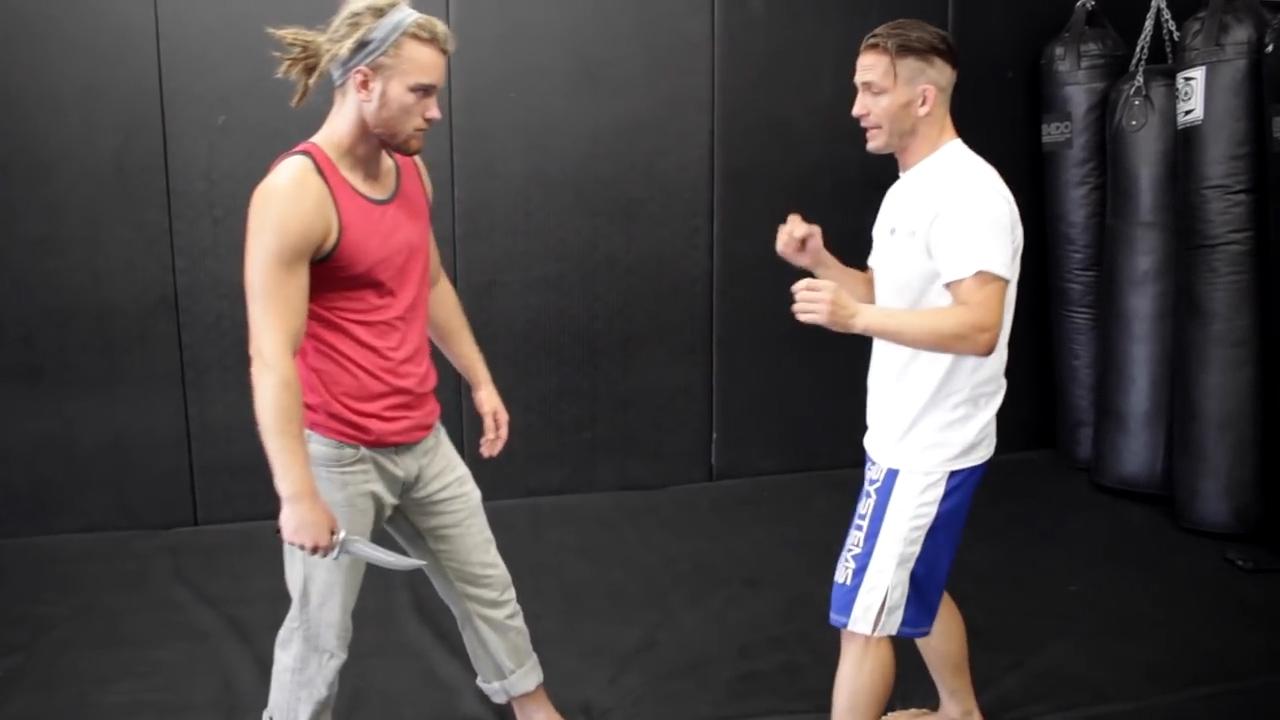

Supplement: Supplementary file 2 — Supplementary Information 2. [file 41598_2023_35190_MOESM2_ESM.zip › test/images/KravMagaKnifeDefenseTechniques628_jpg.rf.48aff7939e2734b281a5061870f3db0c.jpg]

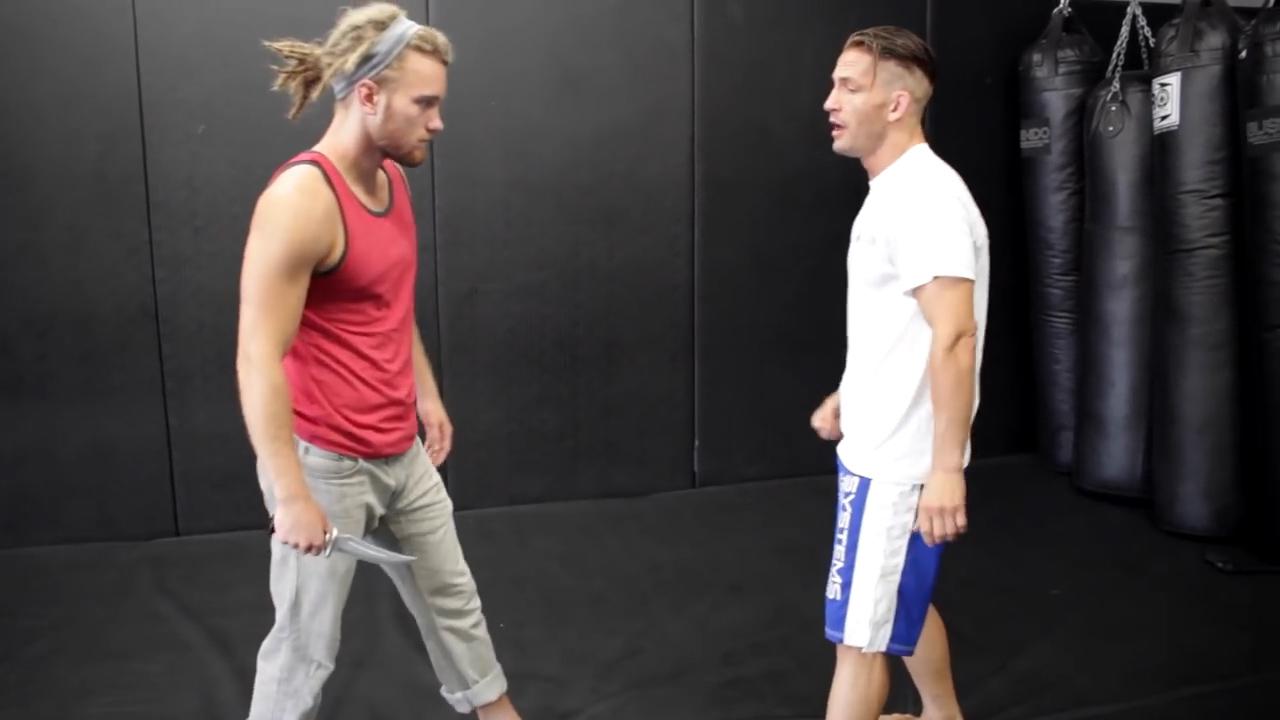

Supplement: Supplementary file 2 — Supplementary Information 2. [file 41598_2023_35190_MOESM2_ESM.zip › test/images/KravMagaKnifeDefenseTechniques630_jpg.rf.54b99e6e729b98940dfc3045d096305d.jpg]

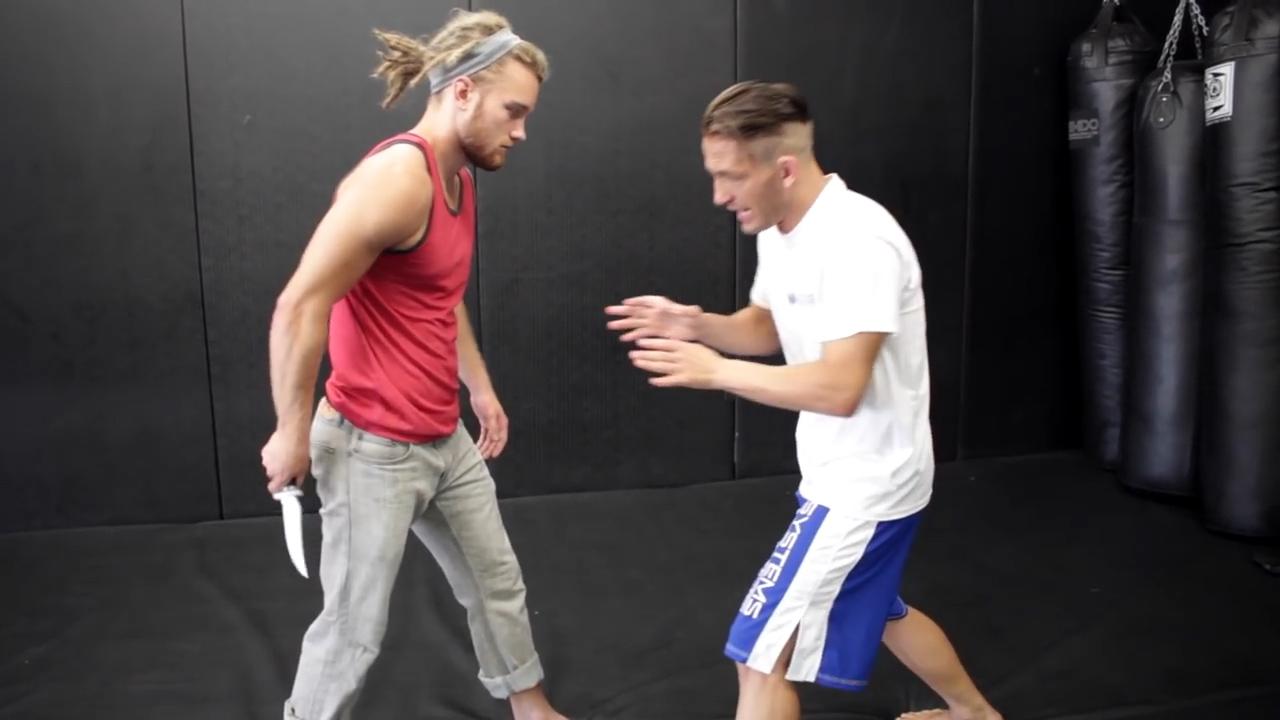

Supplement: Supplementary file 2 — Supplementary Information 2. [file 41598_2023_35190_MOESM2_ESM.zip › test/images/KravMagaKnifeDefenseTechniques655_jpg.rf.02b65a08e099d47c7ab98035fb1c733d.jpg]

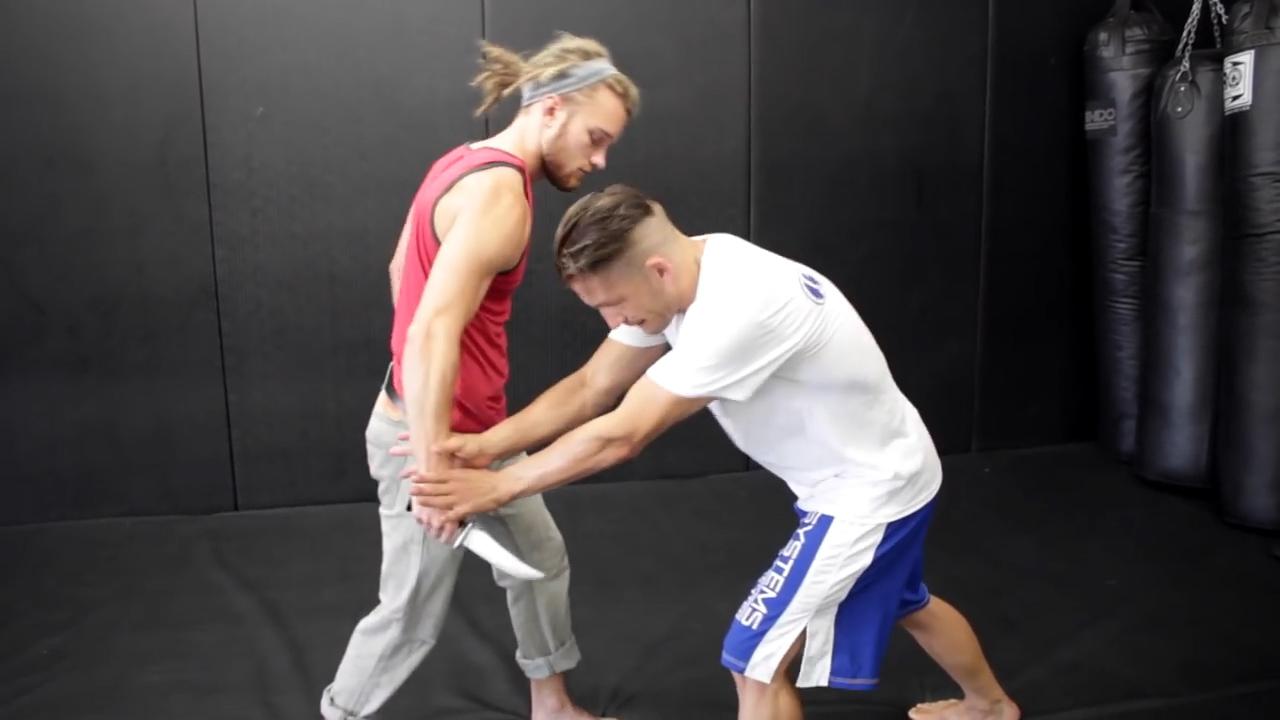

Supplement: Supplementary file 2 — Supplementary Information 2. [file 41598_2023_35190_MOESM2_ESM.zip › test/images/KravMagaKnifeDefenseTechniques658_jpg.rf.c45a37b8a8517c8f87c09ba0cf20a543.jpg]

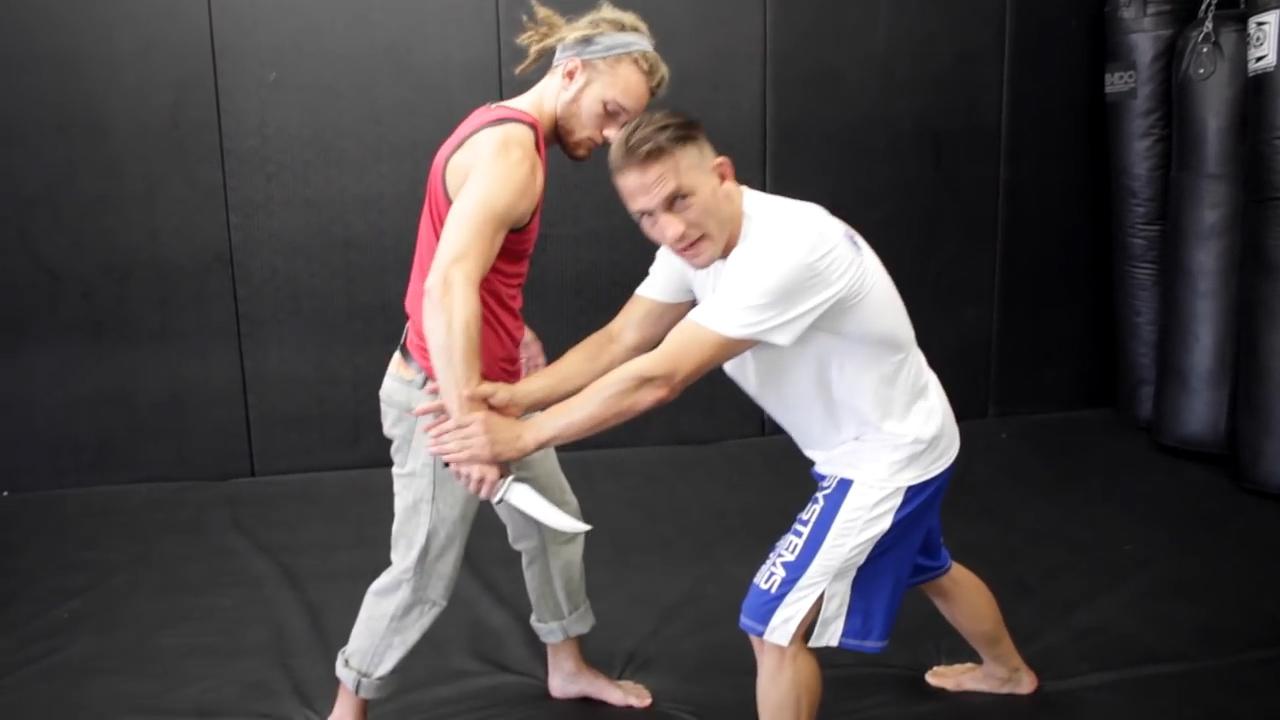

Supplement: Supplementary file 2 — Supplementary Information 2. [file 41598_2023_35190_MOESM2_ESM.zip › test/images/KravMagaKnifeDefenseTechniques660_jpg.rf.74b8739d411636d2b3dcb2c4cca50540.jpg]

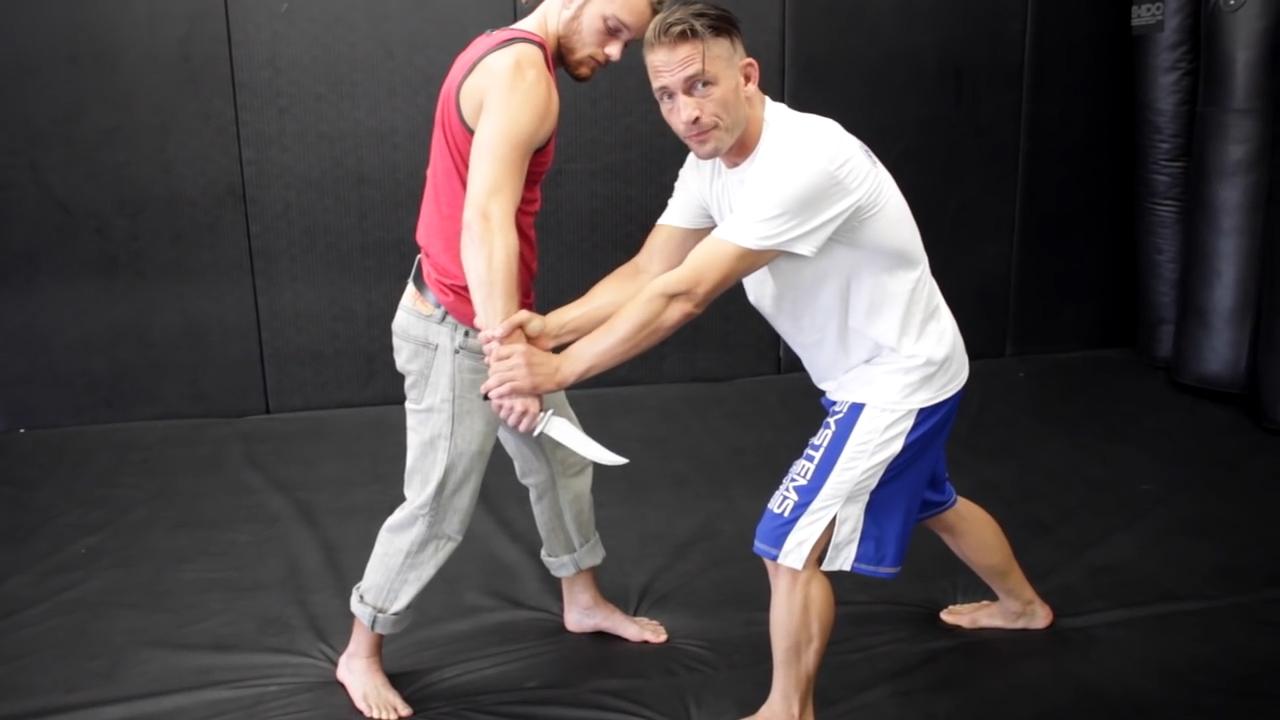

Supplement: Supplementary file 2 — Supplementary Information 2. [file 41598_2023_35190_MOESM2_ESM.zip › test/images/KravMagaKnifeDefenseTechniques670_jpg.rf.6bfdb3216451cf94d815bd3cc2dd2074.jpg]

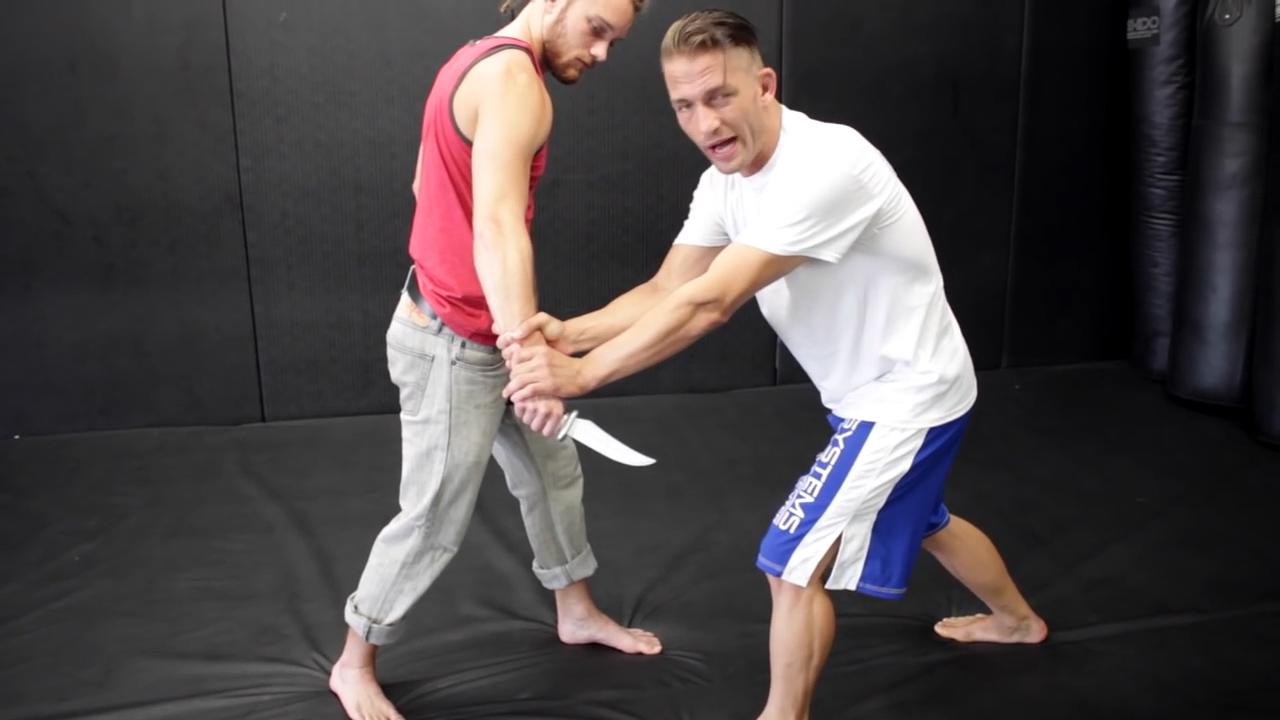

Supplement: Supplementary file 2 — Supplementary Information 2. [file 41598_2023_35190_MOESM2_ESM.zip › test/images/KravMagaKnifeDefenseTechniques674_jpg.rf.77eb31476e8df7e7678e83def1751ec8.jpg]

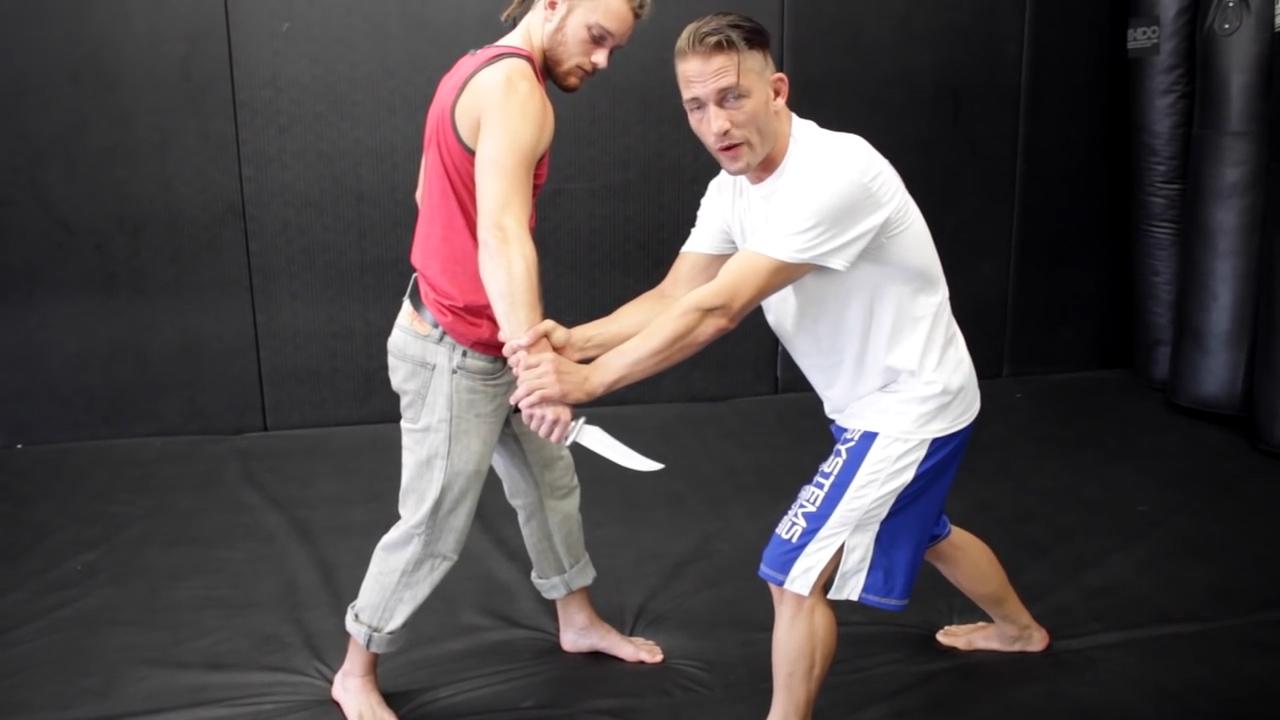

Supplement: Supplementary file 2 — Supplementary Information 2. [file 41598_2023_35190_MOESM2_ESM.zip › test/images/KravMagaKnifeDefenseTechniques676_jpg.rf.ad483aa4d7dc8d623a7ec8c98eb0b7ee.jpg]

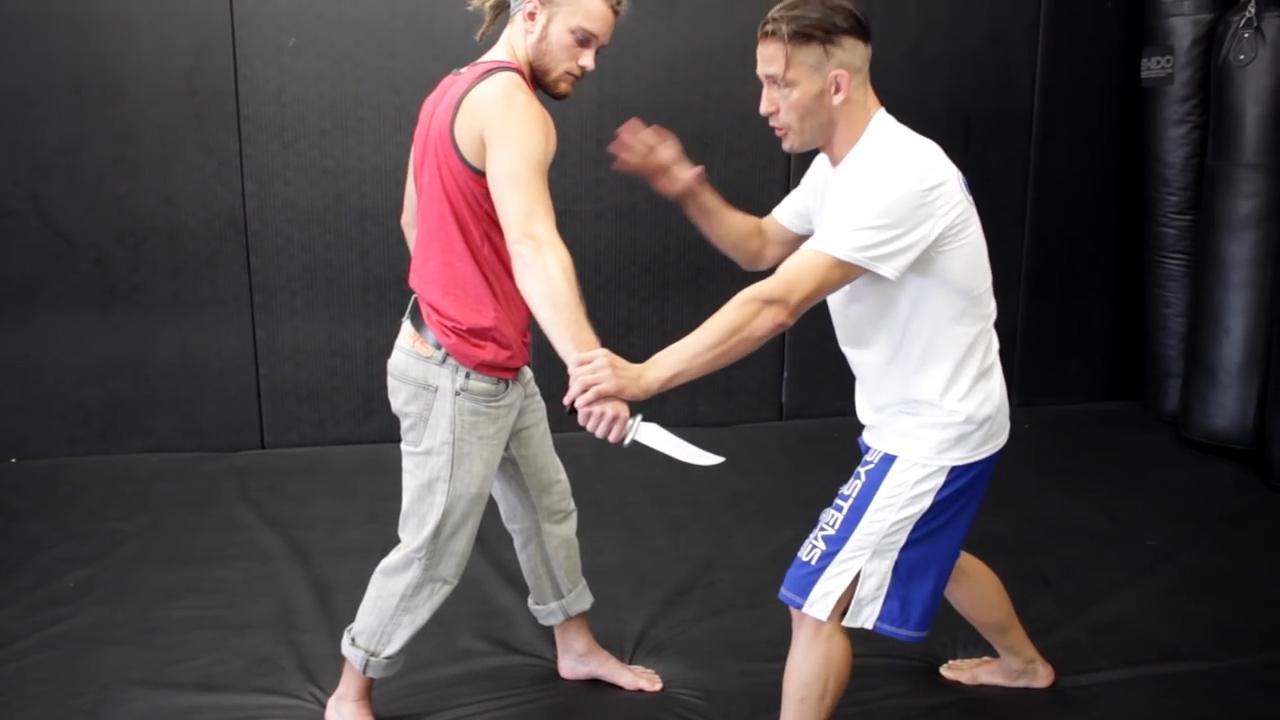

Supplement: Supplementary file 2 — Supplementary Information 2. [file 41598_2023_35190_MOESM2_ESM.zip › test/images/KravMagaKnifeDefenseTechniques695_jpg.rf.c65cab4522d484425e4edba77870c78b.jpg]

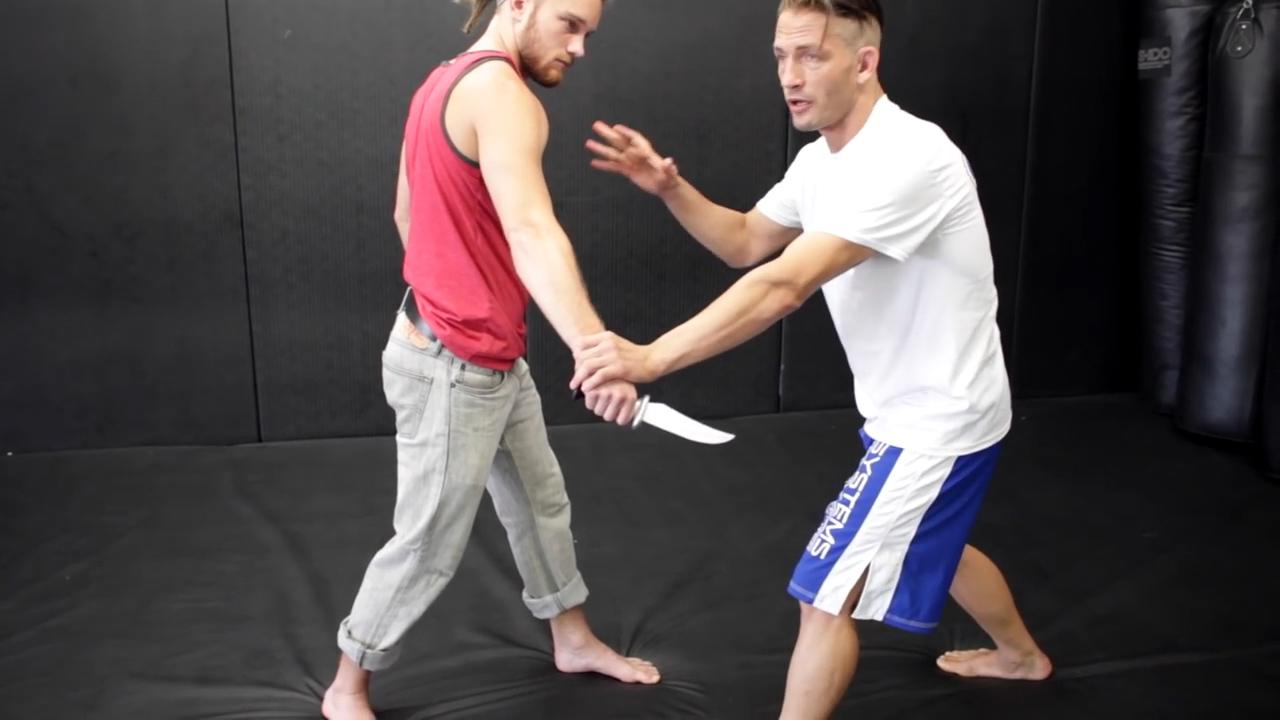

Supplement: Supplementary file 2 — Supplementary Information 2. [file 41598_2023_35190_MOESM2_ESM.zip › test/images/KravMagaKnifeDefenseTechniques697_jpg.rf.22a7db9bd6cc799d56188dcddc11a7cb.jpg]

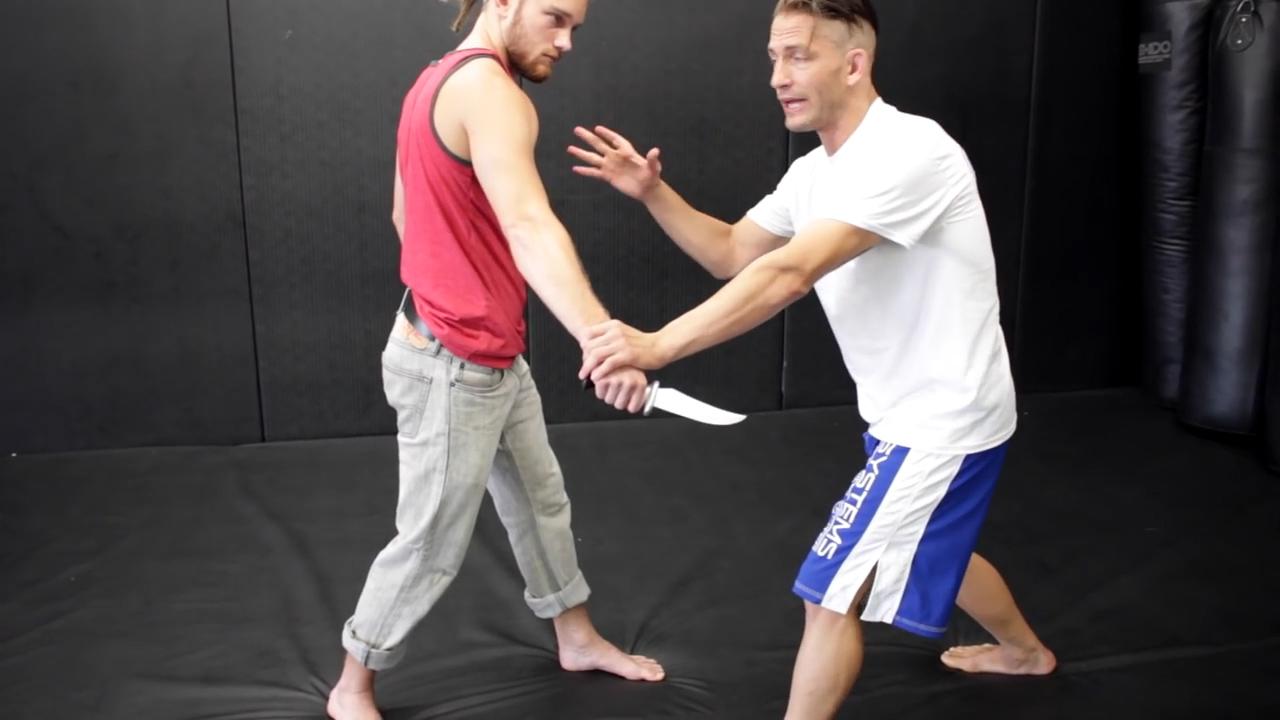

Supplement: Supplementary file 2 — Supplementary Information 2. [file 41598_2023_35190_MOESM2_ESM.zip › test/images/KravMagaKnifeDefenseTechniques699_jpg.rf.bd2caf1cb697f410e3f3c6d8376502e8.jpg]

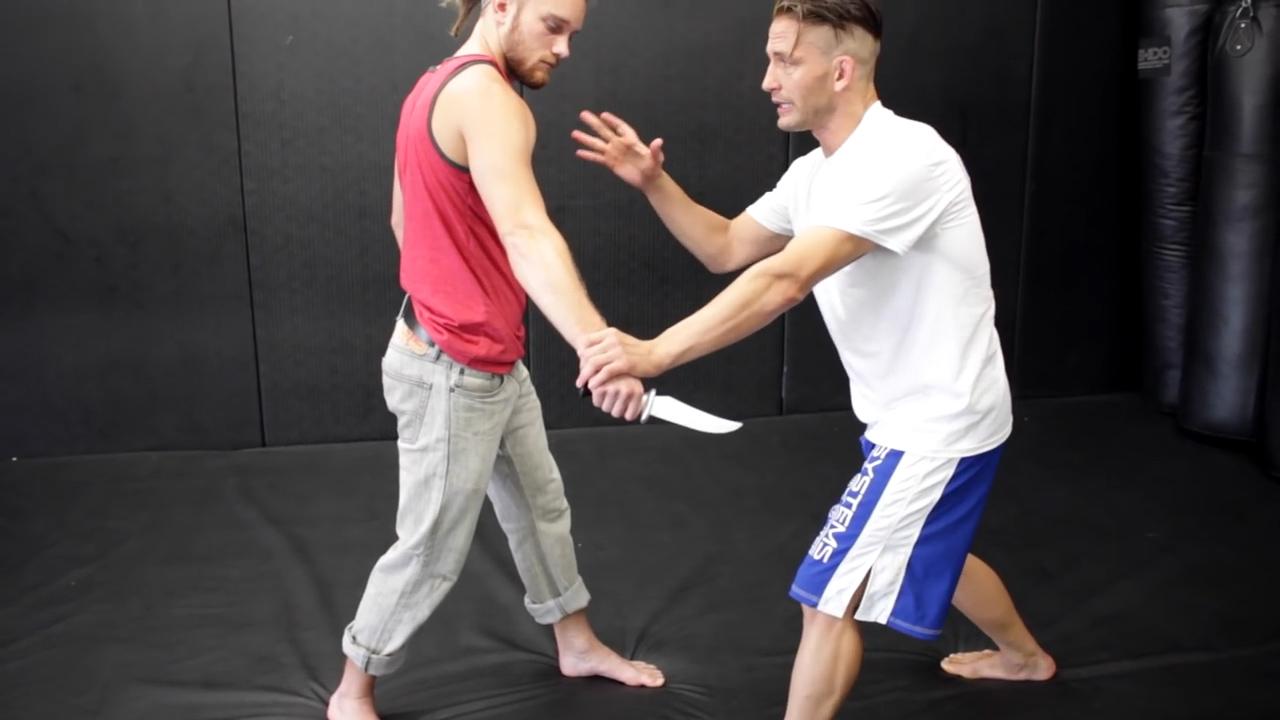

Supplement: Supplementary file 2 — Supplementary Information 2. [file 41598_2023_35190_MOESM2_ESM.zip › test/images/KravMagaKnifeDefenseTechniques700_jpg.rf.da8ba4f6d130698bf4515dfceb63b0fe.jpg]

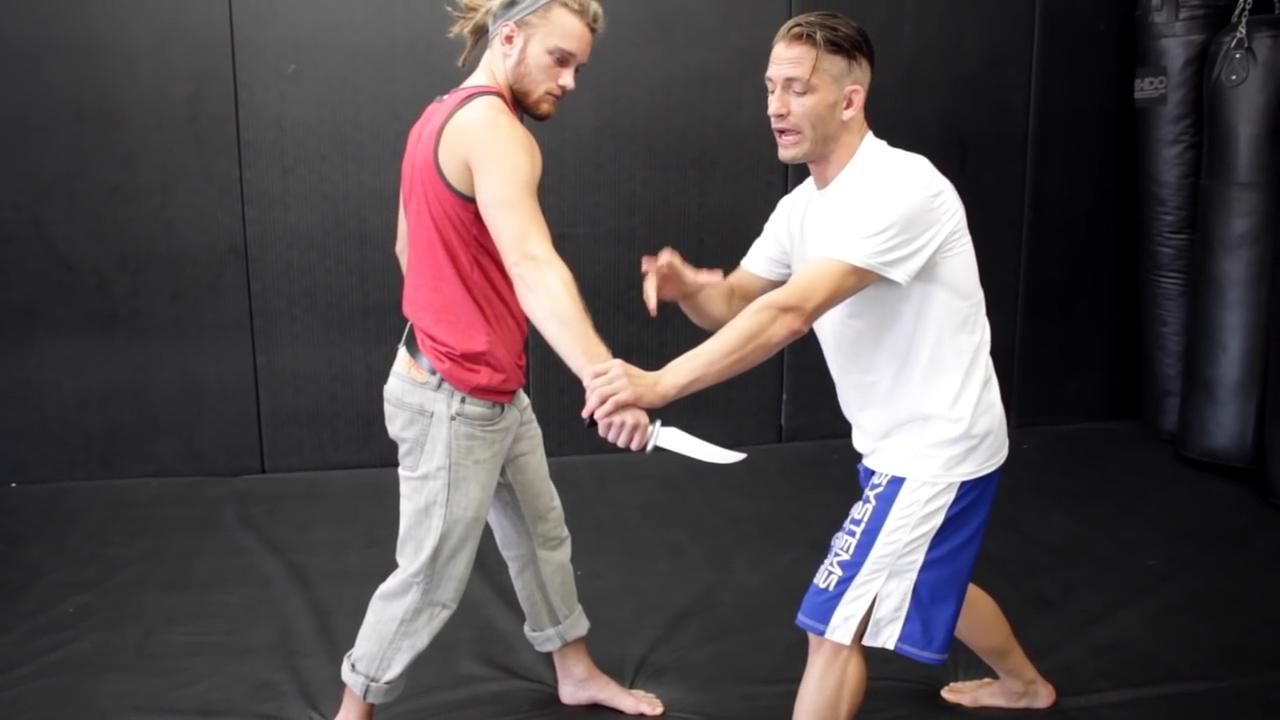

Supplement: Supplementary file 2 — Supplementary Information 2. [file 41598_2023_35190_MOESM2_ESM.zip › test/images/KravMagaKnifeDefenseTechniques703_jpg.rf.b13cda301d265c27f4e7f6b316b8fe36.jpg]

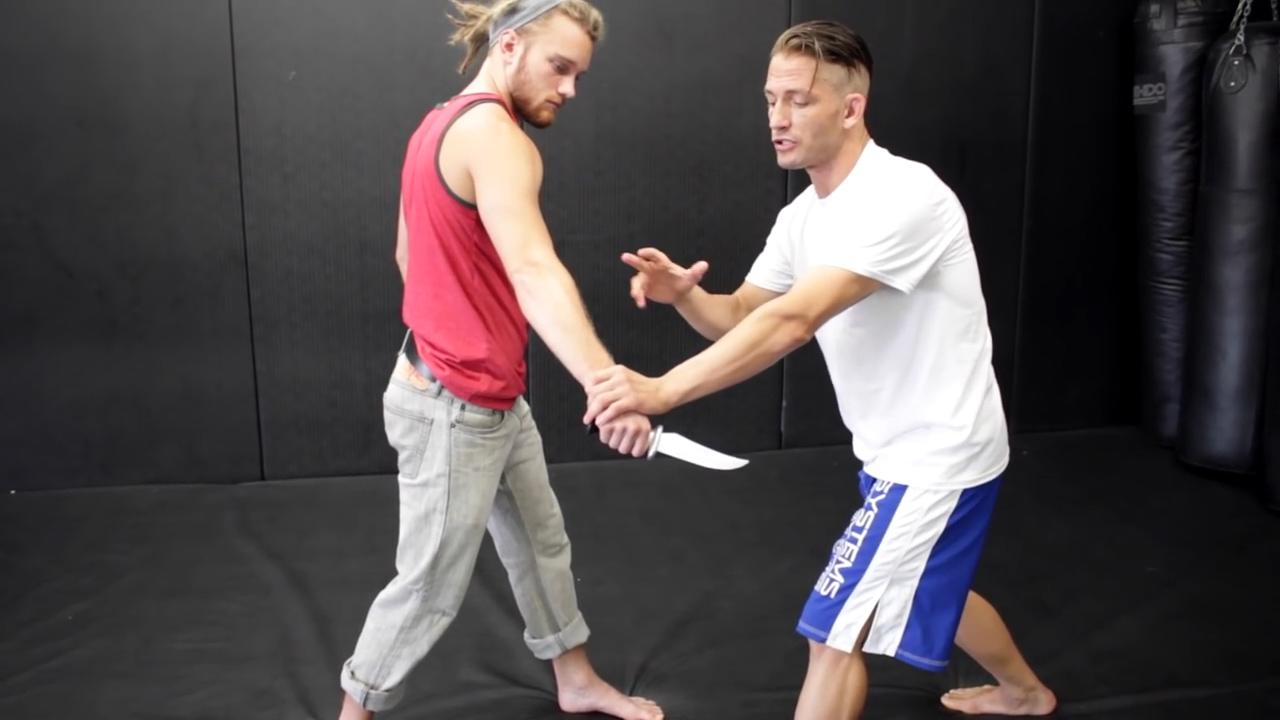

Supplement: Supplementary file 2 — Supplementary Information 2. [file 41598_2023_35190_MOESM2_ESM.zip › test/images/KravMagaKnifeDefenseTechniques704_jpg.rf.933b3de9dbe81d367357888187dd3315.jpg]

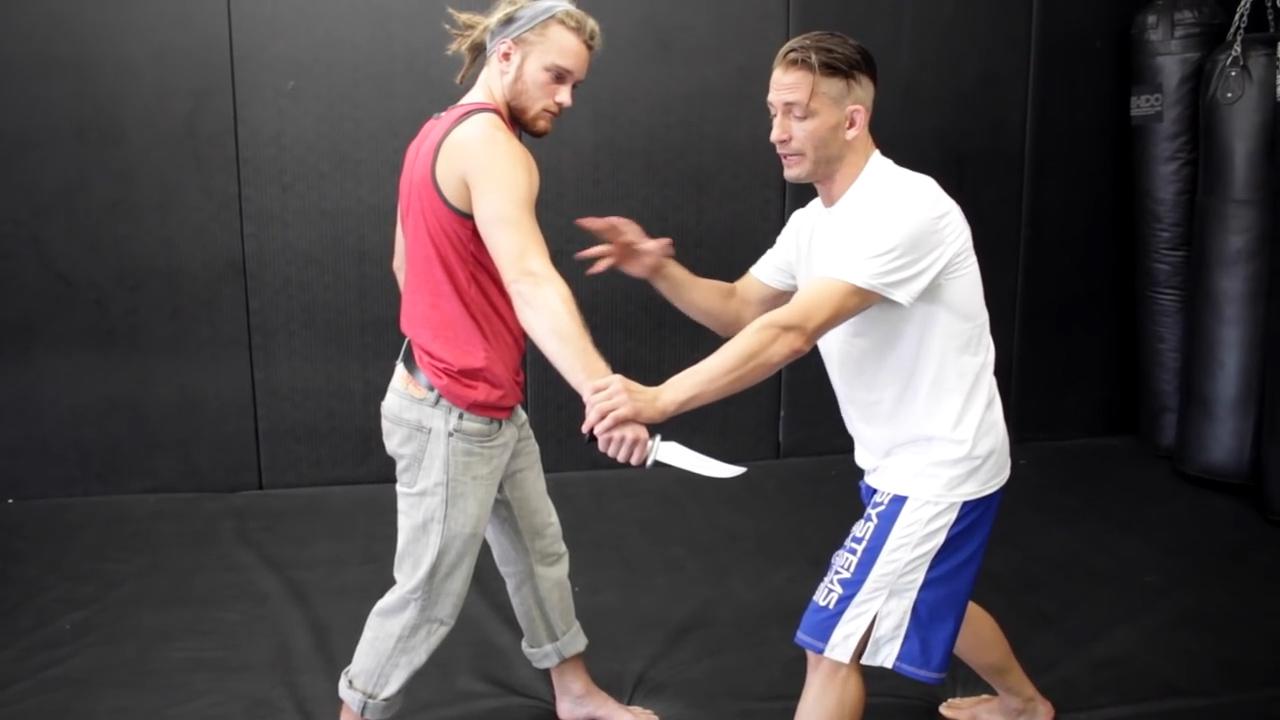

Supplement: Supplementary file 2 — Supplementary Information 2. [file 41598_2023_35190_MOESM2_ESM.zip › test/images/KravMagaKnifeDefenseTechniques706_jpg.rf.27c46d38417202aa1554edddf4e9a19c.jpg]

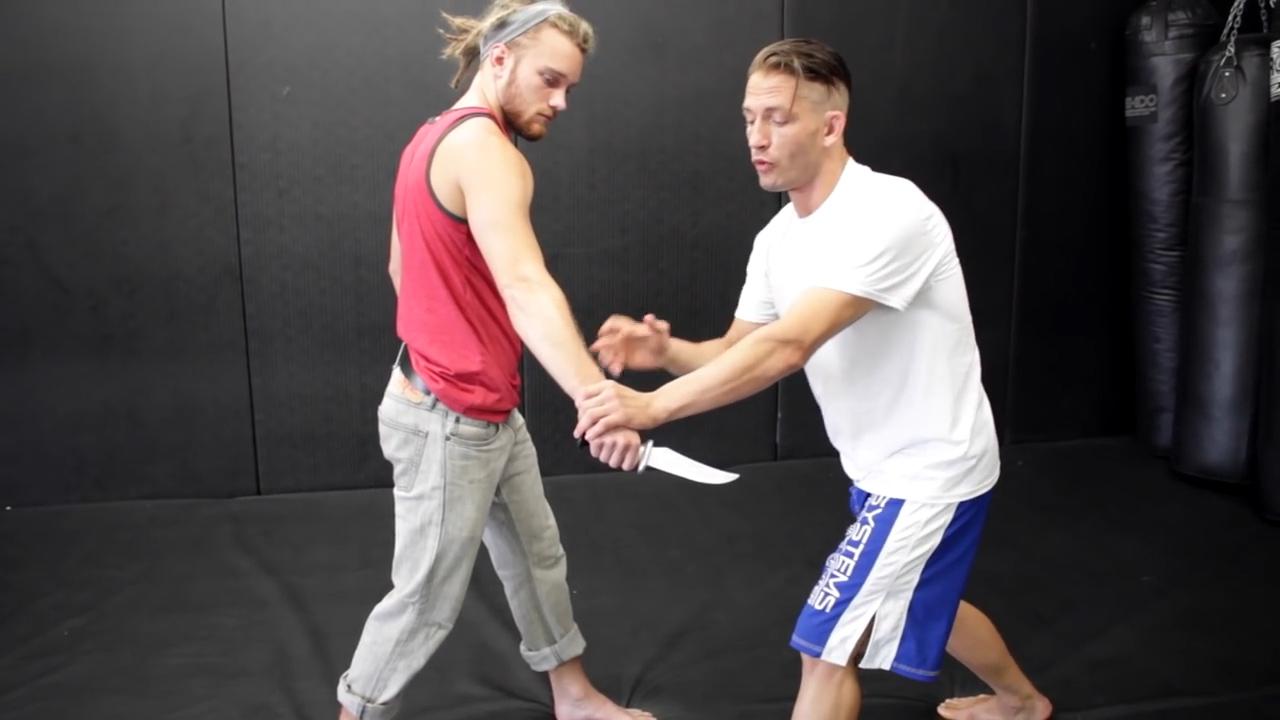

Supplement: Supplementary file 2 — Supplementary Information 2. [file 41598_2023_35190_MOESM2_ESM.zip › test/images/KravMagaKnifeDefenseTechniques709_jpg.rf.7ccf5bf2bc5f84403eb5f3e3ad0593d8.jpg]

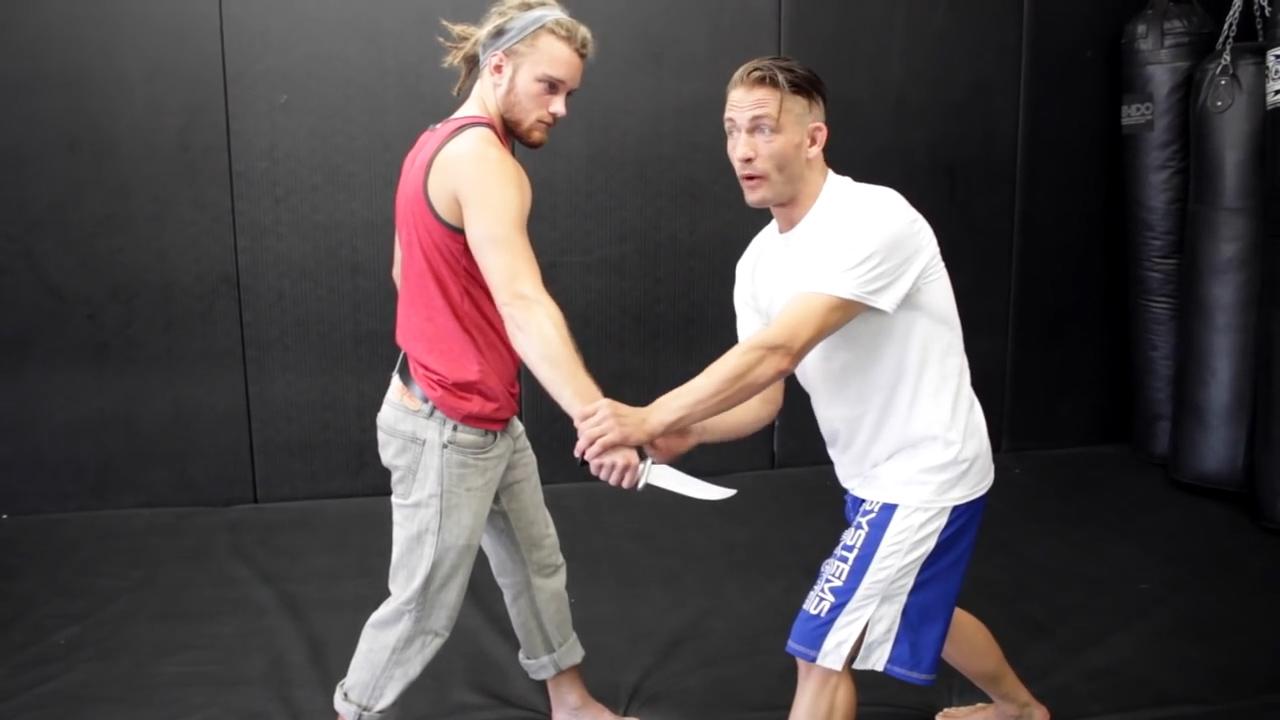

Supplement: Supplementary file 2 — Supplementary Information 2. [file 41598_2023_35190_MOESM2_ESM.zip › test/images/KravMagaKnifeDefenseTechniques713_jpg.rf.338238d8c93d900bc8665d49cc92398a.jpg]

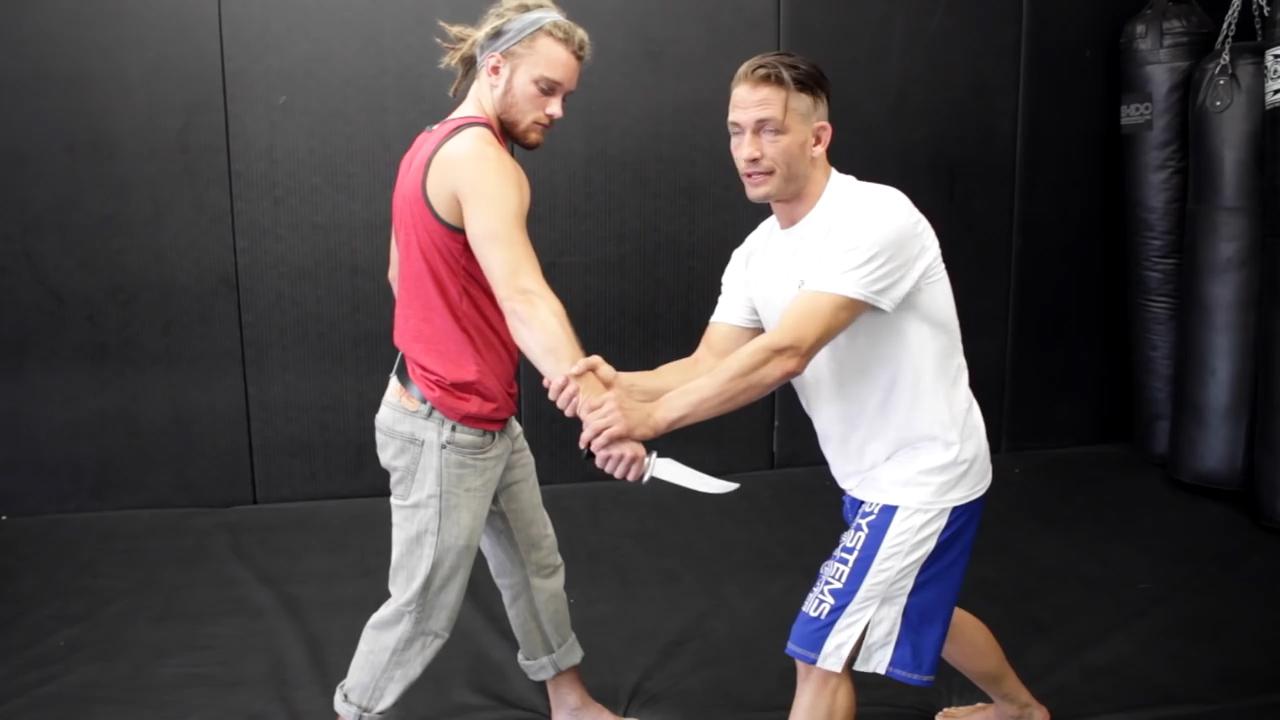

Supplement: Supplementary file 2 — Supplementary Information 2. [file 41598_2023_35190_MOESM2_ESM.zip › test/images/KravMagaKnifeDefenseTechniques715_jpg.rf.d1b5d12e39bf40b506f158c076e49ac0.jpg]

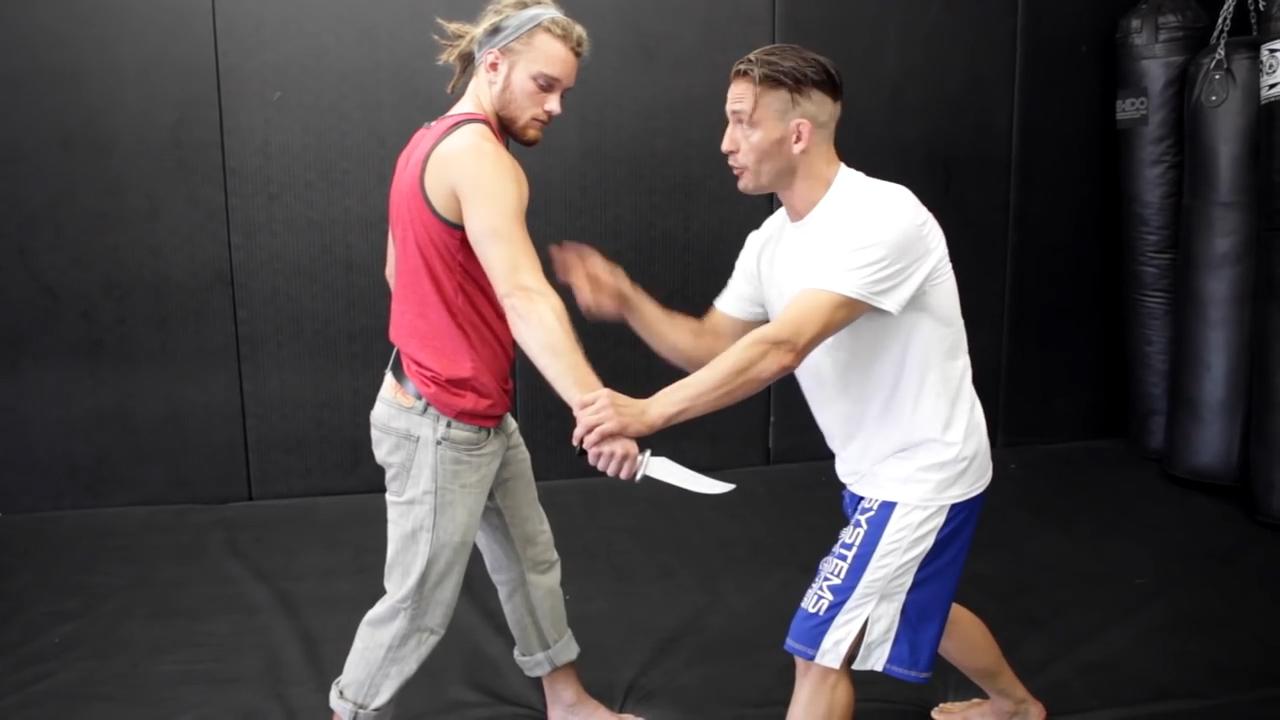

Supplement: Supplementary file 2 — Supplementary Information 2. [file 41598_2023_35190_MOESM2_ESM.zip › test/images/KravMagaKnifeDefenseTechniques719_jpg.rf.68ac23b83bf4bef049c131c28b4de52a.jpg]

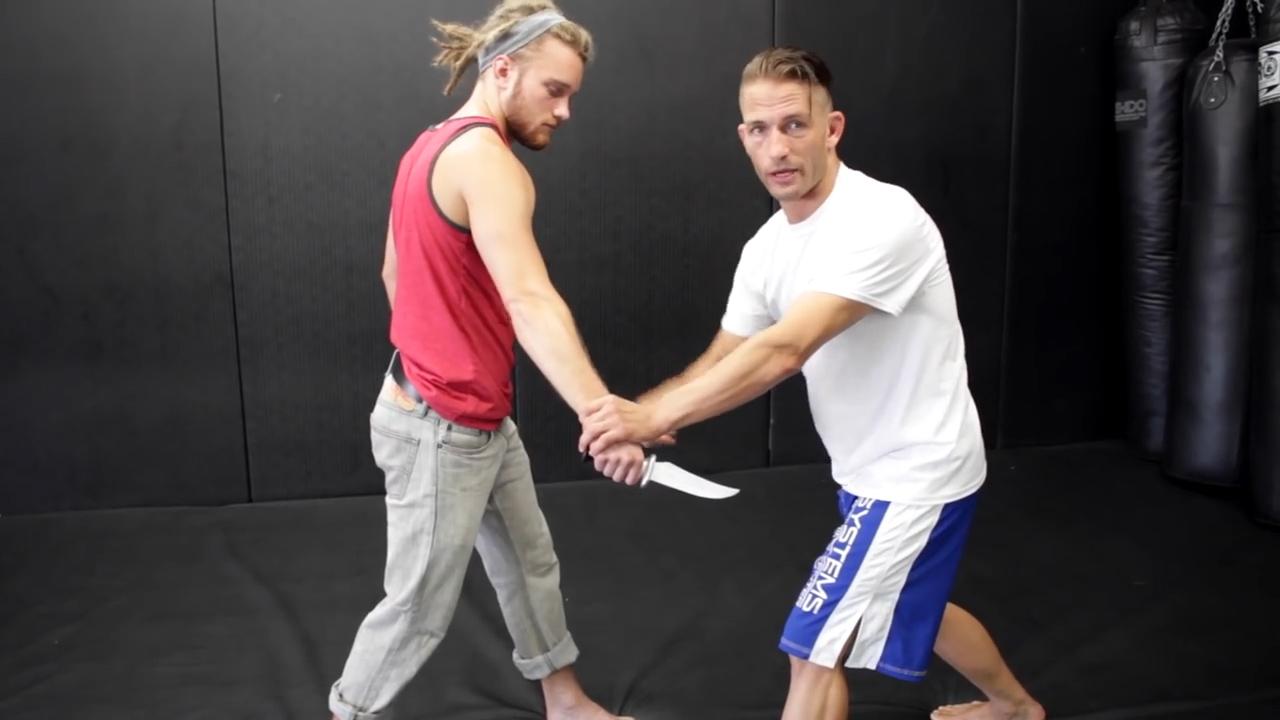

Supplement: Supplementary file 2 — Supplementary Information 2. [file 41598_2023_35190_MOESM2_ESM.zip › test/images/KravMagaKnifeDefenseTechniques721_jpg.rf.6afdd97c5457370de66817a346d734c3.jpg]

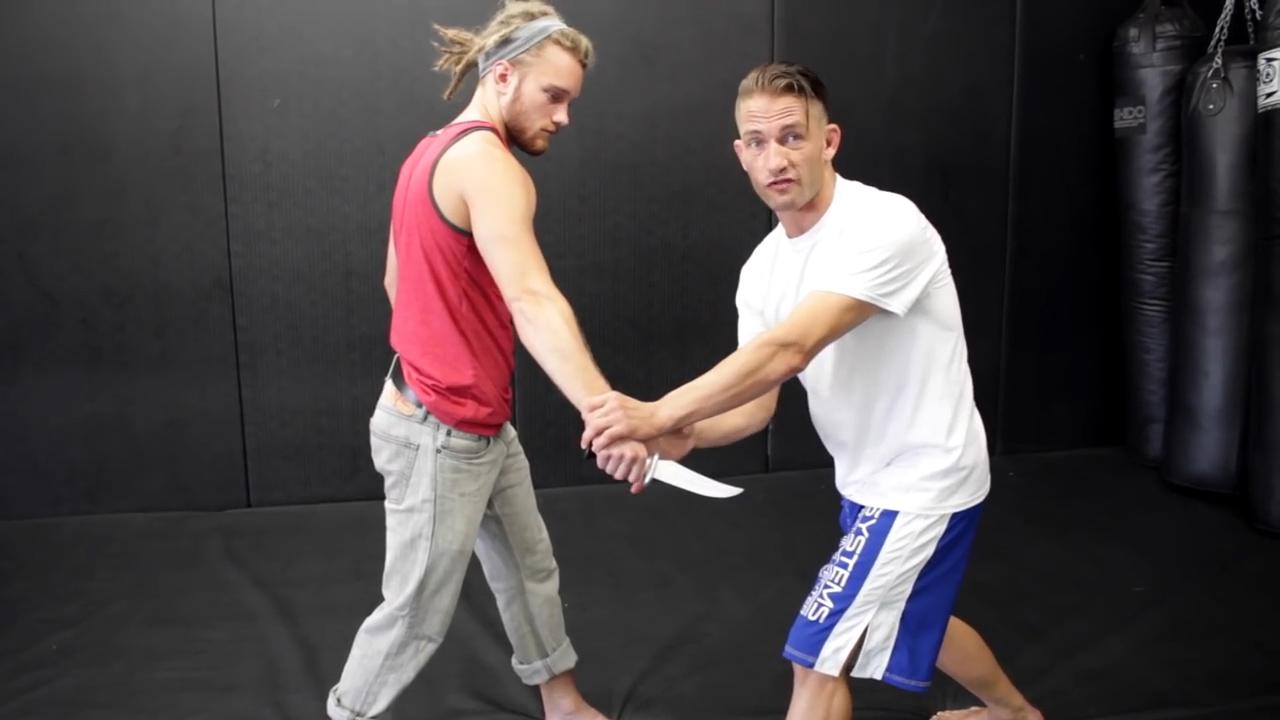

Supplement: Supplementary file 2 — Supplementary Information 2. [file 41598_2023_35190_MOESM2_ESM.zip › test/images/KravMagaKnifeDefenseTechniques724_jpg.rf.82d857d43596f5aa354ffbec012974f9.jpg]

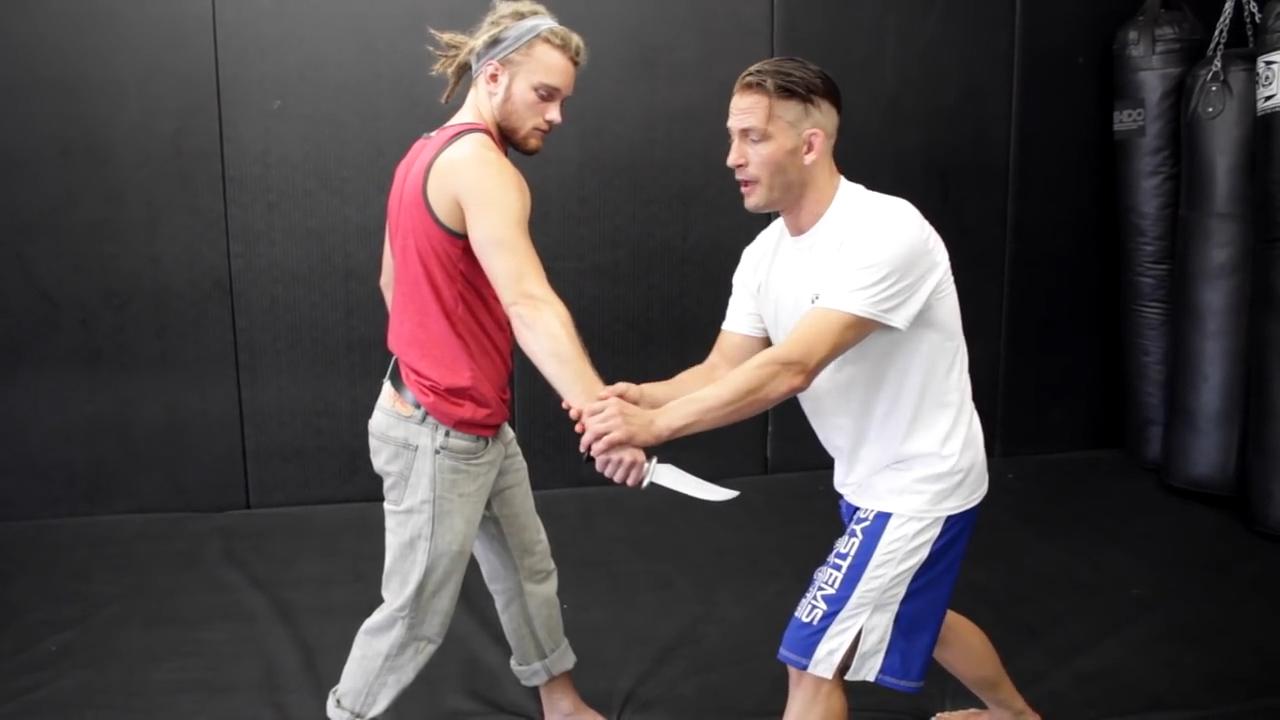

Supplement: Supplementary file 2 — Supplementary Information 2. [file 41598_2023_35190_MOESM2_ESM.zip › test/images/KravMagaKnifeDefenseTechniques730_jpg.rf.9c875d386ba1681d97c7468622c57094.jpg]

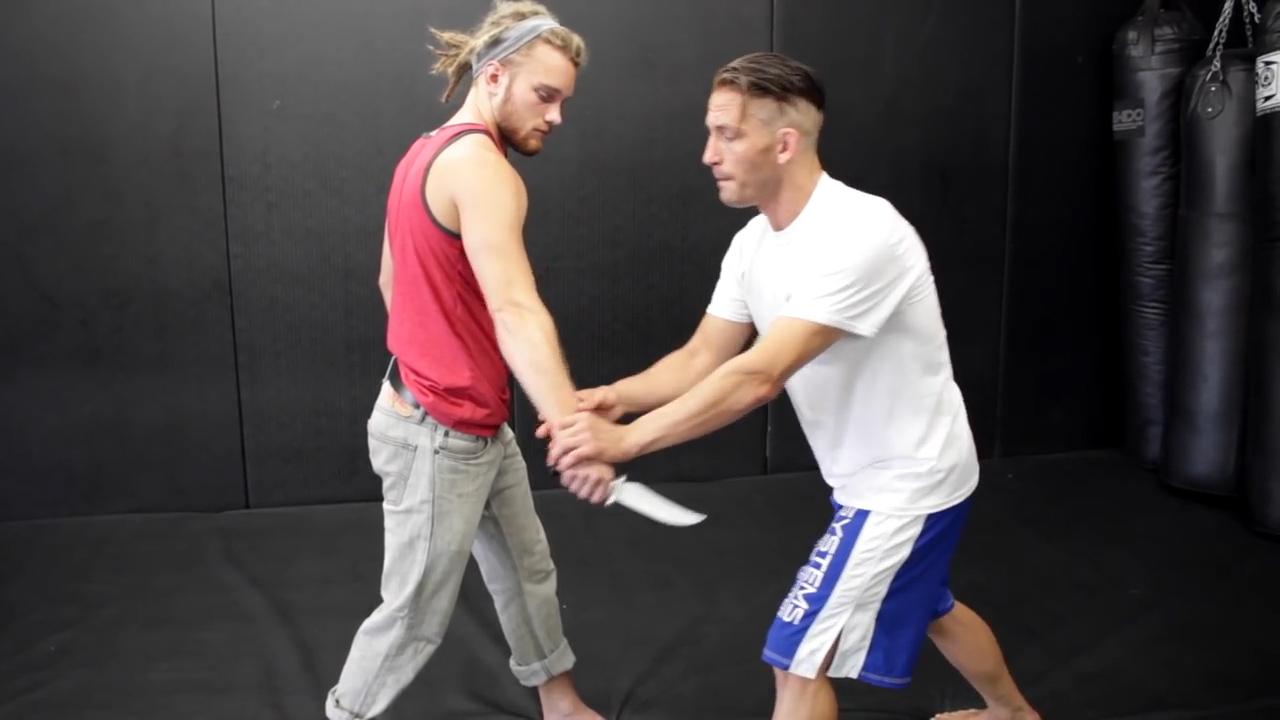

Supplement: Supplementary file 2 — Supplementary Information 2. [file 41598_2023_35190_MOESM2_ESM.zip › test/images/KravMagaKnifeDefenseTechniques731_jpg.rf.ca6c597ec6b1b5eb4cf8c62eed2aee7c.jpg]

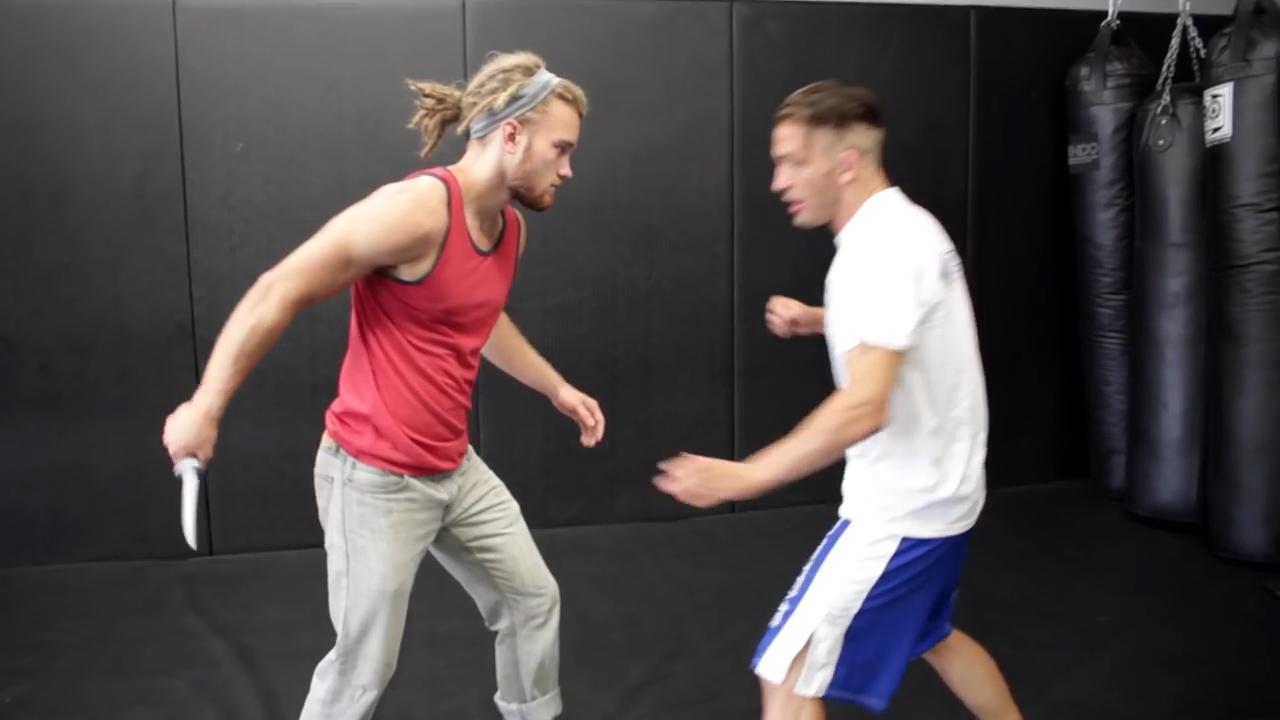

Supplement: Supplementary file 2 — Supplementary Information 2. [file 41598_2023_35190_MOESM2_ESM.zip › test/images/KravMagaKnifeDefenseTechniques743_jpg.rf.41e1b834574d6d955ad0b487beaa7467.jpg]

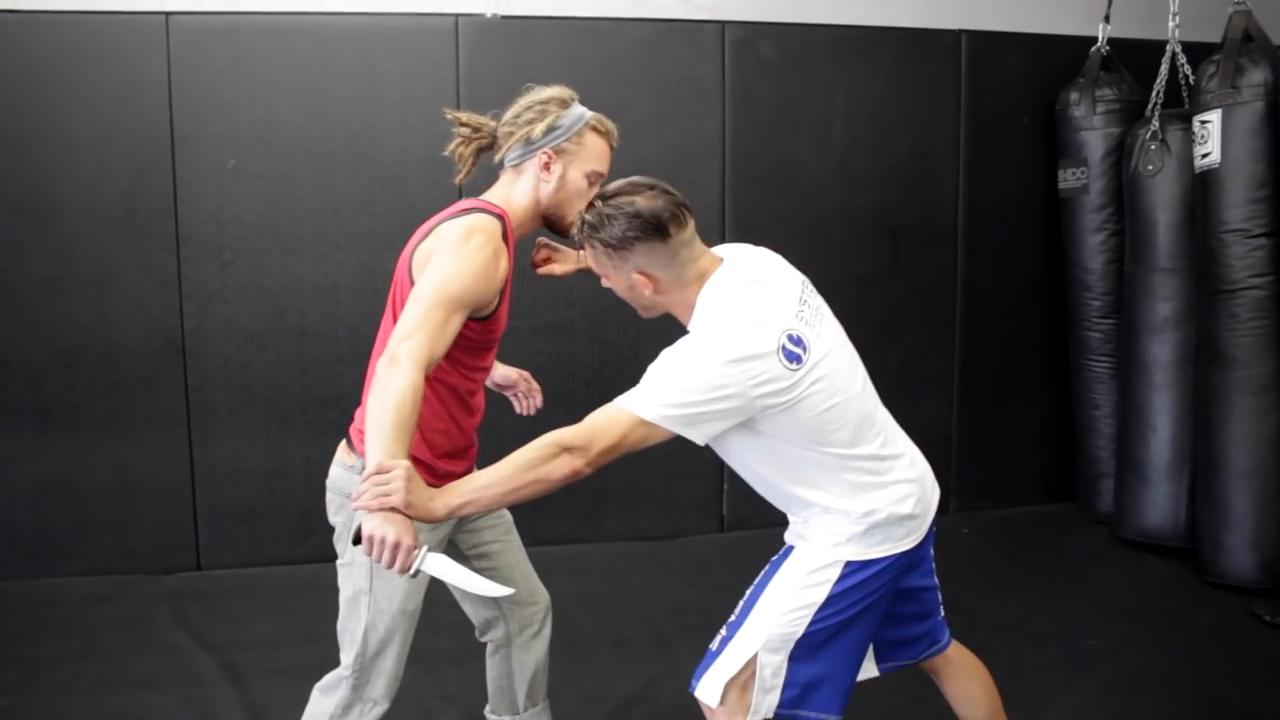

Supplement: Supplementary file 2 — Supplementary Information 2. [file 41598_2023_35190_MOESM2_ESM.zip › test/images/KravMagaKnifeDefenseTechniques746_jpg.rf.ba76c23b6de5eb3a2e93ba0fc410d303.jpg]

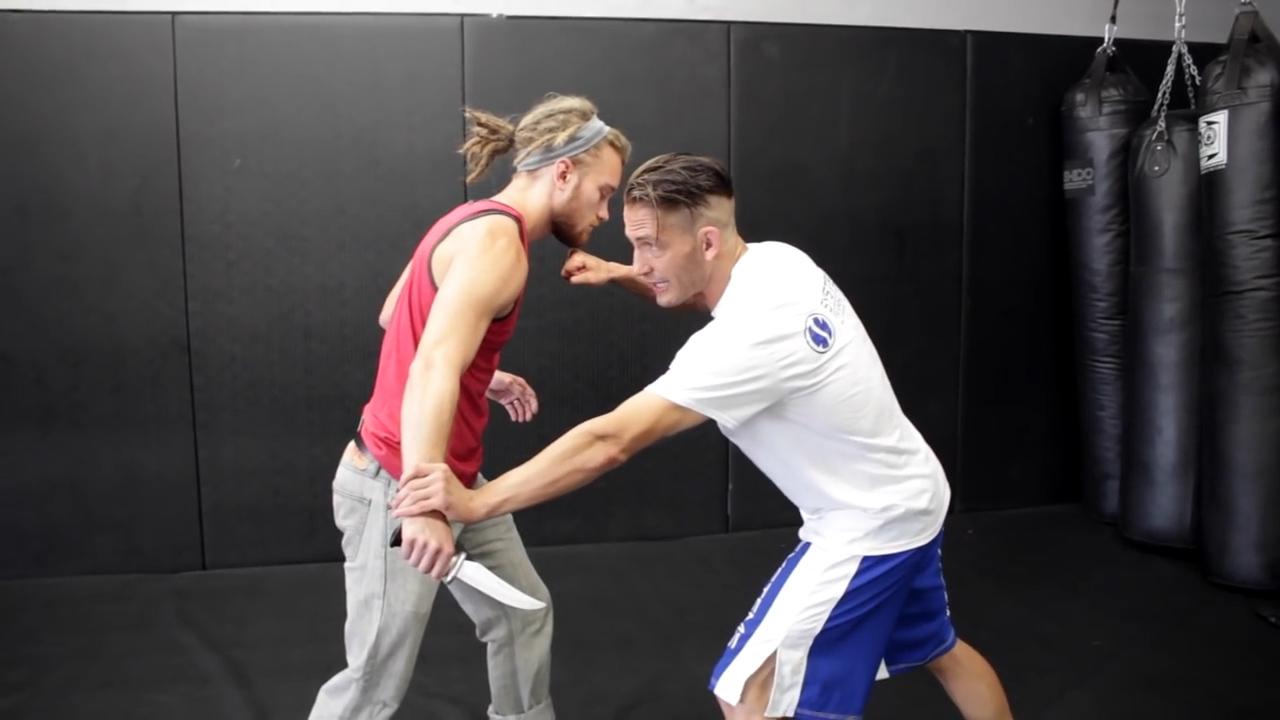

Supplement: Supplementary file 2 — Supplementary Information 2. [file 41598_2023_35190_MOESM2_ESM.zip › test/images/KravMagaKnifeDefenseTechniques750_jpg.rf.9dee0f7ca9df1367ae567206d02af499.jpg]

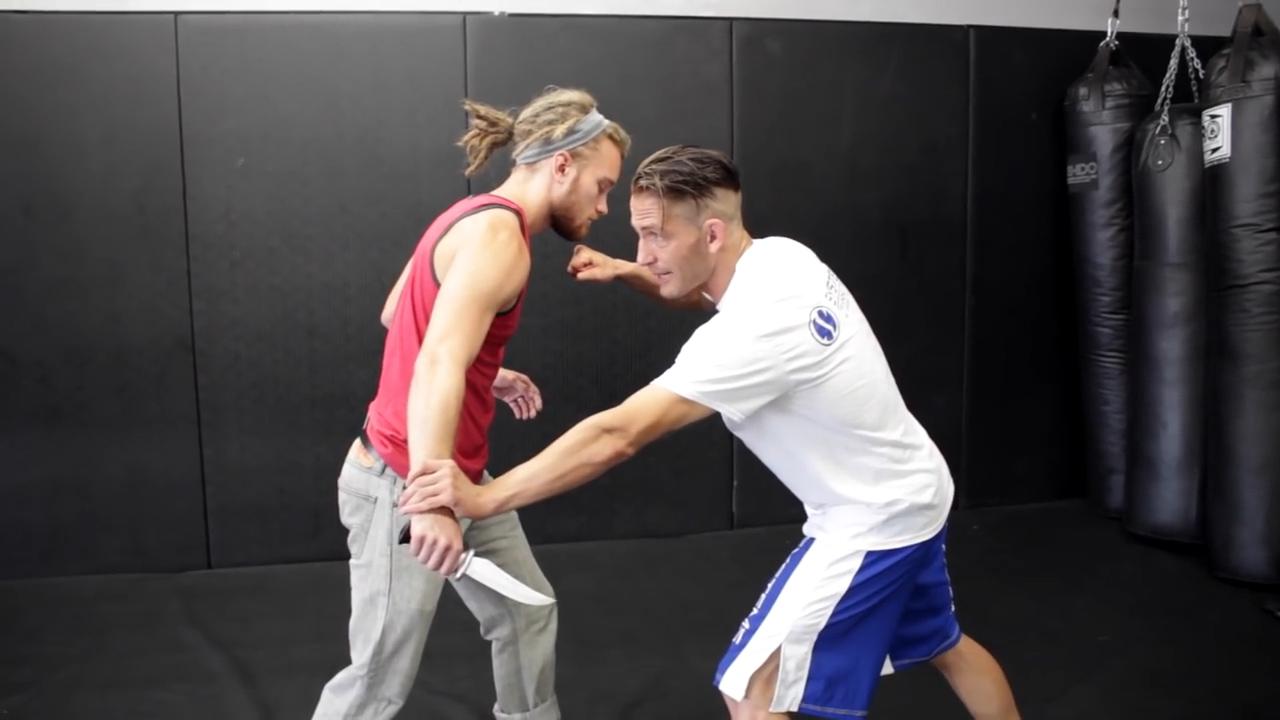

Supplement: Supplementary file 2 — Supplementary Information 2. [file 41598_2023_35190_MOESM2_ESM.zip › test/images/KravMagaKnifeDefenseTechniques752_jpg.rf.09f45997c2a19d60eb19ca534b1807af.jpg]

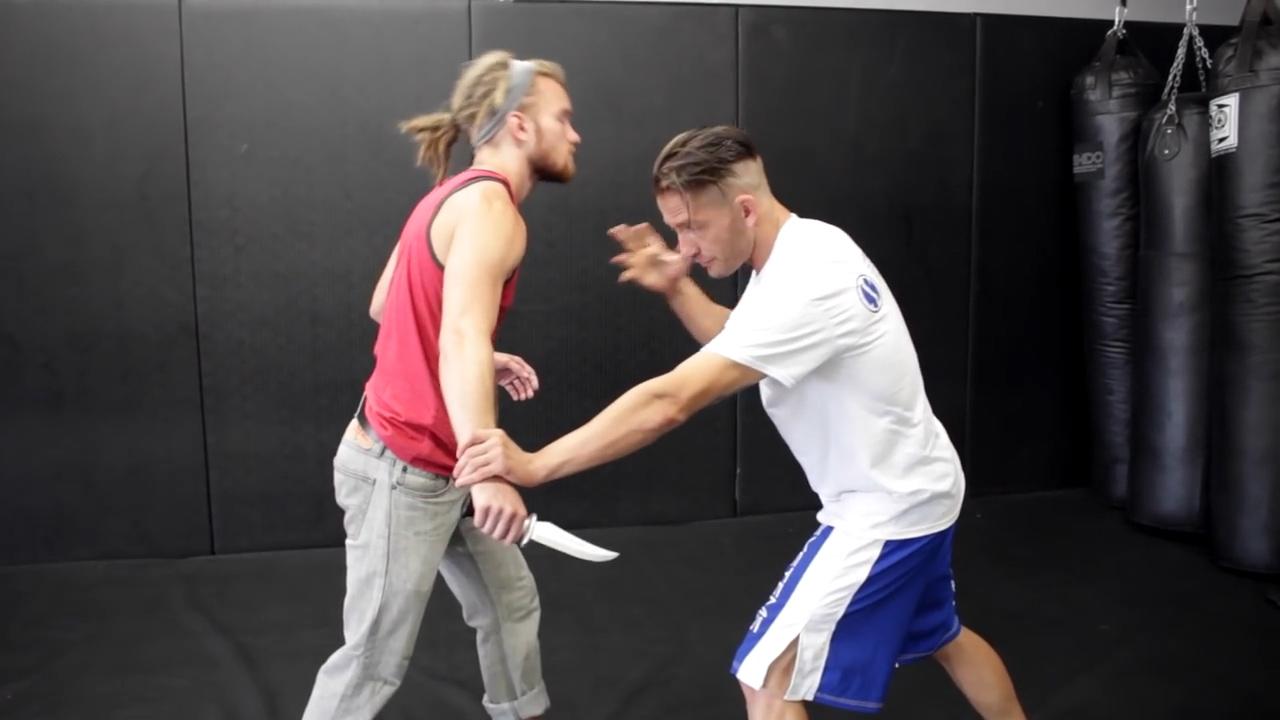

Supplement: Supplementary file 2 — Supplementary Information 2. [file 41598_2023_35190_MOESM2_ESM.zip › test/images/KravMagaKnifeDefenseTechniques757_jpg.rf.7fd5e17721f2f74f4f954490fd18b9e7.jpg]

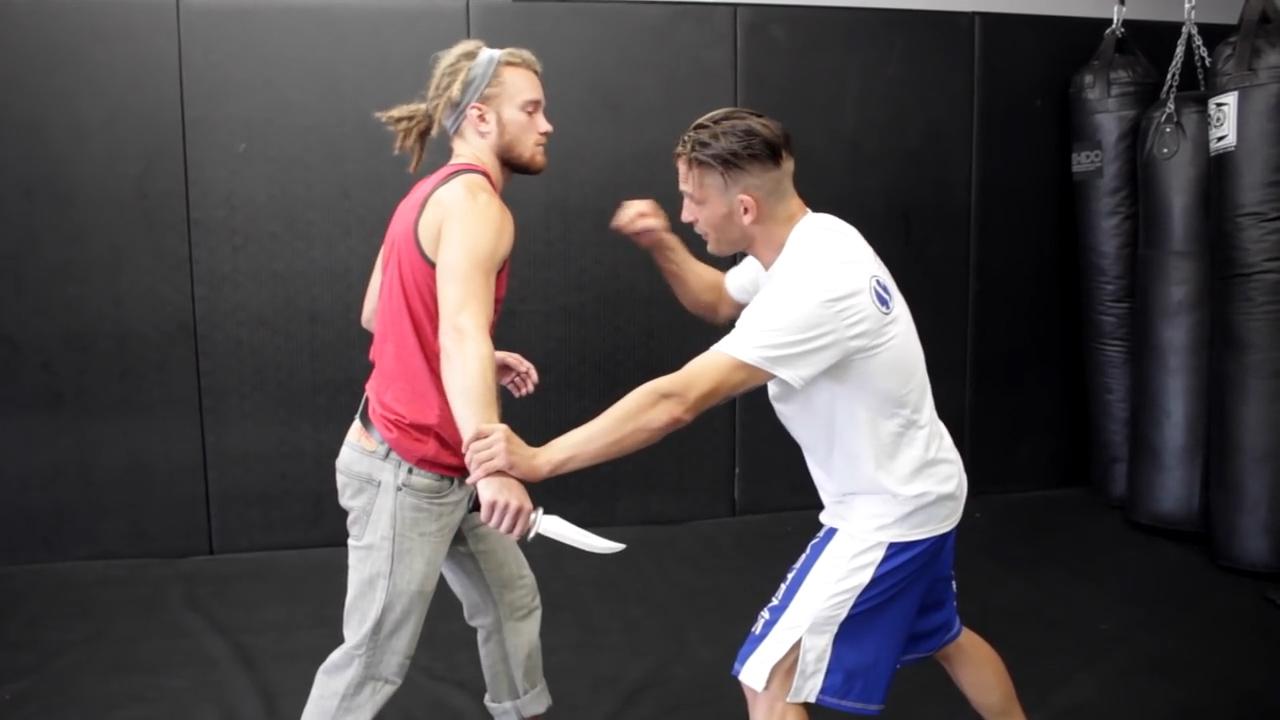

Supplement: Supplementary file 2 — Supplementary Information 2. [file 41598_2023_35190_MOESM2_ESM.zip › test/images/KravMagaKnifeDefenseTechniques759_jpg.rf.7c86a173ed1a893e9bdc6299fdf831ce.jpg]

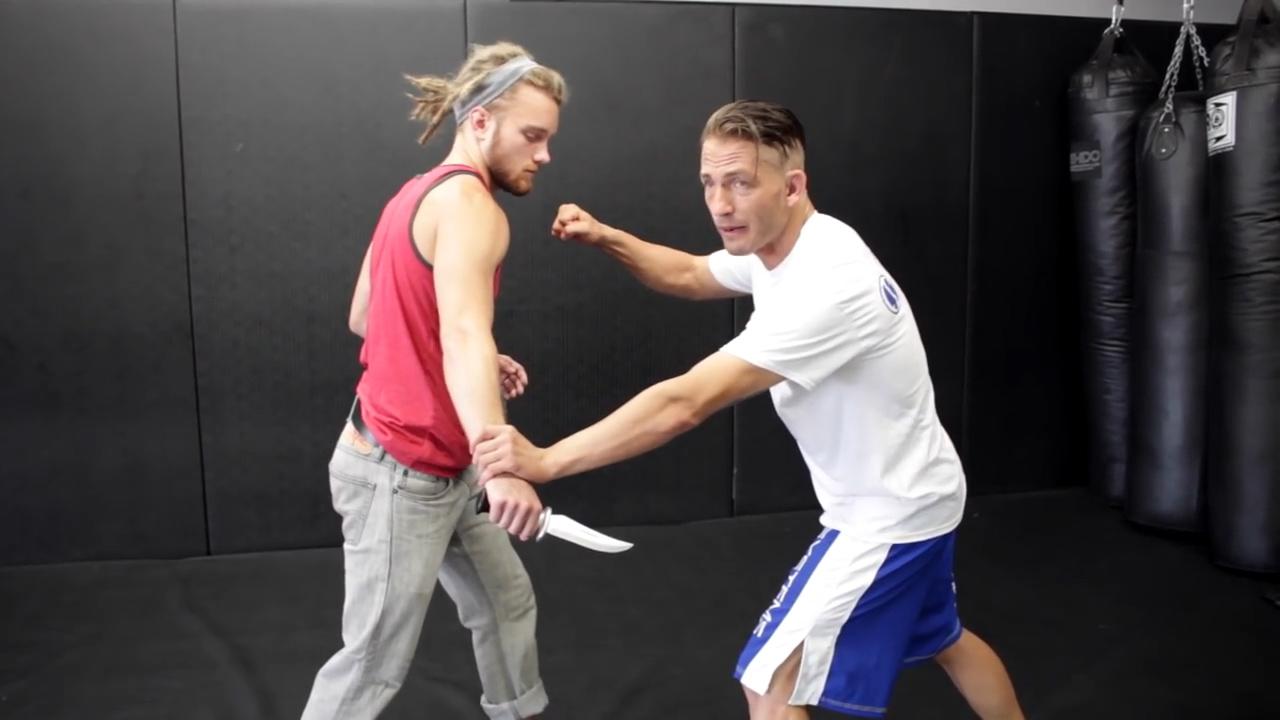

Supplement: Supplementary file 2 — Supplementary Information 2. [file 41598_2023_35190_MOESM2_ESM.zip › test/images/KravMagaKnifeDefenseTechniques762_jpg.rf.4a09e57e577b1b747ebafb0cc457911c.jpg]

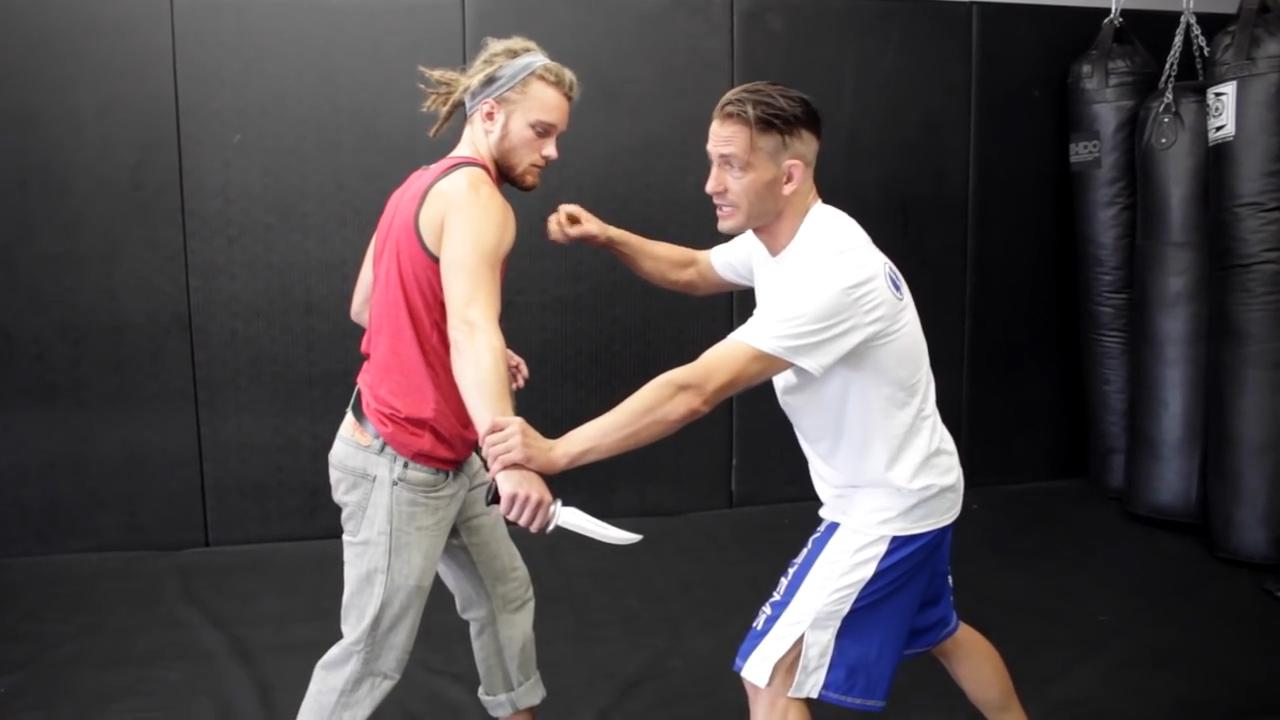

Supplement: Supplementary file 2 — Supplementary Information 2. [file 41598_2023_35190_MOESM2_ESM.zip › test/images/KravMagaKnifeDefenseTechniques765_jpg.rf.608d485ad44237a005c10bd0b5704e1a.jpg]

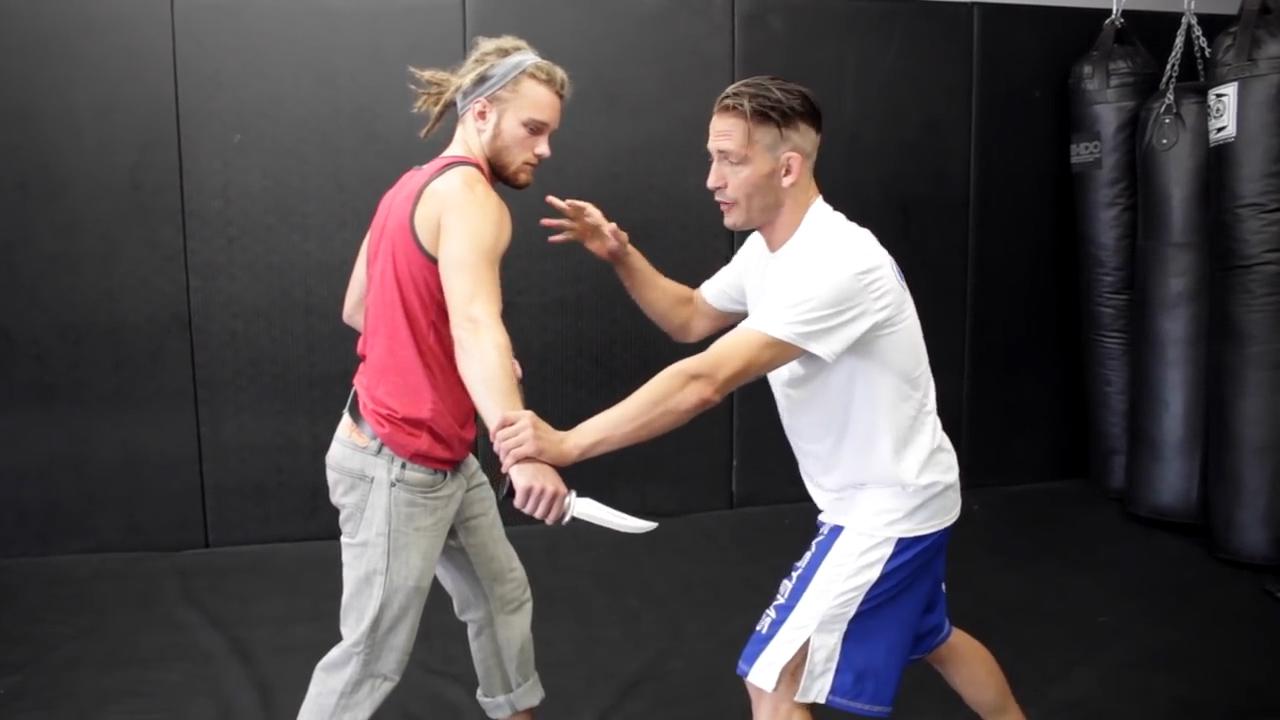

Supplement: Supplementary file 2 — Supplementary Information 2. [file 41598_2023_35190_MOESM2_ESM.zip › test/images/KravMagaKnifeDefenseTechniques768_jpg.rf.9324efb3c001a03ab4772a23f3304928.jpg]

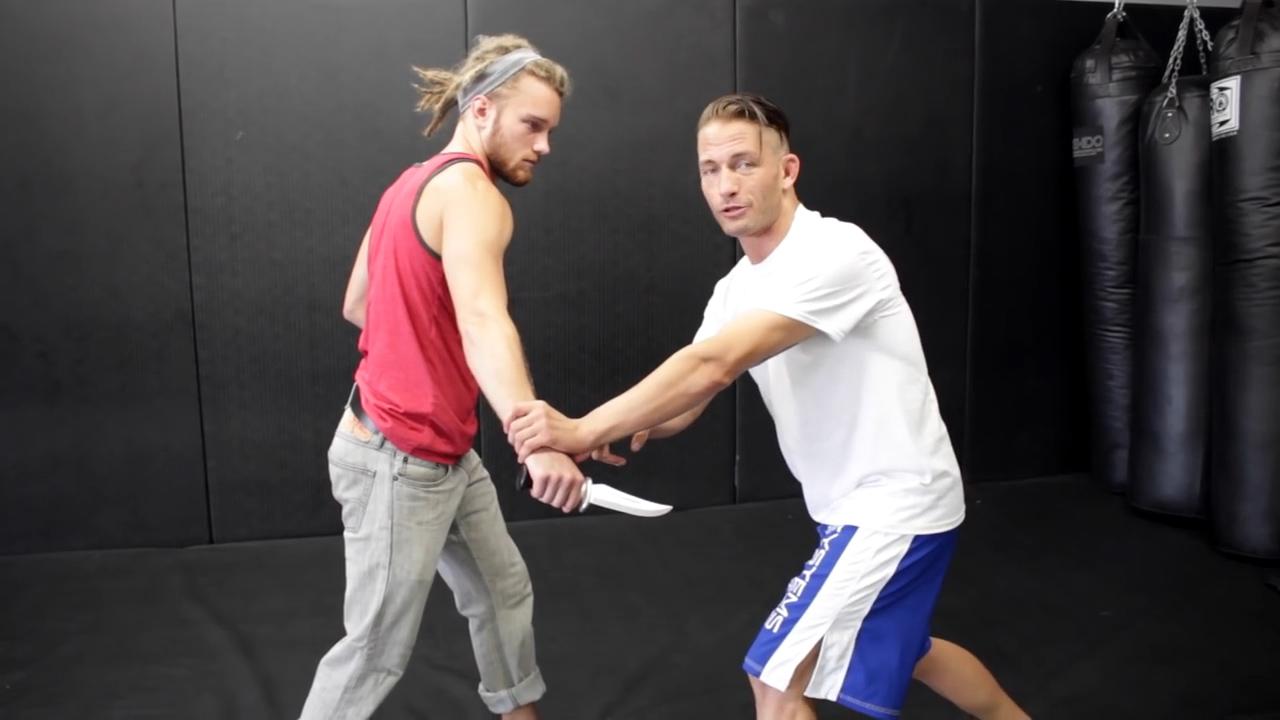

Supplement: Supplementary file 2 — Supplementary Information 2. [file 41598_2023_35190_MOESM2_ESM.zip › test/images/KravMagaKnifeDefenseTechniques773_jpg.rf.76d964af94994ad11be6284e5b1f2910.jpg]

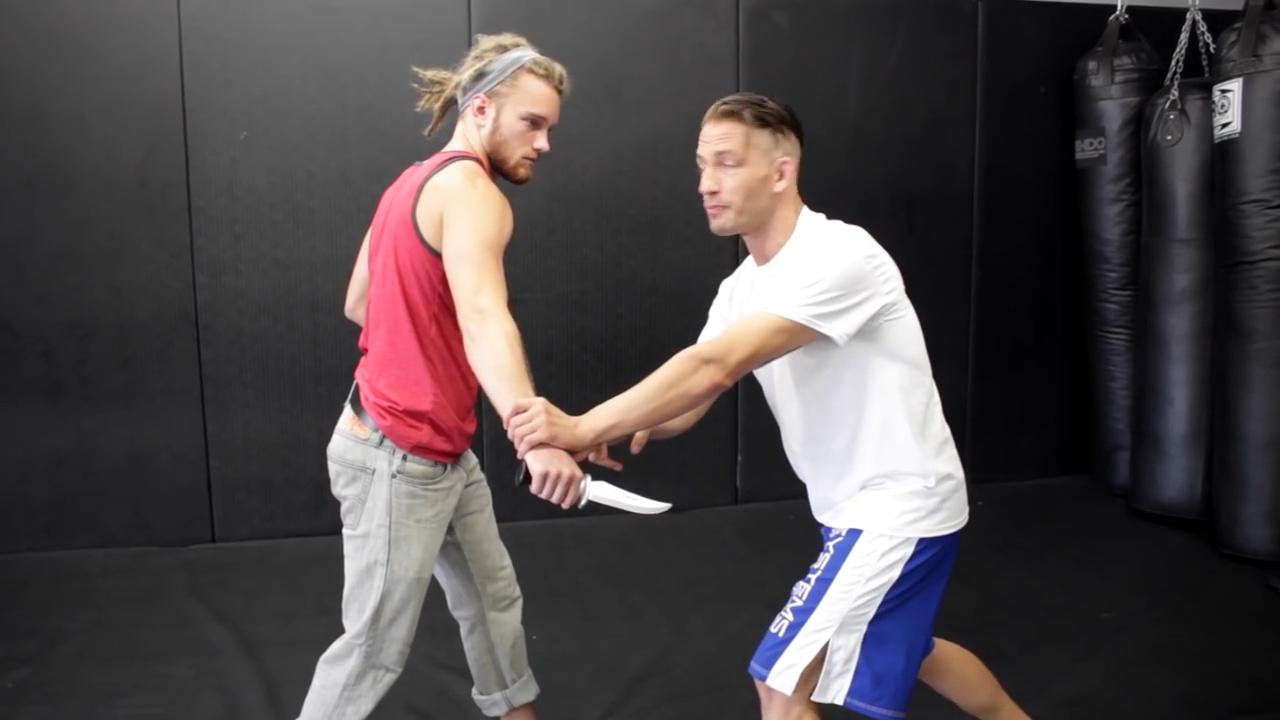

Supplement: Supplementary file 2 — Supplementary Information 2. [file 41598_2023_35190_MOESM2_ESM.zip › test/images/KravMagaKnifeDefenseTechniques775_jpg.rf.4dc7ff1c03506bf78b57774395bcce06.jpg]

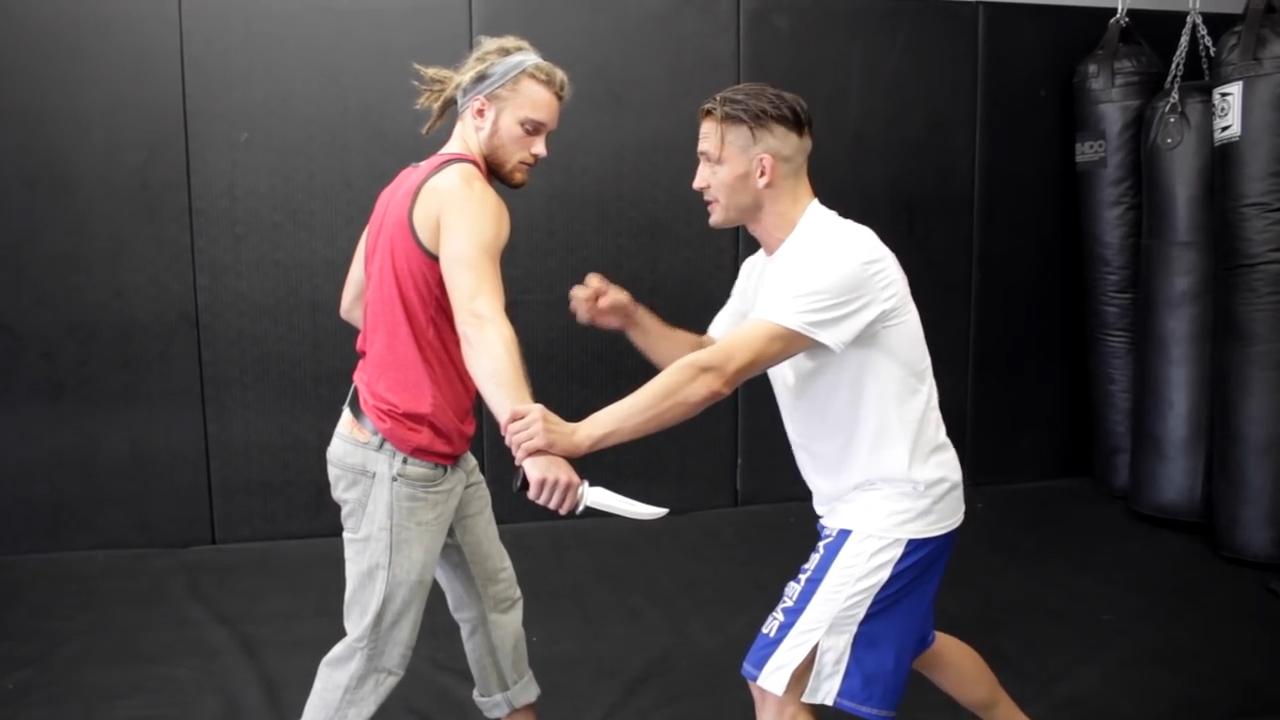

Supplement: Supplementary file 2 — Supplementary Information 2. [file 41598_2023_35190_MOESM2_ESM.zip › test/images/KravMagaKnifeDefenseTechniques777_jpg.rf.47de1fbc7fc3d10d37c94ff189f06845.jpg]

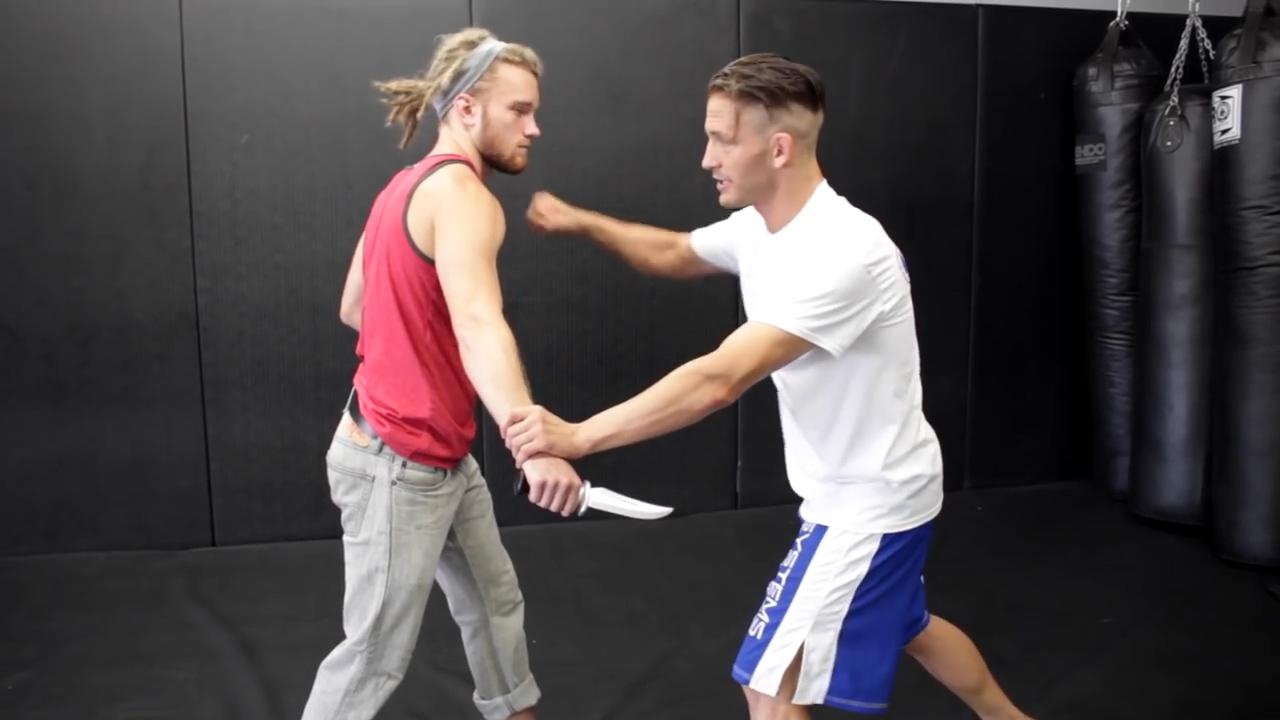

Supplement: Supplementary file 2 — Supplementary Information 2. [file 41598_2023_35190_MOESM2_ESM.zip › test/images/KravMagaKnifeDefenseTechniques780_jpg.rf.57726d290eeb68ac29f51d0c7911ae59.jpg]

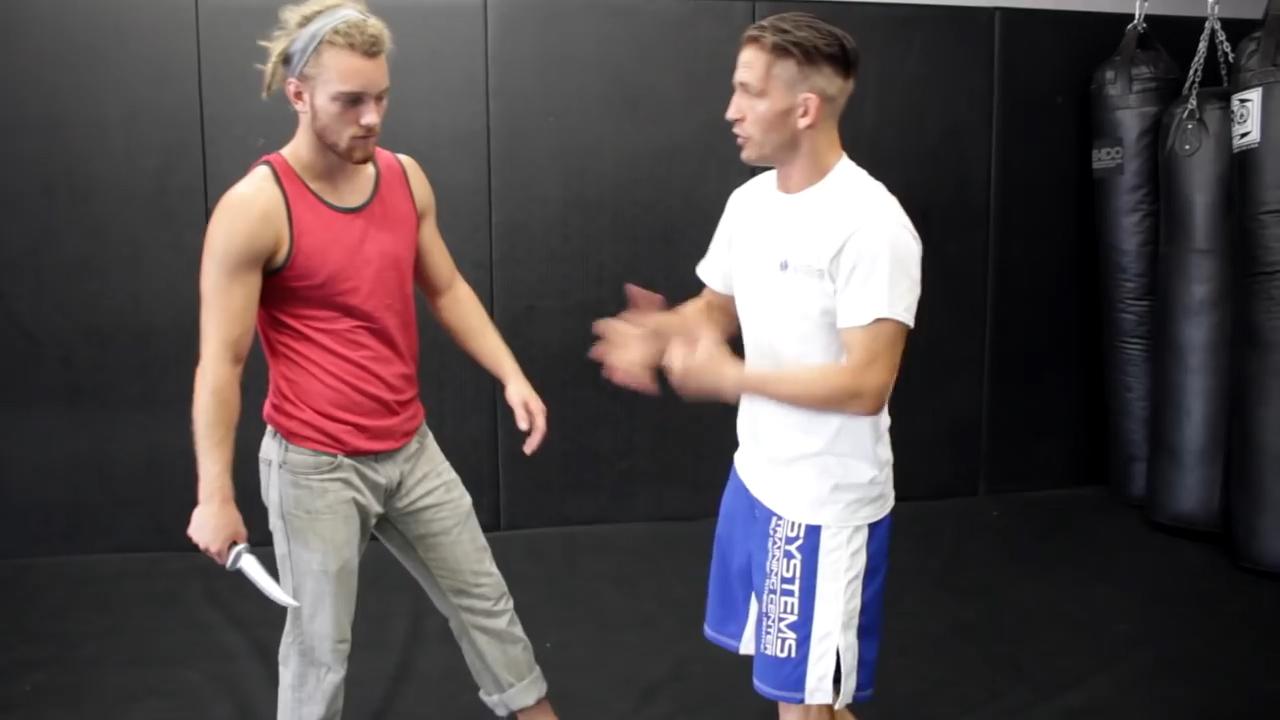

Supplement: Supplementary file 2 — Supplementary Information 2. [file 41598_2023_35190_MOESM2_ESM.zip › test/images/KravMagaKnifeDefenseTechniques795_jpg.rf.1d4771ffe978355487817a92d9b85eb5.jpg]

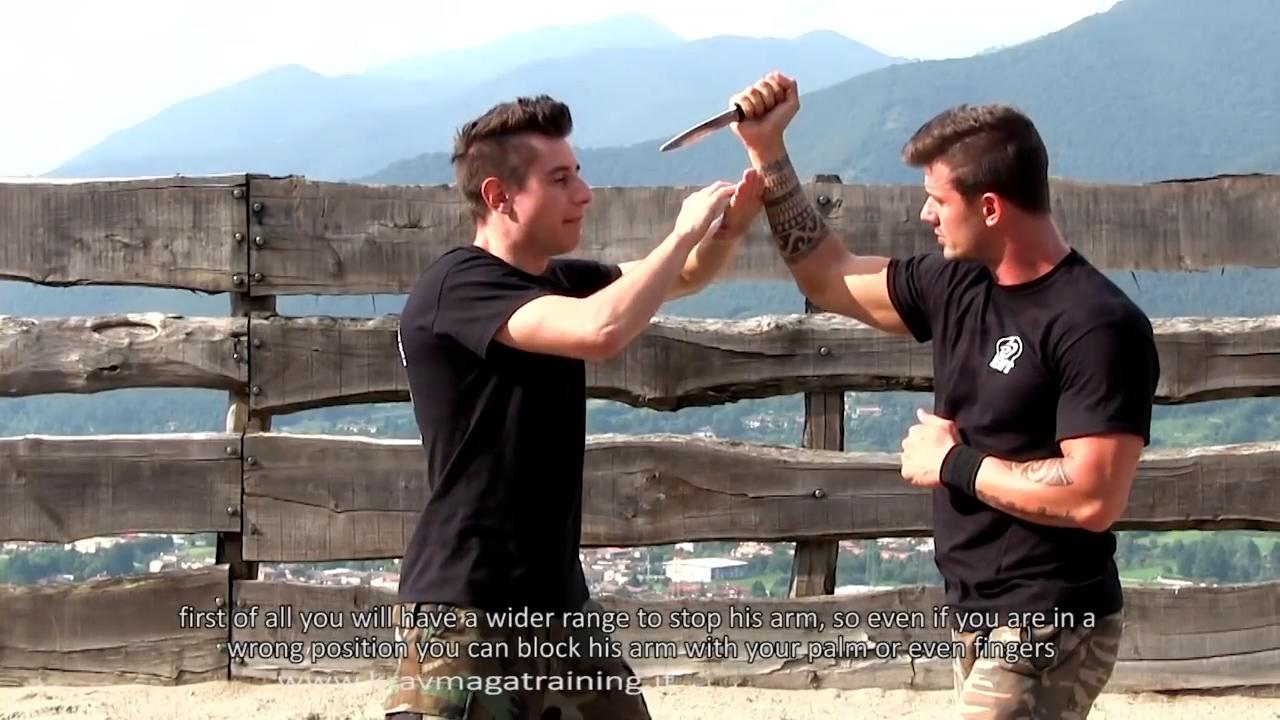

Supplement: Supplementary file 2 — Supplementary Information 2. [file 41598_2023_35190_MOESM2_ESM.zip › test/images/KravMagaTraining000_jpg.rf.4a5cdb073b467948fbc7264e0fa1551f.jpg]

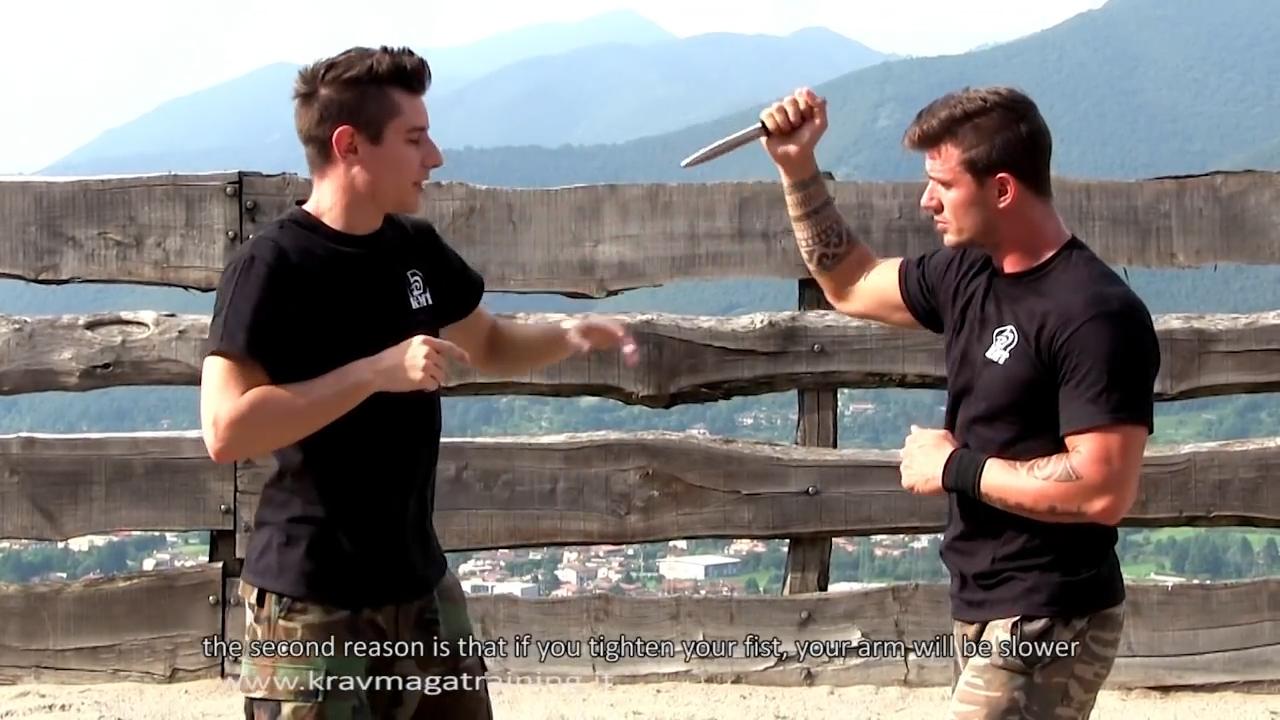

Supplement: Supplementary file 2 — Supplementary Information 2. [file 41598_2023_35190_MOESM2_ESM.zip › test/images/KravMagaTraining019_jpg.rf.fa5e8c86d94441b70a2cb73a03365817.jpg]

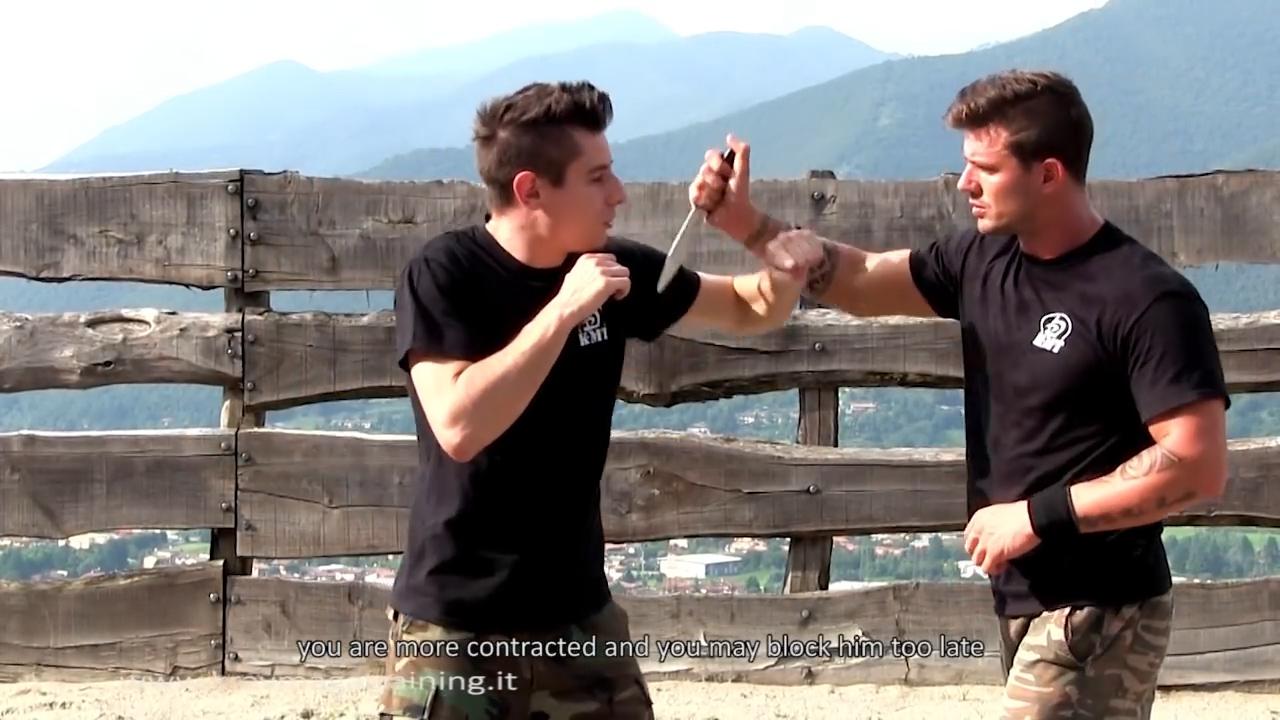

Supplement: Supplementary file 2 — Supplementary Information 2. [file 41598_2023_35190_MOESM2_ESM.zip › test/images/KravMagaTraining049_jpg.rf.4891779f8592e45c8054efda34c20a10.jpg]

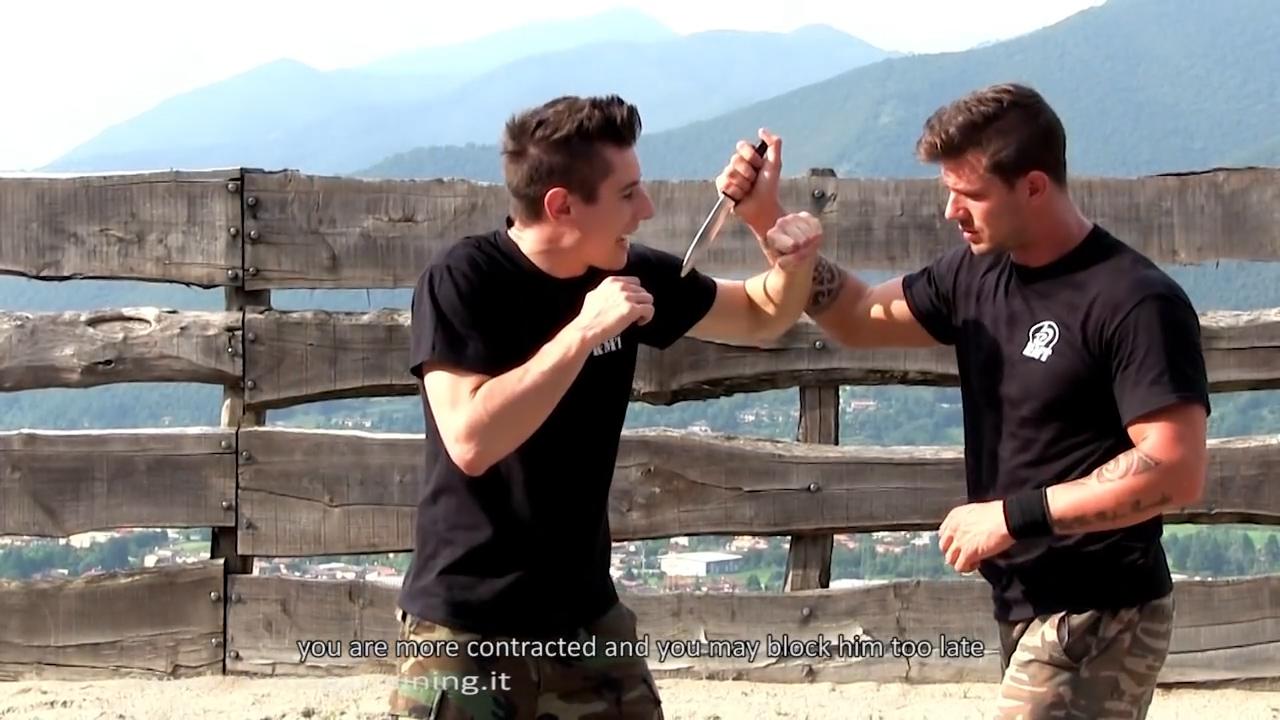

Supplement: Supplementary file 2 — Supplementary Information 2. [file 41598_2023_35190_MOESM2_ESM.zip › test/images/KravMagaTraining051_jpg.rf.6d2c69cc28202e0e6ed1ab8c7b2614ef.jpg]

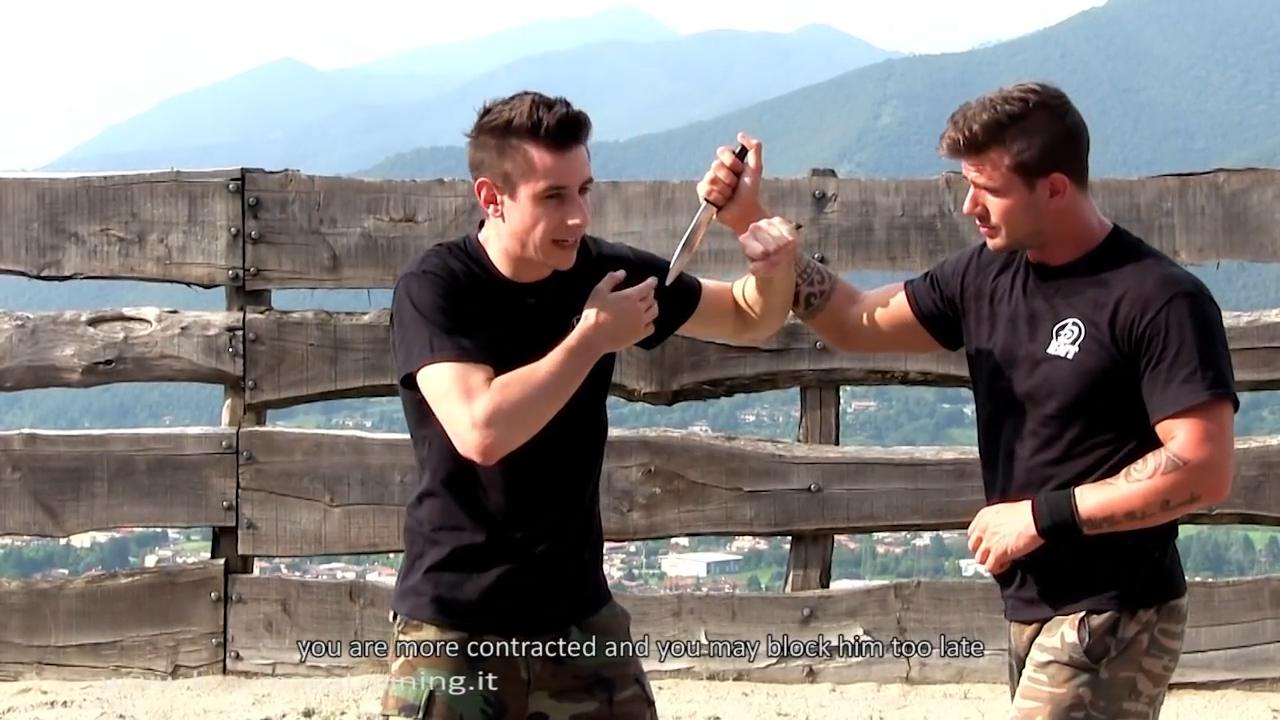

Supplement: Supplementary file 2 — Supplementary Information 2. [file 41598_2023_35190_MOESM2_ESM.zip › test/images/KravMagaTraining055_jpg.rf.3b1544dad096077b4cd689e3d68b8228.jpg]

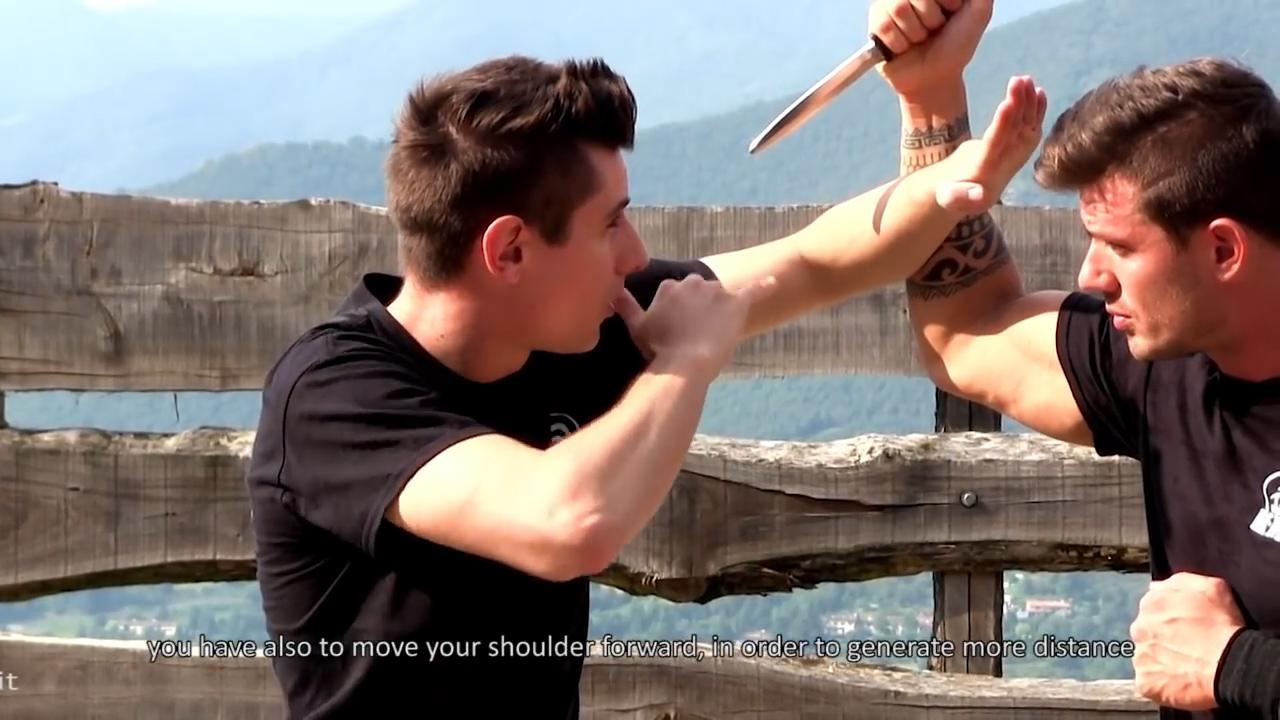

Supplement: Supplementary file 2 — Supplementary Information 2. [file 41598_2023_35190_MOESM2_ESM.zip › test/images/KravMagaTraining076_jpg.rf.f31d3ade8b214a579f4703a4734a3298.jpg]

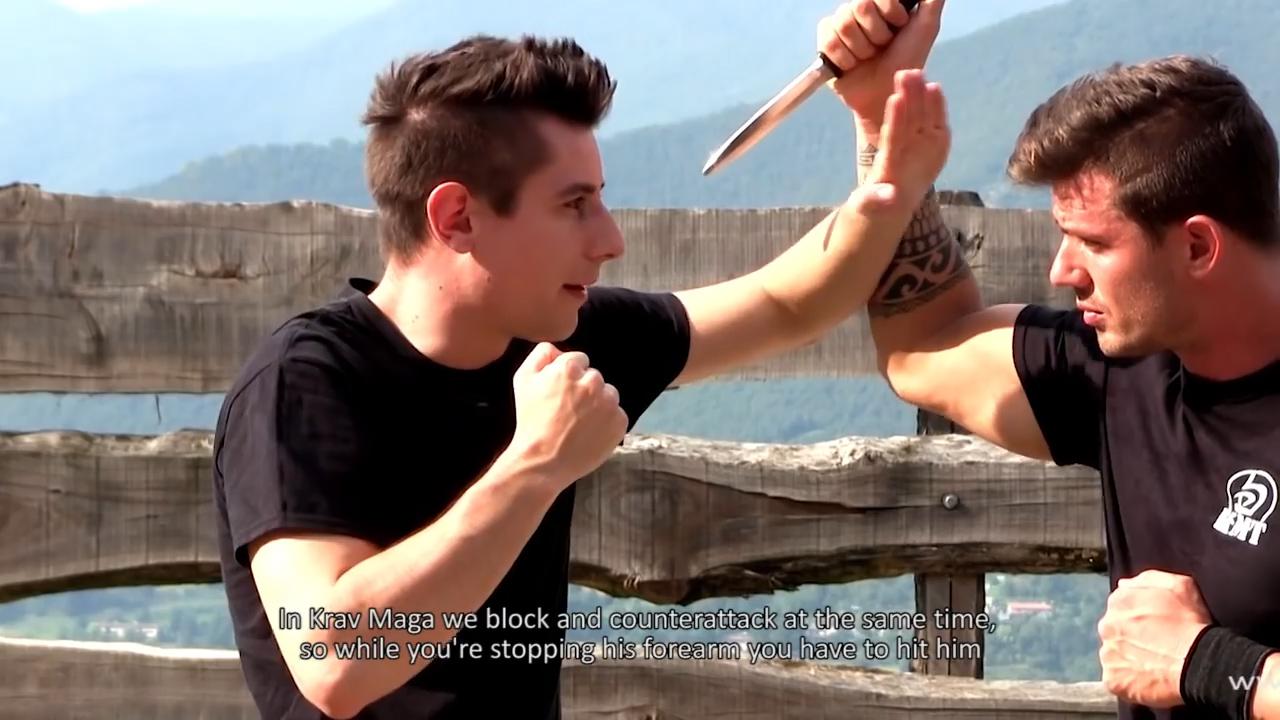

Supplement: Supplementary file 2 — Supplementary Information 2. [file 41598_2023_35190_MOESM2_ESM.zip › test/images/KravMagaTraining094_jpg.rf.ffe37ddbc0ad280057e75911a31f59c3.jpg]

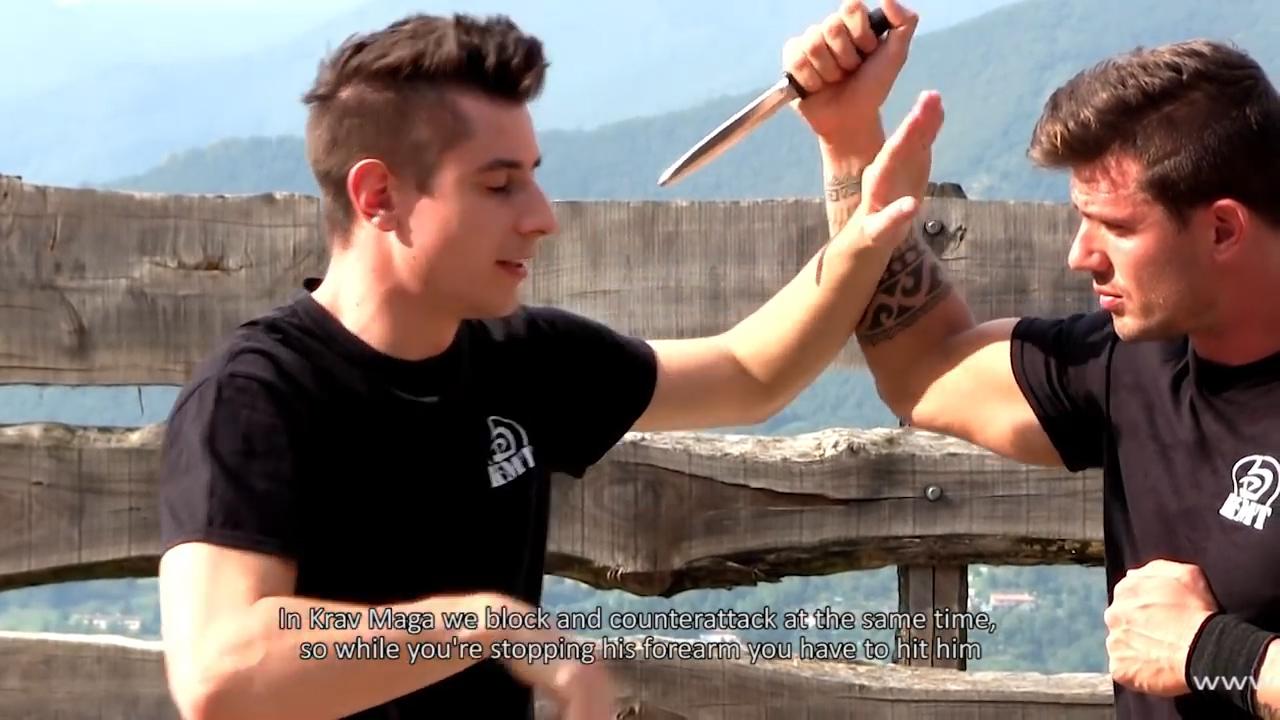

Supplement: Supplementary file 2 — Supplementary Information 2. [file 41598_2023_35190_MOESM2_ESM.zip › test/images/KravMagaTraining105_jpg.rf.d66ce8a451ef1d28996dc640557a012e.jpg]

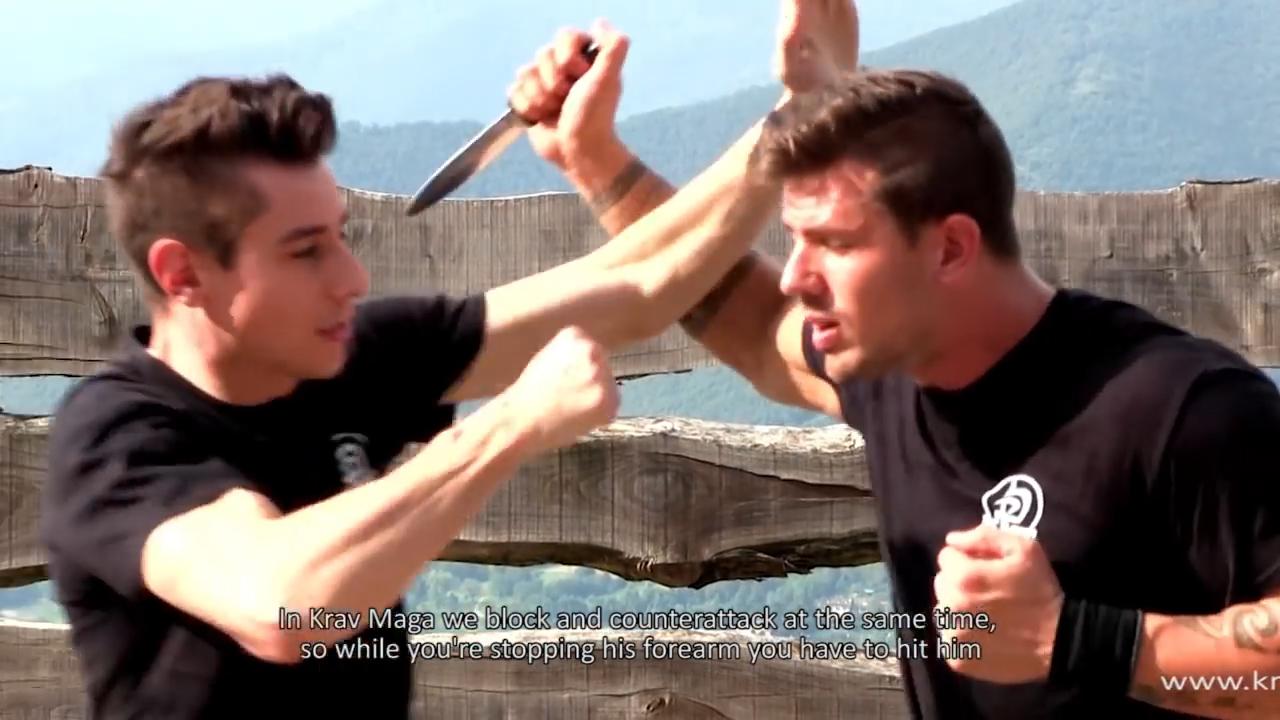

Supplement: Supplementary file 2 — Supplementary Information 2. [file 41598_2023_35190_MOESM2_ESM.zip › test/images/KravMagaTraining115_jpg.rf.74fe23b9ba969efc63110491c0d7e217.jpg]

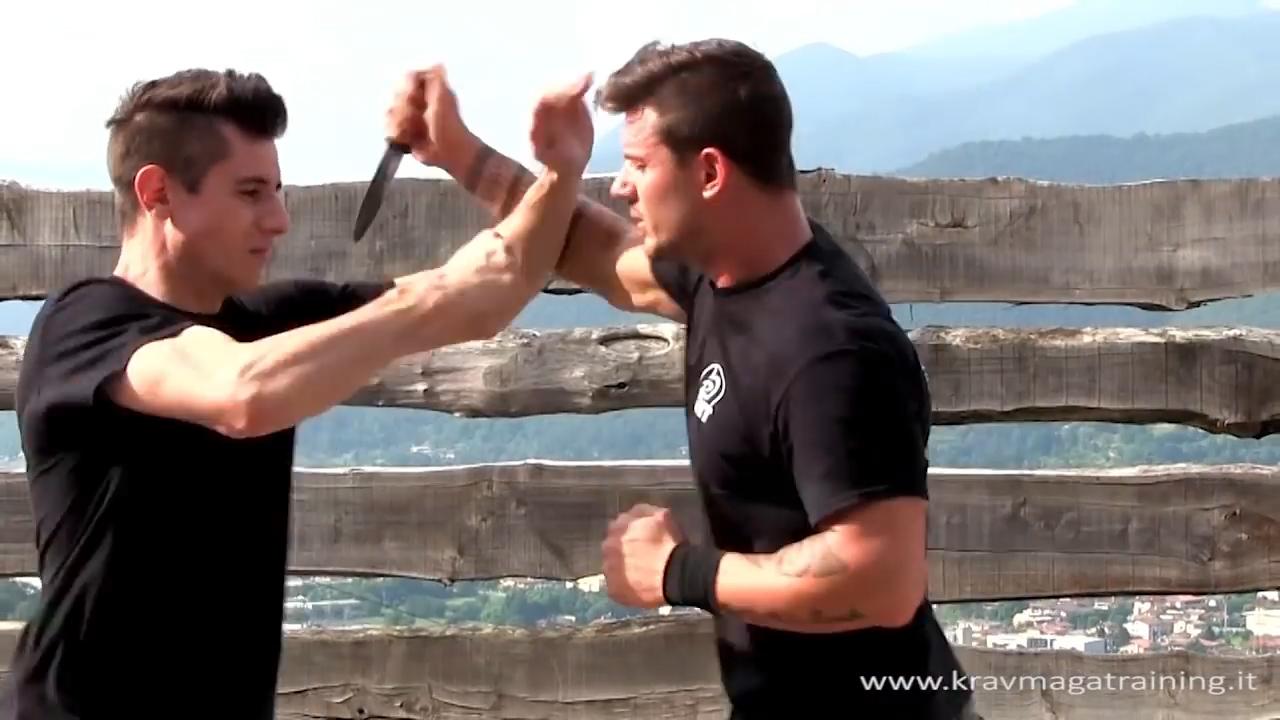

Supplement: Supplementary file 2 — Supplementary Information 2. [file 41598_2023_35190_MOESM2_ESM.zip › test/images/KravMagaTraining119_jpg.rf.3a33a6e11833e8316666aa8134248830.jpg]

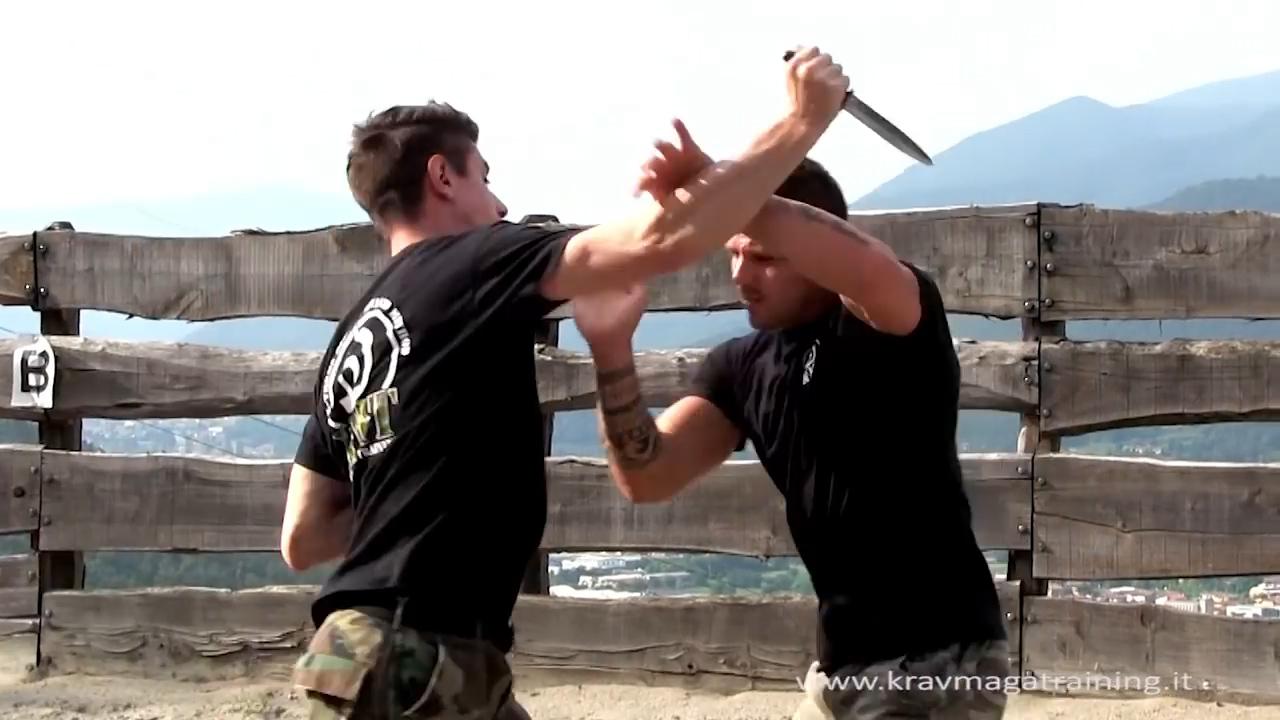

Supplement: Supplementary file 2 — Supplementary Information 2. [file 41598_2023_35190_MOESM2_ESM.zip › test/images/KravMagaTraining139_jpg.rf.47e6017be288bef8a26fddba658727e1.jpg]

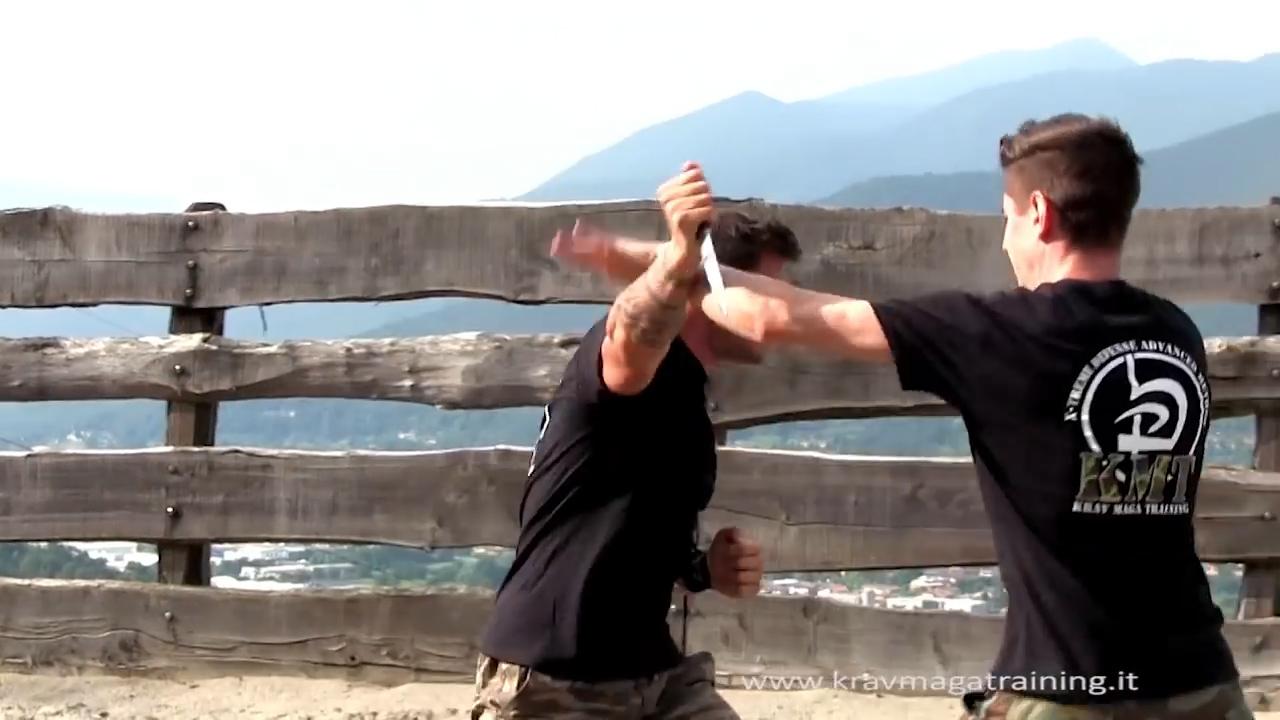

Supplement: Supplementary file 2 — Supplementary Information 2. [file 41598_2023_35190_MOESM2_ESM.zip › test/images/KravMagaTraining156_jpg.rf.da5f666e3899e9eb0cef72ca6917f026.jpg]

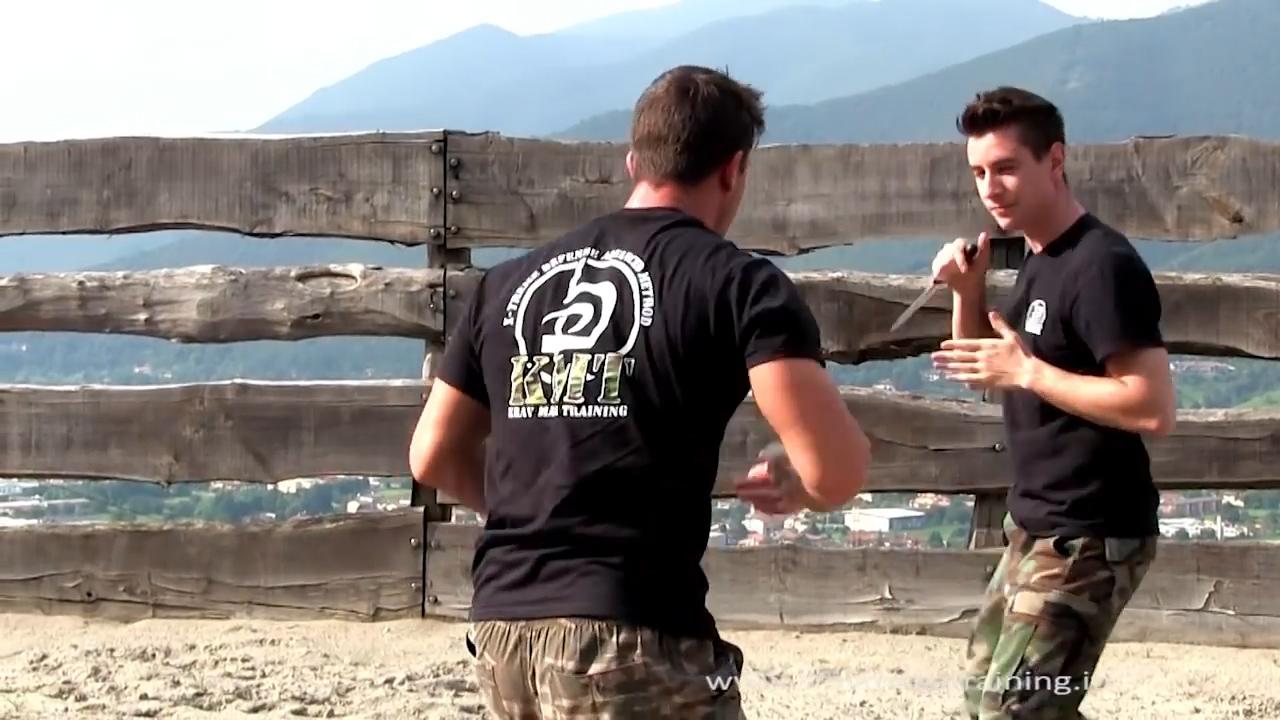

Supplement: Supplementary file 2 — Supplementary Information 2. [file 41598_2023_35190_MOESM2_ESM.zip › test/images/KravMagaTraining167_jpg.rf.15a7e49ee0fb663016bc27f38c4597b3.jpg]

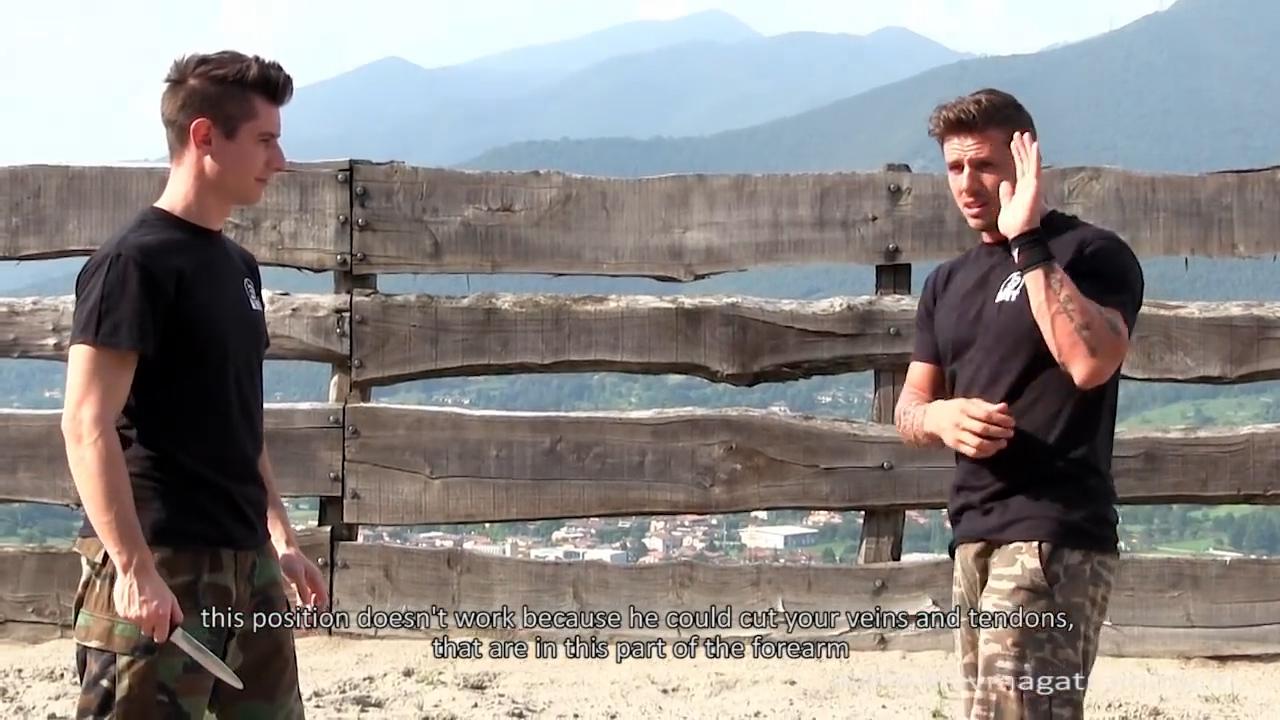

Supplement: Supplementary file 2 — Supplementary Information 2. [file 41598_2023_35190_MOESM2_ESM.zip › test/images/KravMagaTraining20041_jpg.rf.6f88a302f672948dc355e319ba0635f0.jpg]

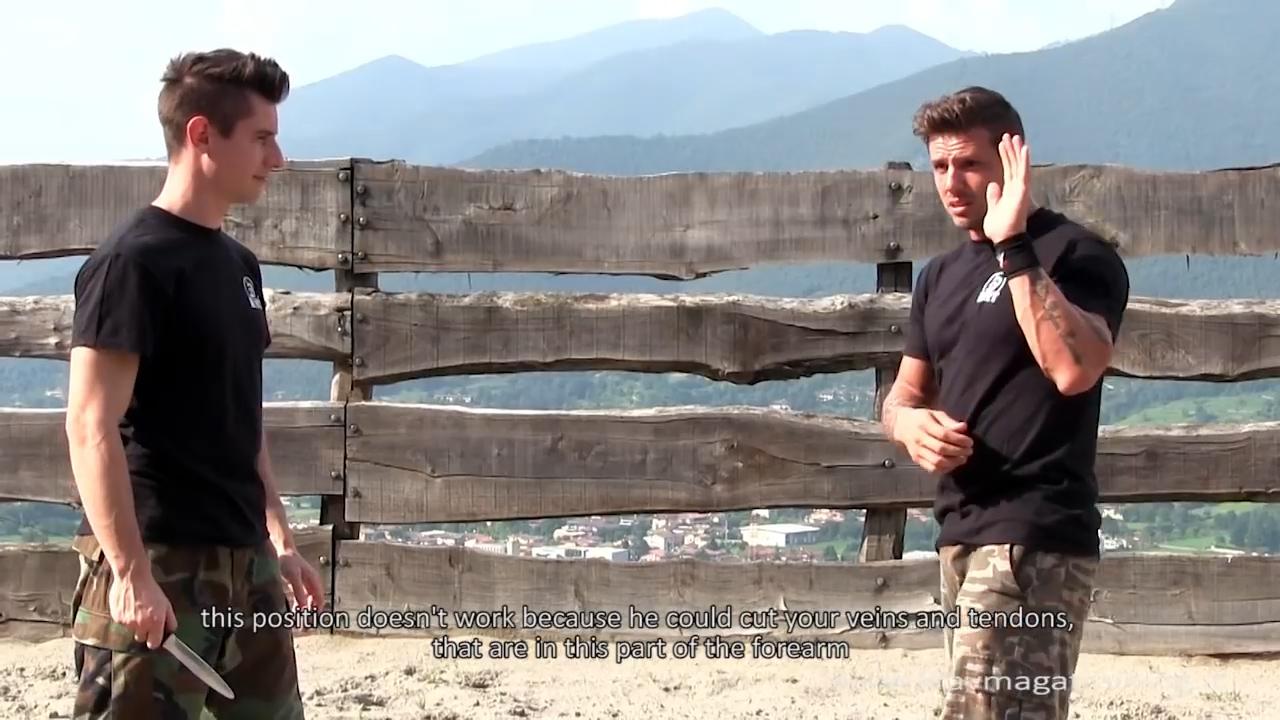

Supplement: Supplementary file 2 — Supplementary Information 2. [file 41598_2023_35190_MOESM2_ESM.zip › test/images/KravMagaTraining20042_jpg.rf.a12ae51ef3795a151a5468f386bdac9c.jpg]

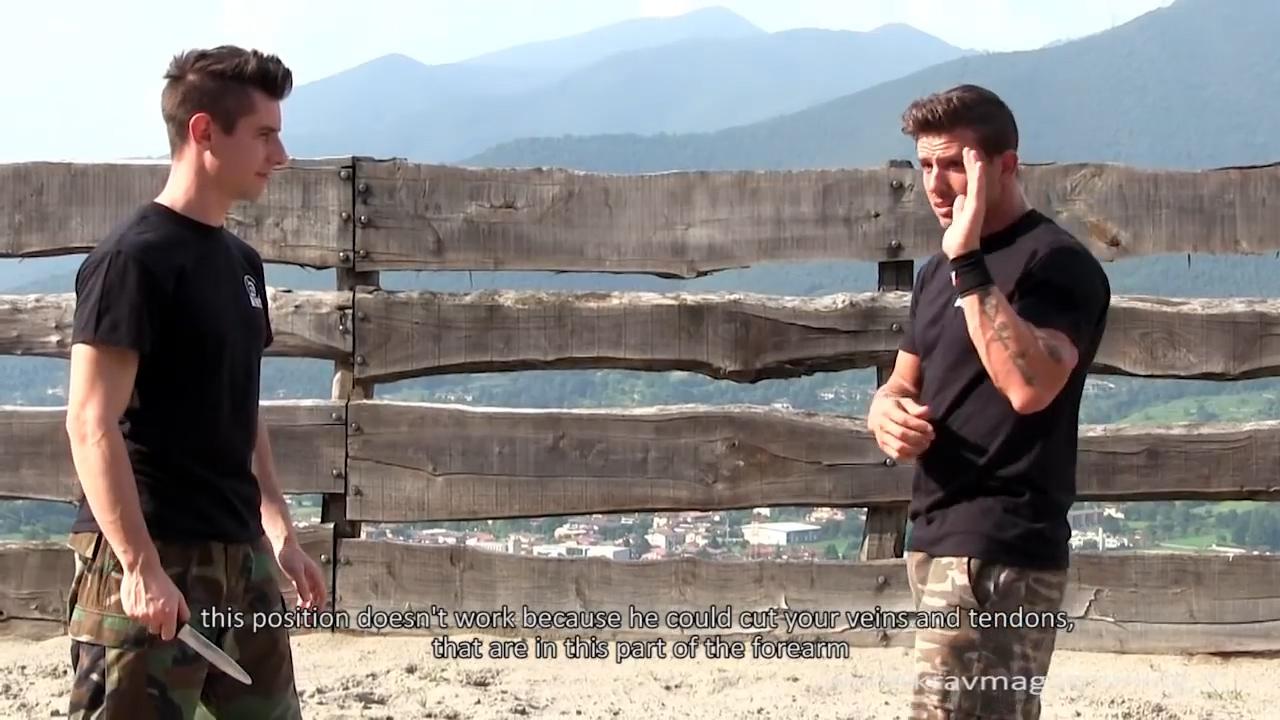

Supplement: Supplementary file 2 — Supplementary Information 2. [file 41598_2023_35190_MOESM2_ESM.zip › test/images/KravMagaTraining20043_jpg.rf.22f2cf47308fe7581ad4efcb9d99dff4.jpg]

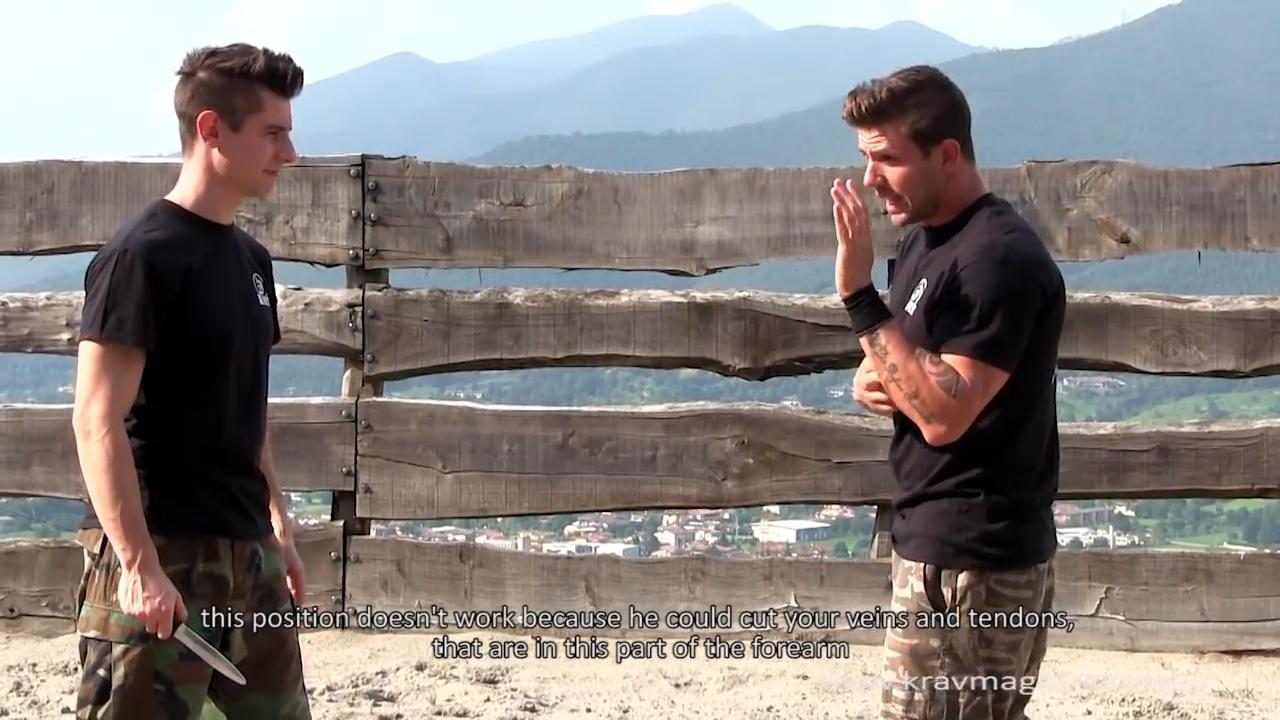

Supplement: Supplementary file 2 — Supplementary Information 2. [file 41598_2023_35190_MOESM2_ESM.zip › test/images/KravMagaTraining20046_jpg.rf.685432c2117b3acee8fbd27789cfb1ba.jpg]

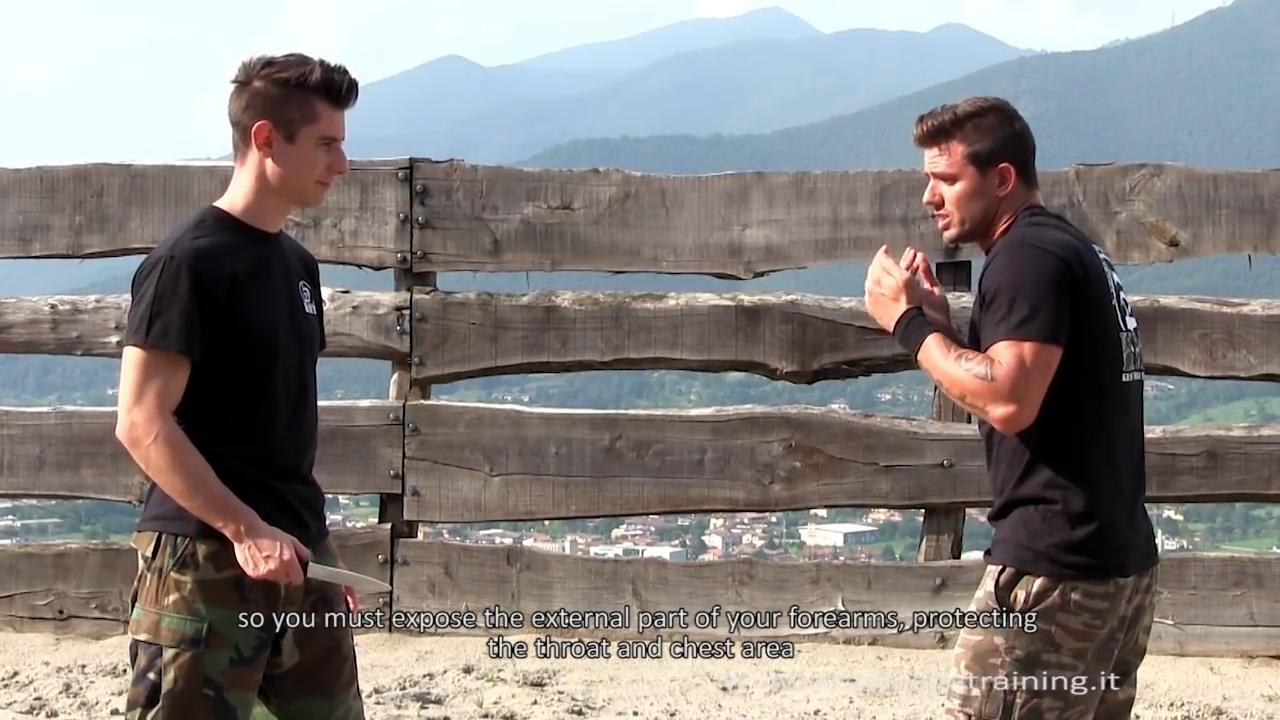

Supplement: Supplementary file 2 — Supplementary Information 2. [file 41598_2023_35190_MOESM2_ESM.zip › test/images/KravMagaTraining20072_jpg.rf.47a1ad2461e1c296d6c0f7b92039eb5e.jpg]

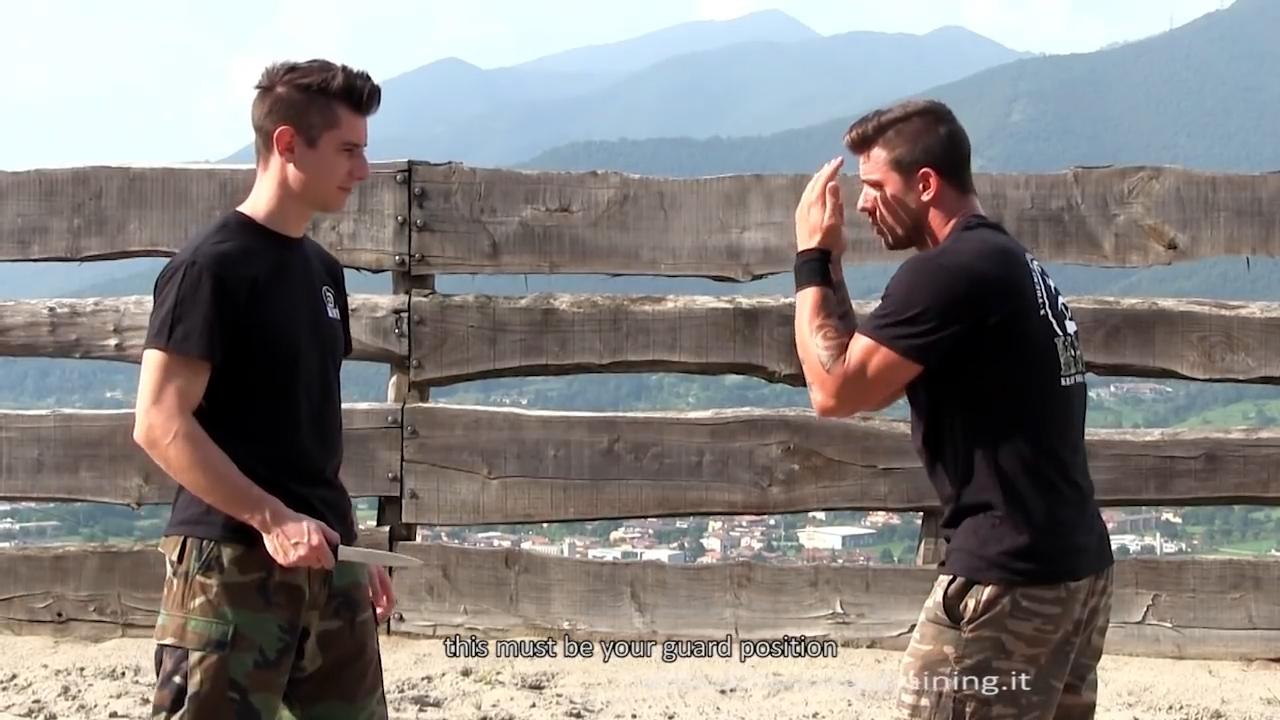

Supplement: Supplementary file 2 — Supplementary Information 2. [file 41598_2023_35190_MOESM2_ESM.zip › test/images/KravMagaTraining20098_jpg.rf.6dd224df87d7973a9c450cca0fade0e2.jpg]

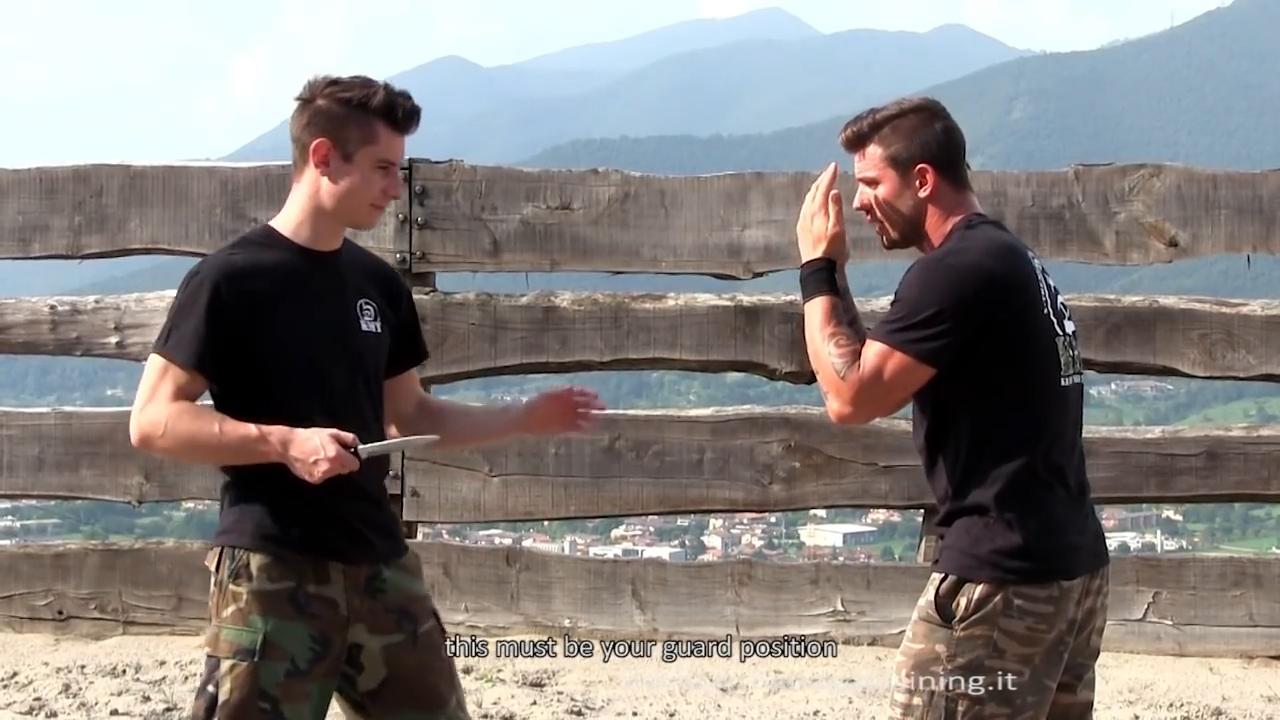

Supplement: Supplementary file 2 — Supplementary Information 2. [file 41598_2023_35190_MOESM2_ESM.zip › test/images/KravMagaTraining20102_jpg.rf.555872f25f7662469331feb31a182653.jpg]

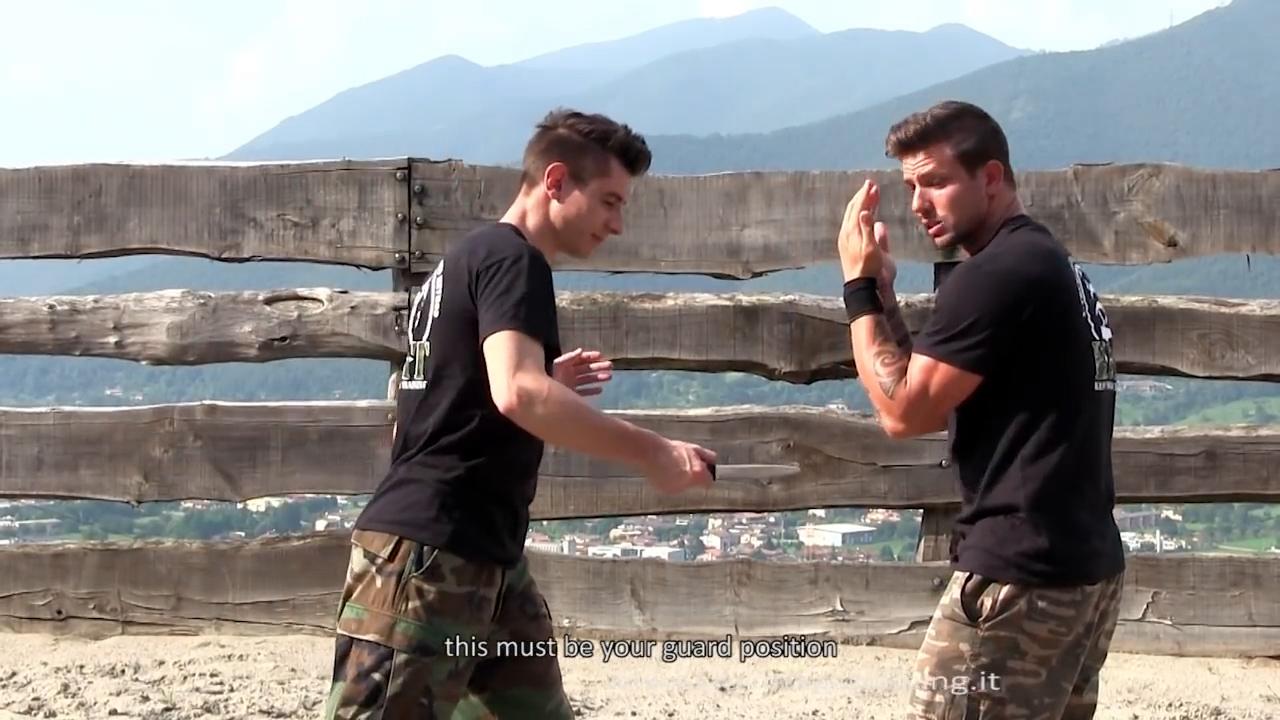

Supplement: Supplementary file 2 — Supplementary Information 2. [file 41598_2023_35190_MOESM2_ESM.zip › test/images/KravMagaTraining20107_jpg.rf.2df7e6dea2c54843cfb6db0ae5d19917.jpg]

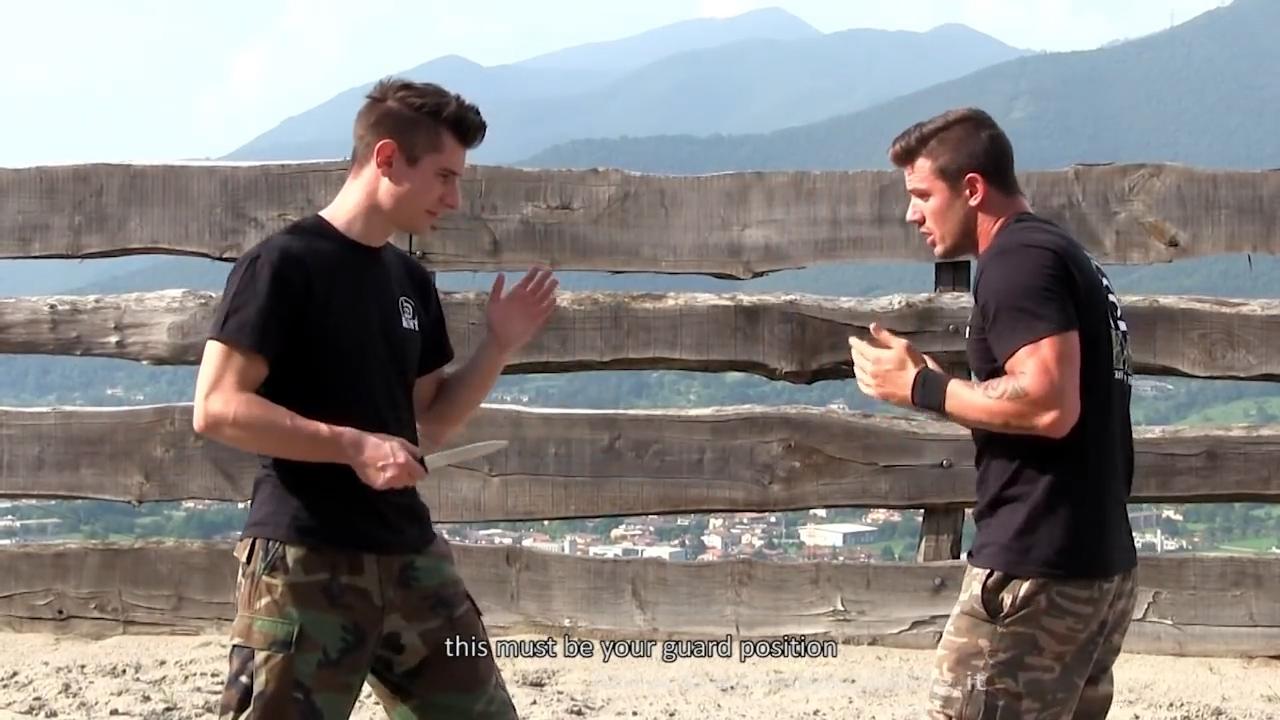

Supplement: Supplementary file 2 — Supplementary Information 2. [file 41598_2023_35190_MOESM2_ESM.zip › test/images/KravMagaTraining20111_jpg.rf.088988454860d962895669a42378486b.jpg]

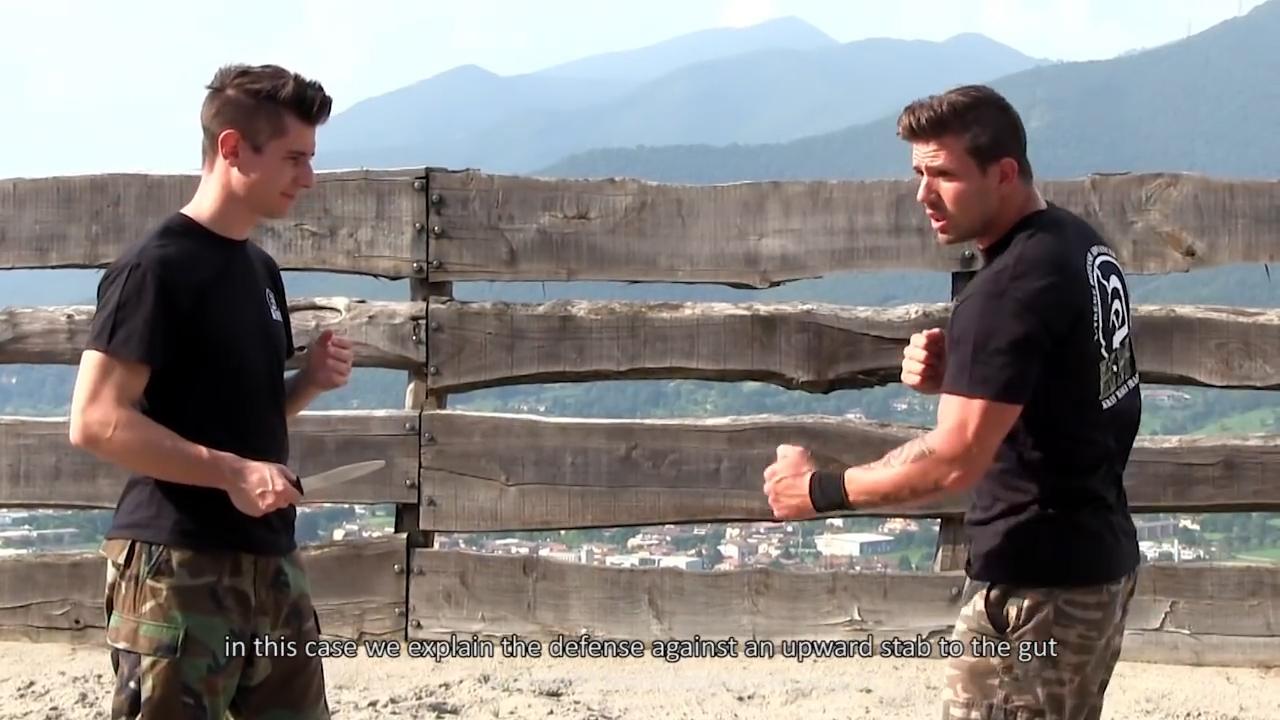

Supplement: Supplementary file 2 — Supplementary Information 2. [file 41598_2023_35190_MOESM2_ESM.zip › test/images/KravMagaTraining20143_jpg.rf.01eb08bb70a7ce13263500f70926d7f3.jpg]

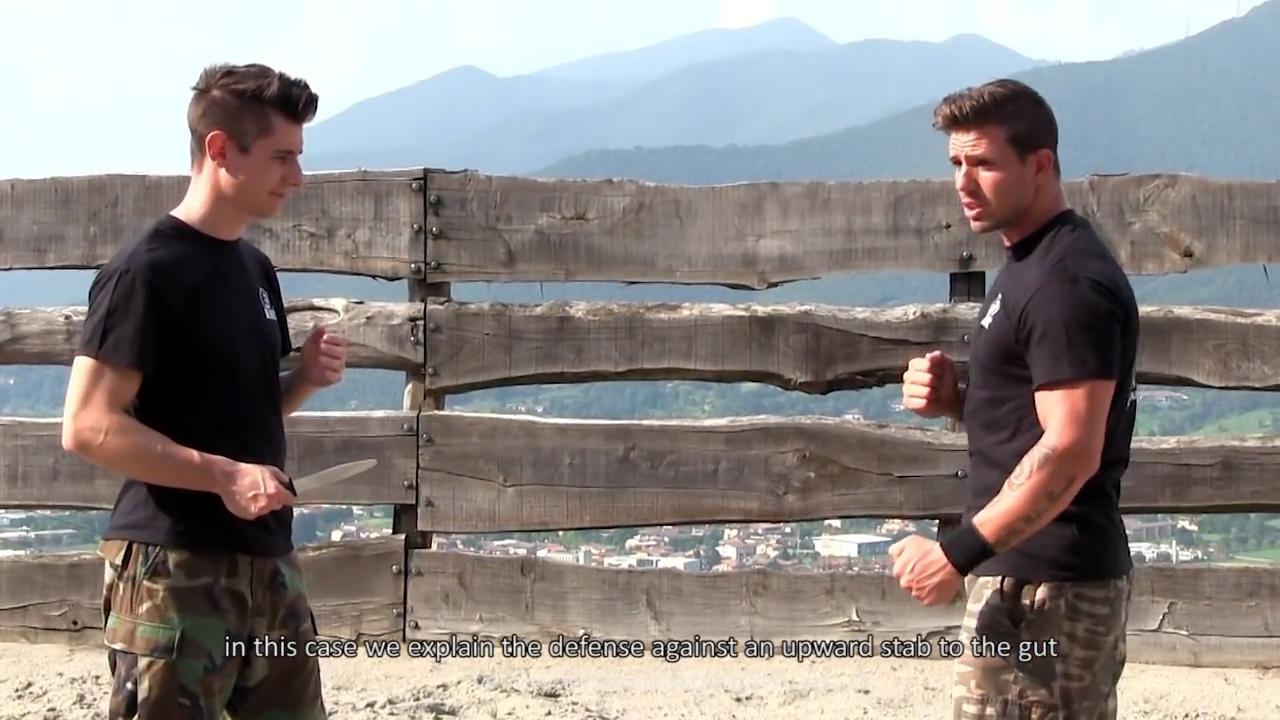

Supplement: Supplementary file 2 — Supplementary Information 2. [file 41598_2023_35190_MOESM2_ESM.zip › test/images/KravMagaTraining20145_jpg.rf.399fce93f256e603b15f37328483dac2.jpg]

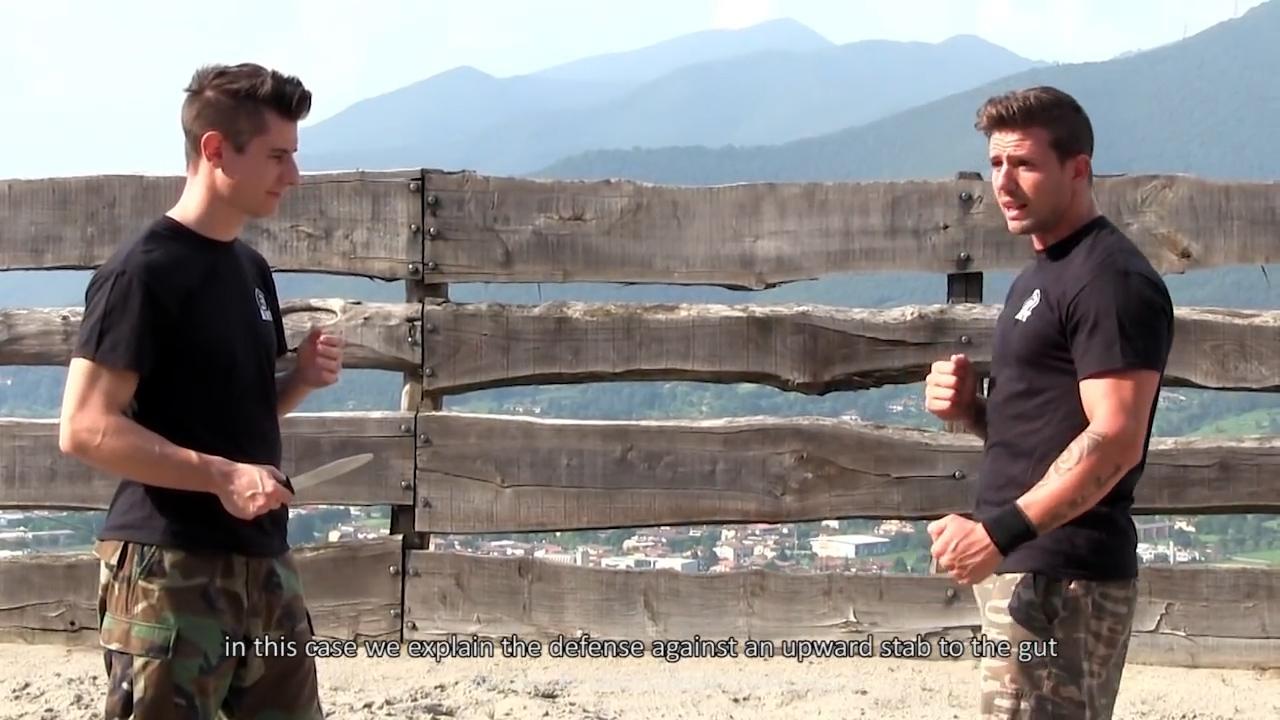

Supplement: Supplementary file 2 — Supplementary Information 2. [file 41598_2023_35190_MOESM2_ESM.zip › test/images/KravMagaTraining20147_jpg.rf.d56d3869c0a7e17f3cf3041962f26bb0.jpg]

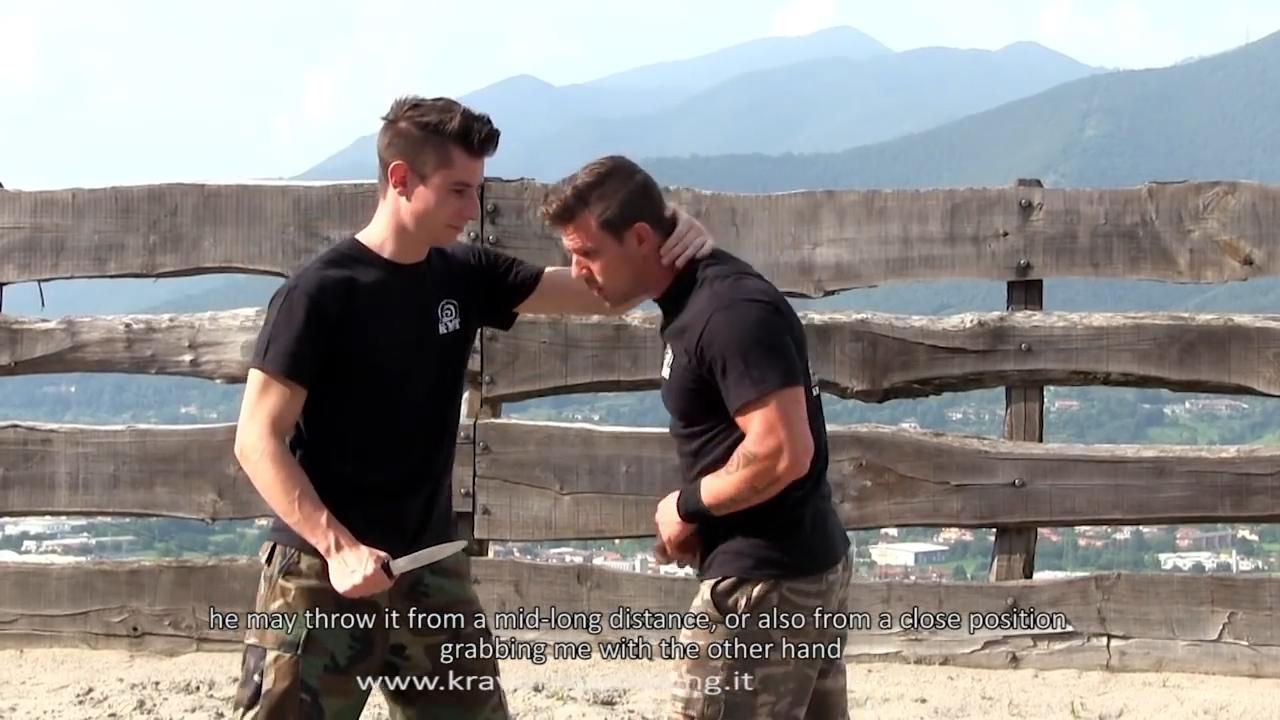

Supplement: Supplementary file 2 — Supplementary Information 2. [file 41598_2023_35190_MOESM2_ESM.zip › test/images/KravMagaTraining20179_jpg.rf.37b5001f2dd930d970c0a30b0de20a31.jpg]

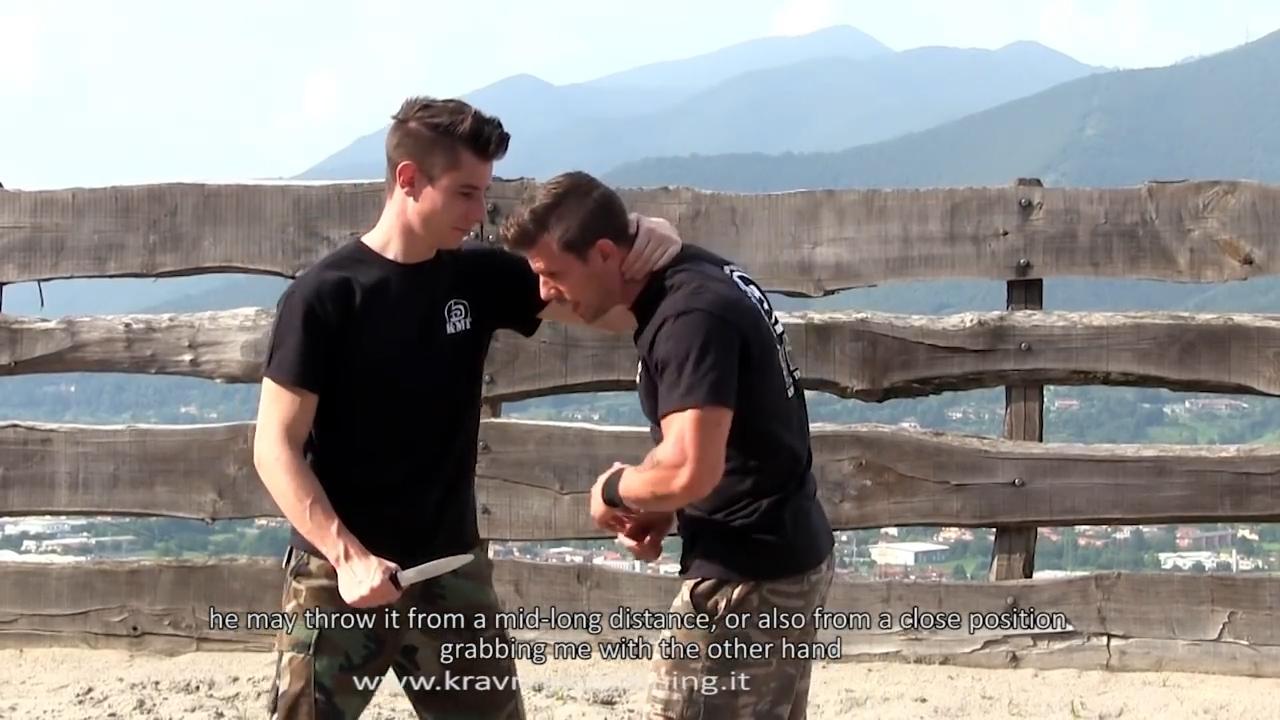

Supplement: Supplementary file 2 — Supplementary Information 2. [file 41598_2023_35190_MOESM2_ESM.zip › test/images/KravMagaTraining20180_jpg.rf.5ba7f1710b06afcb16da35ad820e4562.jpg]

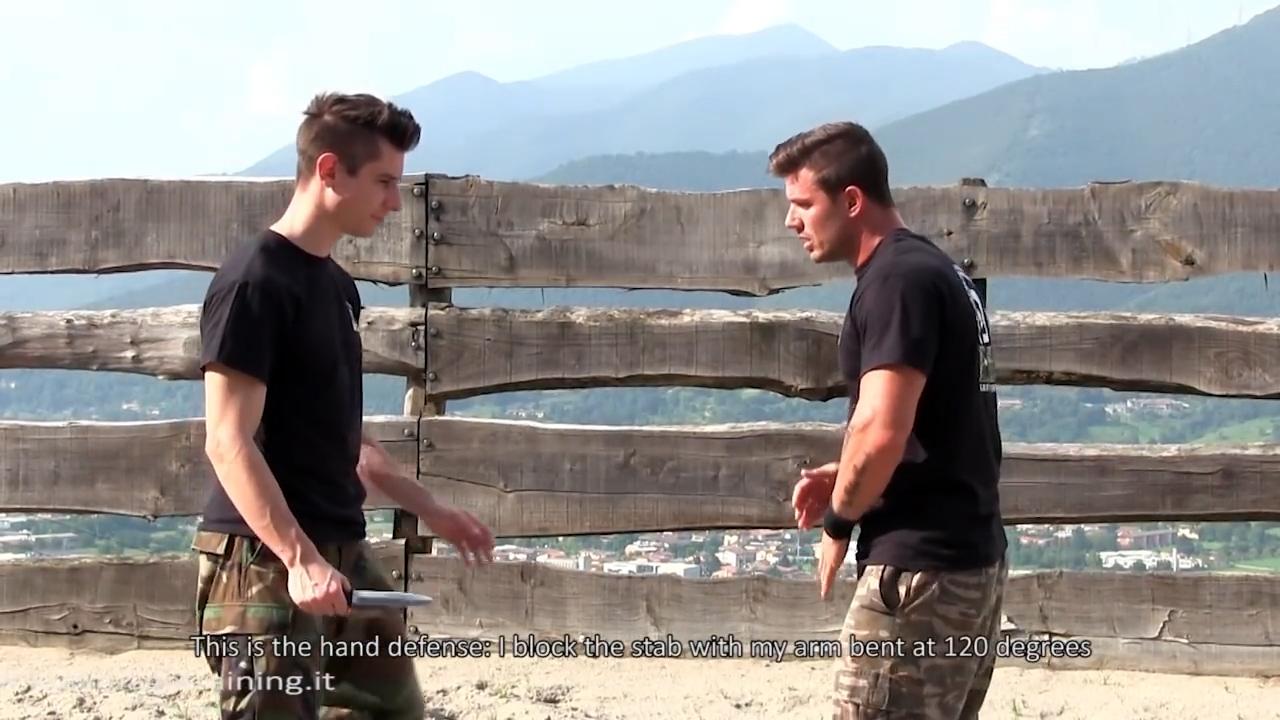

Supplement: Supplementary file 2 — Supplementary Information 2. [file 41598_2023_35190_MOESM2_ESM.zip › test/images/KravMagaTraining20301_jpg.rf.66157b3d539cee7845221216f6a4f0c7.jpg]

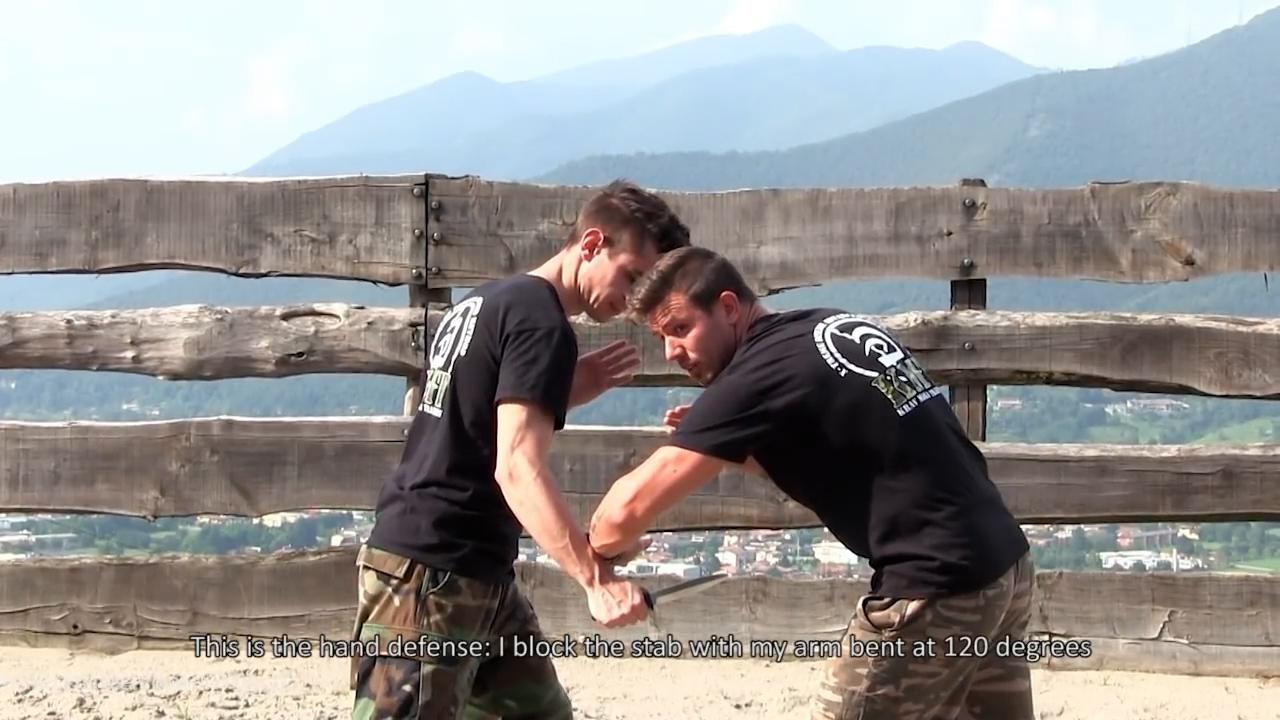

Supplement: Supplementary file 2 — Supplementary Information 2. [file 41598_2023_35190_MOESM2_ESM.zip › test/images/KravMagaTraining20314_jpg.rf.ca49112fb875a6aa30467c69644efe38.jpg]

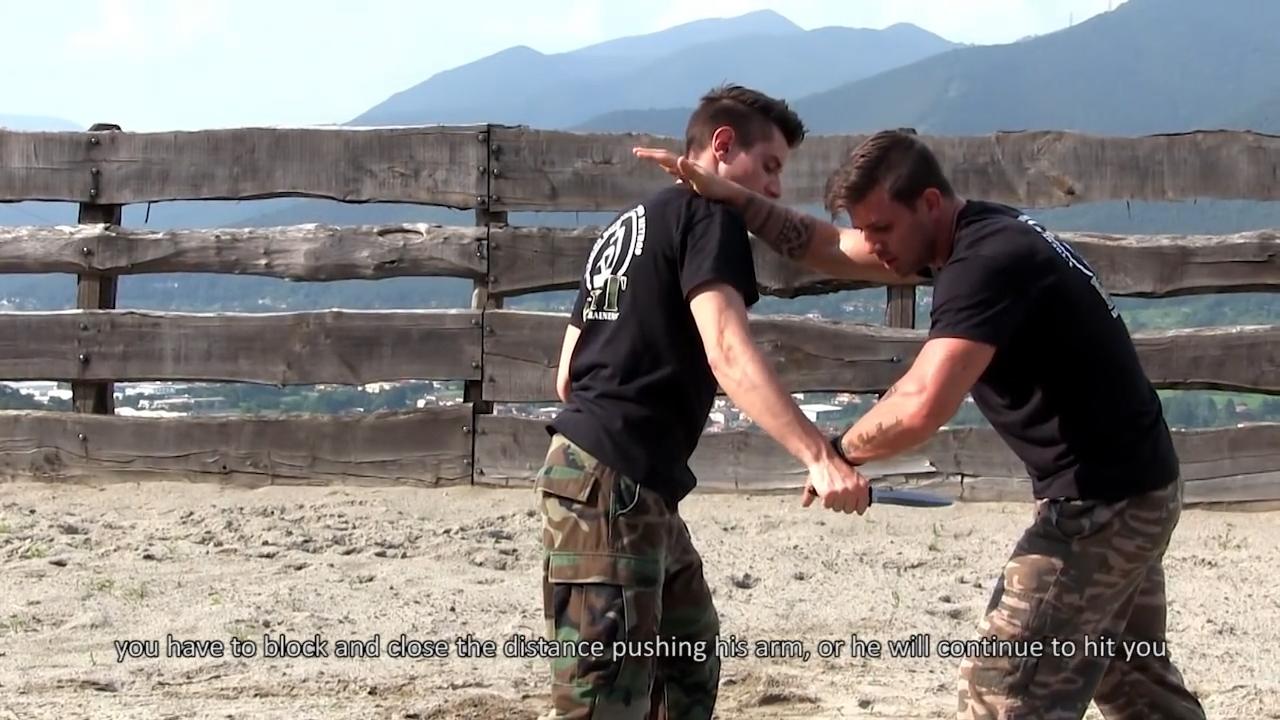

Supplement: Supplementary file 2 — Supplementary Information 2. [file 41598_2023_35190_MOESM2_ESM.zip › test/images/KravMagaTraining20340_jpg.rf.5abf733342786ef1bcbfc8210f27665a.jpg]

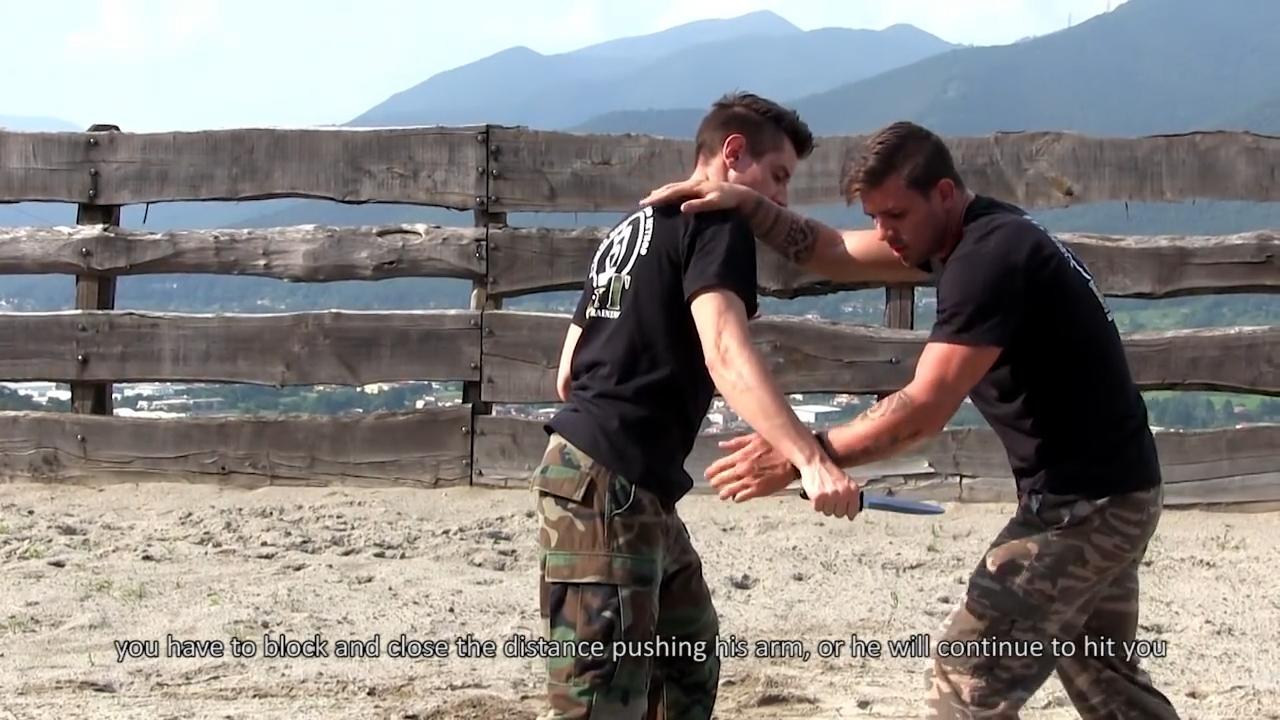

Supplement: Supplementary file 2 — Supplementary Information 2. [file 41598_2023_35190_MOESM2_ESM.zip › test/images/KravMagaTraining20342_jpg.rf.6fd0d08c8a3fe4ba4395a1b2968ed072.jpg]

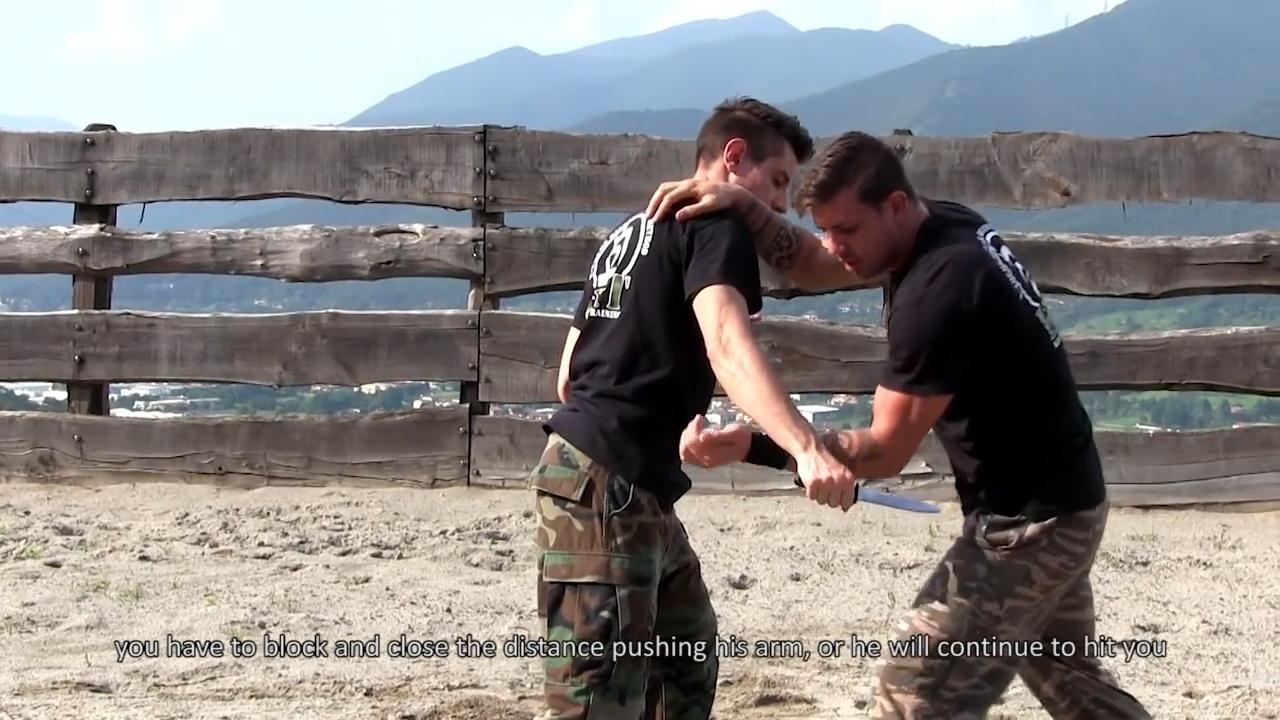

Supplement: Supplementary file 2 — Supplementary Information 2. [file 41598_2023_35190_MOESM2_ESM.zip › test/images/KravMagaTraining20343_jpg.rf.21d6808b675b82b717405c4aef7245a7.jpg]

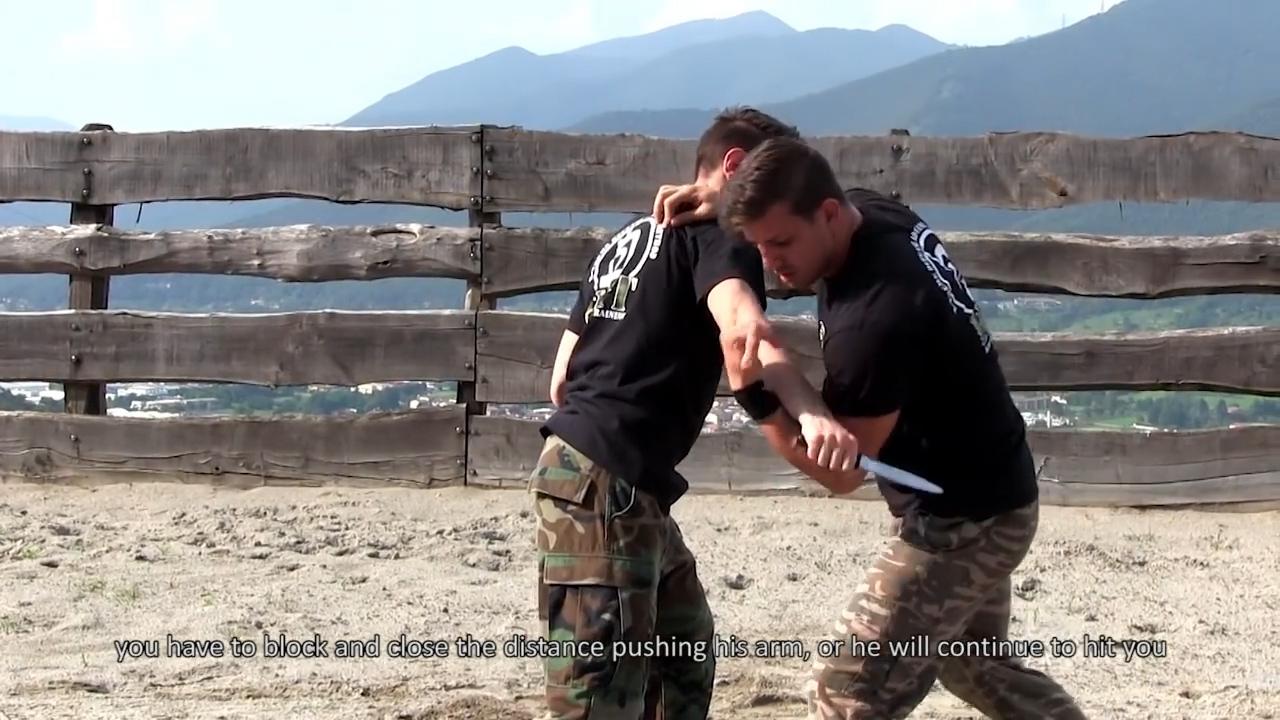

Supplement: Supplementary file 2 — Supplementary Information 2. [file 41598_2023_35190_MOESM2_ESM.zip › test/images/KravMagaTraining20344_jpg.rf.19ae321c911e85adacf21009dc821833.jpg]

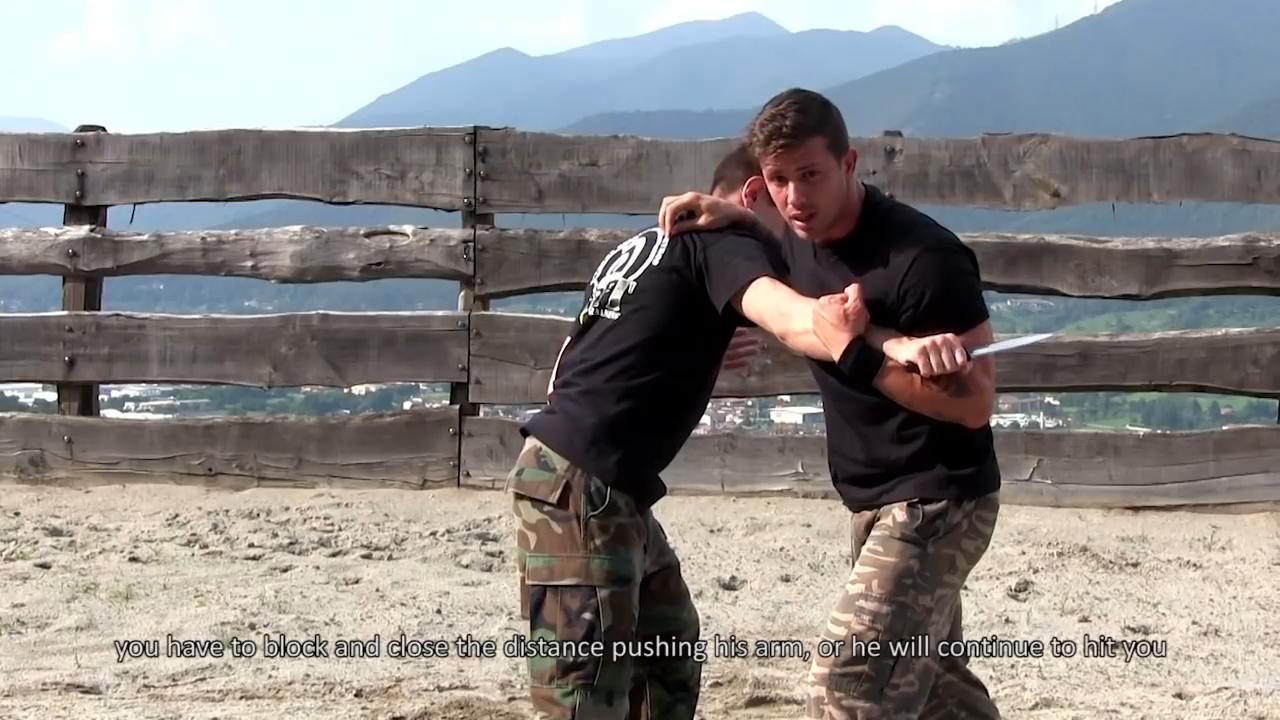

Supplement: Supplementary file 2 — Supplementary Information 2. [file 41598_2023_35190_MOESM2_ESM.zip › test/images/KravMagaTraining20348_jpg.rf.3855667cf95d12645c22142fd0ce736b.jpg]
